# Exploring the oral microbiota of children at various developmental stages of their dentition in the relation to their oral health

**DOI:** 10.1186/1755-8794-4-22

**Published:** 2011-03-04

**Authors:** Wim Crielaard, Egija Zaura, Annemarie A Schuller, Susan M Huse, Roy C Montijn, Bart JF Keijser

**Affiliations:** 1 Department of Experimental Preventive Dentistry, Academic Centre for Dentistry Amsterdam (ACTA), University of Amsterdam and Free University Amsterdam, Gustav Mahlerlaan 3004, 1081 LA Amsterdam, the Netherlands; 2 TNO Quality of Life, PO box 2215, 2301 CE Leiden, the Netherlands; 3 Josephine Bay Paul Center for Comparative Molecular Biology and Evolution, Marine Biological Laboratory, 7 MBL Street, Woods Hole, MA, USA; 4 TNO Quality of Life, Business Unit Food and Biotechnology Innovations, Microbial Genomics Group, Utrechtseweg 48, 3704 HE Zeist, the Netherlands

## Abstract

**Background:**

An understanding of the relation of commensal microbiota to health is essential in preventing disease. Here we studied the oral microbial composition of children (N = 74, aged 3 - 18 years) in natural transition from their deciduous to a permanent dentition and related the microbial profiles to their oral health status. The microbial composition of saliva was assessed by barcoded pyrosequencing of the V5-V6 hypervariable regions of the 16 S rRNA, as well as by using phylogenetic microarrays.

**Results:**

Pyrosequencing reads (126174 reads, 1045 unique sequences) represented 8 phyla and 113 higher taxa in saliva samples. Four phyla - Firmicutes, Bacteriodetes, Proteobacteria and Actinobacteria - predominated in all groups. The deciduous dentition harboured a higher proportion of Proteobacteria (Gammaproteobacteria, Moraxellaceae) than Bacteroidetes, while in all other groups Bacteroidetes were at least as abundant as Proteobacteria. Bacteroidetes (mainly genus *Prevotella*), Veillonellaceae family, Spirochaetes and candidate division TM7 increased with increasing age, reflecting maturation of the microbiome driven by biological changes with age.

Microarray analysis enabled further analysis of the individual salivary microbiota. Of 350 microarray probes, 156 gave a positive signal with, on average, 77 (range 48-93) probes per individual sample.

A caries-free oral status significantly associated with the higher signal of the probes targeting *Porphyromonas catoniae *and *Neisseria flavescens*.

**Conclusions:**

The potential role of *P. catoniae *and *N. flavescens *as oral health markers should be assessed in large-scale clinical studies. The combination of both, open-ended and targeted molecular approaches provides us with information that will increase our understanding of the interplay between the human host and its microbiome.

## Background

The oral cavity is a complex ecological niche, as is reflected by its complex microbial community. Recent advances in sequencing technology, such as 454 pyrosequencing, have revealed an unexpectedly high diversity of the human oral microbiome: dental plaque pooled from 98 healthy adults comprised about 10000 microbial phylotypes [1]. This is an order of magnitude higher than previously reported 700 oral microbial phylotypes as identified by cultivation or traditional cloning and sequencing [2].

Studies have revealed that the various sites in the oral cavity, such as the hard dental surfaces, mucosal sites, and anaerobic pockets, house unique microbial communities [3,4]. The oral ecological system is dramatically influenced by chemical and physical fluctuations as a result of food and drink intake and oral hygiene measures. Nevertheless studies performed so far have revealed a relatively stable oral microbial community that shows fewer differences between individuals as compared to the skin or gastrointestinal microbiomes [5]. The oral microbiota is intimately related to oral health. It is generally accepted that a shift in microbial composition is an important step in the progression of oral disease. However, there are few studies that have actually demonstrated this ecological shift.

For an individual, birth is a borderline between the sterile intrauterine life and the extrauterine existence with a continuous exposure to microorganisms [6]. Microbiota are acquired via other individuals, animals and the local environment [7]. The microbial community is further shaped by diet and personal oral hygiene.

Here we explored the effects of natural changes in the oral ecosystem during the transition from the deciduous to the permanent dentition of children on the microbial composition of their saliva. We started with an open-ended approach: we sequenced tagged 16 S amplicons of pooled saliva samples using 454 pyrosequencing technology. Then, by using a phylogenetic microarray that targets small subunit (16S) ribosomal DNA of the predominant oral microorganisms, we obtained individual profiles of each saliva sample. To assess the relation of the oral health status of each child with their microbial profile, we quantified the amount of certain oral pathogens and health-associated microorganisms by using a q-PCR technique and compared that with the obtained epidemiological data.

## Results

### Clinical findings

We collected saliva from 74 children, aged 3-18 years old, and recorded their oral health status through clinical epidemiological examinations. Of these, 35 children had at least one sibling who also participated in the study. Children's saliva samples were divided into groups according to four stages of their dentition: 1) deciduous (milk) dentition, 2) early mixed dentition (only permanent front teeth and/or the first permanent molars were erupted or were in the process of eruption), 3) late mixed dentition (cuspids, premolars and/or second molars were in the process of eruption) and 4) permanent dentition (Table 1). According to their oral health status, three groups of children were defined - 'healthy' or caries-free children (N = 27), 'treated' or children who have had caries lesions in the past and have completed their dental treatment (N = 25), and a 'caries' group or children with active dental decay present at the time of examination (N = 22). The plaque amount (Table 1) showed a trend to decrease with increasing age (Pearson correlation, *p *= 0.04, r = -0.239) and correlated with the number of carious surfaces (*p *= 0.026, r = 0.259). The highest caries experience was observed in the group with early mixed dentition (Table 1).

**Table 1:** Distribution of children into four subgroups according to the stage of their dentition and the clinical findings.

Dentition	Nr of children	Age in years range, mean (s.d.)	Nr of caries-free children	Nr of children with caries	dmfs* mean (s.d.)	DMFS* mean (s.d.)	Plaque Index** Mean (s.d.)
Deciduous	13	3-6, 4.4 (0.8)	6	5	3.8 (4.9)	0	1.0 (0.5)

Early Mixed	29	5-11, 7.8 (1.6)	10	9	7.9 (10.8)	1.1 (2.7)	0.9 (0.5)

Late Mixed	21	8-12, 10.1 (1.2)	8	6	6.1 (13.0)	1.6 (3.1)	0.9 (0.4)

Permanent	11	11-18, 13.9 (2.4)	3	2	0	2.6 (2.9)	0.7 (0.3)

* - DMFS/dmfs - number of decayed, missing and filled surfaces in deciduous (dmfs) or permanent (DMFS) dentition.

** - Plaque Index - mean plaque score, which is a measure of plaque amount.

### Microbiome analysis

**Figure 1:**
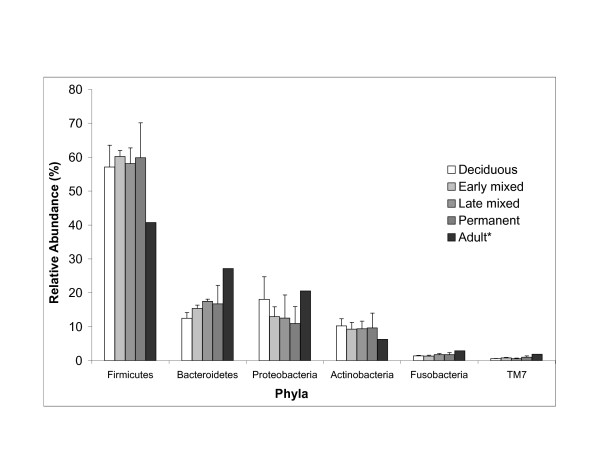
**Relative abundance of the main bacterial phyla (99.9% of the reads) identified in saliva of children at various developmental stages of their dentition**. Average and standard deviations from three pools of samples - healthy, treated and caries - per each developmental stage. * - Adult saliva data are added for comparison from Keijser et al. 2008 [1].

The oral microbiota were examined by parallel pyrosequencing of the 16 S rRNA gene on a GS FLX. In a bar-coding approach, 12 distinct pools of samples were sequenced and analyzed - deciduous, early mixed, late mixed, and permanent dentition at healthy, treated or caries state. Of all reads, 130572 reads (78.3%) passed quality filtering and 126174 reads remained after a cut-off of 5 reads per sequence was applied. On average, 10515 reads per sample pool were analyzed. A total of 1045 unique sequences were identified. The sequences represented 8 phyla and 113 higher taxa (genus or more inclusive taxa when sequences could not be confidently classified to the genus level) (Additional file 1). Four phyla - Firmicutes, Bacteriodetes, Proteobacteria and Actinobacteria - predominated in all groups (Figure 1), while three phyla - Fusobacteria, candidate division TM7 *incertae sedis *and Spirochaetes - were found in relatively low proportions. Representatives of the eighth phylum, Cyanobacteria, possibly reflecting plant chloroplasts, were found only sporadically, with the highest number of reads in the pool of late mixed dentition with caries (Additional file 1).

The deciduous dentition harboured a relatively high proportion of Proteobacteria compared to Bacteroidetes. In all other groups, the Bacteroidetes were found to be more abundant than Proteobacteria. In comparison to adult saliva [1], child saliva appears to have a higher proportion of Firmicutes and Actinobacteria and a lower proportion of Bacteroidetes, Fusobacteria, TM7 (Figure 1) and Spirochaetes at all stages of the dentition.

**Figure 2:**
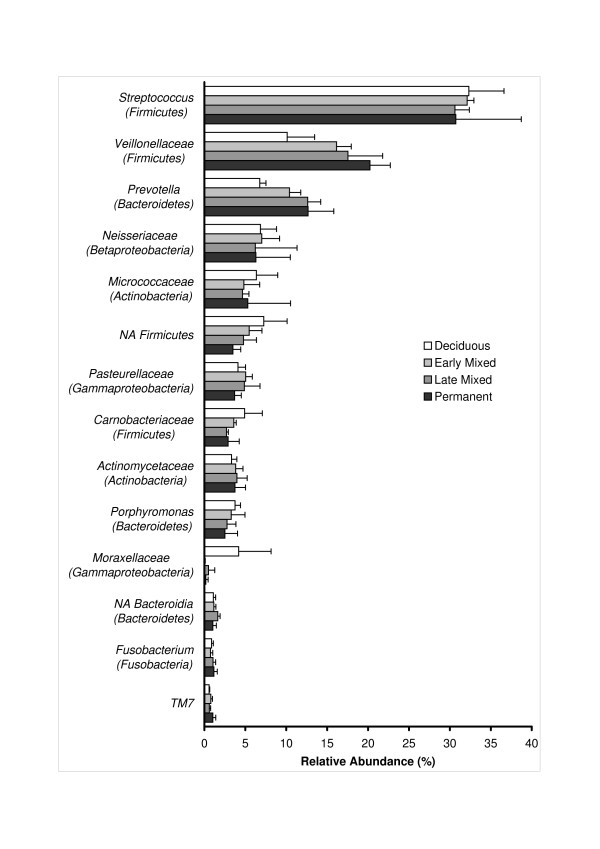
**Relative abundance of the predominant (92-95% of the reads) bacterial groups (family or genus if only one genus comprised the family, or unclassifiable bacteria within one phylum) in saliva samples of children at various developmental stages of the dentition**. Average and standard deviations from three pools of samples - healthy, treated and caries - per each developmental stage. NA - not assigned.

Representatives of the *Veillonellaceae *family (unclassified *Veillonellaceae*, *Veillonella *and *Selenomonas*) and the genus *Prevotella *increased with increasing age (Figure 2), while *Carnobacteriaceae *(mainly representatives of genus *Granulicatella*) showed the opposite trend.

The higher proportion of Proteobacteria in the deciduous dentition than at any other stage was mainly due to a higher proportion of Gammaproteobacteria at this stage of dentition. The order *Pseudomonadales *with *Pseudomonadaceae *(genus *Pseudomonas*) and *Moraxellaceae *families (genus *Acinetobacter, Moraxella *and *Enhydrobacter*) contributed the most to this difference. At the deciduous dentition stage, about 8% of all reads in the healthy dentition and 4% of the reads in the group with untreated caries belonged to genus *Acinetobacter *while sequences belonging to this genus were nearly absent from all other stages (Additional file 1, Figure 2). Other groups that also showed a higher abundance in the deciduous dentition included the *Enterobacteriaceae *family and the genus *Aggregatibacter *within the *Pasteurellaceae *family.

No significant differences were observed with respect to species richness between the different sub-groups. Rarefaction curves however (Additional file 2) did show a decreased estimate in species richness in children with caries compared to healthy or treated group.

### Individual phylogenic profiles of saliva

The oral microbiota were analyzed at an individual level using a taxonomic microarray based on discriminating regions of the small subunit (16S) ribosomal RNA gene. Of the 350 microarray probes, 156 gave a positive signal (signal/background ratio at least 3) in any of the samples analyzed, with, on average, 77 probes (range 48 - 93, s.d. 10.4) per individual sample (Additional file 3). All samples were positive for probes targeting the phyla Actinobacteria (genus *Actinomyces *and *Rothia*), Bacteroidetes (genus *Prevotella *and order *Bacteroidales*), Firmicutes (genus *Streptococcus*, *Veillonella*, *Lactobacillus*, *Granulicatella*) and Fusobacteria (genus *Fusobacterium*). All but one sample were positive for probes targeting the phylum Proteobacteria. Betaproteobacteria was the most prevalent class (genus *Neisseria - *in 97% of the samples), followed by Gammaproteobacteria (genus *Haemophilus*) and Epsilonproteobacteria (genus *Campylobacter*). Probes targeting candidate division TM7 were positive in 40% of the samples; the probes targeting the domain Archaea (Additional file 4: probe o1311, designed to target Crenarchaeota, Class Thermoprotei) were positive in 44% of the samples. The occurrence of Pseudomonas in the group of children with deciduous teeth was confined to one individual, and did not reflect a common feature of the oral microbiota of this group.

**Figure 3:**
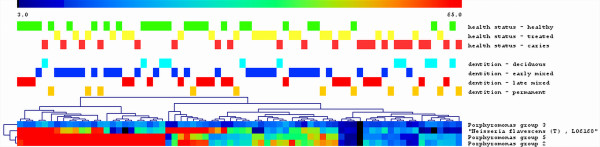
**Results of the Significance Analysis of Microarrays (SAM) on individual sample profiles obtained by phylogenetic microarray**. Individual saliva samples are shown in the columns, the probes - in the rows. Sample groups by health status and by dentition are shown in different colours. High probe signal abundance is depicted red, low - dark blue. Only probes which had significant association with the oral health status are shown. No association with the stage of dentition and individual probes was found.

To assess which probe-signals in the salivary profiles associate with any of the groups (the stage of the dentition and the oral health status), we performed the Significance Analysis of Microarrays (SAM analysis) [8]. No significant associations of microarray probe signals were found with the stage of dentition (deciduous, early or late mixed and permanent dentition). The health status (healthy, treated or carious) did show significant association with the signal of three probes targeting the Porphyromonas group (Additional file 4: probes o1503, o1504, o1506; the closest match with known species - *P. catoniae*, *P. gingivalis, P. gulae, P. macacae*), and one probe targeting *Neisseria flavescens *(o1486) (Figure 3). The healthy group had higher signal values of these probes than the group with active caries.

**Figure 4:**
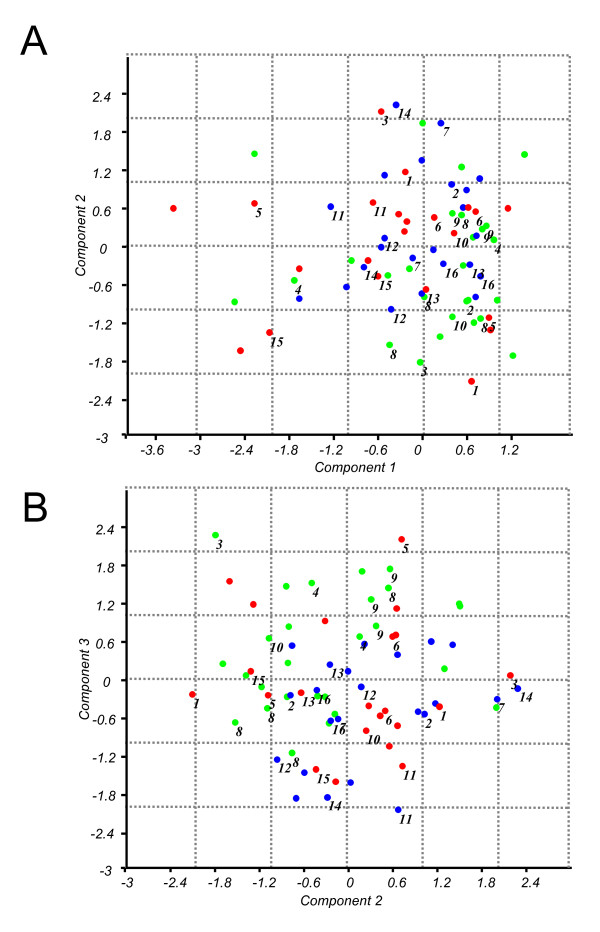
**Results of the Principal Component Analysis (PCA) on individual sample profiles obtained by phylogenetic microarray**. A) the plot of the PCA axis 1 (accounting for 21% of intersample variation) and the axis 2 (14% of intersample variation) B) the plot of the PCA axis 2 and the axis 3 (11.4% of intersample variation). Green dots - samples from the caries-free group, blue dots - samples from the treated group, red dots - samples from children with dental caries. Samples identified with the same number belonged to the siblings from one family. Samples without numbers belonged to unrelated individuals.

Dimensional reduction of the salivary profile data by principal component analysis (PCA) explained 45% of the total variance among the individual samples by the first three components (Figure 4A, B). Principal components PC2 and PC3 (14% and 11.4% of variation, respectively) partly discriminated the treated and carious samples from the samples that belonged to the healthy group. The three probes that contributed most to this difference were the same probes identified by the SAM analysis - probes (probe o1486, p = 0.04; probe, o1503 p = 0.04; probe o1506, p = 0.015; ANOVA, Games-Howell post-hoc test) targeting *N. flavescens *and Porphyromonas.

**Figure 5:**
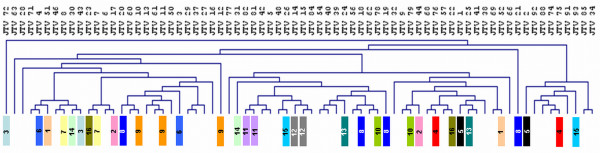
**Visualization of similarities between salivary profiles by hierarchical clustering analysis (average linkage method, Pearson correlation)**. Samples are depicted in the columns. Siblings, who participated in the study, are marked by the same colour, each colour representing different family. Unrelated individuals have no colour code.

In our study group, 35 children had one or more siblings (brothers or sisters) participating in this study, representing 16 families. To quantify the similarities between salivary microbiota profiles of siblings and unrelated individuals we calculated the sample distance matrix (Additional file 5) and visualized it using hierarchical clustering (Figure 5). If two sample profiles were identical, they would receive similarity value of 1. The average similarity between the respective family members was 0.87 (range 0.64 - 0.96; s.d. 0.09). The similarity between saliva profiles of unrelated children was slightly lower than the similarity between siblings (0.85; range 0.51 - 0.98; s.d. 0.08), but this difference was not statistically significant (Independent samples T-test, *p *= 0.275). For a few siblings, a higher similarity score was obtained, as for example members of family number 9 that showed nearly identical oral microbial communities (Figure 4).

### Analysis of specific microorganisms by quantitative PCR

The SAM and PCA analyses of the microarray profiles brought forward multiple probes targeting a group of several species within genus *Porphyromonas *- *P. catoniae, P. gingivalis, P. macacae and P. gulae *- as significantly associated with health. *P. gingivalis *is known as periodontal pathogen associated with destruction of periodontal tissue, and is phylogenetically closely related to *P. catoniae*. To evaluate the abundance of *P. gingivalis *in saliva, we used quantitative PCR. *P. gingivalis *could be detected in only 3 samples, at 1.3 × 10^2^, 4.5 × 10^4 ^and 2.7 × 10^5 ^cells/ml saliva. Although probe o1503 was designed to specifically target *P. gingivalis *in our microarray it showed positive signal/background ratio in 98% of samples, indicating general *Porphyromonas *sensitivity. The pyrosequencing data confirmed the absence (or presence below the detection limit of the method) of *P. gingivalis*, *P. gulae *and *P. macacae *in our samples: the only *Porphyromonas *species identified was *P. catoniae*, and it belonged to relatively abundant taxa (1.2 - 5.2% of all reads). The q-PCR data of *P. catoniae *showed that saliva samples contained between 10^5 ^and 10^8 ^of *P. catoniae *cells/ml saliva. The number of *P. catoniae *cells correlated significantly with the signal of both Porphyromonas probes - o1503, the probe with the closest match for *P. gingivalis*, and o1506, the probe targeting *P. catoniae *(Spearman's correlation, *p *< 0.001; r = 0.618, *p *< 0.001; r = 0.580 respectively). Both *Porphyromonas *probes of the microarray hybridized thus with *P. catoniae*. The number of *P. catoniae *cells correlated negatively with the total number of decayed, missing and filled (DMFS & dmfs) surfaces (*p *= 0.024, r = -0.266) and, although less strongly, also with the number of surfaces with caries lesions (*p *= 0.047, r = -0.235).

## Discussion

To our knowledge, this is the first report on the oral microbiome of children assessed at the depth of next generation sequencing. This open-ended and high resolution approach provided us with the overview of the taxa present in saliva of children, next to our existing knowledge on the adult microbiome [1,4]. Being aware of the current discussions on the artefacts that may be introduced by pyrosequencing [9,10], we applied a stringent protocol for filtering sequences. Through a combined approach of pyrosequencing, as well as taxonomic microarray analysis, we showed that salivary microbiome of children is already complex by the age of 3, and it matures with increasing age. However, at the age of puberty it still differs from the adult microbiome. Although the analysis of the adult oral microbiome was performed on a different sequencing platform (GS20), and the forward primers differed from the ones used in this study, this is unlikely to account for the major differences we observed.

As in previous observations, the oral microbiome was shown to be relatively stable, despite the significant biological changes that occur during the eruption of teeth. However, some differences were observed, with the most striking difference between the deciduous dentition and the rest of the stages. The children in this youngest age group harboured groups of microorganisms that are not usually associated with healthy commensal oral microbiota (*Pseudomonadaceae*, *Moraxellaceae*, *Enterobacteriaceae*) at relatively higher proportions than older children. Although from our sequencing data it is not possible to draw any conclusions regarding the pathogenicity, it is known from literature that the *Moraxellaceae *family member *Acinetobacter *- the genus that contributed most to this difference *- *is considered to be a major nosocomial respiratory pathogen and has been reported at high prevalence in dental plaque and saliva of hospitalized individuals [11,12]. *Acinetobacter baumanii *is infamous due to its multiple resistance to antibiotics [13,14]. Studies on upper respiratory tract infections in children have found an association between recurrent tonsillopharyngitis and another member within *Moraxellaceae *family - *Moraxella catarrhalis *[15]. The occurrence of *Pseudomonas *was shown to be confined to one individual.

In addition to the differences in their salivary microbiome, the children with deciduous teeth also had the highest amount of dental plaque among the four groups. Nevertheless, the prevalence and severity of inflammation of oral tissues (gingivitis and periodontitis) is low in healthy young children and gradually increases with increasing age [16,17]. A classic example of age-related susceptibility to gingivitis was presented three decades ago by an experimental gingivitis study [18]: after discontinued oral hygiene measures for 21 days, both, children and adults accumulated increasing amounts of dental plaque while signs of gingival inflammation were large in adults and remained negligible in children. This age-dependence has multiple natural reasons: the biological changes in tissues around the teeth during the eruption and exfoliation of teeth, the gradual development of the immune defence system and the endocrine system, all contributing to the maturation of the oral microbial communities [19]. With increasing age the proportions of periodontal pathogens also increase [17,20]. We confirmed the maturation of microbial composition by detecting increased proportions of Bacteroidetes (mainly genus *Prevotella*), Spirochaetes and candidate division TM7 with increasing age and found the highest abundance of these obligate anaerobes in the adult population [1].

**Figure 6:**
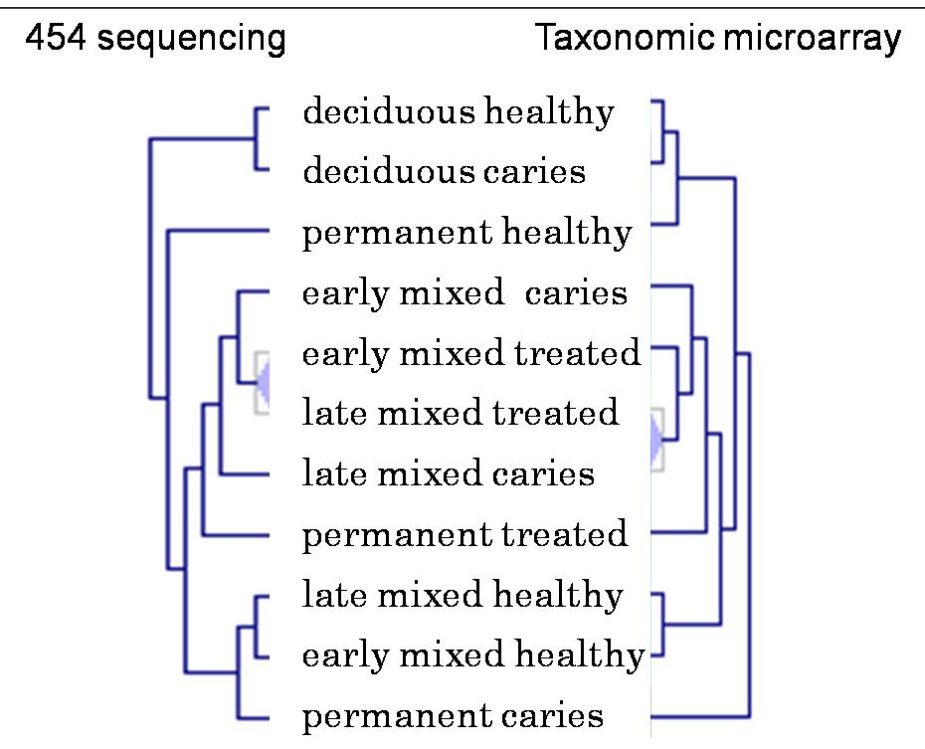
**Comparison of cluster profiling results of oral microbiota data obtained through 454 pyrosequencing (genus level), left part, and taxonomic microarray analysis (right)**. Hierarchical clustering for both datasets was performed in MeV v4.5.

Due to differences in the technological platforms it is difficult to make a direct comparison between microarray and sequencing data. The taxonomic resolution and 'dose response' that is offered by both technologies is quite different and does not allow a comparison of *e.g*. taxa abundance. A comparison was made using hierarchical clustering results based either on microarray or on 454 pyrosequencing data (Figure 6). For a number of samples a similar clustering was obtained. Three main overlapping clusters were identified, one composed of samples derived from the deciduous healthy and deciduous caries groups, a second composed of early mixed caries, early mixed treated, late mixed treated and late mixed caries. The third group overlapping was composed of late mixed healthy and early mixed healthy dentition. Discrepancies were observed in the classification of the permanent healthy, treated caries groups. Since the microarray was designed mainly aiming at children oral microbiota, it may be that the coverage of the current taxonomic microarray is not fully adequate for classification of the oral microbiota associated with permanent teeth. When zooming in further, differences in the clustering patterns were observed, that are likely to be due to differences in the platform technologies.

Our microarray data suggested a positive association between the signal of the probe targeting *Porphyromonas catoniae *with caries-free status of children. Quantitative PCR confirmed that all children in our study harboured *P. catoniae *in their saliva, and that the amount of this species correlated negatively with the number of decayed, missing and filled surfaces in their oral cavity. So far there are no other reports supporting the health association of *P. catoniae *in relation to dental caries. It has however been shown that in periodontitis patients *P. catoniae *is specifically associated with shallow pockets and healthy sites and not the diseased, deep pockets [21]. Another *Porphyromonas *species, *P. gingivalis*, one of the red complex organisms associated with periodontal disease [22], was found in only three samples by q-PCR. Other authors have also found *P. gingivalis *to be in low prevalence and abundance in children [20,23].

*P. catoniae *(previously *Oribaculum catoniae *[24] and Bacteroides D26 [25]) are saccharolytic, Gram-negative anaerobic rods and the only non-pigmented species among *Porphyromonas*. Unlike other *Porphyromonas *species, *P. catoniae *belongs to early colonizers among obligate anaerobes: it has been detected in two month-old pre-dentate infants, and becomes a regular colonizer once the first teeth erupt [7,26,27]. *P. catoniae *differs from other *Porphyromonas *spp. in its frequent ability to produce ß-lactamase [28], an enzyme that inactivates ß-lactam antibiotics. Könönen et al [7] have hypothesized that this, on one hand, may offer a reservoir of resistance genes potentially transferable to pathogens, while on the other hand, this may allow commensal microorganism as *P. catoniae *to remain in the oral cavity after frequent ß-lactam antibiotic courses and prevent the overgrowth of opportunistic pathogens.

An interesting group to include in the study was the group of siblings. Fourteen sibling pairs, one set of three siblings (family number 9) as well as one set of four siblings (family number 8) participated in our study. From genotyping studies it is known that both vertical (from mother to child) and horizontal (among siblings and between spouses) transmission of oral microorganisms can occur, *e.g*., identical *P. gingivalis *genotypes among siblings were found in 26% of the sibships [29], while in two of the three families from which strains from siblings were available, a common *Streptococcus mutans *ribotype occurred in all family members [30]. Recently even the transmission of *S. mutans *between unrelated children at public schools was reported [31]. Besides microbial transmission, host genetic factors may influence the proportions of certain species in genetically related individuals, as has been estimated from the genetic contributions to the increased similarity of the microbiota of twins as compared with microbiota of unrelated people [32,33]. In general, our study did not find higher similarity in saliva microbial profiles among siblings than among unrelated individuals. One exception was the family number 9, where profiles of all three siblings were highly similar (Pearson correlation 0.95-0.96) (Figure 4, Additional file 5). One should interpret our findings with caution though, since the individual saliva profiles in our study were biased by using targeted approach and our study was not designed to trace different genotypes. Only studies using an open-ended approach such as individually barcoded sample pyrosequencing [4] will allow full comparisons of individual microbiomes.

## Conclusions

In conclusion, we have demonstrated that salivary microbiota of children aged 3 to 18 years is still in the process of maturation and that certain oral microorganisms may be associated with caries-free oral cavities. This study indicates the potential for further studies, preferably longitudinal clinical trials involving large study populations in revealing the panel of microorganisms as potential oral health markers. With that knowledge oral health professionals will be able to perform timely interventions in the oral ecosystem to prevent the shift from health to disease.

## Methods

### Study Population and Clinical Screening

Children aged 3-18 years, living in the area of Oss, the Netherlands, and being registered at the Youth Dental Care clinic (Jeugdtandverzorging Noordoost Noord-Brabant) participated in the study. Ethical approval was given by the independent Medical Ethics Committee of TNO. Inclusion criteria were good general health, no use of antibiotics in the last 6 months, no braces and written consent of the parents/caretakers of the child. The children were clinically examined in the dental clinic by one calibrated dental epidemiologist as a part of their regular dental check-up. The oral examination included a visual inspection of the oral mucosa, caries experience and plaque. The caries experience was expressed by the dmfs-index. This index was calculated by adding up the total number of decayed, missing and filled surfaces. Surfaces with early enamel lesions (white spots) were registered as sound. A tooth surface was registered as carious if caries lesion had reached dentin or if tooth enamel was undermined by underlying lesion resulting in at least a 0.25-mm-deep surface defect. The amount of dental plaque (plaque index) was assessed using criteria of Greene and Vermillion [34]. In brief, six dental surfaces - buccal surface of both upper permanent or deciduous molars, labial surface of 11 and 31 (permanent dentition) or 51 and 71 (deciduous dentition), and lingual surface of 36 and 46 (permanent dentition) or 75 and 85 (deciduous dentition) - were inspected. If the selected tooth was missing, the contralateral tooth or the neighbouring tooth was assessed instead. Amount of plaque was determined visually by moving the probe along the tooth surface. The surface received score 0 if no plaque was visible, score 1 if plaque was present only at the cervical third of the surface, score 2 if plaque covered a cervical half of the surface, score 3 if plaque reached the incisal or occlusal surface of the tooth. A mean plaque index (average score per number of surfaces scored) was calculated per child.

### Sample Collection

Unstimulated saliva, as the representation of an average sample of the whole oral ecosystem [3,4], was collected at the day of the dental check-up, at home, before breakfast and toothbrushing, by drooling into a DNA-free, sterile vial for 5 minutes. The parents were instructed to store the saliva refrigerated (4-7°C) and take the vial to the dental clinic, *i.e*., within 6 h after collection.

### DNA Extraction

A 0.1-ml quantity of saliva sample was transferred to a sterile screw-cap Eppendorf tube with 0.25 ml of lysis buffer (AGOWA mag Mini DNA Isolation Kit, AGOWA, Berlin, Germany). Then 0.3 g zirconium beads (diameter, 0.1 mm; Biospec Products, Bartlesville, OK, USA) and 0.2 ml phenol were added to each sample. The samples were homogenized with a Mini-beadbeater (Biospec Products) for 2 min. DNA was extracted with the AGOWA mag Mini DNA Isolation Kit and quantified (Nanodrop ND-1000; NanoDrop Technologies, Montchanin, DE, USA).

### PCR Amplification, Sample Pooling and Pyrosequencing

PCR amplicon libraries of the small subunit ribosomal RNA gene V5-V6 hypervariable region were generated for the individual samples. PCR was performed using the forward primer 785F (GGA TTA GAT ACC CBR GTA GTC) and the reverse primer 1061R (TCA CGR CAC GAG CTG ACG AC). The primers included the 454 Life Sciences Adapter A (forward primer) and B (reverse primer) fused to the 5' end of the 16 S rRNA bacterial primer sequence and a unique trinucleotide sample identification key per each sample group. This resulted in 12 distinctly labelled pools of samples - deciduous, early mixed, late mixed, and permanent dentition at healthy, treated or caries state. The amplification mix contained 2 units of Pfu Ultra II Fusion HS DNA polymerase and 1× *PfuUltra *II reaction buffer (Stratagene), 200 μM dNTP PurePeak DNA polymerase Mix (Pierce Nucleic Acid Technologies, Milwaukee, WI), and 0.2 μM of each primer. After denaturation (94°C; 2 min), 30 cycles were performed that consisted of denaturation (94°C; 30 sec), annealing (50°C; 40 sec), and extension (72°C; 80 sec). DNA was isolated by means of the MinElute kit (Qiagen, Hilden, Germany). The quality and the size of the amplicons were analyzed on the Agilent 2100 Bioanalyser with the DNA 1000 Chip kit (Agilent Technologies, Santa Clara, CA, USA) and quantified using Nanodrop ND-1000 spectrophotometer. The amplicon libraries were pooled in equimolar amounts and sequenced unidirectionally in the reverse direction (B-adaptor) by means of the Genome Sequencer FLX (GS-FLX) system (Roche, Basel, Switzerland).

### Processing of the Pyrosequencing Data

GS-FLX sequencing data were processed as previously described [35]. In brief, we trimmed sequences by removing primer sequences and low-quality data (sequences that did not have an exact match to the reverse primer, that had an ambiguous base call (N) in the sequence, or that were shorter than 50 nt after trimming). We then used the GAST algorithm [36] to calculate the percent difference between each unique sequence and its closest match in a database of 69816 unique eubacterial and 2779 unique archaeal V5-V6 sequences, representing 323499 SSU rRNA sequences from the SILVA database [37]. Taxa were assigned to each full-length reference sequence using several sources including Entrez Genome entries, cultured strain identities, SILVA, and the Ribosomal Database Project Classifier [38]. In cases where reads were equidistant to multiple V5-V6 reference sequences, and/or where identical V5-V6 sequences were derived from longer sequences mapping to different taxa, reads were assigned to the lowest common taxon of at least two-thirds of the sequences. Only sequences that were found at least 5 times were included in the analyses. This strict and conservative approach was chosen to preclude inclusion of sequences from potential contamination or sequencing artefacts.

### Probe Design for the 16S rDNA Microarray

For the design of the taxonomic microarray literature inventory was made on the description of the normal oral microbiota [2,3,39-41]. The literature based inventory was compared to the 454 pyrosequencing data that were obtained in this study. While the majority of taxa observed by 454 sequencing were already included in the literature inventory, a number of taxonomic groups were found to be missing. These were included in the list. Based on the list, probes were designed using ARB [42]. The probes were 20-22 nucleotides in size, with a predicted melting temperature of 60°C ± 5, and a GC level between 40 and 60%. The full list of oligonucleotides is provided in Additional file 4.

### Microarray Printing

Microarrays for this study were produced in house, using the ArrayIt Nanoprint60 Microarray Robot. The 5'-amino-modifier-C6-linked oligonucleotides were diluted to a final concentration of 25 μM in a 50 mM Phosphate buffer (pH 7), and printed onto CodeLink Activated Slides. Following incubation at 80% humidity under ambient conditions, slides were blocked in a buffer of 0.1 M Tris Base and 50 mM ethanolamine (pH 9) for 45 minutes at 50°C while shaking in accordance with the manufacturer's recommendations. After that, the slides were rinsed twice in MilliQ purified water (MilliQ, Millipore), washed in 4 × SSC (600 mM sodium chloride/60 mM sodium citrate), 0.1% sodium dodecyl sulfate (SDS) for 30 minutes at 50°C, and washed in pre-warmed (50°C) MilliQ water. Following that, the slides were washed twice in MilliQ water at a room temperature, and dried under a stream of nitrogen. Slides were stored under nitrogen until use.

### DNA Labelling and Hybridization

For taxonomic microarray analysis, 16 S rDNA was amplified by PCR as described above, using forward primers 8F (AGA GTT TGA TCH TGG YTC AG) and 8F-bif (TGG CTC AGG ATG AAC GCT G) and reverse primer 1061R (TCA CGR CAC GAG CTG ACG AC). Following PCR amplification, DNA was amplified by random Klenow amplification with the BioPrime DNA Amplification kit (Invitrogen) according to the manufacturer's recommendations. Klenow-amplified DNA was passed through an Illustra AutoSeq G-50 column (GE Healthcare G50) for purification and concentrated in a speedvac. Following, the DNA was labelled by Terminal Transferase coupling of Cy3-dUTP (Promega). Following Illustra AutoSeq G-50 column purification, and vacuum concentration, the labelled DNA was dissolved in 40 μl Easyhyb hybridization buffer (Roche), and denatured for 2 min at 95°C. Printed slides were pre-hybridized in 0.45 μm-filtered pre-hybridization buffer [1% BSA, 5 × SSC, and 0.1% SDS] at 42°C for 45 min with rotation, then washed twice with MilliQ purified water, dried with nitrogen, and pre-warmed at 42°C. The microarrays were placed in the ProPlate multi-array system (Grace Bio-Labs). The hybridization mixture was then pipetted in the individual wells, and incubated in a hybridization chamber for four hours. Following hybridization, slides were then thoroughly washed sequentially in 1 × SSC, 0.2% SDS for 10 sec at 37°C, 0.5 × SSC for 10 sec at 37°C, and twice in 0.2 × SSC for 10 min at room temperature. Slides were dried with nitrogen and scanned using a Scanarray Express 680013 Microarray Analysis System (Perkin Elmers Life Analytical Sciences Inc.). Images were obtained and quantified with ImaGene 4.2 software (Biodiscovery).

### Microarray validation

To validate microarray performance, a number of tests were performed. These included: 1) Replicated analysis of samples to test robustness of microarray data; 2) Spiking DNA of underrepresented species to a complex mixture followed by microarray analysis; 3) performing a direct comparison between qPCR and microarray data, and 4) performing a direct comparison between sequencing and microarray data.

### Replicated Analysis

Technical replicates were included to verify robustness of data. In the examples described below, three replicates were compared. Two were analyzed on slides of the same printing series - sMF02-11, and one was analyzed on an older slide series (sMF02-9). The Pearson correlation (r) between the three microarray analyses was high, and varied between 0.91 and 0.93, and was independent of the slide series. For comparison, correlation between different saliva samples ranged from 0.4 to 0.7.

### DNA Spiking

To test the performance of the microarray with the complex oral microbial community, we performed a spiking experiment. For this, DNA was isolated from saliva samples of a healthy adult donor as well as from pure cultures of *Lactobacillus casei *(ATCC 334). Following, DNA was quantified, and pure culture DNA was added to saliva DNA at levels ranging between 0.01 and 1% of the total community. Then the DNA was labelled, and used to hybridize the microarray. A clear signal was detected in all spiked samples for the *L. casei *spot on the microarray in a dose responsive manner. At the family level, also a significant dose responsive increase in microarray spot intensity was detected (with lower sensitivity).

### Direct Comparison between qPCR and Microarray Data

A third method for validating microarray data was by cross comparing species levels established by quantitative PCR with the results obtained using the microarray. In the current study, this was performed for *Porphyromonas catoniae *in saliva samples obtained from the children. During the study, 74 saliva samples were analyzed. The q-PCR data of *P. catoniae *showed that saliva samples contained between 10^5 ^and 10^8 ^of *P. catoniae *cells/ml saliva. The fluorescence intensity for the *P. catoniae *probes on the microarray ranged between 0 and 110. The number of *P. catoniae *cells correlated significantly with the signal of both Porphyromonas probes - o1503 and o1506. The Spearman's correlations were p < 0.001; r = 0.618 and p < 0.001; r = 0.580, respectively.

### Direct Comparison between Sequencing and Microarray Data

To enable a direct comparison between microarray and sequencing data, both data sets were coupled by using the probe and sequence data. For this, a string search was used to identify matching probe sequences within the 454 pyrosequencing data. For 26 probes a direct match was identified. This relatively low number of matching probe sequences is due to the fact that the pyrosequencing data span the V5-V6 region, while the majority of probes were defined in the V2, V3 and V4 region of the 16 S ribosomal gene. We then calculated the Pearson correlation between the microarray and 454 sequencing data. Good (r = 0.85) to excellent (p > 0.9) correlation was found for probes corresponding to relatively abundant taxa (>0.1% of all reads; signal/background fluorescence value >20). The correlation for less abundant species was weak. No false positive probes were found. Only two probes (o1402 and o1445) were classified as false negatives.

### Targeting of specific microorganisms by quantitative PCR

Quantitative PCR was performed on the Applied Biosystems 7500 Fast Real-Time PCR System. The total microbial load was determined using the universal primer set described by Nadkarni et al [43]. The *P. catoniae *and *P. gingivalis *primer-probe sets were designed using Primer Express (Applied Biosystems). To quantify *P. catoniae*, the primer-probe set of *P. catoniae*-16S-F (CGG TTG CCA TCAG GTA ATG C), *P. catoniae*-16S-R (CAC CTT CCT CAC GCC TTA CG) and *P. catoniae*-16S-probe (TCC GTA GAG ACT GCC G), a minor-groove binding probe (MGB) labeled with 6-carboxy-fluorescein (FAM) was used. The *P. gingivalis *primer-probe set was composed of *P. gingivalis*-16S-F (GCG CTCA ACG TTC AGC C), *P. gingivalis*-16S-R (CAC GAA TTC CGC CTG C) and the FAM labeled minor-groove binding (MGB) probe *P. gingivalis*-16S-probe (CAC TGA ACT CAA GCC CGG CAG TTT CAA). Quantitative PCR was performed using the Diagenode Universal Mastermix, in accordance with the manufacturer's recommendations. Standard controls included a serial dilution series of purified genomic DNA of *P. gingivalis *ATCC BAA-308 and *P. catoniae *ATCC 51270.

### Statistical analyses

To identify the probes that contribute significantly to the different response variables (oral health status, the stage of dentition), we performed the Significance Analysis of Microarrays (SAM analysis) - a non-parametric statistical technique for finding significant differences between microarray data that are grouped based on experimental conditions [8]. To reduce the dimensions of the array data we performed the principal component analysis (PCA). Similarities among individual sample profiles were calculated using the sample distance matrix. The SAM and PCA analyses were performed using the MeV software package, as part of the TM4 microarray software suite [44]. The sample distance matrix was calculated using Pearson correlation coefficient and visualized using hierarchical clustering with the average linkage method. Independent samples T-test, Pearson and Spearman correlations were calculated using SPSS (Version 17.0). Abundances of probes that showed significant differences in SAM and contributed most to the PCA were tested by ANOVA, Games-Howell post-hoc test using SPSS (Version 17.0).

## Authors' contributions

BJFK, AAS, EZ, RCM and WC contributed to the design of the study; AAS carried out clinical epidemiological procedures; BJFK processed the samples; SMH performed sequence analyses; EZ, WC and BJFK wrote the manuscript. All authors have read and approved the final manuscript.

## Pre-publication history

The pre-publication history for this paper can be accessed here:

http://www.biomedcentral.com/1755-8794/4/22/prepub

## Supplementary Material

Additional file 1 **Full list and relative abundance of higher taxa per group by health status and dentition stage, as obtained by 454 pyrosequencing**. This file lists all 113 higher taxa (genera or more inclusive taxa when sequences could not be confidently classified to the genus level) and their relative abundance in saliva samples of children. Click here for file

Additional file 2 **Rarefaction plot of the unique sequences in saliva samples of three oral health groups of children with early mixed dentition (healthy, treated or with caries)**. This is a rarefaction plot of all unique sequences by the number of sequences sampled in children with early mixed dentition by their oral health status. Click here for file

Additional file 3 **Microarray data**. This is an Excel file with normalized microarray signal intensities for all samples used in this study. Click here for file

Additional file 4 **Full list of the microarray 16 S rDNA probes and their targets used in the microarray**. This file lists all 16 S rDNA probes used in the microarray, probe sequences and their targets as identified by RDP blast search. Click here for file

Additional file 5 **Distance matrix of saliva sample profiles from microarray data**. This is a file with sample distance matrix calculated using Pearson correlation. The samples that belonged to siblings of one family are depicted with the same number. Samples that are numbered with 0, originated from children without siblings in the study group. Click here for file

## References

[B1] Keijser BJF Zaura E Huse SM van der Vossen JMBM Schuren FHJ Montijn RC ten Cate JM Crielaard W Pyrosequencing analysis of the oral microflora of healthy adults J Dent Res 2008 87 1016 1020 10.1177/154405910808701104 18946007

[B2] Paster BJ Olsen I Aas JA Dewhirst FE The breadth of bacterial diversity in the human periodontal pocket and other oral sites Periodontol 2000 2006 42 80 87 10.1111/j.1600-0757.2006.00174.x 16930307

[B3] Aas JA Paster BJ Stokes LN Olsen I Dewhirst FE Defining the normal bacterial flora of the oral cavity J Clin Microbiol 2005 43 5721 5732 10.1128/JCM.43.11.5721-5732.2005 16272510 PMC1287824

[B4] Zaura E Keijser BJF Huse SM Crielaard W Defining the healthy "core microbiome" of oral microbial communities BMC Microbiol 2009 9 259 10.1186/1471-2180-9-259 20003481 PMC2805672

[B5] Costello EK Lauber CL Hamady M Fierer N Gordon JI Knight R Bacterial community variation in human body habitats across space and time Science 2009 326 1694 1697 10.1126/science.1177486 19892944 PMC3602444

[B6] Könönen E Oral colonization by anaerobic bacteria during childhood: role in health and disease Oral Diseases 1999 5 4 278 285 10561714 10.1111/j.1601-0825.1999.tb00090.x

[B7] Kononen E Development of oral bacterial flora in young children Ann Med 2000 32 107 112 10.3109/07853890009011759 10766401

[B8] Tusher VG Tibshirani R Chu G Significance analysis of microarrays applied to the ionizing radiation response Proc Natl Acad Sci USA 2001 98 5116 5121 10.1073/pnas.091062498 11309499 PMC33173

[B9] Quince C Lanzen A Curtis TP Davenport RJ Hall N Head IM Read LF Sloan WT Accurate determination of microbial diversity from 454 pyrosequencing data Nat Meth 2009 6 639 641 10.1038/nmeth.1361 19668203

[B10] Kunin V Engelbrektson A Ochman H Hugenholtz P Wrinkles in the rare biosphere: pyrosequencing errors can lead to artificial inflation of diversity estimates Environ Microbiol 2009 19725865 10.1111/j.1462-2920.2009.02051.x

[B11] Zuanazzi D Souto R Mattos MBA Zuanazzi MR Tura BR Sansone C Colombo APV Prevalence of potential bacterial respiratory pathogens in the oral cavity of hospitalised individuals Arch Oral Biol 2009 in press Corrected Proof 19939349 10.1016/j.archoralbio.2009.10.005

[B12] Fourrier FMD Duvivier BDMD Boutigny HDMD Roussel-Delvallez MMD Chopin CMD Colonization of dental plaque: A source of nosocomial infections in intensive care unit patients Crit Care Med 1998 26 301 308 10.1097/00003246-199802000-00032 9468169

[B13] Livermore DM Has the era of untreatable infections arrived? J Antimicrob Chemother 2009 64 suppl_1 i29 36 10.1093/jac/dkp255 19675016

[B14] Peleg AY Seifert H Paterson DL *Acinetobacter baumannii: *Emergence of a successful pathogen Clin Microbiol Rev 2008 21 538 582 10.1128/CMR.00058-07 18625687 PMC2493088

[B15] Van Staaij BK Akker EHVD Van Dorsser EHMDH Fleer A Hoes AW Schilder AGM Does the tonsillar surface flora differ in children with and without tonsillar disease? Acta Otolaryngol 2003 123 873 878 10.1080/00016480310000395 14575405

[B16] Matsson L Factors influencing the susceptibility to gingivitis during childhood--a review Int J Paediatr Dent 1993 3 119 127 10.1111/j.1365-263X.1993.tb00067.x 8260459

[B17] Papaioannou W Gizani S Haffajee AD Quirynen M Mamai-Homata E Papagiannoulis L The microbiota on different oral surfaces in healthy children Oral Microbiol Immunol 2009 24 183 189 10.1111/j.1399-302X.2008.00493.x 19416446

[B18] Matsson L Development of gingivitis in pre-school children and young adults. A comparative experimental study J Clin Periodontol 1978 5 24 34 10.1111/j.1600-051X.1978.tb01903.x 353084

[B19] Bimstein E Matsson L Growth and development considerations in the diagnosis of gingivitis and periodontitis in children Pediatr Dent 1999 21 186 191 10355010

[B20] Kimura S Ooshima T Takiguchi M Sasaki Y Amano A Morisaki I Hamada S Periodontopathic bacterial infection in childhood J Periodontol 2002 73 20 26 10.1902/jop.2002.73.1.20 11846195

[B21] de Lillo A Booth V Kyriacou L Weightman AJ Wade WG Culture-independent identification of periodontitis-associated Porphyromonas and Tannerella populations by targeted molecular analysis J Clin Microbiol 2004 42 5523 5527 10.1128/JCM.42.12.5523-5527.2004 15583276 PMC535285

[B22] Socransky SS Haffajee AD Cugini MA Smith C Kent RL Microbial complexes in subgingival plaque J Clin Periodontol 1998 25 134 144 10.1111/j.1600-051X.1998.tb02419.x 9495612

[B23] Könönen E Asikainen S Saarela M Karjalainen J Jousimies-Somer H The oral gram-negative anaerobic microflora in young children: longitudinal changes from edentulous to dentate mouth Oral Microbiol Immunol 1994 9 136 141 7936718 10.1111/j.1399-302x.1994.tb00049.x

[B24] Willems A Collins M Reclassification of *Oribaculum catoniae *(Moore and Moore 1994) as *Porphyromonas catoniae *comb. nov. and emendation of the genus Porphyromonas Int J Syst Bacteriol 1995 45 578 581 10.1099/00207713-45-3-578 8590687

[B25] Moore LVH Moore WEC *Oribaculum catoniae *gen. nov., sp. nov.; *Catonella morbi *gen. nov., sp. nov.; *Hallella seregens *gen. nov., sp. nov.; *Johnsonella ignava *gen. nov., sp. nov.; and *Dialister pneumosintes *gen. nov., comb. nov., nom. rev., Anaerobic Gram-Negative Bacilli from the Human Gingival Crevice Int J Syst Bacteriol 1994 44 187 192 10.1099/00207713-44-2-187 8186083

[B26] Kononen E Kanervo A Takala A Asikainen S Jousimies-Somer H Establishment of oral anaerobes during the first year of life J Dent Res 1999 78 1634 1639 10.1177/00220345990780100801 10520968

[B27] Könönen E Väisänen ML Finegold SM Heine R Jousimies-Somer H Cellular fatty acid analysis and enzyme profiles of *Porphyromonas catoniae *-- a frequent colonizer of the oral cavity in children Anaerobe 1996 2 329 335

[B28] Nyfors S Kononen E Takala A Jousimies-Somer H beta -Lactamase Production by Oral Anaerobic Gram-Negative Species in Infants in Relation to Previous Antimicrobial Therapy Antimicrob Agents Chemother 1999 43 1591 1594 10390208 10.1128/aac.43.7.1591 PMC89329

[B29] van Winkelhoff AJ Rijnsburger MC van der Velden U Clonal stability of *Porphyromonas gingivalis *in untreated periodontitis J Clin Periodontol 2008 35 674 679 10.1111/j.1600-051X.2008.01285.x 18616757

[B30] Köhler B Lundberg A-B Birkhed D Papapanou PN Longitudinal study of intrafamilial mutans streptococci ribotypes Eur J Oral Sci 2003 111 383 389 12974680 10.1034/j.1600-0722.2003.00068.x

[B31] Domejean S Zhan L DenBesten PK Stamper J Boyce WT Featherstone JD Horizontal transmission of mutans streptococci in children J Dent Res 2010 89 51 55 10.1177/0022034509353400 19918090 PMC3318045

[B32] Moore WE Burmeister JA Brooks CN Ranney RR Hinkelmann KH Schieken RM Moore LV Investigation of the influences of puberty, genetics, and environment on the composition of subgingival periodontal floras Infect Immun 1993 61 2891 2898 8514392 10.1128/iai.61.7.2891-2898.1993 PMC280936

[B33] Corby PM Bretz WA Hart TC Schork NJ Wessel J Lyons-Weiler J Paster BJ Heritability of oral microbial species in caries-active and caries-free twins Twin Res Hum Genet 2007 10 821 828 10.1375/twin.10.6.821 18179393 PMC3148892

[B34] Greene JC Vermillion JR The simplified oral hygiene index J Am Dent Assoc 1964 68 7 13 14076341 10.14219/jada.archive.1964.0034

[B35] Sogin ML Morrison HG Huber JA Mark Welch D Huse SM Neal PR Arrieta JM Herndl GJ Microbial diversity in the deep sea and the underexplored "rare biosphere" Proc Natl Acad Sci USA 2006 103 12115 12120 10.1073/pnas.0605127103 16880384 PMC1524930

[B36] Huse SM Dethlefsen L Huber JA Mark Welch D Relman DA Sogin ML Exploring microbial diversity and taxonomy using SSU rRNA hypervariable tag sequencing PLoS Genet 2008 4 e1000255 10.1371/journal.pgen.1000255 19023400 PMC2577301

[B37] Pruesse E Quast C Knittel K Fuchs BM Ludwig W Peplies J Glockner FO SILVA: a comprehensive online resource for quality checked and aligned ribosomal RNA sequence data compatible with ARB Nucl Acids Res 2007 35 7188 7196 10.1093/nar/gkm864 17947321 PMC2175337

[B38] Cole JR Chai B Farris RJ Wang Q Kulam SA McGarrell DM Garrity GM Tiedje JM The Ribosomal Database Project (RDP-II): sequences and tools for high-throughput rRNA analysis Nucl Acids Res 2005 33 D294 296 10.1093/nar/gki038 15608200 PMC539992

[B39] Paster BJ Boches SK Galvin JL Ericson RE Lau CN Levanos VA Sahasrabudhe A Dewhirst FE Bacterial diversity in human subgingival plaque J Bacteriol 2001 183 12 3770 3783 10.1128/JB.183.12.3770-3783.2001 11371542 PMC95255

[B40] Aas JA Griffen AL Dardis SR Lee AM Olsen I Dewhirst FE Leys EJ Paster BJ Bacteria of Dental Caries in Primary and Permanent Teeth in Children and Young Adults J Clin Microbiol 2008 46 1407 1417 10.1128/JCM.01410-07 18216213 PMC2292933

[B41] Sakamoto M Rocas IN Siqueira JF Benno Y Molecular analysis of bacteria in asymptomatic and symptomatic endodontic infections Oral Microbiology and Immunology 2006 21 112 122 10.1111/j.1399-302X.2006.00270.x 16476021

[B42] Ludwig W Strunk O Westram R Richter L Meier H Buchner A Lai T Steppi S Jobb G ARB: a software environment for sequence data Nucl Acids Res 2004 32 1363 1371 10.1093/nar/gkh293 14985472 PMC390282

[B43] Nadkarni MA Martin FE Jacques NA Hunter N Determination of bacterial load by real-time PCR using a broad-range (universal) probe and primers set Microbiology 2002 148 257 266 11782518 10.1099/00221287-148-1-257

[B44] Saeed AI Bhagabati NK Braisted JC Liang W Sharov V Howe EA Li J Thiagarajan M White JA Quackenbush J TM4 Microarray Software Suite Methods in Enzymology 2006 411 Academic Press 134 193 10.1016/S0076-6879(06)11009-5 16939790

