# Supplementary material for: Exploring the oral microbiota of children at various developmental stages of their dentition in the relation to their oral health
Source: BMC Med Genomics. 2011 Mar 4;4:22. doi: 10.1186/1755-8794-4-22 (PMC3058002; doi:10.1186/1755-8794-4-22)
Supplement: Additional file 1 — Full list and relative abundance of higher taxa per group by health status and dentition stage, as obtained by 454 pyrosequencing. This file lists all 113 higher taxa (genera or more inclusive taxa when sequences could not be confidently classified to the genus level) and their relative abundance in saliva samples of children. [file 1755-8794-4-22-S1.PDF]

					Healthy				Treated				Carious			
Phylum	Class	Order	Family	Genus	Deciduous	Early mixed	Late mixed	Permanent	Deciduous	Early mixed	Late mixed	Permanent	Deciduous	Early mixed	Late mixed	Permanent
Actinobacteria	Actinobacteria	Actinomycetales	Actinomycetaceae	Actinobaculum	0.00	0.00	0.06	0.01	0.01	0.00	0.01	0.00	0.07	0.07	0.02	0.02
Actinobacteria	Actinobacteria	Actinomycetales	Actinomycetaceae	Actinomyces	2.68	2.99	2.48	2.45	3.95	3.73	4.56	5.03	3.24	4.69	4.80	3.70
Actinobacteria	Actinobacteria	Actinomycetales	Corynebacteriaceae	Corynebacterium	0.10	0.40	0.26	0.18	0.27	0.44	0.37	0.13	0.36	0.18	0.20	0.21
Actinobacteria	Actinobacteria	Actinomycetales	Micrococcaceae	Arthrobacter	0.01	0.04	0.01	0.00	0.02	0.06	0.01	0.03	0.03	0.05	0.00	0.01
Actinobacteria	Actinobacteria	Actinomycetales	Micrococcaceae	Kocuria	0.02	0.04	0.00	0.00	0.01	0.08	0.01	0.01	0.02	0.00	0.01	0.00
Actinobacteria	Actinobacteria	Actinomycetales	Micrococcaceae	Nesterenkonia	0.00	0.00	0.01	0.00	0.02	0.00	0.01	0.00	0.00	0.00	0.02	0.00
Actinobacteria	Actinobacteria	Actinomycetales	Micrococcaceae	Renibacterium	0.01	0.00	0.00	0.01	0.01	0.02	0.00	0.00	0.00	0.00	0.00	0.00
Actinobacteria	Actinobacteria	Actinomycetales	Micrococcaceae	Rothia	9.08	3.94	3.78	11.23	5.84	6.87	5.30	3.16	3.94	3.37	4.81	1.44
Actinobacteria	Actinobacteria	Actinomycetales	Micrococcaceae	NA	0.03	0.00	0.01	0.00	0.00	0.00	0.00	0.03	0.00	0.00	0.00	0.00
Actinobacteria	Actinobacteria	Actinomycetales	Propionibacteriaceae	Propionibacterium	0.00	0.01	0.04	0.01	0.02	0.03	0.00	0.00	0.04	0.00	0.02	0.00
Actinobacteria	Actinobacteria	Actinomycetales	Pseudonocardiaceae	Saccharomonospora	0.00	0.00	0.02	0.00	0.00	0.00	0.02	0.00	0.00	0.00	0.00	0.01
Actinobacteria	Actinobacteria	Actinomycetales	Pseudonocardiaceae	NA	0.00	0.01	0.00	0.00	0.00	0.01	0.00	0.01	0.01	0.00	0.01	0.00
Actinobacteria	Actinobacteria	Bifidobacteriales	Bifidobacteriaceae	Alloscardovia	0.00	0.00	0.00	0.00	0.00	0.00	0.01	0.00	0.00	0.07	0.00	0.00
Actinobacteria	Actinobacteria	Bifidobacteriales	Bifidobacteriaceae	Bifidobacterium	0.00	0.00	0.00	0.00	0.00	0.00	0.05	0.01	0.02	0.10	0.00	0.00
Actinobacteria	Actinobacteria	Bifidobacteriales	Bifidobacteriaceae	Metascardovia	0.00	0.00	0.00	0.00	0.00	0.00	0.19	0.00	0.00	0.00	0.00	0.00
Actinobacteria	Actinobacteria	Bifidobacteriales	Bifidobacteriaceae	NA	0.00	0.01	0.00	0.01	0.00	0.01	0.02	0.01	0.01	0.02	0.02	0.03
Actinobacteria	Actinobacteria	Coriobacteriales	Coriobacteriaceae	Atopobium	0.30	0.08	0.08	0.35	0.12	0.19	0.35	0.44	0.39	0.20	0.28	0.22
Actinobacteria	Actinobacteria	NA	NA	NA	0.09	0.03	0.04	0.06	0.01	0.05	0.11	0.11	0.04	0.02	0.09	0.09
Bacteroidetes	Bacteroidia	Bacteroidales	Porphyromonadaceae	Porphyromonas	3.06	5.15	3.84	2.14	4.40	2.77	2.79	1.17	3.77	1.90	1.66	4.20
Bacteroidetes	Bacteroidia	Bacteroidales	Prevotellaceae	Prevotella	6.14	9.08	11.22	12.49	6.54	10.26	12.30	9.61	7.61	11.84	14.35	15.90
Bacteroidetes	Bacteroidia	Bacteroidales	Prevotellaceae	NA	0.00	0.00	0.00	0.01	0.01	0.00	0.00	0.00	0.01	0.00	0.02	0.00
Bacteroidetes	Bacteroidia	Bacteroidales	NA	NA	0.99	1.35	1.31	0.89	0.88	1.24	1.77	0.69	1.41	0.87	1.81	1.51
Bacteroidetes	Flavobacteria	Flavobacteriales	Flavobacteriaceae	Capnocytophaga	0.15	0.35	0.32	0.25	0.48	0.19	0.14	0.06	0.44	0.13	0.24	0.69
Bacteroidetes	Flavobacteria	Flavobacteriales	Flavobacteriaceae	Chryseobacterium	0.00	0.00	0.02	0.00	0.02	0.01	0.01	0.03	0.00	0.00	0.01	0.00
Bacteroidetes	Flavobacteria	Flavobacteriales	Flavobacteriaceae	Cloacibacterium	0.35	0.50	0.19	0.08	0.49	0.28	0.07	0.07	0.41	0.15	0.10	0.21
Bacteroidetes	Flavobacteria	Flavobacteriales	Flavobacteriaceae	NA	0.06	0.06	0.06	0.08	0.01	0.01	0.05	0.06	0.02	0.04	0.03	0.07
Bacteroidetes	Sphingobacteria	Sphingobacteriales	Cytophagaceae	Sporocytophaga	0.00	0.00	0.00	0.00	0.00	0.00	0.00	0.00	0.25	0.00	0.00	0.00
Bacteroidetes	NA	NA	NA	NA	0.00	0.00	0.22	0.00	0.03	0.06	0.00	0.00	0.01	0.00	0.00	0.00
Cyanobacteria	NA	NA	NA	NA	0.00	0.00	0.01	0.00	0.00	0.00	0.00	0.00	0.00	0.00	0.06	0.00
Firmicutes	Bacilli	Bacillales	Staphylococcaceae	Gemella	0.01	0.03	0.02	0.00	0.07	0.02	0.00	0.00	0.01	0.04	0.03	0.01
Firmicutes	Bacilli	Bacillales	Staphylococcaceae	Staphylococcus	0.00	0.02	0.02	0.00	0.03	0.01	0.01	0.00	0.00	0.01	0.01	0.00
Firmicutes	Bacilli	Lactobacillales	Aerococcaceae	Abiotrophia	0.18	0.34	0.16	0.29	0.86	0.29	0.35	0.13	0.22	0.29	0.12	0.64
Firmicutes	Bacilli	Lactobacillales	Aerococcaceae	Aerococcus	0.09	0.06	0.08	0.11	0.09	0.06	0.08	0.02	0.03	0.05	0.06	0.07
Firmicutes	Bacilli	Lactobacillales	Carnobacteriaceae	Desemzia	0.01	0.00	0.00	0.00	0.00	0.01	0.00	0.01	0.00	0.02	0.00	0.00
Firmicutes	Bacilli	Lactobacillales	Carnobacteriaceae	Granulicatella	4.72	3.44	2.43	4.46	7.17	3.92	2.79	2.09	2.85	3.40	2.86	2.14

Firmicutes	Bacilli	Lactobacillales	Enterococcaceae	Tetragenococcus	0.02	0.00	0.00	0.00	0.01	0.02	0.00	0.00	0.00	0.00	0.00	0.00
Firmicutes	Bacilli	Lactobacillales	Enterococcaceae	Vagococcus	0.05	0.01	0.03	0.00	0.14	0.04	0.01	0.03	0.07	0.01	0.03	0.02
Firmicutes	Bacilli	Lactobacillales	Lactobacillaceae	Lactobacillus	0.24	0.28	0.20	0.33	0.36	0.31	0.26	0.21	0.16	0.34	0.12	0.24
Firmicutes	Bacilli	Lactobacillales	Streptococcaceae	Streptococcus	28.84	31.33	28.83	25.31	37.13	32.97	30.69	39.91	30.96	32.01	32.34	26.96
Firmicutes	Bacilli	Lactobacillales	NA	NA	0.37	0.41	0.23	0.28	0.84	0.42	0.13	0.29	0.21	0.19	0.21	0.23
Firmicutes	Clostridia	Clostridiales	Eubacteriaceae	Anaerovorax	0.15	0.11	0.09	0.18	0.06	0.11	0.33	0.17	0.18	0.11	0.12	0.08
Firmicutes	Clostridia	Clostridiales	Eubacteriaceae	Mogibacterium	0.11	0.06	0.08	0.01	0.07	0.15	0.11	0.10	0.08	0.07	0.14	0.06
Firmicutes	Clostridia	Clostridiales	Lachnospiraceae	Butyrivibrio	0.04	0.00	0.03	0.02	0.02	0.01	0.03	0.00	0.03	0.01	0.01	0.00
Firmicutes	Clostridia	Clostridiales	Lachnospiraceae	Catonella	0.02	0.05	0.02	0.00	0.03	0.03	0.07	0.03	0.04	0.02	0.06	0.07
Firmicutes	Clostridia	Clostridiales	Lachnospiraceae	Moryella	0.03	0.06	0.09	0.04	0.07	0.03	0.11	0.05	0.04	0.06	0.08	0.04
Firmicutes	Clostridia	Clostridiales	Lachnospiraceae	Oribacterium	0.18	0.08	0.09	0.16	0.04	0.36	0.19	0.31	0.05	0.15	0.35	0.20
Firmicutes	Clostridia	Clostridiales	Lachnospiraceae	NA	0.43	0.38	0.18	0.34	0.36	0.26	0.27	0.16	0.33	0.31	0.48	0.31
Firmicutes	Clostridia	Clostridiales	Peptococcaceae	Peptococcus	0.01	0.02	0.00	0.01	0.00	0.01	0.00	0.01	0.00	0.00	0.00	0.01
Firmicutes	Clostridia	Clostridiales	Peptostreptococcaceae	Parvimonas	0.03	0.03	0.01	0.02	0.03	0.06	0.15	0.02	0.01	0.06	0.00	0.02
Firmicutes	Clostridia	Clostridiales	Peptostreptococcaceae	Peptostreptococcus	0.40	0.51	0.34	0.20	0.34	0.18	0.36	0.30	0.23	0.27	0.17	0.47
Firmicutes	Clostridia	Clostridiales	Ruminococcaceae	Acetivibrio	0.00	0.02	0.00	0.01	0.00	0.00	0.00	0.00	0.00	0.01	0.00	0.02
Firmicutes	Clostridia	Clostridiales	Ruminococcaceae	Ruminococcus	0.02	0.00	0.00	0.00	0.00	0.01	0.01	0.00	0.02	0.01	0.02	0.01
Firmicutes	Clostridia	Clostridiales	Ruminococcaceae	NA	0.44	0.85	0.25	0.27	0.29	0.89	0.70	0.54	0.32	0.76	0.40	0.64
Firmicutes	Clostridia	Clostridiales	Veillonellaceae	Acidaminococcus	0.12	0.12	0.20	0.16	0.12	0.18	0.10	0.08	0.07	0.10	0.15	0.19
Firmicutes	Clostridia	Clostridiales	Veillonellaceae	Propionispora	0.01	0.01	0.01	0.01	0.02	0.00	0.02	0.02	0.01	0.05	0.04	0.07
Firmicutes	Clostridia	Clostridiales	Veillonellaceae	Selenomonas	0.23	0.31	0.23	0.70	0.45	0.67	0.21	0.14	0.23	0.30	2.09	0.45
Firmicutes	Clostridia	Clostridiales	Veillonellaceae	Veillonella	0.76	1.09	1.09	1.20	0.52	1.22	1.12	2.22	0.95	1.13	1.63	1.47
Firmicutes	Clostridia	Clostridiales	Veillonellaceae	NA	10.29	12.54	13.61	15.90	5.20	15.21	13.60	20.44	11.37	15.50	18.54	17.67
Firmicutes	Clostridia	Clostridiales	NA	NA	0.04	0.08	0.10	0.12	0.08	0.05	0.10	0.03	0.12	0.11	0.17	0.14
Firmicutes	Erysipelotrichi	Erysipelotrichales	Erysipelotrichaceae	Bulleidia	0.00	0.00	0.02	0.01	0.00	0.00	0.01	0.00	0.00	0.00	0.01	0.02
Firmicutes	Erysipelotrichi	Erysipelotrichales	Erysipelotrichaceae	NA	0.11	0.19	0.02	0.08	0.11	0.06	0.24	0.13	0.20	0.08	0.26	0.18
Firmicutes	NA	NA	NA	NA	4.43	6.76	5.91	2.41	9.90	4.69	4.70	4.21	5.75	3.71	2.77	2.75
Fusobacteria	Fusobacteria	Fusobacteriales	Fusobacteriaceae	Fusobacterium	0.70	1.06	1.06	1.00	1.11	0.56	1.37	0.85	0.83	0.59	0.72	1.63
Fusobacteria	Fusobacteria	Fusobacteriales	Leptotrichiaceae	Leptotrichia	0.54	0.59	0.53	0.27	0.21	0.47	0.71	0.76	0.68	0.62	0.75	0.77
Fusobacteria	Fusobacteria	Fusobacteriales	Leptotrichiaceae	Streptobacillus	0.07	0.00	0.03	0.01	0.00	0.03	0.07	0.01	0.06	0.05	0.02	0.04
Proteobacteria	Betaproteobacteria	Burkholderiales	Alcaligenaceae	Derxia	0.08	0.08	0.04	0.04	0.05	0.02	0.05	0.03	0.17	0.11	0.04	0.13
Proteobacteria	Betaproteobacteria	Burkholderiales	Burkholderiaceae	Ralstonia	0.01	0.00	0.00	0.00	0.00	0.00	0.00	0.00	0.01	0.01	0.06	0.00
Proteobacteria	Betaproteobacteria	Burkholderiales	Comamonadaceae	Acidovorax	0.08	0.00	0.01	0.01	0.00	0.00	0.00	0.00	0.03	0.00	0.04	0.00
Proteobacteria	Betaproteobacteria	Burkholderiales	Comamonadaceae	NA	0.00	0.01	0.00	0.01	0.06	0.01	0.00	0.00	0.00	0.00	0.00	0.00
Proteobacteria	Betaproteobacteria	Burkholderiales	NA	NA	0.02	0.00	0.00	0.00	0.02	0.00	0.00	0.00	0.00	0.00	0.00	0.00
Proteobacteria	Betaproteobacteria	Neisseriales	Neisseriaceae	Chitinibacter	0.31	0.30	0.26	0.11	0.18	0.13	0.13	0.06	0.29	0.18	0.07	0.22
Proteobacteria	Betaproteobacteria	Neisseriales	Neisseriaceae	Conchiformibius	0.00	0.02	0.04	0.00	0.00	0.02	0.01	0.00	0.00	0.00	0.00	0.01

Proteobacteria	Betaproteobacteria	Neisseriales	Neisseriaceae	Kingella	0.18	0.14	0.12	0.13	0.07	0.15	0.02	0.03	0.09	0.02	0.06	0.10
Proteobacteria	Betaproteobacteria	Neisseriales	Neisseriaceae	Neisseria	8.47	7.73	11.37	8.70	4.89	4.10	3.88	1.28	5.58	7.91	2.22	7.87
Proteobacteria	Betaproteobacteria	Neisseriales	Neisseriaceae	Simonsiella	0.02	0.01	0.05	0.07	0.00	0.01	0.07	0.05	0.05	0.01	0.01	0.00
Proteobacteria	Betaproteobacteria	Neisseriales	Neisseriaceae	NA	0.09	0.16	0.17	0.03	0.18	0.09	0.14	0.02	0.15	0.05	0.06	0.17
Proteobacteria	Betaproteobacteria	Rhodocyclales	Rhodocyclaceae	Propionivibrio	0.01	0.01	0.01	0.01	0.01	0.00	0.00	0.00	0.00	0.00	0.00	0.00
Proteobacteria	Betaproteobacteria	NA	NA	NA	0.02	0.02	0.00	0.01	0.00	0.01	0.00	0.02	0.01	0.00	0.00	0.00
Proteobacteria	Epsilonproteobacteria	Campylobacterales	Campylobacteraceae	Campylobacter	0.08	0.10	0.06	0.17	0.04	0.06	0.23	0.20	0.13	0.13	0.20	0.16
Proteobacteria	Gammaproteobacteria	Alteromonadales	Alteromonadaceae	Marinobacterium	0.00	0.01	0.00	0.00	0.01	0.01	0.02	0.00	0.02	0.02	0.01	0.00
Proteobacteria	Gammaproteobacteria	Alteromonadales	Shewanellaceae	Shewanella	0.21	0.23	0.20	0.09	0.24	0.12	0.25	0.25	0.08	0.16	0.11	0.10
Proteobacteria	Gammaproteobacteria	Cardiobacteriales	Cardiobacteriaceae	Cardiobacterium	0.03	0.04	0.05	0.07	0.02	0.02	0.01	0.01	0.05	0.00	0.00	0.07
Proteobacteria	Gammaproteobacteria	Chromatiales	Chromatiaceae	Rheinheimera	0.00	0.00	0.00	0.00	0.00	0.00	0.00	0.00	0.42	0.00	0.00	0.00
Proteobacteria	Gammaproteobacteria	Enterobacteriales	Enterobacteriaceae	Enterobacter	0.04	0.21	0.10	0.03	0.02	0.15	0.13	0.05	0.06	0.13	0.10	0.09
Proteobacteria	Gammaproteobacteria	Enterobacteriales	Enterobacteriaceae	Erwinia	0.00	0.00	0.00	0.00	0.00	0.00	0.00	0.00	0.05	0.00	0.00	0.00
Proteobacteria	Gammaproteobacteria	Enterobacteriales	Enterobacteriaceae	Morganella	0.00	0.00	0.00	0.00	0.00	0.02	0.01	0.02	0.00	0.01	0.03	0.00
Proteobacteria	Gammaproteobacteria	Enterobacteriales	Enterobacteriaceae	Plesiomonas	0.00	0.01	0.02	0.00	0.01	0.01	0.01	0.00	0.00	0.04	0.00	0.00
Proteobacteria	Gammaproteobacteria	Enterobacteriales	Enterobacteriaceae	Serratia	0.00	0.01	0.01	0.01	0.00	0.00	0.01	0.01	0.00	0.00	0.00	0.00
Proteobacteria	Gammaproteobacteria	Enterobacteriales	Enterobacteriaceae	NA	0.02	0.00	0.00	0.01	0.01	0.00	0.00	0.00	0.50	0.00	0.00	0.02
Proteobacteria	Gammaproteobacteria	Methylococcales	Methylococcaceae	Methylocaldum	0.03	0.03	0.04	0.01	0.01	0.00	0.00	0.01	0.01	0.04	0.01	0.02
Proteobacteria	Gammaproteobacteria	Oceanospirillales	Oceanospirillaceae	Marinospirillum	0.02	0.06	0.10	0.03	0.02	0.05	0.10	0.03	0.02	0.03	0.05	0.08
Proteobacteria	Gammaproteobacteria	Pasteurellales	Pasteurellaceae	Actinobacillus	0.14	0.03	0.03	0.00	0.02	0.03	0.07	0.00	0.08	0.12	0.06	0.05
Proteobacteria	Gammaproteobacteria	Pasteurellales	Pasteurellaceae	Aggregatibacter	0.12	0.16	0.12	0.09	0.21	0.08	0.14	0.03	0.17	0.05	0.13	0.01
Proteobacteria	Gammaproteobacteria	Pasteurellales	Pasteurellaceae	Haemophilus	4.08	4.08	5.89	4.04	3.89	4.07	4.46	2.90	2.28	4.19	2.38	3.11
Proteobacteria	Gammaproteobacteria	Pasteurellales	Pasteurellaceae	Pasteurella	0.01	0.03	0.01	0.02	0.01	0.02	0.02	0.04	0.02	0.03	0.01	0.02
Proteobacteria	Gammaproteobacteria	Pasteurellales	Pasteurellaceae	Phocoenobacter	0.01	0.01	0.01	0.01	0.00	0.00	0.00	0.00	0.00	0.01	0.00	0.00
Proteobacteria	Gammaproteobacteria	Pasteurellales	Pasteurellaceae	NA	0.52	0.41	0.51	0.46	0.22	0.25	0.60	0.09	0.54	1.57	0.24	0.20
Proteobacteria	Gammaproteobacteria	Pseudomonadales	Moraxellaceae	Acinetobacter	7.86	0.00	0.00	0.01	0.00	0.00	0.01	0.00	4.44	0.00	0.02	0.00
Proteobacteria	Gammaproteobacteria	Pseudomonadales	Moraxellaceae	Branhamella	0.00	0.00	0.05	0.13	0.00	0.00	0.00	0.00	0.00	0.00	0.00	0.01
Proteobacteria	Gammaproteobacteria	Pseudomonadales	Moraxellaceae	Enhydrobacter	0.00	0.00	0.00	0.00	0.00	0.00	0.00	0.00	0.25	0.00	0.03	0.00
Proteobacteria	Gammaproteobacteria	Pseudomonadales	Moraxellaceae	Moraxella	0.01	0.00	0.00	0.00	0.00	0.00	1.20	0.00	0.01	0.05	0.01	0.00
Proteobacteria	Gammaproteobacteria	Pseudomonadales	Moraxellaceae	Psychrobacter	0.00	0.00	0.00	0.00	0.00	0.00	0.16	0.00	0.00	0.00	0.00	0.00
Proteobacteria	Gammaproteobacteria	Pseudomonadales	Moraxellaceae	NA	0.00	0.00	0.03	0.36	0.00	0.01	0.00	0.00	0.00	0.06	0.00	0.00
Proteobacteria	Gammaproteobacteria	Pseudomonadales	Pseudomonadaceae	Pseudomonas	0.00	0.00	0.00	0.00	0.00	0.00	0.00	0.00	5.22	0.00	0.00	0.00
Proteobacteria	Gammaproteobacteria	Pseudomonadales	Pseudomonadaceae	NA	0.05	0.01	0.03	0.06	0.04	0.04	0.03	0.02	0.16	0.01	0.02	0.02
Proteobacteria	Gammaproteobacteria	Pseudomonadales	NA	NA	0.00	0.00	0.01	0.00	0.01	0.01	0.00	0.00	0.02	0.00	0.00	0.02
Proteobacteria	Gammaproteobacteria	Vibrionales	Vibrionaceae	Enterovibrio	0.02	0.00	0.01	0.00	0.02	0.00	0.00	0.00	0.00	0.00	0.00	0.02
Proteobacteria	Gammaproteobacteria	Vibrionales	Vibrionaceae	Salinivibrio	0.05	0.14	0.08	0.03	0.07	0.12	0.10	0.10	0.10	0.08	0.07	0.05
Proteobacteria	Gammaproteobacteria	Vibrionales	Vibrionaceae	Vibrio	0.03	0.05	0.03	0.00	0.00	0.00	0.00	0.00	0.04	0.06	0.01	0.04

Proteobacteria	Gammaproteobacteria	NA	NA	NA	0.09	0.06	0.14	0.03	0.06	0.07	0.19	0.14	0.08	0.06	0.05	0.11
Spirochaetes	Spirochaetes	Spirochaetales	Spirochaetaceae	Treponema	0.06	0.00	0.02	0.02	0.04	0.04	0.09	0.00	0.01	0.01	0.01	0.01
TM7	NA	NA	NA	NA	0.47	1.01	0.43	0.99	0.60	0.70	0.76	0.69	0.64	0.62	0.61	1.40
NA	NA	NA	NA	NA	0.01	0.03	0.01	0.00	0.03	0.01	0.00	0.02	0.04	0.01	0.01	0.03
