# Supplementary material for: Exploring the oral microbiota of children at various developmental stages of their dentition in the relation to their oral health
Source: BMC Med Genomics. 2011 Mar 4;4:22. doi: 10.1186/1755-8794-4-22 (PMC3058002; doi:10.1186/1755-8794-4-22)
Supplement: Additional file 2 — Rarefaction plot of the unique sequences in saliva samples of three oral health groups of children with early mixed dentition (healthy, treated or with caries). This is a rarefaction plot of all unique sequences by the number of sequences sampled in children with early mixed dentition by their oral health status. [file 1755-8794-4-22-S2.PPTX]

## Slide 1
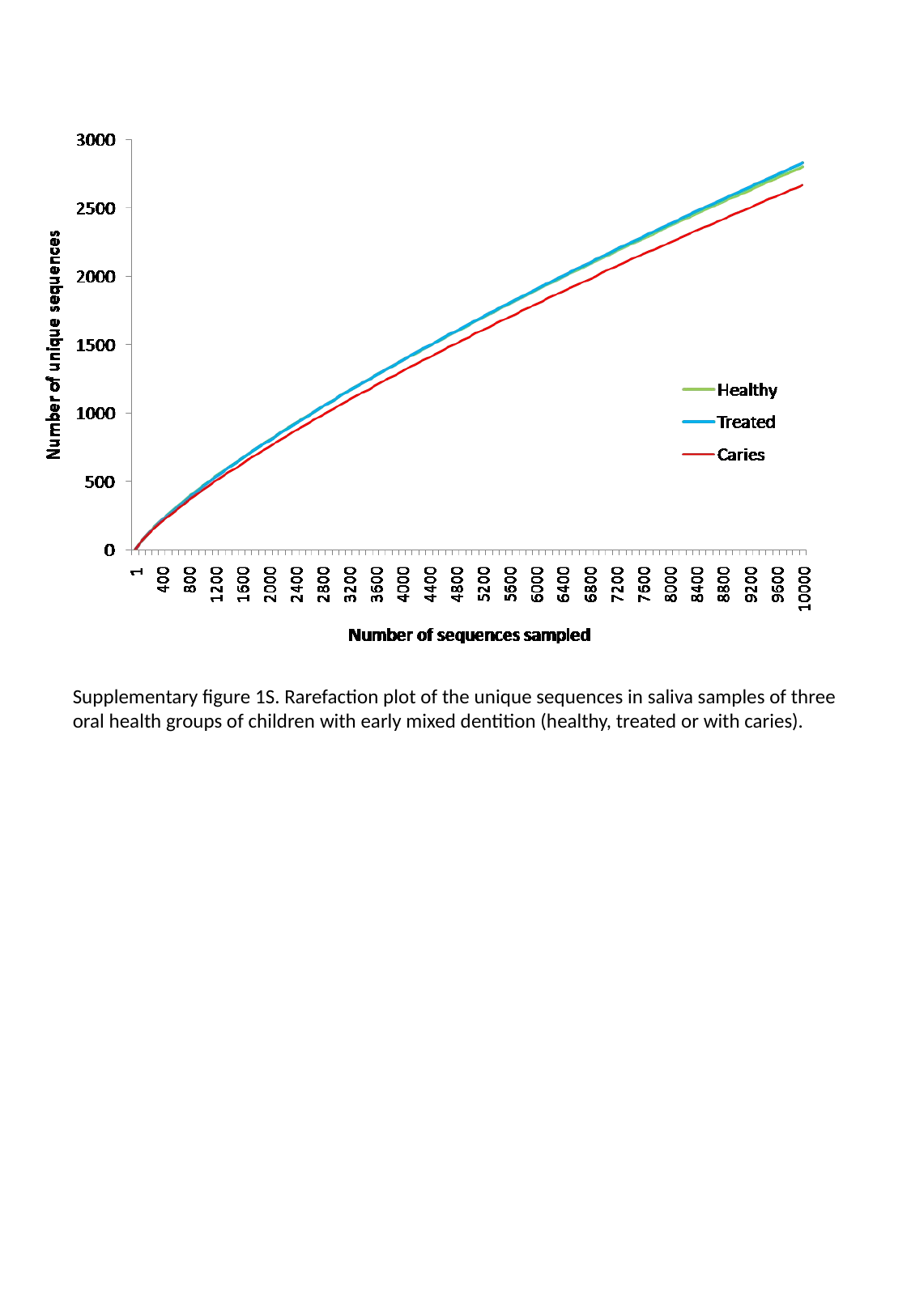

Supplementary figure 1S. Rarefaction plot of the unique sequences in saliva samples of three oral health groups of children with early mixed dentition (healthy, treated or with caries).
