# Supplementary material for: Exploring the oral microbiota of children at various developmental stages of their dentition in the relation to their oral health
Source: BMC Med Genomics. 2011 Mar 4;4:22. doi: 10.1186/1755-8794-4-22 (PMC3058002; doi:10.1186/1755-8794-4-22)
Supplement: Additional file 4 — Full list of the microarray 16 S rDNA probes and their targets used in the microarray. This file lists all 16 S rDNA probes used in the microarray, probe sequences and their targets as identified by RDP blast search. [file 1755-8794-4-22-S4.PDF]

Probe nr	Probe name	Selected probe	domain	phylum	class	order	family	genus	species (max 20 shown)
o1310	o1310 16S-AbiDef	AGGTGCTTGCACTTGGTCAA	Bacteria	Firmicutes	Bacilli	Lactobacillales	Aerococcaceae	Abiotrophia	Abiotrophia defectiva; 00394037; AY879307
			Bacteria	Firmicutes	Bacilli	Lactobacillales	Aerococcaceae	Abiotrophia	uncultured Abiotrophia sp.; 501D03(oral); AM420170
			Bacteria	Firmicutes	Bacilli	Lactobacillales	Aerococcaceae	Abiotrophia	uncultured Streptococcus sp.; EHFS1_S16c; EU071531
			Bacteria	Firmicutes	Bacilli	Lactobacillales	Aerococcaceae	Abiotrophia	Abiotrophia sp. oral clone P4PA_155 P1; AY207063
			Bacteria	Firmicutes	Bacilli	Lactobacillales	Aerococcaceae	Abiotrophia	uncultured bacterium; P2D1-718; EF510367
			Bacteria	Firmicutes	Bacilli	Lactobacillales	Aerococcaceae	Abiotrophia	uncultured bacterium; nbw634f04c1; GQ113680
			Bacteria	Firmicutes	Bacilli	Lactobacillales	Aerococcaceae	Abiotrophia	uncultured bacterium; nbw582f01c1; GQ111972
			Bacteria	Firmicutes	Bacilli	Lactobacillales	Aerococcaceae	Abiotrophia	uncultured bacterium; nbw02f11c1; GQ060515
			Bacteria	Firmicutes	Bacilli	Lactobacillales	Aerococcaceae	Abiotrophia	uncultured bacterium; nbw633f09c1; GQ113074
			Bacteria	Firmicutes	Bacilli	Lactobacillales	Aerococcaceae	Abiotrophia	uncultured bacterium; nbw636g09c1; GQ113848
			Bacteria	Firmicutes	Bacilli	Lactobacillales	Aerococcaceae	Abiotrophia	uncultured Abiotrophia sp.; 7BB825; FJ976282
			Bacteria	Firmicutes	Bacilli	Lactobacillales	Aerococcaceae	Abiotrophia	uncultured bacterium; nbw03g04c1; GQ060655
			Bacteria	Firmicutes	Bacilli	Lactobacillales	Aerococcaceae	Abiotrophia	Abiotrophia defectiva (T); GIFU12707 (ATCC49176); D50541
o1311	o1311 16S-Achea	TTAAAGGAATTGGCGGGGG	Archaea	Crenarchaeota	Thermoprotei	Thermoproteales	Thermoproteaceae	Pyrobaculum	Pyrobaculum aerophilum (T); im2; L07510
			Archaea	Crenarchaeota	Thermoprotei	Thermoproteales	Thermoproteaceae	Thermocladium	Thermocladium modestius (T); IC-125; AB005296
			Archaea	Crenarchaeota	Thermoprotei	Thermoproteales	Thermoproteaceae	Pyrobaculum	Thermoproteus tenax; M35966
			Archaea	Crenarchaeota	Thermoprotei	Thermoproteales	Thermoproteaceae	Pyrobaculum	Pyrobaculum calidifontis; VA1; AB078332
			Archaea	Crenarchaeota	Thermoprotei	Desulfurococcales	Desulfurococcaceae	Staphylothermus	Staphylothermus marinus (T); F1 - DSM 3639; X99560
			Archaea	Crenarchaeota	Thermoprotei	Desulfurococcales	Desulfurococcaceae	Ignicoccus	Ignicoccus islandicus (T); Ko18; X99562
			Archaea	Crenarchaeota	Thermoprotei	Desulfurococcales	Desulfurococcaceae	Thermosphaera	Thermosphaera aggregans (T); M11TL; X99556
			Archaea	Crenarchaeota	Thermoprotei	Thermoproteales	Thermoproteaceae	Pyrobaculum	Pyrobaculum oguniense (T); TE7; AB029339
			Archaea	Crenarchaeota	Thermoprotei	Thermoproteales	Thermoproteaceae	Pyrobaculum	Pyrobaculum oguniense; TE7; AB087402
			Archaea	Crenarchaeota	Thermoprotei	Thermoproteales	Thermoproteaceae	Pyrobaculum	Pyrobaculum neutrophilum; NZ1; X81886
			Archaea	Crenarchaeota	Thermoprotei	Thermoproteales	Thermoproteaceae	Pyrobaculum	Thermoproteus sp. IC-033; AB009616
			Archaea	Crenarchaeota	Thermoprotei	Desulfurococcales	Desulfurococcaceae	Staphylothermus	Staphylothermus hellenicus (T); P8(BK20S6-10-b1); AJ012645
			Archaea	Crenarchaeota	Thermoprotei	Thermoproteales	Thermoproteaceae	Pyrobaculum	uncultured crenarchaeote pBA2; AF176346
			Archaea	Crenarchaeota	Thermoprotei	Thermoproteales	Thermofilaceae	Thermofilum	Thermofilaceae archaeon SRI-370; AF255605
			Archaea	Crenarchaeota	Thermoprotei	Thermoproteales	Thermoproteaceae	Pyrobaculum	Pyrobaculum islandicum (T); geo3; L07511
			Archaea	Crenarchaeota	Thermoprotei	Desulfurococcales	Desulfurococcaceae	Thermodiscus	Thermodiscus maritimus (T); S2; X99554
			Archaea	Crenarchaeota	Thermoprotei	Desulfurococcales	Desulfurococcaceae	Aeropyrum	Aeropyrum camini (T); SY1; AB109559
			Archaea	Crenarchaeota	Thermoprotei	Thermoproteales	Thermoproteaceae	Pyrobaculum	Pyrobaculum arsenaticum (T); PZ6; AJ277124
			Archaea	Crenarchaeota	Thermoprotei	Desulfurococcales	Desulfurococcaceae	Ignicoccus	Ignicoccus pacificus (T); LPC33; AJ271794
			Archaea	Crenarchaeota	Thermoprotei	Thermoproteales	Thermoproteaceae	Pyrobaculum	Pyrobaculum sp. WIJ3; AJ277125
o1313	o1313 16S-ActGroup1	TTGCTGGTTCTGGATGAGTG	Bacteria	Actinobacteria	Actinobacteria	Actinomycetales	Actinomycetaceae	Actinomyces	Actinomyces sp. oral clone IO077; AY349364
			Bacteria	Actinobacteria	Actinobacteria	Actinomycetales	Actinomycetaceae	Actinomyces	uncultured bacterium; A_D_03_85; FJ470516
			Bacteria	Actinobacteria	Actinobacteria	Actinomycetales	Actinomycetaceae	Actinomyces	Actinomyces sp. oral clone GU067; AY349362
			Bacteria	Actinobacteria	Actinobacteria	Actinomycetales	Actinomycetaceae	Actinomyces	Actinomyces naeslundii; AJ635359
			Bacteria	Actinobacteria	Actinobacteria	Actinomycetales	Actinomycetaceae	Actinomyces	uncultured Actinomyces sp.; 201G06(oral); AM420034
			Bacteria	Actinobacteria	Actinobacteria	Actinomycetales	Actinomycetaceae	Actinomyces	uncultured Actinomyces sp.; 3.68k; DQ016919
			Bacteria	Actinobacteria	Actinobacteria	Actinomycetales	Actinomycetaceae	Actinomyces	Actinomyces viscosus; ChDC OS34; AF543286
			Bacteria	Actinobacteria	Actinobacteria	Actinomycetales	Actinomycetaceae	Actinomyces	uncultured bacterium; BF0001B009; AM696983
			Bacteria	Actinobacteria	Actinobacteria	Actinomycetales	Actinomycetaceae	Actinomyces	Actinomyces sp. oral clone EP005; AY008314
			Bacteria	Actinobacteria	Actinobacteria	Actinomycetales	Actinomycetaceae	Actinomyces	uncultured bacterium; Sed4; EF064162
			Bacteria	Actinobacteria	Actinobacteria	Actinomycetales	Actinomycetaceae	Actinomyces	uncultured bacterium; FA04B03; FM872615
			Bacteria	Actinobacteria	Actinobacteria	Actinomycetales	Actinomycetaceae	Actinomyces	Actinomyces sp. ChDC B197; AF543275
			Bacteria	Actinobacteria	Actinobacteria	Actinomycetales	Actinomycetaceae	Actinomyces	uncultured bacterium; MA01B02; FM873522
			Bacteria	Actinobacteria	Actinobacteria	Actinomycetales	Actinomycetaceae	Actinomyces	Actinomyces sp. ChDC OS47; AF543297
			Bacteria	Actinobacteria	Actinobacteria	Actinomycetales	Actinomycetaceae	Actinomyces	uncultured bacterium; BF0002C047; AM697350
			Bacteria	Actinobacteria	Actinobacteria	Actinomycetales	Actinomycetaceae	Actinomyces	uncultured bacterium; MC01H07; FM874123
			Bacteria	Actinobacteria	Actinobacteria	Actinomycetales	Actinomycetaceae	Actinomyces	uncultured bacterium; SA04F10; FM874888
			Bacteria	Actinobacteria	Actinobacteria	Actinomycetales	Actinomycetaceae	Actinomyces	Actinomyces sp. oral clone AG004; AF287747
			Bacteria	Actinobacteria	Actinobacteria	Actinomycetales	Actinomycetaceae	Actinomyces	Actinomyces viscosus (T); NCTC 10951; X82453
			Bacteria	Actinobacteria	Actinobacteria	Actinomycetales	Actinomycetaceae	Actinomyces	Actinomyces naeslundii; CCUG 34285; AJ234050
o1314	o1314 16S-ActGroup2	CGGGGTTTCCTCCTTTGTG	Bacteria	Actinobacteria	Actinobacteria	Actinomycetales	Actinomycetaceae	Actinomyces	Actinomyces naeslundii; ChDC B204; AF543282
			Bacteria	Actinobacteria	Actinobacteria	Actinomycetales	Actinomycetaceae	Actinomyces	Actinomyces sp. oral clone AP064; AF287749
			Bacteria	Actinobacteria	Actinobacteria	Actinomycetales	Actinomycetaceae	Actinomyces	Actinomyces oris; P6N; GQ421318
			Bacteria	Actinobacteria	Actinobacteria	Actinomycetales	Actinomycetaceae	Actinomyces	Actinomyces naeslundii; NCTC 10301; X81062
			Bacteria	Actinobacteria	Actinobacteria	Actinomycetales	Actinomycetaceae	Actinomyces	uncultured bacterium; nbw458c04c1; GQ066497
			Bacteria	Actinobacteria	Actinobacteria	Actinomycetales	Actinomycetaceae	Actinomyces	uncultured bacterium; nbw460f01c1; GQ066674
			Bacteria	Actinobacteria	Actinobacteria	Actinomycetales	Actinomycetaceae	Actinomyces	uncultured bacterium; nbw458e05c1; GQ066517
			Bacteria	Actinobacteria	Actinobacteria	Actinomycetales	Actinomycetaceae	Actinomyces	uncultured bacterium; nbw1122a11c1; GQ067098
			Bacteria	Actinobacteria	Actinobacteria	Actinomycetales	Actinomycetaceae	Actinomyces	uncultured bacterium; nbw1156b11c1; GQ077217
			Bacteria	Actinobacteria	Actinobacteria	Actinomycetales	Actinomycetaceae	Actinomyces	uncultured bacterium; nbw200c06c1; GQ072716
			Bacteria	Actinobacteria	Actinobacteria	Actinomycetales	Actinomycetaceae	Actinomyces	uncultured bacterium; nbw1155e01c1; GQ076938
			Bacteria	Actinobacteria	Actinobacteria	Actinomycetales	Actinomycetaceae	Actinomyces	uncultured bacterium; nbw227e12c1; GQ069014
			Bacteria	Actinobacteria	Actinobacteria	Actinomycetales	Actinomycetaceae	Actinomyces	uncultured bacterium; nbw145c02c1; GQ067994
			Bacteria	Actinobacteria	Actinobacteria	Actinomycetales	Actinomycetaceae	Actinomyces	uncultured bacterium; nbw208h11c1; GQ073428
o1315	o1315 16S-ActGroup3	GCATTTAGTTGGTCGTGTGC	Bacteria	Actinobacteria	Actinobacteria	Actinomycetales	Actinomycetaceae	Actinomyces	Actinomyces sp. oral strain Hal-1065; AF385521
			Bacteria	Actinobacteria	Actinobacteria	Actinomycetales	Actinomycetaceae	Actinomyces	uncultured Actinomyces sp.; EHFS1_S032; EU071474
			Bacteria	Actinobacteria	Actinobacteria	Actinomycetales	Actinomycetaceae	Actinomyces	Actinomyces genomosp. C2; C3M24; AY278611
			Bacteria	Actinobacteria	Actinobacteria	Actinomycetales	Actinomycetaceae	Actinomyces	Actinomyces sp. oral strain Hal-1083; AF385522
			Bacteria	Actinobacteria	Actinobacteria	Actinomycetales	Actinomycetaceae	Actinomyces	Actinomyces sp. oral strain C29KA; AF287751
			Bacteria	Actinobacteria	Actinobacteria	Actinomycetales	Actinomycetaceae	Actinomyces	uncultured bacterium; LC1022b39; DQ228573
			Bacteria	Actinobacteria	Actinobacteria	Actinomycetales	Actinomycetaceae	Actinomyces	Actinomyces lingnae; CCUG 32865; AJ271894
			Bacteria	Actinobacteria	Actinobacteria	Actinomycetales	Actinomycetaceae	Actinomyces	Actinomyces odontolyticus; CCUG 20536T; AJ234040
			Bacteria	Actinobacteria	Actinobacteria	Actinomycetales	Actinomycetaceae	Actinomyces	Actinomyces odontolyticus; CCUG 32834; AJ234042
			Bacteria	Actinobacteria	Actinobacteria	Actinomycetales	Actinomycetaceae	Actinomyces	Actinomyces turicensis; APL11; X78721
			Bacteria	Actinobacteria	Actinobacteria	Actinomycetales	Actinomycetaceae	Actinomyces	Actinomyces odontolyticus; CCUG 33767; AJ234047
			Bacteria	Actinobacteria	Actinobacteria	Actinomycetales	Actinomycetaceae	Actinomyces	Actinomyces odontolyticus (T); NCTC 9935; X80504
			Bacteria	Actinobacteria	Actinobacteria	Actinomycetales	Actinomycetaceae	Actinomyces	Actinomyces odontolyticus; AJ234037
			Bacteria	Actinobacteria	Actinobacteria	Actinomycetales	Actinomycetaceae	Actinomyces	uncultured bacterium; nbu58b10c1; GQ003855
			Bacteria	Actinobacteria	Actinobacteria	Actinomycetales	Actinomycetaceae	Actinomyces	uncultured bacterium; nbu105a03c1; GQ000541
			Bacteria	Actinobacteria	Actinobacteria	Actinomycetales	Actinomycetaceae	Actinomyces	uncultured bacterium; nbu85h05c1; GQ006638
			Bacteria	Actinobacteria	Actinobacteria	Actinomycetales	Actinomycetaceae	Actinomyces	uncultured bacterium; TS51_a03d10; FJ369670
			Bacteria	Actinobacteria	Actinobacteria	Actinomycetales	Actinomycetaceae	Actinomyces	Actinomyces odontolyticus; CCUG 34689; AJ234054
			Bacteria	Actinobacteria	Actinobacteria	Actinomycetales	Actinomycetaceae	Actinomyces	Actinomyces odontolyticus; CCUG 32897; AJ234043
			Bacteria	Actinobacteria	Actinobacteria	Actinomycetales	Actinomycetaceae	Actinomyces	uncultured bacterium; NH01; AY978941

o1316	o1316 16S-ActGroup4	AGTTCGGATCGGTGTCTGC	Bacteria	Actinobacteria	Actinobacteria	Actinomycetales	Actinomycetaceae	Actinomyces	Actinomyces sp.; M2231/94/1; AJ234063
			Bacteria	Actinobacteria	Actinobacteria	Actinomycetales	Actinomycetaceae	Actinomyces	Actinomyces naeslundii; CCUG 33519; AJ234045
			Bacteria	Actinobacteria	Actinobacteria	Actinomycetales	Actinomycetaceae	Actinomyces	Actinomyces naeslundii; NCTC 10301; X81062
			Bacteria	Actinobacteria	Actinobacteria	Actinomycetales	Actinomycetaceae	Actinomyces	Actinomyces slackii; CCUG 32792; clone 2; AJ234067
			Bacteria	Actinobacteria	Actinobacteria	Actinomycetales	Actinomycetaceae	Actinomyces	Actinomyces viscosus; CCUG 35332; AJ234056
			Bacteria	Actinobacteria	Actinobacteria	Actinomycetales	Actinomycetaceae	Actinomyces	Actinomyces viscosus (T); NCTC 10951; X82453
			Bacteria	Actinobacteria	Actinobacteria	Actinomycetales	Actinomycetaceae	Actinomyces	Actinomyces naeslundii; CCUG 33914; AJ234048
			Bacteria	Actinobacteria	Actinobacteria	Actinomycetales	Actinomycetaceae	Actinomyces	Actinomyces urogenitalis (T); AJ243791
			Bacteria	Actinobacteria	Actinobacteria	Actinomycetales	Actinomycetaceae	Actinomyces	Actinomyces naeslundii; CCUG 34286; clone 2; AJ234052
			Bacteria	Actinobacteria	Actinobacteria	Actinomycetales	Actinomycetaceae	Actinomyces	Actinomyces slackii; CCUG 32792; clone 1; AJ234066
			Bacteria	Actinobacteria	Actinobacteria	Actinomycetales	Actinomycetaceae	Actinomyces	Actinomyces sp.; ATCC 49338; X81063
			Bacteria	Actinobacteria	Actinobacteria	Actinomycetales	Actinomycetaceae	Actinomyces	Actinomyces israelii (T); C.I.P. 103259T; X82450
			Bacteria	Actinobacteria	Actinobacteria	Actinomycetales	Actinomycetaceae	Actinomyces	Actinomyces denticolens (T); NCTC 11490; X80412
			Bacteria	Actinobacteria	Actinobacteria	Actinomycetales	Actinomycetaceae	Actinomyces	Actinomyces naeslundii; CCUG 34286; clone 1; AJ234051
			Bacteria	Actinobacteria	Actinobacteria	Actinomycetales	Actinomycetaceae	Actinomyces	Actinomyces howellii (T); NCTC 11636; X80411
			Bacteria	Actinobacteria	Actinobacteria	Actinomycetales	Actinomycetaceae	Actinomyces	Actinomyces radicidentis (T); CCUG 36733T; AJ251986
			Bacteria	Actinobacteria	Actinobacteria	Actinomycetales	Actinomycetaceae	Actinomyces	Actinomyces gerencseriae (T); DSM 6844; X80414
			Bacteria	Actinobacteria	Actinobacteria	Actinomycetales	Actinomycetaceae	Actinomyces	Actinomyces viscosus; CCUG 34288; AJ234053
			Bacteria	Actinobacteria	Actinobacteria	Actinomycetales	Actinomycetaceae	Actinomyces	Actinomyces sp.; CCUG 33931; AJ234049
			Bacteria	Actinobacteria	Actinobacteria	Actinomycetales	Actinomycetaceae	Actinomyces	Actinomyces slackii (T); NCTC 11923; X82452
o1317	o1317 16S-ActGroup5	TGAAATCCTCTGGCTCAGCT	Bacteria	Actinobacteria	Actinobacteria	Actinomycetales	Actinomycetaceae	Actinomyces	Actinomyces sp. oral taxon 180; F0310; GQ131415
			Bacteria	Actinobacteria	Actinobacteria	Actinomycetales	Actinomycetaceae	Actinomyces	uncultured bacterium; FA04H03; FM872662
			Bacteria	Actinobacteria	Actinobacteria	Actinomycetales	Actinomycetaceae	Actinomyces	uncultured bacterium; FA02F04; FM872699
			Bacteria	Actinobacteria	Actinobacteria	Actinomycetales	Actinomycetaceae	Actinomyces	Actinomyces massiliensis; 4401292; EF558367
			Bacteria	Actinobacteria	Actinobacteria	Actinomycetales	Actinomycetaceae	Actinomyces	Actinomyces lingnae; CCUG 32865; AJ271894
			Bacteria	Actinobacteria	Actinobacteria	Actinomycetales	Actinomycetaceae	Actinomyces	Actinomyces israelii; A1; AF479270
			Bacteria	Actinobacteria	Actinobacteria	Actinomycetales	Actinomycetaceae	Actinomyces	Actinomyces israelii (T); C.I.P. 103259T; X82450
			Bacteria	Actinobacteria	Actinobacteria	Actinomycetales	Actinomycetaceae	Actinomyces	Actinomyces odontolyticus; CCUG 32834; AJ234042
			Bacteria	Actinobacteria	Actinobacteria	Actinomycetales	Actinomycetaceae	Actinomyces	Actinomyces gerencseriae (T); DSM 6844; X80414
			Bacteria	Actinobacteria	Actinobacteria	Actinomycetales	Actinomycetaceae	Actinomyces	uncultured Actinomyces sp.; 10B569; FJ976310
			Bacteria	Actinobacteria	Actinobacteria	Actinomycetales	Actinomycetaceae	Actinomyces	uncultured Actinomyces sp.; 6BB471; FJ976253
			Bacteria	Actinobacteria	Actinobacteria	Actinomycetales	Actinomycetaceae	Actinomyces	uncultured Actinomyces sp.; 10B152; FJ976300
			Bacteria	Actinobacteria	Actinobacteria	Actinomycetales	Actinomycetaceae	Actinomyces	uncultured bacterium; nbu104f08c1; GQ000517
			Bacteria	Actinobacteria	Actinobacteria	Actinomycetales	Actinomycetaceae	Actinomyces	uncultured bacterium; nbw708h11c1; GQ103039
			Bacteria	Actinobacteria	Actinobacteria	Actinomycetales	Actinomycetaceae	Actinomyces	uncultured bacterium; nbw207d05c1; GQ073294
			Bacteria	Actinobacteria	Actinobacteria	Actinomycetales	Actinomycetaceae	Actinomyces	uncultured bacterium; nbw495b03c1; GQ101439
			Bacteria	Actinobacteria	Actinobacteria	Actinomycetales	Actinomycetaceae	Actinomyces	uncultured bacterium; nbw496e09c1; GQ101562
o1319	o1319 16S-Actin	GGGTCAACTCGGAGGAAG	Bacteria	Actinobacteria	Actinobacteria	Actinomycetales	Micrococcaceae	Arthrobacter	Arthrobacter ilicis (T); DSM 20138; X83407
			Bacteria	Actinobacteria	Actinobacteria	Actinomycetales	Micrococcaceae	Arthrobacter	Arthrobacter citreus (T); DSM 20133; X80737
			Bacteria	Actinobacteria	Actinobacteria	Actinomycetales	Micrococcaceae	Arthrobacter	Arthrobacter pascens (T); DSM 20545; X80740
			Bacteria	Actinobacteria	Actinobacteria	Actinomycetales	Micrococcaceae	Arthrobacter	Arthrobacter ramosus (T); DSM 20546; X80742
			Bacteria	Actinobacteria	Actinobacteria	Actinomycetales	Micrococcaceae	Arthrobacter	Arthrobacter aurescens (T); DSM20116; X83405
			Bacteria	Actinobacteria	Actinobacteria	Actinomycetales	Micrococcaceae	Arthrobacter	Arthrobacter globiformis (T); DSM 20124; X80736
			Bacteria	Actinobacteria	Actinobacteria	Actinomycetales	Micrococcaceae	Arthrobacter	Arthrobacter crystallopoietes (T); DSM 20117; X80738
			Bacteria	Actinobacteria	Actinobacteria	Actinomycetales	Micrococcaceae	Arthrobacter	Arthrobacter uratoxydans (T); DSM 20647; X83410
			Bacteria	Actinobacteria	Actinobacteria	Actinomycetales	Micrococcaceae	Arthrobacter	Sinomonas atrocyanea (T); DSM 20127; X80746
			Bacteria	Actinobacteria	Actinobacteria	Actinomycetales	Actinomycetaceae	Actinomyces	Actinomyces sp.; M2231/94/1; AJ234063
			Bacteria	Actinobacteria	Actinobacteria	Actinomycetales	Actinomycetaceae	Actinomyces	Actinomyces naeslundii; CCUG 33519; AJ234045
			Bacteria	Actinobacteria	Actinobacteria	Actinomycetales	Actinomycetaceae	Actinomyces	Actinomyces naeslundii; NCTC 10301; X81062
			Bacteria	Actinobacteria	Actinobacteria	Actinomycetales	Actinomycetaceae	Actinomyces	Actinomyces viscosus; CCUG 35332; AJ234056
			Bacteria	Actinobacteria	Actinobacteria	Actinomycetales	Actinomycetaceae	Actinomyces	Actinomyces viscosus (T); NCTC 10951; X82453
			Bacteria	Actinobacteria	Actinobacteria	Actinomycetales	Actinomycetaceae	Actinomyces	Actinomyces urogenitalis (T); AJ243791
			Bacteria	Actinobacteria	Actinobacteria	Actinomycetales	Actinomycetaceae	Actinomyces	Actinomyces slackii; CCUG 32792; clone 1; AJ234066
			Bacteria	Actinobacteria	Actinobacteria	Actinomycetales	Actinomycetaceae	Actinomyces	Actinomyces viscosus; CCUG 34288; AJ234053
			Bacteria	Actinobacteria	Actinobacteria	Actinomycetales	Actinomycetaceae	Actinomyces	Actinomyces sp.; CCUG 33931; AJ234049
			Bacteria	Actinobacteria	Actinobacteria	Actinomycetales	Actinomycetaceae	Actinomyces	Actinomyces slackii (T); NCTC 11923; X82452
			Bacteria	Actinobacteria	Actinobacteria	Actinomycetales	Actinomycetaceae	Actinomyces	Actinomyces graevenitzii; CCUG 27294; Y09589
o1320	o1320 16S-ActJun	GAGGAGGCTACTGAGACTAA	Bacteria	Proteobacteria	Gammaproteobacteria	Pseudomonadales	Moraxellaceae	Acinetobacter	Acinetobacter junii; S33; AB101444
			Bacteria	Proteobacteria	Gammaproteobacteria	Pseudomonadales	Moraxellaceae	Acinetobacter	uncultured bacterium; BE16FW031401IDW-SAK37; DQ088754
			Bacteria	Proteobacteria	Gammaproteobacteria	Pseudomonadales	Moraxellaceae	Acinetobacter	uncultured bacterium; PDB-OTU1; AY700608
			Bacteria	Proteobacteria	Gammaproteobacteria	Pseudomonadales	Moraxellaceae	Acinetobacter	uncultured bacterium; EV818BHEB5102502DRLWq27f037; DQ256330
			Bacteria	Proteobacteria	Gammaproteobacteria	Pseudomonadales	Moraxellaceae	Acinetobacter	uncultured bacterium; EV818SWSAP49; DQ337079
			Bacteria	Proteobacteria	Gammaproteobacteria	Pseudomonadales	Moraxellaceae	Acinetobacter	uncultured bacterium; ELB19-080; DQ015783
			Bacteria	Proteobacteria	Gammaproteobacteria	Pseudomonadales	Moraxellaceae	Acinetobacter	uncultured bacterium; s1s9a; DQ068891
			Bacteria	Proteobacteria	Gammaproteobacteria	Pseudomonadales	Moraxellaceae	Acinetobacter	uncultured bacterium; DR9IPCB16SCT1; AY604050
			Bacteria	Proteobacteria	Gammaproteobacteria	Pseudomonadales	Moraxellaceae	Acinetobacter	Acinetobacter junii (T); DSM 6964; X81664
			Bacteria	Proteobacteria	Gammaproteobacteria	Pseudomonadales	Moraxellaceae	Acinetobacter	Acinetobacter junii; DSM 1532; X81658
			Bacteria	Proteobacteria	Gammaproteobacteria	Pseudomonadales	Moraxellaceae	Acinetobacter	Acinetobacter junii; BB1C; AJ785004
			Bacteria	Proteobacteria	Gammaproteobacteria	Pseudomonadales	Moraxellaceae	Acinetobacter	Acinetobacter junii; BB1A; AJ786647
			Bacteria	Proteobacteria	Gammaproteobacteria	Pseudomonadales	Moraxellaceae	Acinetobacter	Acinetobacter junii; AF417863
			Bacteria	Proteobacteria	Gammaproteobacteria	Pseudomonadales	Moraxellaceae	Acinetobacter	Acinetobacter sp. 11; AY177359
			Bacteria	Proteobacteria	Gammaproteobacteria	Pseudomonadales	Moraxellaceae	Acinetobacter	gamma proteobacterium HTC042; AB010848
			Bacteria	Proteobacteria	Gammaproteobacteria	Pseudomonadales	Moraxellaceae	Acinetobacter	Acinetobacter junii; CCF00021; AY972479
			Bacteria	Proteobacteria	Gammaproteobacteria	Pseudomonadales	Moraxellaceae	Acinetobacter	Acinetobacter junii; ATCC 17908T; DNA Group five; Z93438
			Bacteria	Proteobacteria	Gammaproteobacteria	Pseudomonadales	Moraxellaceae	Acinetobacter	gamma proteobacterium HTC038; AB010849
			Bacteria	Proteobacteria	Gammaproteobacteria	Pseudomonadales	Moraxellaceae	Acinetobacter	uncultured bacterium; G3-6; AM179903
			Bacteria	Proteobacteria	Gammaproteobacteria	Pseudomonadales	Moraxellaceae	Acinetobacter	Acinetobacter sp. pjj-1; DQ322071
o1321	o1321 16S-ActNae	CTGGGAAAGATTGCGCCTTT	Bacteria	Actinobacteria	Actinobacteria	Actinomycetales	Actinomycetaceae	Actinomyces	Actinomyces sp. oral clone JA063; AY349367
			Bacteria	Actinobacteria	Actinobacteria	Actinomycetales	Actinomycetaceae	Actinomyces	uncultured bacterium; BF0002B107; AM697157
			Bacteria	Actinobacteria	Actinobacteria	Actinomycetales	Actinomycetaceae	Actinomyces	Actinomyces naeslundii; AB062278
			Bacteria	Actinobacteria	Actinobacteria	Actinomycetales	Actinomycetaceae	Actinomyces	Actinomyces naeslundii; NCTC 10301; X81062
			Bacteria	Actinobacteria	Actinobacteria	Actinomycetales	Actinomycetaceae	Actinomyces	Actinomyces naeslundii; GiTB; FJ823149
			Bacteria	Actinobacteria	Actinobacteria	Actinomycetales	Actinomycetaceae	Actinomyces	Actinomyces oris; WE8B-23; GQ421313
			Bacteria	Actinobacteria	Actinobacteria	Actinomycetales	Actinomycetaceae	Actinomyces	uncultured bacterium; nbw636b06c1; GQ113808
			Bacteria	Actinobacteria	Actinobacteria	Actinomycetales	Actinomycetaceae	Actinomyces	uncultured bacterium; nbw636b08c1; GQ113810
			Bacteria	Actinobacteria	Actinobacteria	Actinomycetales	Actinomycetaceae	Actinomyces	uncultured bacterium; nbw162d10c1; GQ074395
			Bacteria	Actinobacteria	Actinobacteria	Actinomycetales	Actinomycetaceae	Actinomyces	uncultured bacterium; nbw458c04c1; GQ066497
			Bacteria	Actinobacteria	Actinobacteria	Actinomycetales	Actinomycetaceae	Actinomyces	uncultured bacterium; nbw460f01c1; GQ066674

			Bacteria	Actinobacteria	Actinobacteria	Actinomycetales	Actinomycetaceae	Actinomyces	uncultured bacterium; nbw1122a11c1; GQ067098
			Bacteria	Actinobacteria	Actinobacteria	Actinomycetales	Actinomycetaceae	Actinomyces	uncultured bacterium; nbw533c04c1; GQ106168
o1322	o1322 16S-ActOdo	AAAGGTTTGTTCTGGTGGGG	Bacteria	Actinobacteria	Actinobacteria	Actinomycetales	Actinomycetaceae	Actinomyces	Actinomyces sp. oral clone CT068; AF385505
			Bacteria	Actinobacteria	Actinobacteria	Actinomycetales	Actinomycetaceae	Actinomyces	Actinomyces sp. oral clone CT047; AF385504
			Bacteria	Actinobacteria	Actinobacteria	Actinomycetales	Actinomycetaceae	Actinomyces	Actinomyces odontolyticus; CCUG 20536T; AJ234040
			Bacteria	Actinobacteria	Actinobacteria	Actinomycetales	Actinomycetaceae	Actinomyces	Actinomyces odontolyticus; CCUG 28084; AJ234041
			Bacteria	Actinobacteria	Actinobacteria	Actinomycetales	Actinomycetaceae	Actinomyces	uncultured bacterium; nbw881a11c1; GQ030658
			Bacteria	Actinobacteria	Actinobacteria	Actinomycetales	Actinomycetaceae	Actinomyces	uncultured bacterium; nbu545b02c1; GQ045168
			Bacteria	Actinobacteria	Actinobacteria	Actinomycetales	Actinomycetaceae	Actinomyces	uncultured bacterium; nbw1021d10c1; GQ032877
			Bacteria	Actinobacteria	Actinobacteria	Actinomycetales	Actinomycetaceae	Actinomyces	uncultured bacterium; nbw883f04c1; GQ030838
			Bacteria	Actinobacteria	Actinobacteria	Actinomycetales	Actinomycetaceae	Actinomyces	uncultured bacterium; nbw881a06c1; GQ030653
			Bacteria	Actinobacteria	Actinobacteria	Actinomycetales	Actinomycetaceae	Actinomyces	uncultured bacterium; nbw881f11c1; GQ030703
			Bacteria	Actinobacteria	Actinobacteria	Actinomycetales	Actinomycetaceae	Actinomyces	uncultured bacterium; nbw884f05c1; GQ030916
			Bacteria	Actinobacteria	Actinobacteria	Actinomycetales	Actinomycetaceae	Actinomyces	uncultured bacterium; nbw881b01c1; GQ030660
			Bacteria	Actinobacteria	Actinobacteria	Actinomycetales	Actinomycetaceae	Actinomyces	uncultured bacterium; nbw881e05c1; GQ030688
			Bacteria	Actinobacteria	Actinobacteria	Actinomycetales	Actinomycetaceae	Actinomyces	uncultured bacterium; nbw883a01c1; GQ030784
			Bacteria	Actinobacteria	Actinobacteria	Actinomycetales	Actinomycetaceae	Actinomyces	uncultured bacterium; nbw884c03c1; GQ030889
			Bacteria	Actinobacteria	Actinobacteria	Actinomycetales	Actinomycetaceae	Actinomyces	uncultured bacterium; NW06; AY981845
			Bacteria	Actinobacteria	Actinobacteria	Actinomycetales	Actinomycetaceae	Actinomyces	uncultured bacterium; nbw884g09c1; GQ030930
			Bacteria	Actinobacteria	Actinobacteria	Actinomycetales	Actinomycetaceae	Actinomyces	uncultured bacterium; nbw883g01c1; GQ030846
			Bacteria	Actinobacteria	Actinobacteria	Actinomycetales	Actinomycetaceae	Actinomyces	Actinomyces sp.; CCUG 37290; AJ234058
			Bacteria	Actinobacteria	Actinobacteria	Actinomycetales	Actinomycetaceae	Actinomyces	uncultured bacterium; nbw883a02c1; GQ030785
o1323	o1323 16S-ActOral	TAGGTGTGGGGTTCTTTCCA	Bacteria	Actinobacteria	Actinobacteria	Actinomycetales	Actinomycetaceae	Actinobaculum	Actinobaculum sp. oral clone EL030; AY008311
			Bacteria	Actinobacteria	Actinobacteria	Actinomycetales	Thermomonosporaceae	Actinomadura	Actinomadura sp. I43-1; EU181254
			Bacteria	Actinobacteria	Actinobacteria	Actinomycetales	Actinomycetaceae	Actinobaculum	Actinobaculum sp. P2P_19 P1; AY207066
			Bacteria	Actinobacteria	Actinobacteria	Actinomycetales	unclassified_Actinomycetales		uncultured bacterium; nbw1025h02c1; GQ031895
			Bacteria	Actinobacteria	Actinobacteria	Actinomycetales	unclassified_Actinomycetales		uncultured bacterium; nbw740b01c1; GQ010869
			Bacteria	Actinobacteria	Actinobacteria	Actinomycetales	unclassified_Actinomycetales		uncultured bacterium; nbw338c06c1; GQ090790
			Bacteria	Actinobacteria	Actinobacteria	Actinomycetales	unclassified_Actinomycetales		uncultured bacterium; nbu428f05c1; GQ039773
			Bacteria	Actinobacteria	Actinobacteria	unclassified_Actinobacteridae			uncultured bacterium; nbw1088f08c1; GQ053947
			Bacteria	Actinobacteria	Actinobacteria	Actinomycetales	Actinomycetaceae	Actinobaculum	uncultured bacterium; nbw205d07c1; GQ073126
			Bacteria	Actinobacteria	Actinobacteria	Actinomycetales	Actinomycetaceae	Actinobaculum	uncultured bacterium; nbw13h07c1; GQ059872
			Bacteria	Actinobacteria	Actinobacteria	Actinomycetales	Actinomycetaceae	Actinobaculum	uncultured bacterium; nbw161e06c1; GQ074318
			Bacteria	Actinobacteria	Actinobacteria	Actinomycetales	Actinomycetaceae	Actinobaculum	uncultured Actinobaculum sp.; 12B759; FJ976344
			Bacteria	Actinobacteria	Actinobacteria	Actinomycetales	Actinomycetaceae	Actinobaculum	uncultured bacterium; nbw164c10c1; GQ074539
			Bacteria	Actinobacteria	Actinobacteria	Actinomycetales	Actinomycetaceae	Actinobaculum	Actinobaculum sp. BM#101342; AY282578
o1324	o1324 16S-ActOral1	CATGGGTGGGTTTGGAAAGA	Bacteria	Actinobacteria	Actinobacteria	Actinomycetales	Actinomycetaceae	Actinomyces	Actinomyces massiliensis; 4401292; EF558367
			Bacteria	Actinobacteria	Actinobacteria	Actinomycetales	Actinomycetaceae	Actinomyces	uncultured Actinomyces sp.; 10B152; FJ976300
			Bacteria	Actinobacteria	Actinobacteria	Actinomycetales	Actinomycetaceae	Actinomyces	uncultured bacterium; nbu104f08c1; GQ000517
			Bacteria	Actinobacteria	Actinobacteria	Actinomycetales	Actinomycetaceae	Actinomyces	uncultured bacterium; nbw708h11c1; GQ103039
			Bacteria	Actinobacteria	Actinobacteria	Actinomycetales	Actinomycetaceae	Actinomyces	uncultured bacterium; nbw207d05c1; GQ073294
			Bacteria	Actinobacteria	Actinobacteria	Actinomycetales	Actinomycetaceae	Actinomyces	uncultured bacterium; nbw495b03c1; GQ101439
			Bacteria	Actinobacteria	Actinobacteria	Actinomycetales	Actinomycetaceae	Actinomyces	uncultured bacterium; nbw496e09c1; GQ101562
o1325	o1325 16S-ActVis	TGGGAAAGGTTTGTTCCGGT	Bacteria	Actinobacteria	Actinobacteria	Actinomycetales	Actinomycetaceae	Actinomyces	Actinomyces viscosus (T); NCTC 10951; X82453
o1326	o1326 16S-Aerom	GTACAGAGGGCTGCAAGCT	Bacteria	Proteobacteria	Gammaproteobacteria	Aeromonadales	Aeromonadaceae	Aeromonas	unidentified bacterium; W4-B32; AY345498
			Bacteria	Proteobacteria	Gammaproteobacteria	Aeromonadales	Aeromonadaceae	Aeromonas	uncultured gamma proteobacterium Bioluz K33; AF324531
			Bacteria	Proteobacteria	Gammaproteobacteria	Aeromonadales	Aeromonadaceae	Aeromonas	Aeromonas salmonicida subsp. smithia (T); CCM 4103; AJ009859
			Bacteria	Proteobacteria	Gammaproteobacteria	Aeromonadales	Aeromonadaceae	Aeromonas	Aeromonas enteropelogenes (T); type strain: ATCC 49657; X60415
			Bacteria	Proteobacteria	Gammaproteobacteria	Aeromonadales	Aeromonadaceae	Aeromonas	uncultured gamma proteobacterium Bioluz K30; AF324530
			Bacteria	Proteobacteria	Gammaproteobacteria	Aeromonadales	Aeromonadaceae	Aeromonas	Aeromonas bestiarum (T); type strain: CIP 7430; X60406
			Bacteria	Proteobacteria	Gammaproteobacteria	Aeromonadales	Aeromonadaceae	Aeromonas	Aeromonas hydrophila subsp. ranaei (T); type strain: LMG 19707; AJ508766
			Bacteria	Proteobacteria	Gammaproteobacteria	Aeromonadales	Aeromonadaceae	Aeromonas	Aeromonas media (T); ATCC 33907T; X74679
			Bacteria	Proteobacteria	Gammaproteobacteria	Aeromonadales	Aeromonadaceae	Aeromonas	Aeromonas hydrophila subsp. hydrophila (T); ATCC 7966T; X74677
			Bacteria	Proteobacteria	Gammaproteobacteria	Aeromonadales	Aeromonadaceae	Aeromonas	Aeromonas jandaei (T); ATCC 49568T; X74678
			Bacteria	Proteobacteria	Gammaproteobacteria	Aeromonadales	Aeromonadaceae	Aeromonas	Aeromonas schubertii (T); ATCC 43700T; X74682
			Bacteria	Proteobacteria	Gammaproteobacteria	Aeromonadales	Aeromonadaceae	Aeromonas	Aeromonas salmonicida subsp. salmonicida (T); ATCC 27013T; X74680
			Bacteria	Proteobacteria	Gammaproteobacteria	Aeromonadales	Aeromonadaceae	Aeromonas	Aeromonas sobria (T); ATCC 43979T; X74683
			Bacteria	Proteobacteria	Gammaproteobacteria	Aeromonadales	Aeromonadaceae	Aeromonas	Aeromonas eucrenophila (T); X74675
			Bacteria	Proteobacteria	Gammaproteobacteria	Aeromonadales	Aeromonadaceae	Aeromonas	Aeromonas veronii (T); ATCC 35624T; X74684
			Bacteria	Proteobacteria	Gammaproteobacteria	Aeromonadales	Aeromonadaceae	Aeromonas	Aeromonas simiae (T); type strain: IBS S6874; AJ536821
			Bacteria	Proteobacteria	Gammaproteobacteria	Aeromonadales	Aeromonadaceae	Aeromonas	Aeromonas punctata subsp. punctata (T); ATCC 15468T; X74674
			Bacteria	Proteobacteria	Gammaproteobacteria	Aeromonadales	Aeromonadaceae	Aeromonas	Aeromonas popoffii (T); LMG 317541; AJ224308
			Bacteria	Proteobacteria	Gammaproteobacteria	Aeromonadales	Aeromonadaceae	Aeromonas	uncultured gamma proteobacterium; OS-123A; AJ311969
			Bacteria	Proteobacteria	Gammaproteobacteria	Aeromonadales	Aeromonadaceae	Aeromonas	Aeromonas encheleia (T); type strain: LMG 16331 = CECT 4343; AJ458409
o1327	o1327 16S-Alicyc	GAGAGGCAAGGGGAAUUCC	Bacteria	Firmicutes	Bacilli	Bacillales	Alicyclobacillaceae	Alicyclobacillus	Alicyclobacillus sp. DSM 11984; AB059668
			Bacteria	Firmicutes	Bacilli	Bacillales	Alicyclobacillaceae	Alicyclobacillus	Alicyclobacillus sp. MIH 2; AB060162
			Bacteria	Firmicutes	Bacilli	Bacillales	Alicyclobacillaceae	Alicyclobacillus	Alicyclobacillus sp. UZ 1; AB060161
			Bacteria	Firmicutes	Bacilli	Bacillales	Alicyclobacillaceae	Alicyclobacillus	Alicyclobacillus sp. KHC 3; AB060163
			Bacteria	Firmicutes	Bacilli	Bacillales	unclassified_Bacillales		uncultured bacterium; DA113; Y07608
			Bacteria	Firmicutes	Bacilli	Bacillales	Alicyclobacillaceae	Alicyclobacillus	Alicyclobacillus acidocaldarius subsp. acidocaldarius; MIH 321; AB060166
			Bacteria	Firmicutes	Bacilli	Bacillales	Alicyclobacillaceae	Alicyclobacillus	Alicyclobacillus acidocaldarius subsp. acidocaldarius; type strain: DSM 446; AJ496806
			Bacteria	Firmicutes	Bacilli	Bacillales	Alicyclobacillaceae	Alicyclobacillus	Alicyclobacillus sp.; MIH-2; AB004580
			Bacteria	Firmicutes	Bacilli	Bacillales	Alicyclobacillaceae	Alicyclobacillus	Alicyclobacillus sp.; UZ-1; AB004579
			Bacteria	Firmicutes	Bacilli	Bacillales	Alicyclobacillaceae	Alicyclobacillus	Alicyclobacillus cycloheptanicus; SCH; X52489
			Bacteria	Firmicutes	Bacilli	Bacillales	Alicyclobacillaceae	Alicyclobacillus	Alicyclobacillus hesperidum; FR-1B; AJ133632
			Bacteria	Firmicutes	Bacilli	Bacillales	Alicyclobacillaceae	Alicyclobacillus	Alicyclobacillus hesperidum (T); FR-11; AJ133633
			Bacteria	Firmicutes	Bacilli	Bacillales	Alicyclobacillaceae	Alicyclobacillus	Alicyclobacillus sp.; KHA-31; AB004581
			Bacteria	Firmicutes	Bacilli	Bacillales	Alicyclobacillaceae	Alicyclobacillus	Alicyclobacillus sp. FR-6; AJ133635
			Bacteria	Firmicutes	Bacilli	Bacillales	Alicyclobacillaceae	Alicyclobacillus	Alicyclobacillus acidoterrestris; DSM 3922T; AJ133631
			Bacteria	Firmicutes	Bacilli	Bacillales	Alicyclobacillaceae	Alicyclobacillus	Alicyclobacillus sp. FR-3; AJ133634
			Bacteria	Firmicutes	Bacilli	Bacillales	Alicyclobacillaceae	Alicyclobacillus	Alicyclobacillus acidocaldarius subsp. rittmannii; type strain:MR1; AJ493667
			Bacteria	Firmicutes	Bacilli	Bacillales	Alicyclobacillaceae	Alicyclobacillus	Alicyclobacillus cycloheptanicus; X51928
			Bacteria	Firmicutes	Bacilli	Bacillales	Alicyclobacillaceae	Alicyclobacillaceae Incertae Sedis	Bacillus tusciae (T); DSM 2912; Z26933
			Bacteria	Firmicutes	Bacilli	Bacillales	Alicyclobacillaceae	Alicyclobacillus	Alicyclobacillus sendaiensis (T); NTAP-1; AB084128
o1328	o1328 16S-Ali-F	TCTGACCGGTGCAGAGATG	Bacteria	Firmicutes	Bacilli	Bacillales	Alicyclobacillaceae	Alicyclobacillus	Alicyclobacillus acidoterrestris; ATCC 49025T; AB042057
			Bacteria	Firmicutes	Bacilli	Bacillales	Alicyclobacillaceae	Alicyclobacillus	Alicyclobacillus acidoterrestris; DSM 3923; AB042058
			Bacteria	Firmicutes	Bacilli	Bacillales	Alicyclobacillaceae	Alicyclobacillus	Alicyclobacillus acidoterrestris; DSM 2498; AB059675
			Bacteria	Firmicutes	Bacilli	Bacillales	Alicyclobacillaceae	Alicyclobacillus	Alicyclobacillus acidoterrestris; DSM 3924; AB059676

			Bacteria	Firmicutes	Bacilli	Bacillales	Alicyclobacillaceae	Alicyclobacillus	Alicyclobacillus pomorum (T); 3A; AB089840
			Bacteria	Firmicutes	Bacilli	Bacillales	Alicyclobacillaceae	Alicyclobacillus	Alicyclobacillus acidoterrestris; TK25; EF095718
			Bacteria	Firmicutes	Bacilli	Bacillales	Alicyclobacillaceae	Alicyclobacillus	Alicyclobacillus acidocaldarius subsp. acidocaldarius; DSM 451; AB059672
			Bacteria	Firmicutes	Bacilli	Bacillales	Alicyclobacillaceae	Alicyclobacillus	Alicyclobacillus acidocaldarius subsp. acidocaldarius; DSM 452; AB059673
			Bacteria	Firmicutes	Bacilli	Bacillales	Alicyclobacillaceae	Alicyclobacillus	Alicyclobacillus acidocaldarius subsp. acidocaldarius (T); ATCC 27009; AB042056
			Bacteria	Firmicutes	Bacilli	Bacillales	Alicyclobacillaceae	Alicyclobacillus	Alicyclobacillus acidocaldarius subsp. acidocaldarius; type strain: DSM 446; AJ496806
			Bacteria	Firmicutes	Bacilli	Bacillales	Alicyclobacillaceae	Alicyclobacillus	Alicyclobacillus acidoterrestris; ATCC 49025; AY573797
			Bacteria	Firmicutes	Bacilli	Bacillales	Alicyclobacillaceae	Alicyclobacillus	Alicyclobacillus acidocaldarius subsp. acidocaldarius; DSM 448; AB059670
			Bacteria	Firmicutes	Bacilli	Bacillales	Alicyclobacillaceae	Alicyclobacillus	Alicyclobacillus acidocaldarius subsp. acidocaldarius; DSM 449; AB059671
			Bacteria	Firmicutes	Bacilli	Bacillales	Alicyclobacillaceae	Alicyclobacillus	Alicyclobacillus hesperidum; FR-1B; AJ133632
			Bacteria	Firmicutes	Bacilli	Bacillales	Alicyclobacillaceae	Alicyclobacillus	Alicyclobacillus hesperidum (T); FR-11; AJ133633
			Bacteria	Firmicutes	Bacilli	Bacillales	Alicyclobacillaceae	Alicyclobacillus	Alicyclobacillus acidoterrestris; DSM 3922T; AJ133631
			Bacteria	Firmicutes	Bacilli	Bacillales	Alicyclobacillaceae	Alicyclobacillus	Alicyclobacillus sp. T131; AM749784
			Bacteria	Firmicutes	Bacilli	Bacillales	Alicyclobacillaceae	Alicyclobacillus	Alicyclobacillus sp. P244; AM749776
			Bacteria	Firmicutes	Bacilli	Bacillales	Alicyclobacillaceae	Alicyclobacillus	Alicyclobacillus acidoterrestris; CBMAI 0495; AY686617
			Bacteria	Firmicutes	Bacilli	Bacillales	Alicyclobacillaceae	Alicyclobacillus	Alicyclobacillus acidoterrestris; CBMAI 0497; AY686619
o1329	o1329 16S-Arcob	TCAGAAGGAATACCGATTGC	Bacteria	Proteobacteria	Epsilonproteobacteria	Campylobacterales	Campylobacteraceae	Arcobacter	unidentified bacterium; T24; Z93999
			Bacteria	Proteobacteria	Epsilonproteobacteria	Campylobacterales	Campylobacteraceae	Arcobacter	unidentified bacterium; T31; Z93951
			Bacteria	Proteobacteria	Epsilonproteobacteria	Campylobacterales	Campylobacteraceae	Arcobacter	unidentified bacterium; T31; Z94002
			Bacteria	Proteobacteria	Epsilonproteobacteria	Campylobacterales	Campylobacteraceae	Arcobacter	uncultured epsilon proteobacterium KTc1160; AF235116
			Bacteria	Proteobacteria	Epsilonproteobacteria	Campylobacterales	Campylobacteraceae	Arcobacter	unidentified bacterium; T55; Z94001
			Bacteria	Proteobacteria	Epsilonproteobacteria	Campylobacterales	Campylobacteraceae	Arcobacter	Arcobacter sp. D1a1; AJ271654
			Bacteria	Proteobacteria	Epsilonproteobacteria	Campylobacterales	Campylobacteraceae	Arcobacter	Arcobacter sp. B4b1; AJ271653
			Bacteria	Proteobacteria	Epsilonproteobacteria	Campylobacterales	Campylobacteraceae	Arcobacter	Arcobacter sp. A3b2; AJ271655
			Bacteria	Proteobacteria	Epsilonproteobacteria	Campylobacterales	Campylobacteraceae	Arcobacter	uncultured bacterium; sacC13A7; AY548997
			Bacteria	Proteobacteria	Epsilonproteobacteria	Campylobacterales	Campylobacteraceae	Arcobacter	uncultured bacterium; sacC3E4; AY548986
			Bacteria	Proteobacteria	Epsilonproteobacteria	Campylobacterales	Campylobacteraceae	Arcobacter	uncultured eubacterium CHA3-127; AJ132726
			Bacteria	Proteobacteria	Epsilonproteobacteria	Campylobacterales	Campylobacteraceae	Arcobacter	uncultured eubacterium CHA3-124; AJ132724
			Bacteria	Proteobacteria	Epsilonproteobacteria	Campylobacterales	Campylobacteraceae	Arcobacter	uncultured eubacterium CHA3-437; AJ132728
			Bacteria	Proteobacteria	Epsilonproteobacteria	Campylobacterales	Campylobacteraceae	Arcobacter	uncultured eubacterium CHA3-101; AJ132723
			Bacteria	Proteobacteria	Epsilonproteobacteria	Campylobacterales	Campylobacteraceae	Arcobacter	uncultured eubacterium CHA3-30; AJ132732
			Bacteria	Proteobacteria	Epsilonproteobacteria	Campylobacterales	Campylobacteraceae	Arcobacter	uncultured eubacterium CHA3-217; AJ132725
			Bacteria	Proteobacteria	Epsilonproteobacteria	Campylobacterales	Campylobacteraceae	Arcobacter	uncultured eubacterium CHA3-215; AJ132729
			Bacteria	Proteobacteria	Epsilonproteobacteria	Campylobacterales	Campylobacteraceae	Arcobacter	uncultured eubacterium CHA3-4; AJ132730
			Bacteria	Proteobacteria	Epsilonproteobacteria	Campylobacterales	Campylobacteraceae	Arcobacter	uncultured eubacterium CHA3-102; AJ132731
			Bacteria	Proteobacteria	Epsilonproteobacteria	Campylobacterales	Campylobacteraceae	Arcobacter	uncultured eubacterium CHA3-66; AJ132727
o1331	o1331 16S-AtoGroup	AATGATGGGTAGCTGGGTTG	Bacteria	Bacteroidetes	unclassified_Bacteroidetes				uncultured Bacteroidetes bacterium; MBAE28; AJ567587
			Bacteria	Actinobacteria	Actinobacteria	Coriobacteriales	Coriobacteriaceae	Atopobium	uncultured bacterium; P2D15-510; EF510762
			Bacteria	Actinobacteria	Actinobacteria	Coriobacteriales	Coriobacteriaceae	Atopobium	uncultured bacterium; P2D15-495; EF510777
			Bacteria	Actinobacteria	Actinobacteria	Coriobacteriales	Coriobacteriaceae	Atopobium	Atopobium rimae (T); ATCC49626; AF292371
			Bacteria	Actinobacteria	Actinobacteria	Coriobacteriales	Coriobacteriaceae	Atopobium	Atopobium sp. DMCT15023; EU186380
			Bacteria	Actinobacteria	Actinobacteria	Coriobacteriales	Coriobacteriaceae	Atopobium	Atopobium sp. oral clone C019; AF287760
			Bacteria	Actinobacteria	Actinobacteria	Coriobacteriales	Coriobacteriaceae	Atopobium	Atopobium rimae; EU828366
			Bacteria	Actinobacteria	Actinobacteria	Coriobacteriales	Coriobacteriaceae	Atopobium	Atopobium parvulum; ATCC22793; AF292372
			Bacteria	Actinobacteria	Actinobacteria	Coriobacteriales	Coriobacteriaceae	Atopobium	uncultured bacterium; P4D1-741; EF509772
			Bacteria	Actinobacteria	Actinobacteria	Coriobacteriales	Coriobacteriaceae	Atopobium	Atopobium parvulum; S44206
			Bacteria	Actinobacteria	Actinobacteria	Coriobacteriales	Coriobacteriaceae	Atopobium	Atopobium rimae; S44205
			Bacteria	Actinobacteria	Actinobacteria	Coriobacteriales	Coriobacteriaceae	Atopobium	Atopobium rimae; X67149
			Bacteria	Actinobacteria	Actinobacteria	Coriobacteriales	Coriobacteriaceae	Atopobium	Atopobium parvulum (T); X67150
			Bacteria	Actinobacteria	Actinobacteria	Coriobacteriales	Coriobacteriaceae	Atopobium	uncultured bacterium; nbw777a06c1; GQ009475
			Bacteria	Actinobacteria	Actinobacteria	Coriobacteriales	Coriobacteriaceae	Atopobium	uncultured bacterium; nbw828b02c1; GQ009170
			Bacteria	Actinobacteria	Actinobacteria	Coriobacteriales	Coriobacteriaceae	Atopobium	uncultured bacterium; nbw779g08c1; GQ009818
			Bacteria	Actinobacteria	Actinobacteria	Coriobacteriales	Coriobacteriaceae	Atopobium	uncultured bacterium; nbw784a11c1; GQ010207
			Bacteria	Actinobacteria	Actinobacteria	Coriobacteriales	Coriobacteriaceae	Atopobium	uncultured bacterium; nbw777e09c1; GQ009519
			Bacteria	Actinobacteria	Actinobacteria	Coriobacteriales	Coriobacteriaceae	Atopobium	uncultured bacterium; nbw784b03c1; GQ010209
			Bacteria	Actinobacteria	Actinobacteria	Coriobacteriales	Coriobacteriaceae	Atopobium	uncultured Atopobium sp.; T15FO04; DQ093271
o1332	o1332 16S-AtoPar	CGCATGGAAGACTCGAGAAA	Bacteria	Actinobacteria	Actinobacteria	Coriobacteriales	Coriobacteriaceae	Atopobium	uncultured bacterium; P2D15-510; EF510762
			Bacteria	Actinobacteria	Actinobacteria	Coriobacteriales	Coriobacteriaceae	Atopobium	uncultured bacterium; P2D15-495; EF510777
			Bacteria	Actinobacteria	Actinobacteria	Coriobacteriales	Coriobacteriaceae	Atopobium	Atopobium parvulum; ATCC22793; AF292372
			Bacteria	Actinobacteria	Actinobacteria	Coriobacteriales	Coriobacteriaceae	Atopobium	Atopobium parvulum; S44206
			Bacteria	Actinobacteria	Actinobacteria	Coriobacteriales	Coriobacteriaceae	Atopobium	Atopobium parvulum (T); X67150
			Bacteria	Actinobacteria	Actinobacteria	Coriobacteriales	Coriobacteriaceae	Atopobium	uncultured bacterium; nbw777a06c1; GQ009475
			Bacteria	Actinobacteria	Actinobacteria	Coriobacteriales	Coriobacteriaceae	Atopobium	uncultured bacterium; nbw894c02c1; GQ031759
			Bacteria	Actinobacteria	Actinobacteria	Coriobacteriales	Coriobacteriaceae	Atopobium	uncultured bacterium; nbw903c10c1; GQ032167
			Bacteria	Actinobacteria	Actinobacteria	Coriobacteriales	Coriobacteriaceae	Atopobium	uncultured bacterium; nbw904d02c1; GQ032255
			Bacteria	Actinobacteria	Actinobacteria	Coriobacteriales	Coriobacteriaceae	Atopobium	uncultured bacterium; nbw828b02c1; GQ009170
			Bacteria	Actinobacteria	Actinobacteria	Coriobacteriales	Coriobacteriaceae	Atopobium	uncultured bacterium; nbw779g08c1; GQ009818
			Bacteria	Actinobacteria	Actinobacteria	Coriobacteriales	Coriobacteriaceae	Atopobium	uncultured bacterium; nbw895b06c1; GQ026670
			Bacteria	Actinobacteria	Actinobacteria	Coriobacteriales	Coriobacteriaceae	Atopobium	uncultured bacterium; nbw903h01c1; GQ032214
			Bacteria	Actinobacteria	Actinobacteria	Coriobacteriales	Coriobacteriaceae	Atopobium	uncultured bacterium; nbw903h08c1; GQ032221
			Bacteria	Actinobacteria	Actinobacteria	Coriobacteriales	Coriobacteriaceae	Atopobium	uncultured bacterium; nbw789e05c1; GQ010975
			Bacteria	Actinobacteria	Actinobacteria	Coriobacteriales	Coriobacteriaceae	Atopobium	uncultured bacterium; nbw788c09c1; GQ010611
			Bacteria	Actinobacteria	Actinobacteria	Coriobacteriales	Coriobacteriaceae	Atopobium	uncultured bacterium; nbw790b08c1; GQ011029
			Bacteria	Actinobacteria	Actinobacteria	Coriobacteriales	Coriobacteriaceae	Atopobium	uncultured bacterium; nbw904d01c1; GQ032254
			Bacteria	Actinobacteria	Actinobacteria	Coriobacteriales	Coriobacteriaceae	Atopobium	uncultured bacterium; nbw777e09c1; GQ009519
			Bacteria	Actinobacteria	Actinobacteria	Coriobacteriales	Coriobacteriaceae	Atopobium	uncultured bacterium; nbw894h08c1; GQ031815
o1333	o1333 16S-BacGroup1	TGAATACTCGCTGTTTGCGA	Bacteria	Bacteroidetes	Bacteroidetes	Bacteroidales	Bacteroidaceae	Bacteroides	Bacteroides coprosuis (T); PC139; AF319778
			Bacteria	Bacteroidetes	Bacteroidetes	Bacteroidales	Bacteroidaceae	Bacteroides	Bacteroides acidifaciens; A24; AB021159
			Bacteria	Bacteroidetes	Bacteroidetes	Bacteroidales	Bacteroidaceae	Bacteroides	Bacteroides acidifaciens; AB021157
			Bacteria	Bacteroidetes	Bacteroidetes	Bacteroidales	Bacteroidaceae	Bacteroides	Bacteroides acidifaciens; A29; AB021160
			Bacteria	Bacteroidetes	Bacteroidetes	Bacteroidales	Bacteroidaceae	Bacteroides	Bacteroides acidifaciens; A31; AB021161
			Bacteria	Bacteroidetes	Bacteroidetes	Bacteroidales	Bacteroidaceae	Bacteroides	uncultured bacterium; from human colonic sample; HuCA36; AJ408983
			Bacteria	Bacteroidetes	Bacteroidetes	Bacteroidales	Bacteroidaceae	Bacteroides	Bacteroides acidifaciens; A1; AB021158
			Bacteria	Bacteroidetes	Bacteroidetes	Bacteroidales	Bacteroidaceae	Bacteroides	Bacteroides acidifaciens; A43; AB021165
			Bacteria	Bacteroidetes	Bacteroidetes	Bacteroidales	Bacteroidaceae	Bacteroides	uncultured bacterium; from human colonic sample; HuCB3; AJ408988
			Bacteria	Bacteroidetes	Bacteroidetes	Bacteroidales	Bacteroidaceae	Bacteroides	uncultured bacterium; from human colonic sample; HuCA9; AJ408965
			Bacteria	Bacteroidetes	Bacteroidetes	Bacteroidales	Bacteroidaceae	Bacteroides	uncultured bacterium; from human colonic sample; HuCA21; AJ409009
			Bacteria	Bacteroidetes	Bacteroidetes	Bacteroidales	Bacteroidaceae	Bacteroides	Bacteroides fragilis; Bfr189; 10; X83944

			Bacteria	Bacteroidetes	Bacteroidetes	Bacteroidales	Bacteroidaceae	Bacteroides	Bacteroides fragilis; B119; @8; X83942
			Bacteria	Bacteroidetes	Bacteroidetes	Bacteroidales	Bacteroidaceae	Bacteroides	Bacteroides fragilis; Bfr901; 11; X83945
			Bacteria	Bacteroidetes	Bacteroidetes	Bacteroidales	Bacteroidaceae	Bacteroides	Bacteroides fragilis; 790; @3; X83937
			Bacteria	Bacteroidetes	Bacteroidetes	Bacteroidales	Bacteroidaceae	Bacteroides	Bacteroides fragilis; AIP 638; @2; X83936
			Bacteria	Bacteroidetes	Bacteroidetes	Bacteroidales	Bacteroidaceae	Bacteroides	Bacteroides fragilis; Bfr920; 12; X83946
			Bacteria	Bacteroidetes	Bacteroidetes	Bacteroidales	Bacteroidaceae	Bacteroides	Bacteroides uniformis; JCM 5828T; AB050110
			Bacteria	Bacteroidetes	Bacteroidetes	Bacteroidales	Bacteroidaceae	Bacteroides	Bacteroides fragilis; 2393; 14; X83948
			Bacteria	Bacteroidetes	Bacteroidetes	Bacteroidales	Bacteroidaceae	Bacteroides	Bacteroides fragilis; 4225; 16; X83950
o1336	o1336 16S-BacSpe	GCGGAATTCGTGGTGTAGC	Bacteria	Bacteroidetes	Bacteroidetes	Bacteroidales	Bacteroidaceae	Bacteroides	Bacteroides acidifaciens; A24; AB021159
			Bacteria	Bacteroidetes	Bacteroidetes	Bacteroidales	Bacteroidaceae	Bacteroides	Bacteroides acidifaciens; AB021157
			Bacteria	Bacteroidetes	Bacteroidetes	Bacteroidales	Bacteroidaceae	Bacteroides	Bacteroides acidifaciens; A29; AB021160
			Bacteria	Bacteroidetes	Bacteroidetes	Bacteroidales	Bacteroidaceae	Bacteroides	Bacteroides acidifaciens; A31; AB021161
			Bacteria	Bacteroidetes	Bacteroidetes	Bacteroidales	Bacteroidaceae	Bacteroides	uncultured bacterium; from human colonic sample; HuCA36; AJ408983
			Bacteria	Bacteroidetes	Bacteroidetes	Bacteroidales	Bacteroidaceae	Bacteroides	Bacteroides acidifaciens; A1; AB021158
			Bacteria	Bacteroidetes	Bacteroidetes	Bacteroidales	Bacteroidaceae	Bacteroides	Bacteroides acidifaciens; A43; AB021165
			Bacteria	Bacteroidetes	Bacteroidetes	Bacteroidales	Bacteroidaceae	Bacteroides	uncultured bacterium; from human colonic sample; HuCB3; AJ408988
			Bacteria	Bacteroidetes	Bacteroidetes	Bacteroidales	Prevotellaceae	Prevotella	Prevotella bryantii (T); B14 (DSM 11371, species type strain); AJ006457
			Bacteria	Bacteroidetes	Bacteroidetes	Bacteroidales	Bacteroidaceae	Bacteroides	uncultured bacterium; from human colonic sample; HuCA9; AJ408965
			Bacteria	Bacteroidetes	Bacteroidetes	Bacteroidales	Bacteroidaceae	Bacteroides	uncultured bacterium; from human colonic sample; HuCA21; AJ409009
			Bacteria	Bacteroidetes	Bacteroidetes	Bacteroidales	Bacteroidaceae	Bacteroides	Bacteroides fragilis; Bfr189; 10; X83944
			Bacteria	Bacteroidetes	Bacteroidetes	Bacteroidales	Bacteroidaceae	Bacteroides	Bacteroides fragilis; B119; @8; X83942
			Bacteria	Bacteroidetes	Bacteroidetes	Bacteroidales	Bacteroidaceae	Bacteroides	Bacteroides fragilis; Bfr901; 11; X83945
			Bacteria	Bacteroidetes	Bacteroidetes	Bacteroidales	Bacteroidaceae	Bacteroides	Bacteroides fragilis; 790; @3; X83937
			Bacteria	Bacteroidetes	Bacteroidetes	Bacteroidales	Bacteroidaceae	Bacteroides	Bacteroides eggerthii; DSM 20697T; AB050107
			Bacteria	Bacteroidetes	Bacteroidetes	Bacteroidales	Bacteroidaceae	Bacteroides	Bacteroides fragilis; Bfr920; 12; X83946
			Bacteria	Bacteroidetes	Bacteroidetes	Bacteroidales	Bacteroidaceae	Bacteroides	Bacteroides uniformis; JCM 5828T; AB050110
			Bacteria	Bacteroidetes	Bacteroidetes	Bacteroidales	Bacteroidaceae	Bacteroides	Bacteroides fragilis; 2393; 14; X83948
			Bacteria	Bacteroidetes	Bacteroidetes	Bacteroidales	Bacteroidaceae	Bacteroides	Bacteroides fragilis; 4225; 16; X83950
o1337	o1337 16S-Bactdl	TGCCGTGAGGTGTCGGCT	Bacteria	Bacteroidetes	Bacteroidetes	Bacteroidales	Bacteroidaceae	Bacteroides	Bacteroides coprosuis (T); PC139; AF319778
			Bacteria	Bacteroidetes	Bacteroidetes	Bacteroidales	Bacteroidaceae	Bacteroides	Bacteroides acidifaciens; A24; AB021159
			Bacteria	Bacteroidetes	Bacteroidetes	Bacteroidales	Bacteroidaceae	Bacteroides	Bacteroides acidifaciens; AB021157
			Bacteria	Bacteroidetes	Bacteroidetes	Bacteroidales	Bacteroidaceae	Bacteroides	Bacteroides acidifaciens; A29; AB021160
			Bacteria	Bacteroidetes	Bacteroidetes	Bacteroidales	Bacteroidaceae	Bacteroides	Bacteroides acidifaciens; A31; AB021161
			Bacteria	Bacteroidetes	Bacteroidetes	Bacteroidales	Bacteroidaceae	Bacteroides	uncultured bacterium; from human colonic sample; HuCA36; AJ408983
			Bacteria	Bacteroidetes	Bacteroidetes	Bacteroidales	Bacteroidaceae	Bacteroides	Bacteroides acidifaciens; A1; AB021158
			Bacteria	Bacteroidetes	Bacteroidetes	Bacteroidales	Bacteroidaceae	Bacteroides	Bacteroides acidifaciens; A43; AB021165
			Bacteria	Bacteroidetes	Bacteroidetes	Bacteroidales	Bacteroidaceae	Bacteroides	uncultured bacterium; from human colonic sample; HuCB3; AJ408988
			Bacteria	Bacteroidetes	Bacteroidetes	Bacteroidales	Bacteroidaceae	Bacteroides	uncultured bacterium; from human colonic sample; HuCA9; AJ408965
			Bacteria	Bacteroidetes	Bacteroidetes	Bacteroidales	Bacteroidaceae	Bacteroides	Bacteroides fragilis; Bfr189; 10; X83944
			Bacteria	Bacteroidetes	Bacteroidetes	Bacteroidales	Bacteroidaceae	Bacteroides	Bacteroides fragilis; B119; @8; X83942
			Bacteria	Bacteroidetes	Bacteroidetes	Bacteroidales	Bacteroidaceae	Bacteroides	Bacteroides fragilis; Bfr901; 11; X83945
			Bacteria	Bacteroidetes	Bacteroidetes	Bacteroidales	Bacteroidaceae	Bacteroides	Bacteroides fragilis; 790; @3; X83937
			Bacteria	Bacteroidetes	Bacteroidetes	Bacteroidales	Bacteroidaceae	Bacteroides	Bacteroides eggerthii; DSM 20697T; AB050107
			Bacteria	Bacteroidetes	Bacteroidetes	Bacteroidales	Bacteroidaceae	Bacteroides	Bacteroides fragilis; AIP 638; @2; X83936
			Bacteria	Bacteroidetes	Bacteroidetes	Bacteroidales	Bacteroidaceae	Bacteroides	Bacteroides fragilis; Bfr920; 12; X83946
			Bacteria	Bacteroidetes	Bacteroidetes	Bacteroidales	Bacteroidaceae	Bacteroides	Bacteroides uniformis; JCM 5828T; AB050110
			Bacteria	Bacteroidetes	Bacteroidetes	Bacteroidales	Bacteroidaceae	Bacteroides	Bacteroides fragilis; 2393; 14; X83948
			Bacteria	Bacteroidetes	Bacteroidetes	Bacteroidales	Bacteroidaceae	Bacteroides	Bacteroides fragilis; 4225; 16; X83950
o1339	o1339 16S-BerOral	AAAAGCTACGGCGGATAAGG	Bacteria	Bacteroidetes	Flavobacteria	Flavobacteriales	Flavobacteriaceae	unclassified_Flavobacteriaceae	uncultured Bergeyella sp.; 602D02(oral); AM420234
			Bacteria	Bacteroidetes	Flavobacteria	Flavobacteriales	Flavobacteriaceae	unclassified_Flavobacteriaceae	uncultured Bergeyella sp.; 450a; EU644465
			Bacteria	Bacteroidetes	Flavobacteria	Flavobacteriales	Flavobacteriaceae	unclassified_Flavobacteriaceae	Bergeyella sp. oral clone AK152; AY008691
			Bacteria	Bacteroidetes	Flavobacteria	Flavobacteriales	Flavobacteriaceae	unclassified_Flavobacteriaceae	uncultured bacterium; nbw491b01c1; GQ101136
			Bacteria	Bacteroidetes	Flavobacteria	Flavobacteriales	Flavobacteriaceae	unclassified_Flavobacteriaceae	uncultured bacterium; nbw492d08c1; GQ101217
			Bacteria	Bacteroidetes	Flavobacteria	Flavobacteriales	Flavobacteriaceae	unclassified_Flavobacteriaceae	uncultured bacterium; nbw205f11c1; GQ073152
			Bacteria	Bacteroidetes	Flavobacteria	Flavobacteriales	Flavobacteriaceae	unclassified_Flavobacteriaceae	uncultured bacterium; nbw164a01c1; GQ074513
			Bacteria	Bacteroidetes	Flavobacteria	Flavobacteriales	Flavobacteriaceae	unclassified_Flavobacteriaceae	uncultured bacterium; nbw491g09c1; GQ101166
			Bacteria	Bacteroidetes	Flavobacteria	Flavobacteriales	Flavobacteriaceae	unclassified_Flavobacteriaceae	uncultured Bergeyella sp.; 12B448; FJ976338
			Bacteria	Bacteroidetes	Flavobacteria	Flavobacteriales	Flavobacteriaceae	unclassified_Flavobacteriaceae	uncultured bacterium; nbw791g04c1; GQ011168
			Bacteria	Bacteroidetes	Flavobacteria	Flavobacteriales	Flavobacteriaceae	unclassified_Flavobacteriaceae	uncultured bacterium; nbu189b03c1; GQ019358
			Bacteria	Bacteroidetes	Flavobacteria	Flavobacteriales	Flavobacteriaceae	unclassified_Flavobacteriaceae	uncultured bacterium; nbu190f12c1; GQ019501
o1340	o1340 16S-Betap	AGTGAAATGCGTAGAGATGT	Bacteria	Proteobacteria	Betaproteobacteria	Burkholderiales	Burkholderiaceae	Burkholderia	Burkholderia graminis (T); C4D1M (type strain); U96939
			Bacteria	Proteobacteria	Betaproteobacteria	Burkholderiales	Burkholderiaceae	Burkholderia	Burkholderia plantarii; LMG9035T; U96933
			Bacteria	Proteobacteria	Betaproteobacteria	Burkholderiales	Burkholderiaceae	Burkholderia	Burkholderia phenazinium (T); LMG2247T; U96936
			Bacteria	Proteobacteria	Betaproteobacteria	Burkholderiales	Burkholderiaceae	Burkholderia	Burkholderia anthina; R-4183; AJ420880
			Bacteria	Proteobacteria	Betaproteobacteria	Burkholderiales	Burkholderiaceae	Burkholderia	Burkholderia gladioli; NCIMB 9898T; AB021384
			Bacteria	Proteobacteria	Betaproteobacteria	Burkholderiales	Burkholderiaceae	Burkholderia	Burkholderia sp. EN-B9; AF074712
			Bacteria	Proteobacteria	Betaproteobacteria	Burkholderiales	Burkholderiaceae	Burkholderia	Burkholderia sp. C3B1M; U96938
			Bacteria	Firmicutes	Clostridia	Clostridiales	Veillonellaceae	Sporomusa	Sporomusa sp. DR6; Y17760
			Bacteria	Proteobacteria	Betaproteobacteria	Burkholderiales	Burkholderiaceae	Burkholderia	Burkholderia sp. S-2; AB079372
			Bacteria	Proteobacteria	Betaproteobacteria	Burkholderiales	Burkholderiaceae	Burkholderia	Burkholderia sp. JR7B3; AF439777
			Bacteria	Proteobacteria	Betaproteobacteria	Burkholderiales	Burkholderiaceae	Burkholderia	Burkholderia glathei; ATCC 29195T; AB021374
			Bacteria	Proteobacteria	Betaproteobacteria	Burkholderiales	Burkholderiaceae	Burkholderia	Burkholderia caryophylli; ATCC 25418T; AB021423
			Bacteria	Proteobacteria	Betaproteobacteria	Burkholderiales	Burkholderiaceae	Burkholderia	Burkholderia sp. AB119; AF364859
			Bacteria	Proteobacteria	Betaproteobacteria	Burkholderiales	Burkholderiaceae	Burkholderia	Burkholderia glathei (T); ATCC 29195T; LMG 14190T; Y17052
			Bacteria	Proteobacteria	Betaproteobacteria	Burkholderiales	Burkholderiaceae	Burkholderia	Burkholderia sp. TH2; AB041772
			Bacteria	Proteobacteria	Betaproteobacteria	Burkholderiales	Burkholderiaceae	Burkholderia	Burkholderia gladioli (T); ATCC 10248, pathovar gladioli; X67038
			Bacteria	Proteobacteria	Betaproteobacteria	Burkholderiales	Burkholderiaceae	Burkholderia	Burkholderia caryophylli (T); ATCC 25418; X67039
			Bacteria	Firmicutes	Clostridia	Clostridiales	Veillonellaceae	Sporomusa	Sporomusa sp. DR15; Y17762
			Bacteria	Proteobacteria	Betaproteobacteria	Burkholderiales	Burkholderiaceae	Burkholderia	Burkholderia cepacia; X80287
			Bacteria	Proteobacteria	Betaproteobacteria	Burkholderiales	Burkholderiaceae	Burkholderia	Burkholderia cepacia; X80286
o1341	o1341 16S-Bifbac	TGGAATGTGTAGATATCGGG	Bacteria	Actinobacteria	Actinobacteria	Bifidobacteriales	Bifidobacteriaceae	Bifidobacterium	Bifidobacterium animalis subsp. lactis; YIT 4121; AB050136
			Bacteria	Actinobacteria	Actinobacteria	Bifidobacteriales	Bifidobacteriaceae	Bifidobacterium	Bifidobacterium animalis subsp. animalis; YIT 4119; AB050137
			Bacteria	Actinobacteria	Actinobacteria	Bifidobacteriales	Bifidobacteriaceae	Bifidobacterium	Bifidobacterium animalis subsp. animalis (T); DSM 10140; UR1; X89513
			Bacteria	Actinobacteria	Actinobacteria	Bifidobacteriales	Bifidobacteriaceae	Bifidobacterium	Bifidobacterium sp.; ATCC 15697; X89111
			Bacteria	Actinobacteria	Actinobacteria	Bifidobacteriales	Bifidobacteriaceae	Bifidobacterium	Bifidobacterium animalis subsp. animalis; YIT 4090; AB050138
			Bacteria	Actinobacteria	Actinobacteria	Bifidobacteriales	Bifidobacteriaceae	Bifidobacterium	Bifidobacterium thermophilum; AB016246
			Bacteria	Actinobacteria	Actinobacteria	Bifidobacteriales	Bifidobacteriaceae	Bifidobacterium	Bifidobacterium longum biovar Longum; Y10; AJ311606
			Bacteria	Actinobacteria	Actinobacteria	Bifidobacteriales	Bifidobacteriaceae	Bifidobacterium	Bifidobacterium breve; JCM 7019; AF491836

			Bacteria	Actinobacteria	Actinobacteria	Bifidobacteriales	Bifidobacteriaceae	Bifidobacterium	Bifidobacterium breve; Y8; AJ311605
			Bacteria	Actinobacteria	Actinobacteria	Bifidobacteriales	Bifidobacteriaceae	Bifidobacterium	Bifidobacterium breve; JCM 1273; AF491832
			Bacteria	Actinobacteria	Actinobacteria	Bifidobacteriales	Bifidobacteriaceae	Bifidobacterium	Bifidobacterium breve; JCM 7016; AF491834
			Bacteria	Actinobacteria	Actinobacteria	Bifidobacteriales	Bifidobacteriaceae	Bifidobacterium	Bifidobacterium breve; JCM 7017; AF491835
			Bacteria	Actinobacteria	Actinobacteria	Bifidobacteriales	Bifidobacteriaceae	Bifidobacterium	Bifidobacterium breve; JCM 7020; AF491833
			Bacteria	Actinobacteria	Actinobacteria	Bifidobacteriales	Bifidobacteriaceae	Bifidobacterium	Bifidobacterium breve; KB 94; AY513713
			Bacteria	Actinobacteria	Actinobacteria	Bifidobacteriales	Bifidobacteriaceae	Bifidobacterium	Bifidobacterium breve; KB 92; AY513712
			Bacteria	Actinobacteria	Actinobacteria	Bifidobacteriales	Bifidobacteriaceae	Bifidobacterium	Bifidobacterium scardovii (T); CCUG 13008; AJ307005
			Bacteria	Actinobacteria	Actinobacteria	Bifidobacteriales	Bifidobacteriaceae	Bifidobacterium	Bifidobacterium simiae; CCUG 30289T; AJ420965
			Bacteria	Actinobacteria	Actinobacteria	Bifidobacteriales	Bifidobacteriaceae	Bifidobacterium	Bifidobacterium pseudocatenulatum; F34; AF333390
			Bacteria	Chlorobi	Chlorobia	Chlorobiales	Chlorobiaceae	Chlorobium	Chlorobium phaeobacteroides (T); 2430; Y08104
			Bacteria	Actinobacteria	Actinobacteria	Bifidobacteriales	Bifidobacteriaceae	Bifidobacterium	Bifidobacterium animalis subsp. animalis (T); ATCC 25527; X70971
o1342	o1342 16S-BifDen1	ATGCATGTCCTTCCGGGAAA	Bacteria	Actinobacteria	Actinobacteria	Bifidobacteriales	Bifidobacteriaceae	Bifidobacterium	Bifidobacterium dentium (T); ATCC 27534; D86183
			Bacteria	Actinobacteria	Actinobacteria	Bifidobacteriales	Bifidobacteriaceae	Bifidobacterium	Bifidobacterium sp. oral strain A32ED; AF287759
			Bacteria	Actinobacteria	Actinobacteria	Bifidobacteriales	Bifidobacteriaceae	Bifidobacterium	uncultured bacterium (human infant) N55G; AF253364
o1343	o1343 16S-BifDen2	ATGCATGTCCTTCCGGGAAA	Bacteria	Actinobacteria	Actinobacteria	Bifidobacteriales	Bifidobacteriaceae	Bifidobacterium	Bifidobacterium dentium (T); ATCC 27534; D86183
			Bacteria	Actinobacteria	Actinobacteria	Bifidobacteriales	Bifidobacteriaceae	Bifidobacterium	Bifidobacterium sp. oral strain A32ED; AF287759
			Bacteria	Actinobacteria	Actinobacteria	Bifidobacteriales	Bifidobacteriaceae	Bifidobacterium	uncultured bacterium (human infant) N55G; AF253364
o1344	o1344 16S-BifOral	AAACCGCTTTTATAGGGGGG	Bacteria	Actinobacteria	Actinobacteria	Bifidobacteriales	Bifidobacteriaceae	unclassified_Bifidobacteriaceae	Bifidobacterium sp. oral clone CX010; AF287758
			Bacteria	Actinobacteria	Actinobacteria	Bifidobacteriales	Bifidobacteriaceae	unclassified_Bifidobacteriaceae	uncultured bacterium; rRNA129; AY958902
			Bacteria	Actinobacteria	Actinobacteria	Bifidobacteriales	Bifidobacteriaceae	unclassified_Bifidobacteriaceae	Scardovia sp. 29D03; GQ892826
			Bacteria	Actinobacteria	Actinobacteria	Bifidobacteriales	Bifidobacteriaceae	unclassified_Bifidobacteriaceae	Scardovia genomosp. C1; C1A_55; AY278626
			Bacteria	Actinobacteria	Actinobacteria	Bifidobacteriales	Bifidobacteriaceae	unclassified_Bifidobacteriaceae	Scardovia sp. 04C01; GQ892825
			Bacteria	Actinobacteria	Actinobacteria	Bifidobacteriales	Bifidobacteriaceae	unclassified_Bifidobacteriaceae	Scardovia sp. 2008322; GQ914993
			Bacteria	Actinobacteria	Actinobacteria	Bifidobacteriales	Bifidobacteriaceae	unclassified_Bifidobacteriaceae	Scardovia sp. C04-09; GQ892823
			Bacteria	Actinobacteria	Actinobacteria	Bifidobacteriales	Bifidobacteriaceae	unclassified_Bifidobacteriaceae	Scardovia sp. T01-04; GQ892824
			Bacteria	Actinobacteria	Actinobacteria	Bifidobacteriales	Bifidobacteriaceae	unclassified_Bifidobacteriaceae	uncultured Scardovia sp.; 22BB47; FJ976387
			Bacteria	Actinobacteria	Actinobacteria	Bifidobacteriales	Bifidobacteriaceae	unclassified_Bifidobacteriaceae	Scardovia sp. T01-04; AY880045
o1347	o1347 16S-BiSpe	GCGGAATTCGTGGTGTAGC	Bacteria	Bacteroidetes	Bacteroidetes	Bacteroidales	Bacteroidaceae	Bacteroides	Bacteroides acidifaciens; A24; AB021159
			Bacteria	Bacteroidetes	Bacteroidetes	Bacteroidales	Bacteroidaceae	Bacteroides	Bacteroides acidifaciens; AB021157
			Bacteria	Bacteroidetes	Bacteroidetes	Bacteroidales	Bacteroidaceae	Bacteroides	Bacteroides acidifaciens; A29; AB021160
			Bacteria	Bacteroidetes	Bacteroidetes	Bacteroidales	Bacteroidaceae	Bacteroides	Bacteroides acidifaciens; A31; AB021161
			Bacteria	Bacteroidetes	Bacteroidetes	Bacteroidales	Bacteroidaceae	Bacteroides	uncultured bacterium; from human colonic sample; HuCA36; AJ408983
			Bacteria	Bacteroidetes	Bacteroidetes	Bacteroidales	Bacteroidaceae	Bacteroides	Bacteroides acidifaciens; A1; AB021158
			Bacteria	Bacteroidetes	Bacteroidetes	Bacteroidales	Bacteroidaceae	Bacteroides	Bacteroides acidifaciens; A43; AB021165
			Bacteria	Bacteroidetes	Bacteroidetes	Bacteroidales	Bacteroidaceae	Bacteroides	uncultured bacterium; from human colonic sample; HuCB3; AJ408988
			Bacteria	Bacteroidetes	Bacteroidetes	Bacteroidales	Prevotellaceae	Prevotella	Prevotella bryantii (T); B14 (DSM 11371, species type strain); AJ006457
			Bacteria	Bacteroidetes	Bacteroidetes	Bacteroidales	Bacteroidaceae	Bacteroides	uncultured bacterium; from human colonic sample; HuCA9; AJ408965
			Bacteria	Bacteroidetes	Bacteroidetes	Bacteroidales	Bacteroidaceae	Bacteroides	uncultured bacterium; from human colonic sample; HuCA21; AJ409009
			Bacteria	Bacteroidetes	Bacteroidetes	Bacteroidales	Bacteroidaceae	Bacteroides	Bacteroides fragilis; Bfr189; 10; X83944
			Bacteria	Bacteroidetes	Bacteroidetes	Bacteroidales	Bacteroidaceae	Bacteroides	Bacteroides fragilis; B119; @8; X83942
			Bacteria	Bacteroidetes	Bacteroidetes	Bacteroidales	Bacteroidaceae	Bacteroides	Bacteroides fragilis; Bfr901; 11; X83945
			Bacteria	Bacteroidetes	Bacteroidetes	Bacteroidales	Bacteroidaceae	Bacteroides	Bacteroides fragilis; 790; @3; X83937
			Bacteria	Bacteroidetes	Bacteroidetes	Bacteroidales	Bacteroidaceae	Bacteroides	Bacteroides eggerthii; DSM 20697T; AB050107
			Bacteria	Bacteroidetes	Bacteroidetes	Bacteroidales	Bacteroidaceae	Bacteroides	Bacteroides fragilis; Bfr920; 12; X83946
			Bacteria	Bacteroidetes	Bacteroidetes	Bacteroidales	Bacteroidaceae	Bacteroides	Bacteroides uniformis; JCM 5828T; AB050110
			Bacteria	Bacteroidetes	Bacteroidetes	Bacteroidales	Bacteroidaceae	Bacteroides	Bacteroides fragilis; 2393; 14; X83948
			Bacteria	Bacteroidetes	Bacteroidetes	Bacteroidales	Bacteroidaceae	Bacteroides	Bacteroides fragilis; 4225; 16; X83950
o1348	o1348 16S-Brebac	TGCAGTAGGGGAGTCTGGA	Bacteria	Actinobacteria	Actinobacteria	Actinomycetales	Brevibacteriaceae	Brevibacterium	Brevibacterium antiquum (T); VKM Ac-2118; AY243344
			Bacteria	Actinobacteria	Actinobacteria	Actinomycetales	Brevibacteriaceae	Brevibacterium	Brevibacterium sanguinis (T); type strain: CF63; AJ564859
			Bacteria	Actinobacteria	Actinobacteria	Actinomycetales	Brevibacteriaceae	Brevibacterium	Brevibacterium sanguinis; CF 52; AJ628351
			Bacteria	Actinobacteria	Actinobacteria	Actinomycetales	Brevibacteriaceae	Brevibacterium	Brevibacterium antarcticum; type strain: DVS 5a2; AJ577724
			Bacteria	Actinobacteria	Actinobacteria	Actinomycetales	Brevibacteriaceae	Brevibacterium	Brevibacterium linens; SB1; AJ315491
			Bacteria	Actinobacteria	Actinobacteria	Actinomycetales	Brevibacteriaceae	Brevibacterium	Brevibacterium iodinum (T); DSM 2062T; X83813
			Bacteria	Actinobacteria	Actinobacteria	Actinomycetales	Brevibacteriaceae	Brevibacterium	Brevibacterium epidermidis (T); NCDO 2286; X76565
			Bacteria	Actinobacteria	Actinobacteria	Actinomycetales	Brevibacteriaceae	Brevibacterium	Brevibacterium linens (T); DSM 20425; X77451
			Bacteria	Actinobacteria	Actinobacteria	Actinomycetales	Brevibacteriaceae	Brevibacterium	Brevibacterium linens; VKM Ac-2119; AY243345
			Bacteria	Actinobacteria	Actinobacteria	Actinomycetales	Brevibacteriaceae	Brevibacterium	Brevibacterium luteolum; type strain: CF87; AJ488509
			Bacteria	Actinobacteria	Actinobacteria	Actinomycetales	Brevibacteriaceae	Brevibacterium	Brevibacterium permense (T); VKM Ac-2280; AY243343
			Bacteria	Actinobacteria	Actinobacteria	Actinomycetales	Brevibacteriaceae	Brevibacterium	uncultured bacterium; PeH13; AJ576331
			Bacteria	Actinobacteria	Actinobacteria	Actinomycetales	Brevibacteriaceae	Brevibacterium	unidentified bacterium; GH443; X76703
			Bacteria	Actinobacteria	Actinobacteria	Actinomycetales	Brevibacteriaceae	Brevibacterium	Brevibacterium casei; DSM 20657; AJ251418
			Bacteria	Actinobacteria	Actinobacteria	Actinomycetales	Brevibacteriaceae	Brevibacterium	Brevibacterium casei (T); NCDO 2048; X76564
			Bacteria	Actinobacteria	Actinobacteria	Actinomycetales	Brevibacteriaceae	Brevibacterium	Brevibacterium iodinum; NCDO 613; X76567
			Bacteria	Actinobacteria	Actinobacteria	Actinomycetales	Brevibacteriaceae	Brevibacterium	Brevibacterium paucivorans (T); CF 62; AJ251463
			Bacteria	Actinobacteria	Actinobacteria	Actinomycetales	Brevibacteriaceae	Brevibacterium	Brevibacterium mcbrellneri (T); ATCC 49030; X93594
			Bacteria	Actinobacteria	Actinobacteria	Actinomycetales	Brevibacteriaceae	Brevibacterium	Brevibacterium avium (T); NCFB 3055; Y17962
			Bacteria	Actinobacteria	Actinobacteria	Actinomycetales	Brevibacteriaceae	Brevibacterium	Brevibacterium otitidis (T); A37/73 (NCFB3053); X93593
o1349	o1349 16S-BurOral	GGGGGATTCATTTCCTCAGT	Bacteria	Proteobacteria	Betaproteobacteria	Burkholderiales	Burkholderiaceae	Cupriavidus	Ralstonia sp. BA-0323; AB050020
			Bacteria	Proteobacteria	Betaproteobacteria	Burkholderiales	Burkholderiaceae	Cupriavidus	Cupriavidus necator; JS705; AF027407
			Bacteria	Proteobacteria	Betaproteobacteria	Burkholderiales	Burkholderiaceae	Cupriavidus	Cupriavidus campinensis; LMG 20576; AY040355
			Bacteria	Proteobacteria	Betaproteobacteria	Burkholderiales	Burkholderiaceae	Cupriavidus	Ralstonia sp. A-471; AF525456
			Bacteria	Proteobacteria	Betaproteobacteria	Burkholderiales	Burkholderiaceae	Cupriavidus	Ralstonia sp. PHS1; AF239160
			Bacteria	Proteobacteria	Betaproteobacteria	Burkholderiales	Burkholderiaceae	Cupriavidus	Cupriavidus campinensis (T); WS2; AF312020
			Bacteria	Proteobacteria	Betaproteobacteria	Burkholderiales	Burkholderiaceae	Cupriavidus	Cupriavidus campinensis; LMG 20568; AY040349
			Bacteria	Proteobacteria	Betaproteobacteria	Burkholderiales	Burkholderiaceae	Cupriavidus	Ralstonia sp. 80; AY238507
			Bacteria	Proteobacteria	Betaproteobacteria	Burkholderiales	Burkholderiaceae	Cupriavidus	Cupriavidus campinensis; LMG 20579; AY040358
			Bacteria	Proteobacteria	Betaproteobacteria	Burkholderiales	Burkholderiaceae	Cupriavidus	Burkholderia sp. oral strain C37KA; AY005039
			Bacteria	Proteobacteria	Betaproteobacteria	Burkholderiales	Burkholderiaceae	Cupriavidus	Ralstonia sp.; CT14; D88001
			Bacteria	Proteobacteria	Betaproteobacteria	Burkholderiales	Comamonadaceae	Caldimonas	Caldimonas taiwanensis (T); On1; AY845052
			Bacteria	Proteobacteria	Betaproteobacteria	Burkholderiales	Burkholderiaceae	Cupriavidus	Ralstonia sp.; CT12; D88009
			Bacteria	Proteobacteria	Betaproteobacteria	Burkholderiales	Burkholderiaceae	Cupriavidus	Cupriavidus gilardii; AU4927; AY860229
			Bacteria	Proteobacteria	Betaproteobacteria	Burkholderiales	Burkholderiaceae	Cupriavidus	Cupriavidus gilardii; AU2756; AY860226
			Bacteria	Proteobacteria	Betaproteobacteria	Burkholderiales	Burkholderiaceae	Cupriavidus	Cupriavidus gilardii; AU3713; AY860227
			Bacteria	Proteobacteria	Betaproteobacteria	Burkholderiales	Burkholderiaceae	Cupriavidus	Ralstonia sp. 1-1; AY583652
			Bacteria	Proteobacteria	Betaproteobacteria	Burkholderiales	Burkholderiaceae	Cupriavidus	Cupriavidus gilardii; AU5554; AY860230
			Bacteria	Proteobacteria	Betaproteobacteria	Burkholderiales	Burkholderiaceae	Cupriavidus	Cupriavidus gilardii; 009\817; AJ306571
			Bacteria	Proteobacteria	Betaproteobacteria	Burkholderiales	Burkholderiaceae	Cupriavidus	Cupriavidus gilardii; AU1529; AY860225

o1350	o1350 16S-BurPhe	GGATCTTAGGACCTCTCGC	Bacteria	Proteobacteria	Betaproteobacteria	Burkholderiales	Burkholderiaceae	Burkholderia	Burkholderia phenazinium (T); LMG2247T; U96936
			Bacteria	Proteobacteria	Betaproteobacteria	Burkholderiales	Burkholderiaceae	Burkholderia	uncultured eubacterium WD268; AJ292643
			Bacteria	Proteobacteria	Betaproteobacteria	Burkholderiales	Burkholderiaceae	Burkholderia	uncultured eubacterium WD202; AJ292637
			Bacteria	Proteobacteria	Betaproteobacteria	Burkholderiales	Burkholderiaceae	Burkholderia	Burkholderia sp. N1US7; AJ884850
			Bacteria	Proteobacteria	Betaproteobacteria	Burkholderiales	Burkholderiaceae	Burkholderia	Burkholderia sp. N1MM10; AJ884849
			Bacteria	Proteobacteria	Betaproteobacteria	Burkholderiales	Burkholderiaceae	Burkholderia	Burkholderia sp. CS2; CS 2; AY178065
			Bacteria	Proteobacteria	Betaproteobacteria	Burkholderiales	Burkholderiaceae	Burkholderia	Burkholderia sp. CS6; CS 6; AY178066
			Bacteria	Proteobacteria	Betaproteobacteria	Burkholderiales	Burkholderiaceae	Burkholderia	Burkholderia sp. LMG 22932; RA13-6; AY949192
			Bacteria	Proteobacteria	Betaproteobacteria	Burkholderiales	Burkholderiaceae	Burkholderia	Burkholderia phenazinium; WT1 4; AM502987
			Bacteria	Proteobacteria	Betaproteobacteria	Burkholderiales	Burkholderiaceae	Burkholderia	Burkholderia phenazinium; WT1 6; AM502989
			Bacteria	Proteobacteria	Betaproteobacteria	Burkholderiales	Burkholderiaceae	Burkholderia	Burkholderia phenazinium; WT1 10; AM502992
			Bacteria	Proteobacteria	Betaproteobacteria	Burkholderiales	Burkholderiaceae	Burkholderia	Burkholderia phenazinium; WT1 5; AM502988
			Bacteria	Proteobacteria	Betaproteobacteria	Burkholderiales	Burkholderiaceae	Burkholderia	Burkholderia sp. DM5; DQ419951
			Bacteria	Proteobacteria	Betaproteobacteria	Burkholderiales	Burkholderiaceae	Burkholderia	Burkholderia phenazinium; WT1 7; AM502990
			Bacteria	Proteobacteria	Betaproteobacteria	Burkholderiales	Burkholderiaceae	Burkholderia	Burkholderia phenazinium; WT1 11; AM502993
			Bacteria	Proteobacteria	Betaproteobacteria	Burkholderiales	Burkholderiaceae	Burkholderia	Burkholderia phenazinium; WT1 9; AM502991
			Bacteria	Proteobacteria	Betaproteobacteria	Burkholderiales	Burkholderiaceae	Burkholderia	Burkholderia sp. DM-Ni1; DQ419958
			Bacteria	Proteobacteria	Betaproteobacteria	Burkholderiales	Burkholderiaceae	Burkholderia	uncultured bacterium; WIM13.6; DQ202245
			Bacteria	Proteobacteria	Betaproteobacteria	Burkholderiales	Burkholderiaceae	Burkholderia	Burkholderia sp. RW36; AJ300692
			Bacteria	Proteobacteria	Betaproteobacteria	Burkholderiales	Burkholderiaceae	Burkholderia	Burkholderia sp. 2As; AM503077
o1352	o1352 16S-BuvibGroup	CCGTAAGGAGGGAGCAGC	Bacteria	Firmicutes	Clostridia	Clostridiales	Lachnospiraceae	Pseudobutyrivibrio	Pseudobutyrivibrio ruminis; pC-XS2; AF202260
			Bacteria	Firmicutes	Clostridia	Clostridiales	Lachnospiraceae	Pseudobutyrivibrio	Pseudobutyrivibrio ruminis; Ce1; AY178841
			Bacteria	Firmicutes	Clostridia	Clostridiales	Lachnospiraceae	Pseudobutyrivibrio	Pseudobutyrivibrio ruminis; pC-XS7; AF202262
			Bacteria	Firmicutes	Clostridia	Clostridiales	Lachnospiraceae	Butyrivibrio	Firmicutes oral clone BB124; AF287772
			Bacteria	Firmicutes	Clostridia	Clostridiales	Lachnospiraceae	Pseudobutyrivibrio	Pseudobutyrivibrio ruminis; M80; AY699284
			Bacteria	Firmicutes	Clostridia	Clostridiales	Lachnospiraceae	Pseudobutyrivibrio	Pseudobutyrivibrio ruminis; L4; AY699285
			Bacteria	Firmicutes	Clostridia	Clostridiales	Lachnospiraceae	Pseudobutyrivibrio	Pseudobutyrivibrio ruminis; pC-XS6; AF202261
			Bacteria	Firmicutes	Clostridia	Clostridiales	Lachnospiraceae	Pseudobutyrivibrio	Butyrivibrio fibrisolvens; NCDO 2249; X89978
			Bacteria	Firmicutes	Clostridia	Clostridiales	Lachnospiraceae	Pseudobutyrivibrio	Butyrivibrio fibrisolvens; B835; AF125217
			Bacteria	Firmicutes	Clostridia	Clostridiales	Lachnospiraceae	Pseudobutyrivibrio	Butyrivibrio fibrisolvens; NCDO 2397; X89979
			Bacteria	Firmicutes	Clostridia	Clostridiales	Lachnospiraceae	Pseudobutyrivibrio	Pseudobutyrivibrio ruminis; Ce4; AY178843
			Bacteria	Firmicutes	Clostridia	Clostridiales	Lachnospiraceae	Pseudobutyrivibrio	Butyrivibrio fibrisolvens; NCDO 2399; X89980
			Bacteria	Firmicutes	Clostridia	Clostridiales	Lachnospiraceae	Pseudobutyrivibrio	Pseudobutyrivibrio ruminis (T); DSM 9787; X95893
			Bacteria	Firmicutes	Clostridia	Clostridiales	Lachnospiraceae	Pseudobutyrivibrio	Butyrivibrio fibrisolvens; NCDO 2223; X89977
			Bacteria	Firmicutes	Clostridia	Clostridiales	Lachnospiraceae	Pseudobutyrivibrio	Pseudobutyrivibrio xylanivorans; JK 23/2; AJ428550
			Bacteria	Firmicutes	Clostridia	Clostridiales	Lachnospiraceae	Pseudobutyrivibrio	Butyrivibrio fibrisolvens; 16.4; human feaces; AJ250365
			Bacteria	Firmicutes	Clostridia	Clostridiales	Lachnospiraceae	Pseudobutyrivibrio	unidentified rumen bacterium RC13; AF001704
			Bacteria	Firmicutes	Clostridia	Clostridiales	Lachnospiraceae	Pseudobutyrivibrio	Pseudobutyrivibrio ruminis; JK 626; AJ428549
			Bacteria	Firmicutes	Clostridia	Clostridiales	Lachnospiraceae	Pseudobutyrivibrio	Pseudobutyrivibrio xylanivorans (T); Mz 5; AJ428548
			Bacteria	Firmicutes	Clostridia	Clostridiales	Lachnospiraceae	Pseudobutyrivibrio	Butyrivibrio cf. fibrisolvens EAT6; AF264034
o1353	o1353 16S-CaGroup1	TATGGATGGACCCGCGGC	Bacteria	Firmicutes	Bacilli	Lactobacillales	Carnobacteriaceae	Carnobacterium	uncultured bacterium; wet197; AY212748
			Bacteria	Firmicutes	Bacilli	Bacillales	Bacillaceae	Bacillus d	Bacillus cereus; AY986507
			Bacteria	Firmicutes	Bacilli	Lactobacillales	Carnobacteriaceae	Trichococcus	Trichococcus flocculiformis (T); DSM 2094; Y17301
			Bacteria	Firmicutes	Bacilli	Lactobacillales	Carnobacteriaceae	Trichococcus	Trichococcus collinsii (T); 37AN3; AJ306612
			Bacteria	Firmicutes	Bacilli	Lactobacillales	Carnobacteriaceae	Carnobacterium	uncultured rumen bacterium; BS28; AY244979
			Bacteria	Firmicutes	Bacilli	Lactobacillales	Carnobacteriaceae	Trichococcus	Trichococcus palustris (T); DSM 9172T; AJ296179
			Bacteria	Firmicutes	Bacilli	Lactobacillales	Carnobacteriaceae	Isobaculum	Isobaculum melis (T); CCUG 37660T; AJ302648
			Bacteria	Firmicutes	Bacilli	Lactobacillales	Carnobacteriaceae	Carnobacterium	Carnobacterium pleistocenium (T); FTR1; AF450136
			Bacteria	Firmicutes	Bacilli	Lactobacillales	Carnobacteriaceae	Carnobacterium	Carnobacterium funditum (T); pf3 (DSM 5970); S86170
			Bacteria	Firmicutes	Bacilli	Bacillales	Paenibacillaceae	Paenibacillus	uncultured bacterium; 17RHF17; AJ863344
			Bacteria	Firmicutes	Bacilli	Lactobacillales	Carnobacteriaceae	Trichococcus	Trichococcus patagoniensis (T); PMagG1; AF394926
			Bacteria	Firmicutes	Bacilli	Lactobacillales	Carnobacteriaceae	Carnobacterium	Carnobacterium viridans (T); MPL-11; AF425608
			Bacteria	Firmicutes	Bacilli	Lactobacillales	Carnobacteriaceae	Marinilactibacillus	Marinilactibacillus psychrotolerans (T); M13-2; AB083406
			Bacteria	Firmicutes	Bacilli	Bacillales	Paenibacillaceae	Paenibacillus	Paenibacillus glycanilyticus (T); DS-1; AB042938
			Bacteria	Firmicutes	Bacilli	Bacillales	Bacillaceae	Bacillus a	Bacillus subtilis subsp. subtilis; BGSC 3A23; AY310301
			Bacteria	Firmicutes	Bacilli	Lactobacillales	Carnobacteriaceae	Alkalibacterium	Alkalibacterium psychrotolerans (T); IDR2-2; AB125938
			Bacteria	Firmicutes	Bacilli	Lactobacillales	Carnobacteriaceae	Trichococcus	Trichococcus pasteurii (T); ATCC 35945; L76599
			Bacteria	Firmicutes	Bacilli	Lactobacillales	Carnobacteriaceae	Marinilactibacillus	Marinilactibacillus piezotolerans (T); LT20; AY485792
			Bacteria	Firmicutes	Bacilli	Lactobacillales	Carnobacteriaceae	Carnobacterium	uncultured bacterium; wet51; AY212754
			Bacteria	Firmicutes	Bacilli	Lactobacillales	Carnobacteriaceae	Carnobacterium	Carnobacterium gallinarum (T); NCFB 2766 T; X54269
o1354	o1354 16S-Cagroup3	AACTGGGGAAGGTCATTGGA	Bacteria	Firmicutes	Bacilli	Lactobacillales	Carnobacteriaceae	Carnobacterium	Carnobacterium sp. enrichment culture clone 12-11E; EU517560
			Bacteria	Firmicutes	Bacilli	Lactobacillales	Enterococcaceae	Vagococcus	Vagococcus elongatus; PPC9; AF445297
			Bacteria	Firmicutes	Bacilli	Lactobacillales	Carnobacteriaceae	Carnobacterium	Carnobacterium alterfunditum; L08623
			Bacteria	Firmicutes	Bacilli	Lactobacillales	Carnobacteriaceae	Carnobacterium	Carnobacterium sp. NJ-46; AM396913
			Bacteria	Firmicutes	Bacilli	Lactobacillales	Carnobacteriaceae	Carnobacterium	Carnobacterium sp. ARCTIC-P26; AY573047
			Bacteria	Firmicutes	Bacilli	Lactobacillales	Carnobacteriaceae	Carnobacterium	Carnobacterium sp. BBDP54; DQ337521
			Bacteria	Firmicutes	Bacilli	Lactobacillales	Carnobacteriaceae	Carnobacterium	Carnobacterium sp. BBDP71; DQ337531
			Bacteria	Firmicutes	Bacilli	Lactobacillales	Carnobacteriaceae	Carnobacterium	Carnobacterium sp.; R-7279; AJ427446
			Bacteria	Firmicutes	Bacilli	Lactobacillales	Carnobacteriaceae	Carnobacterium	uncultured rumen bacterium; BS28; AY244979
			Bacteria	Firmicutes	Bacilli	Lactobacillales	Carnobacteriaceae	Carnobacterium	uncultured bacterium; WF16S_150; EU939414
			Bacteria	Firmicutes	Bacilli	Bacillales	Bacillaceae	Bacillus a	Bacillus sp. G2DM-52; DQ416787
			Bacteria	Firmicutes	Bacilli	Lactobacillales	Enterococcaceae	Vagococcus	Vagococcus sp. SSCS12; AB211029
			Bacteria	Firmicutes	Bacilli	Lactobacillales	Carnobacteriaceae	Carnobacterium	Carnobacterium pleistocenium (T); FTR1; AF450136
			Bacteria	Firmicutes	Bacilli	Lactobacillales	Carnobacteriaceae	Carnobacterium	uncultured Carnobacterium sp.; 12-11E; EF420230
			Bacteria	Firmicutes	Bacilli	Lactobacillales	Carnobacteriaceae	Carnobacterium	Carnobacterium funditum (T); pf3 (DSM 5970); S86170
			Bacteria	Firmicutes	Bacilli	Lactobacillales	Carnobacteriaceae	Carnobacterium	Carnobacterium mobile; DSM 4848; AB083414
			Bacteria	Firmicutes	Bacilli	Lactobacillales	Enterococcaceae	Vagococcus	Vagococcus lutrae; F01595; DQ395281
			Bacteria	Firmicutes	Bacilli	Lactobacillales	Carnobacteriaceae	Carnobacterium	Carnobacterium sp. St2; AF061558
			Bacteria	Firmicutes	Bacilli	Lactobacillales	Enterococcaceae	Vagococcus	Vagococcus lutrae (T); m1134/97/1; CCUG 39187; Y17152
			Bacteria	Firmicutes	Bacilli	Bacillales	Bacillaceae	Bacillus a	Bacillus pumilus; 4RS-5f; EU379284
o1355	o1355 16S-CamCon	AGAACTGCTTGGGAAACTGG	Bacteria	Proteobacteria	Epsilonproteobacteria	Campylobacterales	Campylobacteraceae	Campylobacter	Campylobacter concisus; LMG 7545; AF550653
			Bacteria	Proteobacteria	Epsilonproteobacteria	Campylobacterales	Campylobacteraceae	Campylobacter	Campylobacter concisus (T); L04322
			Bacteria	Proteobacteria	Epsilonproteobacteria	Campylobacterales	Campylobacteraceae	Campylobacter	Campylobacter concisus; ATCC 33237; DQ174166
			Bacteria	Proteobacteria	Epsilonproteobacteria	Campylobacterales	Campylobacteraceae	Campylobacter	Campylobacter concisus; L06977
			Bacteria	Proteobacteria	Epsilonproteobacteria	Campylobacterales	Campylobacteraceae	Campylobacter	Campylobacter concisus; NZ539-95; DQ174167
			Bacteria	Proteobacteria	Epsilonproteobacteria	Campylobacterales	Campylobacteraceae	Campylobacter	uncultured Campylobacter sp.; 7BB730; FJ976279
			Bacteria	Proteobacteria	Epsilonproteobacteria	Campylobacterales	Campylobacteraceae	Campylobacter	uncultured bacterium; hoa5_05e09; EU344611
o1356	o1356 16S-CamGra	ACCTATTGCGAAGGCGATCT	Bacteria	Proteobacteria	Epsilonproteobacteria	Campylobacterales	Campylobacteraceae	Campylobacter	uncultured Campylobacter sp.; 601D08(oral); AM420221

			Bacteria	Proteobacteria	Epsilonproteobacteria	Campylobacterales	Campylobacteraceae	Campylobacter	uncultured bacterium; P3D1-427; EF509169
			Bacteria	Proteobacteria	Epsilonproteobacteria	Campylobacterales	Helicobacteraceae	unclassified_Helicobacteraceae	uncultured organism; MAT-CR-M2-D10; EU245507
			Bacteria	Proteobacteria	Epsilonproteobacteria	Campylobacterales	Campylobacteraceae	Campylobacter	Campylobacter gracilis; L37787
			Bacteria	Proteobacteria	Epsilonproteobacteria	Campylobacterales	Campylobacteraceae	Campylobacter	Campylobacter gracilis; CCUG 27721; AF550658
			Bacteria	Proteobacteria	Epsilonproteobacteria	Campylobacterales	Campylobacteraceae	Campylobacter	Campylobacter gracilis; LMG 7616; AF550656
			Bacteria	Proteobacteria	Epsilonproteobacteria	Campylobacterales	Campylobacteraceae	Campylobacter	Campylobacter gracilis; CCUG 13143; AF550657
			Bacteria	Proteobacteria	Epsilonproteobacteria	Campylobacterales	Campylobacteraceae	Campylobacter	Campylobacter gracilis (T); L04320
			Bacteria	Proteobacteria	Epsilonproteobacteria	Campylobacterales	Campylobacteraceae	Campylobacter	Campylobacter sp. oral clone OH5A; AY947499
			Bacteria	Proteobacteria	Epsilonproteobacteria	Campylobacterales	Campylobacteraceae	Campylobacter	uncultured Campylobacter sp.; 6BBA45; FJ976262
			Bacteria	Proteobacteria	Epsilonproteobacteria	Campylobacterales	Campylobacteraceae	Campylobacter	uncultured bacterium; LY37; AY976358
			Bacteria	Proteobacteria	Epsilonproteobacteria	Campylobacterales	Campylobacteraceae	Campylobacter	uncultured bacterium; LL76; AY980672
			Bacteria	Proteobacteria	Epsilonproteobacteria	Campylobacterales	Campylobacteraceae	Campylobacter	Campylobacter gracilis; ATCC 33236; DQ174168
			Bacteria	Proteobacteria	Epsilonproteobacteria	Campylobacterales	Campylobacteraceae	Campylobacter	uncultured bacterium; nbu87f12c1; GQ006788
o1357	o1357 16S-CamOral	CCATAGTGGAGGACAACAGT	Bacteria	Proteobacteria	Epsilonproteobacteria	Campylobacterales	Campylobacteraceae	Campylobacter	Campylobacter curvus; LMG 11034; AF550650
			Bacteria	Proteobacteria	Epsilonproteobacteria	Campylobacterales	Campylobacteraceae	Campylobacter	Campylobacter curvus; LMG 11127; AF550651
			Bacteria	Proteobacteria	Epsilonproteobacteria	Campylobacterales	Campylobacteraceae	Campylobacter	Campylobacter curvus; LMG 13936; AF550652
			Bacteria	Proteobacteria	Epsilonproteobacteria	Campylobacterales	Campylobacteraceae	Campylobacter	Campylobacter sp. oral clone BB120; AY005038
			Bacteria	Proteobacteria	Epsilonproteobacteria	Campylobacterales	Campylobacteraceae	Campylobacter	Campylobacter curvus; ATCC 35224; DQ174165
			Bacteria	Proteobacteria	Epsilonproteobacteria	Campylobacterales	Campylobacteraceae	Campylobacter	uncultured bacterium; 284; GQ398428
			Bacteria	Proteobacteria	Epsilonproteobacteria	Campylobacterales	Campylobacteraceae	Campylobacter	uncultured Campylobacter sp.; 202B08(oral); AM420041
			Bacteria	Proteobacteria	Epsilonproteobacteria	Campylobacterales	Campylobacteraceae	Campylobacter	uncultured Campylobacter sp.; 3178; FJ976205
o1358	o1358 16S-CamSho	CTTGCCCATTACTAAGGGAC	Bacteria	Proteobacteria	Epsilonproteobacteria	Campylobacterales	Campylobacteraceae	Campylobacter	uncultured bacterium; A_D_02_02; FJ470450
			Bacteria	Proteobacteria	Epsilonproteobacteria	Campylobacterales	Campylobacteraceae	Campylobacter	uncultured Campylobacter sp.; 102B05(oral); AM419984
			Bacteria	Proteobacteria	Epsilonproteobacteria	Campylobacterales	Campylobacteraceae	Campylobacter	Campylobacter showae; UNSWCD; GQ167660
			Bacteria	Proteobacteria	Epsilonproteobacteria	Campylobacterales	Campylobacteraceae	Campylobacter	Campylobacter showae; LMG 12636; AF550655
			Bacteria	Proteobacteria	Epsilonproteobacteria	Campylobacterales	Campylobacteraceae	Campylobacter	Campylobacter showae (T); L06974
			Bacteria	Proteobacteria	Epsilonproteobacteria	Campylobacterales	Campylobacteraceae	Campylobacter	Campylobacter showae; L06975
			Bacteria	Proteobacteria	Epsilonproteobacteria	Campylobacterales	Campylobacteraceae	Campylobacter	Campylobacter showae; CCUG 30254; DQ174155
			Bacteria	Proteobacteria	Epsilonproteobacteria	Campylobacterales	Campylobacteraceae	Campylobacter	Campylobacter showae; CCUG 11641; DQ174156
			Bacteria	Proteobacteria	Epsilonproteobacteria	Campylobacterales	Campylobacteraceae	Campylobacter	uncultured Campylobacter sp.; 6BB501; FJ976254
			Bacteria	Proteobacteria	Epsilonproteobacteria	Campylobacterales	Campylobacteraceae	Campylobacter	Campylobacter showae; 3; EU781616
o1359	o1359 16S-CapGin	AACTGCGGTGGTGAAAGATG	Bacteria	Bacteroidetes	Flavobacteria	Flavobacteriales	Flavobacteriaceae	Capnocytophaga	Capnocytophaga gingivalis; ChDC OS45; AF543295
			Bacteria	Bacteroidetes	Flavobacteria	Flavobacteriales	Flavobacteriaceae	Capnocytophaga	uncultured bacterium; A_S_01_44; FJ470580
			Bacteria	Bacteroidetes	Flavobacteria	Flavobacteriales	Flavobacteriaceae	Capnocytophaga	uncultured Capnocytophaga sp.; 501H09(oral); AM420180
			Bacteria	Bacteroidetes	Flavobacteria	Flavobacteriales	Flavobacteriaceae	Capnocytophaga	uncultured Capnocytophaga sp.; GI5-008-G03; FJ192591
			Bacteria	Bacteroidetes	Flavobacteria	Flavobacteriales	Flavobacteriaceae	Capnocytophaga	Capnocytophaga gingivalis (T); ATCC 33624; X67608
			Bacteria	Bacteroidetes	Flavobacteria	Flavobacteriales	Flavobacteriaceae	Capnocytophaga	Capnocytophaga gingivalis; L14639
			Bacteria	Firmicutes	Clostridia	Clostridiales	Veillonellaceae	Succiniclasticum	uncultured bacterium; 1103200842882; EU843614
			Bacteria	Bacteroidetes	Flavobacteria	Flavobacteriales	Flavobacteriaceae	Capnocytophaga	uncultured Capnocytophaga sp.; 18B743; FJ976371
			Bacteria	Bacteroidetes	Flavobacteria	Flavobacteriales	Flavobacteriaceae	Capnocytophaga	uncultured bacterium; nbw357f12c1; GQ087460
			Bacteria	Bacteroidetes	Flavobacteria	Flavobacteriales	Flavobacteriaceae	Capnocytophaga	uncultured bacterium; nbw1131b05c1; GQ079749
			Bacteria	Bacteroidetes	Flavobacteria	Flavobacteriales	Flavobacteriaceae	Capnocytophaga	uncultured Capnocytophaga sp.; 10B747; FJ976319
o1360	o1360 16S-CapGroup1	GAGCAGGCGCGAATCTATAA	Bacteria	Bacteroidetes	Flavobacteria	Flavobacteriales	Flavobacteriaceae	Capnocytophaga	Capnocytophaga ochracea; ATCC 33596; X67610
			Bacteria	Bacteroidetes	Flavobacteria	Flavobacteriales	Flavobacteriaceae	Capnocytophaga	Capnocytophaga sp. oral clone EL043; AY008312
			Bacteria	Bacteroidetes	Flavobacteria	Flavobacteriales	Flavobacteriaceae	Capnocytophaga	uncultured Capnocytophaga sp.; DS022; AF366270
			Bacteria	Bacteroidetes	Flavobacteria	Flavobacteriales	Flavobacteriaceae	Capnocytophaga	Capnocytophaga ochracea; ChDC OS42; AF543292
			Bacteria	Bacteroidetes	Flavobacteria	Flavobacteriales	Flavobacteriaceae	Capnocytophaga	Capnocytophaga ochracea; ChDC OS48; AF543298
			Bacteria	Bacteroidetes	Flavobacteria	Flavobacteriales	Flavobacteriaceae	Capnocytophaga	Capnocytophaga sp. ChDC OS44; AF543294
			Bacteria	Bacteroidetes	Flavobacteria	Flavobacteriales	Flavobacteriaceae	Capnocytophaga	Capnocytophaga sp. P2 oral strain P4P_12; AY429469
			Bacteria	Bacteroidetes	Flavobacteria	Flavobacteriales	Flavobacteriaceae	Capnocytophaga	Capnocytophaga sp. oral clone BU084; AF385569
			Bacteria	Bacteroidetes	Flavobacteria	Flavobacteriales	Flavobacteriaceae	Capnocytophaga	Capnocytophaga sp. ChDC OS43; AF543293
			Bacteria	Bacteroidetes	Flavobacteria	Flavobacteriales	Flavobacteriaceae	Capnocytophaga	Capnocytophaga sp. oral clone BB167; AY005076
			Bacteria	Bacteroidetes	Flavobacteria	Flavobacteriales	Flavobacteriaceae	Capnocytophaga	Capnocytophaga ochracea (T); ATCC 27872 T; U41350
			Bacteria	Bacteroidetes	Flavobacteria	Flavobacteriales	Flavobacteriaceae	Capnocytophaga	Capnocytophaga genomosp. C1; C2MKM106; AY278613
			Bacteria	Bacteroidetes	Flavobacteria	Flavobacteriales	Flavobacteriaceae	Capnocytophaga	Capnocytophaga ochracea; L14635
			Bacteria	Bacteroidetes	Flavobacteria	Flavobacteriales	Flavobacteriaceae	Capnocytophaga	Capnocytophaga sp. oral clone X089; AY005080
			Bacteria	Bacteroidetes	Flavobacteria	Flavobacteriales	Flavobacteriaceae	Capnocytophaga	Capnocytophaga sp. oral clone X066; AY005078
			Bacteria	Bacteroidetes	Flavobacteria	Flavobacteriales	Flavobacteriaceae	Capnocytophaga	Capnocytophaga sp. oral strain P4G_35 P4; AF538853
			Bacteria	Bacteroidetes	Flavobacteria	Flavobacteriales	Flavobacteriaceae	Capnocytophaga	Capnocytophaga sp. oral clone AA032; AY005079
			Bacteria	Bacteroidetes	Flavobacteria	Flavobacteriales	Flavobacteriaceae	Capnocytophaga	Capnocytophaga ochracea; LMG 12117; FDC 43; U41351
			Bacteria	Bacteroidetes	Flavobacteria	Flavobacteriales	Flavobacteriaceae	Capnocytophaga	Capnocytophaga ochracea; FDC 7b; U41354
			Bacteria	Bacteroidetes	Flavobacteria	Flavobacteriales	Flavobacteriaceae	Capnocytophaga	Capnocytophaga ochracea; LMG 12115; FDC 7; U41353
o1361	o1361 16S-CapGroup2	AGGGGTGAAAGGTTTCAGCT	Bacteria	Bacteroidetes	Flavobacteria	Flavobacteriales	Flavobacteriaceae	Capnocytophaga	Capnocytophaga gingivalis; ChDC OS45; AF543295
			Bacteria	Bacteroidetes	Flavobacteria	Flavobacteriales	Flavobacteriaceae	Capnocytophaga	uncultured Capnocytophaga sp.; 502B09(oral); AM420187
			Bacteria	Bacteroidetes	Flavobacteria	Flavobacteriales	Flavobacteriaceae	Capnocytophaga	uncultured bacterium; A_D_02_21; FJ470456
			Bacteria	Bacteroidetes	Flavobacteria	Flavobacteriales	Flavobacteriaceae	Capnocytophaga	uncultured bacterium; A_D_02_52; FJ470473
			Bacteria	Bacteroidetes	Flavobacteria	Flavobacteriales	Flavobacteriaceae	Capnocytophaga	uncultured Capnocytophaga sp.; 102E05(oral); AM419994
			Bacteria	Bacteroidetes	Flavobacteria	Flavobacteriales	Flavobacteriaceae	Capnocytophaga	uncultured Capnocytophaga sp.; 303G05(oral); AM420112
			Bacteria	Bacteroidetes	Flavobacteria	Flavobacteriales	Flavobacteriaceae	Capnocytophaga	uncultured Capnocytophaga sp.; 501C07(oral); AM420168
			Bacteria	Bacteroidetes	Flavobacteria	Flavobacteriales	Flavobacteriaceae	Capnocytophaga	uncultured Capnocytophaga sp.; 501H09(oral); AM420180
			Bacteria	Bacteroidetes	Flavobacteria	Flavobacteriales	Flavobacteriaceae	Capnocytophaga	uncultured Capnocytophaga sp.; GI5-008-G03; FJ192591
			Bacteria	Bacteroidetes	Flavobacteria	Flavobacteriales	Flavobacteriaceae	Capnocytophaga	Capnocytophaga sp. P1 oral clone P4GA_52; AY429468
			Bacteria	Bacteroidetes	Flavobacteria	Flavobacteriales	Flavobacteriaceae	Capnocytophaga	Capnocytophaga sp.; S3; U42010
			Bacteria	Bacteroidetes	Flavobacteria	Flavobacteriales	Flavobacteriaceae	Capnocytophaga	Capnocytophaga sp. oral strain S3; AY005073
			Bacteria	Bacteroidetes	Flavobacteria	Flavobacteriales	Flavobacteriaceae	Capnocytophaga	Capnocytophaga gingivalis; LMG 12118; FDC 44; U41346
			Bacteria	Bacteroidetes	Flavobacteria	Flavobacteriales	Flavobacteriaceae	Capnocytophaga	Capnocytophaga granulosa; LMG16022 T; JCM8566 T; U41347
			Bacteria	Bacteroidetes	Flavobacteria	Flavobacteriales	Flavobacteriaceae	Capnocytophaga	Capnocytophaga granulosa; LMG 12119; FDC SD4; U41348
			Bacteria	Bacteroidetes	Flavobacteria	Flavobacteriales	Flavobacteriaceae	Capnocytophaga	Capnocytophaga granulosa (T); ATCC 51502; X97248
			Bacteria	Bacteroidetes	Flavobacteria	Flavobacteriales	Flavobacteriaceae	Capnocytophaga	Capnocytophaga sp. oral clone AH015; AY005074
			Bacteria	Bacteroidetes	Flavobacteria	Flavobacteriales	Flavobacteriaceae	Capnocytophaga	Capnocytophaga granulosa; GiTC; FJ823150
			Bacteria	Bacteroidetes	Flavobacteria	Flavobacteriales	Flavobacteriaceae	Capnocytophaga	uncultured bacterium; nbw1131b05c1; GQ079749
			Bacteria	Bacteroidetes	Flavobacteria	Flavobacteriales	Flavobacteriaceae	Capnocytophaga	uncultured bacterium; nbw207f10c1; GQ073322
o1362	o1362 16S-CapOch	AGCTCTGGTGGTGAAAGATG	Bacteria	Bacteroidetes	Flavobacteria	Flavobacteriales	Flavobacteriaceae	Capnocytophaga	Capnocytophaga ochracea; ATCC 33596; X67610
			Bacteria	Bacteroidetes	Flavobacteria	Flavobacteriales	Flavobacteriaceae	Capnocytophaga	Capnocytophaga ochracea; F0287; FJ577260
			Bacteria	Bacteroidetes	Flavobacteria	Flavobacteriales	Flavobacteriaceae	Capnocytophaga	Capnocytophaga ochracea; L14635
			Bacteria	Bacteroidetes	Flavobacteria	Flavobacteriales	Flavobacteriaceae	Capnocytophaga	uncultured Bacteroidetes bacterium; INT-G-35A-1; EU432420
			Bacteria	Bacteroidetes	Flavobacteria	Flavobacteriales	Flavobacteriaceae	Capnocytophaga	Capnocytophaga sputigena (T); ATCC 33612; X67609
o1363	o1363 16S-Capspu	ACATAGGGATAGCCCGAAGA	Bacteria	Bacteroidetes	Flavobacteria	Flavobacteriales	Flavobacteriaceae	Capnocytophaga	Capnocytophaga sputigena; CIP 100 897; AF133536

			Bacteria	Bacteroidetes	Flavobacteria	Flavobacteriales	Flavobacteriaceae	Capnocytophaga	uncultured Capnocytophaga sp.; 402F05(oral); AM420149
			Bacteria	Bacteroidetes	Flavobacteria	Flavobacteriales	Flavobacteriaceae	Capnocytophaga	uncultured Capnocytophaga sp.; 502A03(oral); AM420156
			Bacteria	Bacteroidetes	Flavobacteria	Flavobacteriales	Flavobacteriaceae	Capnocytophaga	uncultured Capnocytophaga sp.; 601H01(oral); AM420227
			Bacteria	Bacteroidetes	Flavobacteria	Flavobacteriales	Flavobacteriaceae	Capnocytophaga	Capnocytophaga leadbetteri; AHN8855; DQ009623
			Bacteria	Bacteroidetes	Flavobacteria	Flavobacteriales	Flavobacteriaceae	Capnocytophaga	Capnocytophaga sp. AHN8996; DQ012361
			Bacteria	Bacteroidetes	Flavobacteria	Flavobacteriales	Flavobacteriaceae	Capnocytophaga	Capnocytophaga sp. oral clone BU084; AF385569
			Bacteria	Bacteroidetes	Flavobacteria	Flavobacteriales	Flavobacteriaceae	Capnocytophaga	Capnocytophaga sp. AHN8725; DQ012357
			Bacteria	Bacteroidetes	Flavobacteria	Flavobacteriales	Flavobacteriaceae	Capnocytophaga	Capnocytophaga sp. oral clone BR085; AY134894
			Bacteria	Bacteroidetes	Flavobacteria	Flavobacteriales	Flavobacteriaceae	Capnocytophaga	Capnocytophaga sp. AHN8708; DQ012356
			Bacteria	Bacteroidetes	Flavobacteria	Flavobacteriales	Flavobacteriaceae	Capnocytophaga	Capnocytophaga sputigena; L14636
			Bacteria	Bacteroidetes	Flavobacteria	Flavobacteriales	Flavobacteriaceae	Capnocytophaga	uncultured bacterium; nbw107g02c1; GQ007639
			Bacteria	Bacteroidetes	Flavobacteria	Flavobacteriales	Flavobacteriaceae	Capnocytophaga	uncultured bacterium; nbw765f09c1; GQ015205
			Bacteria	Bacteroidetes	Flavobacteria	Flavobacteriales	Flavobacteriaceae	Capnocytophaga	uncultured bacterium; nbw766d09c1; GQ015269
			Bacteria	Bacteroidetes	Flavobacteria	Flavobacteriales	Flavobacteriaceae	Capnocytophaga	uncultured bacterium; nbu305g01c1; GQ022838
			Bacteria	Bacteroidetes	Flavobacteria	Flavobacteriales	Flavobacteriaceae	Capnocytophaga	uncultured bacterium; nbu88b04c1; GQ006820
			Bacteria	Bacteroidetes	Flavobacteria	Flavobacteriales	Flavobacteriaceae	Capnocytophaga	uncultured bacterium; nbu306h04c1; GQ022937
			Bacteria	Bacteroidetes	Flavobacteria	Flavobacteriales	Flavobacteriaceae	Capnocytophaga	Capnocytophaga sp. S12-14; AY880056
			Bacteria	Bacteroidetes	Flavobacteria	Flavobacteriales	Flavobacteriaceae	Capnocytophaga	Capnocytophaga sp. AHN8730; DQ012358
o1364	o1364 16S-CarGroup	ACTGACCGCTATGGAGACAT	Bacteria	Firmicutes	Bacilli	Bacillales	Planococcaceae	Pasteuriaceae Incertae Sedis	Bacillus sp. YT0042; AB362831
			Bacteria	Firmicutes	Bacilli	Bacillales	Planococcaceae	Pasteuriaceae Incertae Sedis	uncultured Bacillus sp.; DGGE band a; ACf184; AM489493
			Bacteria	Firmicutes	Bacilli	Bacillales	Planococcaceae	Pasteuriaceae Incertae Sedis	uncultured Bacillus sp.; SMa248; AM930505
			Bacteria	Firmicutes	Bacilli	Bacillales	Planococcaceae	Pasteuriaceae Incertae Sedis	uncultured bacterium; AKIW935; DQ129414
			Bacteria	Firmicutes	Bacilli	Bacillales	Planococcaceae	Caryophanon	Caryophanon latum; NCDO 2034; X70319
			Bacteria	Firmicutes	Bacilli	Bacillales	Planococcaceae	Caryophanon	Caryophanon latum; NCIMB 9533; X70314
			Bacteria	Firmicutes	Bacilli	Bacillales	Planococcaceae	Caryophanon	Caryophanon tenue; NCDO 2324; X70315
			Bacteria	Firmicutes	Bacilli	Bacillales	Planococcaceae	Pasteuriaceae Incertae Sedis	Bacillus massiliensis (T); 4400831; AY677116
			Bacteria	Firmicutes	Bacilli	Bacillales	Planococcaceae	Pasteuriaceae Incertae Sedis	Bacillus massiliensis; ZC9; FJ263024
o1366	o1366 16S-CarSpB	AAGATGAGCCCATGTCGGAT	Bacteria	Proteobacteria	Gammaproteobacteria	Cardiobacteriales	Cardiobacteriaceae	Cardiobacterium	Cardiobacterium valvarum; CCUG31208; AY596468
			Bacteria	Proteobacteria	Gammaproteobacteria	Cardiobacteriales	Cardiobacteriaceae	Cardiobacterium	Cardiobacterium valvarum (T); MDA3079; AF506987
			Bacteria	Proteobacteria	Gammaproteobacteria	Cardiobacteriales	Cardiobacteriaceae	Cardiobacterium	Cardiobacterium valvarum; CCUG13150; AY596469
			Bacteria	Proteobacteria	Gammaproteobacteria	Cardiobacteriales	Cardiobacteriaceae	Cardiobacterium	Cardiobacterium valvarum; CCUG12990; AY596470
			Bacteria	Proteobacteria	Gammaproteobacteria	Cardiobacteriales	Cardiobacteriaceae	Cardiobacterium	Cardiobacterium sp. B; AB 2168; AF144696
			Bacteria	Proteobacteria	Gammaproteobacteria	Cardiobacteriales	Cardiobacteriaceae	Cardiobacterium	uncultured Cardiobacterium sp.; S969; FJ976444
			Bacteria	Proteobacteria	Gammaproteobacteria	Cardiobacteriales	Cardiobacteriaceae	Cardiobacterium	Cardiobacterium valvarum; VA1941; DQ645464
o1367	o1367 16S-CatMor	AAAGCCTACCAAAGCGACGA	Bacteria	Firmicutes	Clostridia	Clostridiales	Lachnospiraceae	Catonella	uncultured Catonella sp.; 302E10(oral); AM420092
			Bacteria	Firmicutes	Clostridia	Clostridiales	Ruminococcaceae	unclassified_"Ruminococcaceae"	uncultured rumen bacterium; BF407; EU850587
			Bacteria	Firmicutes	Clostridia	Clostridiales	Lachnospiraceae	Catonella	Catonella morbi (T); ATCC 51271; X87151
			Bacteria	Firmicutes	Clostridia	Clostridiales	Lachnospiraceae	Catonella	Catonella genomosp. P1 oral clone MB5_P12; DQ003629
			Bacteria	Firmicutes	Clostridia	Clostridiales	Lachnospiraceae	unclassified_"Lachnospiraceae"	uncultured bacterium; WD11_aak22f07; EU509860
			Bacteria	Firmicutes	Clostridia	Clostridiales	Lachnospiraceae	unclassified_"Lachnospiraceae"	uncultured bacterium; WD11_aak22c09; EU509842
			Bacteria	Firmicutes	Clostridia	Clostridiales	Lachnospiraceae	unclassified_"Lachnospiraceae"	uncultured bacterium; WD11_aak22f01; EU509856
			Bacteria	Firmicutes	Clostridia	Clostridiales	Lachnospiraceae	Catonella	uncultured Catonella sp.; 18B968; FJ976374
			Bacteria	Firmicutes	Clostridia	Clostridiales	Lachnospiraceae	unclassified_"Lachnospiraceae"	uncultured bacterium; WD1_aaf15g03; EU510011
o1368	o1368 16S-CatOral	AACAATCTTAGTGGCGGACG	Bacteria	Firmicutes	Clostridia	Clostridiales	Lachnospiraceae	unclassified_"Lachnospiraceae"	Catonella sp. oral clone AH153; AF287769
			Bacteria	Firmicutes	Clostridia	Clostridiales	Ruminococcaceae	unclassified_"Ruminococcaceae"	uncultured bacterium; KO2_aai17f04; EU776406
o1369	o1369 16S-ClGroup1	CCTAGACTTGACATCTCCTG	Bacteria	Firmicutes	Clostridia	Clostridiales	Clostridiaceae	Clostridium	Clostridium beijerinckii; NCIMB9362; X68180
			Bacteria	Firmicutes	Clostridia	Clostridiales	Clostridiaceae	Clostridium	Clostridium botulinum; Eklund 202F ATCC23387; X68171
			Bacteria	Firmicutes	Clostridia	Clostridiales	Clostridiaceae	Clostridium	Clostridium botulinum; Eklund 17B ATCC25765; X68173
			Bacteria	Firmicutes	Clostridia	Clostridiales	Clostridiaceae	Clostridium	Clostridium cellulovorans (T); DSM 3052; X71849
			Bacteria	Firmicutes	Clostridia	Clostridiales	Clostridiaceae	Clostridium	Clostridium thermopalmarium (T); X72869
			Bacteria	Firmicutes	Clostridia	Clostridiales	Clostridiaceae	Clostridium	Clostridium thermobutyricum; X72868
			Bacteria	Firmicutes	Clostridia	Clostridiales	Clostridiaceae	Clostridium	Clostridium chartatabidum (T); DSM 5482; X71850
			Bacteria	Firmicutes	Clostridia	Clostridiales	Clostridiaceae	Clostridium	Clostridium tertium; DSM 2485; Y18174
			Bacteria	Firmicutes	Clostridia	Clostridiales	Clostridiaceae	Clostridium	Clostridium beijerinckii; JCM 7833; AB020187
			Bacteria	Firmicutes	Clostridia	Clostridiales	Clostridiaceae	Clostridium	Clostridium sartagoforme (T); DSM 1292; Y18175
			Bacteria	Firmicutes	Clostridia	Clostridiales	Clostridiaceae	Clostridium	uncultured bacterium; from human colonic sample; HuCB15; AJ408995
			Bacteria	Firmicutes	Clostridia	Clostridiales	Clostridiaceae	Clostridium	Clostridium haemolyticum; DSM 5565; Y18173
			Bacteria	Firmicutes	Clostridia	Clostridiales	Clostridiaceae	Clostridium	Clostridium novyi; ATCC27323; AB041865
			Bacteria	Firmicutes	Clostridia	Clostridiales	Clostridiaceae	Clostridium	Clostridium haemolyticum (T); ATCC9650; AB037910
			Bacteria	Firmicutes	Clostridia	Clostridiales	Clostridiaceae	Clostridium	Clostridium acetobutylicum; NCIMB 8052; X81021
			Bacteria	Firmicutes	Clostridia	Clostridiales	Clostridiaceae	Clostridium	Clostridium thermopalmarium; JW/JH-Fiji-1; AF286862
			Bacteria	Firmicutes	Clostridia	Clostridiales	Clostridiaceae	Clostridium	Clostridium sp. T7; AF281142
			Bacteria	Firmicutes	Clostridia	Clostridiales	Clostridiaceae	Clostridium	Clostridium beijerinckii; JCM 7847; AB020191
			Bacteria	Firmicutes	Clostridia	Clostridiales	Clostridiaceae	Clostridium	Clostridium sp. VeCb10; AJ229244
			Bacteria	Firmicutes	Clostridia	Clostridiales	Clostridiaceae	Clostridium	Clostridium acetobutylicum; NCIMB8052; X68182
o1370	o1370 16S-ClGroup2	AGATGGACCCGCGTCGCA	Bacteria	Firmicutes	Clostridia	Clostridiales	Clostridiaceae	Clostridium	Clostridium beijerinckii; NCIMB9362; X68180
			Bacteria	Firmicutes	Clostridia	Clostridiales	Clostridiaceae	Clostridium	Clostridium beijerinckii (T); DSM791; X68179
			Bacteria	Firmicutes	Clostridia	Clostridiales	Clostridiaceae	Clostridium	Clostridium butyricum; ATCC43755; X68176
			Bacteria	Firmicutes	Clostridia	Clostridiales	Clostridiaceae	Clostridium	Clostridium butyricum; DSM2478; X68177
			Bacteria	Firmicutes	Clostridia	Clostridiales	Clostridiaceae	Clostridium	Clostridium butyricum; NCIMB8082; X68178
			Bacteria	Firmicutes	Clostridia	Clostridiales	Clostridiaceae	Clostridium	Clostridium beijerinckii; JCM 7844; AB020189
			Bacteria	Firmicutes	Clostridia	Clostridiales	Clostridiaceae	Clostridium	Clostridium beijerinckii; JCM 7839; AB020188
			Bacteria	Firmicutes	Clostridia	Clostridiales	Clostridiaceae	Clostridium	Clostridium beijerinckii; JCM 7845; AB020190
			Bacteria	Firmicutes	Clostridia	Clostridiales	Clostridiaceae	Clostridium	Clostridium beijerinckii; JCM 7833; AB020187
			Bacteria	Firmicutes	Clostridia	Clostridiales	Clostridiaceae	Clostridium	Clostridium puniceum (T); DSM 2619; X71857
			Bacteria	Firmicutes	Clostridia	Clostridiales	Clostridiaceae	Clostridium	Clostridium acetobutylicum; NCIMB 8052; X81021
			Bacteria	Firmicutes	Clostridia	Clostridiales	Clostridiaceae	Clostridium	Clostridium sp.; RXyl1; Y15984
			Bacteria	Firmicutes	Clostridia	Clostridiales	Clostridiaceae	Clostridium	Clostridium butyricum; IBUN 22A; AJ289704
			Bacteria	Firmicutes	Clostridia	Clostridiales	Clostridiaceae	Clostridium	Clostridium butyricum; IBUN 125C; AJ289705
			Bacteria	Firmicutes	Clostridia	Clostridiales	Clostridiaceae	Clostridium	Clostridium butyricum; ATCC 19398; AB075768
			Bacteria	Firmicutes	Clostridia	Clostridiales	Clostridiaceae	Clostridium	Clostridium butyricum; strain MW8; AJ002592
			Bacteria	Firmicutes	Clostridia	Clostridiales	Clostridiaceae	Clostridium	Clostridium beijerinckii; JCM 7847; AB020191
			Bacteria	Firmicutes	Clostridia	Clostridiales	Clostridiaceae	Clostridium	Clostridium roseum (T); ATCC 17797; Y18171
			Bacteria	Firmicutes	Clostridia	Clostridiales	Clostridiaceae	Clostridium	Clostridium sp. VeCb10; AJ229244
			Bacteria	Firmicutes	Clostridia	Clostridiales	Clostridiaceae	Clostridium	Clostridium acetobutylicum; NCIMB8052; X68182
o1371	o1371 16S-ClGroup3	GCAACCTGCCTTGTAGAGG	Bacteria	Firmicutes	Clostridia	Clostridiales	Clostridiaceae	Clostridium	Clostridium chauvoei (T); ATCC 10092T; U51843
			Bacteria	Firmicutes	Clostridia	Clostridiales	Clostridiaceae	Clostridium	Clostridium septicum (T); ATCC 12464T; U59278
			Bacteria	Firmicutes	Clostridia	Clostridiales	Clostridiaceae	Clostridium	Clostridium tertium; DSM 2485; Y18174

			Bacteria	Firmicutes	Clostridia	Clostridiales	Clostridiaceae	Clostridium	Clostridium sp. 75064; AF227826
			Bacteria	Firmicutes	Clostridia	Clostridiales	Clostridiaceae	Clostridium	rumen bacterium R3_91_28; EU124834
			Bacteria	Firmicutes	Clostridia	Clostridiales	Clostridiaceae	unclassified_"Clostridiaceae 1"	Clostridium hveragerdense; AK14; EF088329
			Bacteria	Firmicutes	Clostridia	Clostridiales	Clostridiaceae	Clostridium	uncultured Clostridium sp.; N87; DQ232863
			Bacteria	Firmicutes	Clostridia	Clostridiales	Clostridiaceae	Clostridium	uncultured bacterium; aab20a12; DQ816593
			Bacteria	Firmicutes	Clostridia	Clostridiales	Clostridiaceae	Clostridium	Clostridium sp. BL-24; DQ196631
			Bacteria	Firmicutes	Clostridia	Clostridiales	Clostridiaceae	Clostridium	uncultured bacterium; aab20c10; DQ816613
			Bacteria	Firmicutes	Clostridia	Clostridiales	Clostridiaceae	Clostridium	Clostridium tertium (T); ATCC 14573; AJ245413
			Bacteria	Firmicutes	Clostridia	Clostridiales	Clostridiaceae	Clostridium	Clostridium carnis (T); M59091
			Bacteria	Firmicutes	Clostridia	Clostridiales	Ruminococcaceae	Ruminococcus	uncultured rumen bacterium; T20H60F57; AB269990
			Bacteria	Firmicutes	Clostridia	Clostridiales	Clostridiaceae	Clostridium	Clostridium septicum; CNM1300_06; EF194080
			Bacteria	Firmicutes	Clostridia	Clostridiales	Clostridiaceae	Clostridium	Clostridium chauvoei; CC5; EU106371
			Bacteria	Firmicutes	Clostridia	Clostridiales	Clostridiaceae	Clostridium	Clostridium chauvoei; CC6; EU106372
			Bacteria	Firmicutes	Clostridia	Clostridiales	Clostridiaceae	Clostridium	Clostridium chauvoei; CC1; EU081297
			Bacteria	Firmicutes	Clostridia	Clostridiales	Clostridiaceae	Clostridium	Clostridium chauvoei; CC2; EU106368
			Bacteria	Firmicutes	Clostridia	Clostridiales	Clostridiaceae	Clostridium	Clostridium chauvoei; CC4; EU106370
			Bacteria	Firmicutes	Clostridia	Clostridiales	Clostridiaceae	Clostridium	uncultured bacterium; Cadhufec45ml; AY850532
o1372	o1372 16S-CloLit	ACCTTTCGCATGGAAGGATC	Bacteria	Firmicutes	Clostridia	Clostridiales	Peptostreptococcaceae	Peptostreptococcaceae Incertae Sedis	Clostridium litorale (T); DSM 5388; X77845
			Bacteria	Firmicutes	Clostridia	Clostridiales	Peptostreptococcaceae	Peptostreptococcaceae Incertae Sedis	uncultured bacterium; CK_2C4_59; EU488348
			Bacteria	Firmicutes	Clostridia	Clostridiales	Peptostreptococcaceae	Peptostreptococcaceae Incertae Sedis	uncultured bacterium; CK_2C4_71; EU488360
			Bacteria	Firmicutes	Clostridia	Clostridiales	Peptostreptococcaceae	Peptostreptococcaceae Incertae Sedis	uncultured bacterium; CK_2C4_84; EU488373
			Bacteria	Firmicutes	Clostridia	Clostridiales	Peptostreptococcaceae	Peptostreptococcaceae Incertae Sedis	uncultured bacterium; CK_2C4_108; EU488397
			Bacteria	Firmicutes	Clostridia	Clostridiales	Lachnospiraceae	unclassified_"Lachnospiraceae"	uncultured bacterium; CATTLE_28; AY854290
o1374	o1374 16S-Corbac1	AGCCGTAAACGATGGACGC	Bacteria	unclassified_Bacteria					uncultured soil bacterium; HS9-75; AY221615
			Bacteria	Bacteroidetes	unclassified_Bacteroidetes				uncultured rumen bacterium; BF24; AY244902
			Bacteria	Actinobacteria	Actinobacteria	Coriobacteriales	Coriobacteriaceae	Collinsella	Collinsella aerofaciens; JCM7790; AB011814
			Bacteria	Actinobacteria	Actinobacteria	Coriobacteriales	Coriobacteriaceae	Collinsella	Collinsella aerofaciens; JCM7791; AB011815
			Bacteria	Actinobacteria	Actinobacteria	Coriobacteriales	Coriobacteriaceae	Collinsella	Collinsella aerofaciens (T); JCM10188; AB011816
			Bacteria	Actinobacteria	Actinobacteria	Coriobacteriales	Coriobacteriaceae	Collinsella	Collinsella intestinalis; RCA56-80; AB037383
			Bacteria	Actinobacteria	Actinobacteria	Coriobacteriales	Coriobacteriaceae	Collinsella	Collinsella stercoris; RCA55-4; AB031059
			Bacteria	Actinobacteria	Actinobacteria	Coriobacteriales	Coriobacteriaceae	Collinsella	Collinsella intestinalis (T); RCA56-68; AB031063
			Bacteria	Actinobacteria	Actinobacteria	Coriobacteriales	Coriobacteriaceae	Collinsella	Coriobacterium sp. CCUG 33918; AJ131150
			Bacteria	Actinobacteria	Actinobacteria	Coriobacteriales	Coriobacteriaceae	Collinsella	Coriobacterium sp. CCUG 33917; AJ131149
			Bacteria	Actinobacteria	Actinobacteria	Coriobacteriales	Coriobacteriaceae	Collinsella	Collinsella stercoris (T); RCA55-54; AB031061
			Bacteria	Actinobacteria	Actinobacteria	Coriobacteriales	Coriobacteriaceae	Collinsella	Collinsella stercoris; RCA55-5; AB037381
			Bacteria	Actinobacteria	Actinobacteria	Coriobacteriales	Coriobacteriaceae	Collinsella	Collinsella stercoris; RCA55-34; AB031060
			Bacteria	Actinobacteria	Actinobacteria	Coriobacteriales	Coriobacteriaceae	Collinsella	Collinsella stercoris; RCA55-27; AB037382
			Bacteria	Actinobacteria	Actinobacteria	Coriobacteriales	Coriobacteriaceae	Collinsella	Coriobacterium sp. EKSO3; AJ245921
			Bacteria	Actinobacteria	Actinobacteria	Coriobacteriales	Coriobacteriaceae	Collinsella	Collinsella stercoris; RCA55-49; AB037384
			Bacteria	Actinobacteria	Actinobacteria	Coriobacteriales	Coriobacteriaceae	Collinsella	Collinsella aerofaciens; G118; AJ245919
			Bacteria	Actinobacteria	Actinobacteria	Coriobacteriales	Coriobacteriaceae	Collinsella	Collinsella stercoris; RCA55-58; AB037385
			Bacteria	Actinobacteria	Actinobacteria	Coriobacteriales	Coriobacteriaceae	Collinsella	Collinsella aerofaciens; H818; AJ245920
			Bacteria	Actinobacteria	Actinobacteria	Coriobacteriales	Coriobacteriaceae	Coriobacterium	Coriobacterium glomerans (T); DSM 20642; X79048
o1375	o1375 16S-Corbac2	TCACACCACCCGAGTCGT	Bacteria	unclassified_Bacteria					uncultured spirochete; LF045; AY605161
			Bacteria	Actinobacteria	Actinobacteria	Coriobacteriales	Coriobacteriaceae	Atopobium	Atopobium fossor; JCM 9981; AB015945
			Bacteria	Actinobacteria	Actinobacteria	Coriobacteriales	Coriobacteriaceae	Atopobium	Atopobium minutum (T); M59059
			Bacteria	Actinobacteria	Actinobacteria	Coriobacteriales	Coriobacteriaceae	Collinsella	Collinsella aerofaciens; JCM7790; AB011814
			Bacteria	Actinobacteria	Actinobacteria	Coriobacteriales	Coriobacteriaceae	Collinsella	Collinsella aerofaciens; JCM7791; AB011815
			Bacteria	Actinobacteria	Actinobacteria	Coriobacteriales	Coriobacteriaceae	Collinsella	Collinsella aerofaciens (T); JCM10188; AB011816
			Bacteria	Actinobacteria	Actinobacteria	Coriobacteriales	Coriobacteriaceae	Collinsella	Collinsella intestinalis; RCA56-80; AB037383
			Bacteria	Actinobacteria	Actinobacteria	Coriobacteriales	Coriobacteriaceae	Collinsella	Collinsella intestinalis (T); RCA56-68; AB031063
			Bacteria	Actinobacteria	Actinobacteria	Coriobacteriales	Coriobacteriaceae	Collinsella	Coriobacterium sp. CCUG 33918; AJ131150
			Bacteria	Actinobacteria	Actinobacteria	Coriobacteriales	Coriobacteriaceae	Collinsella	Coriobacterium sp. CCUG 33917; AJ131149
			Bacteria	Actinobacteria	Actinobacteria	Coriobacteriales	Coriobacteriaceae	Collinsella	Collinsella stercoris (T); RCA55-54; AB031061
			Bacteria	Actinobacteria	Actinobacteria	Coriobacteriales	Coriobacteriaceae	Collinsella	Collinsella stercoris; RCA55-5; AB037381
			Bacteria	Actinobacteria	Actinobacteria	Coriobacteriales	Coriobacteriaceae	Collinsella	Collinsella stercoris; RCA55-27; AB037382
			Bacteria	Actinobacteria	Actinobacteria	Coriobacteriales	Coriobacteriaceae	Collinsella	Coriobacterium sp. EKSO3; AJ245921
			Bacteria	Actinobacteria	Actinobacteria	Coriobacteriales	Coriobacteriaceae	Collinsella	Collinsella stercoris; RCA55-49; AB037384
			Bacteria	Actinobacteria	Actinobacteria	Coriobacteriales	Coriobacteriaceae	Collinsella	Collinsella aerofaciens; G118; AJ245919
			Bacteria	Actinobacteria	Actinobacteria	Coriobacteriales	Coriobacteriaceae	Cryptobacterium	Cryptobacterium curtum (T); ATCC700683; 12-3; AB019260
			Bacteria	Actinobacteria	Actinobacteria	Coriobacteriales	Coriobacteriaceae	Collinsella	Collinsella stercoris; RCA55-58; AB037385
			Bacteria	Actinobacteria	Actinobacteria	Coriobacteriales	Coriobacteriaceae	Collinsella	Collinsella aerofaciens; H818; AJ245920
			Bacteria	Actinobacteria	Actinobacteria	Coriobacteriales	Coriobacteriaceae	Coriobacterium	Coriobacterium glomerans (T); DSM 20642; X79048
o1376	o1376 16S-CorDur	AAAGCCTTATTGCGGTGTGG	Bacteria	Actinobacteria	Actinobacteria	Actinomycetales	Corynebacteriaceae	Corynebacterium	Corynebacterium durum (T); IBS G15036; Z97069
o1377	o1377 16S-CorExi	AATACGCTTTGCCTTGACGG	Bacteria	Proteobacteria	Deltaproteobacteria	Myxococcales	Myxococcaceae	Corallococcus	Corallococcus coralloides; DSM 52499; AJ811603
			Bacteria	Proteobacteria	Deltaproteobacteria	Myxococcales	Myxococcaceae	Corallococcus	Corallococcus coralloides (T); type strain: DSM 2259; AJ811588
			Bacteria	Proteobacteria	Deltaproteobacteria	Myxococcales	Myxococcaceae	Corallococcus	Corallococcus coralloides; DSM 52497; AJ811597
			Bacteria	Proteobacteria	Deltaproteobacteria	Myxococcales	Myxococcaceae	Corallococcus	Corallococcus coralloides; DSM 51492; AJ811601
			Bacteria	Proteobacteria	Deltaproteobacteria	Myxococcales	Myxococcaceae	Corallococcus	Corallococcus coralloides; DSM 51558; AJ811602
			Bacteria	Proteobacteria	Deltaproteobacteria	Myxococcales	Myxococcaceae	Corallococcus	Corallococcus coralloides; DSM 52502; AJ811604
			Bacteria	Proteobacteria	Deltaproteobacteria	Myxococcales	Myxococcaceae	Corallococcus	Corallococcus coralloides; DSM 52503; AJ811605
			Bacteria	Proteobacteria	Deltaproteobacteria	Myxococcales	Myxococcaceae	Corallococcus	Corallococcus coralloides; DSM 51425; AJ811600
			Bacteria	Proteobacteria	Deltaproteobacteria	Myxococcales	Myxococcaceae	Corallococcus	Corallococcus coralloides; DSM 51359; AJ811589
			Bacteria	Proteobacteria	Deltaproteobacteria	Myxococcales	Myxococcaceae	Corallococcus	Corallococcus coralloides; DSM 51548; AJ811593
			Bacteria	Proteobacteria	Deltaproteobacteria	Myxococcales	Myxococcaceae	Corallococcus	Corallococcus exiguus; Cc e100; AJ233932
			Bacteria	Proteobacteria	Deltaproteobacteria	Myxococcales	Myxococcaceae	Corallococcus	Corallococcus coralloides; DSM 51547; AJ811592
			Bacteria	Proteobacteria	Deltaproteobacteria	Myxococcales	Myxococcaceae	Corallococcus	Corallococcus coralloides; DSM 51557; AJ811594
			Bacteria	Proteobacteria	Deltaproteobacteria	Myxococcales	Myxococcaceae	Corallococcus	Corallococcus coralloides; DSM 51651; AJ811595
			Bacteria	Proteobacteria	Deltaproteobacteria	Myxococcales	Myxococcaceae	Corallococcus	Corallococcus exiguus (T); type strain: DSM 14696; AJ811598
			Bacteria	Proteobacteria	Deltaproteobacteria	Myxococcales	Myxococcaceae	Corallococcus	Corallococcus coralloides; DSM 51307; AJ811599
			Bacteria	Proteobacteria	Deltaproteobacteria	Myxococcales	Myxococcaceae	Corallococcus	Corallococcus coralloides; DSM 51427; AJ811591
			Bacteria	Proteobacteria	Deltaproteobacteria	Myxococcales	Myxococcaceae	Corallococcus	Corallococcus coralloides; DSM 51379; AJ811590
			Bacteria	Proteobacteria	Deltaproteobacteria	Myxococcales	Myxococcaceae	Corallococcus	Corallococcus coralloides; DSM 51682; AJ811596
			Bacteria	Proteobacteria	Deltaproteobacteria	Myxococcales	Myxococcaceae	Corallococcus	Corallococcus exiguus; Cc e12b; AJ233931
o1378	o1378 16S-CorOral	AACTCCTTTCGTTAGGGACG	Bacteria	Actinobacteria	Actinobacteria	Actinomycetales	Corynebacteriaceae	Corynebacterium	Corynebacterium matruchotii; ChDC OS35; AF543287
			Bacteria	Actinobacteria	Actinobacteria	Actinomycetales	Corynebacteriaceae	Corynebacterium	Corynebacterium matruchotii; ChDC OS36; AF543288
			Bacteria	Actinobacteria	Actinobacteria	Actinomycetales	Corynebacteriaceae	Corynebacterium	uncultured Corynebacterium sp.; 201F03(oral); AM420027
			Bacteria	Actinobacteria	Actinobacteria	Actinomycetales	Corynebacteriaceae	Corynebacterium	uncultured Corynebacterium sp.; 201C03(oral); AM420019

			Bacteria	Actinobacteria	Actinobacteria	Actinomycetales	Corynebacteriaceae	Corynebacterium	uncultured Corynebacterium sp.; 601B03(oral); AM420211
			Bacteria	Actinobacteria	Actinobacteria	Actinomycetales	Corynebacteriaceae	Corynebacterium	uncultured bacterium; A_D_01_43; FJ470423
			Bacteria	Actinobacteria	Actinobacteria	Actinomycetales	Corynebacteriaceae	Corynebacterium	uncultured bacterium; MA04A05; FM873748
			Bacteria	Actinobacteria	Actinobacteria	Actinomycetales	Corynebacteriaceae	Corynebacterium	uncultured bacterium; MA03B12; FM873706
			Bacteria	Actinobacteria	Actinobacteria	Actinomycetales	Corynebacteriaceae	Corynebacterium	uncultured bacterium; MA03B02; FM873549
			Bacteria	Actinobacteria	Actinobacteria	Actinomycetales	Corynebacteriaceae	Corynebacterium	Corynebacterium sp. oral clone AK153; AF287755
			Bacteria	Actinobacteria	Actinobacteria	Actinomycetales	Corynebacteriaceae	Corynebacterium	uncultured Corynebacterium sp.; 8837-D0-A-8A; EU289120
			Bacteria	Actinobacteria	Actinobacteria	Actinomycetales	Corynebacteriaceae	Corynebacterium	uncultured Corynebacterium sp.; 8837-D0-C-2A; EU289039
			Bacteria	Actinobacteria	Actinobacteria	Actinomycetales	Corynebacteriaceae	Corynebacterium	uncultured bacterium; nbt63g07; EU536613
			Bacteria	Actinobacteria	Actinobacteria	Actinomycetales	Corynebacteriaceae	Corynebacterium	uncultured bacterium; nbt63c05; EU536580
			Bacteria	Actinobacteria	Actinobacteria	Actinomycetales	Corynebacteriaceae	Corynebacterium	uncultured bacterium; nbu197b04c1; GQ002575
			Bacteria	Actinobacteria	Actinobacteria	Actinomycetales	Corynebacteriaceae	Corynebacterium	uncultured bacterium; nbw793d10c1; GQ011554
			Bacteria	Actinobacteria	Actinobacteria	Actinomycetales	Corynebacteriaceae	Corynebacterium	uncultured bacterium; nbw795a09c1; GQ011666
			Bacteria	Actinobacteria	Actinobacteria	Actinomycetales	Corynebacteriaceae	Corynebacterium	uncultured bacterium; nbu94e01c1; GQ005594
			Bacteria	Actinobacteria	Actinobacteria	Actinomycetales	Corynebacteriaceae	Corynebacterium	uncultured bacterium; nbu83e02c1; GQ006433
			Bacteria	Actinobacteria	Actinobacteria	Actinomycetales	Corynebacteriaceae	Corynebacterium	Corynebacterium matruchotii (T); CIP 81.82T; X82065
o1380	o1380 16S-Crearc	CTGGTGTCAGCCGCCGC	Archaea	Crenarchaeota	Thermoprotei	Thermoproteales	Thermoproteaceae	Pyrobaculum	Pyrobaculum aerophilum (T); im2; L07510
			Archaea	Crenarchaeota	Thermoprotei	Thermoproteales	Thermoproteaceae	Thermocladium	Thermocladium modestius (T); IC-125; AB005296
			Archaea	Crenarchaeota	Thermoprotei	Thermoproteales	Thermoproteaceae	Pyrobaculum	Thermoproteus tenax; M35966
			Archaea	Crenarchaeota	Thermoprotei	Desulfurococcales	Desulfurococcaceae	Acidilobus	Caldococcus noboribetus; NC12; D85038
			Archaea	Crenarchaeota	Thermoprotei	Thermoproteales	Thermoproteaceae	Pyrobaculum	Pyrobaculum calidifontis; VA1; AB078332
			Archaea	Crenarchaeota	Thermoprotei	Desulfurococcales	Desulfurococcaceae	Staphylothermus	Staphylothermus marinus (T); F1 - DSM 3639; X99560
			Archaea	Crenarchaeota	Thermoprotei	Desulfurococcales	Desulfurococcaceae	Ignicoccus	Ignicoccus islandicus (T); Ko18; X99562
			Archaea	Crenarchaeota	Thermoprotei	Desulfurococcales	Desulfurococcaceae	Thermosphaera	Thermosphaera aggregans (T); M11TL; X99556
			Archaea	Crenarchaeota	Thermoprotei	Thermoproteales	Thermoproteaceae	Pyrobaculum	Pyrobaculum oguniense (T); TE7; AB029339
			Archaea	Crenarchaeota	Thermoprotei	Thermoproteales	Thermoproteaceae	Pyrobaculum	Pyrobaculum oguniense; TE7; AB087402
			Archaea	Crenarchaeota	Thermoprotei	Thermoproteales	Thermoproteaceae	Pyrobaculum	Pyrobaculum neutrophilum; NZ1; X81886
			Archaea	Crenarchaeota	Thermoprotei	Desulfurococcales	Desulfurococcaceae	Staphylothermus	Staphylothermus hellenicus (T); P8(BK20S6-10-b1); AJ012645
			Archaea	Crenarchaeota	Thermoprotei	Thermoproteales	Thermoproteaceae	Pyrobaculum	uncultured crenarchaeote pBA2; AF176346
			Archaea	Crenarchaeota	Thermoprotei	Thermoproteales	Thermoproteaceae	Pyrobaculum	Pyrobaculum islandicum (T); geo3; L07511
			Archaea	Crenarchaeota	Thermoprotei	Desulfurococcales	Desulfurococcaceae	Thermodiscus	Thermodiscus maritimus (T); S2; X99554
			Archaea	Crenarchaeota	Thermoprotei	Desulfurococcales	Desulfurococcaceae	Aeropyrum	Aeropyrum camini (T); SY1; AB109559
			Archaea	Crenarchaeota	Thermoprotei	Thermoproteales	Thermoproteaceae	Pyrobaculum	Pyrobaculum arsenaticum (T); PZ6; AJ277124
			Archaea	Crenarchaeota	Thermoprotei	Desulfurococcales	Desulfurococcaceae	Ignicoccus	Ignicoccus pacificus (T); LPC33; AJ271794
			Archaea	Crenarchaeota	Thermoprotei	Thermoproteales	Thermoproteaceae	Pyrobaculum	Pyrobaculum sp. WIJ3; AJ277125
			Archaea	Crenarchaeota	Thermoprotei	Desulfurococcales	Desulfurococcaceae	Acidilobus	Acidilobus aceticus (T); Z-1904; AF191225
o1381	o1381 16S-Cyabac	CCCCAGTAGTCCTAGCCG	Bacteria	Cyanobacteria	Cyanobacteria		Family I	GpI	Nodularia sp. BCNOD9427; AJ224447
			Bacteria	Cyanobacteria	Cyanobacteria		Family I	GpI	Aphanizomenon sp. 'BC-Aph 9601'; AJ245457
			Bacteria	Cyanobacteria	Cyanobacteria		Family I	GpI	Anabaena sp. 299; AJ293106
			Bacteria	Cyanobacteria	Cyanobacteria		Family I	GpI	Anabaena lemmermannii; 202A2; AJ293104
			Bacteria	Cyanobacteria	Cyanobacteria		Family I	GpI	Aphanizomenon flos-aquae; PMC9706; AJ293129
			Bacteria	Cyanobacteria	Cyanobacteria		Family I	GpI	Anabaena compacta; 118; AJ293109
			Bacteria	Cyanobacteria	Cyanobacteria		Family I	GpI	Aphanizomenon flos-aquae var. klebahnii; 218; AJ293123
			Bacteria	Cyanobacteria	Cyanobacteria		Family I	GpI	Anabaena compacta; 189; AJ293111
			Bacteria	Cyanobacteria	Cyanobacteria		Family I	GpI	Aphanizomenon flos-aquae; PMC9707; AJ293130
			Bacteria	Cyanobacteria	Cyanobacteria		Family I	GpI	Chlorogloeopsis sp. PCC 7518; PCC7518; I-15; X68780
			Bacteria	Cyanobacteria	Cyanobacteria		Family I	GpI	Anabaena planctonica; 71; AJ293108
			Bacteria	Cyanobacteria	Cyanobacteria		Family I	GpI	Anabaena spiroides; PMC9403; AJ293116
			Bacteria	Cyanobacteria	Cyanobacteria		Family I	GpI	Anabaena cf. scheremetievi PMC9701; AJ293117
			Bacteria	Cyanobacteria	Cyanobacteria		Family I	GpI	Anabaena crassa; 215; AJ293112
			Bacteria	Cyanobacteria	Cyanobacteria		Family I	GpI	Anabaena spiroides; PMC9702; AJ293118
			Bacteria	Cyanobacteria	Cyanobacteria		Family I	GpI	Fischerella muscicola; PCC 7414; AB039003
			Bacteria	Cyanobacteria	Cyanobacteria	unclassified_Cyanobacteria			Chroococcidiopsis thermalis (T); PCC 7203; AB039005
			Bacteria	Cyanobacteria	Cyanobacteria		Family I	GpI	Nodularia spumigena (T); PCC 73104; AB039002
			Bacteria	Cyanobacteria	Cyanobacteria		Family I	GpI	uncultured cyanobacterium; watersample from Lake Loosdrecht; LD8; AJ006281
			Bacteria	Cyanobacteria	Cyanobacteria		Family I	GpI	Anabaena azotica; type strain: Ley HB686 = HBAS 44; AJ488603
o1382	o1382 16S-DefThe	AAAGGTCCAGGCAGTAACTG	Bacteria	Deferribacteres	Deferribacteres	Deferribacterales	Deferribacteraceae	Deferribacter	Deferribacter thermophilus (T); BMA1; U75602
			Bacteria	Deferribacteres	Deferribacteres	Deferribacterales	Deferribacteraceae	Deferribacter	uncultured bacterium; Hda_Bac26; FN356326
			Bacteria	Deferribacteres	Deferribacteres	Deferribacterales	Deferribacteraceae	Deferribacter	uncultured bacterium; NAK1-b1; DQ867052
o1383	o1383 16S-DerOral	AACCCAACGCTTAACGTTGG	Bacteria	Actinobacteria	Actinobacteria	Actinomycetales	Intrasporangiaceae	Arsenicicoccus	Dermabacter sp. oral strain B46KS; AF287753
o1384	o1384 16S-DesGroup	TGGGTTTGACATCTGGGGAA	Bacteria	Proteobacteria	Deltaproteobacteria	Desulfovibrionales	Desulfovibrionaceae	Desulfovibrio	uncultured bacterium; SJTU_D_16_36; EF401883
			Bacteria	Proteobacteria	Deltaproteobacteria	unclassified_Deltaproteobacteria			uncultured bacterium; 115G73; EU925894
			Bacteria	Proteobacteria	Deltaproteobacteria	unclassified_Deltaproteobacteria			uncultured bacterium; EPR3965-I2-Bc72; EU491876
			Bacteria	Proteobacteria	Deltaproteobacteria	unclassified_Deltaproteobacteria			uncultured bacterium; 044C49; EU734952
			Bacteria	Proteobacteria	Deltaproteobacteria	unclassified_Deltaproteobacteria			uncultured bacterium; 042C45; EU734950
			Bacteria	Proteobacteria	Deltaproteobacteria	Desulfovibrionales	Desulfovibrionaceae	Desulfovibrio	bacterium 'New Zealand D'; AY721628
			Bacteria	Proteobacteria	Deltaproteobacteria	Desulfovibrionales	Desulfovibrionaceae	Desulfovibrio	uncultured Shigella sp.; ABXD_AD4; FJ440030
			Bacteria	Proteobacteria	Deltaproteobacteria	unclassified_Deltaproteobacteria			uncultured bacterium; G3DCM-20; EU037315
			Bacteria	Proteobacteria	Deltaproteobacteria	Desulfovibrionales	Desulfovibrionaceae	Desulfovibrio	uncultured Desulfovibrio sp.; BF252; EU850487
			Bacteria	Proteobacteria	Deltaproteobacteria	Desulfovibrionales	Desulfovibrionaceae	Desulfovibrio	Desulfovibrio sp. ABHU2SB; AF056090
			Bacteria	Proteobacteria	Deltaproteobacteria	Desulfovibrionales	Desulfovibrionaceae	Desulfovibrio	Desulfovibrio sp. ABHU1SBfatS; AF056089
			Bacteria	unclassified_Bacteria					unidentified bacterium wb1_N15; AF317771
			Bacteria	Proteobacteria	Deltaproteobacteria	Desulfovibrionales	Desulfovibrionaceae	Desulfovibrio	Desulfovibrio sp. ABHU1SB; AF056088
			Bacteria	Proteobacteria	Deltaproteobacteria	unclassified_Deltaproteobacteria			uncultured bacterium; G2DMC-155; DQ899888
			Bacteria	Proteobacteria	Deltaproteobacteria	Desulfovibrionales	Desulfovibrionaceae	Desulfovibrio	Desulfovibrio sp. LNB1; AY554145
			Bacteria	Proteobacteria	Deltaproteobacteria	Desulfovibrionales	Desulfovibrionaceae	Desulfovibrio	uncultured bacterium; TS51_a04b08; FJ369732
			Bacteria	Proteobacteria	Deltaproteobacteria	unclassified_Deltaproteobacteria			uncultured Myxococcales bacterium; AMGG8; AM935423
			Bacteria	Proteobacteria	Deltaproteobacteria	Desulfovibrionales	Desulfovibrionaceae	Desulfovibrio	Desulfovibrio sp. oral clone BB161; AY005035
			Bacteria	Proteobacteria	Deltaproteobacteria	unclassified_Deltaproteobacteria			uncultured bacterium; OPPB016; AY861759
			Bacteria	Proteobacteria	Deltaproteobacteria	unclassified_Deltaproteobacteria			uncultured bacterium; OPPB030; AY861773
o1385	o1385 16S-DesOra	AACTGCACTTGAAACTGCGG	Bacteria	Proteobacteria	Deltaproteobacteria	Desulfovibrionales	Desulfomicrobiaceae	Desulfomicrobium	Desulfomicrobium orale; NY676; AJ251628
			Bacteria	Proteobacteria	Deltaproteobacteria	Desulfovibrionales	Desulfomicrobiaceae	Desulfomicrobium	Desulfomicrobium orale; NY679; AJ251624
			Bacteria	Proteobacteria	Deltaproteobacteria	Desulfovibrionales	Desulfomicrobiaceae	Desulfomicrobium	Desulfomicrobium orale (T); DSM12838; AJ251623
			Bacteria	Proteobacteria	Deltaproteobacteria	Desulfovibrionales	Desulfomicrobiaceae	Desulfomicrobium	Desulfomicrobium orale; NY683; AJ251627
			Bacteria	Proteobacteria	Deltaproteobacteria	Desulfovibrionales	Desulfomicrobiaceae	Desulfomicrobium	Desulfomicrobium orale; NY677; AJ251629
			Bacteria	Proteobacteria	Deltaproteobacteria	Desulfovibrionales	Desulfomicrobiaceae	Desulfomicrobium	uncultured delta proteobacterium; P1-1; AY083022
			Bacteria	Proteobacteria	Deltaproteobacteria	Desulfovibrionales	Desulfomicrobiaceae	Desulfomicrobium	uncultured delta proteobacterium; P4-16; AY083023
			Bacteria	Proteobacteria	Deltaproteobacteria	Desulfovibrionales	Desulfomicrobiaceae	Desulfomicrobium	uncultured delta proteobacterium; P4-14; AY083019

			Bacteria	Proteobacteria	Deltaproteobacteria	Desulfovibrionales	Desulfomicrobiaceae	Desulfomicrobium	uncultured delta proteobacterium; P4-15; AY083020
			Bacteria	Proteobacteria	Deltaproteobacteria	Desulfovibrionales	Desulfomicrobiaceae	Desulfomicrobium	uncultured delta proteobacterium; P4-13; AY083018
			Bacteria	Proteobacteria	Deltaproteobacteria	Desulfovibrionales	Desulfomicrobiaceae	Desulfomicrobium	uncultured delta proteobacterium; P4-17; AY083021
			Bacteria	Proteobacteria	Deltaproteobacteria	Desulfobacterales	Desulfobulbaceae	Desulfobulbus	Desulfobulbus sp. oral clone CH031; AY005036
o1386	o1386 16S-DesOral	AAAGTGGGTGGTGCCTTCTT	Bacteria	Proteobacteria	Deltaproteobacteria	Desulfobacterales	Desulfobulbaceae	Desulfobulbus	uncultured Desulfobulbus sp.; VHS-B4-9; DQ394978
			Bacteria	Proteobacteria	Deltaproteobacteria	Desulfobacterales	Desulfobulbaceae	Desulfobulbus	Desulfobulbus sp. oral clone R004; AY005037
			Bacteria	Firmicutes	Clostridia	Clostridiales	Veillonellaceae	Dialister	Dialister pneumosintes; SC3D; 3D-5; Z36297
o1387	o1387 16S-DiaGroup	CAGACGGAAACGACTGCTAA	Bacteria	Firmicutes	Clostridia	Clostridiales	Veillonellaceae	Dialister	Dialister sp. oral clone BS095; AF287787
			Bacteria	Firmicutes	Clostridia	Clostridiales	Veillonellaceae	Dialister	Allisonella histaminiformans (T); MR2; AF548373
			Bacteria	Firmicutes	Clostridia	Clostridiales	Veillonellaceae	Dialister	Dialister invisus DSM 15470 (T); E7.25; AY162469
			Bacteria	Firmicutes	Clostridia	Clostridiales	Veillonellaceae	Dialister	Dialister sp. oral clone MCE7_134; AF481210
			Bacteria	Firmicutes	Clostridia	Clostridiales	Veillonellaceae	Dialister	Dialister pneumosintes (T); ATCC 33048; X82500
			Bacteria	Firmicutes	Clostridia	Clostridiales	Veillonellaceae	Dialister	uncultured bacterium; p-1941-s962-3; AF371694
			Bacteria	Firmicutes	Clostridia	Clostridiales	Veillonellaceae	Dialister	Dialister pneumosintes; SC3D; 3D-5; U13040
			Bacteria	Firmicutes	Clostridia	Clostridiales	Veillonellaceae	Dialister	uncultured bacterium; p-1740-b3; AF371695
			Bacteria	Firmicutes	Clostridia	Clostridiales	Veillonellaceae	Dialister	uncultured bacterium; p-922-s962-5; AF371693
			Bacteria	Firmicutes	Clostridia	Clostridiales	Veillonellaceae	Dialister	Dialister sp. GBA27; AF287788
			Bacteria	Firmicutes	Clostridia	Clostridiales	Veillonellaceae	Dialister	Dialister sp. oral clone BS016; AF287786
			Bacteria	Firmicutes	Clostridia	Clostridiales	Veillonellaceae	Dialister	Dialister micraerophilus (T); ADV 04.01; AF473837
			Bacteria	Firmicutes	Clostridia	Clostridiales	Veillonellaceae	Dialister	Dialister micraerophilus; ADV 2470.02; AY850109
			Bacteria	Firmicutes	Clostridia	Clostridiales	Veillonellaceae	Dialister	Dialister micraerophilus; ADV 253.01; AY850107
			Bacteria	Firmicutes	Clostridia	Clostridiales	Veillonellaceae	Dialister	Dialister micraerophilus; ADV 1368.02; AY850108
			Bacteria	Firmicutes	Clostridia	Clostridiales	Veillonellaceae	Dialister	Dialister micraerophilus; ADV 1413.03; AY850112
			Bacteria	Firmicutes	Clostridia	Clostridiales	Veillonellaceae	Dialister	Dialister micraerophilus; ADV 3269.02; AY850110
			Bacteria	Firmicutes	Clostridia	Clostridiales	Veillonellaceae	Dialister	Dialister micraerophilus; ADV 5290.03; AY850113
			Bacteria	Firmicutes	Clostridia	Clostridiales	Veillonellaceae	Dialister	uncultured bacterium; 178k07; AJ812140
			Bacteria	Firmicutes	Clostridia	Clostridiales	Veillonellaceae	Dialister	Dialister pneumosintes; SC3D; 3D-5; Z36297
o1388	o1388 16S-DiaPne	AAAGGGTGGCCTCTACAAGA	Bacteria	Firmicutes	Clostridia	Clostridiales	Veillonellaceae	Dialister	uncultured Dialister sp.; 201E08(oral); AM420012
			Bacteria	Firmicutes	Clostridia	Clostridiales	Veillonellaceae	Dialister	Dialister pneumosintes (T); ATCC 33048; X82500
			Bacteria	Firmicutes	Clostridia	Clostridiales	Veillonellaceae	Dialister	Dialister pneumosintes; SC3D; 3D-5; U13040
			Bacteria	Firmicutes	Clostridia	Clostridiales	Veillonellaceae	Dialister	uncultured bacterium; 014B-E12; DQ905593
			Bacteria	Firmicutes	Clostridia	Clostridiales	Veillonellaceae	Dialister	uncultured bacterium; nbu86e12c1; GQ006699
			Bacteria	Firmicutes	Clostridia	Clostridiales	Veillonellaceae	Dialister	uncultured bacterium; nbu87e12c1; GQ006779
			Bacteria	Firmicutes	Clostridia	Clostridiales	Veillonellaceae	Dialister	uncultured bacterium; nbu85c10c1; GQ006590
			Bacteria	Firmicutes	Clostridia	Clostridiales	Veillonellaceae	Dialister	uncultured bacterium; nbu86f03c1; GQ006700
			Bacteria	Firmicutes	Bacilli	Lactobacillales	Enterococcaceae	Enterococcus	Enterococcus faecium; LMG 11423; AJ301830
o1389	o1389 16S-EcGroup2	GAAGTCGCGAGGCTAAGCT	Bacteria	Firmicutes	Bacilli	Lactobacillales	Enterococcaceae	Enterococcus	Enterococcus casseliflavus (T); LMG 13518; AJ301832
			Bacteria	Firmicutes	Bacilli	Lactobacillales	Enterococcaceae	Enterococcus	Enterococcus gallinarum; LMG 13129; AJ301833
			Bacteria	Firmicutes	Bacilli	Lactobacillales	Enterococcaceae	Enterococcus	Enterococcus pseudoavium; LMG 11426; AJ301837
			Bacteria	Firmicutes	Bacilli	Lactobacillales	Enterococcaceae	Enterococcus	Enterococcus hirae (T); DSM20160; AJ276356
			Bacteria	Firmicutes	Bacilli	Lactobacillales	Enterococcaceae	Enterococcus	Enterococcus faecium (T); DSM20477; AJ276355
			Bacteria	Firmicutes	Bacilli	Lactobacillales	Enterococcaceae	Enterococcus	Enterococcus durans; DSM20633; AJ276354
			Bacteria	Firmicutes	Bacilli	Lactobacillales	Enterococcaceae	Enterococcus	Enterococcus casseliflavus; AF039903
			Bacteria	Firmicutes	Bacilli	Lactobacillales	Enterococcaceae	Enterococcus	uncultured bacterium; AJ538350
			Bacteria	Firmicutes	Bacilli	Lactobacillales	Enterococcaceae	Enterococcus	Enterococcus malodoratus; NCFB 846T; Y18339
			Bacteria	Firmicutes	Bacilli	Lactobacillales	Enterococcaceae	Enterococcus	Enterococcus saccharolyticus; NCDO 2594T; Y18357
			Bacteria	Firmicutes	Bacilli	Lactobacillales	Enterococcaceae	Enterococcus	Enterococcus avium; NCFB 2369T; Y18274
			Bacteria	Firmicutes	Bacilli	Lactobacillales	Enterococcaceae	Enterococcus	Enterococcus casseliflavus; NCIMB 13226T; Y18295
			Bacteria	Firmicutes	Bacilli	Lactobacillales	Enterococcaceae	Enterococcus	Enterococcus raffinosus (T); NCIMB 12901T; Y18296
			Bacteria	Firmicutes	Bacilli	Lactobacillales	Enterococcaceae	Enterococcus	Enterococcus casseliflavus (T); NCIMB 11449; Y18161
			Bacteria	Firmicutes	Bacilli	Lactobacillales	Enterococcaceae	Enterococcus	Enterococcus pseudoavium; NCFB 2138T; Y18356
			Bacteria	Firmicutes	Bacilli	Lactobacillales	Enterococcaceae	Enterococcus	Enterococcus gallinarum; 42; AF039898
			Bacteria	Firmicutes	Bacilli	Lactobacillales	Enterococcaceae	Enterococcus	Enterococcus sulfureus (T); NCDO 2379 (= MUTK31); X55133
			Bacteria	Firmicutes	Bacilli	Lactobacillales	Enterococcaceae	Enterococcus	Enterococcus dispar; NCIMB 13000T; Y18358
			Bacteria	Firmicutes	Bacilli	Lactobacillales	Enterococcaceae	Enterococcus	Enterococcus sulfureus; NCIMB 13117T; Y18341
			Bacteria	Firmicutes	Bacilli	Lactobacillales	Enterococcaceae	Enterococcus	Enterococcus faecium; LMG 11423; AJ301830
			Bacteria	Firmicutes	Bacilli	Lactobacillales	Enterococcaceae	Enterococcus	Enterococcus faecalis; LMG 7937; AJ301831
o1390	o1390 16S-EcGroup3	GCTTCTCTCAGTTCGGATTG	Bacteria	Firmicutes	Bacilli	Lactobacillales	Enterococcaceae	Enterococcus	Enterococcus faecalis; UK873; from a traditional fermented milk; AJ271856
			Bacteria	Firmicutes	Bacilli	Lactobacillales	Enterococcaceae	Enterococcus	Enterococcus casseliflavus (T); LMG 13518; AJ301832
			Bacteria	Firmicutes	Bacilli	Lactobacillales	Enterococcaceae	Enterococcus	Enterococcus gallinarum; LMG 13129; AJ301833
			Bacteria	Firmicutes	Bacilli	Lactobacillales	Enterococcaceae	Enterococcus	Enterococcus pseudoavium; LMG 11426; AJ301837
			Bacteria	Firmicutes	Bacilli	Lactobacillales	Enterococcaceae	Enterococcus	Enterococcus raffinosus; LMG 12999; AJ301838
			Bacteria	Firmicutes	Bacilli	Lactobacillales	Enterococcaceae	Enterococcus	Enterococcus hirae (T); DSM20160; AJ276356
			Bacteria	Firmicutes	Bacilli	Lactobacillales	Enterococcaceae	Enterococcus	Enterococcus durans; DSM20633; AJ276354
			Bacteria	Firmicutes	Bacilli	Lactobacillales	Enterococcaceae	Enterococcus	Enterococcus casseliflavus; AF039903
			Bacteria	Firmicutes	Bacilli	Lactobacillales	Enterococcaceae	Enterococcus	Enterococcus malodoratus; NCFB 846T; Y18339
			Bacteria	Firmicutes	Bacilli	Lactobacillales	Enterococcaceae	Enterococcus	Enterococcus faecalis; Y18293
			Bacteria	Firmicutes	Bacilli	Lactobacillales	Enterococcaceae	Enterococcus	Enterococcus saccharolyticus; NCDO 2594T; Y18357
			Bacteria	Firmicutes	Bacilli	Lactobacillales	Enterococcaceae	Enterococcus	Enterococcus avium; NCFB 2369T; Y18274
			Bacteria	Firmicutes	Bacilli	Lactobacillales	Enterococcaceae	Enterococcus	Enterococcus casseliflavus; NCIMB 13226T; Y18295
			Bacteria	Firmicutes	Bacilli	Lactobacillales	Enterococcaceae	Enterococcus	Enterococcus pseudoavium; NCFB 2138T; Y18356
			Bacteria	Firmicutes	Bacilli	Lactobacillales	Enterococcaceae	Enterococcus	Enterococcus gallinarum; 42; AF039898
			Bacteria	Firmicutes	Bacilli	Lactobacillales	Enterococcaceae	Enterococcus	Enterococcus gallinarum; NCFB 2314; Y18160
			Bacteria	Firmicutes	Bacilli	Lactobacillales	Enterococcaceae	Enterococcus	Enterococcus sp.; LMG12316; X76177
			Bacteria	Firmicutes	Bacilli	Lactobacillales	Enterococcaceae	Enterococcus	Enterococcus dispar; NCIMB 13000T; Y18358
			Bacteria	Proteobacteria	Gammaproteobacteria	Enterobacteriales	Enterobacteriaceae	Citrobacter	Citrobacter braakii (T); CDC 080-58; AF025368
o1394	o1394 16S-Entero1	TGGAGGTTGTGCCCTTGAG	Bacteria	Proteobacteria	Gammaproteobacteria	Aeromonadales	Aeromonadaceae	Aeromonas	Aeromonas schubertii; type strain: ATCC 43700; X60416
			Bacteria	Proteobacteria	Gammaproteobacteria	Enterobacteriales	Enterobacteriaceae	Citrobacter	Citrobacter freundii (T); DSM 30039; AJ233408
			Bacteria	Proteobacteria	Gammaproteobacteria	Enterobacteriales	Enterobacteriaceae	Enterobacter	Enterobacter cloacae subsp. cloacae (T); ATCC13047T; AJ251469
			Bacteria	Proteobacteria	Gammaproteobacteria	Aeromonadales	Aeromonadaceae	Aeromonas	Aeromonas schubertii (T); ATCC 43700T; X74682
			Bacteria	Proteobacteria	Gammaproteobacteria	Enterobacteriales	Enterobacteriaceae	Edwardsiella	Edwardsiella hoshinae (T); JCM1679; AB050825
			Bacteria	Proteobacteria	Gammaproteobacteria	Enterobacteriales	Enterobacteriaceae	Edwardsiella	Edwardsiella ictaluri (T); JCM1680; AB050826
			Bacteria	Proteobacteria	Gammaproteobacteria	Aeromonadales	Aeromonadaceae	Aeromonas	Aeromonas simiae (T); type strain: IBS S6874; AJ536821
			Bacteria	Proteobacteria	Gammaproteobacteria	Enterobacteriales	Enterobacteriaceae	Enterobacter	Cronobacter sakazakii (T); JCM1233; AB004746
			Bacteria	Proteobacteria	Gammaproteobacteria	Enterobacteriales	Enterobacteriaceae	Escherichia	Escherichia coli (T); ATCC 11775T; X80725
			Bacteria	Proteobacteria	Gammaproteobacteria	Enterobacteriales	Enterobacteriaceae	Enterobacter	Enterobacter kobei (T); type strain: CIP 105566; AJ508301
			Bacteria	Proteobacteria	Gammaproteobacteria	Enterobacteriales	Enterobacteriaceae	Enterobacter	Enterobacter asburiae (T); JCM6051; AB004744
			Bacteria	Proteobacteria	Gammaproteobacteria	Enterobacteriales	Enterobacteriaceae	Escherichia	Escherichia coli; PK3; X80723

			Bacteria	Proteobacteria	Gammaproteobacteria	Enterobacteriales	Enterobacteriaceae	Escherichia	Escherichia hermannii; X80675
			Bacteria	Proteobacteria	Gammaproteobacteria	Enterobacteriales	Enterobacteriaceae	Escherichia	Escherichia vulneris (T); ATCC 33821T; X80734
			Bacteria	Proteobacteria	Gammaproteobacteria	Enterobacteriales	Enterobacteriaceae	Edwardsiella	Edwardsiella tarda (T); ATCC15947; AB050827
			Bacteria	Proteobacteria	Gammaproteobacteria	Enterobacteriales	Enterobacteriaceae	Cedecea	Cedecea davisae (T); DSM 4568; AF493976
			Bacteria	Proteobacteria	Gammaproteobacteria	Enterobacteriales	Enterobacteriaceae	Enterobacter	Enterobacter cowanii (T); type strain: CIP 107300; AJ508303
			Bacteria	Proteobacteria	Gammaproteobacteria	Enterobacteriales	Enterobacteriaceae	Enterobacter	Enterobacter hormaechei (T); CIP 103441; AJ508302
			Bacteria	Proteobacteria	Gammaproteobacteria	Aeromonadales	Aeromonadaceae	Aeromonas	Aeromonas simiae; IBS S6652; AJ536820
			Bacteria	Proteobacteria	Gammaproteobacteria	Alteromonadales	Shewanellaceae	Shewanella	Shewanella colwelliana (T); KMM 3587; AY351983
			Bacteria	Proteobacteria	Gammaproteobacteria	Alteromonadales	Shewanellaceae	Shewanella	Shewanella sp. SG133; AB038035
o1395	o1395 16S-Entero2	CCTACTCTTGACATCCAGAGA	Bacteria	Proteobacteria	Gammaproteobacteria	Chromatiales	Chromatiaceae	Rheinheimera	Alkalimonas amylolytica; N10; AF250323
			Bacteria	Proteobacteria	Gammaproteobacteria	Alteromonadales	Shewanellaceae	Shewanella	Shewanella kaireitica (T); c931; AB094598
			Bacteria	Proteobacteria	Gammaproteobacteria	Alteromonadales	Incertae sedis 7	Marinobacterium	Marinobacterium georgiense (T); IAM 1419T; AB021408
			Bacteria	Proteobacteria	Gammaproteobacteria	Oceanospirillales	Oceanospirillaceae	Marinomonas	Marinomonas sp. BSw10005; AY646429
			Bacteria	Proteobacteria	Gammaproteobacteria	Alteromonadales	Shewanellaceae	Shewanella	Barophile WHB 46; WHB 46-1; X54744
			Bacteria	Proteobacteria	Gammaproteobacteria	Alteromonadales	Incertae sedis 7	Marinobacterium	Marinobacterium sp. DMS-S1; AB052100
			Bacteria	Proteobacteria	Gammaproteobacteria	Alteromonadales	Alteromonadaceae	Aestuariibacter	Aestuariibacter salexigens (T); JC2042; AY207502
			Bacteria	Proteobacteria	Gammaproteobacteria	Alteromonadales	Shewanellaceae	Shewanella	Shewanella decolorationis (T); type strain: CCTCC M 203093; S12; AJ609571
			Bacteria	Proteobacteria	Gammaproteobacteria	Alteromonadales	Shewanellaceae	Shewanella	Shewanella sairae (T); SM2-1; AB081762
			Bacteria	Proteobacteria	Gammaproteobacteria	Alteromonadales	Shewanellaceae	Shewanella	Shewanella marinintestina (T); IK-1; AB081757
			Bacteria	Proteobacteria	Gammaproteobacteria	Alteromonadales	Shewanellaceae	Shewanella	Shewanella schlegeliana (T); HRKA1; AB081760
			Bacteria	Proteobacteria	Gammaproteobacteria	Oceanospirillales	Oceanospirillaceae	Marinospirillum	Marinospirillum alkaliphilum (T); Z4; AF275713
			Bacteria	Proteobacteria	Gammaproteobacteria	Oceanospirillales	Oceanospirillaceae	Marinospirillum	Marinospirillum insulare (T); K; AB098510
			Bacteria	Proteobacteria	Gammaproteobacteria	Oceanospirillales	Oceanospirillaceae	Nitrincola	Nitrincola lacisaponensis (T); 4CA; AY567473
			Bacteria	Proteobacteria	Gammaproteobacteria	Alteromonadales	Incertae sedis 7	Marinobacterium	Marinobacterium halophilum; mano11; AY563030
			Bacteria	Proteobacteria	Gammaproteobacteria	Legionellales	Coxiellaceae	unclassified_Coxiellaceae	uncultured gamma proteobacterium; JG30a-KF-94; AJ536872
			Bacteria	Proteobacteria	Gammaproteobacteria	Methylococcales	Methylococcaceae	Methylobacter	Methylobacter vinelandii; 87; L20841
			Bacteria	Proteobacteria	Gammaproteobacteria	Methylococcales	Methylococcaceae	Methylobacter	Methylobacter capsulatus; L20843
			Bacteria	Proteobacteria	Gammaproteobacteria	Enterobacteriales	Enterobacteriaceae	Salmonella	Salmonella enterica subsp. enterica (T); Ty2, ATCC 19430; type strain; pHRC1, pHRC2
o1396	o1396 16S-Entero3	CCTGGTCTTGACATCCACAG	Bacteria	Proteobacteria	Gammaproteobacteria	Enterobacteriales	Enterobacteriaceae	Buchnera	Buchnera aphidicola (Aphis fabae fabae); AY518294
			Bacteria	Proteobacteria	Gammaproteobacteria	Enterobacteriales	Enterobacteriaceae	Buchnera	Buchnera aphidicola (Eriosoma lanuginosum); AJ296748
			Bacteria	Proteobacteria	Gammaproteobacteria	Enterobacteriales	Enterobacteriaceae	Salmonella	Salmonella enterica subsp. enterica (T); ATCC 13311; X80681
			Bacteria	Proteobacteria	Gammaproteobacteria	Enterobacteriales	Enterobacteriaceae	Enterobacter	Enterobacter cloacae subsp. cloacae (T); ATCC13047T; AJ251469
			Bacteria	Proteobacteria	Gammaproteobacteria	Enterobacteriales	Enterobacteriaceae	Salmonella	Salmonella subterranea (T); FRC1; AY373829
			Bacteria	Proteobacteria	Gammaproteobacteria	Enterobacteriales	Enterobacteriaceae	Shigella	Shigella dysenteriae (T); X96966
			Bacteria	Proteobacteria	Gammaproteobacteria	Oceanospirillales	unclassified_Oceanospirillales		uncultured bacterium; ZA3235c; AF382112
			Bacteria	Proteobacteria	Gammaproteobacteria	Enterobacteriales	Enterobacteriaceae	Buchnera	Buchnera sp.; AB033772
			Bacteria	Proteobacteria	Gammaproteobacteria	Enterobacteriales	Enterobacteriaceae	Buchnera	Buchnera sp.; AB033776
			Bacteria	Proteobacteria	Gammaproteobacteria	Enterobacteriales	Enterobacteriaceae	Buchnera	Buchnera sp.; AB033773
			Bacteria	Proteobacteria	Gammaproteobacteria	Enterobacteriales	Enterobacteriaceae	Buchnera	Buchnera sp.; AB033774
			Bacteria	Proteobacteria	Gammaproteobacteria	Enterobacteriales	Enterobacteriaceae	Buchnera	Buchnera sp.; AB033775
			Bacteria	Proteobacteria	Gammaproteobacteria	Enterobacteriales	Enterobacteriaceae	Klebsiella	Klebsiella pneumoniae (T); ATCC13883T; Y17656
			Bacteria	Proteobacteria	Gammaproteobacteria	Enterobacteriales	Enterobacteriaceae	Klebsiella	Serratia liquefaciens (T); JCM1245; AB004752
			Bacteria	Proteobacteria	Gammaproteobacteria	Enterobacteriales	Enterobacteriaceae	Klebsiella	Klebsiella pneumoniae subsp. ozaenae (T); ATCC11296T; Y17654
			Bacteria	Proteobacteria	Gammaproteobacteria	Enterobacteriales	Enterobacteriaceae	Citrobacter	uncultured bacterium; copi44; AY563462
			Bacteria	Proteobacteria	Gammaproteobacteria	Enterobacteriales	Enterobacteriaceae	Klebsiella	Klebsiella variicola (T); F2R9; AJ783916
			Bacteria	Proteobacteria	Gammaproteobacteria	Enterobacteriales	Enterobacteriaceae	Escherichia	Escherichia hermannii; X80675
			Bacteria	Proteobacteria	Gammaproteobacteria	Enterobacteriales	Enterobacteriaceae	Enterobacter	Enterobacter cowanii (T); type strain: CIP 107300; AJ508303
			Bacteria	Proteobacteria	Gammaproteobacteria	Enterobacteriales	Enterobacteriaceae	Salmonella	Salmonella enterica subsp. enterica (T); Ty2, ATCC 19430; type strain; pHRC1, pHRC2
o1397	o1397 16S-Entero4	AAACTGGCAGGCTTGAGTCT	Bacteria	Proteobacteria	Gammaproteobacteria	Enterobacteriales	Enterobacteriaceae	Citrobacter	swine manure bacterium 37-11; AY167970
			Bacteria	Proteobacteria	Gammaproteobacteria	Enterobacteriales	Enterobacteriaceae	Citrobacter	Escherichia coli; MPEC Y-69; AB045731
			Bacteria	Proteobacteria	Gammaproteobacteria	Enterobacteriales	Enterobacteriaceae	Enterobacter	Enterobacter pyrinus (T); KCTC2520; AJ010486
			Bacteria	Proteobacteria	Gammaproteobacteria	Enterobacteriales	Enterobacteriaceae	Citrobacter	Escherichia coli; MPEC EX-33; AB045730
			Bacteria	Proteobacteria	Gammaproteobacteria	Enterobacteriales	Enterobacteriaceae	Citrobacter	Pantoea agglomerans; MY1; AJ506794
			Bacteria	Proteobacteria	Gammaproteobacteria	Enterobacteriales	Enterobacteriaceae	Citrobacter	Enterobacter asburiae; MY2; AJ506159
			Bacteria	Proteobacteria	Gammaproteobacteria	Enterobacteriales	Enterobacteriaceae	Salmonella	Salmonella enterica subsp. enterica (T); ATCC 13311; X80681
			Bacteria	Proteobacteria	Gammaproteobacteria	Enterobacteriales	Enterobacteriaceae	Salmonella	Salmonella subterranea (T); FRC1; AY373829
			Bacteria	Proteobacteria	Gammaproteobacteria	Enterobacteriales	Enterobacteriaceae	Pantoea	Pantoea stewartii (T); LMG 2715; Z96080
			Bacteria	Proteobacteria	Gammaproteobacteria	Enterobacteriales	Enterobacteriaceae	Pantoea	Pantoea ananatis (T); ATCC 33244; U80196
			Bacteria	Proteobacteria	Gammaproteobacteria	Enterobacteriales	Enterobacteriaceae	unclassified_Enterobacteriaceae	Pantoea ananatis (T); ATCC 19321; U80209
			Bacteria	Proteobacteria	Gammaproteobacteria	Enterobacteriales	Enterobacteriaceae	Erwinia	Pantoea agglomerans (T); ATCC 33243; U80202
			Bacteria	Proteobacteria	Gammaproteobacteria	Enterobacteriales	Enterobacteriaceae	Enterobacter	Enterobacter gergoviae (T); JCM1234; AB004748
			Bacteria	Proteobacteria	Gammaproteobacteria	Enterobacteriales	Enterobacteriaceae	Citrobacter	uncultured bacterium; copi44; AY563462
			Bacteria	Proteobacteria	Gammaproteobacteria	Enterobacteriales	Enterobacteriaceae	Erwinia	Erwinia mallotivora (T); LMG 2708; Z96084
			Bacteria	Actinobacteria	Actinobacteria	Coriobacteriales	Coriobacteriaceae	unclassified_Coriobacteriaceae	uncultured actinobacterium; S15A-MN100; AJ534681
			Bacteria	Proteobacteria	Gammaproteobacteria	Enterobacteriales	Enterobacteriaceae	Escherichia	Escherichia hermannii; X80675
			Bacteria	Proteobacteria	Gammaproteobacteria	Enterobacteriales	Enterobacteriaceae	Citrobacter	Citrobacter sp. VA53; AB088049
			Bacteria	Proteobacteria	Gammaproteobacteria	Enterobacteriales	Enterobacteriaceae	Enterobacter	Enterobacter cowanii (T); type strain: CIP 107300; AJ508303
			Bacteria	Proteobacteria	Gammaproteobacteria	Enterobacteriales	Enterobacteriaceae	Buttiauxella	Buttiauxella warmboldiae (T); DSM 9404; AJ233406
o1398	o1398 16S-Entero5	GGAGGGGGATAACTACTGG	Bacteria	Proteobacteria	Gammaproteobacteria	Enterobacteriales	Enterobacteriaceae	Buttiauxella	Buttiauxella ferragutiae (T); DSM 9390; AJ233402
			Bacteria	Proteobacteria	Gammaproteobacteria	Enterobacteriales	Enterobacteriaceae	Citrobacter	Citrobacter freundii (T); DSM 30039; AJ233408
			Bacteria	Proteobacteria	Gammaproteobacteria	Enterobacteriales	Enterobacteriaceae	Buttiauxella	Buttiauxella brennerae (T); DSM 9396; AJ233401
			Bacteria	Proteobacteria	Gammaproteobacteria	Enterobacteriales	Enterobacteriaceae	Brenneria	Brenneria alni (T); ICMP 12481-T; AJ223468
			Bacteria	Proteobacteria	Gammaproteobacteria	Enterobacteriales	Enterobacteriaceae	Buttiauxella	Buttiauxella izardii (T); DSM 9397; AJ233404
			Bacteria	Proteobacteria	Gammaproteobacteria	Enterobacteriales	Enterobacteriaceae	Buttiauxella	Buttiauxella gaviniae (T); DSM 9393; AJ233403
			Bacteria	Proteobacteria	Gammaproteobacteria	Enterobacteriales	Enterobacteriaceae	Brenneria	Brenneria quercina (T); LMG 2724-T; AJ223469
			Bacteria	Proteobacteria	Gammaproteobacteria	Enterobacteriales	Enterobacteriaceae	Budvicia	Budvicia aquatica (T); DSM 5075; AJ233407
			Bacteria	Proteobacteria	Gammaproteobacteria	Enterobacteriales	Enterobacteriaceae	Buttiauxella	Buttiauxella noackiae (T); NSW11T (ATCC 51607T); AJ293689
			Bacteria	Proteobacteria	Gammaproteobacteria	Enterobacteriales	Enterobacteriaceae	Escherichia	Escherichia coli (T); ATCC 11775T; X80725
			Bacteria	Proteobacteria	Epsilonproteobacteria	Campylobacterales	Helicobacteraceae	Sulfurovum	uncultured epsilon proteobacterium; P. palm C 84; AJ441204
			Bacteria	Proteobacteria	Gammaproteobacteria	Enterobacteriales	Enterobacteriaceae	Buttiauxella	Buttiauxella agrestis (T); CDC 1176-81T (ATCC 33320T); AJ293685
			Bacteria	Proteobacteria	Gammaproteobacteria	Enterobacteriales	Enterobacteriaceae	Escherichia	Escherichia coli; PK3; X80723
			Bacteria	Proteobacteria	Gammaproteobacteria	Enterobacteriales	Enterobacteriaceae	Escherichia	Escherichia hermannii; X80675
			Bacteria	Proteobacteria	Gammaproteobacteria	Enterobacteriales	Enterobacteriaceae	Escherichia	Escherichia vulneris (T); ATCC 33821T; X80734
			Bacteria	Proteobacteria	Gammaproteobacteria	Enterobacteriales	Enterobacteriaceae	Cedecea	Cedecea davisae (T); DSM 4568; AF493976
			Bacteria	Proteobacteria	Gammaproteobacteria	Aeromonadales	Aeromonadaceae	Aeromonas	Aeromonas sp. F518; AJ458402
			Bacteria	Proteobacteria	Gammaproteobacteria	Aeromonadales	Aeromonadaceae	Aeromonas	Aeromonas sp. F666D; AJ458404
			Bacteria	Proteobacteria	Gammaproteobacteria	Aeromonadales	Aeromonadaceae	Aeromonas	Aeromonas sp. Aer58; AJ458403
			Bacteria	Firmicutes	Bacilli	Lactobacillales	Enterococcaceae	Enterococcus	swine manure bacterium RT-19B; AY167958

o1399	o1399 16S-EntFae	AAGAGTGAAAGGCGCTTTCG	Bacteria	Firmicutes	Bacilli	Lactobacillales	Enterococcaceae	Enterococcus	Enterococcus faecalis; SL5; AY692453
			Bacteria	Firmicutes	Bacilli	Lactobacillales	Enterococcaceae	Enterococcus	swine manure bacterium RT-2A; AY167933
			Bacteria	Firmicutes	Bacilli	Lactobacillales	Enterococcaceae	Enterococcus	Enterococcus faecalis; SFL; AY850358
			Bacteria	Firmicutes	Bacilli	Lactobacillales	Enterococcaceae	Enterococcus	Enterococcus faecalis; LMG 7937; AJ301831
			Bacteria	Firmicutes	Bacilli	Lactobacillales	Enterococcaceae	Enterococcus	bacterium Te14R; AY587805
			Bacteria	Firmicutes	Bacilli	Lactobacillales	Enterococcaceae	Enterococcus	bacterium Te65R; AY587831
			Bacteria	Firmicutes	Bacilli	Lactobacillales	Enterococcaceae	Enterococcus	Enterococcus faecalis; EC-12; AB154827
			Bacteria	Firmicutes	Bacilli	Lactobacillales	Enterococcaceae	Enterococcus	Enterococcus faecalis; RO90; AF515223
			Bacteria	Firmicutes	Bacilli	Lactobacillales	Enterococcaceae	Enterococcus	Enterococcus faecalis; P7812; AJ276460
			Bacteria	Firmicutes	Bacilli	Lactobacillales	Enterococcaceae	Enterococcus	Enterococcus faecalis; JCM 5803; AB012212
			Bacteria	Firmicutes	Bacilli	Lactobacillales	Enterococcaceae	Enterococcus	Enterococcus faecalis; JH2-2; AF070224
			Bacteria	Firmicutes	Bacilli	Lactobacillales	Enterococcaceae	Enterococcus	Enterococcus faecalis; DJ1; AF447490
			Bacteria	Firmicutes	Bacilli	Lactobacillales	Enterococcaceae	Enterococcus	Enterococcus faecalis; NAB11; AY395018
			Bacteria	Firmicutes	Bacilli	Lactobacillales	Enterococcaceae	Enterococcus	Enterococcus faecalis; AF039902
			Bacteria	Firmicutes	Bacilli	Lactobacillales	Enterococcaceae	Enterococcus	Enterococcus faecalis; CECT481T; AJ420803
			Bacteria	Firmicutes	Bacilli	Lactobacillales	Enterococcaceae	Enterococcus	Enterococcus faecalis; PL9003; AF477496
			Bacteria	Firmicutes	Bacilli	Lactobacillales	Enterococcaceae	Enterococcus	Enterococcus faecalis; C13115; AY550919
			Bacteria	Firmicutes	Bacilli	Lactobacillales	Enterococcaceae	Enterococcus	Enterococcus faecalis; K-4; AB092693
			Bacteria	Firmicutes	Bacilli	Lactobacillales	Enterococcaceae	Enterococcus	Enterococcus faecalis; Y18293
			Bacteria	Proteobacteria	Epsilonproteobacteria	Campylobacterales	Campylobacteraceae	Campylobacter	Campylobacter lanienae; FK172; AB076676
o1400	o1400 16S-Epprot	GGGTAAAATCCGTAGAGATC	Bacteria	Proteobacteria	Epsilonproteobacteria	Campylobacterales	Helicobacteraceae	Helicobacter	Helicobacter mustelae (T); ATCC 43772; M35048
			Bacteria	Proteobacteria	Epsilonproteobacteria	Campylobacterales	Helicobacteraceae	Helicobacter	Helicobacter cinaedi (T); CCUG 18818; M88150
			Bacteria	Proteobacteria	Epsilonproteobacteria	Campylobacterales	Campylobacteraceae	Campylobacter	Campylobacter sp. oral clone BB120; AY005038
			Bacteria	Proteobacteria	Epsilonproteobacteria	Campylobacterales	Campylobacteraceae	Campylobacter	Campylobacter fetus subsp. fetus; 85-388; AY621302
			Bacteria	Proteobacteria	Epsilonproteobacteria	Campylobacterales	Campylobacteraceae	Campylobacter	Campylobacter fetus subsp. fetus; MGH 97-2126; AF219233
			Bacteria	Proteobacteria	Epsilonproteobacteria	Campylobacterales	Campylobacteraceae	Campylobacter	Campylobacter fetus subsp. fetus; 23D; AY621301
			Bacteria	Proteobacteria	Epsilonproteobacteria	Campylobacterales	Helicobacteraceae	Helicobacter	Helicobacter hepaticus (T); Hh-2; U07574
			Bacteria	Proteobacteria	Epsilonproteobacteria	Campylobacterales	Helicobacteraceae	Helicobacter	Candidatus Helicobacter heilmannii (T); HU2; AF506786
			Bacteria	Proteobacteria	Epsilonproteobacteria	Campylobacterales	Helicobacteraceae	Helicobacter	Helicobacter muridarum (T); ST1; ATCC 49282; M80205
			Bacteria	Proteobacteria	Epsilonproteobacteria	Campylobacterales	Campylobacteraceae	Campylobacter	Campylobacter lanienae; FK171; AB076675
			Bacteria	Proteobacteria	Epsilonproteobacteria	Campylobacterales	Campylobacteraceae	Campylobacter	Campylobacter lanienae; FK176; AB076677
			Bacteria	Proteobacteria	Epsilonproteobacteria	Campylobacterales	Helicobacteraceae	Helicobacter	Helicobacter pylori (T); 11637 (NCTC Type strain); Z25741
			Bacteria	Proteobacteria	Epsilonproteobacteria	Campylobacterales	Campylobacteraceae	Campylobacter	Campylobacter fetus subsp. fetus; ATCC 25936; AY621110
			Bacteria	Proteobacteria	Epsilonproteobacteria	Campylobacterales	Campylobacteraceae	Campylobacter	uncultured bacterium; p-4321-4Wa3; AF371868
			Bacteria	Proteobacteria	Epsilonproteobacteria	Campylobacterales	Campylobacteraceae	Sulfurospirillum	Sulfurospirillum arcachonense (T); F1F6 (DSMZ 9755); Y11561
			Bacteria	Proteobacteria	Epsilonproteobacteria	Campylobacterales	Helicobacteraceae	Helicobacter	Helicobacter bizzozeronii (T); CCUG 35045; Y09404
			Bacteria	Proteobacteria	Epsilonproteobacteria	Campylobacterales	Campylobacteraceae	Campylobacter	uncultured bacterium; p-2602-9F5; AF371867
			Bacteria	Proteobacteria	Epsilonproteobacteria	Campylobacterales	Campylobacteraceae	Campylobacter	Campylobacter fetus subsp. fetus; 102\804; AJ306569
			Bacteria	Proteobacteria	Epsilonproteobacteria	Campylobacterales	Campylobacteraceae	Campylobacter	Campylobacter fetus subsp. fetus; 002\827; AJ306568
			Bacteria	Proteobacteria	Alphaproteobacteria	Sphingomonadales	Sphingomonadaceae	Erythrobacter	uncultured organism; ctg_NISA022; DQ396204
o1401	o1401 16S-EryLit	ACTGGATGGCTAGAATCCTG	Bacteria	Proteobacteria	Alphaproteobacteria	Sphingomonadales	Sphingomonadaceae	unclassified_Sphingomonadaceae	uncultured organism; ctg_NISA360; DQ396339
			Bacteria	Proteobacteria	Alphaproteobacteria	Sphingomonadales	Sphingomonadaceae	Erythrobacter	uncultured Novosphingobium sp.; B78; EU360290
			Bacteria	Proteobacteria	Alphaproteobacteria	Sphingomonadales	Sphingomonadaceae	Erythrobacter	uncultured Novosphingobium sp.; B29; EU360289
			Bacteria	Proteobacteria	Alphaproteobacteria	Sphingomonadales	Sphingomonadaceae	Erythrobacter	Altererythrobacter sp. H32; EU726272
			Bacteria	Proteobacteria	Alphaproteobacteria	Sphingomonadales	Sphingomonadaceae	Erythrobacter	alpha proteobacterium MBIC3035; PC4; AB008815
			Bacteria	Proteobacteria	Alphaproteobacteria	Sphingomonadales	Sphingomonadaceae	Erythrobacter	Erythrobacter litoralis; DSM 8509; AF465836
			Bacteria	Proteobacteria	Alphaproteobacteria	Sphingomonadales	Sphingomonadaceae	Erythrobacter	Erythrobacter litoralis (T); DSM8509T; AB013354
			Bacteria	Proteobacteria	Alphaproteobacteria	Sphingomonadales	Sphingomonadaceae	Erythrobacter	Altererythrobacter sp. MSW-14; type strain: MSW-14; FM177586
			Bacteria	Proteobacteria	Alphaproteobacteria	Sphingomonadales	Sphingomonadaceae	Erythrobacter	mucus bacterium 26; AY654770
			Bacteria	Proteobacteria	Alphaproteobacteria	Sphingomonadales	Sphingomonadaceae	Erythrobacter	bacterium s1cb26; DQ416556
			Bacteria	Proteobacteria	Alphaproteobacteria	Sphingomonadales	Sphingomonadaceae	unclassified_Sphingomonadaceae	uncultured bacterium; 23; FN396951
			Bacteria	Proteobacteria	Alphaproteobacteria	Sphingomonadales	Sphingomonadaceae	unclassified_Sphingomonadaceae	uncultured bacterium; TE8; EU911957
			Bacteria	Proteobacteria	Alphaproteobacteria	Sphingomonadales	Sphingomonadaceae	unclassified_Sphingomonadaceae	uncultured bacterium; TE14; EU911963
			Bacteria	Proteobacteria	Gammaproteobacteria	Enterobacteriales	Enterobacteriaceae	Escherichia	Escherichia coli UTI89; CP000243
o1402	o1402 16S-EsCol1	AGTTTTCAGAGATGAGAATGTG	Bacteria	Proteobacteria	Gammaproteobacteria	Enterobacteriales	Enterobacteriaceae	Escherichia	Escherichia coli UTI89; CP000243
			Bacteria	Proteobacteria	Gammaproteobacteria	Enterobacteriales	Enterobacteriaceae	Escherichia	Escherichia coli UTI89; CP000243
			Bacteria	Proteobacteria	Gammaproteobacteria	Enterobacteriales	Enterobacteriaceae	Escherichia	Escherichia coli 536; CP000247
			Bacteria	Proteobacteria	Gammaproteobacteria	Enterobacteriales	Enterobacteriaceae	Escherichia	Escherichia coli 536; CP000247
			Bacteria	Proteobacteria	Gammaproteobacteria	Enterobacteriales	Enterobacteriaceae	Escherichia	Escherichia coli 536; CP000247
			Bacteria	Proteobacteria	Gammaproteobacteria	Enterobacteriales	Enterobacteriaceae	unclassified_Enterobacteriaceae	Escherichia albertii (T); type strain: LMG 20976; AJ508775
			Bacteria	Proteobacteria	Gammaproteobacteria	Enterobacteriales	Enterobacteriaceae	Escherichia	uncultured bacterium; 16saw44-1a05.p1k; EF604190
			Bacteria	Proteobacteria	Gammaproteobacteria	Enterobacteriales	Enterobacteriaceae	Shigella	Shigella flexneri (T); X96963
			Bacteria	Proteobacteria	Betaproteobacteria	Nitrosomonadales	Nitrosomonadaceae	Nitrosomonas	Nitrosomonas sp. Is79A3; AJ621026
			Bacteria	Proteobacteria	Gammaproteobacteria	Enterobacteriales	Enterobacteriaceae	unclassified_Enterobacteriaceae	Escherichia fergusonii (T); ATCC 35469; AF530475
			Bacteria	Proteobacteria	Gammaproteobacteria	Enterobacteriales	Enterobacteriaceae	Escherichia	Escherichia coli (T); ATCC 11775T; X80725
			Bacteria	Proteobacteria	Gammaproteobacteria	Enterobacteriales	Enterobacteriaceae	Escherichia	uncultured bacterium; HH_aai35d03; EU459274
			Bacteria	Proteobacteria	Gammaproteobacteria	Enterobacteriales	Enterobacteriaceae	Escherichia	Escherichia vulneris (T); ATCC 33821T; X80734
			Bacteria	Proteobacteria	Gammaproteobacteria	Enterobacteriales	Enterobacteriaceae	Escherichia	gamma proteobacterium Sflu5; AJ699169
			Bacteria	Proteobacteria	Gammaproteobacteria	Enterobacteriales	Enterobacteriaceae	Escherichia	uncultured gamma proteobacterium; AnCha112f; EF076230
			Bacteria	Proteobacteria	Gammaproteobacteria	Enterobacteriales	Enterobacteriaceae	Escherichia	uncultured gamma proteobacterium; NEC03035; AY911444
			Bacteria	Proteobacteria	Gammaproteobacteria	Enterobacteriales	Enterobacteriaceae	Escherichia	uncultured gamma proteobacterium; JG35-2-JT11; AM072416
			Bacteria	Proteobacteria	Gammaproteobacteria	Enterobacteriales	Enterobacteriaceae	Escherichia	uncultured bacterium; 16saw29-1b03.p1k; EF603477
			Bacteria	Firmicutes	Clostridia	Clostridiales	unclassified_Clostridiales		uncultured Eubacterium sp.; 202G02(oral); AM420046
o1404	o1404 16S-EubGroup1	AACGCAATAAGTGTTCCGCC	Bacteria	Proteobacteria	Deltaproteobacteria	Desulfobacterales	Desulfobacteraceae	Desulfofaba	uncultured bacterium; MD2896-B202; EU385785
			Bacteria	Firmicutes	Clostridia	Clostridiales	unclassified_Clostridiales		uncultured Eubacteriaceae bacterium; 102C12(oral); AM419991
			Bacteria	Firmicutes	Clostridia	unclassified_"Clostridia"			uncultured bacterium; SHA-16; AJ306751
			Bacteria	Firmicutes	Clostridia	Clostridiales	unclassified_Clostridiales		Eubacterium sp. oral clone BB142; AF287764
			Bacteria	Firmicutes	Clostridia	unclassified_"Clostridia"			uncultured bacterium; SHA-98; AJ306758
			Bacteria	Firmicutes	Clostridia	Clostridiales	unclassified_Clostridiales		uncultured bacterium; MA01C09; FM873603
			Bacteria	Firmicutes	Clostridia	Clostridiales	unclassified_Clostridiales		Eubacterium yurii subsp. schtitka; ATCC 43716; AY533382
			Bacteria	Firmicutes	Clostridia	Clostridiales	unclassified_Clostridiales		uncultured bacterium; MA02B11; FM873655
			Bacteria	Firmicutes	Clostridia	Clostridiales	unclassified_Clostridiales		uncultured Eubacteriaceae bacterium; 303E10(oral); AM420107
			Bacteria	Firmicutes	Clostridia	unclassified_"Clostridia"			uncultured bacterium; BSA2B-06; AB175378
			Bacteria	Firmicutes	Clostridia	Clostridiales	unclassified_Clostridiales		Eubacteriaceae bacterium P4P_50 P4; AY207060
			Bacteria	Firmicutes	Clostridia	unclassified_"Clostridia"			uncultured bacterium; BSA2B-07; AB175379
			Bacteria	Firmicutes	Clostridia	Clostridiales	unclassified_Clostridiales		Eubacterium sp. oral strain A03MT; AF287765
			Bacteria	Firmicutes	Clostridia	Clostridiales	unclassified_Clostridiales		Eubacterium yurii subsp. yurii; L34629
			Bacteria	Firmicutes	Clostridia	Clostridiales	unclassified_Clostridiales		Eubacterium yurii subsp. margaretiae; ATCC 43715; AY533381

			Bacteria	Firmicutes	Clostridia	unclassified_"Clostridia"			uncultured bacterium; PLB02; AB232802
			Bacteria	unclassified_Bacteria					uncultured bacterium; S26-95; EU287395
			Bacteria	Firmicutes	Clostridia	Clostridiales	unclassified_Clostridiales		Eubacterium sp. oral clone OH3A; AY947497
			Bacteria	Firmicutes	Clostridia	Clostridiales	unclassified_Clostridiales		uncultured bacterium; nbw843c02c1; GQ026618
			Bacteria	Firmicutes	Clostridia	Clostridiales	Incertae Sedis XIII	Anaerovorax	Eubacterium infirmum (T); W 1471; 1471-3; U13039
o1405	o1405 16S-EubGroup2	AGCCACCCGAAAAGGTGATT	Bacteria	Firmicutes	Clostridia	Clostridiales	Incertae Sedis XIII	Anaerovorax	uncultured bacterium; A_D_01_69; FJ470437
			Bacteria	Firmicutes	Clostridia	Clostridiales	Incertae Sedis XIII	Anaerovorax	Eubacterium sp. oral strain A35MT; AF287761
			Bacteria	Firmicutes	Clostridia	Clostridiales	Incertae Sedis XIII	Anaerovorax	Eubacterium sulci (T); ATCC 35585; AJ006963
			Bacteria	Firmicutes	Clostridia	Clostridiales	Incertae Sedis XIII	Anaerovorax	uncultured Eubacteriaceae bacterium; 301C06(oral); AM420061
			Bacteria	Firmicutes	Clostridia	Clostridiales	Incertae Sedis XIII	Anaerovorax	uncultured bacterium; nbw1209b08c1; GQ077474
			Bacteria	Firmicutes	Clostridia	Clostridiales	Incertae Sedis XIII	Anaerovorax	uncultured bacterium; X444; AY980542
			Bacteria	Firmicutes	Clostridia	Clostridiales	Incertae Sedis XIII	Anaerovorax	uncultured bacterium; nbw1075e11c1; GQ052871
			Bacteria	Firmicutes	Clostridia	Clostridiales	Incertae Sedis XIII	Anaerovorax	uncultured bacterium; nbw1068b04c1; GQ057704
			Bacteria	Firmicutes	Clostridia	Clostridiales	Incertae Sedis XIII	Anaerovorax	uncultured Peptostreptococcaceae bacterium; 3B85; FJ976242
			Bacteria	Firmicutes	Clostridia	Clostridiales	Incertae Sedis XIII	Anaerovorax	uncultured bacterium; nbw1076c11c1; GQ052917
			Bacteria	Firmicutes	Clostridia	Clostridiales	Incertae Sedis XIII	Anaerovorax	uncultured bacterium; nbw1073c08c1; GQ057858
			Bacteria	Firmicutes	Clostridia	Clostridiales	Lachnospiraceae	unclassified_"Lachnospiraceae"	uncultured bacterium; A_D_01_58; FJ470430
o1406	o1406 16S-EubGroup3	TCTTTCGGGACAAGGGATAC	Bacteria	Firmicutes	Clostridia	Clostridiales	Lachnospiraceae	unclassified_"Lachnospiraceae"	uncultured human oral bacterium; DWG06; AY672089
			Bacteria	Firmicutes	Clostridia	Clostridiales	Lachnospiraceae	unclassified_"Lachnospiraceae"	Eubacterium saburreum; JCM 11021; AB525414
			Bacteria	Firmicutes	Clostridia	Clostridiales	Lachnospiraceae	unclassified_"Lachnospiraceae"	uncultured bacterium; AP07S.29; AM277332
			Bacteria	Firmicutes	Clostridia	Clostridiales	Lachnospiraceae	unclassified_"Lachnospiraceae"	human oral bacterium C73; AF201981
			Bacteria	Firmicutes	Clostridia	Clostridiales	Lachnospiraceae	unclassified_"Lachnospiraceae"	uncultured bacterium; nbw816h06c1; GQ015462
			Bacteria	Firmicutes	Clostridia	Clostridiales	Lachnospiraceae	unclassified_"Lachnospiraceae"	uncultured bacterium; nbw208d02c1; GQ073379
			Bacteria	Firmicutes	Clostridia	Clostridiales	Lachnospiraceae	unclassified_"Lachnospiraceae"	uncultured bacterium; nbw205e08c1; GQ073138
			Bacteria	Firmicutes	Clostridia	Clostridiales	Lachnospiraceae	unclassified_"Lachnospiraceae"	uncultured bacterium; nbw1154c07c1; GQ076862
			Bacteria	Firmicutes	Clostridia	Clostridiales	Lachnospiraceae	unclassified_"Lachnospiraceae"	uncultured Lachnospiraceae bacterium; 12B113; FJ976333
			Bacteria	Firmicutes	Clostridia	Clostridiales	Lachnospiraceae	unclassified_"Lachnospiraceae"	uncultured Lachnospiraceae bacterium; 3681; FJ976217
			Bacteria	Firmicutes	Clostridia	Clostridiales	Lachnospiraceae	unclassified_"Lachnospiraceae"	uncultured Lachnospiraceae bacterium; 7BB544; FJ976273
			Bacteria	Firmicutes	Clostridia	Clostridiales	Lachnospiraceae	unclassified_"Lachnospiraceae"	Eubacterium saburreum-like sp. oral clone CK004; AF287776
o1407	o1407 16S-EubOral	AAACTTTTCGGTGCCGAAGC	Bacteria	Firmicutes	Clostridia	Clostridiales	Incertae Sedis XII	Fusibacter	uncultured bacterium; PL-5B6; AY570635
o1409	o1409 16S-EuGroup1	TGGGGAGTATGCACGCAAG	Bacteria	Firmicutes	Erysipelotrichi	Erysipelotrichales	Erysipelotrichaceae	Erysipelotrichaceae Incertae Sedis	Eubacterium cylindroides; JCM 7786; AB018186
			Bacteria	Firmicutes	Erysipelotrichi	Erysipelotrichales	Erysipelotrichaceae	Erysipelotrichaceae Incertae Sedis	Eubacterium cylindroides; JCM 7787; AB018187
			Bacteria	Firmicutes	Erysipelotrichi	Erysipelotrichales	Erysipelotrichaceae	Erysipelothrix	uncultured bacterium; PL-6B11; AY570636
			Bacteria	Firmicutes	Erysipelotrichi	Erysipelotrichales	Erysipelotrichaceae	Erysipelotrichaceae Incertae Sedis	Clostridium aff. innocuum CM970; AF028352
			Bacteria	Firmicutes	Erysipelotrichi	Erysipelotrichales	Erysipelotrichaceae	Allobaculum	Allobaculum stercoricanis (T); type strain: DSM 13633; AJ417075
			Bacteria	Firmicutes	Erysipelotrichi	Erysipelotrichales	Erysipelotrichaceae	Erysipelotrichaceae Incertae Sedis	butyrate-producing bacterium T2-87; AY305306
			Bacteria	Firmicutes	Erysipelotrichi	Erysipelotrichales	Erysipelotrichaceae	Erysipelotrichaceae Incertae Sedis	butyrate-producing bacterium SM7/11; AY305313
			Bacteria	Firmicutes	Erysipelotrichi	Erysipelotrichales	Erysipelotrichaceae	Erysipelotrichaceae Incertae Sedis	Eubacterium tortuosum (T); L34683
			Bacteria	Firmicutes	Erysipelotrichi	Erysipelotrichales	Erysipelotrichaceae	Erysipelotrichaceae Incertae Sedis	Eubacterium dolichum (T); L34682
			Bacteria	Firmicutes	Erysipelotrichi	Erysipelotrichales	Erysipelotrichaceae	Erysipelotrichaceae Incertae Sedis	Clostridium innocuum (T); M23732
			Bacteria	Firmicutes	Erysipelotrichi	Erysipelotrichales	Erysipelotrichaceae	unclassified_Erysipelotrichaceae	cf. Clostridium sp. MLG055; AF304435
			Bacteria	Firmicutes	Erysipelotrichi	Erysipelotrichales	Erysipelotrichaceae	Erysipelotrichaceae Incertae Sedis	Eubacterium cylindroides (T); ATCC27803; L34617
			Bacteria	Firmicutes	Erysipelotrichi	Erysipelotrichales	Erysipelotrichaceae	Erysipelotrichaceae Incertae Sedis	Eubacterium biforme (T); ATCC 27806; M59230
			Bacteria	Firmicutes	Erysipelotrichi	Erysipelotrichales	Erysipelotrichaceae	Erysipelothrix	Mollicutes bacterium pACH93; AY297808
			Bacteria	Firmicutes	Erysipelotrichi	Erysipelotrichales	Erysipelotrichaceae	Holdemania	Holdemania filiformis (T); ATCC 51649; Y11466
			Bacteria	Firmicutes	Erysipelotrichi	Erysipelotrichales	Erysipelotrichaceae	Erysipelotrichaceae Incertae Sedis	Eubacterium cylindroides; L34616
			Bacteria	Firmicutes	Erysipelotrichi	Erysipelotrichales	Erysipelotrichaceae	Erysipelotrichaceae Incertae Sedis	Streptococcus pleomorphus (T); M23730
			Bacteria	Cyanobacteria	Cyanobacteria	unclassified_Cyanobacteria			Pseudanabaena tremula UTCC 471; AF218371
			Bacteria	Firmicutes	Erysipelotrichi	Erysipelotrichales	Erysipelotrichaceae	Holdemania	Clostridium sp. MLG480; AY653232
			Bacteria	Firmicutes	Clostridia	Clostridiales	Ruminococcaceae	Papillibacter	butyrate-producing bacterium A2-207; AJ270471
o1410	o1410 16S-EuGroup2	CACGTACTACAATGGCAGTC	Bacteria	Firmicutes	Clostridia	Clostridiales	Ruminococcaceae	Papillibacter	uncultured bacterium; SK49; AY753394
			Bacteria	Firmicutes	Clostridia	Clostridiales	Ruminococcaceae	unclassified_"Ruminococcaceae"	uncultured bacterium; p-1877-s962-3; AF371805
			Bacteria	Firmicutes	Clostridia	Clostridiales	Ruminococcaceae	unclassified_"Ruminococcaceae"	uncultured bacterium; p-2560-9F5; AF371806
			Bacteria	Firmicutes	Clostridia	Clostridiales	Ruminococcaceae	Papillibacter	uncultured eubacterium; vadinBA08; U81642
			Bacteria	Firmicutes	Clostridia	Clostridiales	Ruminococcaceae	Papillibacter	uncultured bacterium; p-900-a5; AF371807
			Bacteria	Firmicutes	Clostridia	Clostridiales	Ruminococcaceae	Papillibacter	uncultured bacterium adhufec296; AF132258
			Bacteria	Firmicutes	Clostridia	Clostridiales	Ruminococcaceae	unclassified_"Ruminococcaceae"	uncultured bacterium; L11-2; AJ400270
			Bacteria	Firmicutes	Clostridia	Clostridiales	Ruminococcaceae	unclassified_"Ruminococcaceae"	uncultured bacterium; rc3-49; AY239438
			Bacteria	Firmicutes	Clostridia	Clostridiales	Ruminococcaceae	unclassified_"Ruminococcaceae"	uncultured bacterium; KG01; AY974810
			Bacteria	Firmicutes	Clostridia	Clostridiales	Ruminococcaceae	unclassified_"Ruminococcaceae"	uncultured bacterium; KN93; AY975232
			Bacteria	Firmicutes	Clostridia	Clostridiales	Ruminococcaceae	unclassified_"Ruminococcaceae"	uncultured bacterium; L860; AY981623
			Bacteria	Firmicutes	Clostridia	Clostridiales	Ruminococcaceae	unclassified_"Ruminococcaceae"	uncultured bacterium; LA38; AY981697
			Bacteria	Firmicutes	Clostridia	Clostridiales	Ruminococcaceae	Papillibacter	Eubacterium desmolans (T); L34618
			Bacteria	Firmicutes	Clostridia	Clostridiales	Ruminococcaceae	unclassified_"Ruminococcaceae"	uncultured bacterium; KY20; AY982707
			Bacteria	Firmicutes	Clostridia	Clostridiales	Ruminococcaceae	unclassified_"Ruminococcaceae"	uncultured bacterium; E048; AY979854
			Bacteria	Firmicutes	Clostridia	Clostridiales	Ruminococcaceae	unclassified_"Ruminococcaceae"	uncultured bacterium; G094; AY981157
			Bacteria	Firmicutes	Clostridia	Clostridiales	Ruminococcaceae	unclassified_"Ruminococcaceae"	uncultured bacterium; F245; AY978338
			Bacteria	Firmicutes	Clostridia	Clostridiales	Ruminococcaceae	Papillibacter	uncultured bacterium; Z015; AY916177
			Bacteria	Firmicutes	Clostridia	Clostridiales	Ruminococcaceae	Papillibacter	uncultured bacterium; ckncm325-B3-50; AF376229
			Bacteria	Firmicutes	Clostridia	Clostridiales	unclassified_Clostridiales		uncultured Eubacterium sp.; 202G02(oral); AM420046
o1411	o1411 16S-EuGroup3	ACGCAATAAGTGTTCCGCCT	Bacteria	Proteobacteria	Deltaproteobacteria	Desulfobacterales	Desulfobacteraceae	Desulfofaba	uncultured bacterium; MD2896-B202; EU385785
			Bacteria	Firmicutes	Clostridia	Clostridiales	unclassified_Clostridiales		uncultured Eubacteriaceae bacterium; 102C12(oral); AM419991
			Bacteria	Firmicutes	Clostridia	unclassified_"Clostridia"			uncultured bacterium; SHA-16; AJ306751
			Bacteria	Firmicutes	Clostridia	Clostridiales	unclassified_Clostridiales		Eubacterium sp. oral clone BB142; AF287764
			Bacteria	Firmicutes	Clostridia	unclassified_"Clostridia"			uncultured bacterium; SHA-98; AJ306758
			Bacteria	Firmicutes	Clostridia	Clostridiales	unclassified_Clostridiales		uncultured bacterium; MA01C09; FM873603
			Bacteria	Firmicutes	Clostridia	Clostridiales	unclassified_Clostridiales		Eubacterium yurii subsp. schtitka; ATCC 43716; AY533382
			Bacteria	Firmicutes	Clostridia	Clostridiales	unclassified_Clostridiales		uncultured bacterium; MA02B11; FM873655
			Bacteria	Firmicutes	Clostridia	Clostridiales	unclassified_Clostridiales		uncultured Eubacteriaceae bacterium; 303E10(oral); AM420107
			Bacteria	Firmicutes	Clostridia	unclassified_"Clostridia"			uncultured bacterium; BSA2B-06; AB175378
			Bacteria	Firmicutes	Clostridia	Clostridiales	unclassified_Clostridiales		Eubacteriaceae bacterium P4P_50 P4; AY207060
			Bacteria	Firmicutes	Clostridia	unclassified_"Clostridia"			uncultured bacterium; BSA2B-07; AB175379
			Bacteria	Firmicutes	Clostridia	Clostridiales	unclassified_Clostridiales		Eubacterium sp. oral strain A03MT; AF287765
			Bacteria	Firmicutes	Clostridia	Clostridiales	unclassified_Clostridiales		Eubacterium yurii subsp. yurii; L34629
			Bacteria	Firmicutes	Clostridia	Clostridiales	unclassified_Clostridiales		Eubacterium yurii subsp. margaretiae; ATCC 43715; AY533381
			Bacteria	Firmicutes	Clostridia	unclassified_"Clostridia"			uncultured bacterium; PLB02; AB232802
			Bacteria	unclassified_Bacteria					uncultured bacterium; S26-95; EU287395
			Bacteria	Firmicutes	Clostridia	Clostridiales	unclassified_Clostridiales		Eubacterium sp. oral clone OH3A; AY947497

			Bacteria	Firmicutes	Clostridia	Clostridiales	unclassified_Clostridiales		uncultured bacterium; nbw843c02c1; GQ026618
o1412	o1412 16S-Euyac	TGCATGGCCGCCGTCAG	Archaea	Euryarchaeota	Methanobacteria	Methanobacteriales	Methanobacteriaceae	Methanobacterium	Methanobacterium curvum; AF276958
			Archaea	Euryarchaeota	Halobacteria	Halobacteriales	Halobacteriaceae	Haloarcula	Haloarcula sp. (T); 801030/1; AB010964
			Archaea	Euryarchaeota	Methanobacteria	Methanobacteriales	Methanobacteriaceae	Methanobacterium	Methanobacterium congolense (T); C; AF233586
			Archaea	Euryarchaeota	Methanobacteria	Methanobacteriales	Methanobacteriaceae	Methanobacterium	Methanobacterium subterraneum (T); A8p, DSM 11074; X99044
			Archaea	Euryarchaeota	Methanobacteria	Methanobacteriales	Methanobacteriaceae	Methanobacterium	Methanobacterium subterraneum; C2BIS, DSM 11075; X99045
			Archaea	Euryarchaeota	Methanobacteria	Methanobacteriales	Methanobacteriaceae	Methanobacterium	Methanobacterium formicicum (T); DSMZ1535; AF169245
			Archaea	Euryarchaeota	Methanobacteria	Methanobacteriales	Methanobacteriaceae	Methanobacterium	Methanobacterium aarhusense (T); H2-LR; AY386124
			Archaea	Euryarchaeota	Methanobacteria	Methanobacteriales	Methanobacteriaceae	Methanobacterium	uncultured euryarchaeote; 15ar16; AY251026
			Archaea	Euryarchaeota	Methanobacteria	Methanobacteriales	Methanobacteriaceae	Methanobacterium	Methanothermobacter thermautotrophicus; X68716
			Archaea	Euryarchaeota	Methanobacteria	Methanobacteriales	Methanobacteriaceae	Methanobacterium	Methanothermobacter thermautotrophicus (T); X68712
			Archaea	Euryarchaeota	Methanobacteria	Methanobacteriales	Methanobacteriaceae	Methanobacterium	Methanothermobacter thermautotrophicus; X68715
			Archaea	Euryarchaeota	Methanobacteria	Methanobacteriales	Methanobacteriaceae	Methanobacterium	Methanothermobacter thermautotrophicus; X68713
			Archaea	Euryarchaeota	Methanobacteria	Methanobacteriales	Methanobacteriaceae	Methanobacterium	Methanothermobacter thermautotrophicus; X68714
			Archaea	Euryarchaeota	Methanobacteria	Methanobacteriales	Methanobacteriaceae	Methanobacterium	Methanothermobacter thermautotrophicus; X68711
			Archaea	Euryarchaeota	Methanobacteria	Methanobacteriales	Methanobacteriaceae	Methanobacterium	Methanothermobacter thermautotrophicus; X68720
			Archaea	Euryarchaeota	Methanobacteria	Methanobacteriales	Methanobacteriaceae	Methanobacterium	Methanobacterium bryantii; MOH; AY196657
			Archaea	Euryarchaeota	Methanobacteria	Methanobacteriales	Methanobacteriaceae	Methanobacterium	Methanothermobacter thermautotrophicus; delta H; AY196660
			Archaea	Euryarchaeota	Methanobacteria	Methanobacteriales	Methanobacteriaceae	Methanobacterium	Methanothermobacter thermautotrophicus; GC-1; AY196661
			Archaea	Euryarchaeota	Methanobacteria	Methanobacteriales	Methanobacteriaceae	Methanobacterium	Methanobacterium bryantii; MOHG; AY196658
			Archaea	Euryarchaeota	Methanobacteria	Methanobacteriales	Methanobacteriaceae	Methanobacterium	Methanobacterium formicicum; MF; AY196659
o1413	o1413 16S-Fermic	GACACGGTCCAGACACCTA	Bacteria	Verrucomicrobia	Verrucomicrobiae	Verrucomicrobiales	Verrucomicrobiaceae	Verrucomicrobium	uncultured bacterium; 300A-H02; AY661973
			Bacteria	Verrucomicrobia	Verrucomicrobiae	Verrucomicrobiales	Verrucomicrobiaceae	Verrucomicrobium	uncultured sludge bacterium A21; AF234742
			Bacteria	Verrucomicrobia	Verrucomicrobiae	Verrucomicrobiales	Verrucomicrobiaceae	Verrucomicrobium	uncultured sludge bacterium A38; AF234714
			Bacteria	Verrucomicrobia	Verrucomicrobiae	Verrucomicrobiales	Opitutaceae	Opitutus	Opitutus sp. VeGlc2; X99390
			Bacteria	Verrucomicrobia	Verrucomicrobiae	Verrucomicrobiales	Opitutaceae	Opitutus	Opitutus sp. VeSm13; X99392
			Bacteria	Verrucomicrobia	Verrucomicrobiae	Verrucomicrobiales	Verrucomicrobiaceae	Verrucomicrobium	uncultured bacterium; MIZ47; AB179538
			Bacteria	Verrucomicrobia	Verrucomicrobiae	Verrucomicrobiales	Opitutaceae	Opitutus	Opitutus terrae (T); PB90-1; AJ229235
			Bacteria	Verrucomicrobia	Verrucomicrobiae	Verrucomicrobiales	Xiphinematobacteriaceae	Xiphinematobacter	Candidatus Xiphinematobacter brevicolli (T); AF217462
			Bacteria	Verrucomicrobia	Verrucomicrobiae	Verrucomicrobiales	Verrucomicrobiaceae	Verrucomicrobium	uncultured bacterium; MIZ37; AB179528
			Bacteria	Verrucomicrobia	Verrucomicrobiae	Verrucomicrobiales	Verrucomicrobiaceae	Verrucomicrobium	Verrucomicrobium spinosum (T); DSM 4136T; X90515
			Bacteria	Verrucomicrobia	Verrucomicrobiae	Verrucomicrobiales	Verrucomicrobiaceae	Verrucomicrobium	uncultured Verrucomicrobia bacterium; JAB FS 112; AY734252
			Bacteria	Verrucomicrobia	Verrucomicrobiae	Verrucomicrobiales	Opitutaceae	Opitutus	unidentified eubacterium clone BSV64; AJ229212
			Bacteria	Verrucomicrobia	Verrucomicrobiae	Verrucomicrobiales	Opitutaceae	Opitutus	Opitutus terrae; PB90-3; AJ229239
			Bacteria	Verrucomicrobia	Verrucomicrobiae	Verrucomicrobiales	Verrucomicrobiaceae	Verrucomicrobium	uncultured bacterium; MIZ43; AB179534
			Bacteria	Verrucomicrobia	Verrucomicrobiae	Verrucomicrobiales	Verrucomicrobiaceae	Prosthecobacter	Prosthecobacter dejongeii (T); FC1; U60012
			Bacteria	Verrucomicrobia	Verrucomicrobiae	Verrucomicrobiales	Verrucomicrobiaceae	Verrucomicrobium	uncultured bacterium; 20BSU56; AJ863205
			Bacteria	Verrucomicrobia	Verrucomicrobiae	Verrucomicrobiales	Verrucomicrobiaceae	Akkermansia	Akkermansia muciniphila; Muc; AY271254
			Bacteria	Verrucomicrobia	Verrucomicrobiae	Verrucomicrobiales	Xiphinematobacteriaceae	Xiphinematobacter	Candidatus Xiphinematobacter americani (T); AF217460
			Bacteria	Verrucomicrobia	Verrucomicrobiae	Verrucomicrobiales	Xiphinematobacteriaceae	Xiphinematobacter	Candidatus Xiphinematobacter rivesi (T); AF217461
			Bacteria	Verrucomicrobia	Verrucomicrobiae	Verrucomicrobiales	Verrucomicrobiaceae	Verrucomicrobium	uncultured bacterium; B2_49BS; AM157350
o1415	o1415 16S-FirOral	ACGGAATGGCAAGTCTGAAG	Bacteria	Firmicutes	Clostridia	Clostridiales	Lachnospiraceae	Oribacterium	Eubacterium sp. oral clone JN088; AY349377
			Bacteria	Firmicutes	Clostridia	Clostridiales	Lachnospiraceae	Oribacterium	uncultured Oribacterium sp.; EHFS1_S01f; EU071470
			Bacteria	Firmicutes	Clostridia	Clostridiales	Lachnospiraceae	Oribacterium	Oribacterium sinus; F0268; FJ577261
			Bacteria	Firmicutes	Clostridia	Clostridiales	Lachnospiraceae	Oribacterium	Oribacterium sp. F0262; FJ577249
			Bacteria	Firmicutes	Clostridia	Clostridiales	Lachnospiraceae	Oribacterium	uncultured bacterium; AP07S.41; AM277344
			Bacteria	Firmicutes	Clostridia	Clostridiales	Lachnospiraceae	Oribacterium	uncultured bacterium; BF0002C015; AM697318
			Bacteria	Firmicutes	Clostridia	Clostridiales	Lachnospiraceae	Oribacterium	Lachnospiraceae oral clone MCE9_31; AF481220
			Bacteria	Firmicutes	Clostridia	Clostridiales	Lachnospiraceae	Oribacterium	Firmicutes oral clone AO068; AF287771
			Bacteria	Firmicutes	Clostridia	Clostridiales	Lachnospiraceae	Oribacterium	Firmicutes sp. oral strain FTB41; AF287770
			Bacteria	Firmicutes	Clostridia	Clostridiales	Lachnospiraceae	Oribacterium	Oribacterium sinus (T); AIP 354.02; AY323228
			Bacteria	Firmicutes	Clostridia	Clostridiales	Lachnospiraceae	Oribacterium	uncultured Lachnospiraceae bacterium; 401E04(oral); AM420120
			Bacteria	Firmicutes	Clostridia	Clostridiales	Lachnospiraceae	Oribacterium	uncultured bacterium; nbu577h04c2; GQ054419
			Bacteria	Firmicutes	Clostridia	Clostridiales	Lachnospiraceae	Oribacterium	uncultured bacterium; nbw904g10c1; GQ032444
			Bacteria	Firmicutes	Clostridia	Clostridiales	Lachnospiraceae	Oribacterium	uncultured bacterium; nbw901e03c1; GQ027271
			Bacteria	Firmicutes	Clostridia	Clostridiales	Lachnospiraceae	Oribacterium	uncultured bacterium; nbw1068b02c1; GQ057703
			Bacteria	Firmicutes	Clostridia	Clostridiales	Lachnospiraceae	Oribacterium	uncultured bacterium; nbw901d08c1; GQ027266
			Bacteria	Firmicutes	Clostridia	Clostridiales	Lachnospiraceae	Oribacterium	uncultured bacterium; nbw904f09c1; GQ032284
			Bacteria	Firmicutes	Clostridia	Clostridiales	Lachnospiraceae	Oribacterium	uncultured bacterium; nbw342a04c1; GQ091089
			Bacteria	Firmicutes	Clostridia	Clostridiales	Lachnospiraceae	Butyrivibrio	uncultured bacterium; CK73; FJ032442
			Bacteria	Firmicutes	Clostridia	Clostridiales	Lachnospiraceae	Oribacterium	uncultured Oribacterium sp.; 3P-4-1-P15; EU706274
o1417	o1417 16S-Flvbac	TGAGGTGTCAGGTTAAGTCC	Bacteria	Bacteroidetes	Flavobacteria	Flavobacteriales	Flavobacteriaceae	Flavobacterium	Flavobacterium aff. xylanivorum A1/C-aer/OIV; AJ297440
			Bacteria	Bacteroidetes	Flavobacteria	Flavobacteriales	Flavobacteriaceae	Flavobacterium	Flavobacterium columnare; EK-28 (= IAM 14820); AB016515
			Bacteria	Bacteroidetes	Flavobacteria	Flavobacteriales	Flavobacteriaceae	Flavobacterium	Flavobacterium columnare; LP 8; AB015480
			Bacteria	Bacteroidetes	Flavobacteria	Flavobacteriales	Flavobacteriaceae	Flavobacterium	Flavobacterium aquatile (T); M62797
			Bacteria	Bacteroidetes	Flavobacteria	Flavobacteriales	Flavobacteriaceae	Flavobacterium	Flavobacterium sp. SW254; AF493695
			Bacteria	Bacteroidetes	Flavobacteria	Flavobacteriales	Flavobacteriaceae	Flavobacterium	Flavobacterium sp. EP037; AF493636
			Bacteria	Bacteroidetes	Flavobacteria	Flavobacteriales	Flavobacteriaceae	Flavobacterium	Flavobacterium sp. EP030; AF493635
			Bacteria	Bacteroidetes	Flavobacteria	Flavobacteriales	Flavobacteriaceae	Flavobacterium	Flavobacterium sp. EP241; AF493644
			Bacteria	Bacteroidetes	Flavobacteria	Flavobacteriales	Flavobacteriaceae	Flavobacterium	Flavobacterium sp. EP130; AF493637
			Bacteria	Bacteroidetes	Flavobacteria	Flavobacteriales	Flavobacteriaceae	Flavobacterium	Flavobacterium sp. EP372; AF493653
			Bacteria	Bacteroidetes	Flavobacteria	Flavobacteriales	Flavobacteriaceae	Flavobacterium	Flavobacterium sp. EP236; AF493642
			Bacteria	Bacteroidetes	Flavobacteria	Flavobacteriales	Flavobacteriaceae	Flavobacterium	Flavobacterium sp. EP233; AF493640
			Bacteria	Bacteroidetes	Flavobacteria	Flavobacteriales	Flavobacteriaceae	Flavobacterium	Flavobacterium sp. EP215; AF493657
			Bacteria	Bacteroidetes	Flavobacteria	Flavobacteriales	Flavobacteriaceae	Flavobacterium	Flavobacterium sp. EP306; AF493651
			Bacteria	Bacteroidetes	Flavobacteria	Flavobacteriales	Flavobacteriaceae	Flavobacterium	Flavobacterium sp. EP235; AF493641
			Bacteria	Bacteroidetes	Flavobacteria	Flavobacteriales	Flavobacteriaceae	Flavobacterium	Flavobacterium sp. EP204; AF493656
			Bacteria	Bacteroidetes	Flavobacteria	Flavobacteriales	Flavobacteriaceae	Flavobacterium	Flavobacterium sp. EP251; AF493646
			Bacteria	Bacteroidetes	Flavobacteria	Flavobacteriales	Flavobacteriaceae	Flavobacterium	Flavobacterium sp. EP217; AF493658
			Bacteria	Bacteroidetes	Flavobacteria	Flavobacteriales	Flavobacteriaceae	Flavobacterium	Flavobacterium sp. EP101; AF493649
			Bacteria	Bacteroidetes	Flavobacteria	Flavobacteriales	Flavobacteriaceae	Flavobacterium	Flavobacterium sp. EP209; AF493638
			Bacteria	Bacteroidetes	Sphingobacteria	Sphingobacteriales	Flexibacteraceae	Hymenobacter	Hymenobacter chitinivorans (T); type strain: Txc1; Y18837
o1418	o1418 16S-Flxbac1	CGTGAAGCTGGAATCACTAG	Bacteria	Bacteroidetes	Sphingobacteria	Sphingobacteriales	Flexibacteraceae	Hymenobacter	Hymenobacter gelipurpurascens (T); type strain: Txg1; Y18836
			Bacteria	Bacteroidetes	Sphingobacteria	Sphingobacteriales	Flexibacteraceae	Hymenobacter	Hymenobacter roseosalivarius; AA688; Y18834
			Bacteria	Bacteroidetes	Sphingobacteria	Sphingobacteriales	Flexibacteraceae	Hymenobacter	Hymenobacter roseosalivarius (T); AA718; Y18833
			Bacteria	Bacteroidetes	Sphingobacteria	Sphingobacteriales	Flexibacteraceae	Hymenobacter	Hymenobacter ocellatus; type strain: Myx 2105; Y18835
			Bacteria	Bacteroidetes	Sphingobacteria	Sphingobacteriales	Flexibacteraceae	Hymenobacter	uncultured Taxeobacter sp.; KL-59-7-7; AF408279
			Bacteria	Bacteroidetes	Sphingobacteria	Sphingobacteriales	Flexibacteraceae	Hymenobacter	uncultured Taxeobacter sp.; KL-2-4-3; AF408278

			Bacteria	Bacteroidetes	Sphingobacteria	Sphingobacteriales	Flexibacteraceae	Hymenobacter	uncultured Hymenobacter group bacterium; KL-27-1-11; AF408277
			Bacteria	Bacteroidetes	Sphingobacteria	Sphingobacteriales	Flexibacteraceae	Hymenobacter	Taxeobacter ocellatus (T); Txo1; Y18838
			Bacteria	Bacteroidetes	Sphingobacteria	Sphingobacteriales	Flexibacteraceae	Hymenobacter	uncultured Hymenobacter sp.; KL-2-4-2; AF408274
			Bacteria	Bacteroidetes	Sphingobacteria	Sphingobacteriales	Flexibacteraceae	Hymenobacter	uncultured Hymenobacter sp.; KL-2-4-9; AF408275
			Bacteria	Bacteroidetes	Sphingobacteria	Sphingobacteriales	Flexibacteraceae	Hymenobacter	uncultured Hymenobacter group bacterium; KL-2-5-13; AF408273
			Bacteria	Bacteroidetes	Sphingobacteria	Sphingobacteriales	Flexibacteraceae	Hymenobacter	uncultured Hymenobacter group bacterium; KL-53-2-6; AF408270
			Bacteria	Bacteroidetes	Sphingobacteria	Sphingobacteriales	Flexibacteraceae	Hymenobacter	uncultured Taxeobacter sp.; KL-2-5-12; AF408272
			Bacteria	Bacteroidetes	Sphingobacteria	Sphingobacteriales	Flexibacteraceae	Hymenobacter	uncultured Hymenobacter sp.; KL-26-2-11; AF408276
			Bacteria	Bacteroidetes	Sphingobacteria	Sphingobacteriales	Flexibacteraceae	Hymenobacter	Hymenobacter norwichensis (T); type strain: NS/50; AJ549285
			Bacteria	Bacteroidetes	Sphingobacteria	Sphingobacteriales	Flexibacteraceae	Hymenobacter	Antarctic bacterium R-7572; AJ440980
			Bacteria	Bacteroidetes	Sphingobacteria	Sphingobacteriales	Flexibacteraceae	Hymenobacter	Hymenobacter sp. NS/2; AJ549284
			Bacteria	Bacteroidetes	Sphingobacteria	Sphingobacteriales	Flexibacteraceae	Hymenobacter	Hymenobacter actinosclerus (T); 1187; Y17356
			Bacteria	Bacteroidetes	Sphingobacteria	Sphingobacteriales	Flexibacteraceae	Hymenobacter	Hymenobacter aerophilus (T); I/26Cor1; AJ276901
o1419	o1419 16S-Flxbac2	GCGGGGACGACGTCAGG	Bacteria	Firmicutes	Bacilli	Lactobacillales	Leuconostocaceae	Leuconostoc	Fructobacillus pseudoficulneus (T); LC51; AY169967
			Bacteria	Bacteroidetes	Sphingobacteria	Sphingobacteriales	Flexibacteraceae	Hymenobacter	Hymenobacter chitinivorans (T); type strain: Txc1; Y18837
			Bacteria	Bacteroidetes	Sphingobacteria	Sphingobacteriales	Flexibacteraceae	Hymenobacter	Hymenobacter gelipurpurascens (T); type strain: Txg1; Y18836
			Bacteria	Bacteroidetes	Sphingobacteria	Sphingobacteriales	Flexibacteraceae	Hymenobacter	Hymenobacter roseosalivarius; AA688; Y18834
			Bacteria	Bacteroidetes	Sphingobacteria	Sphingobacteriales	Flexibacteraceae	Hymenobacter	Hymenobacter roseosalivarius (T); AA718; Y18833
			Bacteria	Firmicutes	Clostridia	Clostridiales	Ruminococcaceae	Papillibacter	uncultured bacterium; IIIA-3; AJ488093
			Bacteria	Bacteroidetes	Sphingobacteria	Sphingobacteriales	Flexibacteraceae	Hymenobacter	Hymenobacter ocellatus; type strain: Myx 2105; Y18835
			Bacteria	Bacteroidetes	Sphingobacteria	Sphingobacteriales	Flexibacteraceae	Hymenobacter	uncultured Hymenobacter group bacterium; KL-27-1-11; AF408277
			Bacteria	Bacteroidetes	Sphingobacteria	Sphingobacteriales	Flexibacteraceae	Hymenobacter	Taxeobacter ocellatus (T); Txo1; Y18838
			Bacteria	Bacteroidetes	Sphingobacteria	Sphingobacteriales	Flexibacteraceae	Hymenobacter	uncultured Hymenobacter sp.; KL-2-4-2; AF408274
			Bacteria	Bacteroidetes	Sphingobacteria	Sphingobacteriales	Flexibacteraceae	Hymenobacter	uncultured Hymenobacter sp.; KL-2-4-9; AF408275
			Bacteria	Bacteroidetes	Sphingobacteria	Sphingobacteriales	Flexibacteraceae	Hymenobacter	uncultured Hymenobacter group bacterium; KL-2-5-13; AF408273
			Bacteria	Bacteroidetes	Sphingobacteria	Sphingobacteriales	Flexibacteraceae	Hymenobacter	uncultured Hymenobacter group bacterium; KL-53-2-6; AF408270
			Bacteria	Bacteroidetes	Sphingobacteria	Sphingobacteriales	Flexibacteraceae	Hymenobacter	uncultured Taxeobacter sp.; KL-2-5-12; AF408272
			Bacteria	Bacteroidetes	Sphingobacteria	Sphingobacteriales	Flexibacteraceae	Hymenobacter	uncultured Hymenobacter sp.; KL-26-2-11; AF408276
			Bacteria	Bacteroidetes	Sphingobacteria	Sphingobacteriales	Flexibacteraceae	Hymenobacter	Hymenobacter norwichensis (T); type strain: NS/50; AJ549285
			Bacteria	Bacteroidetes	Sphingobacteria	Sphingobacteriales	Flexibacteraceae	Hymenobacter	Antarctic bacterium R-7572; AJ440980
			Bacteria	Bacteroidetes	Sphingobacteria	Sphingobacteriales	Flexibacteraceae	Hymenobacter	Hymenobacter sp. NS/2; AJ549284
			Bacteria	Bacteroidetes	Sphingobacteria	Sphingobacteriales	Flexibacteraceae	Hymenobacter	Hymenobacter actinosclerus (T); 1187; Y17356
			Bacteria	Bacteroidetes	Sphingobacteria	Sphingobacteriales	Flexibacteraceae	Hymenobacter	Hymenobacter aerophilus (T); I/26Cor1; AJ276901
o1420	o1420 16S-FuGroup1	AAGCGCGTCTAGGTGGTTAT	Bacteria	Fusobacteria	Fusobacteria	Fusobacteriales	Fusobacteriaceae	Fusobacterium	Fusobacterium sp. oral clone CY024; AF287809
			Bacteria	Fusobacteria	Fusobacteria	Fusobacteriales	Fusobacteriaceae	Fusobacterium	Fusobacterium sp. oral clone CZ006; AF287810
			Bacteria	Fusobacteria	Fusobacteria	Fusobacteriales	Fusobacteriaceae	Fusobacterium	Fusobacterium sp. oral clone EU021; AF385575
			Bacteria	Fusobacteria	Fusobacteria	Fusobacteriales	Fusobacteriaceae	Fusobacterium	Fusobacterium sp. oral clone AJ050; AF287805
			Bacteria	Fusobacteria	Fusobacteria	Fusobacteriales	Fusobacteriaceae	Fusobacterium	Fusobacterium nucleatum subsp. nucleatum; ATCC 25586(T); AJ133496
			Bacteria	Fusobacteria	Fusobacteria	Fusobacteriales	Fusobacteriaceae	Fusobacterium	Fusobacterium naviforme; DMS 20699, NCTC 11464; AJ006965
			Bacteria	Fusobacteria	Fusobacteria	Fusobacteriales	Fusobacteriaceae	Fusobacterium	Fusobacterium sp. oral clone R002; AF287806
			Bacteria	Fusobacteria	Fusobacteria	Fusobacteriales	Fusobacteriaceae	Fusobacterium	Fusobacterium genomosp. C2; C4AKM033; AY278617
			Bacteria	Fusobacteria	Fusobacteria	Fusobacteriales	Fusobacteriaceae	Fusobacterium	Fusobacterium simiae; M58685
			Bacteria	Fusobacteria	Fusobacteria	Fusobacteriales	Fusobacteriaceae	Fusobacterium	uncultured eubacterium E1-K4; AJ289177
			Bacteria	Fusobacteria	Fusobacteria	Fusobacteriales	Fusobacteriaceae	Fusobacterium	Fusobacterium nucleatum subsp. vincentii ATCC 49256 (T); AJ006964
			Bacteria	Fusobacteria	Fusobacteria	Fusobacteriales	Fusobacteriaceae	Fusobacterium	Fusobacterium nucleatum subsp. polymorphum; ATCC 10953; AF287812
			Bacteria	Fusobacteria	Fusobacteria	Fusobacteriales	Fusobacteriaceae	Fusobacterium	uncultured eubacterium E1-K5; AJ289181
			Bacteria	Fusobacteria	Fusobacteria	Fusobacteriales	Fusobacteriaceae	Fusobacterium	Filifactor alocis (T); ATCC 35896T; X55406
			Bacteria	Fusobacteria	Fusobacteria	Fusobacteriales	Fusobacteriaceae	Fusobacterium	Fusobacterium nucleatum subsp. animalis (T); NCTC 12276T; X55404
			Bacteria	Fusobacteria	Fusobacteria	Fusobacteriales	Fusobacteriaceae	Fusobacterium	Fusobacterium nucleatum subsp. polymorphum (T); ATCC 10953T; X55402
			Bacteria	Fusobacteria	Fusobacteria	Fusobacteriales	Fusobacteriaceae	Fusobacterium	Fusobacterium simiae (T); ATCC 33568T; X55407
			Bacteria	Fusobacteria	Fusobacteria	Fusobacteriales	Fusobacteriaceae	Fusobacterium	Fusobacterium nucleatum subsp. nucleatum (T); ATCC 25586T; X55401
			Bacteria	Fusobacteria	Fusobacteria	Fusobacteriales	Fusobacteriaceae	Fusobacterium	Fusobacterium periodonticum (T); ATCC 33693T; X55405
			Bacteria	Fusobacteria	Fusobacteria	Fusobacteriales	Fusobacteriaceae	Fusobacterium	Fusobacterium nucleatum subsp. fusiforme (T); NCTC 11326T; X55403
o1421	o1421 16S-FuGroup2	ACGCGATAAGTAATCCGCCT	Bacteria	Fusobacteria	Fusobacteria	Fusobacteriales	Fusobacteriaceae	Fusobacterium	Fusobacterium necrophorum; AB; FnS-1; X74407
			Bacteria	Fusobacteria	Fusobacteria	Fusobacteriales	Fusobacteriaceae	Fusobacterium	Fusobacterium necrophorum; B; FnS-40; X74408
			Bacteria	Fusobacteria	Fusobacteria	Fusobacteriales	Fusobacteriaceae	Fusobacterium	Fusobacterium equinum (T); horse; VPB 4027; AJ295750
			Bacteria	Fusobacteria	Fusobacteria	Fusobacteriales	Fusobacteriaceae	Fusobacterium	Fusobacterium nucleatum subsp. nucleatum; ATCC 25586(T); AJ133496
			Bacteria	Fusobacteria	Fusobacteria	Fusobacteriales	Fusobacteriaceae	Fusobacterium	Fusobacterium naviforme; DMS 20699, NCTC 11464; AJ006965
			Bacteria	Fusobacteria	Fusobacteria	Fusobacteriales	Fusobacteriaceae	Fusobacterium	Fusobacterium necrophorum; D4; AF044948
			Bacteria	Fusobacteria	Fusobacteria	Fusobacteriales	Fusobacteriaceae	Fusobacterium	Fusobacterium simiae; M58685
			Bacteria	Fusobacteria	Fusobacteria	Fusobacteriales	Fusobacteriaceae	Fusobacterium	uncultured eubacterium E1-K4; AJ289177
			Bacteria	Fusobacteria	Fusobacteria	Fusobacteriales	Fusobacteriaceae	Fusobacterium	Fusobacterium russii; M58681
			Bacteria	Fusobacteria	Fusobacteria	Fusobacteriales	Fusobacteriaceae	Fusobacterium	uncultured eubacterium E1-K5; AJ289181
			Bacteria	Fusobacteria	Fusobacteria	Fusobacteriales	Fusobacteriaceae	Fusobacterium	Fusobacterium varium (T); M58686
			Bacteria	Fusobacteria	Fusobacteria	Fusobacteriales	Fusobacteriaceae	Fusobacterium	Fusobacterium gonidiaformans; M58679
			Bacteria	Fusobacteria	Fusobacteria	Fusobacteriales	Fusobacteriaceae	Fusobacterium	Fusobacterium perfoetens (T); M58684
			Bacteria	Fusobacteria	Fusobacteria	Fusobacteriales	Fusobacteriaceae	Fusobacterium	Fusobacterium mortiferum; M58680
			Bacteria	Fusobacteria	Fusobacteria	Fusobacteriales	Fusobacteriaceae	Fusobacterium	Filifactor alocis (T); ATCC 35896T; X55406
			Bacteria	Fusobacteria	Fusobacteria	Fusobacteriales	Fusobacteriaceae	Fusobacterium	Fusobacterium gonidiaformans (T); ATCC 25563T; X55410
			Bacteria	Fusobacteria	Fusobacteria	Fusobacteriales	Fusobacteriaceae	Fusobacterium	Fusobacterium nucleatum subsp. polymorphum (T); ATCC 10953T; X55402
			Bacteria	Fusobacteria	Fusobacteria	Fusobacteriales	Fusobacteriaceae	Fusobacterium	Fusobacterium simiae (T); ATCC 33568T; X55407
			Bacteria	Fusobacteria	Fusobacteria	Fusobacteriales	Fusobacteriaceae	Fusobacterium	Fusobacterium russii (T); ATCC 25533T; X55409
			Bacteria	Fusobacteria	Fusobacteria	Fusobacteriales	Fusobacteriaceae	Fusobacterium	Fusobacterium necrogenes (T); ATCC 25556T; X55408
o1423	o1423 16S-FuNuc	TAACTTCGATTTGGGTGGCG	Bacteria	Fusobacteria	Fusobacteria	Fusobacteriales	Fusobacteriaceae	Fusobacterium	Fusobacterium nucleatum subsp. nucleatum ATCC 25586; AE009951
			Bacteria	Fusobacteria	Fusobacteria	Fusobacteriales	Fusobacteriaceae	Fusobacterium	Fusobacterium nucleatum subsp. nucleatum ATCC 25586; AE009951
			Bacteria	Fusobacteria	Fusobacteria	Fusobacteriales	Fusobacteriaceae	Fusobacterium	Fusobacterium nucleatum subsp. nucleatum ATCC 25586; AE009951
			Bacteria	Fusobacteria	Fusobacteria	Fusobacteriales	Fusobacteriaceae	Fusobacterium	Fusobacterium nucleatum subsp. nucleatum ATCC 25586; AE009951
			Bacteria	Fusobacteria	Fusobacteria	Fusobacteriales	Fusobacteriaceae	Fusobacterium	uncultured bacterium; OPEN_ROOT_1; FJ982958
			Bacteria	Fusobacteria	Fusobacteria	Fusobacteriales	Fusobacteriaceae	Fusobacterium	Fusobacterium nucleatum subsp. nucleatum ATCC 25586; AE009951
			Bacteria	Fusobacteria	Fusobacteria	Fusobacteriales	Fusobacteriaceae	Fusobacterium	Fusobacterium nucleatum subsp. nucleatum; ATCC 25586(T); AJ133496
			Bacteria	Fusobacteria	Fusobacteria	Fusobacteriales	Fusobacteriaceae	Fusobacterium	Fusobacterium nucleatum subsp. nucleatum; ATCC25586; M58683
			Bacteria	Fusobacteria	Fusobacteria	Fusobacteriales	Fusobacteriaceae	Fusobacterium	Fusobacterium canifelinum; RMA 12701; Q4; AY162219
			Bacteria	Fusobacteria	Fusobacteria	Fusobacteriales	Fusobacteriaceae	Fusobacterium	Fusobacterium canifelinum; RMA 1036; Q20; AY162221
			Bacteria	Fusobacteria	Fusobacteria	Fusobacteriales	Fusobacteriaceae	Fusobacterium	Fusobacterium canifelinum (T); RMA 12708; Q11; AY162220
			Bacteria	Fusobacteria	Fusobacteria	Fusobacteriales	Fusobacteriaceae	Fusobacterium	Fusobacterium canifelinum; RMA 11693; Q24; AY162222
			Bacteria	Fusobacteria	Fusobacteria	Fusobacteriales	Fusobacteriaceae	Fusobacterium	Fusobacterium nucleatum; ChDC F130; FJ471642
			Bacteria	Fusobacteria	Fusobacteria	Fusobacteriales	Fusobacteriaceae	Fusobacterium	Fusobacterium nucleatum; ChDC F311; FJ471655
			Bacteria	Fusobacteria	Fusobacteria	Fusobacteriales	Fusobacteriaceae	Fusobacterium	Fusobacterium nucleatum; ChDC F317; FJ471659

			Bacteria	Fusobacteria	Fusobacteria	Fusobacteriales	Fusobacteriaceae	Fusobacterium	Fusobacterium nucleatum subsp. nucleatum; ATCC 25586; GQ301038
			Bacteria	Fusobacteria	Fusobacteria	Fusobacteriales	Fusobacteriaceae	Fusobacterium	Fusobacterium nucleatum; ChDC F316; FJ471658
			Bacteria	Fusobacteria	Fusobacteria	Fusobacteriales	Fusobacteriaceae	Fusobacterium	uncultured eubacterium E1-K4; AJ289177
			Bacteria	Fusobacteria	Fusobacteria	Fusobacteriales	Fusobacteriaceae	Fusobacterium	uncultured bacterium; nbu106g09c1; GQ000697
o1424	o1424 16S-Fusbac	GGCCACAAGGGGACTGAG	Bacteria	Fusobacteria	Fusobacteria	Fusobacteriales	Fusobacteriaceae	Fusobacterium	Fusobacterium necrophorum; AB; FnS-1; X74407
			Bacteria	Fusobacteria	Fusobacteria	Fusobacteriales	Fusobacteriaceae	Ilyobacter	Ilyobacter polytropus (T); DSM 2926 T; AJ307981
			Bacteria	Fusobacteria	Fusobacteria	Fusobacteriales	Fusobacteriaceae	Fusobacterium	Fusobacterium necrophorum; B; FnS-40; X74408
			Bacteria	Fusobacteria	Fusobacteria	Fusobacteriales	Fusobacteriaceae	Fusobacterium	Fusobacterium equinum (T); horse; VPB 4027; AJ295750
			Bacteria	Fusobacteria	Fusobacteria	Fusobacteriales	Fusobacteriaceae	Fusobacterium	Fusobacterium nucleatum subsp. nucleatum; ATCC 25586(T); AJ133496
			Bacteria	Fusobacteria	Fusobacteria	Fusobacteriales	Fusobacteriaceae	Fusobacterium	Fusobacterium naviforme; DMS 20699, NCTC 11464; AJ006965
			Bacteria	Fusobacteria	Fusobacteria	Fusobacteriales	Fusobacteriaceae	Fusobacterium	Fusobacterium necrophorum; D4; AF044948
			Bacteria	Fusobacteria	Fusobacteria	Fusobacteriales	Fusobacteriaceae	Fusobacterium	Fusobacterium simiae; M58685
			Bacteria	Fusobacteria	Fusobacteria	Fusobacteriales	Fusobacteriaceae	Fusobacterium	uncultured eubacterium E1-K4; AJ289177
			Bacteria	Fusobacteria	Fusobacteria	Fusobacteriales	Fusobacteriaceae	Fusobacterium	Fusobacterium nucleatum subsp. vincentii ATCC 49256 (T); AJ006964
			Bacteria	Fusobacteria	Fusobacteria	Fusobacteriales	Fusobacteriaceae	Fusobacterium	uncultured eubacterium E1-K5; AJ289181
			Bacteria	Fusobacteria	Fusobacteria	Fusobacteriales	Fusobacteriaceae	Fusobacterium	Fusobacterium varium (T); M58686
			Bacteria	Fusobacteria	Fusobacteria	Fusobacteriales	Fusobacteriaceae	Fusobacterium	Fusobacterium gonidiaformans; M58679
			Bacteria	Fusobacteria	Fusobacteria	Fusobacteriales	Fusobacteriaceae	Fusobacterium	Fusobacterium perfoetens (T); M58684
			Bacteria	Fusobacteria	Fusobacteria	Fusobacteriales	Fusobacteriaceae	Fusobacterium	Fusobacterium mortiferum; M58680
			Bacteria	Fusobacteria	Fusobacteria	Fusobacteriales	Fusobacteriaceae	Fusobacterium	Fusobacterium nucleatum subsp. animalis (T); NCTC 12276T; X55404
			Bacteria	Fusobacteria	Fusobacteria	Fusobacteriales	Fusobacteriaceae	Fusobacterium	Fusobacterium simiae (T); ATCC 33568T; X55407
			Bacteria	Fusobacteria	Fusobacteria	Fusobacteriales	Fusobacteriaceae	Fusobacterium	Fusobacterium mortiferum (T); ATCC 25557T; X55414
			Bacteria	Fusobacteria	Fusobacteria	Fusobacteriales	Fusobacteriaceae	Fusobacterium	Fusobacterium nucleatum subsp. nucleatum (T); ATCC 25586T; X55401
			Bacteria	Fusobacteria	Fusobacteria	Fusobacteriales	Fusobacteriaceae	Fusobacterium	Fusobacterium necrogenes (T); ATCC 25556T; X55408
o1425	o1425 16S-FusGroup	AAAGCGCGTCTAGGTGGTTA	Bacteria	Fusobacteria	Fusobacteria	Fusobacteriales	Fusobacteriaceae	Fusobacterium	Fusobacterium sp. oral clone CY024; AF287809
			Bacteria	Fusobacteria	Fusobacteria	Fusobacteriales	Fusobacteriaceae	Fusobacterium	Fusobacterium sp. oral clone CZ006; AF287810
			Bacteria	Fusobacteria	Fusobacteria	Fusobacteriales	Fusobacteriaceae	Fusobacterium	Fusobacterium sp. oral clone EU021; AF385575
			Bacteria	Fusobacteria	Fusobacteria	Fusobacteriales	Fusobacteriaceae	Fusobacterium	Fusobacterium sp. oral clone AJ050; AF287805
			Bacteria	Fusobacteria	Fusobacteria	Fusobacteriales	Fusobacteriaceae	Fusobacterium	Fusobacterium nucleatum subsp. nucleatum; ATCC 25586(T); AJ133496
			Bacteria	Fusobacteria	Fusobacteria	Fusobacteriales	Fusobacteriaceae	Fusobacterium	Fusobacterium naviforme; DMS 20699, NCTC 11464; AJ006965
			Bacteria	Fusobacteria	Fusobacteria	Fusobacteriales	Fusobacteriaceae	Fusobacterium	Fusobacterium sp. oral clone R002; AF287806
			Bacteria	Fusobacteria	Fusobacteria	Fusobacteriales	Fusobacteriaceae	Fusobacterium	Fusobacterium genomosp. C2; C4AKM033; AY278617
			Bacteria	Fusobacteria	Fusobacteria	Fusobacteriales	Fusobacteriaceae	Fusobacterium	Fusobacterium simiae; M58685
			Bacteria	Fusobacteria	Fusobacteria	Fusobacteriales	Fusobacteriaceae	Fusobacterium	uncultured eubacterium E1-K4; AJ289177
			Bacteria	Fusobacteria	Fusobacteria	Fusobacteriales	Fusobacteriaceae	Fusobacterium	Fusobacterium nucleatum subsp. vincentii ATCC 49256 (T); AJ006964
			Bacteria	Fusobacteria	Fusobacteria	Fusobacteriales	Fusobacteriaceae	Fusobacterium	Fusobacterium nucleatum subsp. polymorphum; ATCC 10953; AF287812
			Bacteria	Fusobacteria	Fusobacteria	Fusobacteriales	Fusobacteriaceae	Fusobacterium	uncultured eubacterium E1-K5; AJ289181
			Bacteria	Fusobacteria	Fusobacteria	Fusobacteriales	Fusobacteriaceae	Fusobacterium	Filifactor alocis (T); ATCC 35896T; X55406
			Bacteria	Fusobacteria	Fusobacteria	Fusobacteriales	Fusobacteriaceae	Fusobacterium	Fusobacterium nucleatum subsp. animalis (T); NCTC 12276T; X55404
			Bacteria	Fusobacteria	Fusobacteria	Fusobacteriales	Fusobacteriaceae	Fusobacterium	Fusobacterium nucleatum subsp. polymorphum (T); ATCC 10953T; X55402
			Bacteria	Fusobacteria	Fusobacteria	Fusobacteriales	Fusobacteriaceae	Fusobacterium	Fusobacterium simiae (T); ATCC 33568T; X55407
			Bacteria	Fusobacteria	Fusobacteria	Fusobacteriales	Fusobacteriaceae	Fusobacterium	Fusobacterium nucleatum subsp. nucleatum (T); ATCC 25586T; X55401
			Bacteria	Fusobacteria	Fusobacteria	Fusobacteriales	Fusobacteriaceae	Fusobacterium	Fusobacterium periodonticum (T); ATCC 33693T; X55405
			Bacteria	Fusobacteria	Fusobacteria	Fusobacteriales	Fusobacteriaceae	Fusobacterium	Fusobacterium nucleatum subsp. fusiforme (T); NCTC 11326T; X55403
o1426	o1426 16S-Gaprot	TGTAGAGGGGGGTAGAATT	Bacteria	Proteobacteria	Gammaproteobacteria	Alteromonadales	Shewanellaceae	Shewanella	Shewanella algae; Bry; X81621
			Bacteria	Proteobacteria	Gammaproteobacteria	Alteromonadales	Shewanellaceae	Shewanella	Shewanella frigidimarina; NCIMB 400; Y13699
			Bacteria	Proteobacteria	Gammaproteobacteria	Alteromonadales	Shewanellaceae	Shewanella	Shewanella baltica; OS195; AJ000216
			Bacteria	Proteobacteria	Gammaproteobacteria	Alteromonadales	Shewanellaceae	Shewanella	Shewanella baltica (T); NCTC 10735; AJ000214
			Bacteria	Proteobacteria	Gammaproteobacteria	Alteromonadales	Shewanellaceae	Shewanella	Shewanella baltica; OS155; AJ000215
			Bacteria	Proteobacteria	Gammaproteobacteria	Alteromonadales	Shewanellaceae	Shewanella	Shewanella putrefaciens IR-1; AF170300
			Bacteria	Proteobacteria	Gammaproteobacteria	Alteromonadales	Shewanellaceae	Shewanella	Shewanella sp.; 16.k; NB1-a; AB013822
			Bacteria	Proteobacteria	Gammaproteobacteria	Alteromonadales	Shewanellaceae	Shewanella	Shewanella algae; FeRed; X81622
			Bacteria	Proteobacteria	Gammaproteobacteria	Alteromonadales	Shewanellaceae	Shewanella	Shewanella massilia; AJ006084
			Bacteria	Proteobacteria	Gammaproteobacteria	Alteromonadales	Shewanellaceae	Shewanella	Shewanella putrefaciens; AB057660
			Bacteria	Proteobacteria	Gammaproteobacteria	Alteromonadales	Shewanellaceae	Shewanella	Shewanella putrefaciens; LMG 26268 T; X81623
			Bacteria	Proteobacteria	Gammaproteobacteria	Alteromonadales	Shewanellaceae	Shewanella	Shewanella sp. SG133; AB038035
			Bacteria	Proteobacteria	Gammaproteobacteria	Alteromonadales	Shewanellaceae	Shewanella	Vibrio sp. OS53; AB038028
			Bacteria	Proteobacteria	Gammaproteobacteria	Alteromonadales	Shewanellaceae	Shewanella	unidentified bacterium; Aspo5; X95233
			Bacteria	Proteobacteria	Gammaproteobacteria	Alteromonadales	Shewanellaceae	Shewanella	polar sea bacterium R7216; AJ295714
			Bacteria	Proteobacteria	Gammaproteobacteria	Alteromonadales	Shewanellaceae	Shewanella	Shewanella putrefaciens; LMG 2369; AJ000213
			Bacteria	Proteobacteria	Gammaproteobacteria	Alteromonadales	Shewanellaceae	Shewanella	Shewanella benthica (T); ATCC 43992; X82131
			Bacteria	Proteobacteria	Gammaproteobacteria	Alteromonadales	Shewanellaceae	Shewanella	Shewanella hanedai; CIP 103207T; X82132
			Bacteria	Proteobacteria	Gammaproteobacteria	Alteromonadales	Shewanellaceae	Shewanella	Shewanella putrefaciens (T); ATCC 8071; X82133
			Bacteria	Proteobacteria	Gammaproteobacteria	Alteromonadales	Shewanellaceae	Shewanella	Shewanella putrefaciens; SMCC 576W (CN-32); AF136269
o1427	o1427 16S-GemGroup1	AAGAGATCAGTGCTGCAGCT	Bacteria	Firmicutes	Bacilli	Bacillales	Incertae Sedis XI	Gemella	uncultured Gemella sp.; DSD10; AY672072
			Bacteria	Firmicutes	Bacilli	Bacillales	Incertae Sedis XI	Gemella	Gemella morbillorum (T); L14327
			Bacteria	Firmicutes	Bacilli	Bacillales	Incertae Sedis XI	Gemella	Gemella sp. oral strain C24KA; AY005051
			Bacteria	Firmicutes	Bacilli	Bacillales	Incertae Sedis XI	Gemella	Gemella haemolysans (T); L14326
			Bacteria	Firmicutes	Bacilli	Bacillales	Incertae Sedis XI	Gemella	uncultured bacterium; rRNA283; AY959056
			Bacteria	Firmicutes	Bacilli	Bacillales	Incertae Sedis XI	Gemella	uncultured bacterium; rRNA131; AY958904
			Bacteria	Firmicutes	Bacilli	Bacillales	Incertae Sedis XI	Gemella	uncultured bacterium; rRNA374; AY959147
			Bacteria	Firmicutes	Bacilli	Bacillales	Incertae Sedis XI	Gemella	uncultured bacterium; rRNA241; AY959014
			Bacteria	Firmicutes	Bacilli	Bacillales	Incertae Sedis XI	Gemella	uncultured bacterium; rRNA304; AY959077
			Bacteria	Firmicutes	Bacilli	Bacillales	Incertae Sedis XI	Gemella	Gemella sp. oral clone ASCE02; AY923133
			Bacteria	Firmicutes	Bacilli	Bacillales	Incertae Sedis XI	Gemella	Gemella sanguinis (T); 2045-94; Y13364
			Bacteria	Firmicutes	Bacilli	Bacillales	Incertae Sedis XI	Gemella	Gemella sp. 933-88; Y13366
			Bacteria	Firmicutes	Bacilli	Bacillales	Incertae Sedis XI	Gemella	Gemella haemolysans; mother V4; 12V4; AM157450
			Bacteria	Firmicutes	Bacilli	Bacillales	Incertae Sedis XI	Gemella	Gemella sp. oral clone ASCF04; AY923139
			Bacteria	Firmicutes	Bacilli	Bacillales	Incertae Sedis XI	Gemella	uncultured bacterium; rRNA155; AY958928
			Bacteria	Firmicutes	Bacilli	Bacillales	Incertae Sedis XI	Gemella	Gemella haemolysans; M58799
			Bacteria	Firmicutes	Bacilli	Bacillales	Incertae Sedis XI	Gemella	uncultured bacterium; Z418; AY979245
			Bacteria	Firmicutes	Bacilli	Bacillales	Incertae Sedis XI	Gemella	uncultured bacterium; MU89; AY977299
			Bacteria	Firmicutes	Bacilli	Bacillales	Incertae Sedis XI	Gemella	uncultured bacterium; NP80; AY983680
			Bacteria	Firmicutes	Bacilli	Bacillales	Incertae Sedis XI	Gemella	Gemella sanguinis; MC-1; AY757357
o1428	o1428 16S-GemGroup2	AAAGAGATCAGTGCTGCAGC	Bacteria	Firmicutes	Bacilli	Bacillales	Incertae Sedis XI	Gemella	Gemella morbillorum (T); L14327
			Bacteria	Firmicutes	Bacilli	Bacillales	Incertae Sedis XI	Gemella	Gemella sp. oral strain C24KA; AY005051
			Bacteria	Firmicutes	Bacilli	Bacillales	Incertae Sedis XI	Gemella	Gemella haemolysans (T); L14326
			Bacteria	Firmicutes	Bacilli	Bacillales	Incertae Sedis XI	Gemella	uncultured bacterium; rRNA283; AY959056

			Bacteria	Firmicutes	Bacilli	Bacillales	Incertae Sedis XI	Gemella	uncultured bacterium; rRNA131; AY958904
			Bacteria	Firmicutes	Bacilli	Bacillales	Incertae Sedis XI	Gemella	uncultured bacterium; rRNA374; AY959147
			Bacteria	Firmicutes	Bacilli	Bacillales	Incertae Sedis XI	Gemella	uncultured bacterium; rRNA241; AY959014
			Bacteria	Firmicutes	Bacilli	Bacillales	Incertae Sedis XI	Gemella	uncultured bacterium; rRNA304; AY959077
			Bacteria	Firmicutes	Bacilli	Bacillales	Incertae Sedis XI	Gemella	Gemella sanguinis (T); 2045-94; Y13364
			Bacteria	Firmicutes	Bacilli	Bacillales	Incertae Sedis XI	Gemella	Gemella sp. 933-88; Y13366
			Bacteria	Firmicutes	Bacilli	Bacillales	Incertae Sedis XI	Gemella	Gemella sp. 1754-94; Y13363
			Bacteria	Firmicutes	Bacilli	Bacillales	Incertae Sedis XI	Gemella	uncultured bacterium; rRNA155; AY958928
			Bacteria	Firmicutes	Bacilli	Bacillales	Incertae Sedis XI	Gemella	Gemella bergeri (T); 617-93; Y13365
			Bacteria	Firmicutes	Bacilli	Bacillales	Incertae Sedis XI	Gemella	Gemella sp. oral strain A31SC; AY005050
			Bacteria	Firmicutes	Bacilli	Bacillales	Incertae Sedis XI	Gemella	Gemella sp. M60449/99/1; M60449/99/1 T; AJ251987
			Bacteria	Firmicutes	Bacilli	Bacillales	Incertae Sedis XI	Gemella	Gemella haemolysans; M58799
			Bacteria	Firmicutes	Bacilli	Bacillales	Incertae Sedis XI	Gemella	uncultured bacterium; Z418; AY979245
			Bacteria	Firmicutes	Bacilli	Bacillales	Incertae Sedis XI	Gemella	uncultured bacterium; MU89; AY977299
			Bacteria	Firmicutes	Bacilli	Bacillales	Incertae Sedis XI	Gemella	uncultured bacterium; NP80; AY983680
			Bacteria	Firmicutes	Bacilli	Bacillales	Incertae Sedis XI	Gemella	Gemella sanguinis; MC-1; AY757357
o1429	o1429 16S-GraGroup1	CGGAAACGGATGCTAAAACC	Bacteria	Firmicutes	Bacilli	Lactobacillales	Carnobacteriaceae	Granulicatella	Granulicatella sp. oral clone ASCA05; DQ341469
			Bacteria	Firmicutes	Bacilli	Lactobacillales	Carnobacteriaceae	Granulicatella	Granulicatella elegans (T); DSM 11693; B1333; AF016390
			Bacteria	Firmicutes	Bacilli	Lactobacillales	Carnobacteriaceae	Granulicatella	Granulicatella sp. oral clone ASCG05; AY923146
			Bacteria	Firmicutes	Bacilli	Lactobacillales	Carnobacteriaceae	Granulicatella	Granulicatella adiacens; 04356101; AY879304
			Bacteria	Firmicutes	Bacilli	Lactobacillales	Carnobacteriaceae	Granulicatella	Granulicatella adiacens; 02357001; AY879302
			Bacteria	Firmicutes	Bacilli	Lactobacillales	Carnobacteriaceae	Granulicatella	Granulicatella adiacens; 04424062; AY879305
			Bacteria	Firmicutes	Bacilli	Lactobacillales	Carnobacteriaceae	Granulicatella	Granulicatella adiacens; 03385274; AY879300
			Bacteria	Firmicutes	Bacilli	Lactobacillales	Carnobacteriaceae	Granulicatella	Granulicatella adiacens; 04305056; AY879303
			Bacteria	Firmicutes	Bacilli	Lactobacillales	Carnobacteriaceae	Granulicatella	Granulicatella adiacens; 03252090; AY879299
			Bacteria	Firmicutes	Bacilli	Lactobacillales	Carnobacteriaceae	Granulicatella	Granulicatella para-adiacens; 03476079; AY879301
			Bacteria	Firmicutes	Bacilli	Lactobacillales	Carnobacteriaceae	Granulicatella	Granulicatella para-adiacens; 04401102; AY879298
			Bacteria	Firmicutes	Bacilli	Lactobacillales	Carnobacteriaceae	Granulicatella	Granulicatella adiacens; 10/833; AJ312375
			Bacteria	Firmicutes	Bacilli	Lactobacillales	Carnobacteriaceae	Granulicatella	Granulicatella elegans; 4067-96; Y15413
			Bacteria	Firmicutes	Bacilli	Lactobacillales	Carnobacteriaceae	Granulicatella	uncultured organism; MC0604c8; AY897990
			Bacteria	Firmicutes	Bacilli	Lactobacillales	Carnobacteriaceae	Granulicatella	Abiotrophia para-adiacens; TKT1; AB022027
			Bacteria	Firmicutes	Bacilli	Lactobacillales	Carnobacteriaceae	Granulicatella	Granulicatella elegans; HHC5; AB022026
			Bacteria	Firmicutes	Bacilli	Lactobacillales	Carnobacteriaceae	Granulicatella	uncultured bacterium; LC1022b35; DQ228568
			Bacteria	Firmicutes	Bacilli	Lactobacillales	Carnobacteriaceae	Granulicatella	Granulicatella adiacens (T); GIFU12706; D50540
			Bacteria	Firmicutes	Bacilli	Lactobacillales	Carnobacteriaceae	Granulicatella	Granulicatella elegans; 2640-91; Y15408
			Bacteria	Firmicutes	Bacilli	Lactobacillales	Carnobacteriaceae	Granulicatella	Abiotrophia sp. oral clone OH2A; AY947496
o1430	o1430 16S-GraGroup2	CCATCAGAGGGGGATAACAT	Bacteria	Firmicutes	Bacilli	Lactobacillales	Carnobacteriaceae	Granulicatella	Granulicatella sp. oral clone ASCA05; DQ341469
			Bacteria	Firmicutes	Bacilli	Lactobacillales	Carnobacteriaceae	Granulicatella	Granulicatella elegans (T); DSM 11693; B1333; AF016390
			Bacteria	Firmicutes	Bacilli	Lactobacillales	Carnobacteriaceae	Granulicatella	Granulicatella sp. oral clone ASCG05; AY923146
			Bacteria	Firmicutes	Bacilli	Lactobacillales	Carnobacteriaceae	Granulicatella	Granulicatella adiacens; 04356101; AY879304
			Bacteria	Firmicutes	Bacilli	Lactobacillales	Carnobacteriaceae	Granulicatella	Granulicatella adiacens; 02357001; AY879302
			Bacteria	Firmicutes	Bacilli	Lactobacillales	Carnobacteriaceae	Granulicatella	Granulicatella adiacens; 04424062; AY879305
			Bacteria	Firmicutes	Bacilli	Lactobacillales	Carnobacteriaceae	Granulicatella	Granulicatella adiacens; 03385274; AY879300
			Bacteria	Firmicutes	Bacilli	Lactobacillales	Carnobacteriaceae	Granulicatella	Granulicatella adiacens; 04305056; AY879303
			Bacteria	Firmicutes	Bacilli	Lactobacillales	Carnobacteriaceae	Granulicatella	Granulicatella sp. oral clone ASCB09; AY953251
			Bacteria	Firmicutes	Bacilli	Lactobacillales	Carnobacteriaceae	Granulicatella	Granulicatella adiacens; 03252090; AY879299
			Bacteria	Firmicutes	Bacilli	Lactobacillales	Carnobacteriaceae	Granulicatella	Granulicatella para-adiacens; 03476079; AY879301
			Bacteria	Firmicutes	Bacilli	Lactobacillales	Carnobacteriaceae	Granulicatella	Granulicatella para-adiacens; 04401102; AY879298
			Bacteria	Firmicutes	Bacilli	Lactobacillales	Carnobacteriaceae	Granulicatella	Granulicatella adiacens; 10/833; AJ312375
			Bacteria	Firmicutes	Bacilli	Lactobacillales	Carnobacteriaceae	Granulicatella	Granulicatella elegans; 4067-96; Y15413
			Bacteria	Firmicutes	Bacilli	Lactobacillales	Carnobacteriaceae	Granulicatella	uncultured organism; MC0604c8; AY897990
			Bacteria	Firmicutes	Bacilli	Lactobacillales	Carnobacteriaceae	Granulicatella	Abiotrophia para-adiacens; TKT1; AB022027
			Bacteria	Firmicutes	Bacilli	Lactobacillales	Carnobacteriaceae	Granulicatella	Granulicatella elegans; HHC5; AB022026
			Bacteria	Firmicutes	Bacilli	Lactobacillales	Carnobacteriaceae	Granulicatella	uncultured bacterium; LC1022b35; DQ228568
			Bacteria	Firmicutes	Bacilli	Lactobacillales	Carnobacteriaceae	Granulicatella	Granulicatella elegans; 2640-91; Y15408
			Bacteria	Firmicutes	Bacilli	Lactobacillales	Carnobacteriaceae	Granulicatella	Abiotrophia sp. oral clone OH2A; AY947496
o1431	o1431 16S-HaeAct	CAGAGATGGGTTTGTGCCTT	Bacteria	Proteobacteria	Gammaproteobacteria	Pasteurellales	Pasteurellaceae	Aggregatibacter	uncultured bacterium; OPEN_PLAQUE_8; FJ983034
			Bacteria	Proteobacteria	Gammaproteobacteria	Enterobacteriales	Enterobacteriaceae	Shigella	uncultured Shigella sp.; 538a; EU644470
			Bacteria	Proteobacteria	Gammaproteobacteria	Pasteurellales	Pasteurellaceae	Aggregatibacter	uncultured Actinobacillus sp.; 402F06(oral); AM420150
			Bacteria	Proteobacteria	Gammaproteobacteria	Pasteurellales	Pasteurellaceae	Aggregatibacter	uncultured Actinobacillus sp.; 402B07(oral); AM420138
			Bacteria	Proteobacteria	Gammaproteobacteria	Pasteurellales	Pasteurellaceae	Aggregatibacter	Aggregatibacter actinomycetemcomitans D11S-1; CP001733
			Bacteria	Proteobacteria	Gammaproteobacteria	Pasteurellales	Pasteurellaceae	Aggregatibacter	Aggregatibacter actinomycetemcomitans D11S-1; CP001733
			Bacteria	Proteobacteria	Gammaproteobacteria	Pasteurellales	Pasteurellaceae	Aggregatibacter	Aggregatibacter actinomycetemcomitans D11S-1; CP001733
			Bacteria	Proteobacteria	Gammaproteobacteria	Pasteurellales	Pasteurellaceae	Aggregatibacter	Aggregatibacter actinomycetemcomitans D11S-1; CP001733
			Bacteria	Proteobacteria	Gammaproteobacteria	Pasteurellales	Pasteurellaceae	Aggregatibacter	Aggregatibacter actinomycetemcomitans D11S-1; CP001733
			Bacteria	Proteobacteria	Gammaproteobacteria	Pasteurellales	Pasteurellaceae	Aggregatibacter	Aggregatibacter actinomycetemcomitans D11S-1; CP001733
			Bacteria	Proteobacteria	Gammaproteobacteria	Pasteurellales	Pasteurellaceae	Aggregatibacter	Aggregatibacter actinomycetemcomitans; M75035
			Bacteria	Proteobacteria	Gammaproteobacteria	Pasteurellales	Pasteurellaceae	Aggregatibacter	Aggregatibacter actinomycetemcomitans (T); M75039
			Bacteria	Proteobacteria	Gammaproteobacteria	Pasteurellales	Pasteurellaceae	Aggregatibacter	Aggregatibacter actinomycetemcomitans; M75037
			Bacteria	Proteobacteria	Gammaproteobacteria	Pasteurellales	Pasteurellaceae	Aggregatibacter	Aggregatibacter actinomycetemcomitans; M75036
			Bacteria	Proteobacteria	Gammaproteobacteria	Pasteurellales	Pasteurellaceae	Aggregatibacter	Aggregatibacter actinomycetemcomitans; M75038
			Bacteria	Proteobacteria	Gammaproteobacteria	Pasteurellales	Pasteurellaceae	Aggregatibacter	Aggregatibacter actinomycetemcomitans; ATCC 43718; AY362884
			Bacteria	Proteobacteria	Gammaproteobacteria	Pasteurellales	Pasteurellaceae	Aggregatibacter	uncultured Actinobacillus sp.; 603007(oral); AM420240
			Bacteria	Proteobacteria	Gammaproteobacteria	Pasteurellales	Pasteurellaceae	Aggregatibacter	Aggregatibacter actinomycetemcomitans; ATCC 29523; AY362885
o1432	o1432 16S-HaeGroup	GACGAGGAAGGTTGTTGTGT	Bacteria	Proteobacteria	Gammaproteobacteria	Pasteurellales	Pasteurellaceae	unclassified_Pasteurellaceae	Haemophilus sp. oral clone JM053; AY349380
			Bacteria	Proteobacteria	Gammaproteobacteria	Pasteurellales	Pasteurellaceae	Aggregatibacter	uncultured bacterium; P1D1-564; EF511945
			Bacteria	Proteobacteria	Gammaproteobacteria	Pasteurellales	Pasteurellaceae	unclassified_Pasteurellaceae	uncultured bacterium; P1D1-738; EF511870
			Bacteria	Proteobacteria	Gammaproteobacteria	Pasteurellales	Pasteurellaceae	unclassified_Pasteurellaceae	uncultured bacterium; P1D1-757; EF511871
			Bacteria	Proteobacteria	Gammaproteobacteria	Pasteurellales	Pasteurellaceae	unclassified_Pasteurellaceae	uncultured bacterium; P1D1-686; EF511881
			Bacteria	Proteobacteria	Gammaproteobacteria	Pasteurellales	Pasteurellaceae	Aggregatibacter	uncultured bacterium; P1D1-515; EF511973
			Bacteria	Proteobacteria	Gammaproteobacteria	Pasteurellales	Pasteurellaceae	Aggregatibacter	uncultured bacterium; P1D1-675; EF511981
			Bacteria	Proteobacteria	Gammaproteobacteria	Pasteurellales	Pasteurellaceae	Aggregatibacter	uncultured bacterium; P1D1-575; EF512002
			Bacteria	Proteobacteria	Gammaproteobacteria	Pasteurellales	Pasteurellaceae	unclassified_Pasteurellaceae	uncultured bacterium; P1D1-677; EF511865
			Bacteria	Proteobacteria	Gammaproteobacteria	Pasteurellales	Pasteurellaceae	Aggregatibacter	uncultured bacterium; P1D1-552; EF511863
			Bacteria	Proteobacteria	Gammaproteobacteria	Pasteurellales	Pasteurellaceae	Aggregatibacter	uncultured bacterium; P1D1-522; EF511890
			Bacteria	Proteobacteria	Gammaproteobacteria	Pasteurellales	Pasteurellaceae	Aggregatibacter	uncultured bacterium; P1D1-559; EF511880
			Bacteria	Proteobacteria	Gammaproteobacteria	Pasteurellales	Pasteurellaceae	Aggregatibacter	Haemophilus parainfluenzae; ATCC 7901; M75040
			Bacteria	Proteobacteria	Gammaproteobacteria	Pasteurellales	Pasteurellaceae	Aggregatibacter	Aggregatibacter aphrophilus (T); M75041

			Bacteria	Proteobacteria	Gammaproteobacteria	Pasteurellales	Pasteurellaceae	unclassified_Pasteurellaceae	Aggregatibacter aphrophilus (T); M75042
			Bacteria	Proteobacteria	Gammaproteobacteria	Pasteurellales	Pasteurellaceae	Aggregatibacter	Aggregatibacter aphrophilus; CCUG 3715; AY362906
			Bacteria	Proteobacteria	Gammaproteobacteria	Pasteurellales	Pasteurellaceae	Aggregatibacter	Aggregatibacter aphrophilus; 218631; AY365451
			Bacteria	Proteobacteria	Gammaproteobacteria	Pasteurellales	Pasteurellaceae	Aggregatibacter	Aggregatibacter aphrophilus; 6757; AY365453
			Bacteria	Proteobacteria	Gammaproteobacteria	Pasteurellales	Pasteurellaceae	Aggregatibacter	Aggregatibacter aphrophilus; 94171; AY365449
			Bacteria	Proteobacteria	Gammaproteobacteria	Pasteurellales	Pasteurellaceae	unclassified_Pasteurellaceae	uncultured Haemophilus sp.; ASCB07; DQ366688
o1433	o1433 16S-HaeOral	GACGAGGAAGGTTGGTATGT	Bacteria	Proteobacteria	Gammaproteobacteria	Pasteurellales	Pasteurellaceae	Actinobacillus	Haemophilus sp. oral clone BJ021; AY005034
			Bacteria	Proteobacteria	Gammaproteobacteria	Pasteurellales	Pasteurellaceae	Actinobacillus	uncultured Haemophilus sp.; EHFS1_S12a; EU071511
			Bacteria	Proteobacteria	Gammaproteobacteria	Pasteurellales	Pasteurellaceae	Actinobacillus	uncultured bacterium; nbw691d07c1; GQ116320
			Bacteria	Proteobacteria	Gammaproteobacteria	Pasteurellales	Pasteurellaceae	Actinobacillus	uncultured bacterium; nbw691d08c1; GQ116321
o1434	o1434 16S-Halan1	TCCCGCCTGGGGATTACGA	Bacteria	Firmicutes	Clostridia	Halanaerobiales	Halanaerobiaceae	Halanaerobium	Halanaerobium acetethylicum; DSM 3532; X89071
			Bacteria	Firmicutes	Clostridia	Halanaerobiales	Halobacteroidaceae	Orenia	Orenia marismortui (T); DSM 5156; X89073
			Bacteria	Firmicutes	Clostridia	Halanaerobiales	Halanaerobiaceae	Halanaerobium	Halanaerobium saccharolyticum subsp. saccharolyticum (T); DSM 6643; X89069
			Bacteria	Firmicutes	Clostridia	Halanaerobiales	Halanaerobiaceae	Halanaerobium	Halanaerobium saccharolyticum subsp. senegalense (T); DSM 7379; X89070
			Bacteria	Firmicutes	Clostridia	Halanaerobiales	Halobacteroidaceae	Acetohalobium	Acetohalobium arabaticum (T); DSM 5501; X89077
			Bacteria	Firmicutes	Clostridia	Halanaerobiales	Halobacteroidaceae	Halobacteroides	Halobacteroides halobius; DSM 5150; X89074
			Bacteria	Firmicutes	Clostridia	Halanaerobiales	Halanaerobiaceae	Halanaerobium	Halanaerobium sp. KT-2/3-3; AJ309519
			Bacteria	Firmicutes	Clostridia	Halanaerobiales	Halanaerobiaceae	Halanaerobium	Halanaerobium sp. KT-8-13; AJ309520
			Bacteria	Firmicutes	Clostridia	Halanaerobiales	Halobacteroidaceae	Halanaerobacter	Halanaerobacter chitinivorans; OGC229; X89076
			Bacteria	Firmicutes	Clostridia	Halanaerobiales	Halobacteroidaceae	Halanaerobacter	Halanaerobacter lacunarum (T); DSM 6640; X89075
			Bacteria	Firmicutes	Clostridia	Halanaerobiales	Halanaerobiaceae	Halanaerobium	Halanaerobium praevalens; S-8; AB022035
			Bacteria	Firmicutes	Clostridia	Halanaerobiales	Halanaerobiaceae	Halanaerobium	Halanaerobium praevalens; DSM 2228; AB022034
			Bacteria	Firmicutes	Clostridia	Halanaerobiales	Halanaerobiaceae	Halanaerobium	Halanaerobium fermentans (T); R-9; AB023308
			Bacteria	Firmicutes	Clostridia	Halanaerobiales	Halanaerobiaceae	Halanaerobium	Red Sea bacterium KT-2K28; AJ309525
			Bacteria	Firmicutes	Clostridia	Halanaerobiales	Halobacteroidaceae	Orenia	Orenia sivashensis (T); Z-7191 (T); AF152595
			Bacteria	Firmicutes	Clostridia	Halanaerobiales	Halanaerobiaceae	Halanaerobium	Red Sea bacterium KT-2K23; AJ309524
			Bacteria	Firmicutes	Clostridia	Halanaerobiales	Halobacteroidaceae	Halanaerobacter	Halanaerobacter salinarius (T); SG3903; Y14212
			Bacteria	Firmicutes	Clostridia	Halanaerobiales	Halobacteroidaceae	Halobacteroides	Halobacteroides elegans; Z-7287; AJ238119
			Bacteria	Firmicutes	Clostridia	Halanaerobiales	Halobacteroidaceae	Selenihalanaerobacter	Selenihalanaerobacter shriftii (T); DSSE-1; ATCC BAA-73; AF310247
			Bacteria	Firmicutes	Clostridia	Halanaerobiales	Halanaerobiaceae	Halanaerobium	Halanaerobium sp. S5L4; AJ309521
o1435	o1435 16S-Halan2	ACTAGGTGTTGGAGGTTC	Bacteria	Firmicutes	Clostridia	Halanaerobiales	Halanaerobiaceae	Halothermothrix	Halothermothrix orenii (T); L22016
			Bacteria	Firmicutes	Clostridia	Halanaerobiales	Halanaerobiaceae	Halanaerobium	Halanaerobium acetethylicum; DSM 3532; X89071
			Bacteria	Firmicutes	Clostridia	Halanaerobiales	Halobacteroidaceae	Orenia	Orenia marismortui (T); DSM 5156; X89073
			Bacteria	Firmicutes	Clostridia	Halanaerobiales	Halanaerobiaceae	Halanaerobium	Halanaerobium saccharolyticum subsp. saccharolyticum (T); DSM 6643; X89069
			Bacteria	Firmicutes	Clostridia	Halanaerobiales	Halanaerobiaceae	Halanaerobium	Halanaerobium saccharolyticum subsp. senegalense (T); DSM 7379; X89070
			Bacteria	Firmicutes	Clostridia	Halanaerobiales	Halanaerobiaceae	Halocella	Halocella cellulolsilytica; DSM 7362; X89072
			Bacteria	Firmicutes	Clostridia	Halanaerobiales	Halobacteroidaceae	Halobacteroides	Halobacteroides halobius; DSM 5150; X89074
			Bacteria	Firmicutes	Clostridia	Halanaerobiales	Halanaerobiaceae	Halanaerobium	Halanaerobium sp. KT-8-13; AJ309520
			Bacteria	Firmicutes	Clostridia	Halanaerobiales	Halanaerobiaceae	Halanaerobium	Halanaerobium salsuginis (T); VS-752; L22890
			Bacteria	Firmicutes	Clostridia	Halanaerobiales	Halanaerobiaceae	Halanaerobium	Halanaerobium praevalens; S-8; AB022035
			Bacteria	Firmicutes	Clostridia	Halanaerobiales	Halanaerobiaceae	Halanaerobium	Halanaerobium praevalens; DSM 2228; AB022034
			Bacteria	Firmicutes	Clostridia	Halanaerobiales	Halanaerobiaceae	Halanaerobium	Halanaerobium fermentans (T); R-9; AB023308
			Bacteria	Firmicutes	Clostridia	Halanaerobiales	Halanaerobiaceae	Halanaerobium	Red Sea bacterium KT-2K28; AJ309525
			Bacteria	Firmicutes	Clostridia	Halanaerobiales	Halobacteroidaceae	Orenia	Orenia sivashensis (T); Z-7191 (T); AF152595
			Bacteria	Firmicutes	Clostridia	Halanaerobiales	Halanaerobiaceae	Halanaerobium	Red Sea bacterium KT-2K23; AJ309524
			Bacteria	Firmicutes	Clostridia	Halanaerobiales	Halobacteroidaceae	Selenihalanaerobacter	Selenihalanaerobacter shriftii (T); DSSE-1; ATCC BAA-73; AF310247
			Bacteria	Firmicutes	Clostridia	Halanaerobiales	Halanaerobiaceae	Halanaerobium	Halanaerobium saccharolyticum subsp. saccharolyticum; Z-7787; L37424
			Bacteria	Firmicutes	Clostridia	Halanaerobiales	Halanaerobiaceae	Halanaerobium	Halanaerobium sp. S5L4; AJ309521
			Bacteria	Firmicutes	Clostridia	Halanaerobiales	Halobacteroidaceae	Orenia	Orenia salinaria (T); SG3902; Y18485
			Bacteria	Firmicutes	Clostridia	Halanaerobiales	Halobacteroidaceae	Halonatronum	Halonatronum saccharophilum (T); Z-7986; AY014858
o1436	o1436 16S-HalLit	AAGTACCGTGCGAATAGAGC	Bacteria	Firmicutes	Bacilli	Bacillales	Bacillaceae	Halobacillus	Halobacillus sp. YIM-kkny2; AY121429
			Bacteria	Firmicutes	Bacilli	Bacillales	Bacillaceae	Halobacillus	Halobacillus litoralis (T); SL-4; X94558
			Bacteria	Firmicutes	Bacilli	Bacillales	Bacillaceae	Halobacillus	Halobacillus sp. IS-Hb6; AB189300
			Bacteria	Firmicutes	Bacilli	Bacillales	Bacillaceae	Halobacillus	Halobacillus trueperi (T); DSM 10404T; AJ310149
			Bacteria	Firmicutes	Bacilli	Bacillales	Bacillaceae	Halobacillus	Halobacillus sp. GSP34; AY505519
			Bacteria	Firmicutes	Bacilli	Bacillales	Bacillaceae	Halobacillus	Halobacillus sp. IS-Hb5; AB189299
			Bacteria	Firmicutes	Bacilli	Bacillales	Bacillaceae	Halobacillus	Halobacillus sp. GSP15; AY505518
			Bacteria	Firmicutes	Bacilli	Bacillales	Bacillaceae	Halobacillus	Halobacillus karajensis (T); type strain: DSM 14948; AJ486874
			Bacteria	Firmicutes	Bacilli	Bacillales	Bacillaceae	Halobacillus	Halobacillus sp. IS-Hb1; AB189295
			Bacteria	Firmicutes	Bacilli	Bacillales	Bacillaceae	Halobacillus	Halobacillus trueperi; GSP38; AY505522
			Bacteria	Firmicutes	Bacilli	Bacillales	Bacillaceae	Halobacillus	Halobacillus sp. GSP35; AY553078
			Bacteria	Firmicutes	Bacilli	Bacillales	Bacillaceae	Halobacillus	Halobacillus sp. NT N165; AB167051
			Bacteria	Firmicutes	Bacilli	Bacillales	Bacillaceae	Halobacillus	Halobacillus sp. NT N104; AB167023
			Bacteria	Firmicutes	Bacilli	Bacillales	Bacillaceae	Halobacillus	Halobacillus sp. NT N143; AB167045
			Bacteria	Firmicutes	Bacilli	Bacillales	Bacillaceae	Halobacillus	Halobacillus sp. NT N168; AB167053
			Bacteria	Firmicutes	Bacilli	Bacillales	Bacillaceae	Halobacillus	Halobacillus sp. SB J82; AB167058
			Bacteria	Firmicutes	Bacilli	Bacillales	Bacillaceae	Halobacillus	Halobacillus dabanensis (T); D-8; AY351395
			Bacteria	Firmicutes	Bacilli	Bacillales	Bacillaceae	Halobacillus	Halobacillus sp. K3-1; AJ291752
			Bacteria	Firmicutes	Bacilli	Bacillales	Bacillaceae	Halobacillus	Halobacillus sp. SA-Hb4; AB189304
			Bacteria	Firmicutes	Bacilli	Bacillales	Bacillaceae	Halobacillus	Halobacillus sp. JM-Hb; AB189294
o1437	o1437 16S-HeGroup1	CAAGAGGAATACTCATTGCG	Bacteria	Proteobacteria	Epsilonproteobacteria	Campylobacterales	Helicobacteraceae	Helicobacter	Helicobacter pylori; 108; AY062898
			Bacteria	Proteobacteria	Epsilonproteobacteria	Campylobacterales	Helicobacteraceae	Helicobacter	Candidatus Helicobacter heilmannii; C2S; AF506775
			Bacteria	Proteobacteria	Epsilonproteobacteria	Campylobacterales	Helicobacteraceae	Helicobacter	Candidatus Helicobacter heilmannii; C4E; AF506778
			Bacteria	Proteobacteria	Epsilonproteobacteria	Campylobacterales	Helicobacteraceae	Helicobacter	Candidatus Helicobacter heilmannii; RM1; AF506793
			Bacteria	Proteobacteria	Epsilonproteobacteria	Campylobacterales	Helicobacteraceae	Helicobacter	Candidatus Helicobacter heilmannii; HU1; AF506784
			Bacteria	Proteobacteria	Epsilonproteobacteria	Campylobacterales	Helicobacteraceae	Helicobacter	Candidatus Helicobacter heilmannii; MM2; AF506783
			Bacteria	Proteobacteria	Epsilonproteobacteria	Campylobacterales	Helicobacteraceae	Helicobacter	Candidatus Helicobacter heilmannii; TD1; AF506794
			Bacteria	Proteobacteria	Epsilonproteobacteria	Campylobacterales	Helicobacteraceae	Helicobacter	Candidatus Helicobacter heilmannii; P5; AF506791
			Bacteria	Proteobacteria	Epsilonproteobacteria	Campylobacterales	Helicobacteraceae	Helicobacter	Helicobacter pylori; ATCC49396; AF363064
			Bacteria	Proteobacteria	Epsilonproteobacteria	Campylobacterales	Helicobacteraceae	Helicobacter	Helicobacter winghamensis; NLEP 98-0305; AF363063
			Bacteria	Proteobacteria	Epsilonproteobacteria	Campylobacterales	Helicobacteraceae	Helicobacter	Helicobacter winghamensis; NLEP 97-1611; AF363062
			Bacteria	Proteobacteria	Epsilonproteobacteria	Campylobacterales	Helicobacteraceae	Helicobacter	Candidatus Helicobacter heilmannii; P4; AF506790
			Bacteria	Proteobacteria	Epsilonproteobacteria	Campylobacterales	Helicobacteraceae	Helicobacter	Candidatus Helicobacter heilmannii; HU5; AF506769
			Bacteria	Proteobacteria	Epsilonproteobacteria	Campylobacterales	Helicobacteraceae	Helicobacter	Candidatus Helicobacter heilmannii; BC1; AF506772
			Bacteria	Proteobacteria	Epsilonproteobacteria	Campylobacterales	Helicobacteraceae	Helicobacter	Candidatus Helicobacter heilmannii; P3; AF506789
			Bacteria	Proteobacteria	Epsilonproteobacteria	Campylobacterales	Helicobacteraceae	Helicobacter	Candidatus Helicobacter heilmannii; MM1; AF506782
			Bacteria	Proteobacteria	Epsilonproteobacteria	Campylobacterales	Helicobacteraceae	Helicobacter	Candidatus Helicobacter heilmannii; WD1; AF506785
			Bacteria	Proteobacteria	Epsilonproteobacteria	Campylobacterales	Helicobacteraceae	Helicobacter	Helicobacter heilmannii type 1; 2; L10080

			Bacteria	Proteobacteria	Epsilonproteobacteria	Campylobacterales	Helicobacteraceae	Helicobacter	Helicobacter heilmannii type 1; L10079
			Bacteria	Proteobacteria	Epsilonproteobacteria	Campylobacterales	Helicobacteraceae	Helicobacter	Candidatus Helicobacter heilmannii; P1; AF506787
o1438	o1438 16S-KinDen	ACCTTGCGCTATTTGAGCAG	Bacteria	Proteobacteria	Betaproteobacteria	Neisseriales	Neisseriaceae	Kingella	uncultured Kingella sp.; 602B08(oral); AM420233
			Bacteria	Proteobacteria	Betaproteobacteria	Neisseriales	Neisseriaceae	Kingella	uncultured bacterium; P4D1-716; EF509629
			Bacteria	Proteobacteria	Betaproteobacteria	Neisseriales	Neisseriaceae	unclassified_Neisseriaceae	uncultured bacterium; MB-A2-115; AY093465
			Bacteria	Proteobacteria	Betaproteobacteria	Neisseriales	Neisseriaceae	Kingella	Kingella denitrificans (T); ATCC 33394; M22516
			Bacteria	Proteobacteria	Betaproteobacteria	Neisseriales	Neisseriaceae	Kingella	uncultured Kingella sp.; 10B353; FJ976305
o1439	o1439 16S-KlGroup1	TCCGAAACTGGCAGGCTAG	Bacteria	Proteobacteria	Gammaproteobacteria	Enterobacteriales	Enterobacteriaceae	Citrobacter	Citrobacter braakii (T); CDC 080-58; AF025368
			Bacteria	Proteobacteria	Gammaproteobacteria	Enterobacteriales	Enterobacteriaceae	Citrobacter	Citrobacter murliniae (T); CDC 2970-59; AF025369
			Bacteria	Proteobacteria	Gammaproteobacteria	Enterobacteriales	Enterobacteriaceae	Citrobacter	Citrobacter gillenii; CDC 4693-86; AF025367
			Bacteria	Proteobacteria	Gammaproteobacteria	Enterobacteriales	Enterobacteriaceae	Citrobacter	Citrobacter freundii; CDC 621-64; AF025365
			Bacteria	Proteobacteria	Gammaproteobacteria	Enterobacteriales	Enterobacteriaceae	Citrobacter	uncultured bacterium; 1013-1-CG30; AY532549
			Bacteria	Proteobacteria	Gammaproteobacteria	Enterobacteriales	Enterobacteriaceae	Citrobacter	Citrobacter freundii (T); DSM 30039; AJ233408
			Bacteria	Proteobacteria	Gammaproteobacteria	Enterobacteriales	Enterobacteriaceae	Citrobacter	Citrobacter freundii; SSCT56; AB210978
			Bacteria	Proteobacteria	Gammaproteobacteria	Enterobacteriales	Enterobacteriaceae	Citrobacter	Candidatus Cuticobacterium kirbyi; SA17; AY567708
			Bacteria	Proteobacteria	Gammaproteobacteria	Enterobacteriales	Enterobacteriaceae	Citrobacter	bacterium KA55; AY345447
			Bacteria	Proteobacteria	Gammaproteobacteria	Enterobacteriales	Enterobacteriaceae	Citrobacter	Citrobacter werkmanii (T); CDC 0876-58; AF025373
			Bacteria	Proteobacteria	Gammaproteobacteria	Enterobacteriales	Enterobacteriaceae	Citrobacter	rainbow trout intestinal bacterium 92; T92; AY374105
			Bacteria	Proteobacteria	Gammaproteobacteria	Enterobacteriales	Enterobacteriaceae	Citrobacter	Citrobacter freundii; A1; AY163805
			Bacteria	Proteobacteria	Gammaproteobacteria	Enterobacteriales	Enterobacteriaceae	Citrobacter	selenate-reducing bacterium TSA; AB018593
			Bacteria	Proteobacteria	Gammaproteobacteria	Enterobacteriales	Enterobacteriaceae	Citrobacter	selenate-reducing bacterium JSA; AB018592
			Bacteria	Proteobacteria	Gammaproteobacteria	Enterobacteriales	Enterobacteriaceae	Citrobacter	Citrobacter sp. TSA-1; AF463533
			Bacteria	Proteobacteria	Gammaproteobacteria	Enterobacteriales	Enterobacteriaceae	Citrobacter	Citrobacter freundii; WA1; AY259630
			Bacteria	Proteobacteria	Gammaproteobacteria	Enterobacteriales	Enterobacteriaceae	Raoultella	Raoultella terrigena (T); ATCC 33257T; AF129442
			Bacteria	Proteobacteria	Gammaproteobacteria	Enterobacteriales	Enterobacteriaceae	Citrobacter	Citrobacter freundii; HQ010516B-1; DQ010114
			Bacteria	Proteobacteria	Gammaproteobacteria	Enterobacteriales	Enterobacteriaceae	Citrobacter	uncultured gamma proteobacterium; APe4_69; AB074607
			Bacteria	Proteobacteria	Gammaproteobacteria	Enterobacteriales	Enterobacteriaceae	Citrobacter	uncultured gamma proteobacterium; APe2_81; AB074608
o1440	o1440 16S-Koarc	CGTGAACCCGGAATCCCTA	Archaea	Korarchaeota				Korarchaeota_genera_incertae_sedis	unidentified archaeon; pOWA19; AB007304
			Archaea	Korarchaeota				Korarchaeota_genera_incertae_sedis	unidentified archaeon; pUWA43; AB007306
			Archaea	Korarchaeota				Korarchaeota_genera_incertae_sedis	unidentified archaeon; pOWA54; AB007305
			Archaea	Korarchaeota				Korarchaeota_genera_incertae_sedis	uncultured archaeon; C1_R025; AF419639
			Archaea	Korarchaeota				Korarchaeota_genera_incertae_sedis	uncultured archaeon; F99a104; DQ228517
			Archaea	Korarchaeota				Korarchaeota_genera_incertae_sedis	uncultured korarchaeote pBA5; AF176347
			Archaea	Korarchaeota				Korarchaeota_genera_incertae_sedis	uncultured archaeon; F99a11; DQ228519
			Archaea	Korarchaeota				Korarchaeota_genera_incertae_sedis	uncultured archaeon; OUTa13; DQ228632
			Archaea	Korarchaeota				Korarchaeota_genera_incertae_sedis	uncultured korarchaeote; KorY38; DQ465908
			Archaea	Korarchaeota				Korarchaeota_genera_incertae_sedis	uncultured archaeon; F99a109; DQ228520
			Archaea	Korarchaeota				Korarchaeota_genera_incertae_sedis	uncultured korarchaeote; KorY03; DQ465910
			Archaea	Crenarchaeota	Thermoprotei	unclassified_Thermoprotei		Korarchaeota_genera_incertae_sedis	uncultured archaeon; FnvA94; AB213086
			Archaea	Korarchaeota				Korarchaeota_genera_incertae_sedis	uncultured archaeon; OUTa24; DQ228631
			Archaea	Korarchaeota					uncultured archaeon; CH1a42; DQ228611
			Archaea	Crenarchaeota	Thermoprotei	unclassified_Thermoprotei			uncultured archaeon; FnvA63; AB213074
			Archaea	Korarchaeota				Korarchaeota_genera_incertae_sedis	uncultured archaeon; OUTa12; DQ228629
			Archaea	Korarchaeota				Korarchaeota_genera_incertae_sedis	korarchaeote SRI-306; AF255604
			Archaea	Crenarchaeota	Thermoprotei	unclassified_Thermoprotei			uncultured crenarchaeote; pSL22; U63340
			Archaea	Korarchaeota				Korarchaeota_genera_incertae_sedis	uncultured korarchaeote pJP27; L25852
			Archaea	Korarchaeota				Korarchaeota_genera_incertae_sedis	uncultured korarchaeote pJP78; L25303
o1441	o1441 16S-KocKri	AATACCGGATGCGACTACTG	Bacteria	Actinobacteria	Actinobacteria	Actinomycetales	Micrococcaceae	Kocuria	Kocuria rosea; CU24; EF522129
			Bacteria	Actinobacteria	Actinobacteria	Actinomycetales	Micrococcaceae	Kocuria	uncultured bacterium; 457; DQ158131
			Bacteria	Actinobacteria	Actinobacteria	Actinomycetales	Micrococcaceae	Kocuria	uncultured bacterium; 405; DQ158123
			Bacteria	Actinobacteria	Actinobacteria	Actinomycetales	Micrococcaceae	Kocuria	uncultured bacterium; 462; DQ158132
			Bacteria	Actinobacteria	Actinobacteria	Actinomycetales	Micrococcaceae	Kocuria	uncultured bacterium; 431; DQ158129
			Bacteria	Actinobacteria	Actinobacteria	Actinomycetales	Micrococcaceae	Kocuria	uncultured bacterium; 411; DQ158124
			Bacteria	Actinobacteria	Actinobacteria	Actinomycetales	Micrococcaceae	Kocuria	uncultured bacterium; 430; DQ158128
			Bacteria	Actinobacteria	Actinobacteria	Actinomycetales	Micrococcaceae	Kocuria	uncultured bacterium; 425; DQ158126
			Bacteria	Actinobacteria	Actinobacteria	Actinomycetales	Micrococcaceae	Kocuria	Kocuria kristinae (T); DSM 20032; X80749
			Bacteria	Actinobacteria	Actinobacteria	Actinomycetales	Micrococcaceae	Kocuria	uncultured bacterium; 401; DQ158122
			Bacteria	Actinobacteria	Actinobacteria	Actinomycetales	Micrococcaceae	Kocuria	glacial ice bacterium Trans4678; AF479328
			Bacteria	Actinobacteria	Actinobacteria	Actinomycetales	Micrococcaceae	Kocuria	Kocuria kristinae; 6J-5b; EU379300
			Bacteria	Actinobacteria	Actinobacteria	Actinomycetales	Micrococcaceae	Kocuria	Kocuria kristinae; Ag17; EU554443
			Bacteria	Actinobacteria	Actinobacteria	Actinomycetales	Micrococcaceae	Kocuria	Kocuria sp. NP6; EU196302
o1442	o1442 16S-LaAciM	CCAAGAGACTGGGATAACAC	Bacteria	Firmicutes	Bacilli	Lactobacillales	Lactobacillaceae	Lactobacillus	Lactobacillus gasseri; ATCC 33323; AF519171
			Bacteria	Firmicutes	Bacilli	Lactobacillales	Lactobacillaceae	Lactobacillus	Lactobacillus acidophilus; BMF 6Lb6; M99704
			Bacteria	Firmicutes	Bacilli	Lactobacillales	Lactobacillaceae	Lactobacillus	Lactobacillus sp. IDSAc; AY094066
			Bacteria	Firmicutes	Bacilli	Lactobacillales	Lactobacillaceae	Lactobacillus	Lactobacillus sp. ID9203; AY862434
			Bacteria	Firmicutes	Bacilli	Lactobacillales	Lactobacillaceae	Lactobacillus	Lactobacillus gasseri; KC36a; AF243151
			Bacteria	Firmicutes	Bacilli	Lactobacillales	Lactobacillaceae	Lactobacillus	Lactobacillus gasseri; KC5a; AF243165
			Bacteria	Firmicutes	Bacilli	Lactobacillales	Lactobacillaceae	Lactobacillus	Lactobacillus gasseri; BLB1b; AF243144
			Bacteria	Firmicutes	Bacilli	Lactobacillales	Lactobacillaceae	Lactobacillus	Lactobacillus gasseri; TL33a; AF243173
			Bacteria	Firmicutes	Bacilli	Lactobacillales	Lactobacillaceae	Lactobacillus	uncultured bacterium; rRNA118; AY958891
			Bacteria	Firmicutes	Bacilli	Lactobacillales	Lactobacillaceae	Lactobacillus	Lactobacillus gasseri; BLB3; AF243142
			Bacteria	Firmicutes	Bacilli	Lactobacillales	Lactobacillaceae	Lactobacillus	Lactobacillus gasseri; KC26; AF243156
			Bacteria	Firmicutes	Bacilli	Lactobacillales	Lactobacillaceae	Lactobacillus	Lactobacillus gasseri; KC29; AF243157
			Bacteria	Firmicutes	Bacilli	Lactobacillales	Lactobacillaceae	Lactobacillus	uncultured bacterium; p-2315-962-2; AF371478
			Bacteria	Firmicutes	Bacilli	Lactobacillales	Lactobacillaceae	Lactobacillus	uncultured bacterium; p-136-a2; AF371476
			Bacteria	Firmicutes	Bacilli	Lactobacillales	Lactobacillaceae	Lactobacillus	Lactobacillus johnsonii (T); ATCC 33200 (T); AJ002515
			Bacteria	Firmicutes	Bacilli	Lactobacillales	Lactobacillaceae	Lactobacillus	Lactobacillus gasseri (T); M58820
			Bacteria	Firmicutes	Bacilli	Lactobacillales	Lactobacillaceae	Lactobacillus	uncultured bacterium; p-2865-6C5; AF371479
			Bacteria	Firmicutes	Bacilli	Lactobacillales	Lactobacillaceae	Lactobacillus	uncultured bacterium; rc1-3(8); AY239419
			Bacteria	Firmicutes	Bacilli	Lactobacillales	Lactobacillaceae	Lactobacillus	uncultured bacterium; p-13-a5; AF371477
			Bacteria	Firmicutes	Bacilli	Lactobacillales	Lactobacillaceae	Lactobacillus	Lactobacillus gasseri; 130704-6172; AY730721
o1443	o1443 16S-Labac	GAGTGGAACTCCATGTGTAG	Bacteria	Firmicutes	Bacilli	Lactobacillales	Lactobacillaceae	Lactobacillus	Lactobacillus versmoldensis (T); type strain: KU-3; AJ496791
			Bacteria	Firmicutes	Bacilli	Lactobacillales	Lactobacillaceae	Lactobacillus	Lactobacillus perolens (T); L532; Y19167
			Bacteria	Firmicutes	Bacilli	Lactobacillales	Lactobacillaceae	Lactobacillus	Lactobacillus gasseri; ATCC 33323; AF519171
			Bacteria	Firmicutes	Bacilli	Lactobacillales	Lactobacillaceae	Lactobacillus	Lactobacillus letivazi; JCL3994; AJ417738
			Bacteria	Firmicutes	Bacilli	Lactobacillales	Lactobacillaceae	Lactobacillus	Lactobacillus perolens; L534; Y19168
			Bacteria	Firmicutes	Bacilli	Lactobacillales	Lactobacillaceae	Lactobacillus	Lactobacillus sp. LA-6; AB005739
			Bacteria	Firmicutes	Bacilli	Lactobacillales	Lactobacillaceae	Lactobacillus	Lactobacillus amylolyticus (T); DSM 1664 T; Y17361

			Bacteria	Firmicutes	Bacilli	Lactobacillales	Lactobacillaceae	Lactobacillus	Lactobacillus sp. 121B; AF305930
			Bacteria	Firmicutes	Bacilli	Lactobacillales	Lactobacillaceae	Lactobacillus	Lactobacillus crispatus (T); DSM 20584 T; Y17362
			Bacteria	Firmicutes	Bacilli	Lactobacillales	Lactobacillaceae	Lactobacillus	Lactobacillus sp.; CCUG 28746; Y16329
			Bacteria	Firmicutes	Bacilli	Lactobacillales	Lactobacillaceae	Lactobacillus	Lactobacillus crispatus; NCTC 4; AJ242969
			Bacteria	Firmicutes	Bacilli	Lactobacillales	Lactobacillaceae	Lactobacillus	Lactobacillus salivarius; AF335475
			Bacteria	Firmicutes	Bacilli	Lactobacillales	Lactobacillaceae	Lactobacillus	Lactobacillus intestinalis (T); DSM 6629T; AJ306299
			Bacteria	Firmicutes	Bacilli	Lactobacillales	Lactobacillaceae	Lactobacillus	Lactobacillus hamsteri (T); DSM 5661T; AJ306298
			Bacteria	Firmicutes	Bacilli	Lactobacillales	Lactobacillaceae	Lactobacillus	Lactobacillus johnsonii (T); ATCC 33200 (T); AJ002515
			Bacteria	Firmicutes	Bacilli	Lactobacillales	Lactobacillaceae	Lactobacillus	Lactobacillus sp.; AB016864
			Bacteria	Firmicutes	Bacilli	Lactobacillales	Lactobacillaceae	Lactobacillus	Lactobacillus fornicalis (T); TV1018; Y18654
			Bacteria	Firmicutes	Bacilli	Lactobacillales	Lactobacillaceae	Lactobacillus	Lactobacillus animalis; NCDO 2425 (T); X61133
			Bacteria	Firmicutes	Bacilli	Lactobacillales	Lactobacillaceae	Lactobacillus	Lactobacillus acidophilus; NCDO 1748 (T); X61138
			Bacteria	Firmicutes	Bacilli	Lactobacillales	Lactobacillaceae	Lactobacillus	Lactobacillus gasseri; NCDO 2233 (T); X61137
o1444	o1444 16S-LacBuc	CTGTCACTTTAGGATGGACC	Bacteria	Firmicutes	Bacilli	Lactobacillales	Lactobacillaceae	Lactobacillus	Lactobacillus sunkii; YIT 11161 (= NRIC 0744, = JCM 15039, = DSM 19904); AB366385
			Bacteria	Firmicutes	Bacilli	Lactobacillales	Lactobacillaceae	Lactobacillus	Lactobacillus sunkii; YIT 11442; AB366390
			Bacteria	Firmicutes	Bacilli	Lactobacillales	Lactobacillaceae	Lactobacillus	Lactobacillus rapi; YIT 11204 (= NRIC 0743, = JCM 15042, = DSM 19907); AB366389
			Bacteria	Firmicutes	Bacilli	Lactobacillales	Lactobacillaceae	Lactobacillus	Lactobacillus sunkii; YIT 11539; AB366394
			Bacteria	Firmicutes	Bacilli	Lactobacillales	Lactobacillaceae	Lactobacillus	Lactobacillus sunkii; YIT 11549 (= JCM 15046, = DSM 19905); AB366395
			Bacteria	Firmicutes	Bacilli	Lactobacillales	Lactobacillaceae	Lactobacillus	Lactobacillus sunkii; YIT 11557; AB366396
			Bacteria	Firmicutes	Bacilli	Lactobacillales	Lactobacillaceae	Lactobacillus	Lactobacillus buchneri; JCM1115; AB205055
			Bacteria	Firmicutes	Bacilli	Lactobacillales	Lactobacillaceae	Lactobacillus	Lactobacillus sp. NGRI 0130; AB362984
			Bacteria	Firmicutes	Bacilli	Lactobacillales	Lactobacillaceae	Lactobacillus	Lactobacillus sp. NGRI 0001; AB362983
			Bacteria	Firmicutes	Bacilli	Lactobacillales	Lactobacillaceae	Lactobacillus	Lactobacillus buchneri; F-32; AB300611
			Bacteria	Firmicutes	Bacilli	Lactobacillales	Lactobacillaceae	Lactobacillus	uncultured bacterium; FB04G08; FM872932
			Bacteria	Firmicutes	Bacilli	Lactobacillales	Lactobacillaceae	Lactobacillus	Lactobacillus parakefiri; JCM 8573; AB370879
			Bacteria	Firmicutes	Bacilli	Lactobacillales	Lactobacillaceae	Lactobacillus	Lactobacillus parabuchneri; 2173; EF535231
			Bacteria	Firmicutes	Bacilli	Lactobacillales	Lactobacillaceae	Lactobacillus	Lactobacillus buchneri; NRRL B-30929; DQ987924
			Bacteria	Firmicutes	Bacilli	Lactobacillales	Lactobacillaceae	Lactobacillus	Lactobacillus sp. Hon2; EF187244
			Bacteria	Firmicutes	Bacilli	Lactobacillales	Lactobacillaceae	Lactobacillus	Lactobacillus parakefiri (T); LMG 15133T; AY026750
			Bacteria	Firmicutes	Bacilli	Lactobacillales	Lactobacillaceae	unclassified_Lactobacillaceae	uncultured Firmicutes bacterium; HBG_A5R3-1; DQ837633
			Bacteria	Firmicutes	Bacilli	Lactobacillales	Lactobacillaceae	unclassified_Lactobacillaceae	uncultured Firmicutes bacterium; HBG_A5R3-3; DQ837632
			Bacteria	Firmicutes	Bacilli	Lactobacillales	Lactobacillaceae	Lactobacillus	Lactobacillus buchneri (T); M58811
			Bacteria	Firmicutes	Bacilli	Lactobacillales	Lactobacillaceae	Lactobacillus	Lactobacillus buchneri; NCDO 110 (T); X61139
o1445	o1445 16S-LacCri	ATACAAAGTTCCCTTCGGGG	Bacteria	Firmicutes	Bacilli	Lactobacillales	Lactobacillaceae	Lactobacillus	Lactobacillus crispatus (T); DSM 20584 T; Y17362
			Bacteria	Firmicutes	Bacilli	Lactobacillales	Lactobacillaceae	Lactobacillus	Lactobacillus kefiranofaciens subsp. kefirgranum; CECT 5919T; AJ575742
			Bacteria	Firmicutes	Bacilli	Lactobacillales	Lactobacillaceae	Lactobacillus	Lactobacillus crispatus; NCTC 4; AJ242969
			Bacteria	Firmicutes	Bacilli	Lactobacillales	Lactobacillaceae	Lactobacillus	Lactobacillus crispatus; ATCC33820; AF257097
			Bacteria	Firmicutes	Bacilli	Lactobacillales	Lactobacillaceae	Lactobacillus	Lactobacillus crispatus; ATCC33197; AF257096
			Bacteria	Firmicutes	Bacilli	Lactobacillales	Lactobacillaceae	Lactobacillus	Lactobacillus crispatus; KC12a; AF243152
			Bacteria	Firmicutes	Bacilli	Lactobacillales	Lactobacillaceae	Lactobacillus	Lactobacillus crispatus; TL39b; AF243175
			Bacteria	Firmicutes	Bacilli	Lactobacillales	Lactobacillaceae	Lactobacillus	Lactobacillus crispatus; KC12b; AF243150
			Bacteria	Firmicutes	Bacilli	Lactobacillales	Lactobacillaceae	Lactobacillus	Lactobacillus kefiranofaciens subsp. kefiranofaciens; type strain: LMG 19149; AJ575259
			Bacteria	Firmicutes	Bacilli	Lactobacillales	Lactobacillaceae	Lactobacillus	Lactobacillus kefiranofaciens subsp. kefiranofaciens; R-14703; AJ575260
			Bacteria	Firmicutes	Bacilli	Lactobacillales	Lactobacillaceae	Lactobacillus	Lactobacillus crispatus; KLB79; AF243169
			Bacteria	Firmicutes	Bacilli	Lactobacillales	Lactobacillaceae	Lactobacillus	Lactobacillus crispatus; TL23a; AF243171
			Bacteria	Firmicutes	Bacilli	Lactobacillales	Lactobacillaceae	Lactobacillus	Lactobacillus crispatus; KC35b; AF243158
			Bacteria	Firmicutes	Bacilli	Lactobacillales	Lactobacillaceae	Lactobacillus	Lactobacillus kefiranofaciens subsp. kefiranofaciens; R-12929; AJ575262
			Bacteria	Firmicutes	Bacilli	Lactobacillales	Lactobacillaceae	Lactobacillus	Lactobacillus crispatus; TL25a; AF243172
			Bacteria	Firmicutes	Bacilli	Lactobacillales	Lactobacillaceae	Lactobacillus	Lactobacillus crispatus; TL35; AF243174
			Bacteria	Firmicutes	Bacilli	Lactobacillales	Lactobacillaceae	Lactobacillus	Lactobacillus kefiranofaciens subsp. kefiranofaciens; type strain: LMG 15132; AJ575261
			Bacteria	Firmicutes	Bacilli	Lactobacillales	Lactobacillaceae	Lactobacillus	Lactobacillus crispatus; BLB2; AF243141
			Bacteria	Firmicutes	Bacilli	Lactobacillales	Lactobacillaceae	Lactobacillus	Lactobacillus crispatus; TL13; AF243170
			Bacteria	Firmicutes	Bacilli	Lactobacillales	Lactobacillaceae	Lactobacillus	Lactobacillus crispatus; KLB46; AF243167
o1446	o1446 16S-LacDel	GCAATCTGCCCTAAAGACTG	Bacteria	Firmicutes	Bacilli	Lactobacillales	Lactobacillaceae	Lactobacillus	Lactobacillus delbrueckii subsp. bulgaricus; LGM2; AY675257
			Bacteria	Firmicutes	Bacilli	Lactobacillales	Lactobacillaceae	Lactobacillus	Lactobacillus delbrueckii subsp. bulgaricus; DL2; AB200872
			Bacteria	Firmicutes	Bacilli	Lactobacillales	Lactobacillaceae	Lactobacillus	Lactobacillus delbrueckii subsp. delbrueckii; BCRC12195; AY773949
			Bacteria	Firmicutes	Bacilli	Lactobacillales	Lactobacillaceae	Lactobacillus	Lactobacillus delbrueckii subsp. bulgaricus; LG1; AY735407
			Bacteria	Firmicutes	Bacilli	Lactobacillales	Lactobacillaceae	Lactobacillus	Lactobacillus delbrueckii subsp. indicus (T); NCC725; AY421720
			Bacteria	Firmicutes	Bacilli	Lactobacillales	Lactobacillaceae	Lactobacillus	Lactobacillus delbrueckii subsp. delbrueckii; ATCC 9649; AY050172
			Bacteria	Firmicutes	Bacilli	Lactobacillales	Lactobacillaceae	Lactobacillus	Lactobacillus delbrueckii subsp. lactis (T); M58823
			Bacteria	Firmicutes	Bacilli	Lactobacillales	Lactobacillaceae	Lactobacillus	Lactobacillus delbrueckii; JCM 1002; AB007908
			Bacteria	Firmicutes	Bacilli	Lactobacillales	Lactobacillaceae	Lactobacillus	Lactobacillus delbrueckii subsp. bulgaricus (T); ATCC 11842; AY050171
			Bacteria	Firmicutes	Bacilli	Lactobacillales	Lactobacillaceae	Lactobacillus	Lactobacillus delbrueckii subsp. lactis; BCRC11051; AY773950
			Bacteria	Firmicutes	Bacilli	Lactobacillales	Lactobacillaceae	Lactobacillus	Lactobacillus delbrueckii subsp. lactis; ATCC 12315; AY050173
			Bacteria	Firmicutes	Bacilli	Lactobacillales	Lactobacillaceae	Lactobacillus	Lactobacillus delbrueckii subsp. bulgaricus; BCRC10696; AY773948
			Bacteria	Firmicutes	Bacilli	Lactobacillales	Lactobacillaceae	Lactobacillus	uncultured bacterium; p-2370-55G5; AF371474
			Bacteria	Firmicutes	Bacilli	Lactobacillales	Lactobacillaceae	Lactobacillus	uncultured bacterium; 51CR; AF349922
			Bacteria	Firmicutes	Bacilli	Lactobacillales	Lactobacillaceae	Lactobacillus	uncultured bacterium; 54CR; AF349925
			Bacteria	Firmicutes	Bacilli	Lactobacillales	Lactobacillaceae	Lactobacillus	uncultured bacterium; 37CR; AF349919
			Bacteria	Firmicutes	Bacilli	Lactobacillales	Lactobacillaceae	Lactobacillus	Lactobacillus delbrueckii (T); M58814
			Bacteria	Firmicutes	Bacilli	Lactobacillales	Lactobacillaceae	Lactobacillus	uncultured bacterium; p-4729-2Wb2; AF371475
			Bacteria	Firmicutes	Bacilli	Lactobacillales	Lactobacillaceae	Lactobacillus	Lactobacillus delbrueckii; NCDO 213; X52654
			Bacteria	Firmicutes	Bacilli	Lactobacillales	Lactobacillaceae	Lactobacillus	Lactobacillus sp. CY1; AY165006
o1447	o1447 16S-LacFer	AGAGTGCAGGCGGTTTTCTA	Bacteria	Firmicutes	Bacilli	Lactobacillales	Lactobacillaceae	Lactobacillus	Lactobacillus sp. rennanqilyf4; AY332396
			Bacteria	Firmicutes	Bacilli	Lactobacillales	Lactobacillaceae	Lactobacillus	Lactobacillus fermentum; YB5; DQ208931
			Bacteria	Firmicutes	Bacilli	Lactobacillales	Lactobacillaceae	Lactobacillus	Lactobacillus fermentum; KLB12; AF522394
			Bacteria	Firmicutes	Bacilli	Lactobacillales	Lactobacillaceae	Lactobacillus	Lactobacillus fermentum; MD-9; AY373589
			Bacteria	Firmicutes	Bacilli	Lactobacillales	Lactobacillaceae	Lactobacillus	Lactobacillus fermentum (T); AJ575812
			Bacteria	Firmicutes	Bacilli	Lactobacillales	Lactobacillaceae	Lactobacillus	Lactobacillus fermentum; KLD; AF302116
			Bacteria	Firmicutes	Bacilli	Lactobacillales	Lactobacillaceae	Lactobacillus	Lactobacillus fermentum; SFCB2-3; DQ399352
			Bacteria	Firmicutes	Bacilli	Lactobacillales	Lactobacillaceae	Lactobacillus	Lactobacillus fermentum; SFCB2-6c; DQ486144
			Bacteria	Firmicutes	Bacilli	Lactobacillales	Lactobacillaceae	Lactobacillus	Lactobacillus fermentum; SFCB2-1; DQ399350
			Bacteria	Firmicutes	Bacilli	Lactobacillales	Lactobacillaceae	Lactobacillus	Lactobacillus fermentum; F53; AF243149
			Bacteria	Firmicutes	Bacilli	Lactobacillales	Lactobacillaceae	Lactobacillus	Lactobacillus fermentum; KC5b; AF243166
			Bacteria	Firmicutes	Bacilli	Lactobacillales	Lactobacillaceae	Lactobacillus	Lactobacillus sp. CR-6AS; crev-7; AF349935
			Bacteria	Firmicutes	Bacilli	Lactobacillales	Lactobacillaceae	Lactobacillus	Lactobacillus fermentum; MCH4-2; AB257865
			Bacteria	Firmicutes	Bacilli	Lactobacillales	Lactobacillaceae	Lactobacillus	uncultured bacterium; 60CR; AF349926
			Bacteria	Firmicutes	Bacilli	Lactobacillales	Lactobacillaceae	Lactobacillus	Lactobacillus fermentum; KRM; U62624

			Bacteria	Firmicutes	Bacilli	Lactobacillales	Lactobacillaceae	Lactobacillus	Lactobacillus fermentum; BFE 6618; AY929279
			Bacteria	Firmicutes	Bacilli	Lactobacillales	Lactobacillaceae	Lactobacillus	Lactobacillus fermentum; M58819
			Bacteria	Firmicutes	Bacilli	Lactobacillales	Lactobacillaceae	Lactobacillus	Lactobacillus fermentum; BFE 6658; AY929282
			Bacteria	Firmicutes	Bacilli	Lactobacillales	Lactobacillaceae	Lactobacillus	Lactobacillus fermentum; BFE 6628; AY929281
			Bacteria	Firmicutes	Bacilli	Lactobacillales	Lactobacillaceae	Lactobacillus	Lactobacillus fermentum; BFE 6620; AY929280
o1448	o1448 16S-LacGal	AAACAGGCGCTAATACCGGA	Bacteria	Firmicutes	Bacilli	Lactobacillales	Lactobacillaceae	Lactobacillus	uncultured bacterium; BF0002B056; AM697106
			Bacteria	Actinobacteria	Actinobacteria	Actinomycetales	unclassified_Propionibacterineae		uncultured bacterium; AB-Mi72; DQ347885
			Bacteria	Firmicutes	Bacilli	Lactobacillales	Lactobacillaceae	Lactobacillus	uncultured bacterium; nbw828e08c1; GQ009208
			Bacteria	Firmicutes	Bacilli	Lactobacillales	Lactobacillaceae	Lactobacillus	uncultured bacterium; nbw831d09c1; GQ009675
			Bacteria	Firmicutes	Bacilli	Lactobacillales	Lactobacillaceae	Lactobacillus	uncultured bacterium; nbw831d04c1; GQ009670
			Bacteria	Firmicutes	Bacilli	Lactobacillales	Lactobacillaceae	Lactobacillus	uncultured bacterium; nbw830a07c1; GQ009589
			Bacteria	Firmicutes	Bacilli	Lactobacillales	Lactobacillaceae	Lactobacillus	uncultured bacterium; nbw831d11c1; GQ009676
			Bacteria	Firmicutes	Bacilli	Lactobacillales	Lactobacillaceae	Lactobacillus	uncultured bacterium; nbw826f05c1; GQ009055
			Bacteria	Firmicutes	Bacilli	Lactobacillales	Lactobacillaceae	Lactobacillus	uncultured bacterium; nbw829e10c1; GQ009287
			Bacteria	Firmicutes	Bacilli	Lactobacillales	Lactobacillaceae	Lactobacillus	uncultured bacterium; nbw830g02c1; GQ009632
			Bacteria	Firmicutes	Bacilli	Lactobacillales	Lactobacillaceae	Lactobacillus	uncultured bacterium; nbw779f08c1; GQ009807
			Bacteria	Firmicutes	Bacilli	Lactobacillales	Lactobacillaceae	Lactobacillus	uncultured bacterium; nbw827e09c1; GQ009129
			Bacteria	Firmicutes	Bacilli	Lactobacillales	Lactobacillaceae	Lactobacillus	uncultured bacterium; nbw829e09c1; GQ009286
			Bacteria	Firmicutes	Bacilli	Lactobacillales	Lactobacillaceae	Lactobacillus	uncultured bacterium; nbw827f06c1; GQ009137
			Bacteria	Firmicutes	Bacilli	Lactobacillales	Lactobacillaceae	Lactobacillus	uncultured bacterium; nbw829b04c1; GQ009247
			Bacteria	Firmicutes	Bacilli	Lactobacillales	Lactobacillaceae	Lactobacillus	uncultured bacterium; nbw831d03c1; GQ009669
			Bacteria	Firmicutes	Bacilli	Lactobacillales	Lactobacillaceae	Lactobacillus	uncultured bacterium; nbw831e11c1; GQ009684
			Bacteria	Firmicutes	Bacilli	Lactobacillales	Lactobacillaceae	Lactobacillus	uncultured bacterium; nbw830a09c1; GQ009590
			Bacteria	Firmicutes	Bacilli	Lactobacillales	Lactobacillaceae	Lactobacillus	uncultured bacterium; nbw831d12c1; GQ009677
			Bacteria	Firmicutes	Bacilli	Lactobacillales	Lactobacillaceae	Lactobacillus	uncultured bacterium; nbw827f01c1; GQ009132
o1449	o1449 16S-LacGroup	CGTGCTTGCACTGAATTGAC	Bacteria	Firmicutes	Bacilli	Lactobacillales	Lactobacillaceae	Lactobacillus	Lactobacillus sp. oral clone CX036; AY005048
			Bacteria	Firmicutes	Bacilli	Lactobacillales	Lactobacillaceae	Lactobacillus	uncultured bacterium; rRNA138; AY958911
			Bacteria	Firmicutes	Bacilli	Lactobacillales	Lactobacillaceae	Lactobacillus	uncultured bacterium; rRNA404; AY959177
			Bacteria	Firmicutes	Bacilli	Lactobacillales	Lactobacillaceae	Lactobacillus	Lactobacillus vaginalis; ATCC49540; AF243177
			Bacteria	Firmicutes	Bacilli	Lactobacillales	Lactobacillaceae	Lactobacillus	uncultured bacterium; BF0002B020; AM697070
			Bacteria	Firmicutes	Bacilli	Lactobacillales	Lactobacillaceae	Lactobacillus	Lactobacillus vaginalis; KC19; AF243154
			Bacteria	Firmicutes	Bacilli	Lactobacillales	Lactobacillaceae	Lactobacillus	uncultured bacterium; R-1210(2); DQ777963
			Bacteria	Firmicutes	Bacilli	Lactobacillales	Lactobacillaceae	Lactobacillus	uncultured bacterium; V2-3_69_E09/20-1518; EF653407
			Bacteria	Firmicutes	Bacilli	Lactobacillales	Lactobacillaceae	Lactobacillus	uncultured bacterium; oi29; DQ057431
			Bacteria	Firmicutes	Bacilli	Lactobacillales	Lactobacillaceae	Lactobacillus	uncultured Lactobacillus sp.; ABXD_L3; FJ440068
			Bacteria	Firmicutes	Bacilli	Lactobacillales	Lactobacillaceae	Lactobacillus	Lactobacillus sp. 34-10B; AY960573
			Bacteria	Firmicutes	Bacilli	Lactobacillales	Lactobacillaceae	Lactobacillus	Lactobacillus vaginalis; MF2123; AB158767
			Bacteria	Firmicutes	Bacilli	Lactobacillales	Lactobacillaceae	Lactobacillus	uncultured bacterium; V3_3_35_C05/21-1506; EF653414
			Bacteria	Firmicutes	Bacilli	Lactobacillales	Lactobacillaceae	Lactobacillus	Lactobacillus vaginalis; BCS154; EU547312
			Bacteria	Firmicutes	Bacilli	Lactobacillales	Lactobacillaceae	Lactobacillus	Lactobacillus vaginalis; AC811103; AB158768
			Bacteria	Firmicutes	Bacilli	Lactobacillales	Lactobacillaceae	Lactobacillus	Lactobacillus vaginalis; BCS68; EU547302
			Bacteria	Firmicutes	Bacilli	Lactobacillales	Lactobacillaceae	Lactobacillus	uncultured bacterium; aab47f02; DQ815403
			Bacteria	Firmicutes	Bacilli	Lactobacillales	Lactobacillaceae	Lactobacillus	uncultured bacterium; aab48c11; DQ815432
			Bacteria	Firmicutes	Bacilli	Lactobacillales	Lactobacillaceae	Lactobacillus	Lactobacillus vaginalis; SR8; EF460497
			Bacteria	Firmicutes	Bacilli	Lactobacillales	Lactobacillaceae	Lactobacillus	uncultured bacterium; ECH_aai40b10; EU460898
o1450	o1450 16S-LacGroup1	GATCCAAGAACCGCATGGTT	Bacteria	Firmicutes	Bacilli	Lactobacillales	Lactobacillaceae	Lactobacillus	Lactobacillus paracasei subsp. paracasei; Akira1; AY369076
			Bacteria	Firmicutes	Bacilli	Lactobacillales	Lactobacillaceae	Lactobacillus	Lactobacillus sp. rennanqilyf8; AY332394
			Bacteria	Firmicutes	Bacilli	Lactobacillales	Lactobacillaceae	Lactobacillus	Lactobacillus sp. rennanqilyf9; AY332393
			Bacteria	Firmicutes	Bacilli	Lactobacillales	Lactobacillaceae	Lactobacillus	Lactobacillus sp. rennanqilyf7; AY332395
			Bacteria	Firmicutes	Bacilli	Lactobacillales	Lactobacillaceae	Lactobacillus	Lactobacillus casei; UK 318; fermented milk; AJ272201
			Bacteria	Firmicutes	Bacilli	Lactobacillales	Lactobacillaceae	Lactobacillus	Lactobacillus paracasei subsp. paracasei; A221(LPH); AB126872
			Bacteria	Firmicutes	Bacilli	Lactobacillales	Lactobacillaceae	Lactobacillus	Lactobacillus paracasei subsp. paracasei; LPC3; AY692452
			Bacteria	Firmicutes	Bacilli	Lactobacillales	Lactobacillaceae	Lactobacillus	Lactobacillus casei; ATCC334; D86517
			Bacteria	Firmicutes	Bacilli	Lactobacillales	Lactobacillaceae	Lactobacillus	Lactobacillus casei; BL23; AF385770
			Bacteria	Firmicutes	Bacilli	Lactobacillales	Lactobacillaceae	Lactobacillus	Lactobacillus paracasei subsp. paracasei; F31; AF243147
			Bacteria	Firmicutes	Bacilli	Lactobacillales	Lactobacillaceae	Lactobacillus	Lactobacillus paracasei subsp. paracasei; KLB58; AF243168
			Bacteria	Firmicutes	Bacilli	Lactobacillales	Lactobacillaceae	Lactobacillus	Lactobacillus casei; JCM 1133; D16548
			Bacteria	Firmicutes	Bacilli	Lactobacillales	Lactobacillaceae	Lactobacillus	Lactobacillus paracasei subsp. tolerans; NBRC 15906; AB181950
			Bacteria	Firmicutes	Bacilli	Lactobacillales	Lactobacillaceae	Lactobacillus	Lactobacillus paracasei; UK179; from a traditional fermented milk; AJ271854
			Bacteria	Firmicutes	Bacilli	Lactobacillales	Lactobacillaceae	Lactobacillus	Lactobacillus casei; JCM 1171; D16550
			Bacteria	Firmicutes	Bacilli	Lactobacillales	Lactobacillaceae	Lactobacillus	Lactobacillus paracasei; UK 3.338; AJ272010
			Bacteria	Firmicutes	Bacilli	Lactobacillales	Lactobacillaceae	Lactobacillus	Lactobacillus casei; JCM 1181; D16549
			Bacteria	Firmicutes	Bacilli	Lactobacillales	Lactobacillaceae	Lactobacillus	Lactobacillus casei; MCRF-284; AY299487
			Bacteria	Firmicutes	Bacilli	Lactobacillales	Lactobacillaceae	Lactobacillus	Lactobacillus sp. rennanqilyf13; AY332391
			Bacteria	Firmicutes	Bacilli	Lactobacillales	Lactobacillaceae	Lactobacillus	Lactobacillus sp. P8; AF306539
o1451	o1451 16S-LacGroup2	AAGCGCATCGGAAACTGGG	Bacteria	Firmicutes	Bacilli	Lactobacillales	Lactobacillaceae	Lactobacillus	Lactobacillus paracasei subsp. paracasei; Akira1; AY369076
			Bacteria	Firmicutes	Bacilli	Lactobacillales	Lactobacillaceae	Lactobacillus	Lactobacillus casei; UK 318; fermented milk; AJ272201
			Bacteria	Firmicutes	Bacilli	Lactobacillales	Lactobacillaceae	Lactobacillus	Lactobacillus paracasei subsp. paracasei; A221(LPH); AB126872
			Bacteria	Firmicutes	Bacilli	Lactobacillales	Lactobacillaceae	Lactobacillus	Lactobacillus paracasei subsp. paracasei; LPC3; AY692452
			Bacteria	Firmicutes	Bacilli	Lactobacillales	Lactobacillaceae	Lactobacillus	Lactobacillus casei; JCM 8129 (=NCDO 173); D86518
			Bacteria	Firmicutes	Bacilli	Lactobacillales	Lactobacillaceae	Lactobacillus	Lactobacillus casei; ATCC334; D86517
			Bacteria	Firmicutes	Bacilli	Lactobacillales	Lactobacillaceae	Lactobacillus	Lactobacillus casei; BL23; AF385770
			Bacteria	Firmicutes	Bacilli	Lactobacillales	Lactobacillaceae	Lactobacillus	Lactobacillus casei; CWBI-B623; AJ558112
			Bacteria	Firmicutes	Bacilli	Lactobacillales	Lactobacillaceae	Lactobacillus	Lactobacillus casei; ATCC 393; AF469172
			Bacteria	Firmicutes	Bacilli	Lactobacillales	Lactobacillaceae	Lactobacillus	Lactobacillus zeae (T); ATCC15820; D86516
			Bacteria	Firmicutes	Bacilli	Lactobacillales	Lactobacillaceae	Lactobacillus	Lactobacillus paracasei subsp. paracasei; F31; AF243147
			Bacteria	Firmicutes	Bacilli	Lactobacillales	Lactobacillaceae	Lactobacillus	Lactobacillus paracasei subsp. paracasei; KLB58; AF243168
			Bacteria	Firmicutes	Bacilli	Lactobacillales	Lactobacillaceae	Lactobacillus	Lactobacillus casei; JCM 1134; D16551
			Bacteria	Firmicutes	Bacilli	Lactobacillales	Lactobacillaceae	Lactobacillus	Lactobacillus casei; JCM 1133; D16548
			Bacteria	Firmicutes	Bacilli	Lactobacillales	Lactobacillaceae	Lactobacillus	Lactobacillus paracasei subsp. tolerans; NBRC 15906; AB181950
			Bacteria	Firmicutes	Bacilli	Lactobacillales	Lactobacillaceae	Lactobacillus	Lactobacillus paracasei; UK179; from a traditional fermented milk; AJ271854
			Bacteria	Firmicutes	Bacilli	Lactobacillales	Lactobacillaceae	Lactobacillus	Lactobacillus casei; JCM 1171; D16550
			Bacteria	Firmicutes	Bacilli	Lactobacillales	Lactobacillaceae	Lactobacillus	Lactobacillus paracasei; UK 3.338; AJ272010
			Bacteria	Firmicutes	Bacilli	Lactobacillales	Lactobacillaceae	Lactobacillus	Lactobacillus casei; JCM 1181; D16549
			Bacteria	Firmicutes	Bacilli	Lactobacillales	Lactobacillaceae	Lactobacillus	Lactobacillus sp. P8; AF306539
			Bacteria	Firmicutes	Bacilli	Lactobacillales	Lactobacillaceae	Lactobacillus	Lactobacillus salivarius; JCM 1047; AY137588
o1452	o1452 16S-LacGroup4	GTAAGAAGTTGAGTGGCGGA	Bacteria	Firmicutes	Bacilli	Lactobacillales	Lactobacillaceae	Lactobacillus	Lactobacillus salivarius; JCM 1042; AY137584
			Bacteria	Firmicutes	Bacilli	Lactobacillales	Lactobacillaceae	Lactobacillus	Lactobacillus salivarius; JCM 1044; AY137585

			Bacteria	Firmicutes	Bacilli	Lactobacillales	Lactobacillaceae	Lactobacillus	Lactobacillus salivarius; JCM 1045; AY137586
			Bacteria	Firmicutes	Bacilli	Lactobacillales	Lactobacillaceae	Lactobacillus	Lactobacillus salivarius; JCM 1230; AY137589
			Bacteria	Firmicutes	Bacilli	Lactobacillales	Lactobacillaceae	Lactobacillus	Lactobacillus salivarius; AF335475
			Bacteria	Firmicutes	Bacilli	Lactobacillales	Lactobacillaceae	Lactobacillus	Lactobacillus salivarius; AF420311
			Bacteria	Firmicutes	Bacilli	Lactobacillales	Lactobacillaceae	Lactobacillus	Lactobacillus salivarius; JCM 1046; AY137587
			Bacteria	Firmicutes	Bacilli	Lactobacillales	Lactobacillaceae	Lactobacillus	uncultured bacterium; oi43; DQ057432
			Bacteria	Firmicutes	Bacilli	Lactobacillales	Lactobacillaceae	Lactobacillus	chicken intestinal bacterium MRS 4.5; AF201900
			Bacteria	Firmicutes	Bacilli	Lactobacillales	Lactobacillaceae	Lactobacillus	uncultured bacterium; p-4323-4Wa3; AF371497
			Bacteria	Firmicutes	Bacilli	Lactobacillales	Lactobacillaceae	Lactobacillus	bacterium ii1306; DQ057461
			Bacteria	Firmicutes	Bacilli	Lactobacillales	Lactobacillaceae	Lactobacillus	bacterium ii1309; DQ057462
			Bacteria	Firmicutes	Bacilli	Lactobacillales	Lactobacillaceae	Lactobacillus	uncultured bacterium; oc46; DQ057418
			Bacteria	Firmicutes	Bacilli	Lactobacillales	Lactobacillaceae	Lactobacillus	Lactobacillus salivarius; M59054
			Bacteria	Firmicutes	Bacilli	Lactobacillales	Lactobacillaceae	Lactobacillus	bacterium ii1222; DQ057439
			Bacteria	Firmicutes	Bacilli	Lactobacillales	Lactobacillaceae	Lactobacillus	bacterium ii1283; DQ057454
			Bacteria	Firmicutes	Bacilli	Lactobacillales	Lactobacillaceae	Lactobacillus	bacterium ii1349; DQ057470
			Bacteria	Firmicutes	Bacilli	Lactobacillales	Lactobacillaceae	Lactobacillus	uncultured bacterium; ci234; DQ057405
			Bacteria	Firmicutes	Bacilli	Lactobacillales	Lactobacillaceae	Lactobacillus	uncultured bacterium; ci243; DQ057407
o1453	o1453 16S-LacGroup5	GAGCGGAACCAGCAGATCTG	Bacteria	Firmicutes	Bacilli	Lactobacillales	Lactobacillaceae	Lactobacillus	Lactobacillus sp. oral clone HT070; AY349383
			Bacteria	Firmicutes	Bacilli	Lactobacillales	Lactobacillaceae	Lactobacillus	Lactobacillus ultunensis; Kx146C1; LMG 22117T; DSM 16047T; CCUG 48460T; AY253
o1454	o1454 16S-LacGroup6	ACGCTGAGGCTCGAAAGTAT	Bacteria	Firmicutes	Bacilli	Lactobacillales	Lactobacillaceae	Lactobacillus	Lactobacillus plantarum; L137; AB112083
			Bacteria	Firmicutes	Bacilli	Lactobacillales	Lactobacillaceae	Lactobacillus	Lactobacillus plantarum; LP3; AY675256
			Bacteria	Firmicutes	Bacilli	Lactobacillales	Lactobacillaceae	Lactobacillus	Lactobacillus sp. CECT 5923; AJ576008
			Bacteria	Firmicutes	Bacilli	Lactobacillales	Lactobacillaceae	Lactobacillus	Lactobacillus rennini (T); CECT 5922; AJ576007
			Bacteria	Firmicutes	Bacilli	Lactobacillales	Lactobacillaceae	Lactobacillus	Lactobacillus plantarum; HSW36; AJ272031
			Bacteria	Firmicutes	Bacilli	Lactobacillales	Lactobacillaceae	Lactobacillus	Lactobacillus coryniformis subsp. coryniformis (T); CECT 4129; AJ575741
			Bacteria	Firmicutes	Bacilli	Lactobacillales	Lactobacillaceae	Lactobacillus	Lactobacillus plantarum; RO7; AF515222
			Bacteria	Firmicutes	Bacilli	Lactobacillales	Lactobacillaceae	Lactobacillus	Lactobacillus plantarum; Chikuso-1; AB104855
			Bacteria	Firmicutes	Bacilli	Lactobacillales	Lactobacillaceae	Lactobacillus	Lactobacillus pentosus (T); JCM 1558; D79211
			Bacteria	Firmicutes	Bacilli	Lactobacillales	Lactobacillaceae	Lactobacillus	Lactobacillus sp. MR-2; AF516755
			Bacteria	Firmicutes	Bacilli	Lactobacillales	Lactobacillaceae	Lactobacillus	Lactobacillus sp.; ATCC 10776; D79213
			Bacteria	Firmicutes	Bacilli	Lactobacillales	Lactobacillaceae	Lactobacillus	Lactobacillus sp. MR-1; AF516754
			Bacteria	Firmicutes	Bacilli	Lactobacillales	Lactobacillaceae	Lactobacillus	Lactobacillus plantarum (T); JCM 1149; D79210
			Bacteria	Firmicutes	Bacilli	Lactobacillales	Lactobacillaceae	Lactobacillus	Lactobacillus paraplantarum (T); DSM 10667T; AJ306297
			Bacteria	Firmicutes	Bacilli	Lactobacillales	Lactobacillaceae	Lactobacillus	Lactobacillus plantarum; HOKKAIDO; AB183696
			Bacteria	Firmicutes	Bacilli	Lactobacillales	Lactobacillaceae	Lactobacillus	Lactobacillus plantarum; UK782; from a traditional fermented milk; AJ271852
			Bacteria	Firmicutes	Clostridia	Clostridiales	Ruminococcaceae	Faecalibacterium	uncultured bacterium; p-2576-9F5; AF371734
			Bacteria	Firmicutes	Bacilli	Lactobacillales	Lactobacillaceae	Lactobacillus	Lactobacillus plantarum; NCDO 1752; X52653
			Bacteria	Firmicutes	Bacilli	Lactobacillales	Lactobacillaceae	Lactobacillus	Lactobacillus plantarum; MiLAB14; AY383631
			Bacteria	Firmicutes	Bacilli	Lactobacillales	Lactobacillaceae	Lactobacillus	Lactobacillus sp. YE07; AF316586
o1455	o1455 16S-LacGroup7	GGGCGACTTGAGTGCAGAA	Bacteria	Firmicutes	Bacilli	Lactobacillales	Lactobacillaceae	Lactobacillus	Lactobacillus frumenti (T); TMW 1.666; AJ250074
			Bacteria	Firmicutes	Bacilli	Lactobacillales	Lactobacillaceae	Lactobacillus	Lactobacillus sp. oral clone CX036; AY005048
			Bacteria	Firmicutes	Bacilli	Lactobacillales	Lactobacillaceae	Lactobacillus	uncultured bacterium; rRNA138; AY958911
			Bacteria	Firmicutes	Bacilli	Lactobacillales	Lactobacillaceae	Lactobacillus	Lactobacillus reuteri; DSM 20016 T; X76328
			Bacteria	Firmicutes	Bacilli	Lactobacillales	Lactobacillaceae	Lactobacillus	Lactobacillus reuteri (T); L23507
			Bacteria	Firmicutes	Bacilli	Lactobacillales	Lactobacillaceae	Lactobacillus	uncultured bacterium; S25-5; AJ308392
			Bacteria	Firmicutes	Bacilli	Lactobacillales	Lactobacillaceae	Lactobacillus	Lactobacillus reuteri; LU3; AY735406
			Bacteria	Firmicutes	Bacilli	Lactobacillales	Lactobacillaceae	Lactobacillus	Lactobacillus vaginalis; ATCC49540; AF243177
			Bacteria	Firmicutes	Bacilli	Lactobacillales	Lactobacillaceae	Lactobacillus	Lactobacillus vaginalis; KC19; AF243154
			Bacteria	Firmicutes	Bacilli	Lactobacillales	Lactobacillaceae	Lactobacillus	Lactobacillus oris (T); DSM 4864T; X94229
			Bacteria	Firmicutes	Bacilli	Lactobacillales	Lactobacillaceae	Lactobacillus	uncultured bacterium; p-165-a2; AF371482
			Bacteria	Firmicutes	Bacilli	Lactobacillales	Lactobacillaceae	Lactobacillus	Lactobacillus antri (T); Kx146A4; LMG 22111T; DSM 16041T; CCUG 48456T; AY2536
			Bacteria	Firmicutes	Bacilli	Lactobacillales	Lactobacillaceae	Lactobacillus	Lactobacillus sp. 34-10B; AY960573
			Bacteria	Firmicutes	Bacilli	Lactobacillales	Lactobacillaceae	Lactobacillus	uncultured bacterium; p-2694-65A5; AF371483
			Bacteria	Firmicutes	Bacilli	Lactobacillales	Lactobacillaceae	Lactobacillus	Lactobacillus panis (T); DSM 6035T; X94230
			Bacteria	Firmicutes	Bacilli	Lactobacillales	Lactobacillaceae	Lactobacillus	uncultured bacterium; p-3979-6Wa2; AF371485
			Bacteria	Firmicutes	Bacilli	Lactobacillales	Lactobacillaceae	Lactobacillus	uncultured bacterium; p-3301-23G2; AF371484
			Bacteria	Firmicutes	Bacilli	Bacillales	Paenibacillaceae	Paenibacillus	Paenibacillus sp. PAMU-2.5; AB118225
			Bacteria	Firmicutes	Bacilli	Lactobacillales	Lactobacillaceae	Lactobacillus	Lactobacillus vaginalis (T); NCTC 12197; X61136
			Bacteria	Firmicutes	Bacilli	Lactobacillales	Lactobacillaceae	Lactobacillus	Lactobacillus oris; NCDO 2160 (T); X61131
o1456	o1456 16S-LacMuc	AAGAACGTGCGTGAGAGCAA	Bacteria	Firmicutes	Bacilli	Lactobacillales	Lactobacillaceae	Lactobacillus	Lactobacillus sp. G24; AF197126
			Bacteria	Firmicutes	Bacilli	Lactobacillales	Lactobacillaceae	Lactobacillus	Lactobacillus mucosae (T); CCUG 43179 (T); S32; DSM 13345; AF126738
			Bacteria	Firmicutes	Bacilli	Lactobacillales	Lactobacillaceae	Lactobacillus	Lactobacillus reuteri; ATCC 55730; EU394679
			Bacteria	Firmicutes	Bacilli	Lactobacillales	Lactobacillaceae	Lactobacillus	Lactobacillus mucosae; LAB87; EF120376
			Bacteria	Firmicutes	Bacilli	Lactobacillales	Lactobacillaceae	Lactobacillus	Lactobacillus mucosae; RA2087; AY445126
			Bacteria	Firmicutes	Bacilli	Lactobacillales	Lactobacillaceae	Lactobacillus	Lactobacillus mucosae; RA2071; AY445125
			Bacteria	Firmicutes	Bacilli	Lactobacillales	Lactobacillaceae	Lactobacillus	Lactobacillus mucosae; RA2070; AY445124
			Bacteria	Firmicutes	Bacilli	Lactobacillales	Lactobacillaceae	Lactobacillus	uncultured Lactobacillus sp.; 16a; EF593047
			Bacteria	Firmicutes	Bacilli	Lactobacillales	Lactobacillaceae	Lactobacillus	Lactobacillus mucosae; BLB1c; AF243145
			Bacteria	Firmicutes	Bacilli	Lactobacillales	Lactobacillaceae	Lactobacillus	Lactobacillus mucosae; F52; AF243148
			Bacteria	Firmicutes	Bacilli	Lactobacillales	Lactobacillaceae	Lactobacillus	Lactobacillus mucosae; DJF_WC35; EU728797
			Bacteria	Firmicutes	Bacilli	Lactobacillales	Lactobacillaceae	Lactobacillus	uncultured bacterium; p-1883-s962-3; AF371488
			Bacteria	Firmicutes	Bacilli	Lactobacillales	Lactobacillaceae	Lactobacillus	Lactobacillus mucosae; DLC 0203; AB186326
			Bacteria	Firmicutes	Bacilli	Lactobacillales	Lactobacillaceae	Lactobacillus	Lactobacillus sp. KLDS 1.0718; EU600922
			Bacteria	Firmicutes	Bacilli	Lactobacillales	Lactobacillaceae	Lactobacillus	Lactobacillus fermentum; KLDS 1.0733; EU626018
			Bacteria	Firmicutes	Bacilli	Lactobacillales	Lactobacillaceae	Lactobacillus	Lactobacillus sp. KLDS 1.0716; EU600921
			Bacteria	Firmicutes	Bacilli	Lactobacillales	Lactobacillaceae	Lactobacillus	Lactobacillus mucosae; DLS 1003; AB186315
			Bacteria	Firmicutes	Bacilli	Lactobacillales	Lactobacillaceae	Lactobacillus	Lactobacillus mucosae; DLC 1110; AB186317
			Bacteria	Firmicutes	Bacilli	Lactobacillales	Lactobacillaceae	Lactobacillus	uncultured bacterium; VWP_aaa02f05; EU475078
			Bacteria	Firmicutes	Bacilli	Lactobacillales	Lactobacillaceae	Lactobacillus	Lactobacillus mucosae; 40E; DQ471799
o1457	o1457 16S-LacRha	CTCGGCTTAACCGAAGAAGT	Bacteria	Firmicutes	Bacilli	Lactobacillales	Lactobacillaceae	Lactobacillus	Lactobacillus reuteri (T); L23507
			Bacteria	Firmicutes	Bacilli	Lactobacillales	Lactobacillaceae	Lactobacillus	Lactobacillus rhamnosus; F11; AF243146
			Bacteria	Firmicutes	Bacilli	Lactobacillales	Lactobacillaceae	Lactobacillus	uncultured bacterium; SHZO766; GQ155670
o1458	o1458 16S-LaGroup2	TGTCACTTTAGGATGAGCCC	Bacteria	Firmicutes	Bacilli	Lactobacillales	Lactobacillaceae	Lactobacillus	Lactobacillus delbrueckii subsp. bulgaricus; LGM2; AY675257
			Bacteria	Firmicutes	Bacilli	Lactobacillales	Lactobacillaceae	Lactobacillus	Lactobacillus delbrueckii subsp. bulgaricus; DL2; AB200872
			Bacteria	Firmicutes	Bacilli	Lactobacillales	Lactobacillaceae	Lactobacillus	Lactobacillus delbrueckii subsp. delbrueckii; BCRC12195; AY773949
			Bacteria	Firmicutes	Bacilli	Lactobacillales	Lactobacillaceae	Lactobacillus	Lactobacillus delbrueckii subsp. bulgaricus; LG1; AY735407
			Bacteria	Firmicutes	Bacilli	Lactobacillales	Lactobacillaceae	Lactobacillus	Lactobacillus delbrueckii subsp. indicus (T); NCC725; AY421720
			Bacteria	Firmicutes	Bacilli	Lactobacillales	Lactobacillaceae	Lactobacillus	Lactobacillus delbrueckii subsp. delbrueckii; ATCC 9649; AY050172

			Bacteria	Firmicutes	Bacilli	Lactobacillales	Lactobacillaceae	Lactobacillus	Lactobacillus delbrueckii subsp. lactis (T); M58823
			Bacteria	Firmicutes	Bacilli	Lactobacillales	Lactobacillaceae	Lactobacillus	Lactobacillus delbrueckii; JCM 1002; AB007908
			Bacteria	Firmicutes	Bacilli	Lactobacillales	Lactobacillaceae	Lactobacillus	Lactobacillus delbrueckii subsp. bulgaricus (T); ATCC 11842; AY050171
			Bacteria	Firmicutes	Bacilli	Lactobacillales	Lactobacillaceae	Lactobacillus	Lactobacillus delbrueckii subsp. lactis; BCRC11051; AY773950
			Bacteria	Firmicutes	Bacilli	Lactobacillales	Lactobacillaceae	Lactobacillus	Lactobacillus delbrueckii subsp. lactis; ATCC 12315; AY050173
			Bacteria	Firmicutes	Bacilli	Lactobacillales	Lactobacillaceae	Lactobacillus	Lactobacillus delbrueckii subsp. bulgaricus; BCRC10696; AY773948
			Bacteria	Firmicutes	Bacilli	Lactobacillales	Lactobacillaceae	Lactobacillus	uncultured bacterium; p-2370-55G5; AF371474
			Bacteria	Firmicutes	Bacilli	Lactobacillales	Lactobacillaceae	Lactobacillus	uncultured bacterium; 51CR; AF349922
			Bacteria	Firmicutes	Bacilli	Lactobacillales	Lactobacillaceae	Lactobacillus	uncultured bacterium; 54CR; AF349925
			Bacteria	Firmicutes	Bacilli	Lactobacillales	Lactobacillaceae	Lactobacillus	uncultured bacterium; 37CR; AF349919
			Bacteria	Firmicutes	Bacilli	Lactobacillales	Lactobacillaceae	Lactobacillus	Lactobacillus delbrueckii (T); M58814
			Bacteria	Firmicutes	Bacilli	Lactobacillales	Lactobacillaceae	Lactobacillus	uncultured bacterium; p-4729-2Wb2; AF371475
			Bacteria	Firmicutes	Bacilli	Lactobacillales	Lactobacillaceae	Lactobacillus	Lactobacillus delbrueckii; NCDO 213; X52654
			Bacteria	Firmicutes	Bacilli	Lactobacillales	Lactobacillaceae	Lactobacillus	Lactobacillus sp. CY1; AY165006
o1459	o1459 16S-LaGroup3	CGGTAATCAACCAGAAAGTC	Bacteria	Firmicutes	Bacilli	Lactobacillales	Lactobacillaceae	Lactobacillus	Lactobacillus amylolyticus (T); DSM 1664 T; Y17361
			Bacteria	Firmicutes	Bacilli	Lactobacillales	Lactobacillaceae	Lactobacillus	Lactobacillus crispatus (T); DSM 20584 T; Y17362
			Bacteria	Firmicutes	Bacilli	Lactobacillales	Lactobacillaceae	Lactobacillus	Lactobacillus gallinarum; ATCC 33199; AJ417737
			Bacteria	Firmicutes	Bacilli	Lactobacillales	Lactobacillaceae	Lactobacillus	Lactobacillus kefiranofaciens subsp. kefirgranum; CECT 5919T; AJ575742
			Bacteria	Firmicutes	Bacilli	Lactobacillales	Lactobacillaceae	Lactobacillus	Lactobacillus crispatus; NCTC 4; AJ242969
			Bacteria	Firmicutes	Bacilli	Lactobacillales	Lactobacillaceae	Lactobacillus	uncultured bacterium; L10-37; AJ308390
			Bacteria	Firmicutes	Bacilli	Lactobacillales	Lactobacillaceae	Lactobacillus	Lactobacillus helveticus; NCIMB 11971; AY369116
			Bacteria	Firmicutes	Bacilli	Lactobacillales	Lactobacillaceae	Lactobacillus	Lactobacillus intestinalis (T); DSM 6629T; AJ306299
			Bacteria	Firmicutes	Bacilli	Lactobacillales	Lactobacillaceae	Lactobacillus	Lactobacillus hamsteri (T); DSM 5661T; AJ306298
			Bacteria	Firmicutes	Bacilli	Lactobacillales	Lactobacillaceae	Lactobacillus	Lactobacillus kitasatonis; KM9212; AB107637
			Bacteria	Firmicutes	Bacilli	Lactobacillales	Lactobacillaceae	Lactobacillus	Lactobacillus helveticus; M4; AY644397
			Bacteria	Firmicutes	Bacilli	Lactobacillales	Lactobacillaceae	Lactobacillus	Lactobacillus kefiranofaciens subsp. kefiranofaciens; type strain: LMG 19149; AJ575259
			Bacteria	Firmicutes	Bacilli	Lactobacillales	Lactobacillaceae	Lactobacillus	Lactobacillus kefiranofaciens subsp. kefiranofaciens; R-14703; AJ575260
			Bacteria	Firmicutes	Bacilli	Lactobacillales	Lactobacillaceae	Lactobacillus	Lactobacillus kefiranofaciens subsp. kefiranofaciens; R-12929; AJ575262
			Bacteria	Firmicutes	Bacilli	Lactobacillales	Lactobacillaceae	Lactobacillus	Lactobacillus kitasatonis (T); JCM 1039; AB107638
			Bacteria	Firmicutes	Bacilli	Lactobacillales	Lactobacillaceae	Lactobacillus	Lactobacillus kefiranofaciens subsp. kefiranofaciens; type strain: LMG 15132; AJ575261
			Bacteria	Firmicutes	Bacilli	Lactobacillales	Lactobacillaceae	Lactobacillus	Lactobacillus sp. GV6; E.colipTZ57R_16S5E/3E; AY330709
			Bacteria	Firmicutes	Bacilli	Lactobacillales	Lactobacillaceae	Lactobacillus	Lactobacillus fornicalis (T); TV1018; Y18654
			Bacteria	Firmicutes	Bacilli	Lactobacillales	Lactobacillaceae	Lactobacillus	Lactobacillus delbrueckii; NCDO 213; X52654
			Bacteria	Firmicutes	Bacilli	Lactobacillales	Lactobacillaceae	Lactobacillus	Lactobacillus acidophilus; NCDO 1748 (T); X61138
o1460	o1460 16S-LaGroup4	TTGTTACTAGTTGCCAGCAT	Bacteria	Firmicutes	Bacilli	Lactobacillales	Lactobacillaceae	Lactobacillus	Lactobacillus pontis; LTH 2587; X76329
			Bacteria	Firmicutes	Bacilli	Lactobacillales	Lactobacillaceae	Lactobacillus	Lactobacillus frumenti (T); TMW 1.666; AJ250074
			Bacteria	Firmicutes	Bacilli	Lactobacillales	Lactobacillaceae	Lactobacillus	Lactobacillus sp. oral clone CX036; AY005048
			Bacteria	Firmicutes	Bacilli	Lactobacillales	Lactobacillaceae	Lactobacillus	Lactobacillus fermentum; MD-9; AY373589
			Bacteria	Firmicutes	Bacilli	Lactobacillales	Lactobacillaceae	Lactobacillus	Lactobacillus fermentum (T); AJ575812
			Bacteria	Firmicutes	Bacilli	Lactobacillales	Lactobacillaceae	Lactobacillus	Lactobacillus reuteri; DSM 20016 T; X76328
			Bacteria	Firmicutes	Bacilli	Lactobacillales	Lactobacillaceae	Lactobacillus	uncultured bacterium; S25-5; AJ308392
			Bacteria	Firmicutes	Bacilli	Lactobacillales	Lactobacillaceae	Lactobacillus	uncultured bacterium; p-3083-SwA-3; AF371487
			Bacteria	Firmicutes	Bacilli	Lactobacillales	Lactobacillaceae	Lactobacillus	uncultured bacterium; p-1883-s962-3; AF371488
			Bacteria	Firmicutes	Bacilli	Lactobacillales	Lactobacillaceae	Lactobacillus	Lactobacillus sp. CR-6AS; crev-7; AF349935
			Bacteria	Firmicutes	Bacilli	Lactobacillales	Lactobacillaceae	Lactobacillus	Lactobacillus oris (T); DSM 4864T; X94229
			Bacteria	Firmicutes	Bacilli	Lactobacillales	Lactobacillaceae	Lactobacillus	uncultured bacterium; p-165-a2; AF371482
			Bacteria	Firmicutes	Bacilli	Lactobacillales	Lactobacillaceae	Lactobacillus	uncultured bacterium; 60CR; AF349926
			Bacteria	Firmicutes	Bacilli	Lactobacillales	Lactobacillaceae	Lactobacillus	uncultured bacterium; p-4312-4Wa3; AF371486
			Bacteria	Firmicutes	Bacilli	Lactobacillales	Lactobacillaceae	Lactobacillus	uncultured bacterium; p-2694-65A5; AF371483
			Bacteria	Firmicutes	Bacilli	Lactobacillales	Lactobacillaceae	Lactobacillus	Lactobacillus panis (T); DSM 6035T; X94230
			Bacteria	Firmicutes	Bacilli	Lactobacillales	Lactobacillaceae	Lactobacillus	Lactobacillus reuteri; OJU5; AY324629
			Bacteria	Firmicutes	Bacilli	Lactobacillales	Lactobacillaceae	Lactobacillus	uncultured bacterium; p-3301-23G2; AF371484
			Bacteria	Firmicutes	Bacilli	Lactobacillales	Lactobacillaceae	Lactobacillus	Lactobacillus reuteri; ONK5; AY324628
			Bacteria	Firmicutes	Bacilli	Lactobacillales	Lactobacillaceae	Lactobacillus	Lactobacillus coleohominis; AJ292530
o1461	o1461 16S-LaGroup5	GAAGGGGATAACACCTGGAA	Bacteria	Firmicutes	Bacilli	Lactobacillales	Lactobacillaceae	Lactobacillus	Lactobacillus sanfranciscensis (T); ATCC 27651 T; X76327
			Bacteria	Firmicutes	Bacilli	Lactobacillales	Lactobacillaceae	Lactobacillus	Lactobacillus lindneri; L2; X95422
			Bacteria	Firmicutes	Bacilli	Lactobacillales	Lactobacillaceae	Lactobacillus	Lactobacillus lindneri; L40; X95423
			Bacteria	Firmicutes	Bacilli	Lactobacillales	Lactobacillaceae	Lactobacillus	Lactobacillus lindneri (T); DSM 20690; X95421
			Bacteria	Firmicutes	Bacilli	Lactobacillales	Lactobacillaceae	Lactobacillus	Lactobacillus sanfranciscensis; DSM 20663; X76331
			Bacteria	Firmicutes	Bacilli	Lactobacillales	Lactobacillaceae	Lactobacillus	Lactobacillus fructivorans; DSM 20203 T; X76330
			Bacteria	Firmicutes	Bacilli	Lactobacillales	Lactobacillaceae	Lactobacillus	Lactobacillus homohiochii (T); type strain:DSM 20571; AM113780
			Bacteria	Firmicutes	Bacilli	Lactobacillales	Lactobacillaceae	Lactobacillus	uncultured Lactobacillus sp.; KL-11-1-6; AF408267
			Bacteria	Firmicutes	Bacilli	Lactobacillales	Lactobacillaceae	Lactobacillus	uncultured Lactobacillus sp.; KL-111-2-6; AF408266
			Bacteria	Firmicutes	Bacilli	Lactobacillales	Lactobacillaceae	Lactobacillus	Lactobacillus lindneri; DA1; D37784
			Bacteria	Firmicutes	Bacilli	Lactobacillales	Lactobacillaceae	Lactobacillus	Lactobacillus sanfranciscensis; C57 = R-15351; AJ422037
			Bacteria	Firmicutes	Bacilli	Lactobacillales	Lactobacillaceae	Lactobacillus	Lactobacillus fructivorans (T); M58818
			Bacteria	Firmicutes	Bacilli	Lactobacillales	Lactobacillaceae	Lactobacillus	Lactobacillus sanfranciscensis; M58830
			Bacteria	Firmicutes	Clostridia	Clostridiales	Lachnospiraceae	unclassified_"Lachnospiraceae"	uncultured bacterium; obob1_aaa03c08; EF096245
			Bacteria	Firmicutes	Clostridia	Clostridiales	Lachnospiraceae	unclassified_"Lachnospiraceae"	uncultured bacterium; obob1_aaa02e08; EF096208
			Bacteria	Firmicutes	Clostridia	Clostridiales	Lachnospiraceae	unclassified_"Lachnospiraceae"	uncultured bacterium; obob2_aaa02a07; EF096388
			Bacteria	Firmicutes	Clostridia	Clostridiales	Lachnospiraceae	unclassified_"Lachnospiraceae"	uncultured bacterium; obob1_aaa01c04; EF096142
			Bacteria	Firmicutes	Clostridia	Clostridiales	Lachnospiraceae	unclassified_"Lachnospiraceae"	uncultured bacterium; C10_F05; AY991395
			Bacteria	Firmicutes	Clostridia	Clostridiales	Lachnospiraceae	unclassified_"Lachnospiraceae"	uncultured bacterium; obob1_aaa04h05; EF096322
			Bacteria	Firmicutes	Bacilli	Lactobacillales	Lactobacillaceae	Lactobacillus	Lactobacillus sanfranciscensis; JCM 5668; X61132
o1462	o1462 16S-LaGroup6	ACGAGTGGCGAACTGGTGA	Bacteria	Firmicutes	Bacilli	Lactobacillales	Lactobacillaceae	Lactobacillus	Lactobacillus sp. rennanqilfy19; AY363377
			Bacteria	Firmicutes	Bacilli	Lactobacillales	Lactobacillaceae	Lactobacillus	Lactobacillus parabuchneri; JCM12493; AB205056
			Bacteria	Firmicutes	Bacilli	Lactobacillales	Lactobacillaceae	Lactobacillus	Lactobacillus sp. MD-1; AY496039
			Bacteria	Firmicutes	Bacilli	Lactobacillales	Lactobacillaceae	Lactobacillus	Lactobacillus buchneri; JCM1115; AB205055
			Bacteria	Firmicutes	Bacilli	Lactobacillales	Lactobacillaceae	Lactobacillus	Lactobacillus spicheri (T); type strain: LTH 5753; AJ534844
			Bacteria	Firmicutes	Bacilli	Lactobacillales	Lactobacillaceae	Lactobacillus	Lactobacillus genomosp. C1; C4M_55; AY278619
			Bacteria	Firmicutes	Bacilli	Lactobacillales	Lactobacillaceae	Lactobacillus	Lactobacillus kefiri; AY363303
			Bacteria	Firmicutes	Bacilli	Lactobacillales	Lactobacillaceae	Lactobacillus	Lactobacillus zymae (T); type strain: LMG 22198 = R-18615; AJ632157
			Bacteria	Firmicutes	Bacilli	Lactobacillales	Lactobacillaceae	Lactobacillus	Lactobacillus genomosp. C2; C4M_47; AY278620
			Bacteria	Firmicutes	Bacilli	Lactobacillales	Lactobacillaceae	Lactobacillus	Lactobacillus zymae; ACA-DC 3411t1 = R-13317; AJ422039
			Bacteria	Firmicutes	Bacilli	Lactobacillales	Lactobacillaceae	Lactobacillus	Lactobacillus parabuchneri (T); R7-84(T); AF275311
			Bacteria	Firmicutes	Bacilli	Lactobacillales	Lactobacillaceae	Lactobacillus	Lactobacillus hilgardii; IOEB0204; AY241664
			Bacteria	Firmicutes	Bacilli	Lactobacillales	Lactobacillaceae	Lactobacillus	Lactobacillus kefiri; NRIC 1693; AB024300
			Bacteria	Firmicutes	Bacilli	Lactobacillales	Lactobacillaceae	Lactobacillus	Lactobacillus sp. ACD7; AY026752

			Bacteria	Firmicutes	Bacilli	Lactobacillales	Lactobacillaceae	Lactobacillus	Lactobacillus sp. JKD6; AF264701
			Bacteria	Firmicutes	Bacilli	Lactobacillales	Lactobacillaceae	Lactobacillus	Lactobacillus kefiri; JCM 5818; AY579584
			Bacteria	Firmicutes	Bacilli	Lactobacillales	Lactobacillaceae	Lactobacillus	Lactobacillus parakefiri (T); LMG 15133T; AY026750
			Bacteria	Firmicutes	Bacilli	Lactobacillales	Lactobacillaceae	Lactobacillus	Lactobacillus vermiforme; M59295
			Bacteria	Firmicutes	Bacilli	Lactobacillales	Lactobacillaceae	Lactobacillus	Lactobacillus hilgardii (T); M58821
			Bacteria	Firmicutes	Bacilli	Lactobacillales	Lactobacillaceae	Lactobacillus	Lactobacillus parabuchneri; LMG 11457T; AY026751
o1463	o1463 16S-LauOral	AACCTTGGAGAGATTCGAGG	Bacteria	Proteobacteria	Betaproteobacteria	Burkholderiales	unclassified_Burkholderiales		uncultured Lautropia sp.; 2.6; DQ016727
			Bacteria	Proteobacteria	Betaproteobacteria	Burkholderiales	unclassified_Burkholderiales		uncultured bacterium; BF0001D034; AM697419
			Bacteria	Proteobacteria	Betaproteobacteria	Burkholderiales	Alcaligenaceae	Derxia	Lautropia sp. oral clone AP009; AY005030
			Bacteria	Proteobacteria	Betaproteobacteria	Burkholderiales	Alcaligenaceae	Derxia	uncultured Lautropia sp.; 24B473; FJ976403
o1464	o1464 16S-LcGroup2	AGTAGGTTGCCTAACCGCAA	Bacteria	Firmicutes	Bacilli	Lactobacillales	Streptococcaceae	Lactococcus	Lactococcus lactis; Akira2; AY348313
			Bacteria	Firmicutes	Bacilli	Lactobacillales	Streptococcaceae	Lactococcus	Lactococcus lactis; ssp. IL 1403; X64887
			Bacteria	Firmicutes	Bacilli	Lactobacillales	Streptococcaceae	Lactococcus	swine manure bacterium RT-3A; AY167937
			Bacteria	Firmicutes	Bacilli	Lactobacillales	Streptococcaceae	Lactococcus	Lactococcus garvieae; M79; AY699289
			Bacteria	Firmicutes	Bacilli	Lactobacillales	Streptococcaceae	Lactococcus	swine manure bacterium RT-6B; AY167948
			Bacteria	Firmicutes	Bacilli	Lactobacillales	Streptococcaceae	Lactococcus	Lactococcus lactis subsp. lactis; K231; AB083092
			Bacteria	Firmicutes	Bacilli	Lactobacillales	Streptococcaceae	Lactococcus	Lactococcus lactis subsp. lactis; K337; AB118035
			Bacteria	Firmicutes	Bacilli	Lactobacillales	Streptococcaceae	Lactococcus	Lactococcus lactis subsp. lactis; K338; AB118036
			Bacteria	Firmicutes	Bacilli	Lactobacillales	Streptococcaceae	Lactococcus	Lactococcus lactis subsp. lactis; K336; AB118034
			Bacteria	Firmicutes	Bacilli	Lactobacillales	Streptococcaceae	Lactococcus	Lactococcus lactis subsp. lactis; K335; AB118033
			Bacteria	Firmicutes	Bacilli	Lactobacillales	Streptococcaceae	Lactococcus	Lactococcus garvieae; JCM 8735; AB012306
			Bacteria	Firmicutes	Bacilli	Lactobacillales	Streptococcaceae	Lactococcus	uncultured bacterium; AJ487019
			Bacteria	Firmicutes	Bacilli	Lactobacillales	Streptococcaceae	Lactococcus	Enterococcus sp. U1-1; AB079369
			Bacteria	Firmicutes	Bacilli	Lactobacillales	Streptococcaceae	Lactococcus	Lactococcus lactis; MRS2; AJ488174
			Bacteria	Firmicutes	Bacilli	Lactobacillales	Streptococcaceae	Lactococcus	uncultured Lactococcus sp.; KL-86-2-7; AF408259
			Bacteria	Firmicutes	Bacilli	Lactobacillales	Streptococcaceae	Lactococcus	Lactococcus lactis subsp. cremoris; CF4; AB181302
			Bacteria	Firmicutes	Bacilli	Lactobacillales	Streptococcaceae	Lactococcus	Lactococcus lactis; MRS1; AJ488173
			Bacteria	Firmicutes	Bacilli	Lactobacillales	Streptococcaceae	Lactococcus	Lactococcus lactis; MRS5; AJ488175
			Bacteria	Firmicutes	Bacilli	Lactobacillales	Streptococcaceae	Lactococcus	Lactococcus lactis; 04; AJ488176
			Bacteria	Firmicutes	Bacilli	Lactobacillales	Streptococcaceae	Lactococcus	Lactococcus lactis; UK1560; from a traditional fermented milk; AJ271851
o1465	o1465 16S-Lecea	ACCTACCCTTGACATACAGT	Bacteria	Proteobacteria	Gammaproteobacteria	Legionellales	Legionellaceae	Legionella	Legionella lytica; PCM 2298; X66835
			Bacteria	Proteobacteria	Gammaproteobacteria	Legionellales	Legionellaceae	Legionella	Legionella sp.; LLAP11; X97362
			Bacteria	Proteobacteria	Gammaproteobacteria	Legionellales	Legionellaceae	Legionella	Legionella lytica (T); L2; X97364
			Bacteria	Proteobacteria	Gammaproteobacteria	Legionellales	Legionellaceae	Legionella	Legionella erythra (T); Z32638
			Bacteria	Proteobacteria	Gammaproteobacteria	Legionellales	Legionellaceae	Legionella	Legionella cherrii (T); ATCC 35252; X73404
			Bacteria	Proteobacteria	Gammaproteobacteria	Legionellales	Legionellaceae	Legionella	Legionella israelensis (T); ATCC 43119; X73408
			Bacteria	Proteobacteria	Gammaproteobacteria	Legionellales	Legionellaceae	Legionella	Legionella sainthelensi (T); ATCC 35248; X73399
			Bacteria	Proteobacteria	Gammaproteobacteria	Legionellales	Legionellaceae	Legionella	Legionella sp.; LLAP7; X97365
			Bacteria	Proteobacteria	Gammaproteobacteria	Legionellales	Legionellaceae	Legionella	Legionella jamestowniensis (T); ATCC 35298; X73409
			Bacteria	Proteobacteria	Gammaproteobacteria	Legionellales	Legionellaceae	Legionella	Legionella rubrilucens (T); ATCC 35304; X73398
			Bacteria	Proteobacteria	Gammaproteobacteria	Legionellales	Legionellaceae	Legionella	Legionella wadsworthii (T); ATCC 33877; X73401
			Bacteria	Proteobacteria	Gammaproteobacteria	Legionellales	Legionellaceae	Legionella	Legionella quinlivanii; sreogroup 2, nctc 12433; Z49733
			Bacteria	Proteobacteria	Gammaproteobacteria	Legionellales	Legionellaceae	Legionella	Legionella adelaidensis; NCTC 12735; Z49716
			Bacteria	Proteobacteria	Gammaproteobacteria	Legionellales	Legionellaceae	Legionella	Legionella jamestowniensis; nctc 11981; Z49726
			Bacteria	Proteobacteria	Gammaproteobacteria	Legionellales	Legionellaceae	Legionella	Legionella gratiana; nctc 12388; Z49725
			Bacteria	Proteobacteria	Gammaproteobacteria	Legionellales	Legionellaceae	Legionella	Legionella moravica; nctc 12239; Z49729
			Bacteria	Proteobacteria	Gammaproteobacteria	Legionellales	Legionellaceae	Legionella	Legionella feeleii (T); ATCC 35072; X73395
			Bacteria	Proteobacteria	Gammaproteobacteria	Legionellales	Legionellaceae	Legionella	Legionella parisiensis; nctc 11983; Z49731
			Bacteria	Proteobacteria	Gammaproteobacteria	Legionellales	Legionellaceae	Legionella	Legionella anisa; Z32635
			Bacteria	Proteobacteria	Gammaproteobacteria	Legionellales	Legionellaceae	Legionella	Legionella sp.; LLAP4; X97357
o1466	o1466 16S-LeGroup1	TTCCGCCTCTTAGTGCCGA	Bacteria	Firmicutes	Bacilli	Lactobacillales	Leuconostocaceae	Leuconostoc	Leuconostoc garlicum; a new species; AY456086
			Bacteria	Firmicutes	Bacilli	Lactobacillales	Leuconostocaceae	Leuconostoc	Leuconostoc kimchii (T); AF173986
			Bacteria	Firmicutes	Bacilli	Lactobacillales	Leuconostocaceae	Leuconostoc	Leuconostoc gelidum (T); DSM5578; AF175402
			Bacteria	Firmicutes	Bacilli	Lactobacillales	Leuconostocaceae	Leuconostoc	Leuconostoc gelidum; JCM10093; AB004661
			Bacteria	Firmicutes	Bacilli	Lactobacillales	Leuconostocaceae	Leuconostoc	uncultured bacterium; LB1C2; AY186084
			Bacteria	Firmicutes	Bacilli	Lactobacillales	Leuconostocaceae	Leuconostoc	Leuconostoc lactis (T); DSM8581; AF175403
			Bacteria	Firmicutes	Bacilli	Lactobacillales	Leuconostocaceae	Leuconostoc	Leuconostoc mesenteroides; NCFB 543; AB023247
			Bacteria	Firmicutes	Bacilli	Lactobacillales	Leuconostocaceae	Leuconostoc	Leuconostoc mesenteroides; NRIC 1539; AB023246
			Bacteria	Firmicutes	Bacilli	Lactobacillales	Leuconostocaceae	Leuconostoc	Leuconostoc lactis; JCM 6123; AB023968
			Bacteria	Firmicutes	Bacilli	Lactobacillales	Leuconostocaceae	Leuconostoc	Leuconostoc mesenteroides; NRIC 1538; AB023245
			Bacteria	Firmicutes	Bacilli	Lactobacillales	Leuconostocaceae	Leuconostoc	Leuconostoc mesenteroides; NCFB 529; AB023244
			Bacteria	Firmicutes	Bacilli	Lactobacillales	Leuconostocaceae	Leuconostoc	Leuconostoc pseudomesenteroides; NRIC 1777; AB023237
			Bacteria	Firmicutes	Bacilli	Lactobacillales	Leuconostocaceae	Leuconostoc	Leuconostoc citreum; NRIC 1776; AB022923
			Bacteria	Firmicutes	Bacilli	Lactobacillales	Leuconostocaceae	Leuconostoc	Leuconostoc carnosum; NRIC 1722; AB022925
			Bacteria	Firmicutes	Bacilli	Lactobacillales	Leuconostocaceae	Leuconostoc	Leuconostoc gelidum; NRIC 1778; AB022921
			Bacteria	Firmicutes	Bacilli	Lactobacillales	Leuconostocaceae	Leuconostoc	Leuconostoc sp. CR-310S; crev-3; AF349931
			Bacteria	Firmicutes	Bacilli	Lactobacillales	Leuconostocaceae	Leuconostoc	Leuconostoc pseudomesenteroides (T); NCDO 768; X95979
			Bacteria	Firmicutes	Bacilli	Lactobacillales	Leuconostocaceae	Leuconostoc	Leuconostoc mesenteroides; NCDO 523; X95978
			Bacteria	Firmicutes	Bacilli	Lactobacillales	Leuconostocaceae	Leuconostoc	Leuconostoc carnosum (T); NCFB 2776; X95977
			Bacteria	Firmicutes	Bacilli	Lactobacillales	Leuconostocaceae	Leuconostoc	Leuconostoc citreum; NCFB 2787; X53963
o1467	o1467 16S-LeGroup2	TGTCCCGAGCGTTATCCG	Bacteria	Firmicutes	Bacilli	Lactobacillales	Leuconostocaceae	Leuconostoc	Leuconostoc garlicum; a new species; AY456086
			Bacteria	Firmicutes	Bacilli	Lactobacillales	Leuconostocaceae	Leuconostoc	Leuconostoc kimchii (T); AF173986
			Bacteria	Firmicutes	Bacilli	Lactobacillales	Leuconostocaceae	Leuconostoc	Leuconostoc gelidum (T); DSM5578; AF175402
			Bacteria	Firmicutes	Bacilli	Lactobacillales	Leuconostocaceae	Leuconostoc	Fructobacillus pseudoficulneus (T); LC51; AY169967
			Bacteria	Firmicutes	Bacilli	Lactobacillales	Leuconostocaceae	Leuconostoc	Leuconostoc fallax; 17D (CNRZ2049); AF218797
			Bacteria	Firmicutes	Bacilli	Lactobacillales	Leuconostocaceae	Leuconostoc	uncultured bacterium; LB1C2; AY186084
			Bacteria	Firmicutes	Bacilli	Lactobacillales	Leuconostocaceae	Leuconostoc	Fructobacillus ficulneus (T); FS-1; AF360736
			Bacteria	Firmicutes	Bacilli	Lactobacillales	Leuconostocaceae	Leuconostoc	Leuconostoc lactis (T); DSM8581; AF175403
			Bacteria	Firmicutes	Bacilli	Lactobacillales	Leuconostocaceae	Leuconostoc	Leuconostoc mesenteroides; NCFB 543; AB023247
			Bacteria	Firmicutes	Bacilli	Lactobacillales	Leuconostocaceae	Leuconostoc	Leuconostoc mesenteroides; NRIC 1539; AB023246
			Bacteria	Firmicutes	Bacilli	Lactobacillales	Leuconostocaceae	Leuconostoc	Leuconostoc lactis; JCM 6123; AB023968
			Bacteria	Firmicutes	Bacilli	Lactobacillales	Leuconostocaceae	Leuconostoc	Leuconostoc mesenteroides; NRIC 1538; AB023245
			Bacteria	Firmicutes	Bacilli	Lactobacillales	Leuconostocaceae	Leuconostoc	Leuconostoc mesenteroides; NCFB 529; AB023244
			Bacteria	Firmicutes	Bacilli	Lactobacillales	Leuconostocaceae	Leuconostoc	Leuconostoc pseudomesenteroides; NRIC 1777; AB023237
			Bacteria	Firmicutes	Bacilli	Lactobacillales	Leuconostocaceae	Leuconostoc	Leuconostoc fallax; NRIC 0210; AB023239
			Bacteria	Firmicutes	Bacilli	Lactobacillales	Leuconostocaceae	Leuconostoc	Leuconostoc citreum; NRIC 1776; AB022923
			Bacteria	Firmicutes	Bacilli	Lactobacillales	Leuconostocaceae	Leuconostoc	Leuconostoc carnosum; NRIC 1722; AB022925
			Bacteria	Firmicutes	Bacilli	Lactobacillales	Leuconostocaceae	Leuconostoc	Leuconostoc gelidum; NRIC 1778; AB022921

			Bacteria	Firmicutes	Bacilli	Lactobacillales	Leuconostocaceae	Leuconostoc	Leuconostoc citreum; 22R; CNRZ1478; AF195784
			Bacteria	Firmicutes	Bacilli	Lactobacillales	Leuconostocaceae	Leuconostoc	Leuconostoc sp. CR-310S; crev-3; AF349931
o1468	o1468 16S-LepOral1	AAGTCTCTGTGCCGAAGCAA	Bacteria	Fusobacteria	Fusobacteria	Fusobacteriales	Fusobacteriaceae	Sneathia	uncultured bacterium; rRNA141; AY958914
			Bacteria	Fusobacteria	Fusobacteria	Fusobacteriales	Fusobacteriaceae	Sneathia	Leptotrichia amnionii; AMN-1; AY078425
			Bacteria	Fusobacteria	Fusobacteria	Fusobacteriales	Fusobacteriaceae	Sneathia	uncultured Leptotrichia sp.; 531; EU644469
			Bacteria	Fusobacteria	Fusobacteria	Fusobacteriales	Fusobacteriaceae	Sneathia	Leptotrichia amnionii; AY489565
			Bacteria	Fusobacteria	Fusobacteria	Fusobacteriales	Fusobacteriaceae	Sneathia	uncultured bacterium; Y1; DQ666091
			Bacteria	Fusobacteria	Fusobacteria	Fusobacteriales	Fusobacteriaceae	Sneathia	uncultured Sneathia sp.; 301; EU644481
			Bacteria	Fusobacteria	Fusobacteria	Fusobacteriales	Fusobacteriaceae	Sneathia	uncultured bacterium; 032a; EU644451
			Bacteria	Fusobacteria	Fusobacteria	Fusobacteriales	Fusobacteriaceae	Sneathia	uncultured Sneathia sp.; 230; EU644479
			Bacteria	Fusobacteria	Fusobacteria	Fusobacteriales	Fusobacteriaceae	Sneathia	uncultured Leptotrichia sp.; MZH0805-11; EF120364
			Bacteria	Fusobacteria	Fusobacteria	Fusobacteriales	Fusobacteriaceae	Sneathia	uncultured Sneathia sp.; MZH0805-40; EF120363
			Bacteria	Fusobacteria	Fusobacteria	Fusobacteriales	Fusobacteriaceae	Sneathia	Sneathia sanguinegens (T); CCUG 41628T; AJ344093
			Bacteria	Fusobacteria	Fusobacteria	Fusobacteriales	Fusobacteriaceae	Sneathia	uncultured bacterium; nbu405e05c1; GQ037637
			Bacteria	Fusobacteria	Fusobacteria	Fusobacteriales	Fusobacteriaceae	Sneathia	uncultured bacterium; nbu405d05c1; GQ037631
			Bacteria	Fusobacteria	Fusobacteria	Fusobacteriales	Fusobacteriaceae	Sneathia	uncultured bacterium; nbu405g05c1; GQ037656
			Bacteria	Fusobacteria	Fusobacteria	Fusobacteriales	Fusobacteriaceae	Sneathia	uncultured bacterium; nbu405f05c1; GQ037645
			Bacteria	Fusobacteria	Fusobacteria	Fusobacteriales	Fusobacteriaceae	Sneathia	uncultured bacterium; nbu406a03c1; GQ037670
			Bacteria	Fusobacteria	Fusobacteria	Fusobacteriales	Fusobacteriaceae	Sneathia	uncultured bacterium; nbu405d08c1; GQ037633
			Bacteria	Fusobacteria	Fusobacteria	Fusobacteriales	Fusobacteriaceae	Sneathia	uncultured bacterium; nbu406e05c1; GQ038456
			Bacteria	Fusobacteria	Fusobacteria	Fusobacteriales	Fusobacteriaceae	Sneathia	Leptotrichia amnionii; CCUG 51846; EF218612
			Bacteria	Fusobacteria	Fusobacteria	Fusobacteriales	Fusobacteriaceae	Sneathia	Leptotrichia amnionii; CCUG 51847; EF218611
o1469	o1469 16S-LepOral2	ATGATTACTGGGTGTGGGCA	Bacteria	Fusobacteria	Fusobacteria	Fusobacteriales	Fusobacteriaceae	Leptotrichia	Leptotrichia sp. oral clone GT020; AY349385
			Bacteria	Fusobacteria	Fusobacteria	Fusobacteriales	Fusobacteriaceae	Leptotrichia	Leptotrichia sp. oral clone GT018; AY349384
			Bacteria	Fusobacteria	Fusobacteria	Fusobacteriales	Fusobacteriaceae	Leptotrichia	Leptotrichia sp. oral clone IK040; AY349387
			Bacteria	Fusobacteria	Fusobacteria	Fusobacteriales	Fusobacteriaceae	Leptotrichia	Leptotrichia sp.; AF189244
			Bacteria	Fusobacteria	Fusobacteria	Fusobacteriales	Fusobacteriaceae	Leptotrichia	Leptotrichia sp. oral clone DA069; AF287811
			Bacteria	Fusobacteria	Fusobacteria	Fusobacteriales	Fusobacteriaceae	Leptotrichia	Leptotrichia sp. oral clone FP036; AF432138
			Bacteria	Fusobacteria	Fusobacteria	Fusobacteriales	Fusobacteriaceae	Leptotrichia	Leptotrichia sp. oral clone EI022; AF385572
			Bacteria	Fusobacteria	Fusobacteria	Fusobacteriales	Fusobacteriaceae	Leptotrichia	Leptotrichia sp. oral clone EI013; AF385571
			Bacteria	Fusobacteria	Fusobacteria	Fusobacteriales	Fusobacteriaceae	Leptotrichia	Leptotrichia trevisanii; LB11; AY029801
			Bacteria	Fusobacteria	Fusobacteria	Fusobacteriales	Fusobacteriaceae	Leptotrichia	uncultured Leptotrichia sp.; DE081; AF366268
			Bacteria	Fusobacteria	Fusobacteria	Fusobacteriales	Fusobacteriaceae	Leptotrichia	Leptotrichia wadeii; LB16; AY029802
			Bacteria	Fusobacteria	Fusobacteria	Fusobacteriales	Fusobacteriaceae	Leptotrichia	Leptotrichia sp. oral clone BU064; AY008309
			Bacteria	Fusobacteria	Fusobacteria	Fusobacteriales	Fusobacteriaceae	Leptotrichia	Leptotrichia hofstadii (T); LB23; AY029803
			Bacteria	Fusobacteria	Fusobacteria	Fusobacteriales	Fusobacteriaceae	Leptotrichia	Leptotrichia trevisanii (T); AF206305
			Bacteria	Fusobacteria	Fusobacteria	Fusobacteriales	Fusobacteriaceae	Leptotrichia	Leptotrichia sp. oral clone F138; AF287814
			Bacteria	Fusobacteria	Fusobacteria	Fusobacteriales	Fusobacteriaceae	Leptotrichia	Leptotrichia genomosp. C1; C3MKM102; AY278621
			Bacteria	Fusobacteria	Fusobacteria	Fusobacteriales	Fusobacteriaceae	Leptotrichia	Leptotrichia sp. oral clone P2PB_51 P1; AY207053
			Bacteria	Fusobacteria	Fusobacteria	Fusobacteriales	Fusobacteriaceae	Leptotrichia	Leptotrichia buccalis; L37788
			Bacteria	Fusobacteria	Fusobacteria	Fusobacteriales	Fusobacteriaceae	Leptotrichia	human oral bacterium AC60; AF201978
			Bacteria	Fusobacteria	Fusobacteria	Fusobacteriales	Fusobacteriaceae	Leptotrichia	Leptotrichia buccalis (T); NCTC 10429; X90831
o1470	o1470 16S-LepSp	CTAATACCCCGGAGTGATGA	Bacteria	Proteobacteria	Betaproteobacteria	Burkholderiales	unclassified_Burkholderiales		Leptothrix sp. MBIC3364; Y8; AB015048
			Bacteria	Proteobacteria	Betaproteobacteria	Burkholderiales	Incertae sedis 5	unclassified_Incertae sedis 5	phenanthrene-degrading bacterium 90; AY177371
o1471	o1471 16S-LiGroup1	GTAAGTTGGGGATAACTCCG	Bacteria	Firmicutes	Bacilli	Bacillales	Listeriaceae	Listeria	Listeria monocytogenes; EGD-e; AL591983
			Bacteria	Firmicutes	Bacilli	Bacillales	Listeriaceae	Listeria	Listeria ivanovii; CLIP12510; X98528
			Bacteria	Firmicutes	Bacilli	Bacillales	Listeriaceae	Listeria	Listeria welshimeri; NCTC11857; X56149
			Bacteria	Firmicutes	Bacilli	Bacillales	Listeriaceae	Listeria	Listeria monocytogenes; LL195; X98530
			Bacteria	Firmicutes	Bacilli	Bacillales	Listeriaceae	Listeria	Listeria ivanovii; CLIP12229; X98529
			Bacteria	Firmicutes	Bacilli	Bacillales	Listeriaceae	Listeria	Listeria seeligeri; ATCC35976; X98531
			Bacteria	Firmicutes	Bacilli	Bacillales	Listeriaceae	Listeria	Listeria welshimeri (T); ATCC35897; X98532
			Bacteria	Firmicutes	Bacilli	Bacillales	Listeriaceae	Listeria	Listeria seeligeri; 50; AJ535698
			Bacteria	Firmicutes	Bacilli	Bacillales	Listeriaceae	Listeria	Listeria innocua (T); ATCC33090; X98527
			Bacteria	Firmicutes	Bacilli	Bacillales	Listeriaceae	Listeria	Listeria monocytogenes; 44; AJ535697
			Bacteria	Firmicutes	Bacilli	Bacillales	Listeriaceae	Listeria	Listeria monocytogenes; 97; AJ535701
			Bacteria	Firmicutes	Bacilli	Bacillales	Listeriaceae	Listeria	Listeria innocua; 169; AJ535693
			Bacteria	Firmicutes	Bacilli	Bacillales	Listeriaceae	Listeria	Listeria innocua; 107; AJ535695
			Bacteria	Firmicutes	Bacilli	Bacillales	Listeriaceae	Listeria	Listeria sp. 87; AJ535700
			Bacteria	Firmicutes	Bacilli	Bacillales	Listeriaceae	Listeria	Listeria welshimeri; 23; AJ535694
			Bacteria	Firmicutes	Bacilli	Bacillales	Listeriaceae	Listeria	Listeria seeligeri; 52; AJ535699
			Bacteria	Firmicutes	Bacilli	Bacillales	Listeriaceae	Listeria	Listeria monocytogenes (T); NCTC10357; X56153
			Bacteria	Firmicutes	Bacilli	Bacillales	Listeriaceae	Listeria	Listeria innocua; NCTC11288; X56152
			Bacteria	Firmicutes	Bacilli	Bacillales	Listeriaceae	Listeria	Listeria seeligeri (T); NCTC11856; X56148
			Bacteria	Firmicutes	Bacilli	Bacillales	Listeriaceae	Listeria	Listeria ivanovii; NCTC11846; X56151
o1473	o1473 16S-MeSpe	GCGACAGCAGTATGATGGC	Archaea	Euryarchaeota	Methanobacteria	Methanobacteriales	Methanobacteriaceae	Methanobacterium	Methanothermobacter thermoflexus; IDZ, VKM B-1963, DSM 7268; X99047
			Archaea	Euryarchaeota	Methanobacteria	Methanobacteriales	Methanobacteriaceae	Methanobacterium	Methanothermobacter defluvii; ADZ, VKM B-1962, DSM 7466; X99046
			Archaea	Euryarchaeota	Methanobacteria	Methanobacteriales	Methanobacteriaceae	Methanobacterium	Methanothermobacter thermophilus; M, VKM B-1786, DSM 6529; X99048
			Archaea	Euryarchaeota	Methanobacteria	Methanobacteriales	Methanobacteriaceae	Methanobrevibacter	Methanobrevibacter arboriphilus; SA; AB065294
			Archaea	Euryarchaeota	Methanobacteria	Methanobacteriales	Methanobacteriaceae	Methanobrevibacter	endosymbiont 'TS1' of Trimyema compressum; AB118591
			Archaea	Euryarchaeota	Methanobacteria	Methanobacteriales	Methanobacteriaceae	Methanobrevibacter	Methanobrevibacter curvatus (T); RFM-2; U62533
			Archaea	Euryarchaeota	Methanobacteria	Methanobacteriales	Methanobacteriaceae	Methanobrevibacter	Methanobrevibacter acididurans (T); ATM; AF242652
			Archaea	Euryarchaeota	Methanobacteria	Methanobacteriales	Methanobacteriaceae	Methanobacterium	Methanothermobacter thermautotrophicus; X68716
			Archaea	Euryarchaeota	Methanobacteria	Methanobacteriales	Methanobacteriaceae	Methanobacterium	Methanothermobacter thermautotrophicus (T); X68712
			Archaea	Euryarchaeota	Methanobacteria	Methanobacteriales	Methanobacteriaceae	Methanobacterium	Methanothermobacter thermautotrophicus; X68715
			Archaea	Euryarchaeota	Methanobacteria	Methanobacteriales	Methanobacteriaceae	Methanobacterium	Methanothermobacter thermautotrophicus; X68713
			Archaea	Euryarchaeota	Methanobacteria	Methanobacteriales	Methanobacteriaceae	Methanobacterium	Methanothermobacter thermautotrophicus; X68714
			Archaea	Euryarchaeota	Methanobacteria	Methanobacteriales	Methanobacteriaceae	Methanobacterium	Methanothermobacter thermautotrophicus; X68711
			Archaea	Euryarchaeota	Methanobacteria	Methanobacteriales	Methanobacteriaceae	Methanobacterium	Methanothermobacter thermautotrophicus; X68720
			Archaea	Euryarchaeota	Methanobacteria	Methanobacteriales	Methanobacteriaceae	Methanobrevibacter	Methanobrevibacter cuticularis (T); RFM-1; U41095
			Archaea	Euryarchaeota	Methanobacteria	Methanobacteriales	Methanobacteriaceae	Methanobacterium	Methanobacterium thermaggregans (T); OCM 141; AF095264
			Archaea	Euryarchaeota	Methanobacteria	Methanobacteriales	Methanobacteriaceae	Methanobacterium	Methanothermobacter thermautotrophicus; delta H; AY196660
			Archaea	Euryarchaeota	Methanobacteria	Methanobacteriales	Methanobacteriaceae	Methanobacterium	Methanothermobacter thermautotrophicus; GC-1; AY196661
			Archaea	Euryarchaeota	Methanobacteria	Methanobacteriales	Methanobacteriaceae	Methanobrevibacter	Methanobrevibacter arboriphilus; AZ; AY196663
			Archaea	Euryarchaeota	Methanobacteria	Methanobacteriales	Methanobacteriaceae	Methanobrevibacter	Methanobrevibacter arboriphilus; A2; AY196662
o1475	o1475 16S-Micbac	AAGGCAGATCTCTGGGCC	Bacteria	Actinobacteria	Actinobacteria	Actinomycetales	Microbacteriaceae	Microbacterium	Curtobacterium sp. VKM Ac-2061; AB042096
			Bacteria	Actinobacteria	Actinobacteria	Actinomycetales	Microbacteriaceae	Microbacterium	Microbacterium sp. VKM Ac-2050; AB042084
			Bacteria	Actinobacteria	Actinobacteria	Actinomycetales	Microbacteriaceae	Microbacterium	Microbacterium sp. VKM Ac-2011; AB042076
			Bacteria	Actinobacteria	Actinobacteria	Actinomycetales	Microbacteriaceae	Microbacterium	Microbacterium sp. VKM Ac-2047; AB042082

			Bacteria	Actinobacteria	Actinobacteria	Actinomycetales	Microbacteriaceae	Microbacterium	Microbacterium flavescens (T); DSM 20643; Y17232
			Bacteria	Actinobacteria	Actinobacteria	Actinomycetales	Microbacteriaceae	Microbacterium	Microbacterium imperiale (T); DSM 20530; X77442
			Bacteria	Actinobacteria	Actinobacteria	Actinomycetales	Microbacteriaceae	Microbacterium	Microbacterium sp. IFO16248; IFO 16248; AB023369
			Bacteria	Actinobacteria	Actinobacteria	Actinomycetales	Microbacteriaceae	Microbacterium	Microbacterium aurum; DSM 8600; Y17229
			Bacteria	Actinobacteria	Actinobacteria	Actinomycetales	Microbacteriaceae	Microbacterium	Microbacterium arabinogalactanolyticum; DSM 8611; Y17228
			Bacteria	Actinobacteria	Actinobacteria	Actinomycetales	Microbacteriaceae	Microbacterium	Microbacterium arborescens (T); DSM 20754; X77443
			Bacteria	Actinobacteria	Actinobacteria	Actinomycetales	Microbacteriaceae	Microbacterium	Microbacterium dextranolyticum; DSM 8607; Y17230
			Bacteria	Actinobacteria	Actinobacteria	Actinomycetales	Microbacteriaceae	Microbacterium	Microbacterium schleiferi; DSM 20489; Y17237
			Bacteria	Actinobacteria	Actinobacteria	Actinomycetales	Microbacteriaceae	Microbacterium	Microbacterium oxydans; DSM 20578; Y17227
			Bacteria	Actinobacteria	Actinobacteria	Actinomycetales	Microbacteriaceae	Microbacterium	Microbacterium resistens (T); DMMZ 1710; Y14699
			Bacteria	Actinobacteria	Actinobacteria	Actinomycetales	Microbacteriaceae	Microbacterium	Microbacterium trichotecenolyticum (T); IFO 15077; AB004722
			Bacteria	Actinobacteria	Actinobacteria	Actinomycetales	Microbacteriaceae	Microbacterium	Microbacterium laevaniformans; AB007416
			Bacteria	Actinobacteria	Actinobacteria	Actinomycetales	Microbacteriaceae	Microbacterium	Microbacterium dextranolyticum; AB007417
			Bacteria	Actinobacteria	Actinobacteria	Actinomycetales	Microbacteriaceae	Microbacterium	Microbacterium sp.; gh942; X95482
			Bacteria	Actinobacteria	Actinobacteria	Actinomycetales	Microbacteriaceae	Microbacterium	Microbacterium luteolum; DSM 20143; Y17235
			Bacteria	Actinobacteria	Actinobacteria	Actinomycetales	Microbacteriaceae	Microbacterium	Microbacterium terregens (T); DSM 20449; Y17239
o1476	o1476 16S-Miccoc	GGATCTGCGGTGGGTACG	Bacteria	Actinobacteria	Actinobacteria	Actinomycetales	Micrococcaceae	Arthrobacter	Arthrobacter sulfureus (T); DSM 20167; X83409
			Bacteria	Actinobacteria	Actinobacteria	Actinomycetales	Micrococcaceae	Arthrobacter	Arthrobacter pascens (T); DSM 20545; X80740
			Bacteria	Actinobacteria	Actinobacteria	Actinomycetales	Micrococcaceae	Arthrobacter	Arthrobacter oxydans (T); DSM 20119; X83408
			Bacteria	Actinobacteria	Actinobacteria	Actinomycetales	Micrococcaceae	Arthrobacter	Arthrobacter ramosus (T); DSM 20546; X80742
			Bacteria	Actinobacteria	Actinobacteria	Actinomycetales	Micrococcaceae	Arthrobacter	Arthrobacter globiformis (T); DSM 20124; X80736
			Bacteria	Actinobacteria	Actinobacteria	Actinomycetales	Micrococcaceae	Arthrobacter	Arthrobacter agilis (T); DSM 20550; X80748
			Bacteria	Actinobacteria	Actinobacteria	Actinomycetales	Micrococcaceae	Micrococcus	Micrococcus lylae (T); DSM 20315; X80750
			Bacteria	Actinobacteria	Actinobacteria	Actinomycetales	Micrococcaceae	Micrococcus	Micrococcus sp. 667-16; AJ296288
			Bacteria	Actinobacteria	Actinobacteria	Actinomycetales	Micrococcaceae	Micrococcus	Micrococcus luteus; IFO 16250; AB023371
			Bacteria	Actinobacteria	Actinobacteria	Actinomycetales	Micrococcaceae	Micrococcus	Micrococcus luteus; Ballarat; AJ409096
			Bacteria	Actinobacteria	Actinobacteria	Actinomycetales	Micrococcaceae	Micrococcus	Micrococcus sp. MN 8.1d.1c; AJ313024
			Bacteria	Actinobacteria	Actinobacteria	Actinomycetales	Micrococcaceae	Arthrobacter	Arthrobacter uratoxydans (T); DSM 20647; X83410
			Bacteria	Actinobacteria	Actinobacteria	Actinomycetales	Micrococcaceae	Arthrobacter	Arthrobacter polychromogenes (T); DSM 20136; X80741
			Bacteria	Actinobacteria	Actinobacteria	Actinomycetales	Micrococcaceae	Arthrobacter	Arthrobacter nicotianae (T); DSM 20123; X80739
			Bacteria	Actinobacteria	Actinobacteria	Actinomycetales	Micrococcaceae	Micrococcus	uncultured eubacterium; Y15857
			Bacteria	Actinobacteria	Actinobacteria	Actinomycetales	Micrococcaceae	Arthrobacter	Arthrobacter rhombi (T); F98.3HR69; Y15885
			Bacteria	Actinobacteria	Actinobacteria	Actinomycetales	Micrococcaceae	Micrococcus	Micrococcus luteus; D7; AJ409095
			Bacteria	Actinobacteria	Actinobacteria	Actinomycetales	Micrococcaceae	Micrococcus	Micrococcus sp.; 1F-15; X86608
			Bacteria	Actinobacteria	Actinobacteria	Actinomycetales	Micrococcaceae	Micrococcus	Micrococcus sp.; AC-47; X86609
			Bacteria	Actinobacteria	Actinobacteria	Actinomycetales	Micrococcaceae	Micrococcus	Micrococcus sp.; S2-07; X86612
o1477	o1477 16S-MicCor	ATGACACATGGTCGCATGAG	Bacteria	Actinobacteria	Actinobacteria	Actinomycetales	Streptosporangiaceae	Microbispora	Microbispora corallina (T); DF-32; AB018046
			Bacteria	Actinobacteria	Actinobacteria	Actinomycetales	Streptosporangiaceae	Microbispora	Microbispora sp. FXJ1.350; FJ938348
o1478	o1478 16S-MicGroup	ATATGAACCGTGGAGGCATC	Bacteria	Actinobacteria	Actinobacteria	Actinomycetales	Microbacteriaceae	Microbacterium	blackwater bioreactor bacterium BW7; AF394172
			Bacteria	Actinobacteria	Actinobacteria	Actinomycetales	Microbacteriaceae	Microbacterium	Microbacterium sp. IFO 15709; AB004728
			Bacteria	Actinobacteria	Actinobacteria	Actinomycetales	Microbacteriaceae	Microbacterium	Microbacterium sp. oral strain C24KA; AF287752
			Bacteria	Actinobacteria	Actinobacteria	Actinomycetales	Microbacteriaceae	Microbacterium	Microbacterium sp. EMB-C-1; DQ152000
			Bacteria	Actinobacteria	Actinobacteria	Actinomycetales	Microbacteriaceae	Microbacterium	uncultured actinobacterium; S15A-MN4; AJ534677
			Bacteria	Actinobacteria	Actinobacteria	Actinomycetales	Microbacteriaceae	Microbacterium	Microbacterium sp.; gh942; X95482
			Bacteria	Actinobacteria	Actinobacteria	Actinomycetales	Microbacteriaceae	Microbacterium	Microbacterium sp. S15-C; AM234163
			Bacteria	Actinobacteria	Actinobacteria	Actinomycetales	Microbacteriaceae	Microbacterium	Microbacterium sp. HINF001; AB426559
			Bacteria	Actinobacteria	Actinobacteria	Actinomycetales	Microbacteriaceae	Microbacterium	Microbacterium sp. 3188BRRJ; FJ200407
			Bacteria	Actinobacteria	Actinobacteria	Actinomycetales	Microbacteriaceae	Microbacterium	uncultured bacterium; TCE16; AF349766
o1479	o1479 16S-MicLut	TCTAATACCGGATAGGAGCG	Bacteria	Actinobacteria	Actinobacteria	Actinomycetales	Corynebacteriaceae	Corynebacterium	Corynebacterium tuberculostearicum (T); CIP107291; AJ438050
			Bacteria	Actinobacteria	Actinobacteria	Actinomycetales	Micrococcaceae	Micrococcus	Antarctic bacterium R-9183; AJ441006
			Bacteria	Actinobacteria	Actinobacteria	Actinomycetales	Micrococcaceae	Micrococcus	Micrococcus lylae (T); DSM 20315; X80750
			Bacteria	Actinobacteria	Actinobacteria	Actinomycetales	Micrococcaceae	Micrococcus	Micrococcus luteus; IFO 16250; AB023371
			Bacteria	Actinobacteria	Actinobacteria	Actinomycetales	Micrococcaceae	Micrococcus	Micrococcus luteus; Ballarat; AJ409096
			Bacteria	Actinobacteria	Actinobacteria	Actinomycetales	Micrococcaceae	Micrococcus	Variovorax sp. Amico6; AY512635
			Bacteria	Actinobacteria	Actinobacteria	Actinomycetales	Micrococcaceae	Micrococcus	Micrococcus sp. LMG-19421; AJ276811
			Bacteria	Actinobacteria	Actinobacteria	Actinomycetales	Corynebacteriaceae	Corynebacterium	uncultured bacterium I-109; AF018035
			Bacteria	Actinobacteria	Actinobacteria	Actinomycetales	Promicromonosporaceae	Promicromonospora	Promicromonospora citrea (T); DSM 43110T; X83808
			Bacteria	Actinobacteria	Actinobacteria	Actinomycetales	Micrococcaceae	Micrococcus	uncultured eubacterium; Y15857
			Bacteria	Actinobacteria	Actinobacteria	Actinomycetales	Micrococcaceae	Micrococcus	Micrococcus luteus; type strain: DSM 20030; AJ536198
			Bacteria	Actinobacteria	Actinobacteria	Actinomycetales	Micrococcaceae	Micrococcus	Micrococcus sp. Ellin149; AF408991
			Bacteria	Actinobacteria	Actinobacteria	Actinomycetales	Micrococcaceae	Micrococcus	Micrococcus luteus; D7; AJ409095
			Bacteria	Actinobacteria	Actinobacteria	Actinomycetales	Corynebacteriaceae	Corynebacterium	Corynebacterium camporealensis (T); CRS-51; CECT 4897; Y09569
			Bacteria	Actinobacteria	Actinobacteria	Actinomycetales	Corynebacteriaceae	Corynebacterium	Corynebacterium mycetoides (T); CIP 55.51T; X82066
			Bacteria	Actinobacteria	Actinobacteria	Actinomycetales	Micrococcaceae	Micrococcus	Micrococcus luteus; 118; AJ312751
			Bacteria	Actinobacteria	Actinobacteria	Actinomycetales	Micrococcaceae	Micrococcus	Micrococcus sp.; 1F-15; X86608
			Bacteria	Actinobacteria	Actinobacteria	Actinomycetales	Micrococcaceae	Micrococcus	rape rhizosphere bacterium cse077; AJ295375
			Bacteria	Actinobacteria	Actinobacteria	Actinomycetales	Micrococcaceae	Micrococcus	Micrococcus sp.; AC-47; X86609
			Bacteria	Actinobacteria	Actinobacteria	Actinomycetales	Micrococcaceae	Micrococcus	Micrococcus sp.; S2-07; X86612
o1480	o1480 16S-MogGroup	AATGACGGTACCTTAGGAGC	Bacteria	Firmicutes	Clostridia	Clostridiales	Incertae Sedis XIII	Anaerovorax	uncultured rumen bacterium; F24-D11; AB185611
			Bacteria	Firmicutes	Clostridia	Clostridiales	Incertae Sedis XIII	Anaerovorax	uncultured rumen bacterium; F24-F07; AB185628
			Bacteria	Firmicutes	Clostridia	Clostridiales	Incertae Sedis XIII	Anaerovorax	uncultured rumen bacterium 3C0d-7; AB034007
			Bacteria	Firmicutes	Clostridia	Clostridiales	Incertae Sedis XIII	Mogibacterium	Mogibacterium timidum; ATCC 33093; 93-3; Z36296
			Bacteria	Firmicutes	Clostridia	Clostridiales	Incertae Sedis XIII	Anaerovorax	uncultured rumen bacterium; L3B_A02; EU381659
			Bacteria	Firmicutes	Clostridia	Clostridiales	Incertae Sedis XIII	Mogibacterium	Mogibacterium vescum (T); ATCC 700697; AB021702
			Bacteria	Firmicutes	Clostridia	Clostridiales	Incertae Sedis XIII	Anaerovorax	uncultured rumen bacterium; GRC46; DQ673511
			Bacteria	Firmicutes	Clostridia	Clostridiales	Incertae Sedis XIII	Anaerovorax	uncultured bacterium; SJTU_B_11_91; EF402748
			Bacteria	Firmicutes	Clostridia	Clostridiales	Incertae Sedis XIII	Anaerovorax	uncultured rumen bacterium; L3A_D07; EU381498
			Bacteria	Firmicutes	Clostridia	Clostridiales	Incertae Sedis XIII	Mogibacterium	Mogibacterium neglectum (T); ATCC 700924 (=P9a-h); AB037875
			Bacteria	Firmicutes	Clostridia	Clostridiales	Incertae Sedis XIII	Anaerovorax	uncultured rumen bacterium; L3A_D01; EU381501
			Bacteria	Firmicutes	Clostridia	Clostridiales	Incertae Sedis XIII	Anaerovorax	uncultured rumen bacterium; L3B_D01; EU381533
			Bacteria	Firmicutes	Clostridia	Clostridiales	Incertae Sedis XIII	Anaerovorax	uncultured rumen bacterium; L3B_D07; EU381544
			Bacteria	Firmicutes	Clostridia	Clostridiales	Incertae Sedis XIII	Mogibacterium	Mogibacterium pumilum (T); ATCC 700696; AB021701
			Bacteria	Firmicutes	Clostridia	Clostridiales	Incertae Sedis XIII	Mogibacterium	Mogibacterium diversum (T); ATCC 700923 (=HM-7); AB037874
			Bacteria	Firmicutes	Clostridia	Clostridiales	Incertae Sedis XIII	Mogibacterium	Mogibacterium timidum (T); ATCC 33093; 93-3; U13042
			Bacteria	Firmicutes	Clostridia	Clostridiales	Incertae Sedis XIII	Anaerovorax	uncultured bacterium; p-3212-42A2; AF371683
			Bacteria	Firmicutes	Clostridia	Clostridiales	Incertae Sedis XIII	Anaerovorax	uncultured bacterium; RL181_aah42c06; DQ798414
			Bacteria	Firmicutes	Clostridia	Clostridiales	Incertae Sedis XIII	Anaerovorax	uncultured rumen bacterium 5C0d-2; AB034036
			Bacteria	Firmicutes	Clostridia	Clostridiales	Incertae Sedis XIII	Anaerovorax	uncultured bacterium; P615; AY916312

o1481	o1481 16S-Mollc	TAAGTACTCCGCCTGAGTAG	Bacteria	Firmicutes	Erysipelotrichi	Erysipelotrichales	Erysipelotrichaceae	Erysipelotrichaceae Incertae Sedis	Clostridium ramosum (T); DSM 1402; X73440
			Bacteria	Firmicutes	Erysipelotrichi	Erysipelotrichales	Erysipelotrichaceae	Erysipelotrichaceae Incertae Sedis	Clostridium ramosum; M91; AY699288
			Bacteria	Tenericutes	Mollicutes	Entomoplasmatales	Spiroplasmataceae	Spiroplasma	Spiroplasma melliferum (T); BC-3; AY325304
			Bacteria	Firmicutes	Erysipelotrichi	Erysipelotrichales	Erysipelotrichaceae	Erysipelotrichaceae Incertae Sedis	Clostridium spiroforme; DSM 1552; X73441
			Bacteria	Tenericutes	Mollicutes	Entomoplasmatales	Entomoplasmataceae	Entomoplasma	Entomoplasma lucivorax (T); ATCC 49196; AF547212
			Bacteria	Tenericutes	Mollicutes	Acholeplasmatales	Acholeplasmataceae	Acholeplasma	uncultured bacterium; EUB33-2; AY693836
			Bacteria	Firmicutes	Erysipelotrichi	Erysipelotrichales	Erysipelotrichaceae	Erysipelotrichaceae Incertae Sedis	Clostridium cocleatum (T); DSM 1551; Y18188
			Bacteria	Firmicutes	Erysipelotrichi	Erysipelotrichales	Erysipelotrichaceae	Coprobacillus	Coprobacillus cateniformis; JCM 10605; AB030220
			Bacteria	Firmicutes	Erysipelotrichi	Erysipelotrichales	Erysipelotrichaceae	Coprobacillus	Coprobacillus cateniformis (T); JCM 10604; AB030219
			Bacteria	Tenericutes	Mollicutes	Entomoplasmatales	Entomoplasmataceae	Entomoplasma	Mycoplasma capricolum subsp. capripneumoniae; 7/1a; AF009830
			Bacteria	Tenericutes	Mollicutes	Entomoplasmatales	Entomoplasmataceae	Entomoplasma	Mycoplasma capricolum subsp. capripneumoniae; 9231; AF009834
			Bacteria	Tenericutes	Mollicutes	Entomoplasmatales	Entomoplasmataceae	Entomoplasma	Mycoplasma capricolum subsp. capripneumoniae; GL102; AF009836
			Bacteria	Tenericutes	Mollicutes	Entomoplasmatales	Entomoplasmataceae	Entomoplasma	Mycoplasma capricolum subsp. capripneumoniae; 9081; AF009832
			Bacteria	Tenericutes	Mollicutes	Entomoplasmatales	Entomoplasmataceae	Entomoplasma	Mycoplasma capricolum subsp. capripneumoniae; 91106/C550/1; AF009833
			Bacteria	Tenericutes	Mollicutes	Entomoplasmatales	Entomoplasmataceae	Entomoplasma	Mycoplasma capricolum subsp. capripneumoniae; 8789; AF009831
			Bacteria	Tenericutes	Mollicutes	Entomoplasmatales	Entomoplasmataceae	Entomoplasma	Mycoplasma capricolum subsp. capripneumoniae; 95043; AF009835
			Bacteria	Tenericutes	Mollicutes	Entomoplasmatales	Entomoplasmataceae	Entomoplasma	Mycoplasma capricolum subsp. capripneumoniae; M79/93; AF009837
			Bacteria	Firmicutes	Erysipelotrichi	Erysipelotrichales	Erysipelotrichaceae	Coprobacillus	uncultured bacterium; PeM10; AJ576381
			Bacteria	Firmicutes	Erysipelotrichi	Erysipelotrichales	Erysipelotrichaceae	Erysipelotrichaceae Incertae Sedis	Clostridium sp. 14774; AJ315981
			Bacteria	Firmicutes	Erysipelotrichi	Erysipelotrichales	Erysipelotrichaceae	Erysipelotrichaceae Incertae Sedis	Clostridium spiroforme (T); ATCC 29900; X75908
o1482	o1482 16S-Morcea	GATCTGGAGGAATACCGATG	Bacteria	Proteobacteria	Gammaproteobacteria	Pseudomonadales	Moraxellaceae	Acinetobacter	unidentified bacterium; T58; Z93982
			Bacteria	Proteobacteria	Gammaproteobacteria	Pseudomonadales	Moraxellaceae	Acinetobacter	unidentified bacterium; T27; Z93987
			Bacteria	Proteobacteria	Gammaproteobacteria	Pseudomonadales	Moraxellaceae	Acinetobacter	unidentified bacterium; T17; Z93992
			Bacteria	Proteobacteria	Gammaproteobacteria	Pseudomonadales	Moraxellaceae	Acinetobacter	unidentified bacterium; T23; Z93988
			Bacteria	Proteobacteria	Gammaproteobacteria	Pseudomonadales	Moraxellaceae	Acinetobacter	Acinetobacter baumannii (T); DSM 30007; X81660
			Bacteria	Proteobacteria	Gammaproteobacteria	Pseudomonadales	Moraxellaceae	Acinetobacter	Acinetobacter junii (T); DSM 6964; X81664
			Bacteria	Proteobacteria	Gammaproteobacteria	Pseudomonadales	Moraxellaceae	Acinetobacter	uncultured gamma proteobacterium; S15A-MN113; AJ534674
			Bacteria	Proteobacteria	Gammaproteobacteria	Pseudomonadales	Moraxellaceae	Acinetobacter	Acinetobacter schindleri (T); LUH5832T; AJ278311
			Bacteria	Proteobacteria	Gammaproteobacteria	Pseudomonadales	Moraxellaceae	Moraxella	Moraxella canis (T); LMG 11194T (N7); AJ269511
			Bacteria	Proteobacteria	Gammaproteobacteria	Pseudomonadales	Moraxellaceae	Acinetobacter	Acinetobacter lwoffii (T); DSM 2403; X81665
			Bacteria	Proteobacteria	Gammaproteobacteria	Pseudomonadales	Moraxellaceae	Acinetobacter	Acinetobacter haemolyticus (T); DSM 6962; X81662
			Bacteria	Proteobacteria	Gammaproteobacteria	Pseudomonadales	Moraxellaceae	Acinetobacter	Acinetobacter radioresistens (T); DSM 6976; X81666
			Bacteria	Proteobacteria	Gammaproteobacteria	Pseudomonadales	Moraxellaceae	Acinetobacter	Acinetobacter johnsonii (T); DSM 6963; X81663
			Bacteria	Proteobacteria	Gammaproteobacteria	Pseudomonadales	Moraxellaceae	Acinetobacter	Acinetobacter ursingii (T); LUH 3792Type; AJ275038
			Bacteria	Proteobacteria	Gammaproteobacteria	Pseudomonadales	Moraxellaceae	Acinetobacter	uncultured eubacterium; 5-20; AJ222834
			Bacteria	Proteobacteria	Gammaproteobacteria	Pseudomonadales	Moraxellaceae	Acinetobacter	uncultured gamma proteobacterium; GuBH2-AD-51; AJ519659
			Bacteria	Proteobacteria	Gammaproteobacteria	Pseudomonadales	Moraxellaceae	Acinetobacter	uncultured gamma proteobacterium; GuBH2-AG-14; AJ519657
			Bacteria	Proteobacteria	Gammaproteobacteria	Pseudomonadales	Moraxellaceae	Acinetobacter	uncultured gamma proteobacterium; GuBH2-AG-72; AJ519661
			Bacteria	Proteobacteria	Gammaproteobacteria	Pseudomonadales	Moraxellaceae	Moraxella	Moraxella lincolnii; CCUG 9405T; AJ417490
			Bacteria	Proteobacteria	Gammaproteobacteria	Pseudomonadales	Moraxellaceae	Acinetobacter	uncultured gamma proteobacterium; Sh765B-TzT-52; AJ519628
o1483	o1483 16S-MycOra	ACTTCGTTGTGAAAGGAGCG	Bacteria	Tenericutes	Mollicutes	Mycoplasmatales	Mycoplasmataceae	Mycoplasma	Mycoplasma orale; AB353273
			Bacteria	Tenericutes	Mollicutes	Mycoplasmatales	Mycoplasmataceae	Mycoplasma	Mycoplasma orale; NC10112; CH 19299; ATCC 23714; AY796060
			Bacteria	Tenericutes	Mollicutes	Mycoplasmatales	Mycoplasmataceae	Mycoplasma	Mycoplasma timone; AY050170
			Bacteria	Tenericutes	Mollicutes	Mycoplasmatales	Mycoplasmataceae	Mycoplasma	Mycoplasma orale (T); M24659
o1484	o1484 16S-MyxGroup	AATCGCAGAAAACCGGTCTC	Bacteria	Proteobacteria	Deltaproteobacteria	Myxococcales	Myxococcaceae	Corallococcus	Corallococcus coralloides; DSM 52499; AJ811603
			Bacteria	Proteobacteria	Deltaproteobacteria	Myxococcales	Myxococcaceae	Corallococcus	Corallococcus coralloides (T); type strain: DSM 2259; AJ811588
			Bacteria	Proteobacteria	Deltaproteobacteria	Myxococcales	Myxococcaceae	Corallococcus	Corallococcus coralloides; DSM 52497; AJ811597
			Bacteria	Proteobacteria	Deltaproteobacteria	Myxococcales	Myxococcaceae	Corallococcus	Corallococcus coralloides; DSM 51558; AJ811602
			Bacteria	Proteobacteria	Deltaproteobacteria	Myxococcales	Myxococcaceae	Corallococcus	Corallococcus coralloides; DSM 52502; AJ811604
			Bacteria	Proteobacteria	Deltaproteobacteria	Myxococcales	Myxococcaceae	Corallococcus	Corallococcus coralloides; DSM 52503; AJ811605
			Bacteria	Proteobacteria	Deltaproteobacteria	Myxococcales	Myxococcaceae	Corallococcus	Corallococcus coralloides; DSM 51425; AJ811600
			Bacteria	Proteobacteria	Deltaproteobacteria	Myxococcales	Myxococcaceae	Corallococcus	Corallococcus coralloides; DSM 51359; AJ811589
			Bacteria	Proteobacteria	Deltaproteobacteria	Myxococcales	Myxococcaceae	Corallococcus	Corallococcus coralloides; DSM 51548; AJ811593
			Bacteria	Proteobacteria	Deltaproteobacteria	Myxococcales	Myxococcaceae	Corallococcus	Corallococcus coralloides; DSM 51547; AJ811592
			Bacteria	Proteobacteria	Deltaproteobacteria	Myxococcales	Myxococcaceae	Corallococcus	Corallococcus coralloides; DSM 51557; AJ811594
			Bacteria	Proteobacteria	Deltaproteobacteria	Myxococcales	Myxococcaceae	Corallococcus	Corallococcus coralloides; DSM 51651; AJ811595
			Bacteria	Proteobacteria	Deltaproteobacteria	Myxococcales	Myxococcaceae	Corallococcus	Corallococcus exiguus (T); type strain: DSM 14696; AJ811598
			Bacteria	Proteobacteria	Deltaproteobacteria	Myxococcales	Myxococcaceae	Corallococcus	Corallococcus coralloides; DSM 51307; AJ811599
			Bacteria	Proteobacteria	Deltaproteobacteria	Myxococcales	Myxococcaceae	Corallococcus	Corallococcus coralloides; DSM 51427; AJ811591
			Bacteria	Proteobacteria	Deltaproteobacteria	Myxococcales	Myxococcaceae	Corallococcus	Corallococcus coralloides; DSM 51379; AJ811590
			Bacteria	Proteobacteria	Deltaproteobacteria	Myxococcales	Myxococcaceae	Corallococcus	Corallococcus coralloides; DSM 51682; AJ811596
			Bacteria	Spirochaetes	Spirochaetes	Spirochaetales	Spirochaetaceae	Spirochaeta	uncultured spirochete; IE037; AY605134
			Bacteria	unclassified_Bacteria					uncultured bacterium; 44; AJ543758
			Bacteria	unclassified_Bacteria					uncultured bacterium; 79; AJ543760
o1485	o1485 16S-NeiElo	AAAGGAGCGGTTAATACCCG	Bacteria	Proteobacteria	Betaproteobacteria	Neisseriales	Neisseriaceae	Neisseria	uncultured bacterium; CLOSED_ROOT_A22; FJ983025
			Bacteria	Proteobacteria	Betaproteobacteria	Neisseriales	Neisseriaceae	Neisseria	uncultured bacterium; 390; EU531781
			Bacteria	Proteobacteria	Betaproteobacteria	Neisseriales	Neisseriaceae	Neisseria	uncultured Neisseria sp.; 102E06(oral); AM419995
			Bacteria	Proteobacteria	Betaproteobacteria	Neisseriales	Neisseriaceae	Neisseria	uncultured bacterium; SA01E06; FM874630
			Bacteria	Proteobacteria	Betaproteobacteria	Neisseriales	Neisseriaceae	Neisseria	uncultured bacterium; SA03D06; FM874782
			Bacteria	Proteobacteria	Betaproteobacteria	Neisseriales	Neisseriaceae	Neisseria	Neisseria elongata (T); L06171
			Bacteria	Proteobacteria	Betaproteobacteria	Neisseriales	Neisseriaceae	Neisseria	uncultured Neisseriaceae bacterium; 12B653; FJ976340
			Bacteria	Proteobacteria	Betaproteobacteria	Neisseriales	Neisseriaceae	Neisseria	uncultured bacterium; nbw1031g03c1; GQ033346
			Bacteria	Proteobacteria	Betaproteobacteria	Neisseriales	Neisseriaceae	Neisseria	uncultured bacterium; nbw150f08c1; GQ068444
			Bacteria	Proteobacteria	Betaproteobacteria	Neisseriales	Neisseriaceae	Neisseria	Neisseria elongata; I4; AJ239303
			Bacteria	Proteobacteria	Betaproteobacteria	Neisseriales	Neisseriaceae	Neisseria	Neisseria elongata; I1; AJ239297
			Bacteria	Proteobacteria	Betaproteobacteria	Neisseriales	Neisseriaceae	Neisseria	Neisseria elongata; I3; AJ239302
			Bacteria	Proteobacteria	Betaproteobacteria	Neisseriales	Neisseriaceae	Neisseria	Neisseria elongata subsp. glycolytica; AY167422
o1486	o1486 16S-NeiFla	ATATCGACAGCTGATGACGG	Bacteria	Proteobacteria	Betaproteobacteria	Neisseriales	Neisseriaceae	Neisseria	uncultured bacterium; P1D1-543; EF511878
			Bacteria	Proteobacteria	Betaproteobacteria	Neisseriales	Neisseriaceae	Neisseria	uncultured bacterium; BF0002B079; AM697129
			Bacteria	Proteobacteria	Betaproteobacteria	Neisseriales	Neisseriaceae	Neisseria	uncultured bacterium; P1D1-741; EF511911
			Bacteria	Proteobacteria	Betaproteobacteria	Neisseriales	Neisseriaceae	Neisseria	uncultured bacterium; P1D1-727; EF511897
			Bacteria	Proteobacteria	Betaproteobacteria	Neisseriales	Neisseriaceae	Neisseria	uncultured bacterium; P1D1-538; EF511922
			Bacteria	Proteobacteria	Betaproteobacteria	Neisseriales	Neisseriaceae	Neisseria	uncultured bacterium; P1D1-718; EF511935
			Bacteria	Proteobacteria	Betaproteobacteria	Neisseriales	Neisseriaceae	Neisseria	uncultured bacterium; P1D1-495; EF511925
			Bacteria	Proteobacteria	Betaproteobacteria	Neisseriales	Neisseriaceae	Neisseria	uncultured bacterium; AH55; AY963348
			Bacteria	Proteobacteria	Betaproteobacteria	Neisseriales	Neisseriaceae	Neisseria	uncultured beta proteobacterium; S15A-MN33; AJ534660
			Bacteria	Proteobacteria	Betaproteobacteria	Neisseriales	Neisseriaceae	Neisseria	uncultured bacterium; P1D1-570; EF511924
			Bacteria	Proteobacteria	Betaproteobacteria	Neisseriales	Neisseriaceae	Neisseria	uncultured bacterium; P1D1-729; EF511930

			Bacteria	Proteobacteria	Betaproteobacteria	Neisseriales	Neisseriaceae	Neisseria	uncultured bacterium; P1D1-500; EF511915
			Bacteria	Proteobacteria	Betaproteobacteria	Neisseriales	Neisseriaceae	Neisseria	uncultured bacterium; P1D1-534; EF511868
			Bacteria	Proteobacteria	Betaproteobacteria	Neisseriales	Neisseriaceae	Neisseria	Neisseria flavescens (T); L06168
			Bacteria	Proteobacteria	Betaproteobacteria	Neisseriales	Neisseriaceae	Neisseria	uncultured bacterium; P1D1-711; EF511861
			Bacteria	Proteobacteria	Betaproteobacteria	Neisseriales	Neisseriaceae	Neisseria	uncultured bacterium; P1D1-716; EF511903
			Bacteria	Proteobacteria	Betaproteobacteria	Neisseriales	Neisseriaceae	Neisseria	uncultured bacterium; P1D1-730; EF511923
			Bacteria	Proteobacteria	Betaproteobacteria	Neisseriales	Neisseriaceae	Neisseria	uncultured bacterium; P1D1-550; EF511936
			Bacteria	Proteobacteria	Betaproteobacteria	Neisseriales	Neisseriaceae	Neisseria	Neisseria subflava; NJ9703; AF479578
			Bacteria	Proteobacteria	Betaproteobacteria	Neisseriales	Neisseriaceae	Neisseria	Neisseria flavescens; LNP444; AJ239280
o1487	o1487 16S-NeiMen	TACCGCATACGTCTTGAGAG	Bacteria	Proteobacteria	Betaproteobacteria	Neisseriales	Neisseriaceae	Neisseria	Neisseria gonorrhoeae (T); NCTC 83785; X07714
			Bacteria	Proteobacteria	Betaproteobacteria	Neisseriales	Neisseriaceae	Neisseria	Neisseria meningitidis; NZ91/40; AY648029
			Bacteria	Proteobacteria	Betaproteobacteria	Neisseriales	Neisseriaceae	Neisseria	Neisseria meningitidis; NZ90/21; AY648028
			Bacteria	Proteobacteria	Betaproteobacteria	Neisseriales	Neisseriaceae	Neisseria	Neisseria polysaccharea; P8; AJ239298
			Bacteria	Proteobacteria	Betaproteobacteria	Neisseriales	Neisseriaceae	Neisseria	Neisseria meningitidis; S3446; AJ239312
			Bacteria	Proteobacteria	Betaproteobacteria	Neisseriales	Neisseriaceae	Neisseria	Neisseria lactamica; L17; AJ239313
			Bacteria	Proteobacteria	Betaproteobacteria	Neisseriales	Neisseriaceae	Neisseria	Neisseria meningitidis; B17; AJ239290
			Bacteria	Proteobacteria	Betaproteobacteria	Neisseriales	Neisseriaceae	Neisseria	Neisseria meningitidis; HF130; AJ239311
			Bacteria	Proteobacteria	Betaproteobacteria	Neisseriales	Neisseriaceae	Neisseria	Neisseria meningitidis; N94II; AJ239310
			Bacteria	Proteobacteria	Betaproteobacteria	Neisseriales	Neisseriaceae	Neisseria	Neisseria meningitidis; 44/76; AJ239307
			Bacteria	Proteobacteria	Betaproteobacteria	Neisseriales	Neisseriaceae	Neisseria	Neisseria meningitidis; HF46; AJ239309
			Bacteria	Proteobacteria	Betaproteobacteria	Neisseriales	Neisseriaceae	Neisseria	Neisseria meningitidis; M470; AJ239306
			Bacteria	Proteobacteria	Betaproteobacteria	Neisseriales	Neisseriaceae	Neisseria	Neisseria meningitidis; HF116; AJ239308
			Bacteria	Proteobacteria	Betaproteobacteria	Neisseriales	Neisseriaceae	Neisseria	Neisseria polysaccharea; NCTC11858; AJ239289
			Bacteria	Proteobacteria	Betaproteobacteria	Neisseriales	Neisseriaceae	Neisseria	Neisseria polysaccharea; P7; AJ239315
			Bacteria	Proteobacteria	Betaproteobacteria	Neisseriales	Neisseriaceae	Neisseria	Neisseria cinerea; LNP1646; AJ239287
			Bacteria	Proteobacteria	Betaproteobacteria	Neisseriales	Neisseriaceae	Neisseria	Neisseria cinerea; F1; AJ239299
			Bacteria	Proteobacteria	Betaproteobacteria	Neisseriales	Neisseriaceae	Neisseria	Neisseria cinerea; F3; AJ239300
			Bacteria	Proteobacteria	Betaproteobacteria	Neisseriales	Neisseriaceae	Neisseria	Neisseria gonorrhoeae; FA19; AJ239304
			Bacteria	Proteobacteria	Betaproteobacteria	Neisseriales	Neisseriaceae	Neisseria	Neisseria lactamica; L20; AJ239284
o1488	o1488 16S-NeiOral1	AAGAAAGCAGGGGACCTTTG	Bacteria	Proteobacteria	Betaproteobacteria	Neisseriales	Neisseriaceae	Neisseria	Neisseria sp. oral taxon 014 str. F0314; GQ131417
			Bacteria	Proteobacteria	Betaproteobacteria	Neisseriales	Neisseriaceae	Neisseria	Neisseria sp. oral clone AP015; AY005025
			Bacteria	Proteobacteria	Betaproteobacteria	Neisseriales	Neisseriaceae	Neisseria	uncultured bacterium; A_D_01_67; FJ470436
			Bacteria	Proteobacteria	Betaproteobacteria	Neisseriales	Neisseriaceae	Neisseria	uncultured Neisseria sp.; 201C11(oral); AM420020
			Bacteria	Proteobacteria	Betaproteobacteria	Neisseriales	Neisseriaceae	Neisseria	Neisseria sp. oral clone AP060; AY005026
			Bacteria	Proteobacteria	Betaproteobacteria	Neisseriales	Neisseriaceae	Neisseria	Neisseria sp. oral clone AP085; AY005023
			Bacteria	Proteobacteria	Betaproteobacteria	Neisseriales	Neisseriaceae	Neisseria	Neisseria sp. oral clone AP067; AY005024
			Bacteria	Proteobacteria	Betaproteobacteria	Neisseriales	Neisseriaceae	Neisseria	uncultured Neisseria sp.; 7BB269; FJ976264
			Bacteria	Proteobacteria	Betaproteobacteria	Neisseriales	Neisseriaceae	Neisseria	uncultured bacterium; nbw166e02c1; GQ074755
			Bacteria	Proteobacteria	Betaproteobacteria	Neisseriales	Neisseriaceae	Neisseria	uncultured bacterium; nbw1153e01c1; GQ076801
			Bacteria	Proteobacteria	Betaproteobacteria	Neisseriales	Neisseriaceae	Neisseria	uncultured bacterium; nbw1209e10c1; GQ077503
			Bacteria	Proteobacteria	Betaproteobacteria	Neisseriales	Neisseriaceae	Neisseria	uncultured Neisseria sp.; 3P-3-2-C19; EU705732
o1491	o1491 16S-OlsGroup	AGCCAACGCATTAAGTGTCC	Bacteria	Actinobacteria	Actinobacteria	Coriobacteriales	Coriobacteriaceae	Olsenella	uncultured bacterium; TuCw47; DQ071473
			Bacteria	Actinobacteria	Actinobacteria	Coriobacteriales	Coriobacteriaceae	Atopobium	uncultured bacterium; P2D15-510; EF510762
			Bacteria	Actinobacteria	Actinobacteria	Coriobacteriales	Coriobacteriaceae	Olsenella	uncultured rumen bacterium; BS34; AY244985
			Bacteria	Actinobacteria	Actinobacteria	Coriobacteriales	Coriobacteriaceae	Olsenella	Olsenella uli; ATCC 49627; AY005814
			Bacteria	Actinobacteria	Actinobacteria	Coriobacteriales	Coriobacteriaceae	Atopobium	uncultured bacterium; P2D15-495; EF510777
			Bacteria	Actinobacteria	Actinobacteria	Coriobacteriales	Coriobacteriaceae	Olsenella	Olsenella uli (T); ATCC49627; AF292373
			Bacteria	Actinobacteria	Actinobacteria	Coriobacteriales	Coriobacteriaceae	Olsenella	Olsenella profusa (T); D315A-29; AF292374
			Bacteria	Actinobacteria	Actinobacteria	Coriobacteriales	Coriobacteriaceae	Olsenella	uncultured Olsenella sp.; J21; DQ168838
			Bacteria	Actinobacteria	Actinobacteria	Coriobacteriales	Coriobacteriaceae	Olsenella	uncultured bacterium; FMFRc5_536; EU531941
			Bacteria	Actinobacteria	Actinobacteria	Coriobacteriales	Coriobacteriaceae	Atopobium	Atopobium vaginae; BVS059A5; AJ585206
			Bacteria	Actinobacteria	Actinobacteria	Coriobacteriales	Coriobacteriaceae	Olsenella	uncultured bacterium; hoa2_44d11; EU344415
			Bacteria	Actinobacteria	Actinobacteria	Coriobacteriales	Coriobacteriaceae	Atopobium	uncultured bacterium; SP2_a08_1; EU468911
			Bacteria	Actinobacteria	Actinobacteria	Coriobacteriales	Coriobacteriaceae	Collinsella	uncultured bacterium; RL181_aah42g05; DQ798448
			Bacteria	Actinobacteria	Actinobacteria	Coriobacteriales	Coriobacteriaceae	Collinsella	uncultured bacterium; RL181_aah42d08; DQ798425
			Bacteria	Actinobacteria	Actinobacteria	Coriobacteriales	Coriobacteriaceae	Collinsella	uncultured bacterium; RL240_aaj31d03; DQ794703
			Bacteria	Actinobacteria	Actinobacteria	Coriobacteriales	Coriobacteriaceae	Collinsella	uncultured bacterium; RL181_aan98d04; DQ798566
			Bacteria	Actinobacteria	Actinobacteria	Coriobacteriales	Coriobacteriaceae	Olsenella	uncultured bacterium; SP2_a05; EU469019
			Bacteria	Actinobacteria	Actinobacteria	Coriobacteriales	Coriobacteriaceae	Olsenella	Olsenella sp. N13-17; AY880046
			Bacteria	Actinobacteria	Actinobacteria	Coriobacteriales	Coriobacteriaceae	Olsenella	Olsenella sp. S13-10; AY880047
			Bacteria	Actinobacteria	Actinobacteria	Coriobacteriales	Coriobacteriaceae	Olsenella	uncultured Olsenella sp.; 1+7-15; AF426827
o1494	o1494 16S-PeaGroup1	GAGAAGAAAGCCCCGGCTA	Bacteria	Firmicutes	Bacilli	Bacillales	Paenibacillaceae	Paenibacillus	Paenibacillus alvei; DSM 29; AJ320491
			Bacteria	Firmicutes	Bacilli	Bacillales	Paenibacillaceae	Paenibacillus	Paenibacillus chitinolyticus (T); IFO15660; AB021183
			Bacteria	Firmicutes	Bacilli	Bacillales	Paenibacillaceae	Paenibacillus	Paenibacillus borealis (T); KK19; AJ011322
			Bacteria	Firmicutes	Bacilli	Bacillales	Paenibacillaceae	Paenibacillus	Paenibacillus borealis; HM31; AJ011327
			Bacteria	Firmicutes	Bacilli	Bacillales	Paenibacillaceae	Paenibacillus	Paenibacillus borealis; HM26; AJ011326
			Bacteria	Firmicutes	Bacilli	Bacillales	Paenibacillaceae	Paenibacillus	Paenibacillus sp.; vortex; AJ131119
			Bacteria	Firmicutes	Bacilli	Bacillales	Paenibacillaceae	Paenibacillus	Paenibacillus sp.; C-168; Y16129
			Bacteria	Firmicutes	Bacilli	Bacillales	Paenibacillaceae	Paenibacillus	Bacillus sp. USC14; AF346495
			Bacteria	Firmicutes	Bacilli	Bacillales	Paenibacillaceae	Paenibacillus	Paenibacillus peoriae; DSM 8320; AJ320494
			Bacteria	Firmicutes	Bacilli	Bacillales	Paenibacillaceae	Paenibacillus	Paenibacillus ehimensis; IFO15659; AB021184
			Bacteria	Firmicutes	Bacilli	Bacillales	Paenibacillaceae	Paenibacillus	Paenibacillus sp. B2; AJ011687
			Bacteria	Firmicutes	Bacilli	Bacillales	Paenibacillaceae	Paenibacillus	Paenibacillus thiaminolyticus; DSM 7262; AJ320490
			Bacteria	Firmicutes	Bacilli	Bacillales	Paenibacillaceae	Paenibacillus	Paenibacillus durus; type strain LMG 14658T; P3L-5; AJ251195
			Bacteria	Firmicutes	Bacilli	Bacillales	Paenibacillaceae	Paenibacillus	Paenibacillus durus (T); type strain ATCC 35681T; P3L-5; AJ251192
			Bacteria	Firmicutes	Bacilli	Bacillales	Paenibacillaceae	Paenibacillus	Bacillus sp. BE1; AB052966
			Bacteria	Firmicutes	Bacilli	Bacillales	Paenibacillaceae	Paenibacillus	Bacillus sp. FE1; AB052967
			Bacteria	Firmicutes	Bacilli	Bacillales	Paenibacillaceae	Paenibacillus	Paenibacillus polymyxa; CF43; AJ223989
			Bacteria	Firmicutes	Bacilli	Bacillales	Paenibacillaceae	Paenibacillus	Paenibacillus borealis; KN25; AJ011325
			Bacteria	Firmicutes	Bacilli	Bacillales	Paenibacillaceae	Paenibacillus	Paenibacillus borealis; KN24; AJ011324
			Bacteria	Firmicutes	Bacilli	Bacillales	Paenibacillaceae	Paenibacillus	Bacillus longisporus; LMG13275T; AJ223991
o1495	o1495 16S-PeMic	GTCCCTCGGGACAACTAT	Bacteria	Firmicutes	Clostridia	Clostridiales	Incertae Sedis XI	Parvimonas	Peptostreptococcus sp. oral clone FG014; AF385543
			Bacteria	Firmicutes	Clostridia	Clostridiales	Incertae Sedis XI	Parvimonas	uncultured bacterium; rRNA167; AY958940
			Bacteria	Firmicutes	Clostridia	Clostridiales	Incertae Sedis XI	Parvimonas	Parvimonas micra ATCC 33270; AY323523
			Bacteria	Firmicutes	Clostridia	Clostridiales	Incertae Sedis XI	Parvimonas	Peptostreptococcus sp. oral clone FJ023; AY349390
			Bacteria	Firmicutes	Clostridia	Clostridiales	Incertae Sedis XI	Parvimonas	Peptostreptococcus sp. oral clone BS044; AF385559
			Bacteria	Firmicutes	Clostridia	Clostridiales	Incertae Sedis XI	Parvimonas	uncultured bacterium; rRNA301; AY959074
			Bacteria	Firmicutes	Clostridia	Clostridiales	Incertae Sedis XI	Parvimonas	Parvimonas micra; gpac131; AM176531

			Bacteria	Firmicutes	Clostridia	Clostridiales	Incertae Sedis XI	Parvimonas	Parvimonas micra; gpac135; AM176533
			Bacteria	Firmicutes	Clostridia	Clostridiales	Incertae Sedis XI	Parvimonas	Parvimonas micra; gpac170; AM176538
			Bacteria	Firmicutes	Clostridia	Clostridiales	Incertae Sedis XI	Parvimonas	uncultured Peptostreptococcus sp.; MZH0805-43; EF120365
			Bacteria	Firmicutes	Clostridia	Clostridiales	Incertae Sedis XI	Parvimonas	Peptostreptococcus sp. 2002-69396; AY244772
			Bacteria	Firmicutes	Clostridia	Clostridiales	Incertae Sedis XI	Parvimonas	Parvimonas micra; gpac015; AM176518
			Bacteria	Firmicutes	Clostridia	Clostridiales	Incertae Sedis XI	Parvimonas	Parvimonas micra ATCC 33270 (T); AF542231
			Bacteria	Firmicutes	Clostridia	Clostridiales	Incertae Sedis XI	Parvimonas	Peptostreptococcus sp. CCUG 42997; AJ277208
			Bacteria	Firmicutes	Clostridia	Clostridiales	Incertae Sedis XI	Parvimonas	uncultured bacterium; LV17; AY976133
			Bacteria	Firmicutes	Clostridia	Clostridiales	Incertae Sedis XI	Parvimonas	uncultured bacterium; MG96; AY982204
			Bacteria	Firmicutes	Clostridia	Clostridiales	Incertae Sedis XI	Parvimonas	uncultured bacterium; LZ92; AY976482
			Bacteria	Firmicutes	Clostridia	Clostridiales	Incertae Sedis XI	Parvimonas	uncultured bacterium; MJ47; AY982344
			Bacteria	Firmicutes	Clostridia	Clostridiales	Incertae Sedis XI	Parvimonas	uncultured bacterium; MM10; AY982466
			Bacteria	Firmicutes	Clostridia	Clostridiales	Incertae Sedis XI	Parvimonas	Parvimonas micra; GIFU 7701; D14143
o1496	o1496 16S-PepGroup1	AAAACATAGGGGGCAAGCGT	Bacteria	Firmicutes	Clostridia	Clostridiales	Peptococcaceae	Peptococcus	uncultured bacterium; SJTU_D_11_95; EF401875
			Bacteria	Firmicutes	Clostridia	Clostridiales	Peptococcaceae	Peptococcus	Peptococcus sp. oral clone MCE10_265; AF481224
			Bacteria	Firmicutes	Clostridia	Clostridiales	Peptococcaceae	Peptococcus	uncultured bacterium; BF0002A054; AM696869
			Bacteria	Firmicutes	Clostridia	Clostridiales	Peptococcaceae	Peptococcus	Peptococcus-like sp. oral clone I070; AF287780
			Bacteria	Firmicutes	Clostridia	Clostridiales	Peptococcaceae	Peptococcus	uncultured bacterium; 8837-D0-C-5H; EU289049
			Bacteria	Firmicutes	Clostridia	Clostridiales	Peptococcaceae	Peptococcus	uncultured bacterium; p-2785-24E5; AF371709
			Bacteria	Firmicutes	Clostridia	Clostridiales	Peptococcaceae	Peptococcus	uncultured bacterium; AP10U.233; AM278590
			Bacteria	Firmicutes	Clostridia	Clostridiales	Peptococcaceae	Peptococcus	uncultured Peptostreptococcaceae bacterium; 8837-D0-C-2D; EU289041
			Bacteria	Firmicutes	Clostridia	Clostridiales	Peptococcaceae	Peptococcus	uncultured bacterium; HY1_h11_1; EU458459
			Bacteria	Firmicutes	Clostridia	Clostridiales	Peptococcaceae	Peptococcus	uncultured bacterium; CE3_aai05d05; EU459357
			Bacteria	Firmicutes	Clostridia	Clostridiales	Peptococcaceae	Peptococcus	uncultured bacterium; CE3_aai06b08; EU459373
			Bacteria	Firmicutes	Clostridia	Clostridiales	Peptococcaceae	Peptococcus	uncultured bacterium; RL183_aao03d06; DQ800782
			Bacteria	Firmicutes	Clostridia	Clostridiales	Peptococcaceae	Peptococcus	uncultured bacterium; RL183_aao04d03; DQ800855
			Bacteria	Firmicutes	Clostridia	Clostridiales	Peptococcaceae	Peptococcus	uncultured bacterium; CE3_aai05g10; EU459364
			Bacteria	Firmicutes	Clostridia	Clostridiales	Peptococcaceae	Peptococcus	uncultured eubacterium E1-K16; AJ289179
			Bacteria	Firmicutes	Clostridia	Clostridiales	Peptococcaceae	Peptococcus	uncultured Peptococcus sp.; 402D01(oral); AM420144
			Bacteria	Firmicutes	Clostridia	Clostridiales	Peptococcaceae	Peptococcus	uncultured bacterium; B583; DQ325836
			Bacteria	Firmicutes	Clostridia	Clostridiales	Peptococcaceae	Peptococcus	Peptococcus niger (T); X55797
			Bacteria	Firmicutes	Clostridia	Clostridiales	Peptococcaceae	Peptococcus	uncultured bacterium; V247; AY916277
			Bacteria	Firmicutes	Clostridia	Clostridiales	Clostridiaceae	Clostridium	uncultured bacterium; CE3_aai05a07; EU459349
o1497	o1497 16S-PepGroup2	AAGTTCTGTTGCAGGGGAAG	Bacteria	Firmicutes	Clostridia	Clostridiales	Peptostreptococcaceae	Peptostreptococcus	uncultured bacterium; 2F12; AY581823
			Bacteria	Firmicutes	Clostridia	Clostridiales	Peptostreptococcaceae	Peptostreptococcus	uncultured bacterium; 2B6; AY581819
			Bacteria	Firmicutes	Clostridia	Clostridiales	Peptostreptococcaceae	Peptostreptococcus	uncultured bacterium; SY1; DQ318876
			Bacteria	Firmicutes	Clostridia	Clostridiales	Peptostreptococcaceae	Peptostreptococcus	uncultured bacterium; 2F11; AY581820
			Bacteria	Firmicutes	Clostridia	Clostridiales	Peptostreptococcaceae	Peptostreptococcus	Peptostreptococcus sp. D1; AF044947
			Bacteria	Firmicutes	Clostridia	Clostridiales	Peptostreptococcaceae	Peptostreptococcus	Peptostreptococcus sp. oral clone CK035; AF287763
			Bacteria	Firmicutes	Clostridia	Clostridiales	Peptostreptococcaceae	Peptostreptococcus	uncultured Peptostreptococcus sp.; 101D05(oral); AM419962
			Bacteria	Firmicutes	Clostridia	Clostridiales	Peptostreptococcaceae	Peptostreptococcus	uncultured Peptostreptococcus sp.; EHFS1_S01a; EU071465
			Bacteria	Firmicutes	Clostridia	Clostridiales	Peptostreptococcaceae	Peptostreptococcus	Peptostreptococcus anaerobius; NCTC 11460; AY326462
			Bacteria	Firmicutes	Clostridia	Clostridiales	Peptostreptococcaceae	Peptostreptococcus	Peptostreptococcus stomatis (T); W2278; DQ160208
			Bacteria	Firmicutes	Clostridia	Clostridiales	Peptostreptococcaceae	Peptostreptococcus	uncultured bacterium; rRNA133; AY958906
			Bacteria	Firmicutes	Clostridia	Clostridiales	Peptostreptococcaceae	Peptostreptococcus	uncultured bacterium; rRNA173; AY958946
			Bacteria	Firmicutes	Clostridia	Clostridiales	Peptostreptococcaceae	Peptostreptococcus	uncultured bacterium; 1E3; AY581818
			Bacteria	Firmicutes	Clostridia	Clostridiales	Peptostreptococcaceae	Peptostreptococcus	Peptostreptococcus anaerobius; ATCC 27337; L04168
			Bacteria	Firmicutes	Clostridia	Clostridiales	Peptostreptococcaceae	Peptostreptococcus	Peptostreptococcus sp.; C; L04166
			Bacteria	Firmicutes	Clostridia	Clostridiales	Peptostreptococcaceae	Peptostreptococcus	Anaerococcus sp. BG1; EU011743
			Bacteria	Firmicutes	Clostridia	Clostridiales	Peptostreptococcaceae	Peptostreptococcus	uncultured bacterium; C120; AY916327
			Bacteria	Firmicutes	Clostridia	Clostridiales	Peptostreptococcaceae	Peptostreptococcus	Peptostreptococcus sp. C27; AY729956
			Bacteria	Firmicutes	Clostridia	Clostridiales	Peptostreptococcaceae	Peptostreptococcus	Anaerococcus sp. BG2; EU011744
			Bacteria	Firmicutes	Clostridia	Clostridiales	Peptostreptococcaceae	Peptostreptococcus	uncultured bacterium; HY1_b02_1; EU458348
o1498	o1498 16S-PepMic	GTCCCTCGGGACAACTAT	Bacteria	Firmicutes	Clostridia	Clostridiales	Incertae Sedis XI	Parvimonas	Peptostreptococcus sp. oral clone FG014; AF385543
			Bacteria	Firmicutes	Clostridia	Clostridiales	Incertae Sedis XI	Parvimonas	uncultured bacterium; rRNA167; AY958940
			Bacteria	Firmicutes	Clostridia	Clostridiales	Incertae Sedis XI	Parvimonas	Parvimonas micra ATCC 33270; AY323523
			Bacteria	Firmicutes	Clostridia	Clostridiales	Incertae Sedis XI	Parvimonas	Peptostreptococcus sp. oral clone FJ023; AY349390
			Bacteria	Firmicutes	Clostridia	Clostridiales	Incertae Sedis XI	Parvimonas	Peptostreptococcus sp. oral clone BS044; AF385559
			Bacteria	Firmicutes	Clostridia	Clostridiales	Incertae Sedis XI	Parvimonas	uncultured bacterium; rRNA301; AY959074
			Bacteria	Firmicutes	Clostridia	Clostridiales	Incertae Sedis XI	Parvimonas	Parvimonas micra; gpac131; AM176531
			Bacteria	Firmicutes	Clostridia	Clostridiales	Incertae Sedis XI	Parvimonas	Parvimonas micra; gpac135; AM176533
			Bacteria	Firmicutes	Clostridia	Clostridiales	Incertae Sedis XI	Parvimonas	Parvimonas micra; gpac170; AM176538
			Bacteria	Firmicutes	Clostridia	Clostridiales	Incertae Sedis XI	Parvimonas	uncultured Peptostreptococcus sp.; MZH0805-43; EF120365
			Bacteria	Firmicutes	Clostridia	Clostridiales	Incertae Sedis XI	Parvimonas	Peptostreptococcus sp. 2002-69396; AY244772
			Bacteria	Firmicutes	Clostridia	Clostridiales	Incertae Sedis XI	Parvimonas	Parvimonas micra; gpac015; AM176518
			Bacteria	Firmicutes	Clostridia	Clostridiales	Incertae Sedis XI	Parvimonas	Parvimonas micra ATCC 33270 (T); AF542231
			Bacteria	Firmicutes	Clostridia	Clostridiales	Incertae Sedis XI	Parvimonas	Peptostreptococcus sp. CCUG 42997; AJ277208
			Bacteria	Firmicutes	Clostridia	Clostridiales	Incertae Sedis XI	Parvimonas	uncultured bacterium; LV17; AY976133
			Bacteria	Firmicutes	Clostridia	Clostridiales	Incertae Sedis XI	Parvimonas	uncultured bacterium; MG96; AY982204
			Bacteria	Firmicutes	Clostridia	Clostridiales	Incertae Sedis XI	Parvimonas	uncultured bacterium; LZ92; AY976482
			Bacteria	Firmicutes	Clostridia	Clostridiales	Incertae Sedis XI	Parvimonas	uncultured bacterium; MJ47; AY982344
			Bacteria	Firmicutes	Clostridia	Clostridiales	Incertae Sedis XI	Parvimonas	uncultured bacterium; MM10; AY982466
			Bacteria	Firmicutes	Clostridia	Clostridiales	Incertae Sedis XI	Parvimonas	Parvimonas micra; GIFU 7701; D14143
o1499	o1499 16S-PepOral	CGAATCGTAAAGCTCTGTCC	Bacteria	Firmicutes	Clostridia	Clostridiales	Incertae Sedis XI	Peptoniphilus	swine manure bacterium 37-5; AY167967
			Bacteria	Firmicutes	Clostridia	Clostridiales	Incertae Sedis XI	Peptoniphilus	swine effluent bacterium CHNDP1; DQ337541
			Bacteria	Firmicutes	Clostridia	Clostridiales	Incertae Sedis XI	Parvimonas	Peptostreptococcus sp. MDA2346-2; AY286545
			Bacteria	Firmicutes	Clostridia	Clostridiales	Incertae Sedis XI	Parvimonas	Peptostreptococcus sp. oral clone HE064; DQ087188
			Bacteria	Firmicutes	Clostridia	Clostridiales	Incertae Sedis XI	Parvimonas	Peptostreptococcus sp. oral clone FG014; AF385543
			Bacteria	Firmicutes	Clostridia	Clostridiales	Incertae Sedis XI	Parvimonas	Peptostreptococcus sp. P4P_31 P3; AY207059
			Bacteria	Firmicutes	Clostridia	Clostridiales	Incertae Sedis XI	Parvimonas	Peptostreptococcus sp. oral clone AJ062; AF287767
			Bacteria	Firmicutes	Clostridia	Clostridiales	Incertae Sedis XI	Parvimonas	Parvimonas micra ATCC 33270; AY323523
			Bacteria	Firmicutes	Clostridia	Clostridiales	Incertae Sedis XI	Parvimonas	Peptostreptococcus sp. oral clone FJ023; AY349390
			Bacteria	Firmicutes	Clostridia	Clostridiales	Incertae Sedis XI	Parvimonas	Peptostreptococcus sp. oral clone BS044; AF385559
			Bacteria	Firmicutes	Clostridia	Clostridiales	Incertae Sedis XI	Parvimonas	Peptostreptococcus sp. 2002-69396; AY244772
			Bacteria	Firmicutes	Clostridia	Clostridiales	Incertae Sedis XI	Parvimonas	unidentified eubacterium clone 8.29; U43700
			Bacteria	Firmicutes	Clostridia	Clostridiales	Incertae Sedis XI	Parvimonas	Parvimonas micra ATCC 33270 (T); AF542231
			Bacteria	Firmicutes	Clostridia	Clostridiales	Incertae Sedis XI	Parvimonas	Peptostreptococcus sp. CCUG 42997; AJ277208
			Bacteria	Firmicutes	Clostridia	Clostridiales	Incertae Sedis XI	Parvimonas	uncultured bacterium; LV17; AY976133

			Bacteria	Firmicutes	Clostridia	Clostridiales	Incertae Sedis XI	Parvimonas	uncultured bacterium; MG96; AY982204
			Bacteria	Firmicutes	Clostridia	Clostridiales	Incertae Sedis XI	Parvimonas	uncultured bacterium; LZ92; AY976482
			Bacteria	Firmicutes	Clostridia	Clostridiales	Incertae Sedis XI	Parvimonas	uncultured bacterium; MJ47; AY982344
			Bacteria	Firmicutes	Clostridia	Clostridiales	Incertae Sedis XI	Parvimonas	uncultured bacterium; MM10; AY982466
			Bacteria	Firmicutes	Clostridia	Clostridiales	Incertae Sedis XI	Parvimonas	Parvimonas micra; GIFU 7701; D14143
o1500	o1500 16S-PestGroup1	TAATGACGGTACCCTGTGAG	Bacteria	Firmicutes	Clostridia	Clostridiales	Peptostreptococcaceae	Peptostreptococcus	uncultured bacterium; 2F12; AY581823
			Bacteria	Firmicutes	Clostridia	Clostridiales	Peptostreptococcaceae	Peptostreptococcus	uncultured bacterium; 2B6; AY581819
			Bacteria	Firmicutes	Clostridia	Clostridiales	Peptostreptococcaceae	Peptostreptococcus	uncultured bacterium; SY1; DQ318876
			Bacteria	Firmicutes	Clostridia	Clostridiales	Peptostreptococcaceae	Peptostreptococcus	uncultured bacterium; 2F11; AY581820
			Bacteria	Firmicutes	Clostridia	Clostridiales	Peptostreptococcaceae	Peptostreptococcus	Peptostreptococcus sp. D1; AF044947
			Bacteria	Firmicutes	Clostridia	Clostridiales	Peptostreptococcaceae	Peptostreptococcus	Peptostreptococcus sp. oral clone CK035; AF287763
			Bacteria	Firmicutes	Clostridia	Clostridiales	Peptostreptococcaceae	Peptostreptococcus	swine manure bacterium RT-18B; AY167956
			Bacteria	Firmicutes	Clostridia	Clostridiales	Peptostreptococcaceae	Peptostreptococcus	swine manure bacterium 37-1; AY167963
			Bacteria	Firmicutes	Clostridia	Clostridiales	Peptostreptococcaceae	Peptostreptococcus	swine manure bacterium RT-8A; AY167949
			Bacteria	Firmicutes	Clostridia	Clostridiales	Peptostreptococcaceae	Peptostreptococcus	swine manure bacterium RT-10B; AY167952
			Bacteria	Firmicutes	Clostridia	Clostridiales	Peptostreptococcaceae	Peptostreptococcus	swine manure bacterium RT-19A; AY167957
			Bacteria	Firmicutes	Clostridia	Clostridiales	Peptostreptococcaceae	Peptostreptococcus	Peptostreptococcus anaerobius; NCTC 11460; AY326462
			Bacteria	Firmicutes	Clostridia	Clostridiales	Peptostreptococcaceae	Peptostreptococcus	Peptostreptococcus stomatis (T); W2278; DQ160208
			Bacteria	Firmicutes	Clostridia	Clostridiales	Peptostreptococcaceae	Peptostreptococcus	uncultured bacterium; rRNA133; AY958906
			Bacteria	Firmicutes	Clostridia	Clostridiales	Peptostreptococcaceae	Peptostreptococcus	uncultured bacterium; rRNA173; AY958946
			Bacteria	Firmicutes	Clostridia	Clostridiales	Peptostreptococcaceae	Peptostreptococcus	uncultured bacterium; 1E3; AY581818
			Bacteria	Firmicutes	Clostridia	Clostridiales	Peptostreptococcaceae	Peptostreptococcus	Peptostreptococcus anaerobius; ATCC 27337; L04168
			Bacteria	Firmicutes	Clostridia	Clostridiales	Peptostreptococcaceae	Peptostreptococcus	Peptostreptococcus sp.; C; L04166
			Bacteria	Firmicutes	Clostridia	Clostridiales	Peptostreptococcaceae	Peptostreptococcus	uncultured bacterium; C120; AY916327
			Bacteria	Firmicutes	Clostridia	Clostridiales	Peptostreptococcaceae	Peptostreptococcus	Peptostreptococcus sp. C27; AY729956
o1501	o1501 16S-Plmyc	TTGACGGGGGCTCACACAA	Bacteria	Planctomycetes	Planctomycetacia	Planctomycetales	Planctomycetaceae	Planctomyces	Planctomyces sp.; Schlesner 130; X81952
			Bacteria	Planctomycetes	Planctomycetacia	Planctomycetales	Planctomycetaceae	Planctomyces	Planctomyces maris (T); DSM 8797T; AJ231184
			Bacteria	Planctomycetes	Planctomycetacia	Planctomycetales	Planctomycetaceae	Blastopirellula	Blastopirellula marina (T); IFAM 1313; X62912
			Bacteria	Planctomycetes	Planctomycetacia	Planctomycetales	Planctomycetaceae	Planctomyces	Planctomyces limnophilus (T); IFAM 1008; X62911
			Bacteria	Planctomycetes	Planctomycetacia	Planctomycetales	Planctomycetaceae	Gemmata	Gemmata obscuriglobus; UQM 2246; mp18 HH1, mp19 HH2; X56305
			Bacteria	Planctomycetes	Planctomycetacia	Planctomycetales	Planctomycetaceae	Planctomyces	Planctomyces sp.; Schlesner 642; X81950
			Bacteria	Planctomycetes	Planctomycetacia	Planctomycetales	Planctomycetaceae	Planctomyces	Planctomyces sp. 599; AJ231189
			Bacteria	Planctomycetes	Planctomycetacia	Planctomycetales	Planctomycetaceae	Gemmata	Gemmata obscuriglobus; Schlesner 633; X81957
			Bacteria	Planctomycetes	Planctomycetacia	Planctomycetales	Planctomycetaceae	Planctomyces	Planctomyces sp.; Schlesner 668; X81956
			Bacteria	Planctomycetes	Planctomycetacia	Planctomycetales	Planctomycetaceae	Planctomyces	Planctomyces sp.; Schlesner 658; X81954
			Bacteria	Planctomycetes	Planctomycetacia	Planctomycetales	Planctomycetaceae	Planctomyces	Planctomyces sp.; Schlesner 269; X81953
			Bacteria	Planctomycetes	Planctomycetacia	Planctomycetales	Planctomycetaceae	Planctomyces	Planctomyces sp.; Schlesner 638; X81951
			Bacteria	Planctomycetes	Planctomycetacia	Planctomycetales	Planctomycetaceae	Planctomyces	Planctomyces sp. 248; AJ231186
			Bacteria	Planctomycetes	Planctomycetacia	Planctomycetales	Planctomycetaceae	Planctomyces	Planctomyces brasiliensis (T); DSM 5305T; AJ231190
			Bacteria	Planctomycetes	Planctomycetacia	Planctomycetales	Planctomycetaceae	Isosphaera	Isosphaera pallida (T); DSM 9630T; AJ231195
			Bacteria	Planctomycetes	Planctomycetacia	Planctomycetales	Planctomycetaceae	Gemmata	Gemmata obscuriglobus (T); DSM 5831T; AJ231191
			Bacteria	Planctomycetes	Planctomycetacia	Planctomycetales	Planctomycetaceae	Planctomyces	Planctomyces sp.; Schlesner 664; X81955
			Bacteria	Planctomycetes	Planctomycetacia	Planctomycetales	Planctomycetaceae	Gemmata	Gemmata obscuriglobus; UQM 2246; X54522
			Bacteria	Planctomycetes	Planctomycetacia	Planctomycetales	Planctomycetaceae	Planctomyces	agricultural soil bacterium SC-I-11; AJ252614
			Bacteria	Planctomycetes	Planctomycetacia	Planctomycetales	Planctomycetaceae	Planctomyces	Planctomyces brasiliensis; DSM 5305; X81949
o1502	o1502 16S-PorGroup1	CGATGATAACTGGGCGTATG	Bacteria	Bacteroidetes	Bacteroidetes	Bacteroidales	Porphyromonadaceae	Porphyromonas	uncultured Porphyromonas sp.; DS033; AF366271
			Bacteria	Bacteroidetes	Bacteroidetes	Bacteroidales	Porphyromonadaceae	Porphyromonas	Porphyromonas sp. oral clone EP003; AY008313
			Bacteria	Bacteroidetes	Bacteroidetes	Bacteroidales	Porphyromonadaceae	Porphyromonas	Porphyromonas sp. oral clone DP023; AF385516
			Bacteria	Bacteroidetes	Bacteroidetes	Bacteroidales	Porphyromonadaceae	Porphyromonas	Porphyromonas sp. oral clone HF001; DQ087194
			Bacteria	Bacteroidetes	Bacteroidetes	Bacteroidales	Porphyromonadaceae	Porphyromonas	Porphyromonas sp. oral clone BS045; AF385560
			Bacteria	Bacteroidetes	Bacteroidetes	Bacteroidales	Porphyromonadaceae	Porphyromonas	uncultured bacterium; P1D1-721; EF511910
			Bacteria	Bacteroidetes	Bacteroidetes	Bacteroidales	Porphyromonadaceae	Porphyromonas	Porphyromonas sp. oral clone BS077; AF385561
			Bacteria	Bacteroidetes	Bacteroidetes	Bacteroidales	Porphyromonadaceae	Porphyromonas	uncultured bacterium; P4D1-570; EF509718
			Bacteria	Bacteroidetes	Bacteroidetes	Bacteroidales	Porphyromonadaceae	Porphyromonas	Porphyromonas sp. oral clone BR037; AF385555
			Bacteria	Bacteroidetes	Bacteroidetes	Bacteroidales	Porphyromonadaceae	Porphyromonas	Porphyromonas sp. oral clone CW034; AY008310
			Bacteria	Bacteroidetes	Bacteroidetes	Bacteroidales	Porphyromonadaceae	Porphyromonas	uncultured bacterium; P3D1-425; EF509229
			Bacteria	Bacteroidetes	Bacteroidetes	Bacteroidales	Porphyromonadaceae	Porphyromonas	Porphyromonas sp. oral clone AW032; AF393476
			Bacteria	Bacteroidetes	Bacteroidetes	Bacteroidales	Porphyromonadaceae	Porphyromonas	uncultured bacterium; P1D1-760; EF511862
			Bacteria	Bacteroidetes	Bacteroidetes	Bacteroidales	Porphyromonadaceae	Porphyromonas	uncultured bacterium; P1D1-698; EF511866
			Bacteria	Bacteroidetes	Bacteroidetes	Bacteroidales	Porphyromonadaceae	Porphyromonas	uncultured bacterium; P1D1-674; EF511874
			Bacteria	Bacteroidetes	Bacteroidetes	Bacteroidales	Porphyromonadaceae	Porphyromonas	Porphyromonas macacae (T); L16494
			Bacteria	Bacteroidetes	Bacteroidetes	Bacteroidales	Porphyromonadaceae	Porphyromonas	Porphyromonas catoniae (T); ATCC 51270; X82823
			Bacteria	Bacteroidetes	Bacteroidetes	Bacteroidales	Porphyromonadaceae	Porphyromonas	uncultured Bacteroidetes bacterium; S15B-MN34; AJ583190
			Bacteria	Bacteroidetes	Bacteroidetes	Bacteroidales	Porphyromonadaceae	Porphyromonas	uncultured Bacteroidetes bacterium; WB1B005; AY862036
			Bacteria	Bacteroidetes	Bacteroidetes	Bacteroidales	Porphyromonadaceae	Porphyromonas	Porphyromonas macacae; L26103
o1503	o1503 16S-PorGroup2	AAGATAGGCATGCGTCCCAT	Bacteria	Bacteroidetes	Bacteroidetes	Bacteroidales	Porphyromonadaceae	Porphyromonas	Porphyromonas gingivalis; W83; AB035456
			Bacteria	Bacteroidetes	Bacteroidetes	Bacteroidales	Porphyromonadaceae	Porphyromonas	Porphyromonas gingivalis; SUNY1021; AB035457
			Bacteria	Bacteroidetes	Bacteroidetes	Bacteroidales	Porphyromonadaceae	Porphyromonas	Porphyromonas gingivalis; FDC 381; AB035455
			Bacteria	Bacteroidetes	Bacteroidetes	Bacteroidales	Porphyromonadaceae	Porphyromonas	Porphyromonas gingivalis; ATCC33277; AB035459
			Bacteria	Bacteroidetes	Bacteroidetes	Bacteroidales	Porphyromonadaceae	Porphyromonas	Porphyromonas gulae (T); Loup-1; ATCC 51700; AF208290
			Bacteria	Bacteroidetes	Bacteroidetes	Bacteroidales	Porphyromonadaceae	Porphyromonas	Porphyromonas gingivalis; ATCC33277; AF285870
			Bacteria	Bacteroidetes	Bacteroidetes	Bacteroidales	Porphyromonadaceae	Porphyromonas	Porphyromonas gulae; chat 3.1; AF285871
			Bacteria	Bacteroidetes	Bacteroidetes	Bacteroidales	Porphyromonadaceae	Porphyromonas	Porphyromonas gulae; B 243; AF285874
			Bacteria	Bacteroidetes	Bacteroidetes	Bacteroidales	Porphyromonadaceae	Porphyromonas	Porphyromonas gulae; chien 4.2; AF285873
			Bacteria	Bacteroidetes	Bacteroidetes	Bacteroidales	Porphyromonadaceae	Porphyromonas	Porphyromonas gulae; ours 3.1; AF285872
			Bacteria	Bacteroidetes	Bacteroidetes	Bacteroidales	Porphyromonadaceae	Porphyromonas	Porphyromonas gingivalis; cerco 1.5.2; AF287987
			Bacteria	Bacteroidetes	Bacteroidetes	Bacteroidales	Porphyromonadaceae	Porphyromonas	Porphyromonas gulae; Manga 1.41; AF285869
			Bacteria	Bacteroidetes	Bacteroidetes	Bacteroidales	Porphyromonadaceae	Porphyromonas	Porphyromonas gulae; coyote 1.5.1; AF287986
			Bacteria	Bacteroidetes	Bacteroidetes	Bacteroidales	Porphyromonadaceae	Porphyromonas	Porphyromonas gingivalis; DSM 20709; X73964
			Bacteria	Bacteroidetes	Bacteroidetes	Bacteroidales	Porphyromonadaceae	Porphyromonas	Porphyromonas gingivalis; 2-PGI; AF414811
			Bacteria	Bacteroidetes	Bacteroidetes	Bacteroidales	Porphyromonadaceae	Porphyromonas	Porphyromonas gingivalis; 1-PGI; AF414810
			Bacteria	Bacteroidetes	Bacteroidetes	Bacteroidales	Porphyromonadaceae	Porphyromonas	Porphyromonas gingivalis; 40-PGI; AF414813
			Bacteria	Bacteroidetes	Bacteroidetes	Bacteroidales	Porphyromonadaceae	Porphyromonas	Porphyromonas gingivalis; ATCC 33277; AF414809
			Bacteria	Bacteroidetes	Bacteroidetes	Bacteroidales	Porphyromonadaceae	Porphyromonas	Porphyromonas gingivalis; 42-PGI; AF414814
			Bacteria	Bacteroidetes	Bacteroidetes	Bacteroidales	Porphyromonadaceae	Porphyromonas	Porphyromonas gingivalis; 3-PGI; AF414812
o1504	o1504 16S-PorGroup3	ACTGGGCGTATGCGATATAC	Bacteria	Bacteroidetes	Bacteroidetes	Bacteroidales	Porphyromonadaceae	Porphyromonas	uncultured Porphyromonas sp.; DS033; AF366271
			Bacteria	Bacteroidetes	Bacteroidetes	Bacteroidales	Porphyromonadaceae	Porphyromonas	Porphyromonas sp. oral clone EP003; AY008313
			Bacteria	Bacteroidetes	Bacteroidetes	Bacteroidales	Porphyromonadaceae	Porphyromonas	Porphyromonas sp. oral clone DP023; AF385516

			Bacteria	Bacteroidetes	Bacteroidetes	Bacteroidales	Porphyromonadaceae	Porphyromonas	Porphyromonas sp. oral clone HF001; DQ087194
			Bacteria	Bacteroidetes	Bacteroidetes	Bacteroidales	Porphyromonadaceae	Porphyromonas	Porphyromonas sp. oral clone BS045; AF385560
			Bacteria	Bacteroidetes	Bacteroidetes	Bacteroidales	Porphyromonadaceae	Porphyromonas	uncultured bacterium; P1D1-721; EF511910
			Bacteria	Bacteroidetes	Bacteroidetes	Bacteroidales	Porphyromonadaceae	Porphyromonas	Porphyromonas sp. oral clone BS077; AF385561
			Bacteria	Bacteroidetes	Bacteroidetes	Bacteroidales	Porphyromonadaceae	Porphyromonas	uncultured bacterium; P4D1-570; EF509718
			Bacteria	Bacteroidetes	Bacteroidetes	Bacteroidales	Porphyromonadaceae	Porphyromonas	Porphyromonas sp. oral clone BR037; AF385555
			Bacteria	Bacteroidetes	Bacteroidetes	Bacteroidales	Porphyromonadaceae	Porphyromonas	Porphyromonas sp. oral clone CW034; AY008310
			Bacteria	Bacteroidetes	Bacteroidetes	Bacteroidales	Porphyromonadaceae	Porphyromonas	uncultured bacterium; P3D1-425; EF509229
			Bacteria	Bacteroidetes	Bacteroidetes	Bacteroidales	Porphyromonadaceae	Porphyromonas	Porphyromonas sp. oral clone AW032; AF393476
			Bacteria	Bacteroidetes	Bacteroidetes	Bacteroidales	Porphyromonadaceae	Porphyromonas	uncultured bacterium; P1D1-760; EF511862
			Bacteria	Bacteroidetes	Bacteroidetes	Bacteroidales	Porphyromonadaceae	Porphyromonas	uncultured bacterium; P1D1-698; EF511866
			Bacteria	Bacteroidetes	Bacteroidetes	Bacteroidales	Porphyromonadaceae	Porphyromonas	uncultured bacterium; P1D1-674; EF511874
			Bacteria	Bacteroidetes	Bacteroidetes	Bacteroidales	Porphyromonadaceae	Porphyromonas	Porphyromonas macacae (T); L16494
			Bacteria	Bacteroidetes	Bacteroidetes	Bacteroidales	Porphyromonadaceae	Porphyromonas	Porphyromonas catoniae (T); ATCC 51270; X82823
			Bacteria	Bacteroidetes	Bacteroidetes	Bacteroidales	Porphyromonadaceae	Porphyromonas	uncultured Bacteroidetes bacterium; S15B-MN34; AJ583190
			Bacteria	Bacteroidetes	Bacteroidetes	Bacteroidales	Porphyromonadaceae	Porphyromonas	uncultured Bacteroidetes bacterium; WB1B005; AY862036
			Bacteria	Bacteroidetes	Bacteroidetes	Bacteroidales	Porphyromonadaceae	Porphyromonas	Porphyromonas macacae; L26103
o1506	o1506 16S-PorGroup5	AAGACGGACTAATACCGCGT	Bacteria	Bacteroidetes	Bacteroidetes	Bacteroidales	Porphyromonadaceae	Porphyromonas	uncultured Porphyromonas sp.; DS033; AF366271
			Bacteria	Bacteroidetes	Bacteroidetes	Bacteroidales	Porphyromonadaceae	Porphyromonas	Porphyromonas sp. oral clone EP003; AY008313
			Bacteria	Bacteroidetes	Bacteroidetes	Bacteroidales	Porphyromonadaceae	Porphyromonas	Porphyromonas sp. oral clone DP023; AF385516
			Bacteria	Bacteroidetes	Bacteroidetes	Bacteroidales	Porphyromonadaceae	Porphyromonas	uncultured Porphyromonas sp.; 302E06(oral); AM420091
			Bacteria	Bacteroidetes	Bacteroidetes	Bacteroidales	Porphyromonadaceae	Porphyromonas	uncultured Porphyromonas sp.; 101H12(oral); AM419980
			Bacteria	Bacteroidetes	Bacteroidetes	Bacteroidales	Porphyromonadaceae	Porphyromonas	uncultured Porphyromonas sp.; 201C12(oral); AM420021
			Bacteria	Bacteroidetes	Bacteroidetes	Bacteroidales	Porphyromonadaceae	Porphyromonas	Porphyromonas sp. oral clone BS045; AF385560
			Bacteria	Bacteroidetes	Bacteroidetes	Bacteroidales	Porphyromonadaceae	Porphyromonas	uncultured bacterium; P1D1-721; EF511910
			Bacteria	Bacteroidetes	Bacteroidetes	Bacteroidales	Porphyromonadaceae	Porphyromonas	Porphyromonas sp. oral clone BS077; AF385561
			Bacteria	Bacteroidetes	Bacteroidetes	Bacteroidales	Porphyromonadaceae	Porphyromonas	uncultured bacterium; P4D1-570; EF509718
			Bacteria	Bacteroidetes	Bacteroidetes	Bacteroidales	Porphyromonadaceae	Porphyromonas	Porphyromonas sp. oral clone BR037; AF385555
			Bacteria	Bacteroidetes	Bacteroidetes	Bacteroidales	Porphyromonadaceae	Porphyromonas	Porphyromonas sp. oral clone CW034; AY008310
			Bacteria	Bacteroidetes	Bacteroidetes	Bacteroidales	Porphyromonadaceae	Porphyromonas	uncultured bacterium; P3D1-425; EF509229
			Bacteria	Bacteroidetes	Bacteroidetes	Bacteroidales	Porphyromonadaceae	Porphyromonas	Porphyromonas sp. oral clone AW032; AF393476
			Bacteria	Bacteroidetes	Bacteroidetes	Bacteroidales	Porphyromonadaceae	Porphyromonas	uncultured bacterium; P1D1-733; EF512005
			Bacteria	Bacteroidetes	Bacteroidetes	Bacteroidales	Porphyromonadaceae	Porphyromonas	uncultured bacterium; P1D1-698; EF511866
			Bacteria	Bacteroidetes	Bacteroidetes	Bacteroidales	Porphyromonadaceae	Porphyromonas	Porphyromonas catoniae (T); ATCC 51270; X82823
			Bacteria	Bacteroidetes	Bacteroidetes	Bacteroidales	Porphyromonadaceae	Porphyromonas	uncultured Bacteroidetes bacterium; S15B-MN34; AJ583190
			Bacteria	Bacteroidetes	Bacteroidetes	Bacteroidales	Porphyromonadaceae	Porphyromonas	uncultured bacterium; G3-4; AM179902
			Bacteria	Bacteroidetes	Bacteroidetes	Bacteroidales	Porphyromonadaceae	Porphyromonas	uncultured Bacteroidetes bacterium; WB1B005; AY862036
o1507	o1507 16S-PorOral	AAATTGTGGGGCTCAACTCC	Bacteria	Bacteroidetes	Bacteroidetes	Bacteroidales	unclassified_Bacteroidales		Porphyromonas-like sp. oral clone DA064; AY005071
			Bacteria	Bacteroidetes	Bacteroidetes	Bacteroidales	unclassified_Bacteroidales		uncultured Bacteroidetes bacterium; 701B10(oral); AM420249
			Bacteria	Bacteroidetes	Bacteroidetes	Bacteroidales	unclassified_Bacteroidales		Bacteroidales oral clone MCE7_120; AF481207
o1508	o1508 16S-Prbac	TCGCGTCGGGAGTGAAAAC	Bacteria	Actinobacteria	Actinobacteria	Actinomycetales	Propionibacteriaceae	unclassified_Propionibacteriaceae	Propionibacterium propionicum; DSM 43307T; AJ315953
			Bacteria	Actinobacteria	Actinobacteria	Actinomycetales	Nocardioidaceae	Aeromicrobium	Aeromicrobium fastidiosum (T); DSM 10552T; Z78209
			Bacteria	Actinobacteria	Actinobacteria	Actinomycetales	Nocardioidaceae	Nocardioides	Nocardioides plantarum (T); DSM 11054T; Z78211
			Bacteria	Actinobacteria	Actinobacteria	Actinomycetales	Nocardioidaceae	Nocardioides	Nocardioides simplex; DSM 20130T; Z78212
			Bacteria	Actinobacteria	Actinobacteria	Actinomycetales	Nocardioidaceae	Nocardioides	Nocardioides sp.; EL-17 lawson; X90830
			Bacteria	Actinobacteria	Actinobacteria	Actinomycetales	Nocardioidaceae	Nocardioides	Nocardioides plantarum; NCIMB 12834 (type strain); X69973
			Bacteria	Actinobacteria	Actinobacteria	Actinomycetales	Nocardioidaceae	Nocardioides	Nocardioides sp. ND6; type strain: ND6; AJ511294
			Bacteria	Actinobacteria	Actinobacteria	Actinomycetales	Nocardioidaceae	Nocardioides	unidentified eubacterium clone FJ21-A; U27856
			Bacteria	Actinobacteria	Actinobacteria	Actinomycetales	Nocardioidaceae	Nocardioides	unidentified eubacterium K44; AJ009714
			Bacteria	Actinobacteria	Actinobacteria	Actinomycetales	Nocardioidaceae	Nocardioides	Nocardioides sp. V4.BE.17; V4.BE.17 = MM_2350; AJ244657
			Bacteria	Actinobacteria	Actinobacteria	Actinomycetales	Nocardioidaceae	Nocardioides	uncultured bacterium; PeH25; AJ576342
			Bacteria	Actinobacteria	Actinobacteria	Actinomycetales	Nocardioidaceae	Nocardioides	Nocardioides sp.; X94145
			Bacteria	Actinobacteria	Actinobacteria	Actinomycetales	Nocardioidaceae	Nocardioides	Nocardioides sp. NS/27; AJ549286
			Bacteria	Actinobacteria	Actinobacteria	Actinomycetales	Nocardioidaceae	Nocardioides	Nocardioides sp.; 30-71; X86618
			Bacteria	Actinobacteria	Actinobacteria	Actinomycetales	Nocardioidaceae	Nocardioides	Actinobacteria PB90-4; AJ229240
			Bacteria	Actinobacteria	Actinobacteria	Actinomycetales	Nocardioidaceae	Nocardioides	Nocardioides sp. V4.ME.19; V4.ME.19 = MM_2333; AJ244656
			Bacteria	Actinobacteria	Actinobacteria	Actinomycetales	Nocardioidaceae	Nocardioides	Nocardioides sp. NS/33; AJ549287
			Bacteria	Actinobacteria	Actinobacteria	Actinomycetales	Nocardioidaceae	Nocardioides	Nocardioides albus (T); DSM 43109 (T).; X53211
			Bacteria	Actinobacteria	Actinobacteria	Actinomycetales	Nocardioidaceae	Nocardioides	Nocardioides sp. EPR 26 W; AY394879
			Bacteria	Actinobacteria	Actinobacteria	Actinomycetales	Nocardioidaceae	Nocardioides	unidentified bacterium; W4-B24; AY345502
o1509	o1509 16S-PreGroup1	ACTGACGCTAAAGCTCGAA	Bacteria	Bacteroidetes	Bacteroidetes	Bacteroidales	Prevotellaceae	Prevotella	Prevotella aff. ruminicola Tc2-24; AJ009933
			Bacteria	Bacteroidetes	Bacteroidetes	Bacteroidales	Prevotellaceae	Prevotella	Prevotella bryantii (T); B14 (DSM 11371, species type strain); AJ006457
			Bacteria	Bacteroidetes	Bacteroidetes	Bacteroidales	Prevotellaceae	Prevotella	unidentified rumen bacterium RC14; AF001705
			Bacteria	Bacteroidetes	Bacteroidetes	Bacteroidales	Prevotellaceae	Prevotella	Prevotella brevis; GA33; AJ011682
			Bacteria	Bacteroidetes	Bacteroidetes	Bacteroidales	Prevotellaceae	Prevotella	unidentified rumen bacterium RFN1; AF001777
			Bacteria	Bacteroidetes	Bacteroidetes	Bacteroidales	Prevotellaceae	Prevotella	unidentified rumen bacterium RC29; AF001731
			Bacteria	Bacteroidetes	Bacteroidetes	Bacteroidales	Prevotellaceae	Prevotella	unidentified rumen bacterium RF25; AF001757
			Bacteria	Bacteroidetes	Bacteroidetes	Bacteroidales	Prevotellaceae	Prevotella	unidentified rumen bacterium RF40; AF001771
			Bacteria	Bacteroidetes	Bacteroidetes	Bacteroidales	Prevotellaceae	Prevotella	unidentified rumen bacterium RF31; AF001763
			Bacteria	Bacteroidetes	Bacteroidetes	Bacteroidales	Prevotellaceae	Prevotella	unidentified rumen bacterium RF37; AF001768
			Bacteria	Bacteroidetes	Bacteroidetes	Bacteroidales	Prevotellaceae	Prevotella	unidentified rumen bacterium RC24; AF001714
			Bacteria	Bacteroidetes	Bacteroidetes	Bacteroidales	Prevotellaceae	Prevotella	unidentified rumen bacterium RF20; AF001752
			Bacteria	Bacteroidetes	Bacteroidetes	Bacteroidales	Prevotellaceae	Prevotella	unidentified rumen bacterium RF27; AF001759
			Bacteria	Bacteroidetes	Bacteroidetes	Bacteroidales	Prevotellaceae	Prevotella	unidentified rumen bacterium RF18; AF001750
			Bacteria	Bacteroidetes	Bacteroidetes	Bacteroidales	Prevotellaceae	Prevotella	unidentified rumen bacterium RC18; AF001709
			Bacteria	Bacteroidetes	Bacteroidetes	Bacteroidales	Prevotellaceae	Prevotella	unidentified rumen bacterium RF19; AF001751
			Bacteria	Bacteroidetes	Bacteroidetes	Bacteroidales	Prevotellaceae	Prevotella	unidentified rumen bacterium RC40; AF001723
			Bacteria	Bacteroidetes	Bacteroidetes	Bacteroidales	Prevotellaceae	Prevotella	unidentified rumen bacterium RF23; AF001755
			Bacteria	Bacteroidetes	Bacteroidetes	Bacteroidales	Prevotellaceae	Prevotella	unidentified rumen bacterium RFP6; AF001773
			Bacteria	Bacteroidetes	Bacteroidetes	Bacteroidales	Prevotellaceae	Prevotella	unidentified rumen bacterium RCP19; AF001729
o1510	o1510 16S-PreGroup1(	ACCGCATGTTCTTCGATGAC	Bacteria	Bacteroidetes	Bacteroidetes	Bacteroidales	Prevotellaceae	Prevotella	Prevotella denticola; ATCC 35308; AY323524
			Bacteria	Bacteroidetes	Bacteroidetes	Bacteroidales	Prevotellaceae	Prevotella	Prevotella sp. oral clone AO036; AY005054
			Bacteria	Bacteroidetes	Bacteroidetes	Bacteroidales	Prevotellaceae	Prevotella	uncultured bacterium; P3D1-660; EF509226
			Bacteria	Bacteroidetes	Bacteroidetes	Bacteroidales	Prevotellaceae	Prevotella	uncultured Prevotella sp.; EHFS1_S14a; EU071526
			Bacteria	Bacteroidetes	Bacteroidetes	Bacteroidales	Prevotellaceae	Prevotella	uncultured bacterium; P3D1-418; EF509134
			Bacteria	Bacteroidetes	Bacteroidetes	Bacteroidales	Prevotellaceae	Prevotella	uncultured bacterium; P3D1-386; EF509246
			Bacteria	Bacteroidetes	Bacteroidetes	Bacteroidales	Prevotellaceae	Prevotella	uncultured bacterium; P3D1-391; EF509242
			Bacteria	Bacteroidetes	Bacteroidetes	Bacteroidales	Prevotellaceae	Prevotella	uncultured bacterium; P3D1-471; EF509155

			Bacteria	Bacteroidetes	Bacteroidetes	Bacteroidales	Prevotellaceae	Prevotella	Prevotella sp. oral clone AH005; AY005053
			Bacteria	Bacteroidetes	Bacteroidetes	Bacteroidales	Prevotellaceae	Prevotella	uncultured bacterium; P3D1-459; EF509179
			Bacteria	Bacteroidetes	Bacteroidetes	Bacteroidales	Prevotellaceae	Prevotella	Prevotella denticola; L16466
			Bacteria	Bacteroidetes	Bacteroidetes	Bacteroidales	Prevotellaceae	Prevotella	uncultured bacterium; nbu484a02c1; GQ050145
			Bacteria	Bacteroidetes	Bacteroidetes	Bacteroidales	Prevotellaceae	Prevotella	uncultured bacterium; nbu361b08c1; GQ036179
			Bacteria	Bacteroidetes	Bacteroidetes	Bacteroidales	Prevotellaceae	Prevotella	uncultured bacterium; nbu484b02c1; GQ050152
			Bacteria	Bacteroidetes	Bacteroidetes	Bacteroidales	Prevotellaceae	Prevotella	uncultured bacterium; nbw711c06c1; GQ103518
			Bacteria	Bacteroidetes	Bacteroidetes	Bacteroidales	Prevotellaceae	Prevotella	uncultured Prevotella sp.; S702; FJ976440
			Bacteria	Bacteroidetes	Bacteroidetes	Bacteroidales	Prevotellaceae	Prevotella	Prevotella denticola (T); L16467
o1511	o1511 16S-PreGroup1	AAAGTGAGGGACGTGTCCTT	Bacteria	Bacteroidetes	Bacteroidetes	Bacteroidales	Prevotellaceae	Prevotella	uncultured Prevotellaceae bacterium; 202F01(oral); AM420047
			Bacteria	Bacteroidetes	Bacteroidetes	Bacteroidales	Prevotellaceae	Prevotella	uncultured Prevotellaceae bacterium; 402B02(oral); AM420137
			Bacteria	Bacteroidetes	Bacteroidetes	Bacteroidales	Prevotellaceae	Prevotella	uncultured Prevotellaceae bacterium; 501E09(oral); AM420011
			Bacteria	Bacteroidetes	Bacteroidetes	Bacteroidales	Prevotellaceae	Prevotella	uncultured Prevotellaceae bacterium; 702G11(oral); AM420284
			Bacteria	Bacteroidetes	Bacteroidetes	Bacteroidales	Porphyromonadaceae	unclassified_Porphyromonadaceae	uncultured bacterium; RsaHf278; AY571450
			Bacteria	Bacteroidetes	Bacteroidetes	Bacteroidales	Prevotellaceae	Prevotella	uncultured bacterium; P3D1-658; EF509174
			Bacteria	Bacteroidetes	Bacteroidetes	Bacteroidales	Prevotellaceae	Prevotella	Prevotella maculosa; W1609; EF534314
			Bacteria	Bacteroidetes	Bacteroidetes	Bacteroidales	Prevotellaceae	Prevotella	Prevotella maculosa; W1609; EF534315
			Bacteria	Bacteroidetes	Bacteroidetes	Bacteroidales	Prevotellaceae	Prevotella	Prevotella sp. oral clone AA016; AY005058
			Bacteria	Bacteroidetes	Bacteroidetes	Bacteroidales	Prevotellaceae	Prevotella	uncultured bacterium; P3D1-653; EF509109
			Bacteria	Bacteroidetes	Bacteroidetes	Bacteroidales	Prevotellaceae	Prevotella	uncultured bacterium; P3D1-634; EF509252
			Bacteria	Bacteroidetes	Bacteroidetes	Bacteroidales	Prevotellaceae	Prevotella	uncultured bacterium; P3D1-591; EF509144
			Bacteria	Bacteroidetes	Bacteroidetes	Bacteroidales	Prevotellaceae	Prevotella	unidentified eubacterium clone 3.3; U43698
			Bacteria	Bacteroidetes	Bacteroidetes	Bacteroidales	Prevotellaceae	Prevotella	Prevotella sp. oral clone AA020; AY005057
			Bacteria	Bacteroidetes	Bacteroidetes	Bacteroidales	Prevotellaceae	Prevotella	Prevotella sp. oral clone F045; AY005056
			Bacteria	Bacteroidetes	Bacteroidetes	Bacteroidales	Prevotellaceae	Prevotella	uncultured bacterium; P3D1-654; EF509128
			Bacteria	Bacteroidetes	Bacteroidetes	Bacteroidales	Prevotellaceae	Prevotella	uncultured eubacterium E1-K13; AJ289182
			Bacteria	Bacteroidetes	Bacteroidetes	Bacteroidales	Prevotellaceae	Prevotella	uncultured bacterium; P3D1-458; EF509131
			Bacteria	Bacteroidetes	Bacteroidetes	Bacteroidales	Prevotellaceae	Prevotella	Prevotella oris (T); L16474
			Bacteria	Bacteroidetes	Bacteroidetes	Bacteroidales	Prevotellaceae	Prevotella	uncultured bacterium; LH76; AY978035
o1512	o1512 16S-PreGroup3	AGCTACAGGCCTAACACATG	Bacteria	Bacteroidetes	Bacteroidetes	Bacteroidales	Prevotellaceae	Prevotella	Prevotella sp. oral clone FL019; AY349392
			Bacteria	Bacteroidetes	Bacteroidetes	Bacteroidales	Bacteroidaceae	Bacteroides	uncultured bacterium; SJTU_E_08_46; EF400067
			Bacteria	Bacteroidetes	Bacteroidetes	Bacteroidales	Prevotellaceae	Hallella	uncultured rumen bacterium; U29-C10; AB185769
			Bacteria	Bacteroidetes	Bacteroidetes	Bacteroidales	Prevotellaceae	Prevotella	Prevotella multisaccharivorax (T); JCM 12954; AB200414
			Bacteria	Bacteroidetes	Bacteroidetes	Bacteroidales	Bacteroidaceae	Bacteroides	uncultured bacterium; SJTU_G_03_40; EF405275
			Bacteria	Bacteroidetes	Bacteroidetes	Bacteroidales	Bacteroidaceae	Bacteroides	uncultured bacterium; CFT112E8; DQ456032
			Bacteria	Bacteroidetes	Bacteroidetes	Bacteroidales	Bacteroidaceae	Bacteroides	uncultured bacterium; C2-12; DQ113672
			Bacteria	Bacteroidetes	Bacteroidetes	Bacteroidales	Bacteroidaceae	Bacteroides	uncultured bacterium; CFT112F7; DQ456039
			Bacteria	Bacteroidetes	Bacteroidetes	Bacteroidales	Prevotellaceae	Hallella	uncultured rumen bacterium; YRC34; EU259410
			Bacteria	Bacteroidetes	Bacteroidetes	Bacteroidales	Prevotellaceae	Prevotella	Prevotella pleuritidis; JCM 14110; AB278593
			Bacteria	Bacteroidetes	Bacteroidetes	Bacteroidales	Prevotellaceae	Prevotella	uncultured bacterium; p-1939-s962-3; AF371901
			Bacteria	Bacteroidetes	Bacteroidetes	Bacteroidales	Bacteroidaceae	Bacteroides	uncultured bacterium; CFT112B6; DQ456003
			Bacteria	Bacteroidetes	Bacteroidetes	Bacteroidales	Prevotellaceae	Prevotella	uncultured bacterium; p-1922-s962-3; AF371902
			Bacteria	Bacteroidetes	Bacteroidetes	Bacteroidales	Bacteroidaceae	Bacteroides	uncultured bacterium; CFT112A8; DQ455996
			Bacteria	Bacteroidetes	Bacteroidetes	Bacteroidales	Prevotellaceae	Prevotella	Prevotella sp. oral clone CY006; AY005063
			Bacteria	Bacteroidetes	Bacteroidetes	Bacteroidales	Prevotellaceae	Prevotella	Prevotella enoeca (T); ATCC 51261; AJ005635
			Bacteria	Bacteroidetes	Bacteroidetes	Bacteroidales	Porphyromonadaceae	unclassified_Porphyromonadaceae	uncultured bacterium; ratAN060309C; AM265440
			Bacteria	Bacteroidetes	Bacteroidetes	Bacteroidales	Bacteroidaceae	Bacteroides	uncultured bacterium; NF15; AY978889
			Bacteria	Bacteroidetes	Bacteroidetes	Bacteroidales	Bacteroidaceae	Bacteroides	uncultured bacterium; K427; AY976809
			Bacteria	Bacteroidetes	Bacteroidetes	Bacteroidales	Bacteroidaceae	Bacteroides	uncultured bacterium; Y486; AY975902
o1513	o1513 16S-PreGroup4	GCGTATCCAACCTTCCCG	Bacteria	Bacteroidetes	Bacteroidetes	Bacteroidales	Prevotellaceae	Prevotella	Prevotella sp. RS2; AY158021
			Bacteria	Bacteroidetes	Bacteroidetes	Bacteroidales	Prevotellaceae	Prevotella	Prevotella sp. DJF_LS17; EU728743
			Bacteria	Bacteroidetes	Bacteroidetes	Bacteroidales	Prevotellaceae	Prevotella	Prevotella sp. E9_42; AF481229
			Bacteria	Bacteroidetes	Bacteroidetes	Bacteroidales	Prevotellaceae	Prevotella	Prevotella multiformis; PPPA19; AB182484
			Bacteria	Bacteroidetes	Bacteroidetes	Bacteroidales	Prevotellaceae	Prevotella	Prevotella multiformis; PPPA30; AB182486
			Bacteria	Bacteroidetes	Bacteroidetes	Bacteroidales	Prevotellaceae	Prevotella	Prevotella multiformis (T); PPPA21; AB182483
			Bacteria	Bacteroidetes	Bacteroidetes	Bacteroidales	Prevotellaceae	Prevotella	Prevotella multiformis; PPPA28; AB182485
			Bacteria	Bacteroidetes	Bacteroidetes	Bacteroidales	Prevotellaceae	Prevotella	Prevotella denticola; ATCC 35308; AY323524
			Bacteria	Bacteroidetes	Bacteroidetes	Bacteroidales	Prevotellaceae	Prevotella	Prevotella baroniae (T); E9.33; AY840553
			Bacteria	Bacteroidetes	Bacteroidetes	Bacteroidales	Prevotellaceae	Prevotella	Prevotella genomosp. P4; P4P_53; AY944134
			Bacteria	Bacteroidetes	Bacteroidetes	Bacteroidales	Prevotellaceae	Prevotella	Prevotella buccae (T); L16478
			Bacteria	Bacteroidetes	Bacteroidetes	Bacteroidales	Bacteroidaceae	Bacteroides	uncultured bacterium; KO1_aai41b01; EU460960
			Bacteria	Bacteroidetes	Bacteroidetes	Bacteroidales	Bacteroidaceae	Bacteroides	uncultured bacterium; KO1_aai41g02; EU460992
			Bacteria	Bacteroidetes	Bacteroidetes	Bacteroidales	Bacteroidaceae	Bacteroides	uncultured bacterium; COL_aai14h02; EU460113
			Bacteria	Bacteroidetes	Bacteroidetes	Bacteroidales	Bacteroidaceae	Bacteroides	uncultured bacterium; KO1_aai41a01; EU460953
			Bacteria	Bacteroidetes	Bacteroidetes	Bacteroidales	Bacteroidaceae	Bacteroides	uncultured bacterium; COL_aai14c06; EU460083
			Bacteria	Bacteroidetes	Bacteroidetes	Bacteroidales	Prevotellaceae	Prevotella	Prevotella buccae (T); L16477
			Bacteria	Bacteroidetes	Bacteroidetes	Bacteroidales	Prevotellaceae	Prevotella	Prevotella denticola; L16466
			Bacteria	Bacteroidetes	Bacteroidetes	Bacteroidales	Prevotellaceae	Prevotella	bacterium mpn-isolate group 4; AF357552
			Bacteria	Bacteroidetes	Bacteroidetes	Bacteroidales	Prevotellaceae	Prevotella	Prevotella denticola (T); L16467
o1514	o1514 16S-PreGroup5	GTTTAAAGGGAGTGTAGGCG	Bacteria	Bacteroidetes	Bacteroidetes	Bacteroidales	Prevotellaceae	Prevotella	uncultured bacterium; MB-A2-107; AY093462
			Bacteria	Bacteroidetes	Bacteroidetes	Bacteroidales	Prevotellaceae	Prevotella	Prevotella pallens (T); 10371; Y13105
			Bacteria	Bacteroidetes	Bacteroidetes	Bacteroidales	Prevotellaceae	Prevotella	Prevotella nigrescens; NCTC 9336; X73963
			Bacteria	Bacteroidetes	Bacteroidetes	Bacteroidales	Prevotellaceae	Prevotella	Prevotella intermedia (T); ATCC 25611; X73965
			Bacteria	Bacteroidetes	Bacteroidetes	Bacteroidales	Prevotellaceae	Prevotella	Prevotella pallens; 9423; Y13106
			Bacteria	Bacteroidetes	Bacteroidetes	Bacteroidales	Prevotellaceae	Prevotella	uncultured eubacterium E1-K11; AJ289187
			Bacteria	Bacteroidetes	Bacteroidetes	Bacteroidales	Prevotellaceae	Prevotella	Prevotella pallens; 8792; Y13107
			Bacteria	Bacteroidetes	Bacteroidetes	Bacteroidales	Prevotellaceae	Prevotella	Prevotella intermedia; 32-PIN; AF414824
			Bacteria	Bacteroidetes	Bacteroidetes	Bacteroidales	Prevotellaceae	Prevotella	Prevotella intermedia; 10-PIN; AF414827
			Bacteria	Bacteroidetes	Bacteroidetes	Bacteroidales	Prevotellaceae	Prevotella	Prevotella intermedia; 15-PIN; AF414828
			Bacteria	Bacteroidetes	Bacteroidetes	Bacteroidales	Prevotellaceae	Prevotella	Prevotella intermedia; 16-PIN; AF414829
			Bacteria	Bacteroidetes	Bacteroidetes	Bacteroidales	Prevotellaceae	Prevotella	Prevotella intermedia; 35-PIN; AF414825
			Bacteria	Bacteroidetes	Bacteroidetes	Bacteroidales	Prevotellaceae	Prevotella	Prevotella intermedia; 5-PIN; AF414822
			Bacteria	Bacteroidetes	Bacteroidetes	Bacteroidales	Prevotellaceae	Prevotella	Prevotella intermedia; 22-PIN; AF414830
			Bacteria	Bacteroidetes	Bacteroidetes	Bacteroidales	Prevotellaceae	Prevotella	Prevotella intermedia; 23-PIN; AF414831
			Bacteria	Bacteroidetes	Bacteroidetes	Bacteroidales	Prevotellaceae	Prevotella	Prevotella intermedia; 24-PIN; AF414832
			Bacteria	Bacteroidetes	Bacteroidetes	Bacteroidales	Prevotellaceae	Prevotella	Prevotella intermedia; 36-PIN; AF414826
			Bacteria	Bacteroidetes	Bacteroidetes	Bacteroidales	Prevotellaceae	Prevotella	Prevotella intermedia; 6-PIN; AF414823
			Bacteria	Bacteroidetes	Bacteroidetes	Bacteroidales	Prevotellaceae	Prevotella	Prevotella nigrescens; ATCC 33563; AF414833

			Bacteria	Bacteroidetes	Bacteroidetes	Bacteroidales	Prevotellaceae	Prevotella	Prevotella intermedia; ATCC 25611; AF414821
o1515	o1515 16S-PreGroup6	CAACTGACGCTTAAGCTCGA	Bacteria	Bacteroidetes	Bacteroidetes	Bacteroidales	Prevotellaceae	Prevotella	Prevotella sp. oral clone FU048; AY349393
			Bacteria	Bacteroidetes	Bacteroidetes	Bacteroidales	Prevotellaceae	Prevotella	Prevotella sp. oral clone GI030; AY349395
			Bacteria	Bacteroidetes	Bacteroidetes	Bacteroidales	Prevotellaceae	Prevotella	Prevotella micans; E7.56; AF481228
			Bacteria	Bacteroidetes	Bacteroidetes	Bacteroidales	Prevotellaceae	Prevotella	Prevotella sp. oral clone HB034; DQ087190
			Bacteria	Bacteroidetes	Bacteroidetes	Bacteroidales	Prevotellaceae	Prevotella	Prevotella sp. oral clone FM005; AF432133
			Bacteria	Bacteroidetes	Bacteroidetes	Bacteroidales	Prevotellaceae	Prevotella	Prevotella melaninogenica; ATCC 25845; AY323525
			Bacteria	Bacteroidetes	Bacteroidetes	Bacteroidales	Prevotellaceae	Prevotella	uncultured bacterium; P2D1-571; EF510358
			Bacteria	Bacteroidetes	Bacteroidetes	Bacteroidales	Prevotellaceae	Prevotella	Prevotella bergensis (T); 94067913; AY350613
			Bacteria	Bacteroidetes	Bacteroidetes	Bacteroidales	Prevotellaceae	Prevotella	Prevotella veroralis (T); L16473
			Bacteria	Bacteroidetes	Bacteroidetes	Bacteroidales	Prevotellaceae	Prevotella	Prevotella genomosp. P7 oral clone MB2_P31; DQ003620
			Bacteria	Bacteroidetes	Bacteroidetes	Bacteroidales	Prevotellaceae	Prevotella	uncultured bacterium; P2D1-492; EF510344
			Bacteria	Bacteroidetes	Bacteroidetes	Bacteroidales	Prevotellaceae	Prevotella	uncultured bacterium; P2D1-575; EF510350
			Bacteria	Bacteroidetes	Bacteroidetes	Bacteroidales	Prevotellaceae	Prevotella	uncultured bacterium; P2D1-762; EF510360
			Bacteria	Bacteroidetes	Bacteroidetes	Bacteroidales	Prevotellaceae	Prevotella	uncultured bacterium; P2D1-486; EF510347
			Bacteria	Bacteroidetes	Bacteroidetes	Bacteroidales	Prevotellaceae	Prevotella	uncultured bacterium; P2D1-744; EF510349
			Bacteria	Bacteroidetes	Bacteroidetes	Bacteroidales	Prevotellaceae	Prevotella	uncultured bacterium; P2D1-767; EF510352
			Bacteria	Bacteroidetes	Bacteroidetes	Bacteroidales	Prevotellaceae	Prevotella	Prevotella melaninogenica; L16470
			Bacteria	Bacteroidetes	Bacteroidetes	Bacteroidales	Prevotellaceae	Prevotella	uncultured bacterium; P2D1-520; EF510343
			Bacteria	Bacteroidetes	Bacteroidetes	Bacteroidales	Prevotellaceae	Prevotella	uncultured bacterium; P2D1-726; EF510346
			Bacteria	Bacteroidetes	Bacteroidetes	Bacteroidales	Prevotellaceae	Prevotella	Prevotella melaninogenica (T); L16469
o1516	o1516 16S-PreGroup7	AACCAGCCAAGTAGCGTGC	Bacteria	Bacteroidetes	Bacteroidetes	Bacteroidales	Prevotellaceae	Prevotella	Prevotella albensis (T); M384; AJ011683
			Bacteria	Bacteroidetes	Bacteroidetes	Bacteroidales	Prevotellaceae	Prevotella	Prevotella sp. Smarlab 121567; AF487886
			Bacteria	Bacteroidetes	Bacteroidetes	Bacteroidales	Prevotellaceae	Prevotella	Prevotella aff. ruminicola Tc2-24; AJ009933
			Bacteria	Bacteroidetes	Bacteroidetes	Bacteroidales	Prevotellaceae	Prevotella	Prevotella bryantii (T); B14 (DSM 11371, species type strain); AJ006457
			Bacteria	Bacteroidetes	Bacteroidetes	Bacteroidales	Prevotellaceae	Prevotella	uncultured Prevotella PUS9.180; AJ012605
			Bacteria	Bacteroidetes	Bacteroidetes	Bacteroidales	Prevotellaceae	Prevotella	Prevotella pallens (T); 10371; Y13105
			Bacteria	Bacteroidetes	Bacteroidetes	Bacteroidales	Prevotellaceae	Prevotella	Prevotella nigrescens; NCTC 9336; X73963
			Bacteria	Bacteroidetes	Bacteroidetes	Bacteroidales	Prevotellaceae	Prevotella	Prevotella intermedia (T); ATCC 25611; X73965
			Bacteria	Bacteroidetes	Bacteroidetes	Bacteroidales	Prevotellaceae	Prevotella	Prevotella brevis; GA33; AJ011682
			Bacteria	Bacteroidetes	Bacteroidetes	Bacteroidales	Prevotellaceae	Prevotella	uncultured eubacterium E1-K13; AJ289182
			Bacteria	Bacteroidetes	Bacteroidetes	Bacteroidales	Prevotellaceae	Prevotella	Prevotella enoeca (T); ATCC 51261; AJ005635
			Bacteria	Bacteroidetes	Bacteroidetes	Bacteroidales	Prevotellaceae	Prevotella	uncultured bacterium; HuCC28; AJ315483
			Bacteria	Bacteroidetes	Bacteroidetes	Bacteroidales	Prevotellaceae	Prevotella	Prevotella pallens; 9423; Y13106
			Bacteria	Bacteroidetes	Bacteroidetes	Bacteroidales	Prevotellaceae	Prevotella	uncultured eubacterium E1-K8; AJ289176
			Bacteria	Bacteroidetes	Bacteroidetes	Bacteroidales	Prevotellaceae	Prevotella	uncultured eubacterium E1-K11; AJ289187
			Bacteria	Bacteroidetes	Bacteroidetes	Bacteroidales	Prevotellaceae	Prevotella	Prevotella pallens; 8792; Y13107
			Bacteria	Bacteroidetes	Bacteroidetes	Bacteroidales	Prevotellaceae	Prevotella	uncultured eubacterium E1-K12; AJ289169
			Bacteria	Bacteroidetes	Bacteroidetes	Bacteroidales	Prevotellaceae	Prevotella	Bacteroidales str. KB9; AB078829
			Bacteria	Bacteroidetes	Bacteroidetes	Bacteroidales	Prevotellaceae	Prevotella	Prevotella paludivivens; A42; AB081578
			Bacteria	Bacteroidetes	Bacteroidetes	Bacteroidales	Prevotellaceae	Prevotella	Bacteroidales str. KB12; AB078832
o1517	o1517 16S-PreGroup8	CAGCGCATACTGGTTTCCTT	Bacteria	Bacteroidetes	Bacteroidetes	Bacteroidales	Prevotellaceae	Prevotella	uncultured rumen bacterium; U29-A10; AB185747
			Bacteria	Bacteroidetes	Bacteroidetes	Bacteroidales	Prevotellaceae	Prevotella	uncultured rumen bacterium 4C3d-6; AB034108
			Bacteria	Bacteroidetes	Bacteroidetes	Bacteroidales	Prevotellaceae	Prevotella	Prevotella sp. oral clone FU048; AY349393
			Bacteria	Bacteroidetes	Bacteroidetes	Bacteroidales	Prevotellaceae	Prevotella	Prevotella sp. oral clone GI030; AY349395
			Bacteria	Bacteroidetes	Bacteroidetes	Bacteroidales	Prevotellaceae	Prevotella	Prevotella sp. oral clone GI059; AY349397
			Bacteria	Bacteroidetes	Bacteroidetes	Bacteroidales	Prevotellaceae	Prevotella	uncultured bacterium; rRNA247; AY959020
			Bacteria	Bacteroidetes	Bacteroidetes	Bacteroidales	Prevotellaceae	Prevotella	Prevotella sp. oral clone HB034; DQ087190
			Bacteria	Bacteroidetes	Bacteroidetes	Bacteroidales	Prevotellaceae	Prevotella	Prevotella sp. oral clone DO027; AF385511
			Bacteria	Bacteroidetes	Bacteroidetes	Bacteroidales	Prevotellaceae	Prevotella	Prevotella sp. oral clone FM005; AF432133
			Bacteria	Bacteroidetes	Bacteroidetes	Bacteroidales	Prevotellaceae	Prevotella	Prevotella sp. oral clone BE073; AF385551
			Bacteria	Bacteroidetes	Bacteroidetes	Bacteroidales	Prevotellaceae	Prevotella	Prevotella melaninogenica; ATCC 25845; AY323525
			Bacteria	Bacteroidetes	Bacteroidetes	Bacteroidales	Prevotellaceae	Prevotella	Prevotella veroralis (T); L16473
			Bacteria	Bacteroidetes	Bacteroidetes	Bacteroidales	Prevotellaceae	Prevotella	Prevotella veroralis; ATCC 33779; AY836507
			Bacteria	Bacteroidetes	Bacteroidetes	Bacteroidales	Prevotellaceae	Prevotella	Prevotella melaninogenica; L16470
			Bacteria	Bacteroidetes	Bacteroidetes	Bacteroidales	Prevotellaceae	Prevotella	uncultured bacterium; ME48; AY916232
			Bacteria	Bacteroidetes	Bacteroidetes	Bacteroidales	Prevotellaceae	Prevotella	Prevotella sp. N19-31; AY880054
			Bacteria	Bacteroidetes	Bacteroidetes	Bacteroidales	Prevotellaceae	Prevotella	Prevotella melaninogenica (T); L16469
			Bacteria	Bacteroidetes	Bacteroidetes	Bacteroidales	Prevotellaceae	Prevotella	Prevotella sp. N12-20; AY880052
			Bacteria	Bacteroidetes	Bacteroidetes	Bacteroidales	Prevotellaceae	Prevotella	Prevotella sp. T05-04; AY880055
			Bacteria	Bacteroidetes	Bacteroidetes	Bacteroidales	Prevotellaceae	Prevotella	Prevotella sp. N19-22; AY880053
o1518	o1518 16S-PreGroup9	GGGTTTAAAGGGAGTGTAGG	Bacteria	Bacteroidetes	Bacteroidetes	Bacteroidales	Prevotellaceae	Prevotella	uncultured bacterium; MB-A2-107; AY093462
			Bacteria	Bacteroidetes	Bacteroidetes	Bacteroidales	Prevotellaceae	Prevotella	Prevotella pallens (T); 10371; Y13105
			Bacteria	Bacteroidetes	Bacteroidetes	Bacteroidales	Prevotellaceae	Prevotella	Prevotella nigrescens; NCTC 9336; X73963
			Bacteria	Bacteroidetes	Bacteroidetes	Bacteroidales	Prevotellaceae	Prevotella	Prevotella intermedia (T); ATCC 25611; X73965
			Bacteria	Bacteroidetes	Bacteroidetes	Bacteroidales	Prevotellaceae	Prevotella	Prevotella pallens; 9423; Y13106
			Bacteria	Bacteroidetes	Bacteroidetes	Bacteroidales	Prevotellaceae	Prevotella	uncultured eubacterium E1-K11; AJ289187
			Bacteria	Bacteroidetes	Bacteroidetes	Bacteroidales	Prevotellaceae	Prevotella	Prevotella pallens; 8792; Y13107
			Bacteria	Bacteroidetes	Bacteroidetes	Bacteroidales	Prevotellaceae	Prevotella	Prevotella intermedia; 32-PIN; AF414824
			Bacteria	Bacteroidetes	Bacteroidetes	Bacteroidales	Prevotellaceae	Prevotella	Prevotella intermedia; 10-PIN; AF414827
			Bacteria	Bacteroidetes	Bacteroidetes	Bacteroidales	Prevotellaceae	Prevotella	Prevotella intermedia; 15-PIN; AF414828
			Bacteria	Bacteroidetes	Bacteroidetes	Bacteroidales	Prevotellaceae	Prevotella	Prevotella intermedia; 16-PIN; AF414829
			Bacteria	Bacteroidetes	Bacteroidetes	Bacteroidales	Prevotellaceae	Prevotella	Prevotella intermedia; 35-PIN; AF414825
			Bacteria	Bacteroidetes	Bacteroidetes	Bacteroidales	Prevotellaceae	Prevotella	Prevotella intermedia; 5-PIN; AF414822
			Bacteria	Bacteroidetes	Bacteroidetes	Bacteroidales	Prevotellaceae	Prevotella	Prevotella intermedia; 22-PIN; AF414830
			Bacteria	Bacteroidetes	Bacteroidetes	Bacteroidales	Prevotellaceae	Prevotella	Prevotella intermedia; 23-PIN; AF414831
			Bacteria	Bacteroidetes	Bacteroidetes	Bacteroidales	Prevotellaceae	Prevotella	Prevotella intermedia; 24-PIN; AF414832
			Bacteria	Bacteroidetes	Bacteroidetes	Bacteroidales	Prevotellaceae	Prevotella	Prevotella intermedia; 36-PIN; AF414826
			Bacteria	Bacteroidetes	Bacteroidetes	Bacteroidales	Prevotellaceae	Prevotella	Prevotella intermedia; 6-PIN; AF414823
			Bacteria	Bacteroidetes	Bacteroidetes	Bacteroidales	Prevotellaceae	Prevotella	Prevotella nigrescens; ATCC 33563; AF414833
			Bacteria	Bacteroidetes	Bacteroidetes	Bacteroidales	Prevotellaceae	Prevotella	Prevotella intermedia; ATCC 25611; AF414821
o1519	o1519 16S-PreOral	AAAGTCGGCCTAATGCCCTA	Bacteria	Bacteroidetes	Bacteroidetes	Bacteroidales	Prevotellaceae	Prevotella	Prevotella sp. oral clone FL019; AY349392
			Bacteria	Bacteroidetes	Bacteroidetes	Bacteroidales	Prevotellaceae	Prevotella	uncultured Prevotella sp.; 102D01(oral); AM419992
			Bacteria	Bacteroidetes	Bacteroidetes	Bacteroidales	Prevotellaceae	Prevotella	uncultured bacterium; MA01F11; FM873532
			Bacteria	Bacteroidetes	Bacteroidetes	Bacteroidales	Prevotellaceae	Prevotella	Prevotella pleuritidis; JCM 14110; AB278593
			Bacteria	Bacteroidetes	Bacteroidetes	Bacteroidales	Prevotellaceae	Prevotella	Prevotella sp. oral clone CY006; AY005063
			Bacteria	Bacteroidetes	Bacteroidetes	Bacteroidales	Prevotellaceae	Prevotella	uncultured Prevotella sp.; 24B764; FJ976412
o1520	o1520 16S-PreTan	AAGGCCCTTGTGGTCGTAAA	Bacteria	Bacteroidetes	Bacteroidetes	Bacteroidales	Prevotellaceae	Prevotella	Prevotella tannerae; 93-1-2; AF183406

			Bacteria	Bacteroidetes	Bacteroidetes	Bacteroidales	Prevotellaceae	Prevotella	Prevotella tannerae; 29-1; AF183403
			Bacteria	Bacteroidetes	Bacteroidetes	Bacteroidales	Prevotellaceae	Prevotella	Prevotella tannerae; 89-9-1; AF183405
			Bacteria	Bacteroidetes	Bacteroidetes	Bacteroidales	Prevotellaceae	Prevotella	Prevotella tannerae; 131-9-1; AF183402
			Bacteria	Bacteroidetes	Bacteroidetes	Bacteroidales	Prevotellaceae	Prevotella	uncultured bacterium; P3D1-426; EF509158
			Bacteria	Bacteroidetes	Bacteroidetes	Bacteroidales	Prevotellaceae	Prevotella	Prevotella tannerae; 83-10-2; AF183404
			Bacteria	Bacteroidetes	Bacteroidetes	Bacteroidales	Prevotellaceae	Prevotella	uncultured bacterium; MB-A2-118; AY093466
			Bacteria	Bacteroidetes	Bacteroidetes	Bacteroidales	Prevotellaceae	Prevotella	uncultured bacterium; P3D1-397; EF509162
			Bacteria	Bacteroidetes	Bacteroidetes	Bacteroidales	Prevotellaceae	Prevotella	Prevotella tannerae (T); ATCC 51259; AJ005634
			Bacteria	Bacteroidetes	Bacteroidetes	Bacteroidales	Prevotellaceae	Prevotella	uncultured bacterium; P3D1-666; EF509195
			Bacteria	Bacteroidetes	Bacteroidetes	Bacteroidales	Prevotellaceae	Prevotella	uncultured bacterium; P3D1-650; EF509220
			Bacteria	Bacteroidetes	Bacteroidetes	Bacteroidales	Prevotellaceae	Prevotella	uncultured bacterium; P3D1-388; EF509076
			Bacteria	Bacteroidetes	Bacteroidetes	Bacteroidales	Prevotellaceae	Prevotella	uncultured eubacterium E1-K12; AJ289169
			Bacteria	Bacteroidetes	Bacteroidetes	Bacteroidales	Prevotellaceae	Prevotella	uncultured Prevotella sp.; 3714; FJ976221
			Bacteria	Bacteroidetes	Bacteroidetes	Bacteroidales	Prevotellaceae	Prevotella	uncultured bacterium; nbu87b06c1; GQ006741
			Bacteria	Bacteroidetes	Bacteroidetes	Bacteroidales	Prevotellaceae	Prevotella	uncultured bacterium; nbu87d08c1; GQ006765
			Bacteria	Bacteroidetes	Bacteroidetes	Bacteroidales	Prevotellaceae	Prevotella	uncultured bacterium; nbu88h07c1; GQ006879
			Bacteria	Bacteroidetes	Bacteroidetes	Bacteroidales	Prevotellaceae	Prevotella	uncultured bacterium; nbu87e06c1; GQ006774
			Bacteria	Bacteroidetes	Bacteroidetes	Bacteroidales	Prevotellaceae	Prevotella	uncultured bacterium; nbu86f06c1; GQ006702
			Bacteria	Bacteroidetes	Bacteroidetes	Bacteroidales	Prevotellaceae	Prevotella	uncultured bacterium; nbu88d07c1; GQ006841
o1521	o1521 16S-ProAcn1	CCTGCTTTTGTGGGGTGCT	Bacteria	Actinobacteria	Actinobacteria	Actinomycetales	Corynebacteriaceae	Corynebacterium	Corynebacterium atypicum (T); CCUG 45804; AJ441057
			Bacteria	Actinobacteria	Actinobacteria	Actinomycetales	Propionibacteriaceae	Propionibacterium	Propionibacterium acnes; #3004; AB041618
			Bacteria	Actinobacteria	Actinobacteria	Actinomycetales	Propionibacteriaceae	Propionibacterium	Propionibacterium acnes; #7168; AB042289
			Bacteria	Actinobacteria	Actinobacteria	Actinomycetales	Propionibacteriaceae	Propionibacterium	Propionibacterium acnes; #5317; AB108480
			Bacteria	Actinobacteria	Actinobacteria	Actinomycetales	Propionibacteriaceae	Propionibacterium	Propionibacterium acnes; #1003; AB108483
			Bacteria	Actinobacteria	Actinobacteria	Actinomycetales	Propionibacteriaceae	Propionibacterium	Propionibacterium acnes; #2929; AB108484
			Bacteria	Actinobacteria	Actinobacteria	Actinomycetales	Propionibacteriaceae	Propionibacterium	Propionibacterium acnes; #6119; AB108479
			Bacteria	Actinobacteria	Actinobacteria	Actinomycetales	Propionibacteriaceae	Propionibacterium	Propionibacterium acnes; #1447; AB041617
			Bacteria	Actinobacteria	Actinobacteria	Actinomycetales	Propionibacteriaceae	Propionibacterium	Propionibacterium acnes; #4584; AB042287
			Bacteria	Actinobacteria	Actinobacteria	Actinomycetales	Propionibacteriaceae	Propionibacterium	Propionibacterium acnes; #8261; AB042291
			Bacteria	Actinobacteria	Actinobacteria	Actinomycetales	Propionibacteriaceae	Propionibacterium	Propionibacterium acnes; ATCC6919; AB042288
			Bacteria	Actinobacteria	Actinobacteria	Actinomycetales	Propionibacteriaceae	Propionibacterium	Propionibacterium acnes; #8644; AB108477
			Bacteria	Actinobacteria	Actinobacteria	Actinomycetales	Propionibacteriaceae	Propionibacterium	Propionibacterium acnes; #5255; AB108482
			Bacteria	Actinobacteria	Actinobacteria	Actinomycetales	Propionibacteriaceae	Propionibacterium	Propionibacterium acnes; 0432; AB042286
			Bacteria	Actinobacteria	Actinobacteria	Actinomycetales	Propionibacteriaceae	Propionibacterium	Propionibacterium sp. MSP09A; AB084066
			Bacteria	Actinobacteria	Actinobacteria	Actinomycetales	Propionibacteriaceae	Propionibacterium	uncultured actinobacterium; S15A-MN99; AJ534680
			Bacteria	Actinobacteria	Actinobacteria	Actinomycetales	Propionibacteriaceae	Propionibacterium	Propionibacterium acnes; LIP4; Y12288
			Bacteria	Actinobacteria	Actinobacteria	Actinomycetales	Propionibacteriaceae	Propionibacterium	uncultured actinobacterium; S15B-MN4; AJ583195
			Bacteria	Actinobacteria	Actinobacteria	Actinomycetales	Propionibacteriaceae	Propionibacterium	Red Sea bacterium KT-2K38; AJ309527
			Bacteria	Actinobacteria	Actinobacteria	Actinomycetales	Propionibacteriaceae	Propionibacterium	Red Sea bacterium KT-2K12; AJ309523
o1522	o1522 16S-ProAcn2	GCTTTTGTGGGGTGCTCGA	Bacteria	Actinobacteria	Actinobacteria	Actinomycetales	Corynebacteriaceae	Corynebacterium	Corynebacterium atypicum (T); CCUG 45804; AJ441057
			Bacteria	Actinobacteria	Actinobacteria	Actinomycetales	Propionibacteriaceae	Propionibacterium	Propionibacterium acnes; #3004; AB041618
			Bacteria	Actinobacteria	Actinobacteria	Actinomycetales	Propionibacteriaceae	Propionibacterium	Propionibacterium acnes; #7168; AB042289
			Bacteria	Actinobacteria	Actinobacteria	Actinomycetales	Propionibacteriaceae	Propionibacterium	Propionibacterium acnes; #5317; AB108480
			Bacteria	Actinobacteria	Actinobacteria	Actinomycetales	Propionibacteriaceae	Propionibacterium	Propionibacterium acnes; #1003; AB108483
			Bacteria	Actinobacteria	Actinobacteria	Actinomycetales	Propionibacteriaceae	Propionibacterium	Propionibacterium acnes; #2929; AB108484
			Bacteria	Actinobacteria	Actinobacteria	Actinomycetales	Propionibacteriaceae	Propionibacterium	Propionibacterium acnes; #6119; AB108479
			Bacteria	Actinobacteria	Actinobacteria	Actinomycetales	Propionibacteriaceae	Propionibacterium	Propionibacterium acnes; #1447; AB041617
			Bacteria	Actinobacteria	Actinobacteria	Actinomycetales	Propionibacteriaceae	Propionibacterium	Propionibacterium acnes; #4584; AB042287
			Bacteria	Actinobacteria	Actinobacteria	Actinomycetales	Propionibacteriaceae	Propionibacterium	Propionibacterium acnes; #8261; AB042291
			Bacteria	Actinobacteria	Actinobacteria	Actinomycetales	Propionibacteriaceae	Propionibacterium	Propionibacterium acnes; #8644; AB108477
			Bacteria	Actinobacteria	Actinobacteria	Actinomycetales	Propionibacteriaceae	Propionibacterium	Propionibacterium acnes; #5255; AB108482
			Bacteria	Actinobacteria	Actinobacteria	Actinomycetales	Propionibacteriaceae	Propionibacterium	Propionibacterium acnes; 0432; AB042286
			Bacteria	Actinobacteria	Actinobacteria	Actinomycetales	Propionibacteriaceae	Propionibacterium	Propionibacterium acnes; #4860; AB108478
			Bacteria	Actinobacteria	Actinobacteria	Actinomycetales	Propionibacteriaceae	Propionibacterium	Propionibacterium sp. MSP09A; AB084066
			Bacteria	Actinobacteria	Actinobacteria	Actinomycetales	Propionibacteriaceae	Propionibacterium	uncultured actinobacterium; S15A-MN99; AJ534680
			Bacteria	Actinobacteria	Actinobacteria	Actinomycetales	Propionibacteriaceae	Propionibacterium	Propionibacterium acnes; LIP4; Y12288
			Bacteria	Actinobacteria	Actinobacteria	Actinomycetales	Propionibacteriaceae	Propionibacterium	uncultured actinobacterium; S15B-MN4; AJ583195
			Bacteria	Actinobacteria	Actinobacteria	Actinomycetales	Propionibacteriaceae	Propionibacterium	Red Sea bacterium KT-2K38; AJ309527
			Bacteria	Actinobacteria	Actinobacteria	Actinomycetales	Propionibacteriaceae	Propionibacterium	Red Sea bacterium KT-2K12; AJ309523
o1523	o1523 16S-ProOral	GTCGGTCCATAGGGTTGG	Bacteria	Proteobacteria	Betaproteobacteria	Rhodocyclales	Rhodocyclaceae	Rhodocyclus	Beta proteobacterium sp. oral strain A08KA; AY005031
o1525	o1525 16S-PropGroup2	TTGAGGAAGGTAGGGGAGAA	Bacteria	Actinobacteria	Actinobacteria	Actinomycetales	Propionibacteriaceae	Propionibacterium	Propionibacterium granulosum (T); DSM 20700; AJ003057
			Bacteria	Actinobacteria	Actinobacteria	Actinomycetales	Propionibacteriaceae	Propionibacterium	Propionibacterium propionicum (T); DSM 43307; AJ003058
			Bacteria	Actinobacteria	Actinobacteria	Actinomycetales	Propionibacteriaceae	Propionibacterium	Propionibacterium avidum (T); DSM 4901; AJ003055
			Bacteria	Actinobacteria	Actinobacteria	Actinomycetales	Propionibacteriaceae	Propionibacterium	Propionibacterium acnes; #5317; AB108480
			Bacteria	Actinobacteria	Actinobacteria	Actinomycetales	Propionibacteriaceae	Propionibacterium	Propionibacterium acnes; #1003; AB108483
			Bacteria	Actinobacteria	Actinobacteria	Actinomycetales	Propionibacteriaceae	Propionibacterium	Propionibacterium acnes; #2929; AB108484
			Bacteria	Actinobacteria	Actinobacteria	Actinomycetales	Propionibacteriaceae	Propionibacterium	Propionibacterium acnes; #6119; AB108479
			Bacteria	Actinobacteria	Actinobacteria	Actinomycetales	Propionibacteriaceae	Propionibacterium	Propionibacterium acnes; #8644; AB108477
			Bacteria	Actinobacteria	Actinobacteria	Actinomycetales	Propionibacteriaceae	Propionibacterium	Propionibacterium acnes; #5255; AB108482
			Bacteria	Actinobacteria	Actinobacteria	Actinomycetales	Propionibacteriaceae	Propionibacterium	Propionibacterium acnes; #4860; AB108478
			Bacteria	Actinobacteria	Actinobacteria	Actinomycetales	Propionibacteriaceae	Propionibacterium	Propionibacterium acnes; #8800; AB108481
			Bacteria	Actinobacteria	Actinobacteria	Actinomycetales	Propionibacteriaceae	Propionibacterium	Propionibacterium sp. V07/12348; Y17821
			Bacteria	Actinobacteria	Actinobacteria	Actinomycetales	Propionibacteriaceae	Propionibacterium	Propionibacterium sp. MSP09A; AB084066
			Bacteria	Actinobacteria	Actinobacteria	Actinomycetales	Propionibacteriaceae	Propionibacterium	uncultured actinobacterium; S15A-MN99; AJ534680
			Bacteria	Actinobacteria	Actinobacteria	Actinomycetales	Propionibacteriaceae	Propionibacterium	Propionibacterium acnes; LIP4; Y12288
			Bacteria	Actinobacteria	Actinobacteria	Actinomycetales	Propionibacteriaceae	Propionibacterium	uncultured actinobacterium; S15B-MN4; AJ583195
			Bacteria	Actinobacteria	Actinobacteria	Actinomycetales	Propionibacteriaceae	Propionibacterium	Red Sea bacterium KT-2K38; AJ309527
			Bacteria	Actinobacteria	Actinobacteria	Actinomycetales	Propionibacteriaceae	Propionibacterium	Red Sea bacterium KT-2K12; AJ309523
			Bacteria	Actinobacteria	Actinobacteria	Actinomycetales	Propionibacteriaceae	Propionibacterium	Propionibacteriaceae str. K4; AB078819
			Bacteria	Actinobacteria	Actinobacteria	Actinomycetales	Propionibacteriaceae	Propionibacterium	Propionibacterium jensenii; DSM 20535.; X53219
o1526	o1526 16S-PrSpe	TTGAATTGCAGATGAACGAT	Bacteria	Bacteroidetes	Bacteroidetes	Bacteroidales	Prevotellaceae	Prevotella	uncultured rumen bacterium; U29-F10; AB185797
			Bacteria	Bacteroidetes	Bacteroidetes	Bacteroidales	Prevotellaceae	Prevotella	uncultured rumen bacterium; U29-A10; AB185747
			Bacteria	Bacteroidetes	Bacteroidetes	Bacteroidales	Prevotellaceae	Prevotella	uncultured rumen bacterium 4C3d-6; AB034108
			Bacteria	Bacteroidetes	Bacteroidetes	Bacteroidales	Prevotellaceae	Prevotella	uncultured rumen bacterium; S11; DQ256291
			Bacteria	Bacteroidetes	Bacteroidetes	Bacteroidales	Prevotellaceae	Prevotella	uncultured rumen bacterium; P5_M08; EU382033
			Bacteria	Bacteroidetes	Bacteroidetes	Bacteroidales	Prevotellaceae	Prevotella	uncultured bacterium; rRNA106; AY958879
			Bacteria	Bacteroidetes	Bacteroidetes	Bacteroidales	Prevotellaceae	Prevotella	uncultured bacterium; rRNA362; AY959135
			Bacteria	Bacteroidetes	Bacteroidetes	Bacteroidales	Prevotellaceae	Prevotella	uncultured Prevotella sp.; 099B; EU644456

			Bacteria	Bacteroidetes	Bacteroidetes	Bacteroidales	Prevotellaceae	Prevotella	uncultured bacterium; V5_1_87_G11/19-1488; EF653423
			Bacteria	Bacteroidetes	Bacteroidetes	Bacteroidales	Prevotellaceae	Prevotella	Prevotella bivia (T); L16475
			Bacteria	Bacteroidetes	Bacteroidetes	Bacteroidales	Prevotellaceae	Prevotella	uncultured bacterium; nbw1031c12c1; GQ033319
			Bacteria	Bacteroidetes	Bacteroidetes	Bacteroidales	Prevotellaceae	Prevotella	uncultured bacterium; nbw1029d12c1; GQ031983
			Bacteria	Bacteroidetes	Bacteroidetes	Bacteroidales	Prevotellaceae	Prevotella	uncultured bacterium; nbw1029f09c1; GQ032001
			Bacteria	Bacteroidetes	Bacteroidetes	Bacteroidales	Prevotellaceae	Prevotella	uncultured bacterium; nbw1032a07c1; GQ033370
			Bacteria	Bacteroidetes	Bacteroidetes	Bacteroidales	Prevotellaceae	Prevotella	uncultured bacterium; nbw969b05c1; GQ043368
			Bacteria	Bacteroidetes	Bacteroidetes	Bacteroidales	Prevotellaceae	Prevotella	uncultured bacterium; nbw969h06c1; GQ043429
			Bacteria	Bacteroidetes	Bacteroidetes	Bacteroidales	Prevotellaceae	Prevotella	uncultured bacterium; nbw108c08c1; GQ007675
			Bacteria	Bacteroidetes	Bacteroidetes	Bacteroidales	Prevotellaceae	Prevotella	uncultured bacterium; nbw1011f08c1; GQ042976
			Bacteria	Bacteroidetes	Bacteroidetes	Bacteroidales	Prevotellaceae	Prevotella	uncultured bacterium; nbw970g09c1; GQ043753
			Bacteria	Bacteroidetes	Bacteroidetes	Bacteroidales	Prevotellaceae	Prevotella	uncultured bacterium; nbw1031h09c1; GQ033361
o1527	o1527 16S-PsGroup1	GCGACCACCTGGGCTAATA	Bacteria	Proteobacteria	Gammaproteobacteria	Pseudomonadales	Pseudomonadaceae	Pseudomonas	uncultured Pseudomonas sp.; 349_11; AF467304
			Bacteria	Proteobacteria	Gammaproteobacteria	Pseudomonadales	Pseudomonadaceae	Pseudomonas	uncultured bacterium; 010D-B08; AY661988
			Bacteria	Proteobacteria	Gammaproteobacteria	Pseudomonadales	Pseudomonadaceae	Pseudomonas	Pseudomonas sp.; RNA-111; AJ387903
			Bacteria	Proteobacteria	Gammaproteobacteria	Pseudomonadales	Pseudomonadaceae	Pseudomonas	Pseudomonas fragi; IAM 12402; D84014
			Bacteria	Proteobacteria	Gammaproteobacteria	Pseudomonadales	Pseudomonadaceae	Pseudomonas	uncultured bacterium; CS243; AY382612
			Bacteria	Proteobacteria	Gammaproteobacteria	Pseudomonadales	Pseudomonadaceae	Pseudomonas	Pseudomonas stutzeri; 11C2; marine sediment, Schillig; AJ270452
			Bacteria	Proteobacteria	Gammaproteobacteria	Pseudomonadales	Pseudomonadaceae	Pseudomonas	Pseudomonas stutzeri; phen8; AF284764
			Bacteria	Proteobacteria	Gammaproteobacteria	Pseudomonadales	Pseudomonadaceae	Pseudomonas	Pseudomonas stutzeri; 3C59; marine sediment, Dangast I; AJ270453
			Bacteria	Proteobacteria	Gammaproteobacteria	Pseudomonadales	Pseudomonadaceae	Pseudomonas	uncultured bacterium; IB-26; AJ488079
			Bacteria	Proteobacteria	Gammaproteobacteria	Pseudomonadales	Pseudomonadaceae	Pseudomonas	Pseudomonas stutzeri; JM-300, DSM 10701; X98607
			Bacteria	Proteobacteria	Gammaproteobacteria	Pseudomonadales	Pseudomonadaceae	Pseudomonas	Pseudomonas stutzeri; 24a13; soil contaminated with mineral oil; AJ270451
			Bacteria	Proteobacteria	Gammaproteobacteria	Pseudomonadales	Pseudomonadaceae	Pseudomonas	Pseudomonas stutzeri; 4C71; marine sediment, Dangast II; AJ270455
			Bacteria	Proteobacteria	Gammaproteobacteria	Pseudomonadales	Pseudomonadaceae	Pseudomonas	Pseudomonas stutzeri; 4C57; marine sediment, Dangast II; AJ270454
			Bacteria	Proteobacteria	Gammaproteobacteria	Pseudomonadales	Pseudomonadaceae	Pseudomonas	Pseudomonas sp. AS-33; AJ391194
			Bacteria	Proteobacteria	Gammaproteobacteria	Pseudomonadales	Pseudomonadaceae	Pseudomonas	uncultured Pseudomonas sp.; SR11d28; AF548761
			Bacteria	Proteobacteria	Gammaproteobacteria	Pseudomonadales	Pseudomonadaceae	Pseudomonas	Pseudomonas stutzeri; ATCC 17682; AJ006107
			Bacteria	Proteobacteria	Gammaproteobacteria	Pseudomonadales	Pseudomonadaceae	Pseudomonas	Pseudomonas sp.; 1F-16; X86622
			Bacteria	Proteobacteria	Gammaproteobacteria	Pseudomonadales	Pseudomonadaceae	Pseudomonas	Pseudomonas stutzeri; DSM 6084; AJ005167
			Bacteria	Proteobacteria	Gammaproteobacteria	Pseudomonadales	Pseudomonadaceae	Pseudomonas	uncultured gamma proteobacterium; Gitt-KF-169; AJ532695
			Bacteria	Proteobacteria	Gammaproteobacteria	Pseudomonadales	Pseudomonadaceae	Pseudomonas	Pseudomonas stutzeri; ATCC 17641; AJ006106
o1528	o1528 16S-PsGroup3	TGCCTGACTAGAGTACGGTA	Bacteria	Proteobacteria	Gammaproteobacteria	Pseudomonadales	Pseudomonadaceae	Pseudomonas	uncultured bacterium SJA-129; AJ009491
			Bacteria	Proteobacteria	Gammaproteobacteria	Pseudomonadales	Pseudomonadaceae	Pseudomonas	Pseudomonas citronellolis (T); DSM 50332T (type strain); Z76659
			Bacteria	Proteobacteria	Gammaproteobacteria	Pseudomonadales	Pseudomonadaceae	Pseudomonas	Pseudomonas sp. PAMU-1.9; AB118221
			Bacteria	Proteobacteria	Gammaproteobacteria	Pseudomonadales	Pseudomonadaceae	Pseudomonas	Pseudomonas sp. strain 1131; AY230195
			Bacteria	Proteobacteria	Gammaproteobacteria	Pseudomonadales	Pseudomonadaceae	Pseudomonas	Pseudomonas sp. KIE171-B; AJ311160
			Bacteria	Proteobacteria	Gammaproteobacteria	Pseudomonadales	Pseudomonadaceae	Pseudomonas	Pseudomonas sp. P400Y-1; AB076857
			Bacteria	Proteobacteria	Gammaproteobacteria	Pseudomonadales	Pseudomonadaceae	Pseudomonas	uncultured bacterium SJA-67; AJ009474
			Bacteria	Proteobacteria	Gammaproteobacteria	Pseudomonadales	Pseudomonadaceae	Pseudomonas	uncultured Pseudomonas sp.; ON3; AJ306834
			Bacteria	Proteobacteria	Gammaproteobacteria	Pseudomonadales	Pseudomonadaceae	Pseudomonas	Pseudomonas sp.; A3; Y13246
			Bacteria	Proteobacteria	Gammaproteobacteria	Pseudomonadales	Pseudomonadaceae	Pseudomonas	Pseudomonas nitroreducens; type strain:ATCC 700690; X96787
			Bacteria	Proteobacteria	Gammaproteobacteria	Pseudomonadales	Pseudomonadaceae	Pseudomonas	Pseudomonas citronellolis; ATCC 13674T; AB021396
			Bacteria	Proteobacteria	Gammaproteobacteria	Pseudomonadales	Pseudomonadaceae	Pseudomonas	Pseudomonas sp. YG-1; AF441203
			Bacteria	Proteobacteria	Gammaproteobacteria	Pseudomonadales	Pseudomonadaceae	Pseudomonas	Pseudomonas sp. 273; AF039488
			Bacteria	Proteobacteria	Gammaproteobacteria	Pseudomonadales	Pseudomonadaceae	Pseudomonas	Pseudomonas sp. AU2510; AY486376
			Bacteria	Proteobacteria	Gammaproteobacteria	Pseudomonadales	Pseudomonadaceae	Pseudomonas	Pseudomonas sp. AT2; AF208025
			Bacteria	Proteobacteria	Gammaproteobacteria	Pseudomonadales	Pseudomonadaceae	Pseudomonas	Pseudomonas sp. WAS2; AB007999
			Bacteria	Proteobacteria	Gammaproteobacteria	Pseudomonadales	Pseudomonadaceae	Pseudomonas	Pseudomonas sp. SOD-10b; AF448042
			Bacteria	Proteobacteria	Gammaproteobacteria	Pseudomonadales	Pseudomonadaceae	Pseudomonas	Pseudomonas sp. SOD-3; AF448043
			Bacteria	Proteobacteria	Gammaproteobacteria	Pseudomonadales	Pseudomonadaceae	Pseudomonas	Pseudomonas sp. strain 1087; AY230196
			Bacteria	Proteobacteria	Gammaproteobacteria	Pseudomonadales	Pseudomonadaceae	Pseudomonas	Pseudomonas nitroreducens; IAM1439; D84021
			Bacteria	Proteobacteria	Gammaproteobacteria	Xanthomonadales	Xanthomonadaceae	Schineria	Ignatzschineria larvae; L1/58; AJ252145
o1530	o1530 16S-PsGroup7	GCCTTGACATGCTGAGAAC	Bacteria	Proteobacteria	Gammaproteobacteria	Pseudomonadales	Pseudomonadaceae	Pseudomonas	Pseudomonas aeruginosa (T); DSM50071; X06684
			Bacteria	Proteobacteria	Gammaproteobacteria	Pseudomonadales	Pseudomonadaceae	Pseudomonas	Pseudomonas aeruginosa; AL98; AJ249451
			Bacteria	Proteobacteria	Gammaproteobacteria	Pseudomonadales	Pseudomonadaceae	Pseudomonas	Pseudomonas aeruginosa; #796; AB037553
			Bacteria	Proteobacteria	Gammaproteobacteria	Pseudomonadales	Pseudomonadaceae	Pseudomonas	Pseudomonas alcaligenes; IAM12411; D84006
			Bacteria	Proteobacteria	Gammaproteobacteria	Pseudomonadales	Pseudomonadaceae	Pseudomonas	Pseudomonas aeruginosa; #777; AB037549
			Bacteria	Proteobacteria	Gammaproteobacteria	Pseudomonadales	Pseudomonadaceae	Pseudomonas	Pseudomonas sp.; RNA-111; AJ387903
			Bacteria	Proteobacteria	Gammaproteobacteria	Pseudomonadales	Pseudomonadaceae	Pseudomonas	Pseudomonas aeruginosa; ATCC 27853; AB037545
			Bacteria	Proteobacteria	Gammaproteobacteria	Pseudomonadales	Pseudomonadaceae	Pseudomonas	Pseudomonas aeruginosa; #885; AB037555
			Bacteria	Proteobacteria	Gammaproteobacteria	Pseudomonadales	Pseudomonadaceae	Pseudomonas	Pseudomonas aeruginosa; H814; AB037563
			Bacteria	Proteobacteria	Gammaproteobacteria	Pseudomonadales	Pseudomonadaceae	Pseudomonas	Pseudomonas aeruginosa; 42A2 NCBI 40045; AJ309500
			Bacteria	Proteobacteria	Gammaproteobacteria	Pseudomonadales	Pseudomonadaceae	Pseudomonas	Pseudomonas alcaliphila (T); AL15-21; AB030583
			Bacteria	Proteobacteria	Gammaproteobacteria	Pseudomonadales	Pseudomonadaceae	Pseudomonas	Pseudomonas pseudoalcaligenes (T); DSM 50018T (type strain); Z76675
			Bacteria	Proteobacteria	Gammaproteobacteria	Pseudomonadales	Pseudomonadaceae	Pseudomonas	Pseudomonas pseudoalcaligenes; JCM 5968T; AB021379
			Bacteria	Proteobacteria	Gammaproteobacteria	Pseudomonadales	Pseudomonadaceae	Pseudomonas	Pseudomonas alcaligenes; LB19; AF390747
			Bacteria	Proteobacteria	Gammaproteobacteria	Pseudomonadales	Pseudomonadaceae	Pseudomonas	Pseudomonas sp.; LT1; AJ007005
			Bacteria	Proteobacteria	Gammaproteobacteria	Pseudomonadales	Pseudomonadaceae	Pseudomonas	Pseudomonas mendocina; PC1; AF232713
			Bacteria	Proteobacteria	Gammaproteobacteria	Pseudomonadales	Pseudomonadaceae	Pseudomonas	Pseudomonas resinovorans (T); LMG 2274T (type strain); Z76668
			Bacteria	Proteobacteria	Gammaproteobacteria	Pseudomonadales	Pseudomonadaceae	Pseudomonas	Pseudomonas aeruginosa; AF137358
			Bacteria	Proteobacteria	Gammaproteobacteria	Pseudomonadales	Pseudomonadaceae	Pseudomonas	Pseudomonas mendocina; ATCC 25413; AJ006109
			Bacteria	Proteobacteria	Gammaproteobacteria	Pseudomonadales	Pseudomonadaceae	Pseudomonas	Pseudomonas alcaligenes; ATCC 12815; AJ006110
o1531	o1531 16S-Psmon	GATGAGCCTAGGTCGGATTA	Bacteria	Proteobacteria	Gammaproteobacteria	Pseudomonadales	Pseudomonadaceae	Pseudomonas	Pseudomonas aeruginosa; #796; AB037553
			Bacteria	Proteobacteria	Gammaproteobacteria	Pseudomonadales	Pseudomonadaceae	Pseudomonas	Pseudomonas mucidolens; IAM12406; D84017
			Bacteria	Proteobacteria	Gammaproteobacteria	Pseudomonadales	Pseudomonadaceae	Pseudomonas	Pseudomonas aeruginosa; #885; AB037555
			Bacteria	Proteobacteria	Gammaproteobacteria	Pseudomonadales	Pseudomonadaceae	Pseudomonas	Pseudomonas aeruginosa; H814; AB037563
			Bacteria	Proteobacteria	Gammaproteobacteria	Pseudomonadales	Pseudomonadaceae	Pseudomonas	Pseudomonas fluorescens; IAM 12022; D84013
			Bacteria	Proteobacteria	Gammaproteobacteria	Pseudomonadales	Pseudomonadaceae	Pseudomonas	Pseudomonas chlororaphis subsp. chlororaphis; DSM 50083T (type strain); Z76673
			Bacteria	Proteobacteria	Gammaproteobacteria	Pseudomonadales	Pseudomonadaceae	Pseudomonas	Pseudomonas amygdali; LMG 2123T (type strain); Z76654
			Bacteria	Proteobacteria	Gammaproteobacteria	Pseudomonadales	Pseudomonadaceae	Pseudomonas	Pseudomonas pseudoalcaligenes; JCM 5968T; AB021379
			Bacteria	Proteobacteria	Gammaproteobacteria	Pseudomonadales	Pseudomonadaceae	Pseudomonas	Pseudomonas borealis; NB6; AJ012712
			Bacteria	Proteobacteria	Gammaproteobacteria	Pseudomonadales	Pseudomonadaceae	Pseudomonas	Pseudomonas alcaligenes; LB19; AF390747
			Bacteria	Proteobacteria	Gammaproteobacteria	Pseudomonadales	Pseudomonadaceae	Pseudomonas	Pseudomonas sp. Y1000; X99676
			Bacteria	Proteobacteria	Gammaproteobacteria	Pseudomonadales	Pseudomonadaceae	Pseudomonas	Pseudomonas mendocina; PC1; AF232713
			Bacteria	Proteobacteria	Gammaproteobacteria	Pseudomonadales	Pseudomonadaceae	Pseudomonas	Pseudomonas agarici (T); ATCC25941; D84005
			Bacteria	Proteobacteria	Gammaproteobacteria	Pseudomonadales	Pseudomonadaceae	Pseudomonas	Pseudomonas sp. LAB-36; AB051702
			Bacteria	Proteobacteria	Gammaproteobacteria	Pseudomonadales	Pseudomonadaceae	Pseudomonas	Pseudomonas sp. AS-33; AJ391194

			Bacteria	Proteobacteria	Gammaproteobacteria	Pseudomonadales	Pseudomonadaceae	Pseudomonas	Pseudomonas putida; P1; AJ271219
			Bacteria	Proteobacteria	Gammaproteobacteria	Pseudomonadales	Pseudomonadaceae	Pseudomonas	Pseudomonas mendocina; ATCC 25413; AJ006109
			Bacteria	Proteobacteria	Gammaproteobacteria	Pseudomonadales	Pseudomonadaceae	Pseudomonas	Pseudomonas fluorescens; type strain:ICMP 3512 = ATCC 13525 = NCIB 9046; AJ308
			Bacteria	Proteobacteria	Gammaproteobacteria	Pseudomonadales	Pseudomonadaceae	Pseudomonas	Pseudomonas sp. Circle; AJ417370
			Bacteria	Proteobacteria	Gammaproteobacteria	Pseudomonadales	Pseudomonadaceae	Pseudomonas	Pseudomonas sp.; GR-04; X86626
o1532	o1532 16S-RhoGroup	TGGGCAACCTAGTTGTTCAG	Bacteria	Proteobacteria	Alphaproteobacteria	Rhodospirillales	Rhodospirillaceae	Rhodovibrio	Rhodovibrio salinarum; JA281; FM177506
			Bacteria	Proteobacteria	Alphaproteobacteria	Rhodospirillales	Rhodospirillaceae	Rhodovibrio	uncultured alpha proteobacterium; HCM3MC90_5E_FL; EU373883
			Bacteria	Proteobacteria	Alphaproteobacteria	Rhodospirillales	Rhodospirillaceae	Rhodovibrio	uncultured alpha proteobacterium; HCM3MC83_6D_FL; EU373881
			Bacteria	Proteobacteria	Alphaproteobacteria	Rhodospirillales	Rhodospirillaceae	Rhodovibrio	Rhodovibrio salinarum; JA137; AM296500
			Bacteria	Proteobacteria	Alphaproteobacteria	Rhodospirillales	Rhodospirillaceae	Rhodovibrio	Rhodovibrio salinarum; NCIMB2243; D14432
			Bacteria	Proteobacteria	Alphaproteobacteria	Rhodospirillales	Rhodospirillaceae	Rhodovibrio	uncultured bacterium; 1.5.20; EU528254
			Bacteria	Proteobacteria	Alphaproteobacteria	Rhodospirillales	Rhodospirillaceae	Rhodovibrio	uncultured Rhodovibrio sp.; SFC1B101; AM981312
			Bacteria	Proteobacteria	Alphaproteobacteria	Rhodospirillales	Rhodospirillaceae	Rhodovibrio	Rhodovibrio salinarum (T); M59069
			Bacteria	Proteobacteria	Alphaproteobacteria	Rhodospirillales	Rhodospirillaceae	Rhodovibrio	Rhodovibrio sodomensis (T); DS1; M59072
			Bacteria	Proteobacteria	Alphaproteobacteria	Rhodospirillales	Rhodospirillaceae	Rhodovibrio	uncultured Rhodovibrio sp.; ntu9; EF153420
o1533	o1533 16S-RotGroup	AAAGTGACGGTAGGTGCAGA	Bacteria	Actinobacteria	Actinobacteria	Actinomycetales	Micrococcaceae	Rothia	Rothia dentocariosa; CV54; AJ717364
			Bacteria	Actinobacteria	Actinobacteria	Actinomycetales	Micrococcaceae	Rothia	Rothia sp. ChDC B201; AF543279
			Bacteria	Actinobacteria	Actinobacteria	Actinomycetales	Micrococcaceae	Rothia	Rothia sp. ChDC B202; AF543280
			Bacteria	Actinobacteria	Actinobacteria	Actinomycetales	Micrococcaceae	Rothia	Rothia dentocariosa; ChDC B198; AF543276
			Bacteria	Actinobacteria	Actinobacteria	Actinomycetales	Micrococcaceae	Rothia	Rothia dentocariosa; ChDC B199; AF543277
			Bacteria	Actinobacteria	Actinobacteria	Actinomycetales	Micrococcaceae	Rothia	Rothia dentocariosa; ChDC OS32; AF543284
			Bacteria	Actinobacteria	Actinobacteria	Actinomycetales	Micrococcaceae	Rothia	Rothia dentocariosa; Inje L1; AY594189
			Bacteria	Actinobacteria	Actinobacteria	Actinomycetales	Micrococcaceae	Rothia	Rothia dentocariosa; ChDC B200; AF543278
			Bacteria	Actinobacteria	Actinobacteria	Actinomycetales	Micrococcaceae	Rothia	Rothia sp. CCUG 25688; AJ131122
			Bacteria	Actinobacteria	Actinobacteria	Actinomycetales	Micrococcaceae	Rothia	uncultured bacterium; P4D1-563; EF509698
			Bacteria	Actinobacteria	Actinobacteria	Actinomycetales	Micrococcaceae	Rothia	Actinomyces sp.; CCUG 25688; Y13025
			Bacteria	Actinobacteria	Actinobacteria	Actinomycetales	Micrococcaceae	Kocuria	uncultured actinobacterium; F14_2A_FL; EF683007
			Bacteria	Actinobacteria	Actinobacteria	Actinomycetales	Micrococcaceae	Rothia	uncultured bacterium; P4D1-549; EF509711
			Bacteria	Actinobacteria	Actinobacteria	Actinomycetales	Micrococcaceae	Rothia	Rothia sp. Smarlab 3302411; AY538697
			Bacteria	Actinobacteria	Actinobacteria	Actinomycetales	Propionibacteriaceae	unclassified_Propionibacteriaceae	Propionibacteriaceae bacterium NML 02-0265; EF599122
			Bacteria	Actinobacteria	Actinobacteria	Actinomycetales	Micrococcaceae	Rothia	Rothia dentocariosa; ATCC BAA-907; CCUG 48652; CDC# C5660; AY546095
			Bacteria	Actinobacteria	Actinobacteria	Actinomycetales	Micrococcaceae	Rothia	Arthrobacter sp. B10; AF332382
			Bacteria	Actinobacteria	Actinobacteria	Actinomycetales	Micrococcaceae	Rothia	Rothia dentocariosa (T); ATCC 17931; M59055
			Bacteria	Actinobacteria	Actinobacteria	Actinomycetales	Micrococcaceae	Rothia	Rothia aeria (T); GTC867; AB071952
			Bacteria	Actinobacteria	Actinobacteria	Actinomycetales	Micrococcaceae	Rothia	uncultured bacterium; CD64; DQ441315
o1534	o1534 16S-RotMuc	ACCGCATCAGAGATGGTGTT	Bacteria	Actinobacteria	Actinobacteria	Actinomycetales	Micrococcaceae	Rothia	uncultured bacterium; A_S_01_77; FJ470589
			Bacteria	Actinobacteria	Actinobacteria	Actinomycetales	Micrococcaceae	Rothia	Rothia mucilaginosa; J04; DQ409140
			Bacteria	Actinobacteria	Actinobacteria	Actinomycetales	Micrococcaceae	Rothia	uncultured bacterium; FC03H03; FM873223
			Bacteria	Actinobacteria	Actinobacteria	Actinomycetales	Micrococcaceae	Rothia	Rothia mucilaginosa; DSM; X87758
			Bacteria	Actinobacteria	Actinobacteria	Actinomycetales	Micrococcaceae	Rothia	uncultured bacterium; P4D1-488; EF509689
			Bacteria	Actinobacteria	Actinobacteria	Actinomycetales	Micrococcaceae	Rothia	Rothia mucilaginosa (T); DSM type strain; X95483
			Bacteria	Actinobacteria	Actinobacteria	Actinomycetales	Micrococcaceae	Rothia	Rothia mucilaginosa; TeTT; FJ823142
			Bacteria	Actinobacteria	Actinobacteria	Actinomycetales	Micrococcaceae	Rothia	uncultured actinobacterium; S15D-MN233; AJ583197
			Bacteria	Actinobacteria	Actinobacteria	Actinomycetales	Micrococcaceae	Rothia	uncultured bacterium; nbt246h09; EU540197
			Bacteria	Actinobacteria	Actinobacteria	Actinomycetales	Micrococcaceae	Rothia	uncultured bacterium; nbt63g05; EU536611
			Bacteria	Actinobacteria	Actinobacteria	Actinomycetales	Micrococcaceae	Rothia	uncultured bacterium; nbw785g07c1; GQ010432
			Bacteria	Actinobacteria	Actinobacteria	Actinomycetales	Micrococcaceae	Rothia	uncultured bacterium; nbw106g08c1; GQ007574
			Bacteria	Actinobacteria	Actinobacteria	Actinomycetales	Micrococcaceae	Rothia	uncultured bacterium; nbw786d10c1; GQ010479
			Bacteria	Actinobacteria	Actinobacteria	Actinomycetales	Micrococcaceae	Rothia	uncultured bacterium; nbw786e12c1; GQ010491
			Bacteria	Actinobacteria	Actinobacteria	Actinomycetales	Micrococcaceae	Rothia	uncultured bacterium; Z360; AY979220
			Bacteria	Actinobacteria	Actinobacteria	Actinomycetales	Micrococcaceae	Rothia	uncultured bacterium; nbw112f11c1; GQ008032
			Bacteria	Actinobacteria	Actinobacteria	Actinomycetales	Micrococcaceae	Rothia	uncultured bacterium; nbw108a10c1; GQ007661
			Bacteria	Actinobacteria	Actinobacteria	Actinomycetales	Micrococcaceae	Rothia	uncultured bacterium; nbw111d10c1; GQ007933
			Bacteria	Actinobacteria	Actinobacteria	Actinomycetales	Micrococcaceae	Rothia	uncultured bacterium; nbw112b05c1; GQ007992
			Bacteria	Actinobacteria	Actinobacteria	Actinomycetales	Micrococcaceae	Rothia	uncultured bacterium; nbw108b04c1; GQ007664
o1535	o1535 16S-Rubac	AGGGCTAGAAACGTGCTACA	Bacteria	Actinobacteria	Actinobacteria	Rubrobacterales	Rubrobacteraceae	Rubrobacter	Rubrobacter radiotolerans; JCM 2153-T; U65647
			Bacteria	Actinobacteria	Actinobacteria	Rubrobacterales	Rubrobacteraceae	Rubrobacter	Rubrobacter radiotolerans; RALC-4; AJ243870
			Bacteria	Actinobacteria	Actinobacteria	Rubrobacterales	Rubrobacteraceae	Rubrobacter	Rubrobacter radiotolerans; type strain:DSM 5868; X98372
			Bacteria	Actinobacteria	Actinobacteria	Rubrobacterales	Rubrobacteraceae	Rubrobacter	uncultured soil bacterium; 1373; AY493979
			Bacteria	Actinobacteria	Actinobacteria	Rubrobacterales	Rubrobacteraceae	Rubrobacter	uncultured actinobacterium; FBP269; AY250873
			Bacteria	Actinobacteria	Actinobacteria	Rubrobacterales	Rubrobacteraceae	Rubrobacter	uncultured soil bacterium; 359; AY493980
			Bacteria	Actinobacteria	Actinobacteria	Rubrobacterales	Rubrobacteraceae	Rubrobacter	uncultured eubacterium Hb7-K4; AJ298579
			Bacteria	Actinobacteria	Actinobacteria	Rubrobacterales	Rubrobacteraceae	Rubrobacter	Rubrobacter sp. CBF L56; AB166956
			Bacteria	Actinobacteria	Actinobacteria	Rubrobacterales	Rubrobacteraceae	Rubrobacter	Rubrobacter taiwanensis; LS-286; AF479791
			Bacteria	Actinobacteria	Actinobacteria	Rubrobacterales	Rubrobacteraceae	Rubrobacter	uncultured eubacterium Hb7-K2; AJ298575
			Bacteria	Actinobacteria	Actinobacteria	Rubrobacterales	Rubrobacteraceae	Rubrobacter	uncultured eubacterium Hb7-K3; AJ298577
			Bacteria	Actinobacteria	Actinobacteria	Rubrobacterales	Rubrobacteraceae	Rubrobacter	uncultured eubacterium Hb7-K1; AJ298576
			Bacteria	Actinobacteria	Actinobacteria	Rubrobacterales	Rubrobacteraceae	Rubrobacter	Rubrobacter xylanophilus; RSPS-15; AJ243871
			Bacteria	Actinobacteria	Actinobacteria	Rubrobacterales	Rubrobacteraceae	Rubrobacter	Rubrobacter xylanophilus (T); PRD-1; X87135
			Bacteria	Actinobacteria	Actinobacteria	Rubrobacterales	Rubrobacteraceae	Rubrobacter	uncultured Rubrobacter sp.; 354H; AY571811
			Bacteria	Actinobacteria	Actinobacteria	Rubrobacterales	Rubrobacteraceae	Rubrobacter	uncultured bacterium #0319-7H2; AF234151
			Bacteria	Actinobacteria	Actinobacteria	Rubrobacterales	Rubrobacteraceae	Rubrobacter	Rubrobacter radiotolerans (T); DSM 46359-T; X87134
			Bacteria	Actinobacteria	Actinobacteria	Rubrobacterales	Rubrobacteraceae	Rubrobacter	uncultured actinobacterium; FBP483; AY250887
			Bacteria	Actinobacteria	Actinobacteria	Rubrobacterales	Rubrobacteraceae	Rubrobacter	uncultured bacterium; DRV-B001; AY923078
			Bacteria	Actinobacteria	Actinobacteria	Rubrobacterales	Rubrobacteraceae	Rubrobacter	uncultured bacterium; DRV-B035; AY923085
o1536	o1536 16S-RuGroup1	TTGGCAGGGCAGAGGCCT	Bacteria	Firmicutes	Clostridia	Clostridiales	Lachnospiraceae	Lachnospiraceae Incertae Sedis	Blautia schinkii (T); B; CIP 105464; DSM 10518; X94965
			Bacteria	Firmicutes	Clostridia	Clostridiales	Lachnospiraceae	Lachnospiraceae Incertae Sedis	Blautia schinkii; Bie 41; X94964
o1537	o1537 16S-RuGroup2	CAGCGATGCCGCGTGGA	Bacteria	Firmicutes	Clostridia	Clostridiales	Ruminococcaceae	Ruminococcus	Ruminococcus flavefaciens; JM1; AY349157
			Bacteria	unclassified_Bacteria					uncultured bacterium; oc34; AY491577
			Bacteria	Firmicutes	Clostridia	Clostridiales	Ruminococcaceae	Ruminococcus	uncultured bacterium; DGGE band A2; AF349415
			Bacteria	Firmicutes	Clostridia	Clostridiales	Ruminococcaceae	Ruminococcus	uncultured bacterium; DGGE band A29-1; AF349422
			Bacteria	Firmicutes	Clostridia	Clostridiales	Ruminococcaceae	Ruminococcus	Ruminococcus callidus; X85100
			Bacteria	Proteobacteria	unclassified_Proteobacteria				hydrothermal vent eubacterium PVB_OTU_4; PVB_13; U15116
			Bacteria	Firmicutes	Clostridia	Clostridiales	Ruminococcaceae	Ruminococcus	Ruminococcus flavefaciens; NJ; AF030446
			Bacteria	Firmicutes	Clostridia	Clostridiales	Ruminococcaceae	Ruminococcus	uncultured bacterium adhufec58; AF132277
			Bacteria	Proteobacteria	Epsilonproteobacteria	Nautiliales	Nautiliaceae	Caminibacter	uncultured bacterium S17sBac14; AF299124
			Bacteria	Firmicutes	Clostridia	Clostridiales	Ruminococcaceae	Ruminococcus	Ruminococcus flavefaciens; X85097
			Bacteria	Firmicutes	Clostridia	Clostridiales	Ruminococcaceae	Ruminococcus	uncultured bacterium; p-1293-a5; AF371743

			Bacteria	Proteobacteria	Epsilonproteobacteria	Nautiliales	Nautiliaceae	Caminibacter	uncultured bacterium S17sBac4; AF299129
			Bacteria	Firmicutes	Clostridia	Clostridiales	Ruminococcaceae	Ruminococcus	unidentified rumen bacterium RC37; AF001720
			Bacteria	Firmicutes	Clostridia	Clostridiales	Ruminococcaceae	Ruminococcus	Ruminococcus flavefaciens; NCFB 2213; X83430
			Bacteria	Firmicutes	Clostridia	Clostridiales	Ruminococcaceae	Ruminococcus	Ruminococcus sp. 25F7; AJ515917
			Bacteria	Firmicutes	Clostridia	Clostridiales	Ruminococcaceae	Ruminococcus	unidentified rumen bacterium RCP6; AF001730
			Bacteria	Firmicutes	Clostridia	Clostridiales	Ruminococcaceae	Ruminococcus	Ruminococcus sp. 18P13; AJ515913
			Bacteria	Firmicutes	Clostridia	Clostridiales	Ruminococcaceae	Ruminococcus	Ruminococcus sp. 25F6; AJ515916
			Bacteria	Firmicutes	Clostridia	Clostridiales	Ruminococcaceae	Ruminococcus	Ruminococcus sp. 25F8; AJ515915
			Bacteria	Firmicutes	Clostridia	Clostridiales	Ruminococcaceae	Ruminococcus	Ruminococcus sp. 7L75; AJ515914
o1538	o1538 16S-RuGroup3	AGCAAGTCTGATGTGAAAGG	Bacteria	Firmicutes	Clostridia	Clostridiales	Lachnospiraceae	Lachnospiraceae Incertae Sedis	uncultured bacterium; DGGE band A22; AF349420
			Bacteria	Firmicutes	Clostridia	Clostridiales	Lachnospiraceae	Lachnospiraceae Incertae Sedis	Ruminococcus sp. YE58; AY442822
			Bacteria	Firmicutes	Clostridia	Clostridiales	Lachnospiraceae	Lachnospiraceae Incertae Sedis	uncultured bacterium; Muc3-5; AY452001
			Bacteria	Firmicutes	Clostridia	Clostridiales	Lachnospiraceae	Lachnospiraceae Incertae Sedis	Ruminococcus obeum; 1-56; AY169424
			Bacteria	Firmicutes	Clostridia	Clostridiales	Lachnospiraceae	Lachnospiraceae Incertae Sedis	uncultured bacterium; Muc3-10; AY452003
			Bacteria	Firmicutes	Clostridia	Clostridiales	Lachnospiraceae	Lachnospiraceae Incertae Sedis	Ruminococcus obeum; 1-33; AY169419
			Bacteria	Firmicutes	Clostridia	Clostridiales	Lachnospiraceae	Lachnospiraceae Incertae Sedis	uncultured bacterium; from human colonic sample; HuCB25; AJ408999
			Bacteria	Firmicutes	Clostridia	Clostridiales	Lachnospiraceae	Lachnospiraceae Incertae Sedis	Clostridium sp.; formate; Y10584
			Bacteria	Firmicutes	Clostridia	Clostridiales	Lachnospiraceae	Lachnospiraceae Incertae Sedis	uncultured bacterium; p-2502-18B5; AF371540
			Bacteria	Firmicutes	Clostridia	Clostridiales	Lachnospiraceae	Lachnospiraceae Incertae Sedis	uncultured bacterium; from human colonic sample; HuCA22; AJ408974
			Bacteria	Firmicutes	Clostridia	Clostridiales	Lachnospiraceae	Lachnospiraceae Incertae Sedis	Blautia schinkii (T); B; CIP 105464; DSM 10518; X94965
			Bacteria	Firmicutes	Clostridia	Clostridiales	Lachnospiraceae	Lachnospiraceae Incertae Sedis	Blautia schinkii; Bie 41; X94964
			Bacteria	Firmicutes	Clostridia	Clostridiales	Lachnospiraceae	Lachnospiraceae Incertae Sedis	uncultured bacterium; PeH33; AJ576350
			Bacteria	Firmicutes	Clostridia	Clostridiales	Lachnospiraceae	Lachnospiraceae Incertae Sedis	Blautia producta; ATCC 27340; X94966
			Bacteria	Firmicutes	Clostridia	Clostridiales	Lachnospiraceae	Lachnospiraceae Incertae Sedis	Ruminococcus sp. M-1; AB125231
			Bacteria	Firmicutes	Clostridia	Clostridiales	Lachnospiraceae	Lachnospiraceae Incertae Sedis	Blautia producta; ATCC 27340; L76595
			Bacteria	Firmicutes	Clostridia	Clostridiales	Lachnospiraceae	Lachnospiraceae Incertae Sedis	uncultured bacterium; NL49; AY916245
			Bacteria	Firmicutes	Clostridia	Clostridiales	Lachnospiraceae	Lachnospiraceae Incertae Sedis	uncultured bacterium; V127; AY916274
			Bacteria	Firmicutes	Clostridia	Clostridiales	Lachnospiraceae	Lachnospiraceae Incertae Sedis	uncultured bacterium; NQ96; AY916255
			Bacteria	Firmicutes	Clostridia	Clostridiales	Lachnospiraceae	Lachnospiraceae Incertae Sedis	uncultured bacterium; KS62; AY916137
o1539	o1539 16S-RuObe	GACCAATCTCCGCATGGGG	Bacteria	Actinobacteria	Actinobacteria	Actinomycetales	Micrococcaceae	Rothia	uncultured actinobacterium; F12_1C_FL; EF683006
			Bacteria	Actinobacteria	Actinobacteria	Actinomycetales	Micrococcaceae	Rothia	Rothia mucilaginosa; J04; DQ409140
			Bacteria	Actinobacteria	Actinobacteria	Actinomycetales	Dermatophilaceae	Dermatophilus	Dermatophilus chelonae (T); DSM 44178 type strain; AJ243919
			Bacteria	Actinobacteria	Actinobacteria	Actinomycetales	Micrococcaceae	Rothia	Rothia dentocariosa; CV54; AJ717364
			Bacteria	Actinobacteria	Actinobacteria	Actinomycetales	Micrococcaceae	Rothia	uncultured bacterium; BF0002A031; AM696846
			Bacteria	Actinobacteria	Actinobacteria	Actinomycetales	Micrococcaceae	Rothia	Rothia dentocariosa; ChDC B198; AF543276
			Bacteria	Actinobacteria	Actinobacteria	Actinomycetales	Micrococcaceae	Rothia	Rothia dentocariosa; ChDC B199; AF543277
			Bacteria	Actinobacteria	Actinobacteria	Actinomycetales	Micrococcaceae	Rothia	Rothia dentocariosa; ChDC OS32; AF543284
			Bacteria	Actinobacteria	Actinobacteria	Actinomycetales	Micrococcaceae	Rothia	Rothia dentocariosa; Inje L1; AY594189
			Bacteria	Actinobacteria	Actinobacteria	Actinomycetales	Micrococcaceae	Rothia	Rothia dentocariosa; ChDC B200; AF543278
			Bacteria	Actinobacteria	Actinobacteria	Actinomycetales	Micrococcaceae	Rothia	Rothia mucilaginosa; DSM; X87758
			Bacteria	Actinobacteria	Actinobacteria	Actinomycetales	Micrococcaceae	Rothia	uncultured bacterium; P4D1-488; EF509689
			Bacteria	Actinobacteria	Actinobacteria	Actinomycetales	Micrococcaceae	Rothia	uncultured bacterium; P4D1-563; EF509698
			Bacteria	Actinobacteria	Actinobacteria	Actinomycetales	Micrococcaceae	Rothia	uncultured bacterium; P4D1-549; EF509711
			Bacteria	Actinobacteria	Actinobacteria	Actinomycetales	Micrococcaceae	Rothia	Rothia mucilaginosa (T); DSM type strain; X95483
			Bacteria	Actinobacteria	Actinobacteria	Actinomycetales	Micrococcaceae	Rothia	Rothia dentocariosa; ATCC BAA-907; CCUG 48652; CDC# C5660; AY546095
			Bacteria	Actinobacteria	Actinobacteria	Actinomycetales	Micrococcaceae	Rothia	Rothia dentocariosa (T); ATCC 17931; M59055
			Bacteria	Actinobacteria	Actinobacteria	Actinomycetales	Micrococcaceae	Rothia	uncultured actinobacterium; S15D-MN233; AJ583197
			Bacteria	Actinobacteria	Actinobacteria	Actinomycetales	Micrococcaceae	Rothia	uncultured bacterium; Z360; AY979220
			Bacteria	Actinobacteria	Actinobacteria	Actinomycetales	Micrococcaceae	Rothia	human oral bacterium C71; AF202004
o1542	o1542 16S-SelGroup2	AATACCGAATGAAGCGGAGG	Bacteria	Firmicutes	Clostridia	Clostridiales	Veillonellaceae	Selenomonas	uncultured Selenomonas sp.; DS071; AF366274
			Bacteria	Firmicutes	Clostridia	Clostridiales	Veillonellaceae	Selenomonas	Selenomonas sp. oral clone EW084; AF385503
			Bacteria	Firmicutes	Clostridia	Clostridiales	Veillonellaceae	Selenomonas	Selenomonas infelix (T); ATCC 43532; AF287802
			Bacteria	Firmicutes	Clostridia	Clostridiales	Veillonellaceae	Selenomonas	Selenomonas sp. oral clone EQ054; AF385495
			Bacteria	Firmicutes	Clostridia	Clostridiales	Veillonellaceae	Selenomonas	Selenomonas sp. oral clone EW051a; AF385497
			Bacteria	Firmicutes	Clostridia	Clostridiales	Veillonellaceae	Selenomonas	uncultured Selenomonas sp.; DWG09; AY672086
			Bacteria	Firmicutes	Clostridia	Clostridiales	Veillonellaceae	Selenomonas	uncultured Selenomonas sp.; 201B03(oral); AM420017
			Bacteria	Firmicutes	Clostridia	Clostridiales	Veillonellaceae	Selenomonas	uncultured Selenomonas sp.; 501B07(oral); AM420160
			Bacteria	Firmicutes	Clostridia	Clostridiales	Veillonellaceae	Selenomonas	uncultured Selenomonas sp.; 201D04(oral); AM420022
			Bacteria	Firmicutes	Clostridia	Clostridiales	Veillonellaceae	Selenomonas	uncultured Selenomonas sp.; 401G09(oral); AM420127
			Bacteria	Firmicutes	Clostridia	Clostridiales	Veillonellaceae	Selenomonas	uncultured Selenomonas sp.; 201F02(oral); AM420026
			Bacteria	Firmicutes	Clostridia	Clostridiales	Veillonellaceae	Selenomonas	uncultured Selenomonas sp.; 302A08(oral); AM420080
			Bacteria	Firmicutes	Clostridia	Clostridiales	Veillonellaceae	Selenomonas	Selenomonas sp. oral clone DY027; AF385492
			Bacteria	Firmicutes	Clostridia	Clostridiales	Veillonellaceae	Selenomonas	Selenomonas sp. oral clone EW076; AF385501
			Bacteria	Firmicutes	Clostridia	Clostridiales	Veillonellaceae	Selenomonas	uncultured bacterium; BF0001D022; AM697407
			Bacteria	Firmicutes	Clostridia	Clostridiales	Veillonellaceae	Selenomonas	Centipeda periodontii; HB-2; AF458222
			Bacteria	Firmicutes	Clostridia	Clostridiales	Veillonellaceae	Selenomonas	Selenomonas flueggei-like sp. clone AH132; AH032; AF287804
			Bacteria	Firmicutes	Clostridia	Clostridiales	Veillonellaceae	Selenomonas	Selenomonas sp. AIP 308-97; AY878647
			Bacteria	Firmicutes	Clostridia	Clostridiales	Veillonellaceae	Selenomonas	Selenomonas noxia; AIP 45-92; AY878646
			Bacteria	Firmicutes	Clostridia	Clostridiales	Veillonellaceae	Selenomonas	Selenomonas flueggei (T); ATCC 43531; AF287803
o1543	o1543 16S-SelGroup3	ACACGTACTACAATGGACG	Bacteria	Firmicutes	Clostridia	Clostridiales	Veillonellaceae	Selenomonas	Selenomonas sp. oral clone IQ048; AY349408
			Bacteria	Firmicutes	Clostridia	Clostridiales	Veillonellaceae	Selenomonas	Selenomonas sp. oral clone CS024; AF287796
			Bacteria	Firmicutes	Clostridia	Clostridiales	Veillonellaceae	Mitsuokella	uncultured bacterium; p-2400-55G5; AF371698
			Bacteria	Firmicutes	Clostridia	Clostridiales	Veillonellaceae	Selenomonas	Selenomonas noxia (T); ATCC 43541; AF287799
			Bacteria	Firmicutes	Clostridia	Clostridiales	Veillonellaceae	Centipeda	uncultured rumen bacterium; BS17; AY244971
			Bacteria	Firmicutes	Clostridia	Clostridiales	Veillonellaceae	unclassified_Veillonellaceae	Selenomonas genomosp. C2; C3MLM071; AY278628
			Bacteria	Firmicutes	Clostridia	Clostridiales	Veillonellaceae	Selenomonas	Centipeda periodontii; HB-2; AF458222
			Bacteria	Firmicutes	Clostridia	Clostridiales	Veillonellaceae	Selenomonas	Selenomonas noxia-like sp. oral clone CI002; AF287798
			Bacteria	Firmicutes	Clostridia	Clostridiales	Veillonellaceae	Selenomonas	Selenomonas-like sp. oral strain GAA14; AF287789
			Bacteria	Cyanobacteria	Cyanobacteria		Family VI	GpVI	uncultured cyanobacterium; SM1F09; AF445691
			Bacteria	Firmicutes	Clostridia	Clostridiales	Veillonellaceae	Centipeda	Centipeda periodontii (T); DSM 2778(T); AJ010963
			Bacteria	Firmicutes	Clostridia	Clostridiales	Veillonellaceae	Mitsuokella	bacterium mpn-isolate group 9; AF357557
			Bacteria	Cyanobacteria	Cyanobacteria		Family VI	GpVI	uncultured cyanobacterium; SM1D04; AF445673
			Bacteria	Cyanobacteria	Cyanobacteria		Family VI	GpVI	uncultured cyanobacterium; SM1C10; AF445667
			Bacteria	Proteobacteria	Gammaproteobacteria	unclassified_Gammaproteobacteria			uncultured bacterium; oc58; AY491599
			Bacteria	Firmicutes	Clostridia	Clostridiales	Veillonellaceae	Selenomonas	Selenomonas sp. oral clone AA024; AF287797
			Bacteria	Firmicutes	Clostridia	Clostridiales	Veillonellaceae	unclassified_Veillonellaceae	Selenomonas-like sp. oral strain FNA3; AF287792
			Bacteria	Firmicutes	Clostridia	Clostridiales	Veillonellaceae	Selenomonas	Selenomonas noxia; AIP 45-92; AY878646
			Bacteria	Firmicutes	Clostridia	Clostridiales	Veillonellaceae	Selenomonas	Selenomonas noxia; AIP 518-78; AY878645

			Bacteria	Planctomycetes	Planctomycetacia	Planctomycetales	Planctomycetaceae	Isosphaera	uncultured soil bacterium; 345-2; AY326528
o1544	o1544 16S-SelGroup4	TGAAGCGGAGGAGAGGCAT	Bacteria	Firmicutes	Clostridia	Clostridiales	Veillonellaceae	Selenomonas	uncultured Selenomonas sp.; DS071; AF366274
			Bacteria	Firmicutes	Clostridia	Clostridiales	Veillonellaceae	Selenomonas	Selenomonas sp. oral clone EW084; AF385503
			Bacteria	Firmicutes	Clostridia	Clostridiales	Veillonellaceae	Selenomonas	Selenomonas infelix (T); ATCC 43532; AF287802
			Bacteria	Firmicutes	Clostridia	Clostridiales	Veillonellaceae	Selenomonas	Selenomonas sp. oral clone EQ054; AF385495
			Bacteria	Firmicutes	Clostridia	Clostridiales	Veillonellaceae	Selenomonas	Selenomonas sp. oral clone EW051a; AF385497
			Bacteria	Firmicutes	Clostridia	Clostridiales	Veillonellaceae	Selenomonas	uncultured Selenomonas sp.; DWG09; AY672086
			Bacteria	Firmicutes	Clostridia	Clostridiales	Veillonellaceae	Selenomonas	uncultured Selenomonas sp.; 201B03(oral); AM420017
			Bacteria	Firmicutes	Clostridia	Clostridiales	Veillonellaceae	Selenomonas	uncultured Selenomonas sp.; 201D04(oral); AM420022
			Bacteria	Firmicutes	Clostridia	Clostridiales	Veillonellaceae	Selenomonas	uncultured Selenomonas sp.; 401G09(oral); AM420127
			Bacteria	Firmicutes	Clostridia	Clostridiales	Veillonellaceae	Selenomonas	uncultured Selenomonas sp.; 201F02(oral); AM420026
			Bacteria	Firmicutes	Clostridia	Clostridiales	Veillonellaceae	Selenomonas	uncultured Selenomonas sp.; 302A08(oral); AM420080
			Bacteria	Firmicutes	Clostridia	Clostridiales	Veillonellaceae	Selenomonas	Selenomonas sp. oral clone DY027; AF385492
			Bacteria	Firmicutes	Clostridia	Clostridiales	Veillonellaceae	Selenomonas	Selenomonas sp. oral clone EW076; AF385501
			Bacteria	Firmicutes	Clostridia	Clostridiales	Veillonellaceae	Selenomonas	uncultured bacterium; BF0001D022; AM697407
			Bacteria	Firmicutes	Clostridia	Clostridiales	Veillonellaceae	Selenomonas	Centipeda periodontii; HB-2; AF458222
			Bacteria	Firmicutes	Clostridia	Clostridiales	Veillonellaceae	Selenomonas	Selenomonas flueggei-like sp. clone AH132; AH032; AF287804
			Bacteria	Firmicutes	Clostridia	Clostridiales	Veillonellaceae	Selenomonas	Selenomonas dianae (T); ATCC 43527; AF287801
			Bacteria	Firmicutes	Clostridia	Clostridiales	Veillonellaceae	Selenomonas	Selenomonas sp. AIP 308-97; AY878647
			Bacteria	Firmicutes	Clostridia	Clostridiales	Veillonellaceae	Selenomonas	Selenomonas noxia; AIP 45-92; AY878646
			Bacteria	Firmicutes	Clostridia	Clostridiales	Veillonellaceae	Selenomonas	Selenomonas flueggei (T); ATCC 43531; AF287803
o1545	o1545 16S-SelNox	TAATGACGGTACCCATCGAG	Bacteria	Firmicutes	Clostridia	Clostridiales	Veillonellaceae	Selenomonas	Selenomonas sp. oral taxon 140; EL028; GQ422720
			Bacteria	Firmicutes	Clostridia	Clostridiales	Veillonellaceae	Selenomonas	uncultured Selenomonas sp.; 102D03(oral); AM419993
			Bacteria	Firmicutes	Clostridia	Clostridiales	Veillonellaceae	Selenomonas	uncultured bacterium; MA01C12; FM873606
			Bacteria	Firmicutes	Clostridia	Clostridiales	Veillonellaceae	Selenomonas	Selenomonas noxia (T); ATCC 43541; AF287799
			Bacteria	Firmicutes	Clostridia	Clostridiales	Veillonellaceae	Selenomonas	Selenomonas sp. oral clone OH4A; AY947498
o1546	o1546 16S-SelOral1	AAGATGGGTCAAACCATCGC	Bacteria	Firmicutes	Clostridia	Clostridiales	Veillonellaceae	Selenomonas	Selenomonas sp. AIP 308-97; AY878647
			Bacteria	Firmicutes	Clostridia	Clostridiales	Veillonellaceae	unclassified_Veillonellaceae	Selenomonas sp. oral clone EW079; AF385502
			Bacteria	Firmicutes	Clostridia	Clostridiales	Veillonellaceae	unclassified_Veillonellaceae	Selenomonas-like sp. oral clone DM071; AF287790
			Bacteria	Firmicutes	Clostridia	Clostridiales	Veillonellaceae	unclassified_Veillonellaceae	uncultured Selenomonas sp.; 303G10(oral); AM420113
			Bacteria	Firmicutes	Clostridia	Clostridiales	Veillonellaceae	unclassified_Veillonellaceae	uncultured Selenomonas sp.; 702E08(oral); AM420278
			Bacteria	Firmicutes	Clostridia	Clostridiales	Veillonellaceae	unclassified_Veillonellaceae	Selenomonas sp. oral clone EZ011; AF385578
			Bacteria	Firmicutes	Clostridia	Clostridiales	Veillonellaceae	unclassified_Veillonellaceae	uncultured Selenomonas sp.; 501C01(oral); AM420163
			Bacteria	Firmicutes	Clostridia	Clostridiales	Veillonellaceae	unclassified_Veillonellaceae	uncultured Selenomonas sp.; 301F12(oral); AM420074
			Bacteria	Firmicutes	Clostridia	Clostridiales	Veillonellaceae	Selenomonas	Veillonellaceae bacterium oral taxon 155; FYA47; GQ422724
			Bacteria	Firmicutes	Clostridia	Clostridiales	Veillonellaceae	unclassified_Veillonellaceae	Selenomonas genomosp. C2; C3MLM071; AY278628
			Bacteria	Firmicutes	Clostridia	Clostridiales	Veillonellaceae	Selenomonas	Selenomonas-like sp. oral strain GAA14; AF287789
			Bacteria	Firmicutes	Clostridia	Clostridiales	Veillonellaceae	Selenomonas	uncultured Selenomonas sp.; 17BA12; FJ976362
			Bacteria	Firmicutes	Clostridia	Clostridiales	Veillonellaceae	unclassified_Veillonellaceae	uncultured bacterium; 3P-3-1-G08; FJ562183
o1547	o1547 16S-SelOral2	AAAGATGGTGCAAGCCATCG	Bacteria	Firmicutes	Clostridia	Clostridiales	Veillonellaceae	unclassified_Veillonellaceae	Veillonellaceae bacterium oral taxon 129; FNA3; GQ422718
			Bacteria	Firmicutes	Clostridia	Clostridiales	Veillonellaceae	unclassified_Veillonellaceae	Veillonellaceae bacterium oral taxon 129; AU096; GQ422717
			Bacteria	Firmicutes	Clostridia	Clostridiales	Veillonellaceae	unclassified_Veillonellaceae	Veillonellaceae bacterium oral taxon 150; K024; GQ422721
			Bacteria	Firmicutes	Clostridia	Clostridiales	Veillonellaceae	unclassified_Veillonellaceae	Selenomonas-like sp. oral clone CS015; AF287791
			Bacteria	Firmicutes	Clostridia	Clostridiales	Veillonellaceae	unclassified_Veillonellaceae	Selenomonas sp. oral clone P2PA_80 P4; AY207052
			Bacteria	Firmicutes	Clostridia	Clostridiales	Veillonellaceae	unclassified_Veillonellaceae	uncultured bacterium; MA02G03; FM873686
			Bacteria	Firmicutes	Clostridia	Clostridiales	Veillonellaceae	unclassified_Veillonellaceae	uncultured Selenomonas sp.; 302C11(oral); AM420087
			Bacteria	Firmicutes	Clostridia	Clostridiales	Veillonellaceae	unclassified_Veillonellaceae	uncultured Selenomonas sp.; 101C02(oral); AM419959
			Bacteria	Firmicutes	Clostridia	Clostridiales	Veillonellaceae	unclassified_Veillonellaceae	Selenomonas-like sp. oral strain FNA3; AF287792
			Bacteria	Firmicutes	Clostridia	Clostridiales	Veillonellaceae	unclassified_Veillonellaceae	uncultured Selenomonas sp.; 7BB323; FJ976267
o1548	o1548 16S-SelOral3	ACGGTACCTTTTGAGGAAGC	Bacteria	Firmicutes	Clostridia	Clostridiales	Veillonellaceae	unclassified_Veillonellaceae	uncultured Selenomonas sp.; DS051; AF366273
			Bacteria	Firmicutes	Clostridia	Clostridiales	Veillonellaceae	Selenomonas	Selenomonas sp. oral clone CS024; AF287796
			Bacteria	Firmicutes	Clostridia	Clostridiales	Veillonellaceae	Selenomonas	Selenomonas artemidis; ATCC 43528; GQ422716
			Bacteria	Firmicutes	Clostridia	Clostridiales	Veillonellaceae	Selenomonas	Selenomonas sp. oral clone AA024; AF287797
			Bacteria	Firmicutes	Clostridia	Clostridiales	Veillonellaceae	Selenomonas	uncultured Selenomonas sp.; 12B680; FJ976341
			Bacteria	Firmicutes	Clostridia	Clostridiales	Veillonellaceae	Selenomonas	uncultured bacterium; nbu86d09c1; GQ006685
			Bacteria	Firmicutes	Clostridia	Clostridiales	Veillonellaceae	Selenomonas	uncultured bacterium; nbu87b09c1; GQ006744
			Bacteria	Firmicutes	Clostridia	Clostridiales	Veillonellaceae	Selenomonas	uncultured Selenomonas sp.; 17BB68; FJ976364
			Bacteria	Firmicutes	Clostridia	Clostridiales	Veillonellaceae	Selenomonas	uncultured bacterium; nbu87h08c1; GQ006802
			Bacteria	Firmicutes	Clostridia	Clostridiales	Veillonellaceae	Selenomonas	uncultured Selenomonas sp.; 6BB012; FJ976245
o1549	o1549 16S-SelOral4	ATACCGAATGAAGTGCAGGG	Bacteria	Firmicutes	Clostridia	Clostridiales	Veillonellaceae	Selenomonas	uncultured Selenomonas sp.; 201F04(oral); AM420028
			Bacteria	Firmicutes	Clostridia	Clostridiales	Veillonellaceae	Selenomonas	uncultured Selenomonas sp.; 502E06(oral); AM420194
			Bacteria	Firmicutes	Clostridia	Clostridiales	Veillonellaceae	Selenomonas	uncultured bacterium; A_D_02_83; FJ470480
			Bacteria	Firmicutes	Clostridia	Clostridiales	Veillonellaceae	Selenomonas	uncultured bacterium; BF0002D016; AM697505
			Bacteria	Firmicutes	Clostridia	Clostridiales	Veillonellaceae	Selenomonas	Selenomonas noxia-like sp. oral clone CI002; AF287798
			Bacteria	Firmicutes	Clostridia	Clostridiales	Veillonellaceae	Selenomonas	Selenomonas noxia; AIP 518-78; AY878645
			Bacteria	Firmicutes	Clostridia	Clostridiales	Veillonellaceae	Selenomonas	uncultured Selenomonas sp.; 7BB815; FJ976281
o1550	o1550 16S-SolGroup	AAACGGGGACTAATACCGGA	Bacteria	Firmicutes	Erysipelotrichi	Erysipelotrichales	Erysipelotrichaceae	Bulleidia	Bulleidia moorei; JCM 10645; RCA59-74; AB031056
			Bacteria	Firmicutes	Erysipelotrichi	Erysipelotrichales	Erysipelotrichaceae	Bulleidia	Bulleidia moorei; JCM 10647; RCA59-77; AB031058
			Bacteria	Firmicutes	Erysipelotrichi	Erysipelotrichales	Erysipelotrichaceae	Bulleidia	Bulleidia moorei; JCM 10646; RCA59-75; AB031057
			Bacteria	Firmicutes	Erysipelotrichi	Erysipelotrichales	Erysipelotrichaceae	Bulleidia	Bulleidia moorei; AHP 13983; AY044915
			Bacteria	Firmicutes	Erysipelotrichi	Erysipelotrichales	Erysipelotrichaceae	Bulleidia	Bulleidia moorei; JCM 10645; AY044916
			Bacteria	Firmicutes	Erysipelotrichi	Erysipelotrichales	Erysipelotrichaceae	Bulleidia	uncultured Bulleidia sp.; 102H01(oral); AM420006
			Bacteria	Firmicutes	Erysipelotrichi	Erysipelotrichales	Erysipelotrichaceae	Bulleidia	Bulleidia extructa (T); DSM 13220; AF220064
			Bacteria	Firmicutes	Erysipelotrichi	Erysipelotrichales	Erysipelotrichaceae	Bulleidia	uncultured bacterium; JPL2-3; DQ532150
			Bacteria	Firmicutes	Erysipelotrichi	Erysipelotrichales	Erysipelotrichaceae	Bulleidia	Bulleidia moorei; N407 0592; AM238669
			Bacteria	Actinobacteria	Actinobacteria	Actinomycetales	Streptomycetaceae	Streptomyces	Streptomyces sp. s5-201; EF012138
			Bacteria	Firmicutes	Erysipelotrichi	Erysipelotrichales	Erysipelotrichaceae	Bulleidia	Solobacterium sp. oral clone K010; AY005052
			Bacteria	Firmicutes	Erysipelotrichi	Erysipelotrichales	Erysipelotrichaceae	Bulleidia	uncultured bacterium; P3D1-639; EF509096
			Bacteria	Firmicutes	Erysipelotrichi	Erysipelotrichales	Erysipelotrichaceae	Bulleidia	uncultured Bulleidia sp.; 702B02(oral); AM420268
			Bacteria	Firmicutes	Erysipelotrichi	Erysipelotrichales	Erysipelotrichaceae	Bulleidia	uncultured bacterium; hoa46_29E04; EU748279
			Bacteria	Firmicutes	Erysipelotrichi	Erysipelotrichales	Erysipelotrichaceae	Bulleidia	uncultured bacterium; hoa12_15G02; EU748017
			Bacteria	Firmicutes	Erysipelotrichi	Erysipelotrichales	Erysipelotrichaceae	Bulleidia	uncultured bacterium; hoa12_60D08; EU344272
			Bacteria	Firmicutes	Erysipelotrichi	Erysipelotrichales	Erysipelotrichaceae	Bulleidia	uncultured bacterium; hoa2_42d08; EU344387
			Bacteria	Firmicutes	Erysipelotrichi	Erysipelotrichales	Erysipelotrichaceae	Bulleidia	uncultured bacterium; LG25; AY977912
			Bacteria	Firmicutes	Erysipelotrichi	Erysipelotrichales	Erysipelotrichaceae	Bulleidia	uncultured bacterium; EA10; DQ327446
			Bacteria	Firmicutes	Erysipelotrichi	Erysipelotrichales	Erysipelotrichaceae	Bulleidia	uncultured bacterium; hoa2_44d10; EU748120
o1551	o1551 16S-SolMoo	AAGCAACCTACCCACGAAGA	Bacteria	Firmicutes	Erysipelotrichi	Erysipelotrichales	Erysipelotrichaceae	Bulleidia	Bulleidia moorei; JCM 10645; RCA59-74; AB031056
			Bacteria	Firmicutes	Erysipelotrichi	Erysipelotrichales	Erysipelotrichaceae	Bulleidia	Bulleidia moorei; JCM 10647; RCA59-77; AB031058

			Bacteria	Firmicutes	Erysipelotrichi	Erysipelotrichales	Erysipelotrichaceae	Bulleidia	uncultured bacterium; A_S_01_01; FJ470569
			Bacteria	Firmicutes	Erysipelotrichi	Erysipelotrichales	Erysipelotrichaceae	Bulleidia	Bulleidia moorei; JCM 10646; RCA59-75; AB031057
			Bacteria	Firmicutes	Erysipelotrichi	Erysipelotrichales	Erysipelotrichaceae	Bulleidia	Bulleidia moorei; AHP 13983; AY044915
			Bacteria	Firmicutes	Erysipelotrichi	Erysipelotrichales	Erysipelotrichaceae	Bulleidia	Bulleidia moorei; JCM 10645; AY044916
			Bacteria	Firmicutes	Erysipelotrichi	Erysipelotrichales	Erysipelotrichaceae	Bulleidia	uncultured bacterium; JPL2-3; DQ532150
			Bacteria	Firmicutes	Erysipelotrichi	Erysipelotrichales	Erysipelotrichaceae	Bulleidia	Bulleidia moorei; N407 0592; AM238669
			Bacteria	Firmicutes	Erysipelotrichi	Erysipelotrichales	Erysipelotrichaceae	Bulleidia	uncultured bacterium; nbw1067h07c1; GQ057695
			Bacteria	Firmicutes	Erysipelotrichi	Erysipelotrichales	Erysipelotrichaceae	Bulleidia	uncultured bacterium; nbw895c01c1; GQ026675
			Bacteria	Firmicutes	Erysipelotrichi	Erysipelotrichales	Erysipelotrichaceae	Bulleidia	uncultured bacterium; LG25; AY977912
			Bacteria	Firmicutes	Erysipelotrichi	Erysipelotrichales	Erysipelotrichaceae	Bulleidia	uncultured bacterium; nbu86b09c1; GQ006662
			Bacteria	Firmicutes	Erysipelotrichi	Erysipelotrichales	Erysipelotrichaceae	Bulleidia	uncultured bacterium; nbw1067g12c1; GQ057693
			Bacteria	Firmicutes	Erysipelotrichi	Erysipelotrichales	Erysipelotrichaceae	Bulleidia	uncultured Bulleidia sp.; 3A78; FJ976235
			Bacteria	Firmicutes	Erysipelotrichi	Erysipelotrichales	Erysipelotrichaceae	Bulleidia	uncultured bacterium; EA10; DQ327446
			Bacteria	Firmicutes	Erysipelotrichi	Erysipelotrichales	Erysipelotrichaceae	Bulleidia	uncultured bacterium; nbw1066a05c1; GQ057603
			Bacteria	Firmicutes	Erysipelotrichi	Erysipelotrichales	Erysipelotrichaceae	Bulleidia	uncultured bacterium; nbw16b05c1; GQ061157
			Bacteria	Firmicutes	Erysipelotrichi	Erysipelotrichales	Erysipelotrichaceae	Bulleidia	uncultured bacterium; nbw1067g08c1; GQ057690
o1552	o1552 16S-SolOral	AACTCAGTGCTGCAGCTAAC	Bacteria	Firmicutes	Erysipelotrichi	Erysipelotrichales	Erysipelotrichaceae	Bulleidia	uncultured bacterium; A_S_01_01; FJ470569
			Bacteria	Firmicutes	Erysipelotrichi	Erysipelotrichales	Erysipelotrichaceae	Bulleidia	Bulleidia moorei; AHP 13983; AY044915
			Bacteria	Firmicutes	Erysipelotrichi	Erysipelotrichales	Erysipelotrichaceae	Bulleidia	Bulleidia moorei; JCM 10645; AY044916
			Bacteria	Firmicutes	Erysipelotrichi	Erysipelotrichales	Erysipelotrichaceae	Bulleidia	uncultured bacterium; JPL2-3; DQ532150
			Bacteria	Firmicutes	Erysipelotrichi	Erysipelotrichales	Erysipelotrichaceae	Bulleidia	Bulleidia moorei; N407 0592; AM238669
			Bacteria	Firmicutes	Erysipelotrichi	Erysipelotrichales	Erysipelotrichaceae	Holdemania	uncultured bacterium; COB P3-20; AY160823
			Bacteria	Firmicutes	Erysipelotrichi	Erysipelotrichales	Erysipelotrichaceae	Bulleidia	Solobacterium sp. oral clone K010; AY005052
			Bacteria	Firmicutes	Erysipelotrichi	Erysipelotrichales	Erysipelotrichaceae	Bulleidia	uncultured Bulleidia sp.; 702B02(oral); AM420268
			Bacteria	Firmicutes	Erysipelotrichi	Erysipelotrichales	Erysipelotrichaceae	Bulleidia	uncultured bacterium; 1103200831110; EU843530
			Bacteria	Firmicutes	Erysipelotrichi	Erysipelotrichales	Erysipelotrichaceae	Bulleidia	uncultured bacterium; 1103200823200; EU842452
			Bacteria	Firmicutes	Erysipelotrichi	Erysipelotrichales	Erysipelotrichaceae	Bulleidia	uncultured bacterium; 1103200827195; EU842699
			Bacteria	Firmicutes	Erysipelotrichi	Erysipelotrichales	Erysipelotrichaceae	Bulleidia	uncultured bacterium; nbw1067h07c1; GQ057695
			Bacteria	Firmicutes	Erysipelotrichi	Erysipelotrichales	Erysipelotrichaceae	Bulleidia	uncultured bacterium; nbw895c01c1; GQ026675
			Bacteria	Firmicutes	Erysipelotrichi	Erysipelotrichales	Erysipelotrichaceae	Bulleidia	uncultured bacterium; LG25; AY977912
			Bacteria	Firmicutes	Erysipelotrichi	Erysipelotrichales	Erysipelotrichaceae	Bulleidia	uncultured bacterium; nbu86b09c1; GQ006662
			Bacteria	Firmicutes	Erysipelotrichi	Erysipelotrichales	Erysipelotrichaceae	Bulleidia	uncultured bacterium; nbw1067g12c1; GQ057693
			Bacteria	Firmicutes	Erysipelotrichi	Erysipelotrichales	Erysipelotrichaceae	Bulleidia	uncultured Bulleidia sp.; 3A78; FJ976235
			Bacteria	Firmicutes	Erysipelotrichi	Erysipelotrichales	Erysipelotrichaceae	Bulleidia	uncultured bacterium; EA10; DQ327446
			Bacteria	Firmicutes	Erysipelotrichi	Erysipelotrichales	Erysipelotrichaceae	Bulleidia	uncultured bacterium; nbw1066a05c1; GQ057603
			Bacteria	Firmicutes	Erysipelotrichi	Erysipelotrichales	Erysipelotrichaceae	Bulleidia	uncultured bacterium; nbw16b05c1; GQ061157
o1553	o1553 16S-Spcha	AATAAGCCCCGGCTAATTAC	Bacteria	Spirochaetes	Spirochaetes	Spirochaetales	Spirochaetaceae	Spirochaeta	Spirochaeta aurantia; M1; AY599019
			Bacteria	Spirochaetes	Spirochaetes	Spirochaetales	Spirochaetaceae	Borrelia	Borrelia japonica (T); HO14; L40597
			Bacteria	Spirochaetes	Spirochaetes	Spirochaetales	Spirochaetaceae	Spirochaeta	uncultured spirochete; IE029; AY605133
			Bacteria	Spirochaetes	Spirochaetes	Spirochaetales	Spirochaetaceae	Spirochaeta	uncultured spirochete; IE015; AY605126
			Bacteria	Spirochaetes	Spirochaetes	Spirochaetales	Spirochaetaceae	Borrelia	Borrelia burgdorferi; CA2; AJ224136
			Bacteria	Spirochaetes	Spirochaetes	Spirochaetales	Spirochaetaceae	Borrelia	Borrelia burgdorferi; 25015; AJ224138
			Bacteria	Spirochaetes	Spirochaetes	Spirochaetales	Spirochaetaceae	Borrelia	Borrelia burgdorferi; CA55; AJ224140
			Bacteria	unclassified_Bacteria					candidate division OP4 clone OPB40; AF027047
			Bacteria	Spirochaetes	Spirochaetes	Spirochaetales	Spirochaetaceae	Borrelia	Borrelia burgdorferi; G2; M60967
			Bacteria	Spirochaetes	Spirochaetes	Spirochaetales	Spirochaetaceae	Spirochaeta	Spirochaetes bacterium pACH87; AY297804
			Bacteria	Spirochaetes	Spirochaetes	Spirochaetales	Spirochaetaceae	Spirochaeta	Spirochaeta alkalica (T); Z-7491; X93927
			Bacteria	Spirochaetes	Spirochaetes	Spirochaetales	Spirochaetaceae	Spirochaeta	Spirochaeta africana (T); Z-7692; X93928
			Bacteria	Spirochaetes	Spirochaetes	Spirochaetales	Spirochaetaceae	Borrelia	Borrelia burgdorferi; G1; M64311
			Bacteria	Spirochaetes	Spirochaetes	Spirochaetales	Spirochaetaceae	Spirochaeta	Spirochaeta asiatica (T); Z-7591; X93926
			Bacteria	Spirochaetes	Spirochaetes	Spirochaetales	Spirochaetaceae	Spirochaeta	Spirochaeta sp.; za29; AJ419823
			Bacteria	Spirochaetes	Spirochaetes	Spirochaetales	Spirochaetaceae	Borrelia	Borrelia garinii; isolate Fis01; AJ009751
			Bacteria	Spirochaetes	Spirochaetes	Spirochaetales	Spirochaetaceae	Borrelia	Borrelia garinii; isolate Far03; AJ009752
			Bacteria	Spirochaetes	Spirochaetes	Spirochaetales	Spirochaetaceae	Spirochaeta	Spirochaeta sp.; TM3; X97096
			Bacteria	Spirochaetes	Spirochaetes	Spirochaetales	Spirochaetaceae	Borrelia	Borrelia hermsii; MC77; AF228029
			Bacteria	Spirochaetes	Spirochaetes	Spirochaetales	Spirochaetaceae	Borrelia	Borrelia hermsii; SB82; AF228031
o1554	o1554 16S-StaGroup1	AGTCGAGCGAACAGATGAGA	Bacteria	Firmicutes	Bacilli	Bacillales	Staphylococcaceae	Staphylococcus	Staphylococcus sp. TUT1203; AB188210
			Bacteria	Firmicutes	Bacilli	Bacillales	Staphylococcaceae	Staphylococcus	Staphylococcus sciuri subsp. sciuri (T); DSM 20345T; AJ421446
			Bacteria	Firmicutes	Bacilli	Bacillales	Staphylococcaceae	Staphylococcus	Staphylococcus sciuri subsp. sciuri; S83569
			Bacteria	Firmicutes	Bacilli	Bacillales	Staphylococcaceae	Staphylococcus	Staphylococcus sp. ORG01; AY940424
			Bacteria	Firmicutes	Bacilli	Bacillales	Staphylococcaceae	Staphylococcus	Staphylococcus sciuri subsp. sciuri; AB212276
			Bacteria	Firmicutes	Bacilli	Bacillales	Staphylococcaceae	Staphylococcus	Staphylococcus sp. TM18_8; DQ279389
			Bacteria	Firmicutes	Bacilli	Bacillales	Staphylococcaceae	Staphylococcus	Staphylococcus lentus; NAB7; AY395014
			Bacteria	Firmicutes	Bacilli	Bacillales	Staphylococcaceae	Staphylococcus	Staphylococcus sp. CHNDP23; DQ337534
			Bacteria	Firmicutes	Bacilli	Bacillales	Staphylococcaceae	Staphylococcus	Staphylococcus vitulinus (T); ATCC 51698T; AB009942
			Bacteria	Firmicutes	Bacilli	Bacillales	Staphylococcaceae	Staphylococcus	Staphylococcus vitulinus (T); ATCC 51145T; AB009946
			Bacteria	Firmicutes	Bacilli	Bacillales	Staphylococcaceae	Staphylococcus	Staphylococcus sciuri subsp. sciuri; SCBM1; AY820254
			Bacteria	Firmicutes	Bacilli	Bacillales	Staphylococcaceae	Staphylococcus	Staphylococcus simiae (T); CCM 7213; AY727530
			Bacteria	Firmicutes	Bacilli	Bacillales	Staphylococcaceae	Staphylococcus	Staphylococcus simiae; CCM 7229; DQ127902
			Bacteria	Firmicutes	Bacilli	Bacillales	Staphylococcaceae	Staphylococcus	Staphylococcus lentus (T); ATCC 29070 (= MAFF 911385); D83370
			Bacteria	Firmicutes	Bacilli	Bacillales	Staphylococcaceae	Staphylococcus	Staphylococcus lentus; SSH39; AB219154
			Bacteria	Firmicutes	Bacilli	Bacillales	Staphylococcaceae	Staphylococcus	Staphylococcus sp. XJU-1; DQ837544
			Bacteria	Firmicutes	Bacilli	Bacillales	Staphylococcaceae	Staphylococcus	Staphylococcus sciuri subsp. sciuri; T131; AB116135
			Bacteria	Firmicutes	Bacilli	Bacillales	Staphylococcaceae	Staphylococcus	Staphylococcus fleurettii; GTC 1999; AB233330
			Bacteria	Firmicutes	Bacilli	Bacillales	Staphylococcaceae	Staphylococcus	Staphylococcus lentus; PLC-6; AY161045
			Bacteria	Firmicutes	Bacilli	Bacillales	Staphylococcaceae	Staphylococcus	Staphylococcus sciuri subsp. sciuri; S7; AF508104
o1555	o1555 16S-StaGroup3	GCCGCATTAGCTAGTTGGTA	Bacteria	Firmicutes	Bacilli	Bacillales	Staphylococcaceae	Staphylococcus	uncultured Staphylococcus sp.; Gexpansis25; AF467430
			Bacteria	Firmicutes	Bacilli	Bacillales	Staphylococcaceae	Staphylococcus	uncultured Staphylococcus sp.; Gexpansis24; AF467416
			Bacteria	Firmicutes	Bacilli	Bacillales	Staphylococcaceae	Staphylococcus	Staphylococcus epidermidis; M99; AY699287
			Bacteria	Firmicutes	Bacilli	Bacillales	Staphylococcaceae	Staphylococcus	bacterium Te66A; AY587794
			Bacteria	Firmicutes	Bacilli	Bacillales	Staphylococcaceae	Staphylococcus	uncultured bacterium; AT425_Eub41; AY053476
			Bacteria	Firmicutes	Bacilli	Bacillales	Staphylococcaceae	Staphylococcus	Staphylococcus sp. MO28; AY553115
			Bacteria	Firmicutes	Bacilli	Bacillales	Staphylococcaceae	Staphylococcus	Staphylococcus epidermidis; AB111112; C4; AF397060
			Bacteria	Firmicutes	Bacilli	Bacillales	Staphylococcaceae	Staphylococcus	Staphylococcus caprae (T); ATCC 35538T; AB009935
			Bacteria	Firmicutes	Bacilli	Bacillales	Staphylococcaceae	Staphylococcus	Staphylococcus capitis subsp. capitis (T); ATCC 49326T; AB009937
			Bacteria	Firmicutes	Bacilli	Bacillales	Staphylococcaceae	Staphylococcus	Staphylococcus sp.; Koll-2; AJ224939
			Bacteria	Firmicutes	Bacilli	Bacillales	Staphylococcaceae	Staphylococcus	Staphylococcus epidermidis; GH561; X75943
			Bacteria	Firmicutes	Bacilli	Bacillales	Staphylococcaceae	Staphylococcus	Staphylococcus caprae; DSM 20608; Y12593

			Bacteria	Firmicutes	Bacilli	Bacillales	Staphylococcaceae	Staphylococcus	Staphylococcus sp. YSS/2001-5; AY057450
			Bacteria	Firmicutes	Bacilli	Bacillales	Staphylococcaceae	Staphylococcus	Staphylococcus sp.; 1F-03; X86635
			Bacteria	Firmicutes	Bacilli	Bacillales	Staphylococcaceae	Staphylococcus	Staphylococcus epidermidis; AZ22L3; AY308046
			Bacteria	Firmicutes	Bacilli	Bacillales	Staphylococcaceae	Staphylococcus	uncultured bacterium; p-888-a5; AF371538
			Bacteria	Firmicutes	Bacilli	Bacillales	Staphylococcaceae	Staphylococcus	Staphylococcus epidermidis; GH 654; X75944
			Bacteria	Firmicutes	Bacilli	Bacillales	Staphylococcaceae	Staphylococcus	uncultured bacterium; Kazan-WB-01/BC19-WB-01; AY592221
			Bacteria	Firmicutes	Bacilli	Bacillales	Staphylococcaceae	Staphylococcus	uncultured bacterium; Kazan-WB-02/BC19-WB-02; AY592222
			Bacteria	Firmicutes	Bacilli	Bacillales	Staphylococcaceae	Staphylococcus	Staphylococcus capitis subsp. capitis; FO-036a; AF234853
o1556	o1556 16S-StaGroupHe	GGGAAGAACATACGTGTAAG	Bacteria	Firmicutes	Bacilli	Bacillales	Staphylococcaceae	Staphylococcus	Staphylococcus haemolyticus JCSC1435; AP006716
			Bacteria	Firmicutes	Bacilli	Bacillales	Staphylococcaceae	Staphylococcus	Staphylococcus haemolyticus JCSC1435; AP006716
			Bacteria	Firmicutes	Bacilli	Bacillales	Staphylococcaceae	Staphylococcus	Staphylococcus haemolyticus JCSC1435; AP006716
			Bacteria	Firmicutes	Bacilli	Bacillales	Staphylococcaceae	Staphylococcus	Staphylococcus haemolyticus JCSC1435; AP006716
			Bacteria	Firmicutes	Bacilli	Bacillales	Staphylococcaceae	Staphylococcus	Staphylococcus haemolyticus JCSC1435; AP006716
			Bacteria	Firmicutes	Bacilli	Bacillales	Staphylococcaceae	Staphylococcus	Staphylococcus haemolyticus; CCM2737; X66100
			Bacteria	Firmicutes	Bacilli	Bacillales	Staphylococcaceae	Staphylococcus	Staphylococcus croceolyticus; MCC10046; AY953148
			Bacteria	Firmicutes	Bacilli	Bacillales	Staphylococcaceae	Staphylococcus	uncultured bacterium; rRNA405; AY959178
			Bacteria	Firmicutes	Bacilli	Bacillales	Staphylococcaceae	Staphylococcus	uncultured bacterium; BF0001C088; AM697247
			Bacteria	Firmicutes	Bacilli	Bacillales	Staphylococcaceae	Staphylococcus	uncultured bacterium; BF0001C092; AM697251
			Bacteria	Firmicutes	Bacilli	Bacillales	Staphylococcaceae	Staphylococcus	Staphylococcus haemolyticus (T); ATCC 29970 (= MAFF 911476); D83367
			Bacteria	Firmicutes	Bacilli	Bacillales	Staphylococcaceae	Staphylococcus	uncultured bacterium; rRNA244; AY959017
			Bacteria	Firmicutes	Bacilli	Bacillales	Staphylococcaceae	Staphylococcus	Staphylococcus haemolyticus; L37600
			Bacteria	Firmicutes	Bacilli	Bacillales	Staphylococcaceae	Staphylococcus	Staphylococcus sp. GYZ; EF188282
			Bacteria	Firmicutes	Bacilli	Bacillales	Staphylococcaceae	Staphylococcus	human oral bacterium C20; AF202011
			Bacteria	Firmicutes	Bacilli	Bacillales	Staphylococcaceae	Staphylococcus	uncultured bacterium; MSFC_2M4H; DQ447795
			Bacteria	Firmicutes	Bacilli	Bacillales	Staphylococcaceae	Staphylococcus	Staphylococcus sp.; 1F-23; X86640
			Bacteria	Firmicutes	Bacilli	Bacillales	Staphylococcaceae	Staphylococcus	uncultured organism; MC061013; AY898006
			Bacteria	Firmicutes	Bacilli	Bacillales	Staphylococcaceae	Staphylococcus	Staphylococcus sp. NIPHL090904/B4; AY748913
			Bacteria	Firmicutes	Bacilli	Bacillales	Staphylococcaceae	Staphylococcus	bacterium 7RO1; AY928248
o1557	o1557 16S-StaGroupHo	ACTTATAGATGGACCTGCGC	Bacteria	Firmicutes	Bacilli	Bacillales	Staphylococcaceae	Staphylococcus	Staphylococcus hominis subsp. hominis; mother C3; 2C3; AM157418
			Bacteria	Firmicutes	Bacilli	Bacillales	Staphylococcaceae	Staphylococcus	Staphylococcus sp. H292; AB177642
			Bacteria	Firmicutes	Bacilli	Bacillales	Staphylococcaceae	Staphylococcus	Staphylococcus hominis subsp. hominis; DSM 20328; X66101
			Bacteria	Firmicutes	Bacilli	Bacillales	Staphylococcaceae	Staphylococcus	Staphylococcus sp. R-25050; AM084016
			Bacteria	Firmicutes	Bacilli	Bacillales	Staphylococcaceae	Staphylococcus	Staphylococcus hominis subsp. hominis; CV21; AJ717375
			Bacteria	Firmicutes	Bacilli	Bacillales	Staphylococcaceae	Staphylococcus	uncultured bacterium; PE08; AY838464
			Bacteria	Firmicutes	Bacilli	Bacillales	Staphylococcaceae	Staphylococcus	uncultured bacterium; PE14; AY838470
			Bacteria	Firmicutes	Bacilli	Bacillales	Staphylococcaceae	Staphylococcus	Staphylococcus sp. R-20811; AJ786779
			Bacteria	Firmicutes	Bacilli	Bacillales	Staphylococcaceae	Staphylococcus	Staphylococcus sp. LMG 21006; AJ316320
			Bacteria	Firmicutes	Bacilli	Bacillales	Staphylococcaceae	Staphylococcus	Staphylococcus sp. Smarlab 3301073; AY538683
			Bacteria	Firmicutes	Bacilli	Bacillales	Staphylococcaceae	Staphylococcus	Staphylococcus hominis subsp. hominis; JPL-5; AY030318
			Bacteria	Firmicutes	Bacilli	Bacillales	Staphylococcaceae	Staphylococcus	Staphylococcus sp. KCM-R4; AJ830708
			Bacteria	Firmicutes	Bacilli	Bacillales	Staphylococcaceae	Staphylococcus	Staphylococcus hominis subsp. hominis (T); L37601
			Bacteria	Firmicutes	Bacilli	Bacillales	Staphylococcaceae	Staphylococcus	Staphylococcus sp.; 1F-09; X86636
			Bacteria	Firmicutes	Bacilli	Bacillales	Staphylococcaceae	Staphylococcus	Staphylococcus sp. AU3605; AY486383
			Bacteria	Firmicutes	Bacilli	Bacillales	Staphylococcaceae	Staphylococcus	Staphylococcus sp.; S5-96; X86650
			Bacteria	Firmicutes	Bacilli	Bacillales	Staphylococcaceae	Staphylococcus	Staphylococcus sp. VM8; DQ238828
			Bacteria	Firmicutes	Bacilli	Bacillales	Staphylococcaceae	Staphylococcus	uncultured organism; MC0610c5; AY897999
			Bacteria	Firmicutes	Bacilli	Bacillales	Staphylococcaceae	Staphylococcus	uncultured organism; MC061010; AY898004
			Bacteria	Firmicutes	Bacilli	Bacillales	Staphylococcaceae	Staphylococcus	Staphylococcus hominis subsp. hominis; SAFN-038; AY167805
o1558	o1558 16S-StaLen	GGTTCAATGTTGAAAGACGG	Bacteria	Firmicutes	Bacilli	Bacillales	Staphylococcaceae	Staphylococcus	Staphylococcus lentus; NAB7; AY395014
			Bacteria	Firmicutes	Bacilli	Bacillales	Staphylococcaceae	Staphylococcus	Staphylococcus sp. CHNDP23; DQ337534
			Bacteria	Firmicutes	Bacilli	Bacillales	Staphylococcaceae	Staphylococcus	uncultured bacterium; aaa49b11; DQ817819
			Bacteria	Firmicutes	Bacilli	Bacillales	Staphylococcaceae	Staphylococcus	uncultured bacterium; aaa49b07; DQ817815
			Bacteria	Firmicutes	Bacilli	Bacillales	Staphylococcaceae	Staphylococcus	uncultured bacterium; aaa49a05; DQ817805
			Bacteria	Firmicutes	Bacilli	Bacillales	Staphylococcaceae	Staphylococcus	uncultured bacterium; aaa49a11; DQ817809
			Bacteria	Firmicutes	Bacilli	Bacillales	Staphylococcaceae	Staphylococcus	Staphylococcus lentus (T); ATCC 29070 (= MAFF 911385); D83370
			Bacteria	Firmicutes	Bacilli	Bacillales	Staphylococcaceae	Staphylococcus	uncultured bacterium; aaa49b02; DQ817812
			Bacteria	Firmicutes	Bacilli	Bacillales	Staphylococcaceae	Staphylococcus	uncultured bacterium; aaa49b09; DQ817817
			Bacteria	Firmicutes	Bacilli	Bacillales	Staphylococcaceae	Staphylococcus	Staphylococcus lentus; SSH39; AB219154
			Bacteria	Firmicutes	Bacilli	Bacillales	Staphylococcaceae	Staphylococcus	Staphylococcus lentus; SSCS5; AB210985
			Bacteria	Firmicutes	Bacilli	Bacillales	Staphylococcaceae	Staphylococcus	uncultured bacterium; aaa49a08; DQ817807
			Bacteria	Firmicutes	Bacilli	Bacillales	Staphylococcaceae	Staphylococcus	uncultured bacterium; aaa49a02; DQ817803
			Bacteria	Firmicutes	Bacilli	Bacillales	Staphylococcaceae	Staphylococcus	uncultured bacterium; aaa49b10; DQ817818
			Bacteria	Firmicutes	Bacilli	Bacillales	Staphylococcaceae	Staphylococcus	uncultured bacterium; aaa49b05; DQ817814
			Bacteria	Firmicutes	Bacilli	Bacillales	Staphylococcaceae	Staphylococcus	uncultured bacterium; aaa49a12; DQ817810
			Bacteria	Firmicutes	Bacilli	Bacillales	Staphylococcaceae	Staphylococcus	uncultured bacterium; aaa49b01; DQ817811
			Bacteria	Firmicutes	Bacilli	Bacillales	Staphylococcaceae	Staphylococcus	uncultured bacterium; aaa49a07; DQ817806
			Bacteria	Firmicutes	Bacilli	Bacillales	Staphylococcaceae	Staphylococcus	uncultured bacterium; aaa49a09; DQ817808
			Bacteria	Firmicutes	Bacilli	Bacillales	Staphylococcaceae	Staphylococcus	Staphylococcus lentus; PLC-6; AY161045
o1559	o1559 16S-StCon1	TGCATGGTAGATGTTTAAAAGG	Bacteria	Firmicutes	Bacilli	Lactobacillales	Streptococcaceae	Streptococcus	Streptococcus intermedius (T); ATCC27335; AF104671
			Bacteria	Firmicutes	Bacilli	Lactobacillales	Streptococcaceae	Streptococcus	Streptococcus constellatus subsp. constellatus; ATCC27823; AF104676
			Bacteria	Firmicutes	Bacilli	Lactobacillales	Streptococcaceae	Streptococcus	Streptococcus intermedius; 488; AF104673
			Bacteria	Firmicutes	Bacilli	Lactobacillales	Streptococcaceae	Streptococcus	Streptococcus intermedius; 535; AF104674
			Bacteria	Firmicutes	Bacilli	Lactobacillales	Streptococcaceae	Streptococcus	Streptococcus intermedius; 539; AF104675
			Bacteria	Firmicutes	Bacilli	Lactobacillales	Streptococcaceae	Streptococcus	Streptococcus constellatus subsp. constellatus; 1; AY277937
			Bacteria	Firmicutes	Bacilli	Lactobacillales	Streptococcaceae	Streptococcus	Streptococcus constellatus subsp. constellatus; 857; AY277938
			Bacteria	Firmicutes	Bacilli	Lactobacillales	Streptococcaceae	Streptococcus	Streptococcus constellatus subsp. constellatus; 1192; AY277940
			Bacteria	Firmicutes	Bacilli	Lactobacillales	Streptococcaceae	Streptococcus	Streptococcus constellatus subsp. constellatus; 919; AY277941
			Bacteria	Firmicutes	Bacilli	Lactobacillales	Streptococcaceae	Streptococcus	Streptococcus constellatus subsp. constellatus; 15; AY277939
			Bacteria	Firmicutes	Bacilli	Lactobacillales	Streptococcaceae	Streptococcus	uncultured bacterium; P3D5-550; EF508925
			Bacteria	Firmicutes	Bacilli	Lactobacillales	Streptococcaceae	Streptococcus	uncultured bacterium; P3D5-707; EF508972
			Bacteria	Firmicutes	Bacilli	Lactobacillales	Streptococcaceae	Streptococcus	Streptococcus constellatus subsp. constellatus; 206; AF104677
			Bacteria	Firmicutes	Bacilli	Lactobacillales	Streptococcaceae	Streptococcus	Streptococcus anginosus; CIP 105053; DQ232517
			Bacteria	Firmicutes	Bacilli	Lactobacillales	Streptococcaceae	Streptococcus	Streptococcus intermedius; CIP 105039; DQ232531
			Bacteria	Firmicutes	Bacilli	Lactobacillales	Streptococcaceae	Streptococcus	Streptococcus constellatus subsp. constellatus; CIP 105046; DQ232521
			Bacteria	Firmicutes	Bacilli	Lactobacillales	Streptococcaceae	Streptococcus	Streptococcus anginosus; CIP 105055; DQ232518
			Bacteria	Firmicutes	Bacilli	Lactobacillales	Streptococcaceae	Streptococcus	Streptococcus constellatus subsp. pharyngis; MM9889a; AY309095
			Bacteria	Firmicutes	Bacilli	Lactobacillales	Streptococcaceae	Streptococcus	Streptococcus constellatus subsp. constellatus; CIP 105032; DQ232520
			Bacteria	Firmicutes	Bacilli	Lactobacillales	Streptococcaceae	Streptococcus	Streptococcus intermedius; 125; AF104672

o1560	o1560 16S-StGroup4	ATAGTGAAAGGCGGCTTTGC	Bacteria	Firmicutes	Bacilli	Bacillales	Staphylococcaceae	Staphylococcus	Staphylococcus sp. es13; AJ704791
			Bacteria	Firmicutes	Bacilli	Bacillales	Staphylococcaceae	Staphylococcus	Staphylococcus sp. es1; AJ704792
			Bacteria	Firmicutes	Bacilli	Bacillales	Staphylococcaceae	Staphylococcus	Staphylococcus pasteuri; CV5; AJ717376
			Bacteria	Firmicutes	Bacilli	Bacillales	Staphylococcaceae	Staphylococcus	Staphylococcus sp. ARCTIC-P62; AY573029
			Bacteria	Firmicutes	Bacilli	Bacillales	Staphylococcaceae	Staphylococcus	Staphylococcus sp. LMG 19417; LMG-19417; AJ276810
			Bacteria	Firmicutes	Bacilli	Bacillales	Staphylococcaceae	Staphylococcus	unidentified bacterium; 2e; AJ223451
			Bacteria	Firmicutes	Bacilli	Bacillales	Staphylococcaceae	Staphylococcus	Staphylococcus sp. 24/1; AY578091
			Bacteria	Firmicutes	Bacilli	Bacillales	Staphylococcaceae	Staphylococcus	Staphylococcus warneri (T); L37603
			Bacteria	Firmicutes	Bacilli	Bacillales	Staphylococcaceae	Staphylococcus	Staphylococcus sp. AC7; DQ356497
			Bacteria	Firmicutes	Bacilli	Bacillales	Staphylococcaceae	Staphylococcus	Staphylococcus sp. R-20810; AJ786778
			Bacteria	Firmicutes	Bacilli	Bacillales	Staphylococcaceae	Staphylococcus	Staphylococcus sp. PF-F; DQ207366
			Bacteria	Firmicutes	Bacilli	Bacillales	Staphylococcaceae	Staphylococcus	Staphylococcus sp. AC10; DQ356498
			Bacteria	Firmicutes	Bacilli	Bacillales	Staphylococcaceae	Staphylococcus	uncultured bacterium; KNA6-NB24; AB179677
			Bacteria	Firmicutes	Bacilli	Bacillales	Staphylococcaceae	Staphylococcus	Staphylococcus sp. Am 14; AJ971853
			Bacteria	Firmicutes	Bacilli	Bacillales	Staphylococcaceae	Staphylococcus	uncultured organism; MC060412; AY897994
			Bacteria	Firmicutes	Bacilli	Bacillales	Staphylococcaceae	Staphylococcus	Staphylococcus sp. TM17_10; DQ279391
			Bacteria	Firmicutes	Bacilli	Bacillales	Staphylococcaceae	Staphylococcus	Staphylococcus sp. B60; AF333342
			Bacteria	Firmicutes	Bacilli	Bacillales	Staphylococcaceae	Staphylococcus	Staphylococcus sp. Ob 03; AJ971888
			Bacteria	Firmicutes	Bacilli	Bacillales	Staphylococcaceae	Staphylococcus	Staphylococcus sp. AC9; DQ356496
			Bacteria	Firmicutes	Bacilli	Bacillales	Staphylococcaceae	Staphylococcus	Staphylococcus sp. SOMBO56; AJ936959
o1561	o1561 16S-StMit1	TGAAGGAGGAGCTTGCTTCTCCG	Bacteria	Firmicutes	Bacilli	Lactobacillales	Streptococcaceae	Streptococcus	Streptococcus sp. oral clone ASCE12; AY923138
			Bacteria	Firmicutes	Bacilli	Lactobacillales	Streptococcaceae	Streptococcus	Streptococcus mitis; mother C4; 2C4; AM157428
			Bacteria	Firmicutes	Bacilli	Lactobacillales	Streptococcaceae	Streptococcus	Streptococcus mitis; mother V4; 2V4; AM157440
			Bacteria	Firmicutes	Bacilli	Lactobacillales	Streptococcaceae	Streptococcus	Streptococcus sp. oral clone GM006; AY349414
			Bacteria	Firmicutes	Bacilli	Lactobacillales	Streptococcaceae	Streptococcus	Streptococcus mitis (T); ATCC 49456; AF003929
			Bacteria	Firmicutes	Bacilli	Lactobacillales	Streptococcaceae	Streptococcus	Streptococcus sp. 3192A; AF316596
			Bacteria	Firmicutes	Bacilli	Lactobacillales	Streptococcaceae	Streptococcus	uncultured bacterium; BF0002C069; AM697372
			Bacteria	Firmicutes	Bacilli	Lactobacillales	Streptococcaceae	Streptococcus	uncultured bacterium; FS275-1B-03; DQ513103
			Bacteria	Firmicutes	Bacilli	Lactobacillales	Streptococcaceae	Streptococcus	uncultured bacterium; BF0001C072; AM697231
			Bacteria	Firmicutes	Bacilli	Lactobacillales	Streptococcaceae	Streptococcus	uncultured bacterium; BF0002A057; AM696872
			Bacteria	Firmicutes	Bacilli	Lactobacillales	Streptococcaceae	Streptococcus	uncultured bacterium; BF0002A063; AM696878
			Bacteria	Firmicutes	Bacilli	Lactobacillales	Streptococcaceae	Streptococcus	Streptococcus mitis; ATCC 49456; GCSS 1303; AY485601
			Bacteria	Firmicutes	Bacilli	Lactobacillales	Streptococcaceae	Streptococcus	Streptococcus genomosp. C5; C4AKM023; AY278633
			Bacteria	Firmicutes	Bacilli	Lactobacillales	Streptococcaceae	Streptococcus	Streptococcus mitis; ATCC 49456; DQ303188
			Bacteria	Firmicutes	Bacilli	Lactobacillales	Streptococcaceae	Streptococcus	Streptococcus oralis; ATCC 700233; AY281080
			Bacteria	Firmicutes	Bacilli	Lactobacillales	Streptococcaceae	Streptococcus	Streptococcus pneumoniae; SI_P25; AY525793
			Bacteria	Firmicutes	Bacilli	Lactobacillales	Streptococcaceae	Streptococcus	Streptococcus mitis; NCTC12261; D38482
			Bacteria	Firmicutes	Bacilli	Lactobacillales	Streptococcaceae	Streptococcus	Streptococcus pneumoniae; SI_P7; AY525792
			Bacteria	Firmicutes	Bacilli	Lactobacillales	Streptococcaceae	Streptococcus	uncultured organism; MC061015; AY898008
			Bacteria	Firmicutes	Bacilli	Lactobacillales	Streptococcaceae	Streptococcus	Streptococcus sp. 10aVMg3; EF151147
o1562	o1562 16S-Stmitbv2	GCATGATAACTGTTTGAAAGGT	Bacteria	Firmicutes	Bacilli	Lactobacillales	Streptococcaceae	Streptococcus	uncultured Streptococcus sp.; 401H08(oral); AM420131
			Bacteria	Firmicutes	Bacilli	Lactobacillales	Streptococcaceae	Streptococcus	uncultured Streptococcus sp.; 601F08(oral); AM420224
			Bacteria	Firmicutes	Bacilli	Lactobacillales	Streptococcaceae	Streptococcus	uncultured bacterium; MA03D04; FM873712
			Bacteria	Firmicutes	Bacilli	Lactobacillales	Streptococcaceae	Streptococcus	Streptococcus sp. oral clone P2PA_41 P2; AY207051
			Bacteria	Firmicutes	Bacilli	Lactobacillales	Streptococcaceae	Streptococcus	uncultured bacterium; P4D1-706; EF509765
			Bacteria	Firmicutes	Bacilli	Lactobacillales	Streptococcaceae	Streptococcus	Streptococcus mitis bv2; SK34; AY005045
			Bacteria	Firmicutes	Bacilli	Lactobacillales	Streptococcaceae	Streptococcus	uncultured bacterium; nbw951c07c1; GQ040198
			Bacteria	Firmicutes	Bacilli	Lactobacillales	Streptococcaceae	Streptococcus	uncultured bacterium; nbw899f11c1; GQ026845
			Bacteria	Firmicutes	Bacilli	Lactobacillales	Streptococcaceae	Streptococcus	uncultured bacterium; nbw786c01c1; GQ010462
			Bacteria	Firmicutes	Bacilli	Lactobacillales	Streptococcaceae	Streptococcus	uncultured bacterium; nbw900b09c1; GQ026876
			Bacteria	Firmicutes	Bacilli	Lactobacillales	Streptococcaceae	Streptococcus	uncultured bacterium; nbw949a10c1; GQ040008
			Bacteria	Firmicutes	Bacilli	Lactobacillales	Streptococcaceae	Streptococcus	uncultured bacterium; nbw785d12c1; GQ010409
			Bacteria	Firmicutes	Bacilli	Lactobacillales	Streptococcaceae	Streptococcus	uncultured bacterium; nbw898e02c1; GQ032113
			Bacteria	Firmicutes	Bacilli	Lactobacillales	Streptococcaceae	Streptococcus	uncultured bacterium; nbw951a08c1; GQ040179
			Bacteria	Firmicutes	Bacilli	Lactobacillales	Streptococcaceae	Streptococcus	endosymbiont of Sphenophorus levis; L_E04full; FJ626265
			Bacteria	Firmicutes	Bacilli	Lactobacillales	Streptococcaceae	Streptococcus	uncultured bacterium; nbw900b01c1; GQ026868
			Bacteria	Firmicutes	Bacilli	Lactobacillales	Streptococcaceae	Streptococcus	uncultured bacterium; nbw950d02c1; GQ040121
			Bacteria	Firmicutes	Bacilli	Lactobacillales	Streptococcaceae	Streptococcus	Streptococcus sp. PSH2; AB038371
			Bacteria	Firmicutes	Bacilli	Lactobacillales	Streptococcaceae	Streptococcus	Streptococcus oralis; CCRI-15017; EU156769
			Bacteria	Firmicutes	Bacilli	Lactobacillales	Streptococcaceae	Streptococcus	Streptococcus sp. 11aTha1; EF151150
o1563	o1563 16S-Stmut1	ATATTAATTATTGCATGATAATTGAT	Bacteria	Firmicutes	Bacilli	Lactobacillales	Streptococcaceae	Streptococcus	Streptococcus mutans; UA96; AF139600
			Bacteria	Firmicutes	Bacilli	Lactobacillales	Streptococcaceae	Streptococcus	Streptococcus mutans; UA140; AF139599
			Bacteria	Firmicutes	Bacilli	Lactobacillales	Streptococcaceae	Streptococcus	Streptococcus mutans; ATCC 25175; AY188348
			Bacteria	Firmicutes	Bacilli	Lactobacillales	Streptococcaceae	Streptococcus	Streptococcus mutans; ChDC YM9; AY691527
			Bacteria	Firmicutes	Bacilli	Lactobacillales	Streptococcaceae	Streptococcus	Streptococcus mutans; ChDC YM71; AY691528
			Bacteria	Firmicutes	Bacilli	Lactobacillales	Streptococcaceae	Streptococcus	Streptococcus mutans; ChDC YM88; AY691531
			Bacteria	Firmicutes	Bacilli	Lactobacillales	Streptococcaceae	Streptococcus	Streptococcus mutans; ChDC YM3; AY691526
			Bacteria	Firmicutes	Bacilli	Lactobacillales	Streptococcaceae	Streptococcus	Streptococcus mutans; ChDC YM97; AY691532
			Bacteria	Firmicutes	Bacilli	Lactobacillales	Streptococcaceae	Streptococcus	Streptococcus mutans; ChDC YM83; AY691529
			Bacteria	Firmicutes	Bacilli	Lactobacillales	Streptococcaceae	Streptococcus	Streptococcus mutans; NCTC 10449; AJ243965
			Bacteria	Firmicutes	Bacilli	Lactobacillales	Streptococcaceae	Streptococcus	Streptococcus mutans; ChDC YM2; AY691525
			Bacteria	Firmicutes	Bacilli	Lactobacillales	Streptococcaceae	Streptococcus	uncultured bacterium; PE07; AY838463
			Bacteria	Firmicutes	Bacilli	Lactobacillales	Streptococcaceae	Streptococcus	uncultured bacterium; PE04; AY838460
			Bacteria	Firmicutes	Bacilli	Lactobacillales	Streptococcaceae	Streptococcus	uncultured bacterium; PE09; AY838465
			Bacteria	Firmicutes	Bacilli	Lactobacillales	Streptococcaceae	Streptococcus	uncultured bacterium; PE11; AY838467
			Bacteria	Firmicutes	Bacilli	Lactobacillales	Streptococcaceae	Streptococcus	uncultured bacterium; PE19; AY838475
			Bacteria	Firmicutes	Bacilli	Lactobacillales	Streptococcaceae	Streptococcus	Streptococcus mutans; UA96; AF077835
			Bacteria	Firmicutes	Bacilli	Lactobacillales	Streptococcaceae	Streptococcus	Streptococcus mutans; UA140; AF077836
			Bacteria	Firmicutes	Bacilli	Lactobacillales	Streptococcaceae	Streptococcus	Streptococcus gordonii; DL1; AJ427479
			Bacteria	Firmicutes	Bacilli	Lactobacillales	Streptococcaceae	Streptococcus	Streptococcus gordonii; ATCC 12396; AY281088
o1564	o1564 16S-Stmyc	GGGAGATCGGAATTCCTGG	Bacteria	Actinobacteria	Actinobacteria	Actinomycetales	Streptomycetaceae	Streptomyces	Streptomyces griseus; 2247; AB030567
			Bacteria	Actinobacteria	Actinobacteria	Actinomycetales	Streptomycetaceae	Streptomyces	Streptomyces griseus; 2247; AB030568
			Bacteria	Actinobacteria	Actinobacteria	Actinomycetales	Streptomycetaceae	Streptomyces	Streptomyces griseus; 2247; AB030572
			Bacteria	Actinobacteria	Actinobacteria	Actinomycetales	Streptomycetaceae	Streptomyces	Streptomycetaceae; SR 179c; X95470
			Bacteria	Actinobacteria	Actinobacteria	Actinomycetales	Streptomycetaceae	Streptomyces	Streptomyces tumescens; Km-2-1; AF346483
			Bacteria	Actinobacteria	Actinobacteria	Actinomycetales	Streptomycetaceae	Streptomyces	Streptomycetaceae; SR 257; X87316
			Bacteria	Actinobacteria	Actinobacteria	Actinomycetales	Streptomycetaceae	Streptomyces	Streptomyces kasugaensis (T); M338-M1; AB024441
			Bacteria	Actinobacteria	Actinobacteria	Actinomycetales	Streptomycetaceae	Streptomyces	Actinomyces sp.; SR 25; X95471

			Bacteria	Actinobacteria	Actinobacteria	Actinomycetales	Streptomycetaceae	Streptomyces	Streptomyces somaliensis; DSM 40760; AJ007399
			Bacteria	Actinobacteria	Actinobacteria	Actinomycetales	Streptomycetaceae	Streptomyces	Streptomyces somaliensis (T); DSM 40738; AJ007403
			Bacteria	Actinobacteria	Actinobacteria	Actinomycetales	Streptomycetaceae	Streptomyces	Streptomyces tumescens; Km-1-1; AF346482
			Bacteria	Actinobacteria	Actinobacteria	Actinomycetales	Streptomycetaceae	Streptomyces	Streptomyces cattleya; JCM 4925; AB045871
			Bacteria	Actinobacteria	Actinobacteria	Actinomycetales	Streptomycetaceae	Streptomyces	Streptomyces griseus; ATCC10137; Y15501
			Bacteria	Actinobacteria	Actinobacteria	Actinomycetales	Streptomycetaceae	Streptomyces	Streptomyces sp.; CFBP4509; AJ007424
			Bacteria	Actinobacteria	Actinobacteria	Actinomycetales	Streptomycetaceae	Streptomyces	Streptomyces sp. 11AG8; AF233375
			Bacteria	Actinobacteria	Actinobacteria	Actinomycetales	Streptomycetaceae	Streptomyces	Streptomyces sp. C9P3; C9P3 (S4); AJ391828
			Bacteria	Actinobacteria	Actinobacteria	Actinomycetales	Streptomycetaceae	Streptomyces	Streptomyces albidoflavus; DSM 40792; Z76677
			Bacteria	Actinobacteria	Actinobacteria	Actinomycetales	Streptomycetaceae	Streptomyces	Streptomyces odorifer (T); DSM 40347T; Z76682
			Bacteria	Actinobacteria	Actinobacteria	Actinomycetales	Streptomycetaceae	Streptomyces	Streptomyces lavendulae subsp. lavendulae; IFO 12343; D85111
			Bacteria	Actinobacteria	Actinobacteria	Actinomycetales	Streptomycetaceae	Streptomyces	Streptomyces arenae (T); ISP 5293; AJ399485
o1566	o1566 16S-StOral1	TGCACCGAGCGGATGAGTT	Bacteria	Firmicutes	Bacilli	Lactobacillales	Streptococcaceae	Streptococcus	Streptococcus sp. oral clone GK051; AY349413
			Bacteria	Firmicutes	Bacilli	Lactobacillales	Streptococcaceae	Streptococcus	Streptococcus sp. oral clone FP064; AF432139
			Bacteria	Firmicutes	Bacilli	Lactobacillales	Streptococcaceae	Streptococcus	Streptococcus sp. oral clone FN051; AF432135
			Bacteria	Firmicutes	Bacilli	Lactobacillales	Streptococcaceae	Streptococcus	Streptococcus sp. oral clone FP015; AF432137
			Bacteria	Firmicutes	Bacilli	Lactobacillales	Streptococcaceae	Streptococcus	Streptococcus sp. oral clone DP009; AF432132
			Bacteria	Firmicutes	Bacilli	Lactobacillales	Streptococcaceae	Streptococcus	Streptococcus sp. oral clone FO042; AF432136
			Bacteria	Firmicutes	Bacilli	Lactobacillales	Streptococcaceae	Streptococcus	Streptococcus sp. oral clone DN025; AF432131
			Bacteria	Firmicutes	Bacilli	Lactobacillales	Streptococcaceae	Streptococcus	Streptococcus gallinaceus (T); type strain: CCUG 42692; AJ307888
			Bacteria	Firmicutes	Bacilli	Lactobacillales	Streptococcaceae	Streptococcus	Streptococcus sp. oral clone MCE7_144; AF481230
			Bacteria	Firmicutes	Bacilli	Lactobacillales	Streptococcaceae	Streptococcus	Streptococcus genomosp. C6; C4MKM110; AY278634
			Bacteria	Firmicutes	Bacilli	Lactobacillales	Streptococcaceae	Streptococcus	Streptococcus genomosp. C7; C5AKM109; AY278635
			Bacteria	Firmicutes	Bacilli	Lactobacillales	Streptococcaceae	Streptococcus	Streptococcus genomosp. C1; C2MKM006; AY278629
			Bacteria	Firmicutes	Bacilli	Lactobacillales	Streptococcaceae	Streptococcus	Streptococcus sp. oral clone AA007; AY005046
			Bacteria	Firmicutes	Bacilli	Lactobacillales	Streptococcaceae	Streptococcus	Streptococcus oralis; ATCC 35037; AY485602
			Bacteria	Firmicutes	Bacilli	Lactobacillales	Streptococcaceae	Streptococcus	Streptococcus oralis; ATCC 35037; AF003932
			Bacteria	Firmicutes	Bacilli	Lactobacillales	Streptococcaceae	Streptococcus	Streptococcus mitis; ATCC 903; AY281078
			Bacteria	Firmicutes	Bacilli	Lactobacillales	Streptococcaceae	Streptococcus	Streptococcus parasanguinis; ATCC 15909; AY281087
			Bacteria	Firmicutes	Bacilli	Lactobacillales	Streptococcaceae	Streptococcus	uncultured Gram-positive bacterium; S15B-MN30; AJ583201
			Bacteria	Firmicutes	Bacilli	Lactobacillales	Streptococcaceae	Streptococcus	Streptococcus parasanguinis; GIFU8099; AB006125
			Bacteria	Firmicutes	Bacilli	Lactobacillales	Streptococcaceae	Streptococcus	Streptococcus parasanguinis; 85-81; X53652
o1567	o1567 16S-StrAga	CTGATGAGTTGCGAACGGGT	Bacteria	Firmicutes	Bacilli	Lactobacillales	Streptococcaceae	Streptococcus	Streptococcus agalactiae; AF459432
			Bacteria	Firmicutes	Bacilli	Lactobacillales	Streptococcaceae	Streptococcus	uncultured bacterium; rRNA232; AY959005
			Bacteria	Firmicutes	Bacilli	Lactobacillales	Streptococcaceae	Streptococcus	uncultured bacterium; rRNA203; AY958976
			Bacteria	Firmicutes	Bacilli	Lactobacillales	Streptococcaceae	Streptococcus	Streptococcus agalactiae 2603V/R; AE014191
			Bacteria	Firmicutes	Bacilli	Lactobacillales	Streptococcaceae	Streptococcus	Streptococcus agalactiae 2603V/R; AE014192
			Bacteria	Firmicutes	Bacilli	Lactobacillales	Streptococcaceae	Streptococcus	Streptococcus agalactiae 2603V/R; AE014195
			Bacteria	Firmicutes	Bacilli	Lactobacillales	Streptococcaceae	Streptococcus	Streptococcus agalactiae 2603V/R; AE014198
			Bacteria	Firmicutes	Bacilli	Lactobacillales	Streptococcaceae	Streptococcus	Streptococcus agalactiae 2603V/R; AE014202
			Bacteria	Firmicutes	Bacilli	Lactobacillales	Streptococcaceae	Streptococcus	Streptococcus agalactiae 2603V/R; AE014207
			Bacteria	Firmicutes	Bacilli	Lactobacillales	Streptococcaceae	Streptococcus	Streptococcus agalactiae 2603V/R; AE014210
			Bacteria	Firmicutes	Bacilli	Lactobacillales	Streptococcaceae	Streptococcus	Streptococcus agalactiae; JCM 5671; AB023574
			Bacteria	Firmicutes	Bacilli	Lactobacillales	Streptococcaceae	Streptococcus	uncultured bacterium; rRNA277; AY959050
			Bacteria	Firmicutes	Bacilli	Lactobacillales	Streptococcaceae	Streptococcus	Streptococcus agalactiae; ATCC 27956; AF015927
			Bacteria	Firmicutes	Bacilli	Lactobacillales	Streptococcaceae	Streptococcus	Streptococcus agalactiae (T); ATCC 13813, NCTC 8181; AB002479
			Bacteria	Firmicutes	Bacilli	Lactobacillales	Streptococcaceae	Streptococcus	Streptococcus agalactiae; GTC 730 (T) =ATCC 51487; AB112407
			Bacteria	Firmicutes	Bacilli	Lactobacillales	Streptococcaceae	Streptococcus	Streptococcus agalactiae; GTC 2049 = CIP 103853; AB175036
			Bacteria	Firmicutes	Bacilli	Lactobacillales	Streptococcaceae	Streptococcus	Streptococcus agalactiae; GTC 1234(T) = NCTC 8181(T); AB175037
			Bacteria	Firmicutes	Bacilli	Lactobacillales	Streptococcaceae	Streptococcus	Streptococcus equi subsp. ruminatorum (T); type strain: CECT 5772; AJ605748
			Bacteria	Firmicutes	Bacilli	Lactobacillales	Streptococcaceae	Streptococcus	Streptococcus agalactiae NEM316; AL766843
			Bacteria	Firmicutes	Bacilli	Lactobacillales	Streptococcaceae	Streptococcus	Streptococcus agalactiae NEM316; AL766843
o1568	o1568 16S-StrAng	CTGAAGTGAAAGGCAGTGGC	Bacteria	Firmicutes	Bacilli	Lactobacillales	Streptococcaceae	Streptococcus	Streptococcus anginosus; 1093; AF145244
			Bacteria	Firmicutes	Bacilli	Lactobacillales	Streptococcaceae	Streptococcus	Streptococcus anginosus; ChDC YA11; AY691541
			Bacteria	Firmicutes	Bacilli	Lactobacillales	Streptococcaceae	Streptococcus	Streptococcus anginosus; ChDC YA4; AY691536
			Bacteria	Firmicutes	Bacilli	Lactobacillales	Streptococcaceae	Streptococcus	Streptococcus anginosus; 367; AF145239
			Bacteria	Firmicutes	Bacilli	Lactobacillales	Streptococcaceae	Streptococcus	Streptococcus anginosus; ChDC YA3; AY691535
			Bacteria	Firmicutes	Bacilli	Lactobacillales	Streptococcaceae	Streptococcus	Streptococcus anginosus; ChDC YA1; AY691534
			Bacteria	Firmicutes	Bacilli	Lactobacillales	Streptococcaceae	Streptococcus	Streptococcus constellatus subsp. constellatus; 1; AY277937
			Bacteria	Firmicutes	Bacilli	Lactobacillales	Streptococcaceae	Streptococcus	Streptococcus constellatus subsp. constellatus; 857; AY277938
			Bacteria	Firmicutes	Bacilli	Lactobacillales	Streptococcaceae	Streptococcus	Streptococcus constellatus subsp. constellatus; 15; AY277939
			Bacteria	Firmicutes	Bacilli	Lactobacillales	Streptococcaceae	Streptococcus	Streptococcus anginosus; 414; AF145243
			Bacteria	Firmicutes	Bacilli	Lactobacillales	Streptococcaceae	Streptococcus	Streptococcus anginosus; 1204; AF145240
			Bacteria	Firmicutes	Bacilli	Lactobacillales	Streptococcaceae	Streptococcus	Streptococcus anginosus; 920; AF145246
			Bacteria	Firmicutes	Bacilli	Lactobacillales	Streptococcaceae	Streptococcus	Streptococcus anginosus; 1007; AF145245
			Bacteria	Firmicutes	Bacilli	Lactobacillales	Streptococcaceae	Streptococcus	Streptococcus milleri; SJMC; X81023
			Bacteria	Firmicutes	Bacilli	Lactobacillales	Streptococcaceae	Streptococcus	Streptococcus anginosus; ChDC YA6; AY691537
			Bacteria	Firmicutes	Bacilli	Lactobacillales	Streptococcaceae	Streptococcus	Streptococcus sp.; 69130; isolated from urine sample, Massachusetts General Hospital
			Bacteria	Firmicutes	Bacilli	Lactobacillales	Streptococcaceae	Streptococcus	Streptococcus sp.; MGH; isolated at Masachusetts General Hospital, USA, source unkr
			Bacteria	Firmicutes	Bacilli	Lactobacillales	Streptococcaceae	Streptococcus	Streptococcus anginosus; GTC820; AB006119
			Bacteria	Firmicutes	Bacilli	Lactobacillales	Streptococcaceae	Streptococcus	Streptococcus anginosus (T); NCTC 10713 (T); X58309
			Bacteria	Firmicutes	Bacilli	Lactobacillales	Streptococcaceae	Streptococcus	Streptococcus constellatus subsp. constellatus (T); X58310
o1569	o1569 16S-StrBov2	GAAGAGTTGCGAACGGGTG	Bacteria	Firmicutes	Bacilli	Lactobacillales	Streptococcaceae	Streptococcus	Streptococcus bovis; H2-1; AF202263
			Bacteria	Firmicutes	Bacilli	Lactobacillales	Streptococcaceae	Streptococcus	Streptococcus gallolyticus subsp. macedonicus; ACA-DC 206, LAB617; Z94012
			Bacteria	Firmicutes	Bacilli	Lactobacillales	Streptococcaceae	Streptococcus	Streptococcus bovis; RD11; AY327523
			Bacteria	Firmicutes	Bacilli	Lactobacillales	Streptococcaceae	Streptococcus	Streptococcus bovis; MPR1; AY324610
			Bacteria	Firmicutes	Bacilli	Lactobacillales	Streptococcaceae	Streptococcus	Streptococcus sp. oral clone BE024; AF385550
			Bacteria	Firmicutes	Bacilli	Lactobacillales	Streptococcaceae	Streptococcus	Streptococcus bovis; MPR2; AY324611
			Bacteria	Firmicutes	Bacilli	Lactobacillales	Streptococcaceae	Streptococcus	Streptococcus bovis; MPR4; AY324612
			Bacteria	Firmicutes	Bacilli	Lactobacillales	Streptococcaceae	Streptococcus	Streptococcus bovis; MPR5; AY324613
			Bacteria	Firmicutes	Bacilli	Lactobacillales	Streptococcaceae	Streptococcus	Streptococcus sp. oral clone AY020; AF385545
			Bacteria	Firmicutes	Bacilli	Lactobacillales	Streptococcaceae	Streptococcus	Streptococcus bovis; ATCC 27960; AB002481
			Bacteria	Firmicutes	Bacilli	Lactobacillales	Streptococcaceae	Streptococcus	Streptococcus bovis; YE49; AY442813
			Bacteria	Firmicutes	Bacilli	Lactobacillales	Streptococcaceae	Streptococcus	Streptococcus infantarius (T); HDP90104; SLB; AF177729
			Bacteria	Firmicutes	Bacilli	Lactobacillales	Streptococcaceae	Streptococcus	Streptococcus bovis; RD09; AY327522
			Bacteria	Firmicutes	Bacilli	Lactobacillales	Streptococcaceae	Streptococcus	Streptococcus luteciae; NEM 782; AJ297215
			Bacteria	Firmicutes	Bacilli	Lactobacillales	Streptococcaceae	Streptococcus	Streptococcus bovis (T); ATCC 33317, NCDO 597; AB002482
			Bacteria	Firmicutes	Bacilli	Lactobacillales	Streptococcaceae	Streptococcus	Streptococcus bovis; 1276; AF135453

			Bacteria	Firmicutes	Bacilli	Lactobacillales	Streptococcaceae	Streptococcus	uncultured streptococcus R16-B35; AF157108
			Bacteria	Firmicutes	Bacilli	Lactobacillales	Streptococcaceae	Streptococcus	Streptococcus luteciae; NEM 760; AJ297214
			Bacteria	Firmicutes	Bacilli	Lactobacillales	Streptococcaceae	Streptococcus	Streptococcus luteciae; NEM1603; AJ297218
			Bacteria	Firmicutes	Bacilli	Lactobacillales	Streptococcaceae	Streptococcus	uncultured Streptococcus sp.; AUSTP265; AY648569
o1570	o1570 16S-StrepSpe2	GATGGACCTGCGTTGTATTAG	Bacteria	Firmicutes	Bacilli	Lactobacillales	Streptococcaceae	Streptococcus	Streptococcus mitis; 127R; AJ295848
			Bacteria	Firmicutes	Bacilli	Lactobacillales	Streptococcaceae	Streptococcus	Streptococcus mitis; 209; AJ295853
			Bacteria	Firmicutes	Bacilli	Lactobacillales	Streptococcaceae	Streptococcus	Streptococcus mutans; AF199; AF139602
			Bacteria	Firmicutes	Bacilli	Lactobacillales	Streptococcaceae	Streptococcus	Streptococcus mutans; UA140; AF139599
			Bacteria	Firmicutes	Bacilli	Lactobacillales	Streptococcaceae	Streptococcus	unidentified bacterium; T44; Z94011
			Bacteria	Firmicutes	Bacilli	Lactobacillales	Streptococcaceae	Streptococcus	Streptococcus thermophilus (T); DSM 20617; X68418
			Bacteria	Firmicutes	Bacilli	Lactobacillales	Streptococcaceae	Streptococcus	Streptococcus uberis; HN1; AB023576
			Bacteria	Firmicutes	Bacilli	Lactobacillales	Streptococcaceae	Streptococcus	Streptococcus sp.; 2285-97; AJ131965
			Bacteria	Firmicutes	Bacilli	Lactobacillales	Streptococcaceae	Streptococcus	Streptococcus suis; 194; AB071340
			Bacteria	Firmicutes	Bacilli	Lactobacillales	Streptococcaceae	Streptococcus	Streptococcus suis; DAT2; AB071337
			Bacteria	Firmicutes	Bacilli	Lactobacillales	Streptococcaceae	Streptococcus	Streptococcus suis; 220; AB071345
			Bacteria	Firmicutes	Bacilli	Lactobacillales	Streptococcaceae	Streptococcus	Streptococcus suis; 243; AB071350
			Bacteria	Firmicutes	Bacilli	Lactobacillales	Streptococcaceae	Streptococcus	Streptococcus suis; 226; AB071347
			Bacteria	Firmicutes	Bacilli	Lactobacillales	Streptococcaceae	Streptococcus	Streptococcus canis; CG40; AJ413205
			Bacteria	Firmicutes	Bacilli	Lactobacillales	Streptococcaceae	Streptococcus	Streptococcus dysgalactiae subsp. dysgalactiae; HKU7; AF433167
			Bacteria	Firmicutes	Bacilli	Lactobacillales	Streptococcaceae	Streptococcus	Streptococcus pasteuri; NEM1202, CIP 105070; AJ297216
			Bacteria	Firmicutes	Bacilli	Lactobacillales	Streptococcaceae	Streptococcus	Streptococcus sobrinus; NCTC 12279; AJ243966
			Bacteria	Firmicutes	Bacilli	Lactobacillales	Streptococcaceae	Streptococcus	Streptococcus pluranimalium; LMG 14257; Y18026
			Bacteria	Firmicutes	Bacilli	Lactobacillales	Streptococcaceae	Streptococcus	Streptococcus gallolyticus; ACM3968; Y10869
			Bacteria	Firmicutes	Bacilli	Lactobacillales	Streptococcaceae	Streptococcus	Streptococcus dysgalactiae subsp. dysgalactiae; NCFB1356; AB008926
o1571	o1571 16S-StrGor	TAATTGAAAGGTGCAATTGC	Bacteria	Firmicutes	Bacilli	Lactobacillales	Streptococcaceae	Streptococcus	uncultured Streptococcus sp.; 4.10; DQ016718
			Bacteria	Firmicutes	Bacilli	Lactobacillales	Streptococcaceae	Streptococcus	uncultured bacterium; OPEN_PLAQUE_5; FJ983031
			Bacteria	Firmicutes	Bacilli	Lactobacillales	Streptococcaceae	Streptococcus	uncultured Streptococcus sp.; 4.18; DQ346422
			Bacteria	Firmicutes	Bacilli	Lactobacillales	Streptococcaceae	Streptococcus	Streptococcus gordonii (T); ATCC 10558; AF003931
			Bacteria	Firmicutes	Bacilli	Lactobacillales	Streptococcaceae	Streptococcus	uncultured bacterium; BF0002A072; AM696887
			Bacteria	Firmicutes	Bacilli	Lactobacillales	Streptococcaceae	Streptococcus	Streptococcus gordonii; ATCC 10558; AY485606
			Bacteria	Firmicutes	Bacilli	Lactobacillales	Streptococcaceae	Streptococcus	Streptococcus sanguinis; TeTG; FJ823140
			Bacteria	Firmicutes	Bacilli	Lactobacillales	Streptococcaceae	Streptococcus	Streptococcus gordonii; ATCC 33399; AY281089
			Bacteria	Firmicutes	Bacilli	Lactobacillales	Streptococcaceae	Streptococcus	uncultured bacterium; P4D1-681; EF509681
			Bacteria	Firmicutes	Bacilli	Lactobacillales	Streptococcaceae	Streptococcus	Streptococcus gordonii; NCTC7865; D38483
			Bacteria	Firmicutes	Bacilli	Lactobacillales	Streptococcaceae	Streptococcus	Streptococcus gordonii; NJ122; AB355603
			Bacteria	Firmicutes	Bacilli	Lactobacillales	Streptococcaceae	Streptococcus	Streptococcus gordonii; NY126; AB355604
			Bacteria	Firmicutes	Bacilli	Lactobacillales	Streptococcaceae	Streptococcus	uncultured bacterium; nbu107d11c1; GQ000747
			Bacteria	Firmicutes	Bacilli	Lactobacillales	Streptococcaceae	Streptococcus	uncultured bacterium; nbw495g02c1; GQ101488
			Bacteria	Firmicutes	Bacilli	Lactobacillales	Streptococcaceae	Streptococcus	Streptococcus mitis; NCTC 3165, MAFF 911479; AB002520
			Bacteria	Firmicutes	Bacilli	Lactobacillales	Streptococcaceae	Streptococcus	uncultured bacterium; nbu105f09c1; GQ000600
			Bacteria	Firmicutes	Bacilli	Lactobacillales	Streptococcaceae	Streptococcus	uncultured bacterium; nbw1163a04c1; GQ078039
			Bacteria	Firmicutes	Bacilli	Lactobacillales	Streptococcaceae	Streptococcus	uncultured bacterium; nbu279b06c1; GQ021506
			Bacteria	Firmicutes	Bacilli	Lactobacillales	Streptococcaceae	Streptococcus	uncultured bacterium; nbu279c07c1; GQ021518
			Bacteria	Firmicutes	Bacilli	Lactobacillales	Streptococcaceae	Streptococcus	Streptococcus gordonii; ATCC 10558; EU156758
o1572	o1572 16S-StrGroup1	GGGAGAGTGGAATTCCATGT	Bacteria	Firmicutes	Bacilli	Lactobacillales	Streptococcaceae	Streptococcus	Streptococcus mutans; UA140; AF139599
			Bacteria	Firmicutes	Bacilli	Lactobacillales	Streptococcaceae	Streptococcus	unidentified bacterium; T44; Z94011
			Bacteria	Firmicutes	Bacilli	Lactobacillales	Streptococcaceae	Streptococcus	Streptococcus thermophilus (T); DSM 20617; X68418
			Bacteria	Firmicutes	Bacilli	Lactobacillales	Streptococcaceae	Streptococcus	Streptococcus suis; DAT2; AB071337
			Bacteria	Firmicutes	Bacilli	Lactobacillales	Streptococcaceae	Streptococcus	Streptococcus suis; 220; AB071345
			Bacteria	Firmicutes	Bacilli	Lactobacillales	Streptococcaceae	Streptococcus	Streptococcus gallinaceus (T); type strain: CCUG 42692; AJ307888
			Bacteria	Firmicutes	Bacilli	Lactobacillales	Streptococcaceae	Streptococcus	Streptococcus pasteuri; NEM1202, CIP 105070; AJ297216
			Bacteria	Firmicutes	Bacilli	Lactobacillales	Streptococcaceae	Streptococcus	Streptococcus sobrinus; NCTC 12279; AJ243966
			Bacteria	Firmicutes	Bacilli	Lactobacillales	Streptococcaceae	Streptococcus	Streptococcus thoraltensis (T); S69; Y09007
			Bacteria	Firmicutes	Bacilli	Lactobacillales	Streptococcaceae	Streptococcus	Streptococcus ferus (T); 8S1; AJ420197
			Bacteria	Firmicutes	Bacilli	Lactobacillales	Streptococcaceae	Streptococcus	Streptococcus ratti (T); ATCC19645; AJ420201
			Bacteria	Firmicutes	Bacilli	Lactobacillales	Streptococcaceae	Streptococcus	Streptococcus gallolyticus (T); ACM 3611; X94337
			Bacteria	Firmicutes	Bacilli	Lactobacillales	Streptococcaceae	Streptococcus	Streptococcus ovis (T); S369-98-1; Y17358
			Bacteria	Firmicutes	Bacilli	Lactobacillales	Streptococcaceae	Streptococcus	Streptococcus entericus (T); CECT 5353; AJ409287
			Bacteria	Firmicutes	Bacilli	Lactobacillales	Enterococcaceae	Vagococcus	Vagococcus salmoninarum (T); NCFB 27777 T; X54272
			Bacteria	Firmicutes	Bacilli	Lactobacillales	Enterococcaceae	Vagococcus	Vagococcus fluvialis; NCDO 2497; X54258
			Bacteria	Firmicutes	Bacilli	Lactobacillales	Streptococcaceae	Streptococcus	Streptococcus mutans (T); NCTC 10449(T); X58303
			Bacteria	Firmicutes	Bacilli	Lactobacillales	Streptococcaceae	Streptococcus	Streptococcus equinus (T); NCDO 1037(T); X58318
			Bacteria	Firmicutes	Bacilli	Lactobacillales	Streptococcaceae	Streptococcus	Streptococcus criceti (T); NCDO 2720 (T); X58305
			Bacteria	Firmicutes	Bacilli	Lactobacillales	Streptococcaceae	Streptococcus	Streptococcus vestibularis (T); NCTC 12166(T); X58321
o1573	o1573 16S-StrGroup2	CTGTGACGGTATCTTACCAG	Bacteria	Firmicutes	Bacilli	Lactobacillales	Streptococcaceae	Streptococcus	Streptococcus mitis; 127R; AJ295848
			Bacteria	Firmicutes	Bacilli	Lactobacillales	Streptococcaceae	Streptococcus	Streptococcus mitis; 209; AJ295853
			Bacteria	Firmicutes	Bacilli	Lactobacillales	Streptococcaceae	Streptococcus	Streptococcus sp. YE54; AY442818
			Bacteria	Firmicutes	Bacilli	Lactobacillales	Streptococcaceae	Streptococcus	Streptococcus sp. oral clone EK048; AF385574
			Bacteria	Firmicutes	Bacilli	Lactobacillales	Streptococcaceae	Streptococcus	Streptococcus sp. oral clone DP009; AF432132
			Bacteria	Firmicutes	Bacilli	Lactobacillales	Streptococcaceae	Streptococcus	Streptococcus sp. oral clone BE024; AF385550
			Bacteria	Firmicutes	Bacilli	Lactobacillales	Streptococcaceae	Streptococcus	Streptococcus genomosp. C3; C3ALM006; AY278631
			Bacteria	Firmicutes	Bacilli	Lactobacillales	Streptococcaceae	Streptococcus	Streptococcus sp. oral clone AY020; AF385545
			Bacteria	Firmicutes	Bacilli	Lactobacillales	Streptococcaceae	Streptococcus	Streptococcus genomosp. C8; C5MLM037; AY278609
			Bacteria	Firmicutes	Bacilli	Lactobacillales	Streptococcaceae	Streptococcus	Streptococcus genomosp. C4; C3MLM097; AY278632
			Bacteria	Firmicutes	Bacilli	Lactobacillales	Streptococcaceae	Streptococcus	Streptococcus sp. oral clone DN025; AF432131
			Bacteria	Firmicutes	Bacilli	Lactobacillales	Streptococcaceae	Streptococcus	Streptococcus genomosp. C7; C5AKM109; AY278635
			Bacteria	Firmicutes	Bacilli	Lactobacillales	Streptococcaceae	Streptococcus	Streptococcus genomosp. C2; C2MKM128; AY278630
			Bacteria	Firmicutes	Bacilli	Lactobacillales	Streptococcaceae	Streptococcus	Streptococcus genomosp. C5; C4AKM023; AY278633
			Bacteria	Firmicutes	Bacilli	Lactobacillales	Streptococcaceae	Streptococcus	Streptococcus gordonii; DL1; AJ427479
			Bacteria	Firmicutes	Bacilli	Lactobacillales	Streptococcaceae	Streptococcus	uncultured Gram-positive bacterium; S15B-MN51; AJ583202
			Bacteria	Firmicutes	Bacilli	Lactobacillales	Streptococcaceae	Streptococcus	uncultured Gram-positive bacterium; S15B-MN30; AJ583201
			Bacteria	Firmicutes	Bacilli	Lactobacillales	Streptococcaceae	Streptococcus	Streptococcus sp. PSH2; AB038371
			Bacteria	Firmicutes	Bacilli	Lactobacillales	Streptococcaceae	Streptococcus	Streptococcus sp. RR1; AB174791
			Bacteria	Firmicutes	Bacilli	Lactobacillales	Streptococcaceae	Streptococcus	Streptococcus sp. RR3; AB174792
o1574	o1574 16S-StrGroup3	CGGTAACTAACCAGAAAGGG	Bacteria	Firmicutes	Bacilli	Lactobacillales	Streptococcaceae	Streptococcus	Streptococcus uberis; JCM 5709; AB023573
			Bacteria	Firmicutes	Bacilli	Lactobacillales	Streptococcaceae	Streptococcus	Streptococcus uberis; HN1; AB023576
			Bacteria	Firmicutes	Bacilli	Lactobacillales	Streptococcaceae	Streptococcus	Streptococcus sp.; 2285-97; AJ131965
			Bacteria	Firmicutes	Bacilli	Lactobacillales	Streptococcaceae	Streptococcus	Streptococcus pyogenes; JCM 5674; AB023575

			Bacteria	Firmicutes	Bacilli	Lactobacillales	Streptococcaceae	Streptococcus	Streptococcus agalactiae; JCM 5671; AB023574
			Bacteria	Firmicutes	Bacilli	Lactobacillales	Streptococcaceae	Streptococcus	Streptococcus canis; CG40; AJ413205
			Bacteria	Firmicutes	Bacilli	Lactobacillales	Streptococcaceae	Streptococcus	Streptococcus canis; NCTC12191; AJ413203
			Bacteria	Firmicutes	Bacilli	Lactobacillales	Streptococcaceae	Streptococcus	Streptococcus dysgalactiae subsp. dysgalactiae; HKU7; AF433167
			Bacteria	Firmicutes	Bacilli	Lactobacillales	Streptococcaceae	Streptococcus	Streptococcus canis; CG5; AJ413204
			Bacteria	Firmicutes	Bacilli	Lactobacillales	Streptococcaceae	Streptococcus	Streptococcus dysgalactiae subsp. equisimilis; ATCC 35666; AJ319643
			Bacteria	Firmicutes	Bacilli	Lactobacillales	Streptococcaceae	Streptococcus	Streptococcus dysgalactiae subsp. equisimilis; AC-2713; AJ314609
			Bacteria	Firmicutes	Bacilli	Lactobacillales	Streptococcaceae	Streptococcus	Streptococcus dysgalactiae subsp. equisimilis; AC-4436; AJ319644
			Bacteria	Firmicutes	Bacilli	Lactobacillales	Streptococcaceae	Streptococcus	Streptococcus dysgalactiae subsp. equisimilis; AC-2074; AJ314611
			Bacteria	Firmicutes	Bacilli	Lactobacillales	Streptococcaceae	Streptococcus	Streptococcus dysgalactiae subsp. equisimilis; AC-2832; AJ314610
			Bacteria	Firmicutes	Bacilli	Lactobacillales	Streptococcaceae	Streptococcus	Streptococcus parauberis; X89967
			Bacteria	Firmicutes	Bacilli	Lactobacillales	Streptococcaceae	Streptococcus	Streptococcus dysgalactiae subsp. dysgalactiae; NCFB1356; AB008926
			Bacteria	Firmicutes	Bacilli	Lactobacillales	Streptococcaceae	Streptococcus	Streptococcus agalactiae; X59032
			Bacteria	Firmicutes	Bacilli	Lactobacillales	Streptococcaceae	Streptococcus	Streptococcus canis; X59061
			Bacteria	Firmicutes	Bacilli	Lactobacillales	Streptococcaceae	Streptococcus	Streptococcus dysgalactiae subsp. dysgalactiae; X59030
			Bacteria	Firmicutes	Bacilli	Lactobacillales	Streptococcaceae	Streptococcus	Streptococcus equi subsp. equi; NCDO 2493(T); X58314
o1575	o1575 16S-StrGroup4	GTGGCTTAACCATTGTTCGC	Bacteria	Firmicutes	Bacilli	Lactobacillales	Streptococcaceae	Streptococcus	Streptococcus gallolyticus subsp. macedonicus; ACA-DC 206, LAB617; Z94012
			Bacteria	Firmicutes	Bacilli	Lactobacillales	Streptococcaceae	Streptococcus	Streptococcus equinus; NCTC11436; AJ305257
			Bacteria	Firmicutes	Bacilli	Lactobacillales	Streptococcaceae	Streptococcus	Streptococcus bovis; MPR1; AY324610
			Bacteria	Firmicutes	Bacilli	Lactobacillales	Streptococcaceae	Streptococcus	Streptococcus bovis; MPR2; AY324611
			Bacteria	Firmicutes	Bacilli	Lactobacillales	Streptococcaceae	Streptococcus	Streptococcus bovis; MPR4; AY324612
			Bacteria	Firmicutes	Bacilli	Lactobacillales	Streptococcaceae	Streptococcus	Streptococcus bovis; MPR5; AY324613
			Bacteria	Firmicutes	Bacilli	Lactobacillales	Streptococcaceae	Streptococcus	Streptococcus bovis; YE49; AY442813
			Bacteria	Firmicutes	Bacilli	Lactobacillales	Streptococcaceae	Streptococcus	Streptococcus pasteuri; NEM1205, CIP 105073; AJ297217
			Bacteria	Firmicutes	Bacilli	Lactobacillales	Streptococcaceae	Streptococcus	uncultured bacterium; p-4-a5; AF371504
			Bacteria	Firmicutes	Bacilli	Lactobacillales	Streptococcaceae	Streptococcus	Streptococcus pasteuri; NEM1202, CIP 105070; AJ297216
			Bacteria	Firmicutes	Bacilli	Lactobacillales	Streptococcaceae	Streptococcus	uncultured bacterium; p-29-a5; AF371506
			Bacteria	Firmicutes	Bacilli	Lactobacillales	Streptococcaceae	Streptococcus	Streptococcus luteciae; NEM 782; AJ297215
			Bacteria	Firmicutes	Bacilli	Lactobacillales	Streptococcaceae	Streptococcus	uncultured streptococcus R16-B35; AF157108
			Bacteria	Firmicutes	Bacilli	Lactobacillales	Streptococcaceae	Streptococcus	uncultured bacterium; p-4289-4Wa3; AF371507
			Bacteria	Firmicutes	Bacilli	Lactobacillales	Streptococcaceae	Streptococcus	Streptococcus luteciae; NEM 760; AJ297214
			Bacteria	Firmicutes	Bacilli	Lactobacillales	Streptococcaceae	Streptococcus	Streptococcus equinus; ATCC 9812; AJ301607
			Bacteria	Firmicutes	Bacilli	Lactobacillales	Streptococcaceae	Streptococcus	Streptococcus gallolyticus (T); ACM 3611; X94337
			Bacteria	Firmicutes	Bacilli	Lactobacillales	Streptococcaceae	Streptococcus	Streptococcus alactolyticus; ONK4; AY324631
			Bacteria	Firmicutes	Bacilli	Lactobacillales	Streptococcaceae	Streptococcus	Streptococcus gallolyticus; ACM3968; Y10869
			Bacteria	Firmicutes	Bacilli	Lactobacillales	Streptococcaceae	Streptococcus	uncultured Streptococcus sp.; AUSTP265; AY648569
o1576	o1576 16S-StrGroup6	CTGAAGTAAAAGGCTGTGGC	Bacteria	Firmicutes	Bacilli	Lactobacillales	Streptococcaceae	Streptococcus	unidentified bacterium; T44; Z94011
			Bacteria	Firmicutes	Bacilli	Lactobacillales	Streptococcaceae	Streptococcus	uncultured sheep mite bacterium Welshpool 44; AF290487
			Bacteria	Firmicutes	Bacilli	Lactobacillales	Streptococcaceae	Streptococcus	Streptococcus suis; 194; AB071340
			Bacteria	Firmicutes	Bacilli	Lactobacillales	Streptococcaceae	Streptococcus	Streptococcus suis; DAT2; AB071337
			Bacteria	Firmicutes	Bacilli	Lactobacillales	Streptococcaceae	Streptococcus	Streptococcus suis; 220; AB071345
			Bacteria	Firmicutes	Bacilli	Lactobacillales	Streptococcaceae	Streptococcus	Streptococcus suis; 243; AB071350
			Bacteria	Firmicutes	Bacilli	Lactobacillales	Streptococcaceae	Streptococcus	Streptococcus suis; 226; AB071347
			Bacteria	Firmicutes	Bacilli	Lactobacillales	Streptococcaceae	Streptococcus	Streptococcus suis; 210; AB071343
			Bacteria	Firmicutes	Bacilli	Lactobacillales	Streptococcaceae	Streptococcus	Streptococcus suis; NIAH11318; AB071338
			Bacteria	Firmicutes	Bacilli	Lactobacillales	Streptococcaceae	Streptococcus	Streptococcus suis; 211; AB071344
			Bacteria	Firmicutes	Bacilli	Lactobacillales	Streptococcaceae	Streptococcus	Streptococcus suis; 203; AB071341
			Bacteria	Firmicutes	Bacilli	Lactobacillales	Streptococcaceae	Streptococcus	Streptococcus suis; 228; AB071348
			Bacteria	Firmicutes	Bacilli	Lactobacillales	Streptococcaceae	Streptococcus	Streptococcus suis; 235; AB071349
			Bacteria	Firmicutes	Bacilli	Lactobacillales	Streptococcaceae	Streptococcus	Streptococcus suis; DAT1; AB071336
			Bacteria	Firmicutes	Bacilli	Lactobacillales	Streptococcaceae	Streptococcus	Streptococcus suis; 222; AB071346
			Bacteria	Firmicutes	Bacilli	Lactobacillales	Streptococcaceae	Streptococcus	Streptococcus suis; 209; AB071342
			Bacteria	Firmicutes	Bacilli	Lactobacillales	Streptococcaceae	Streptococcus	Streptococcus suis; 193; AB071339
			Bacteria	Firmicutes	Bacilli	Lactobacillales	Streptococcaceae	Streptococcus	uncultured bacterium; p-4747-2Wb2; AF371511
			Bacteria	Firmicutes	Bacilli	Lactobacillales	Streptococcaceae	Streptococcus	Streptococcus suis (T); ATCC 43765, NCTC 10234; AB002525
			Bacteria	Firmicutes	Bacilli	Lactobacillales	Streptococcaceae	Streptococcus	Streptococcus suis; X59031
o1577	o1577 16S-StrGroupAn	CAGGCGGTTAGAAAAGTCTG	Bacteria	Firmicutes	Bacilli	Lactobacillales	Streptococcaceae	Streptococcus	Streptococcus anginosus; ATCC33397; AF104678
			Bacteria	Firmicutes	Bacilli	Lactobacillales	Streptococcaceae	Streptococcus	Streptococcus anginosus; 1012; AF104679
			Bacteria	Firmicutes	Bacilli	Lactobacillales	Streptococcaceae	Streptococcus	Streptococcus anginosus; 1093; AF145244
			Bacteria	Firmicutes	Bacilli	Lactobacillales	Streptococcaceae	Streptococcus	Streptococcus anginosus; ChDC YA11; AY691541
			Bacteria	Firmicutes	Bacilli	Lactobacillales	Streptococcaceae	Streptococcus	Streptococcus anginosus; ChDC YA4; AY691536
			Bacteria	Firmicutes	Bacilli	Lactobacillales	Streptococcaceae	Streptococcus	Streptococcus anginosus; 367; AF145239
			Bacteria	Firmicutes	Bacilli	Lactobacillales	Streptococcaceae	Streptococcus	Streptococcus anginosus; ChDC YA3; AY691535
			Bacteria	Firmicutes	Bacilli	Lactobacillales	Streptococcaceae	Streptococcus	Streptococcus anginosus; ChDC YA1; AY691534
			Bacteria	Firmicutes	Bacilli	Lactobacillales	Streptococcaceae	Streptococcus	Streptococcus anginosus; 414; AF145243
			Bacteria	Firmicutes	Bacilli	Lactobacillales	Streptococcaceae	Streptococcus	Streptococcus anginosus; 1204; AF145240
			Bacteria	Firmicutes	Bacilli	Lactobacillales	Streptococcaceae	Streptococcus	Streptococcus anginosus; MAS624; AY309096
			Bacteria	Firmicutes	Bacilli	Lactobacillales	Streptococcaceae	Streptococcus	Streptococcus anginosus; 920; AF145246
			Bacteria	Firmicutes	Bacilli	Lactobacillales	Streptococcaceae	Streptococcus	Streptococcus anginosus; 1007; AF145245
			Bacteria	Firmicutes	Bacilli	Lactobacillales	Streptococcaceae	Streptococcus	Streptococcus milleri; SJMC; X81023
			Bacteria	Firmicutes	Bacilli	Lactobacillales	Streptococcaceae	Streptococcus	Streptococcus anginosus; ChDC YA6; AY691537
			Bacteria	Firmicutes	Bacilli	Lactobacillales	Streptococcaceae	Streptococcus	Streptococcus sp.; 69130; isolated from urine sample, Massachusetts General Hospital
			Bacteria	Firmicutes	Bacilli	Lactobacillales	Streptococcaceae	Streptococcus	Streptococcus sp.; MGH; isolated at Masachusetts General Hospital, USA, source unkn
			Bacteria	Firmicutes	Bacilli	Lactobacillales	Streptococcaceae	Streptococcus	Streptococcus anginosus; GTC820; AB006119
			Bacteria	Firmicutes	Bacilli	Lactobacillales	Streptococcaceae	Streptococcus	Streptococcus anginosus; GTC821; AB006120
			Bacteria	Firmicutes	Bacilli	Lactobacillales	Streptococcaceae	Streptococcus	Streptococcus anginosus (T); NCTC 10713 (T); X58309
o1578	o1578 16S-StrGroupAn	AGTGGCTCAACCATTGTAGG	Bacteria	Firmicutes	Bacilli	Lactobacillales	Streptococcaceae	Streptococcus	Streptococcus dysgalactiae subsp. dysgalactiae; AB102730
			Bacteria	Firmicutes	Bacilli	Lactobacillales	Streptococcaceae	Streptococcus	Streptococcus dysgalactiae subsp. equisimilis; ATCC 35666; AB096755
			Bacteria	Firmicutes	Bacilli	Lactobacillales	Streptococcaceae	Streptococcus	Streptococcus canis; CG40; AJ413205
			Bacteria	Firmicutes	Bacilli	Lactobacillales	Streptococcaceae	Streptococcus	Streptococcus dysgalactiae subsp. dysgalactiae; HKU7; AF433167
			Bacteria	Firmicutes	Bacilli	Lactobacillales	Streptococcaceae	Streptococcus	Streptococcus dysgalactiae subsp. equisimilis; ATCC 35666; AJ319643
			Bacteria	Firmicutes	Bacilli	Lactobacillales	Streptococcaceae	Streptococcus	Streptococcus dysgalactiae subsp. equisimilis; AC-2713; AJ314609
			Bacteria	Firmicutes	Bacilli	Lactobacillales	Streptococcaceae	Streptococcus	Streptococcus dysgalactiae subsp. equisimilis; AC-4436; AJ319644
			Bacteria	Firmicutes	Bacilli	Lactobacillales	Streptococcaceae	Streptococcus	Streptococcus dysgalactiae subsp. equisimilis; AC-2074; AJ314611
			Bacteria	Firmicutes	Bacilli	Lactobacillales	Streptococcaceae	Streptococcus	Streptococcus dysgalactiae subsp. equisimilis; AC-2832; AJ314610
			Bacteria	Firmicutes	Bacilli	Lactobacillales	Streptococcaceae	Streptococcus	Streptococcus thoraltensis (T); S69; Y09007
			Bacteria	Firmicutes	Bacilli	Lactobacillales	Streptococcaceae	Streptococcus	Streptococcus milleri; SJMC; X81023
			Bacteria	Firmicutes	Bacilli	Lactobacillales	Streptococcaceae	Streptococcus	Streptococcus dysgalactiae subsp. equisimilis; 1149; AB104840

			Bacteria	Firmicutes	Bacilli	Lactobacillales	Streptococcaceae	Streptococcus	Streptococcus dysgalactiae subsp. equisimilis; Hiroshima; AB104845
			Bacteria	Firmicutes	Bacilli	Lactobacillales	Streptococcaceae	Streptococcus	Streptococcus dysgalactiae subsp. equisimilis; N1434; AB104844
			Bacteria	Firmicutes	Bacilli	Lactobacillales	Streptococcaceae	Streptococcus	Streptococcus sp.; 69130; isolated from urine sample, Massachusetts General Hospital
			Bacteria	Firmicutes	Bacilli	Lactobacillales	Streptococcaceae	Streptococcus	Streptococcus sp.; MGH; isolated at Masachusetts General Hospital, USA, source unkr
			Bacteria	Firmicutes	Bacilli	Lactobacillales	Streptococcaceae	Streptococcus	Streptococcus entericus (T); CECT 5353; AJ409287
			Bacteria	Firmicutes	Bacilli	Lactobacillales	Streptococcaceae	Streptococcus	Streptococcus anginosus (T); NCTC 10713 (T); X58309
			Bacteria	Firmicutes	Bacilli	Lactobacillales	Streptococcaceae	Streptococcus	Streptococcus equi subsp. equi; NCDO 2493(T); X58314
			Bacteria	Firmicutes	Bacilli	Lactobacillales	Streptococcaceae	Streptococcus	Streptococcus intermedius; NCTC 11324; X58311
o1579	o1579 16S-StrMut	AAGGGAACACACTGTGCTTG	Bacteria	Firmicutes	Bacilli	Lactobacillales	Streptococcaceae	Streptococcus	Streptococcus mutans; DJ1; DQ462439
			Bacteria	Firmicutes	Bacilli	Lactobacillales	Streptococcaceae	Streptococcus	Streptococcus mutans; ATCC 25175; AY188348
			Bacteria	Firmicutes	Bacilli	Lactobacillales	Streptococcaceae	Streptococcus	Streptococcus mutans; ChDC YM101; 35135; DQ677739
			Bacteria	Firmicutes	Bacilli	Lactobacillales	Streptococcaceae	Streptococcus	Streptococcus mutans; ChDC YM211; PD1022; DQ677728
			Bacteria	Firmicutes	Bacilli	Lactobacillales	Streptococcaceae	Streptococcus	Streptococcus mutans; ChDC YM87; 55115; DQ677746
			Bacteria	Firmicutes	Bacilli	Lactobacillales	Streptococcaceae	Streptococcus	Streptococcus mutans; ChDC YM82; 52211; DQ677748
			Bacteria	Firmicutes	Bacilli	Lactobacillales	Streptococcaceae	Streptococcus	Streptococcus mutans; ChDC YM3; AY691526
			Bacteria	Firmicutes	Bacilli	Lactobacillales	Streptococcaceae	Streptococcus	Streptococcus mutans; ChDC YM83; AY691529
			Bacteria	Firmicutes	Bacilli	Lactobacillales	Streptococcaceae	Streptococcus	Streptococcus mutans; ChDC YM226; PD1115; DQ677715
			Bacteria	Firmicutes	Bacilli	Lactobacillales	Streptococcaceae	Streptococcus	Streptococcus mutans; ChDC YM219; PD1053; DQ677720
			Bacteria	Firmicutes	Bacilli	Lactobacillales	Streptococcaceae	Streptococcus	Streptococcus mutans; ChDC YM204; PD32; DQ677734
			Bacteria	Firmicutes	Bacilli	Lactobacillales	Streptococcaceae	Streptococcus	Streptococcus mutans; ChDC YM201; PD4; DQ677737
			Bacteria	Firmicutes	Bacilli	Lactobacillales	Streptococcaceae	Streptococcus	Streptococcus mutans; ChDC YM218; PD1049; DQ677721
			Bacteria	Firmicutes	Bacilli	Lactobacillales	Streptococcaceae	Streptococcus	Streptococcus mutans; ChDC YM213; PD1027; DQ677726
			Bacteria	Firmicutes	Bacilli	Lactobacillales	Streptococcaceae	Streptococcus	Streptococcus mutans; ChDC YM79; 49215; DQ677750
			Bacteria	Firmicutes	Bacilli	Lactobacillales	Streptococcaceae	Streptococcus	Streptococcus mutans; ChDC YM209; PD1018; DQ677730
			Bacteria	Firmicutes	Bacilli	Lactobacillales	Streptococcaceae	Streptococcus	Streptococcus mutans; ChDC YM90; 57211; DQ677745
			Bacteria	Firmicutes	Bacilli	Lactobacillales	Streptococcaceae	Streptococcus	Streptococcus mutans; NCTC 10449; AJ243965
			Bacteria	Firmicutes	Bacilli	Lactobacillales	Streptococcaceae	Streptococcus	Streptococcus mutans; ChDC YM207; PD69; DQ677731
			Bacteria	Firmicutes	Bacilli	Lactobacillales	Streptococcaceae	Streptococcus	Streptococcus mutans; ChDC YM210; PD1019; DQ677729
o1580	o1580 16S-StrOral1	TGAAGGAAGGAGCTTGCTCT	Bacteria	Firmicutes	Bacilli	Lactobacillales	Streptococcaceae	Streptococcus	Streptococcus sp. oral clone ASCC04; AY923127
			Bacteria	Firmicutes	Bacilli	Lactobacillales	Streptococcaceae	Streptococcus	Streptococcus sp. oral strain H3-M2; AF385523
			Bacteria	Firmicutes	Bacilli	Lactobacillales	Streptococcaceae	Streptococcus	uncultured Streptococcus sp.; 1.23; DQ016729
			Bacteria	Firmicutes	Bacilli	Lactobacillales	Streptococcaceae	Streptococcus	Streptococcus sp. oral clone FN042; AF432134
			Bacteria	Firmicutes	Bacilli	Lactobacillales	Streptococcaceae	Streptococcus	Streptococcus oligofermentans (T); 2-4T; AY099095
			Bacteria	Firmicutes	Bacilli	Lactobacillales	Streptococcaceae	Streptococcus	uncultured bacterium; P5D1-660; EF511070
			Bacteria	Firmicutes	Bacilli	Lactobacillales	Streptococcaceae	Streptococcus	uncultured bacterium; P5D1-450; EF511118
			Bacteria	Firmicutes	Bacilli	Lactobacillales	Streptococcaceae	Streptococcus	uncultured bacterium; P5D1-427; EF511129
			Bacteria	Firmicutes	Bacilli	Lactobacillales	Streptococcaceae	Streptococcus	uncultured bacterium; P5D1-645; EF511134
			Bacteria	Firmicutes	Bacilli	Lactobacillales	Streptococcaceae	Streptococcus	uncultured bacterium; P5D1-411; EF511102
			Bacteria	Firmicutes	Bacilli	Lactobacillales	Streptococcaceae	Streptococcus	uncultured bacterium; P5D1-651; EF511072
			Bacteria	Firmicutes	Bacilli	Lactobacillales	Streptococcaceae	Streptococcus	uncultured bacterium; P5D1-443; EF511121
			Bacteria	Firmicutes	Bacilli	Lactobacillales	Streptococcaceae	Streptococcus	uncultured bacterium; P5D1-391; EF511098
			Bacteria	Firmicutes	Bacilli	Lactobacillales	Streptococcaceae	Streptococcus	uncultured bacterium; P5D1-406; EF511124
			Bacteria	Firmicutes	Bacilli	Lactobacillales	Streptococcaceae	Streptococcus	uncultured bacterium; JSC7-29; DQ532197
			Bacteria	Firmicutes	Bacilli	Lactobacillales	Streptococcaceae	Streptococcus	Streptococcus australis; ATCC 700641; AY485604
			Bacteria	Firmicutes	Bacilli	Lactobacillales	Streptococcaceae	Streptococcus	Streptococcus sp. oral strain T1-E5; AF385525
			Bacteria	Firmicutes	Bacilli	Lactobacillales	Streptococcaceae	Streptococcus	uncultured bacterium; RL305aal86e07; DQ799458
			Bacteria	Firmicutes	Bacilli	Lactobacillales	Streptococcaceae	Streptococcus	uncultured Lactobacillales bacterium; 1099973754058; EF434343
			Bacteria	Firmicutes	Bacilli	Lactobacillales	Streptococcaceae	Streptococcus	Streptococcus australis (T); AI1; ATCC 700641; AF184974
o1581	o1581 16S-StrOral2	AAGGGAGAGCTTGCTCTTCT	Bacteria	Firmicutes	Bacilli	Lactobacillales	Streptococcaceae	Streptococcus	Streptococcus sp. oral clone ASCD01; AY923129
			Bacteria	Firmicutes	Bacilli	Lactobacillales	Streptococcaceae	Streptococcus	Streptococcus sp. oral clone ASCE04; AY953253
			Bacteria	Firmicutes	Bacilli	Lactobacillales	Streptococcaceae	Streptococcus	Streptococcus sp. oral clone ASCF05; AY923140
			Bacteria	Firmicutes	Bacilli	Lactobacillales	Streptococcaceae	Streptococcus	uncultured Streptococcus sp.; 502H08(oral); AM420202
			Bacteria	Firmicutes	Bacilli	Lactobacillales	Streptococcaceae	Streptococcus	uncultured bacterium; BF0002B041; AM697091
			Bacteria	Firmicutes	Bacilli	Lactobacillales	Streptococcaceae	Streptococcus	uncultured bacterium; P4D1-693; EF509615
			Bacteria	Firmicutes	Bacilli	Lactobacillales	Streptococcaceae	Streptococcus	uncultured bacterium; P4D1-501; EF509637
			Bacteria	Firmicutes	Bacilli	Lactobacillales	Streptococcaceae	Streptococcus	uncultured bacterium; P4D1-753; EF509695
			Bacteria	Firmicutes	Bacilli	Lactobacillales	Streptococcaceae	Streptococcus	uncultured bacterium; P4D1-573; EF509712
			Bacteria	Firmicutes	Bacilli	Lactobacillales	Streptococcaceae	Streptococcus	uncultured bacterium; P4D1-552; EF509679
			Bacteria	Firmicutes	Bacilli	Lactobacillales	Streptococcaceae	Streptococcus	uncultured bacterium; P4D1-746; EF509728
			Bacteria	Firmicutes	Bacilli	Lactobacillales	Streptococcaceae	Streptococcus	uncultured bacterium; P4D1-730; EF509745
			Bacteria	Firmicutes	Bacilli	Lactobacillales	Streptococcaceae	Streptococcus	uncultured bacterium; P4D1-751; EF509657
			Bacteria	Firmicutes	Bacilli	Lactobacillales	Streptococcaceae	Streptococcus	uncultured bacterium; P4D1-677; EF509722
			Bacteria	Firmicutes	Bacilli	Lactobacillales	Streptococcaceae	Streptococcus	Streptococcus sp. oral strain B5SC; AY005047
			Bacteria	Firmicutes	Bacilli	Lactobacillales	Streptococcaceae	Streptococcus	uncultured bacterium; P4D1-758; EF509652
			Bacteria	Firmicutes	Bacilli	Lactobacillales	Streptococcaceae	Streptococcus	uncultured bacterium; P4D1-572; EF509782
			Bacteria	Firmicutes	Bacilli	Lactobacillales	Streptococcaceae	Streptococcus	Streptococcus sp. oral clone ASCE10; AY923137
			Bacteria	Firmicutes	Bacilli	Lactobacillales	Streptococcaceae	Streptococcus	Streptococcus cristatus; PD01A5; AJ853835
			Bacteria	Firmicutes	Bacilli	Lactobacillales	Streptococcaceae	Streptococcus	Streptococcus cristatus; 0109 93830B; AJ853836
o1582	o1582 16S-StrOral3	GCAAGAGAAGAACGAGTGTG	Bacteria	Firmicutes	Bacilli	Lactobacillales	Streptococcaceae	Streptococcus	uncultured bacterium; 378; EU531782
			Bacteria	Firmicutes	Bacilli	Lactobacillales	Streptococcaceae	Streptococcus	Streptococcus sp. oral clone CH016; AY005044
			Bacteria	Firmicutes	Bacilli	Lactobacillales	Streptococcaceae	Streptococcus	uncultured bacterium; BF0002C063; AM697366
			Bacteria	Firmicutes	Bacilli	Lactobacillales	Streptococcaceae	Streptococcus	uncultured bacterium; P3D1-456; EF509208
			Bacteria	Firmicutes	Bacilli	Lactobacillales	Streptococcaceae	Streptococcus	uncultured bacterium; nbw01c06c1; GQ060441
			Bacteria	Firmicutes	Bacilli	Lactobacillales	Streptococcaceae	Streptococcus	uncultured bacterium; nbw633h11c1; GQ113099
			Bacteria	Firmicutes	Bacilli	Lactobacillales	Streptococcaceae	Streptococcus	uncultured bacterium; nbu206c09c1; GQ019723
o1583	o1583 16S-StrOral4	ACTTGAATGCAAGAGGGGAG	Bacteria	Firmicutes	Bacilli	Lactobacillales	Streptococcaceae	Streptococcus	Streptococcus sp. oral clone DP009; AF432132
			Bacteria	Firmicutes	Bacilli	Lactobacillales	Streptococcaceae	Streptococcus	uncultured bacterium; TB006; EU432160
o1584	o1584 16S-StrOral5	AATTAAAAGGTGCAATTGCA	Bacteria	Firmicutes	Bacilli	Lactobacillales	Streptococcaceae	Streptococcus	Streptococcus sp. oral clone BW009; AY005042
			Bacteria	Firmicutes	Bacilli	Lactobacillales	Streptococcaceae	Streptococcus	Streptococcus sp. oral clone CH016; AY005044
			Bacteria	Firmicutes	Bacilli	Lactobacillales	Streptococcaceae	Streptococcus	Streptococcus sp. oral clone BM035; AY005043
			Bacteria	Firmicutes	Bacilli	Lactobacillales	Streptococcaceae	Streptococcus	uncultured bacterium; A_S_01_21; FJ470575
			Bacteria	Firmicutes	Bacilli	Lactobacillales	Streptococcaceae	Streptococcus	uncultured Streptococcus sp.; 402A06(oral); AM420134
			Bacteria	Firmicutes	Bacilli	Lactobacillales	Streptococcaceae	Streptococcus	Streptococcus genomosp. C4; C3MLM097; AY278632
			Bacteria	Firmicutes	Bacilli	Lactobacillales	Streptococcaceae	Streptococcus	uncultured bacterium; Oh313710D; EU137442
			Bacteria	Firmicutes	Bacilli	Lactobacillales	Streptococcaceae	Streptococcus	uncultured bacterium; FC01B01; FM873030
			Bacteria	Firmicutes	Bacilli	Lactobacillales	Streptococcaceae	Streptococcus	uncultured bacterium; MA04D09; FM873773
			Bacteria	Firmicutes	Bacilli	Lactobacillales	Streptococcaceae	Streptococcus	Streptococcus sp. oral strain 12F; AY134908
			Bacteria	Firmicutes	Bacilli	Lactobacillales	Streptococcaceae	Streptococcus	Streptococcus mitis; ATCC 6249; AY281077

			Bacteria	Firmicutes	Bacilli	Lactobacillales	Streptococcaceae	Streptococcus	Streptococcus cristatus; ATCC 49999; AY281090
			Bacteria	Firmicutes	Bacilli	Lactobacillales	Streptococcaceae	Streptococcus	uncultured bacterium; nbw985d01c1; GQ045308
			Bacteria	Firmicutes	Bacilli	Lactobacillales	Streptococcaceae	Streptococcus	uncultured bacterium; nbw988f07c1; GQ045650
			Bacteria	Firmicutes	Bacilli	Lactobacillales	Streptococcaceae	Streptococcus	uncultured bacterium; nbw985a05c1; GQ045281
			Bacteria	Firmicutes	Bacilli	Lactobacillales	Streptococcaceae	Streptococcus	uncultured bacterium; nbw985f09c1; GQ045335
			Bacteria	Firmicutes	Bacilli	Lactobacillales	Streptococcaceae	Streptococcus	uncultured bacterium; nbw987d08c1; GQ045456
			Bacteria	Firmicutes	Bacilli	Lactobacillales	Streptococcaceae	Streptococcus	uncultured bacterium; nbw997c09c1; GQ046365
			Bacteria	Firmicutes	Bacilli	Lactobacillales	Streptococcaceae	Streptococcus	uncultured bacterium; nbw988e06c1; GQ045640
			Bacteria	Firmicutes	Bacilli	Lactobacillales	Streptococcaceae	Streptococcus	uncultured bacterium; nbw986e08c1; GQ045394
o1585	o1585 16S-StrPne	AGGTGCACTTGCATCACTAC	Bacteria	Firmicutes	Bacilli	Lactobacillales	Streptococcaceae	Streptococcus	Streptococcus pneumoniae R6; AE008537
			Bacteria	Firmicutes	Bacilli	Lactobacillales	Streptococcaceae	Streptococcus	Streptococcus pneumoniae R6; AE008386
			Bacteria	Firmicutes	Bacilli	Lactobacillales	Streptococcaceae	Streptococcus	Streptococcus pneumoniae R6; AE008546
			Bacteria	Firmicutes	Bacilli	Lactobacillales	Streptococcaceae	Streptococcus	Streptococcus pneumoniae R6; AE008552
			Bacteria	Firmicutes	Bacilli	Lactobacillales	Streptococcaceae	Streptococcus	Streptococcus pneumoniae (T); ATCC 33400; AF003930
			Bacteria	Firmicutes	Bacilli	Lactobacillales	Streptococcaceae	Streptococcus	Streptococcus sp. 2061A; AF316591
			Bacteria	Firmicutes	Bacilli	Lactobacillales	Streptococcaceae	Streptococcus	Streptococcus pneumoniae; ATCC 33400; AY485600
			Bacteria	Firmicutes	Bacilli	Lactobacillales	Streptococcaceae	Streptococcus	Streptococcus pneumoniae D39; CP000410
			Bacteria	Firmicutes	Bacilli	Lactobacillales	Streptococcaceae	Streptococcus	Streptococcus pneumoniae D39; CP000410
			Bacteria	Firmicutes	Bacilli	Lactobacillales	Streptococcaceae	Streptococcus	Streptococcus pneumoniae D39; CP000410
			Bacteria	Firmicutes	Bacilli	Lactobacillales	Streptococcaceae	Streptococcus	Streptococcus pneumoniae D39; CP000410
			Bacteria	Firmicutes	Bacilli	Lactobacillales	Streptococcaceae	Streptococcus	Streptococcus pneumoniae Hungary19A-6; CP000936
			Bacteria	Firmicutes	Bacilli	Lactobacillales	Streptococcaceae	Streptococcus	Streptococcus pneumoniae Hungary19A-6; CP000936
			Bacteria	Firmicutes	Bacilli	Lactobacillales	Streptococcaceae	Streptococcus	Streptococcus pneumoniae; ATCC BAA-334; AY281083
			Bacteria	Firmicutes	Bacilli	Lactobacillales	Streptococcaceae	Streptococcus	Streptococcus pneumoniae; ATCC 700669; AY281081
			Bacteria	Firmicutes	Bacilli	Lactobacillales	Streptococcaceae	Streptococcus	Streptococcus pneumoniae; ATCC 49619; AY281082
			Bacteria	Firmicutes	Bacilli	Lactobacillales	Streptococcaceae	Streptococcus	Streptococcus pneumoniae; ATCC 6303; AY281084
			Bacteria	Firmicutes	Bacilli	Lactobacillales	Streptococcaceae	Streptococcus	Streptococcus pneumoniae; MAFF 911410; AB002522
			Bacteria	Firmicutes	Bacilli	Lactobacillales	Streptococcaceae	Streptococcus	Streptococcus pneumoniae; HK_P47; AY525789
			Bacteria	Firmicutes	Bacilli	Lactobacillales	Streptococcaceae	Streptococcus	Streptococcus pneumoniae; NCTC 7465(T); X58312
o1586	o1586 16S-StrSob	TTGTAGGGGAAGAACGTGTG	Bacteria	Firmicutes	Bacilli	Lactobacillales	Streptococcaceae	Streptococcus	Streptococcus sobrinus; ATCC 33478; AY188349
			Bacteria	Firmicutes	Bacilli	Lactobacillales	Streptococcaceae	Streptococcus	Streptococcus sobrinus; ChDC YS207; PD1021; DQ677793
			Bacteria	Firmicutes	Bacilli	Lactobacillales	Streptococcaceae	Streptococcus	Streptococcus sobrinus; ChDC YS13; 40125; DQ677799
			Bacteria	Firmicutes	Bacilli	Lactobacillales	Streptococcaceae	Streptococcus	Streptococcus sobrinus; ChDC YS12; 40225; DQ677800
			Bacteria	Firmicutes	Bacilli	Lactobacillales	Streptococcaceae	Streptococcus	Streptococcus sobrinus; ChDC YS205; PD1010; DQ677795
			Bacteria	Firmicutes	Bacilli	Lactobacillales	Streptococcaceae	Streptococcus	Streptococcus sobrinus; ChDC YS11; 45111; DQ677802
			Bacteria	Firmicutes	Bacilli	Lactobacillales	Streptococcaceae	Streptococcus	Streptococcus sobrinus; ChDC YS211; PD1164; DQ677790
			Bacteria	Firmicutes	Bacilli	Lactobacillales	Streptococcaceae	Streptococcus	Streptococcus sobrinus; ChDC YS209; PD1051; DQ677792
			Bacteria	Firmicutes	Bacilli	Lactobacillales	Streptococcaceae	Streptococcus	Streptococcus sobrinus; ChDC YS203; PD57; DQ677796
			Bacteria	Firmicutes	Bacilli	Lactobacillales	Streptococcaceae	Streptococcus	Streptococcus sobrinus; ChDC YS201; PD14; DQ677798
			Bacteria	Firmicutes	Bacilli	Lactobacillales	Streptococcaceae	Streptococcus	Streptococcus sobrinus; ChDC YS210; PD1135; DQ677791
			Bacteria	Firmicutes	Bacilli	Lactobacillales	Streptococcaceae	Streptococcus	Streptococcus sobrinus; ChDC YS212; PD1109; DQ677789
			Bacteria	Firmicutes	Bacilli	Lactobacillales	Streptococcaceae	Streptococcus	Streptococcus sobrinus; ChDC YS5; 58141; DQ677805
			Bacteria	Firmicutes	Bacilli	Lactobacillales	Streptococcaceae	Streptococcus	Streptococcus sobrinus; ChDC YS1; AY691533
			Bacteria	Firmicutes	Bacilli	Lactobacillales	Streptococcaceae	Streptococcus	Streptococcus sobrinus; ChDC YS206; PD1017; DQ677794
			Bacteria	Firmicutes	Bacilli	Lactobacillales	Streptococcaceae	Streptococcus	Streptococcus sobrinus; ChDC YS7; 59142; DQ677803
			Bacteria	Firmicutes	Bacilli	Lactobacillales	Streptococcaceae	Streptococcus	Streptococcus sobrinus; ChDC YS6; 59111; DQ677804
			Bacteria	Firmicutes	Bacilli	Lactobacillales	Streptococcaceae	Streptococcus	Streptococcus sobrinus; ChDC YS202; PD54; DQ677797
			Bacteria	Firmicutes	Bacilli	Lactobacillales	Streptococcaceae	Streptococcus	Streptococcus sobrinus; NCTC 12279; AJ243966
			Bacteria	Firmicutes	Bacilli	Lactobacillales	Streptococcaceae	Streptococcus	Streptococcus sobrinus; 6715; AF439398
o1587	o1587 16S-StSpp1	CACTCTCCCCTTCTGCAC	Bacteria	Firmicutes	Bacilli	Lactobacillales	Streptococcaceae	Streptococcus	Streptococcus mutans; AF199; AF139602
			Bacteria	Firmicutes	Bacilli	Lactobacillales	Streptococcaceae	Streptococcus	Streptococcus mutans; UA140; AF139599
			Bacteria	Firmicutes	Bacilli	Lactobacillales	Streptococcaceae	Streptococcus	unidentified bacterium; T44; Z94011
			Bacteria	Firmicutes	Bacilli	Lactobacillales	Streptococcaceae	Streptococcus	Streptococcus thermophilus (T); DSM 20617; X68418
			Bacteria	Firmicutes	Bacilli	Lactobacillales	Streptococcaceae	Streptococcus	Streptococcus uberis; HN1; AB023576
			Bacteria	Firmicutes	Bacilli	Lactobacillales	Streptococcaceae	Streptococcus	Streptococcus sp.; 2285-97; AJ131965
			Bacteria	Firmicutes	Bacilli	Lactobacillales	Streptococcaceae	Streptococcus	Streptococcus suis; 194; AB071340
			Bacteria	Firmicutes	Bacilli	Lactobacillales	Streptococcaceae	Streptococcus	Streptococcus suis; DAT2; AB071337
			Bacteria	Firmicutes	Bacilli	Lactobacillales	Streptococcaceae	Streptococcus	Streptococcus suis; 220; AB071345
			Bacteria	Firmicutes	Bacilli	Lactobacillales	Streptococcaceae	Streptococcus	Streptococcus suis; 243; AB071350
			Bacteria	Firmicutes	Bacilli	Lactobacillales	Streptococcaceae	Streptococcus	Streptococcus suis; 226; AB071347
			Bacteria	Firmicutes	Bacilli	Lactobacillales	Streptococcaceae	Streptococcus	Streptococcus suis; 210; AB071343
			Bacteria	Firmicutes	Bacilli	Lactobacillales	Streptococcaceae	Streptococcus	Streptococcus canis; CG40; AJ413205
			Bacteria	Firmicutes	Bacilli	Lactobacillales	Streptococcaceae	Streptococcus	Streptococcus dysgalactiae subsp. dysgalactiae; HKU7; AF433167
			Bacteria	Firmicutes	Bacilli	Lactobacillales	Streptococcaceae	Streptococcus	Streptococcus pasteuri; NEM1202, CIP 105070; AJ297216
			Bacteria	Firmicutes	Bacilli	Lactobacillales	Streptococcaceae	Streptococcus	Streptococcus sobrinus; NCTC 12279; AJ243966
			Bacteria	Firmicutes	Bacilli	Lactobacillales	Streptococcaceae	Streptococcus	Streptococcus pluranimalium; LMG 14257; Y18026
			Bacteria	Firmicutes	Bacilli	Lactobacillales	Streptococcaceae	Streptococcus	Streptococcus thoraltensis (T); S69; Y09007
			Bacteria	Firmicutes	Bacilli	Lactobacillales	Streptococcaceae	Streptococcus	Streptococcus gallolyticus; ACM3968; Y10869
			Bacteria	Firmicutes	Bacilli	Lactobacillales	Streptococcaceae	Streptococcus	Streptococcus dysgalactiae subsp. dysgalactiae; NCFB1356; AB008926
o1588	o1588 16S-StSpp2	CCACTCTCCCCTTCTGCAC	Bacteria	Firmicutes	Bacilli	Lactobacillales	Streptococcaceae	Streptococcus	Streptococcus mutans; AF199; AF139602
			Bacteria	Firmicutes	Bacilli	Lactobacillales	Streptococcaceae	Streptococcus	Streptococcus mutans; UA140; AF139599
			Bacteria	Firmicutes	Bacilli	Lactobacillales	Streptococcaceae	Streptococcus	unidentified bacterium; T44; Z94011
			Bacteria	Firmicutes	Bacilli	Lactobacillales	Streptococcaceae	Streptococcus	Streptococcus thermophilus (T); DSM 20617; X68418
			Bacteria	Firmicutes	Bacilli	Lactobacillales	Streptococcaceae	Streptococcus	Streptococcus uberis; HN1; AB023576
			Bacteria	Firmicutes	Bacilli	Lactobacillales	Streptococcaceae	Streptococcus	Streptococcus sp.; 2285-97; AJ131965
			Bacteria	Firmicutes	Bacilli	Lactobacillales	Streptococcaceae	Streptococcus	Streptococcus suis; 194; AB071340
			Bacteria	Firmicutes	Bacilli	Lactobacillales	Streptococcaceae	Streptococcus	Streptococcus suis; DAT2; AB071337
			Bacteria	Firmicutes	Bacilli	Lactobacillales	Streptococcaceae	Streptococcus	Streptococcus suis; 220; AB071345
			Bacteria	Firmicutes	Bacilli	Lactobacillales	Streptococcaceae	Streptococcus	Streptococcus suis; 243; AB071350
			Bacteria	Firmicutes	Bacilli	Lactobacillales	Streptococcaceae	Streptococcus	Streptococcus suis; 226; AB071347
			Bacteria	Firmicutes	Bacilli	Lactobacillales	Streptococcaceae	Streptococcus	Streptococcus suis; 210; AB071343
			Bacteria	Firmicutes	Bacilli	Lactobacillales	Streptococcaceae	Streptococcus	Streptococcus canis; CG40; AJ413205
			Bacteria	Firmicutes	Bacilli	Lactobacillales	Streptococcaceae	Streptococcus	Streptococcus dysgalactiae subsp. dysgalactiae; HKU7; AF433167
			Bacteria	Firmicutes	Bacilli	Lactobacillales	Streptococcaceae	Streptococcus	Streptococcus pasteuri; NEM1202, CIP 105070; AJ297216
			Bacteria	Firmicutes	Bacilli	Lactobacillales	Streptococcaceae	Streptococcus	Streptococcus sobrinus; NCTC 12279; AJ243966
			Bacteria	Firmicutes	Bacilli	Lactobacillales	Streptococcaceae	Streptococcus	Streptococcus pluranimalium; LMG 14257; Y18026
			Bacteria	Firmicutes	Bacilli	Lactobacillales	Streptococcaceae	Streptococcus	Streptococcus thoraltensis (T); S69; Y09007
			Bacteria	Firmicutes	Bacilli	Lactobacillales	Streptococcaceae	Streptococcus	Streptococcus gallolyticus; ACM3968; Y10869

			Bacteria	Firmicutes	Bacilli	Lactobacillales	Streptococcaceae	Streptococcus	Streptococcus dysgalactiae subsp. dysgalactiae; NCFB1356; AB008926
o1589	o1589 16S-TanFor	GCCGTTGAAACTGGTAGTCT	Bacteria	Bacteroidetes	Bacteroidetes	Bacteroidales	unclassified_Bacteroidales		uncultured rumen bacterium; SR15; DQ394625
			Bacteria	Bacteroidetes	Bacteroidetes	Bacteroidales	Porphyromonadaceae	Porphyromonas	Porphyromonas canis; JCM 10100; AB034799
			Bacteria	Bacteroidetes	Flavobacteria	Flavobacteriales	Flavobacteriaceae	unclassified_Flavobacteriaceae	uncultured bacterium; CS399; AY382614
			Bacteria	Bacteroidetes	Bacteroidetes	Bacteroidales	Porphyromonadaceae	Tannerella	Tannerella forsythensis; Ko3; AB053943
			Bacteria	Bacteroidetes	Bacteroidetes	Bacteroidales	Porphyromonadaceae	Tannerella	Tannerella forsythensis; KS16; AB053944
			Bacteria	Bacteroidetes	Bacteroidetes	Bacteroidales	Porphyromonadaceae	Tannerella	Tannerella forsythensis; TR6; AB053947
			Bacteria	Bacteroidetes	Bacteroidetes	Bacteroidales	Porphyromonadaceae	Tannerella	Tannerella forsythensis; Sai5; AB053946
			Bacteria	Bacteroidetes	Bacteroidetes	Bacteroidales	Porphyromonadaceae	Tannerella	Tannerella forsythensis; L7; AB053945
			Bacteria	Bacteroidetes	Bacteroidetes	Bacteroidales	Porphyromonadaceae	Tannerella	Tannerella forsythensis; G9; AB053939
			Bacteria	Bacteroidetes	Bacteroidetes	Bacteroidales	Porphyromonadaceae	Tannerella	Tannerella forsythensis; KM3; AB053942
			Bacteria	Bacteroidetes	Bacteroidetes	Bacteroidales	Porphyromonadaceae	Tannerella	Tannerella forsythensis; FJ1; AB053938
			Bacteria	Bacteroidetes	Bacteroidetes	Bacteroidales	Porphyromonadaceae	Tannerella	Tannerella forsythensis; HA3; AB053940
			Bacteria	Bacteroidetes	Bacteroidetes	Bacteroidales	Porphyromonadaceae	Tannerella	Tannerella forsythensis; HG3; AB053941
			Bacteria	Bacteroidetes	Bacteroidetes	Bacteroidales	Porphyromonadaceae	Tannerella	Tannerella forsythensis; RMA 8563; DQ341410
			Bacteria	Bacteroidetes	Bacteroidetes	Bacteroidales	Porphyromonadaceae	Tannerella	Tannerella forsythensis; RMA 7251; DQ344914
			Bacteria	Bacteroidetes	Bacteroidetes	Bacteroidales	Porphyromonadaceae	Tannerella	Tannerella forsythensis; FDC 331; X73962
			Bacteria	Bacteroidetes	Bacteroidetes	Bacteroidales	Porphyromonadaceae	Tannerella	Tannerella forsythensis; ATCC43037; AB035460
			Bacteria	Bacteroidetes	Bacteroidetes	Bacteroidales	Porphyromonadaceae	Tannerella	Tannerella forsythensis (T); 338; L16495
			Bacteria	Bacteroidetes	Bacteroidetes	Bacteroidales	Porphyromonadaceae	Porphyromonas	Porphyromonas circumdentaria (T); L26102
			Bacteria	Bacteroidetes	Bacteroidetes	Bacteroidales	Porphyromonadaceae	Porphyromonas	Porphyromonas gingivicanis; ATCC 55562; DQ677835
o1591	o1591 16S-TM72	AAGGAACACCAATGGCGTAG	Bacteria	TM7				TM7_genera_incertae_sedis	uncultured bacterium SBR2096; AF268998
			Bacteria	TM7				TM7_genera_incertae_sedis	uncultured candidate division TM7 bacterium; SM1G12; AF445701
			Bacteria	TM7				TM7_genera_incertae_sedis	uncultured bacterium; Blxi1; AJ318200
			Bacteria	TM7				TM7_genera_incertae_sedis	uncultured bacterium oral clone BS003; AY005448
			Bacteria	TM7				TM7_genera_incertae_sedis	uncultured bacterium; Blgi18; AJ318136
			Bacteria	TM7				TM7_genera_incertae_sedis	uncultured bacterium oral clone BE109; AY005446
			Bacteria	TM7				TM7_genera_incertae_sedis	uncultured bacterium F061; AF125205
			Bacteria	TM7				TM7_genera_incertae_sedis	uncultured soil bacterium; C026; AF507686
			Bacteria	TM7				TM7_genera_incertae_sedis	uncultured bacterium; PeM47; AJ576410
			Bacteria	TM7				TM7_genera_incertae_sedis	uncultured bacterium; MTAC17; AJ619043
			Bacteria	TM7				TM7_genera_incertae_sedis	uncultured soil bacterium; G7-1250-5; AF525833
			Bacteria	TM7				TM7_genera_incertae_sedis	uncultured soil bacterium; G7-1244-5; AF525832
			Bacteria	TM7				TM7_genera_incertae_sedis	uncultured bacterium; C-CM46; AY622231
			Bacteria	TM7				TM7_genera_incertae_sedis	uncultured bacterium; 734; AF513103
			Bacteria	TM7				TM7_genera_incertae_sedis	uncultured bacterium; 8A; AF513108
			Bacteria	TM7				TM7_genera_incertae_sedis	uncultured soil bacterium; G7-1288-5; AF525834
			Bacteria	TM7				TM7_genera_incertae_sedis	uncultured bacterium; 71; AF513102
			Bacteria	TM7				TM7_genera_incertae_sedis	uncultured bacterium; 75; AF513104
			Bacteria	TM7				TM7_genera_incertae_sedis	uncultured bacterium; 54; AJ538353
			Bacteria	TM7				TM7_genera_incertae_sedis	uncultured bacterium; 220; AJ538354
o1592	o1592 16S-TM73	TTACTGGGCGTAAAGAGTTG	Bacteria	TM7				TM7_genera_incertae_sedis	metal-contaminated soil clone K20-27; AF145827
			Bacteria	TM7				TM7_genera_incertae_sedis	metal-contaminated soil clone K20-12; AF145815
			Bacteria	TM7				TM7_genera_incertae_sedis	uncultured candidate division TM7 bacterium; AT-s2-18; AY225653
			Bacteria	Actinobacteria	Actinobacteria	Actinomycetales	Nocardiaceae	Rhodococcus	bacterium W14; AY345526
			Bacteria	TM7				TM7_genera_incertae_sedis	uncultured soil bacterium; C1105; AF507688
			Bacteria	TM7				TM7_genera_incertae_sedis	unidentified bacterium; W4-B20; AY345503
			Bacteria	TM7				TM7_genera_incertae_sedis	uncultured bacterium SBR2060; AF268999
			Bacteria	TM7				TM7_genera_incertae_sedis	uncultured bacterium; Blgi18; AJ318136
			Bacteria	TM7				TM7_genera_incertae_sedis	uncultured bacterium F061; AF125205
			Bacteria	TM7				TM7_genera_incertae_sedis	uncultured soil bacterium; C129; AF507687
			Bacteria	TM7				TM7_genera_incertae_sedis	uncultured bacterium SBR2013; AF269000
			Bacteria	TM7				TM7_genera_incertae_sedis	uncultured bacterium; PeM47; AJ576410
			Bacteria	TM7				TM7_genera_incertae_sedis	uncultured bacterium SBR1071; AF268996
			Bacteria	TM7				TM7_genera_incertae_sedis	uncultured soil bacterium; S1197; AF507689
			Bacteria	TM7				TM7_genera_incertae_sedis	uncultured bacterium SBR2004; AF268997
			Bacteria	TM7				TM7_genera_incertae_sedis	uncultured bacterium GC1; AF268994
			Bacteria	TM7				TM7_genera_incertae_sedis	uncultured bacterium; C-CM46; AY622231
			Bacteria	TM7				TM7_genera_incertae_sedis	uncultured bacterium; 54; AJ538353
			Bacteria	TM7				TM7_genera_incertae_sedis	TM7 phylum sp. oral clone BU080; AF385568
			Bacteria	TM7				TM7_genera_incertae_sedis	uncultured bacterium; FW73; AF524021
o1594	o1594 16S-TreBuc	TGCCCGGGACAGCCTGTA	Bacteria	Spirochaetes	Spirochaetes	Spirochaetales	Spirochaetaceae	Treponema	Treponema Group 6 sp. oral clone IL034; AY349418
			Bacteria	Spirochaetes	Spirochaetes	Spirochaetales	Spirochaetaceae	Treponema	Treponema socranskii subsp. socranskii subsp. '04'; D40DR-2; AY369246
			Bacteria	Spirochaetes	Spirochaetes	Spirochaetales	Spirochaetaceae	Treponema	Treponema socranskii subsp. buccale; OMZ 884; AY369255
			Bacteria	Spirochaetes	Spirochaetes	Spirochaetales	Spirochaetaceae	Treponema	Treponema sp. 6:H:D15A-4; AY005083
			Bacteria	Spirochaetes	Spirochaetes	Spirochaetales	Spirochaetaceae	Treponema	Treponema sp. Smibert-5; D120CR-1; AF033303
			Bacteria	Spirochaetes	Spirochaetes	Spirochaetales	Spirochaetaceae	Treponema	Treponema socranskii subsp. buccale (T); ATCC 35534; AF033305
			Bacteria	Spirochaetes	Spirochaetes	Spirochaetales	Spirochaetaceae	Treponema	Treponema socranskii subsp. buccale; B418; AB015892
			Bacteria	Spirochaetes	Spirochaetes	Spirochaetales	Spirochaetaceae	Treponema	Treponema socranskii subsp. socranskii subsp. '04'; D11B-2; AF033308
			Bacteria	Spirochaetes	Spirochaetes	Spirochaetales	Spirochaetaceae	Treponema	Treponema socranskii subsp. buccale; T10; AB015893
			Bacteria	Spirochaetes	Spirochaetes	Spirochaetales	Spirochaetaceae	Treponema	uncultured Treponema sp.; 10B576; FJ976311
			Bacteria	Spirochaetes	Spirochaetes	Spirochaetales	Spirochaetaceae	Treponema	uncultured Treponema sp.; 24B645; FJ976406
o1595	o1595 16S-TreDen	AAGTCGAACGGTAAGGGACA	Bacteria	Spirochaetes	Spirochaetes	Spirochaetales	Spirochaetaceae	Treponema	Treponema denticola; ATCC35405; AF139203
o1596	o1596 16S-TreGroup1	ACCCTGCTAGACAGGTGCT	Bacteria	Spirochaetes	Spirochaetes	Spirochaetales	Spirochaetaceae	Treponema	Treponema vincentii; OMZ 860; AY369251
			Bacteria	Spirochaetes	Spirochaetes	Spirochaetales	Spirochaetaceae	Treponema	Treponema vincentii; OMZ 861; AY369252
			Bacteria	Spirochaetes	Spirochaetes	Spirochaetales	Spirochaetaceae	Treponema	Treponema vincentii; OMZ 862; AY369253
			Bacteria	Spirochaetes	Spirochaetes	Spirochaetales	Spirochaetaceae	Treponema	Treponema vincentii; OMZ 858; AY369250
			Bacteria	Spirochaetes	Spirochaetes	Spirochaetales	Spirochaetaceae	Treponema	Treponema sp.; ovine footrot; AJ010951
			Bacteria	Spirochaetes	Spirochaetes	Spirochaetales	Spirochaetaceae	Treponema	Treponema sp. 1:AA:DW001 oral clone DW001; AY005082
			Bacteria	Spirochaetes	Spirochaetes	Spirochaetales	Spirochaetaceae	Treponema	Treponema sp. I:T:AT24; AF061350
			Bacteria	Spirochaetes	Spirochaetes	Spirochaetales	Spirochaetaceae	Treponema	Treponema sp. I:S:AT39; AF061349
			Bacteria	Spirochaetes	Spirochaetes	Spirochaetales	Spirochaetaceae	Treponema	Treponema vincentii; ATCC 35580; AF033309
			Bacteria	Spirochaetes	Spirochaetes	Spirochaetales	Spirochaetaceae	Treponema	Treponema sp. I:8:G57; I:P:G57; AF056337
			Bacteria	Spirochaetes	Spirochaetes	Spirochaetales	Spirochaetaceae	Treponema	Treponema genomosp. P1; P4GB_42; AY341822
			Bacteria	Spirochaetes	Spirochaetes	Spirochaetales	Spirochaetaceae	Treponema	Treponema sp. I:G:C1; AF023052
			Bacteria	Spirochaetes	Spirochaetes	Spirochaetales	Spirochaetaceae	Treponema	Treponema sp. I:B:C7; AF023051
			Bacteria	Spirochaetes	Spirochaetes	Spirochaetales	Spirochaetaceae	Treponema	Treponema sp. I:G:T21; AF023055
			Bacteria	Spirochaetes	Spirochaetes	Spirochaetales	Spirochaetaceae	Treponema	Treponema vincentii; N9; ATCC 700013; AF033310

			Bacteria	Spirochaetes	Spirochaetes	Spirochaetales	Spirochaetaceae	Treponema	uncultured eubacterium E1-K2; AJ289172
			Bacteria	Spirochaetes	Spirochaetes	Spirochaetales	Spirochaetaceae	Treponema	uncultured eubacterium E1-K1; AJ289178
			Bacteria	Spirochaetes	Spirochaetes	Spirochaetales	Spirochaetaceae	Treponema	Treponema sp. UB1467; AY119691
			Bacteria	Spirochaetes	Spirochaetes	Spirochaetales	Spirochaetaceae	Treponema	Treponema vincentii; D2A-2; AY119690
			Bacteria	Spirochaetes	Spirochaetes	Spirochaetales	Spirochaetaceae	Treponema	Treponema sp.; DDKL-13; Y08896
o1597	o1597 16S-TreGroup2	AGCGTAGCAATACGAGGTAC	Bacteria	Spirochaetes	Spirochaetes	Spirochaetales	Spirochaetaceae	Treponema	Treponema sp. OMZ 840; AY369248
			Bacteria	Spirochaetes	Spirochaetes	Spirochaetales	Spirochaetaceae	Treponema	uncultured bacterium; A_D_03_61; FJ470506
			Bacteria	Spirochaetes	Spirochaetes	Spirochaetales	Spirochaetaceae	Treponema	Treponema sp. OMZ 839; AY369247
			Bacteria	Spirochaetes	Spirochaetes	Spirochaetales	Spirochaetaceae	Treponema	Treponema sp. oral clone JU025; Ju025; AY349417
			Bacteria	Spirochaetes	Spirochaetes	Spirochaetales	Spirochaetaceae	Treponema	Treponema maltophilum (T); periodontitis patient BR; X87140
			Bacteria	Spirochaetes	Spirochaetes	Spirochaetales	Spirochaetaceae	Treponema	uncultured Treponema sp.; 701E07(oral); AM420254
			Bacteria	Spirochaetes	Spirochaetes	Spirochaetales	Spirochaetaceae	Treponema	Treponema sp. IV:17B:C21; AF023038
			Bacteria	Spirochaetes	Spirochaetes	Spirochaetales	Spirochaetaceae	Treponema	Treponema sp. IV:17E:D36; AF056342
			Bacteria	Spirochaetes	Spirochaetes	Spirochaetales	Spirochaetaceae	Treponema	Treponema sp. IV:17D:D31; AF023041
			Bacteria	Spirochaetes	Spirochaetes	Spirochaetales	Spirochaetaceae	Treponema	Treponema sp. IV:18:C9; AF023042
			Bacteria	Spirochaetes	Spirochaetes	Spirochaetales	Spirochaetaceae	Treponema	uncultured Treponema sp.; 10B277; FJ976302
o1598	o1598 16S-TreGroup3	AAAGCTGGGTGCCAAAGCAA	Bacteria	Spirochaetes	Spirochaetes	Spirochaetales	Spirochaetaceae	Treponema	Treponema sp. IV:17C:GF6; AF023040
			Bacteria	Spirochaetes	Spirochaetes	Spirochaetales	Spirochaetaceae	Treponema	Treponema sp. OMZ 839; AY369247
			Bacteria	Spirochaetes	Spirochaetes	Spirochaetales	Spirochaetaceae	Treponema	Treponema lecithinolyticum; ATCC 700332; PFB4G; AJ131282
			Bacteria	Spirochaetes	Spirochaetes	Spirochaetales	Spirochaetaceae	Treponema	Treponema lecithinolyticum (T); periodontitis patient PFB4G; X87139
o1599	o1599 16S-TreGroup4	AAATGAGGAAAGGAGCTACG	Bacteria	Spirochaetes	Spirochaetes	Spirochaetales	Spirochaetaceae	Treponema	uncultured bacterium; CLOSED_ROOT_A24; FJ983017
			Bacteria	Spirochaetes	Spirochaetes	Spirochaetales	Spirochaetaceae	Treponema	Treponema denticola; ATCC35405; AF139203
			Bacteria	Spirochaetes	Spirochaetes	Spirochaetales	Spirochaetaceae	Treponema	Treponema denticola ATCC 35405; AE017226
			Bacteria	Spirochaetes	Spirochaetes	Spirochaetales	Spirochaetaceae	Treponema	Treponema denticola ATCC 35405; AE017226
			Bacteria	Spirochaetes	Spirochaetes	Spirochaetales	Spirochaetaceae	Treponema	uncultured Treponema sp.; 502B04(oral); AM420186
			Bacteria	Spirochaetes	Spirochaetes	Spirochaetales	Spirochaetaceae	Treponema	Treponema genomosp. P2; P2PB_60; AY345159
			Bacteria	Spirochaetes	Spirochaetes	Spirochaetales	Spirochaetaceae	Treponema	Treponema sp. oral clone P2PB_53 P3; AY207055
			Bacteria	Spirochaetes	Spirochaetes	Spirochaetales	Spirochaetaceae	Treponema	Treponema sp. II:11:U61; II:E:U61; AF023033
			Bacteria	Spirochaetes	Spirochaetes	Spirochaetales	Spirochaetaceae	Treponema	Treponema sp. II:10:D12; AF023030
			Bacteria	Spirochaetes	Spirochaetes	Spirochaetales	Spirochaetaceae	Treponema	Treponema denticola (T); ATCC35404; AF139204
			Bacteria	Spirochaetes	Spirochaetes	Spirochaetales	Spirochaetaceae	Treponema	Treponema denticola II:11:33520; II:11:33520; ATCC 33520; M71236
			Bacteria	Spirochaetes	Spirochaetes	Spirochaetales	Spirochaetaceae	Treponema	Treponema sp. PT7; AM942451
			Bacteria	Spirochaetes	Spirochaetes	Spirochaetales	Spirochaetaceae	Treponema	Treponema denticola; ATCC33520; D85438
			Bacteria	Spirochaetes	Spirochaetes	Spirochaetales	Spirochaetaceae	Treponema	uncultured Treponema sp.; 24B351; FJ976397
			Bacteria	Spirochaetes	Spirochaetes	Spirochaetales	Spirochaetaceae	Treponema	Treponema denticola; GM-1; AY119692
o1600	o1600 16S-TreSoc	TGGACGGCGGGGTAAAGG	Bacteria	Spirochaetes	Spirochaetes	Spirochaetales	Spirochaetaceae	Treponema	Treponema socranskii subsp. socranskii (T); D56BRIII6, ATCC 35536; AF033306
			Bacteria	Spirochaetes	Spirochaetes	Spirochaetales	Spirochaetaceae	Spirochaeta	Spirochaeta sp. MET-E; AY800103
			Bacteria	Spirochaetes	Spirochaetes	Spirochaetales	Spirochaetaceae	Treponema	Treponema socranskii subsp. socranskii subsp. '04'; D40DR-2; AY369246
			Bacteria	Spirochaetes	Spirochaetes	Spirochaetales	Spirochaetaceae	Treponema	Treponema socranskii subsp. buccale; OMZ 884; AY369255
			Bacteria	Spirochaetes	Spirochaetes	Spirochaetales	Spirochaetaceae	Treponema	Treponema sp. 6:H:D15A-4; AY005083
			Bacteria	Spirochaetes	Spirochaetes	Spirochaetales	Spirochaetaceae	Spirochaeta	uncultured Spirochaetaceae bacterium; TCB129x; DQ647164
			Bacteria	Spirochaetes	Spirochaetes	Spirochaetales	Spirochaetaceae	Treponema	Treponema socranskii subsp. socranskii; 122C; AB015887
			Bacteria	Spirochaetes	Spirochaetes	Spirochaetales	Spirochaetaceae	Treponema	Treponema socranskii subsp. socranskii; 128B; AB015889
			Bacteria	Spirochaetes	Spirochaetes	Spirochaetales	Spirochaetaceae	Treponema	Treponema socranskii subsp. socranskii; 1267; AB015890
			Bacteria	Spirochaetes	Spirochaetes	Spirochaetales	Spirochaetaceae	Spirochaeta	uncultured Spirochaetes bacterium; D004011N01; EU721781
			Bacteria	Spirochaetes	Spirochaetes	Spirochaetales	Spirochaetaceae	Treponema	Treponema socranskii subsp. socranskii; 1166; AB015888
			Bacteria	Spirochaetes	Spirochaetes	Spirochaetales	Spirochaetaceae	Spirochaeta	uncultured bacterium; LC65; FJ024715
			Bacteria	Spirochaetes	Spirochaetes	Spirochaetales	Spirochaetaceae	Spirochaeta	uncultured bacterium; L29; FJ024708
o1601	o1601 16S-TreSpe	TTACTACACCGCTAGACAGG	Bacteria	Spirochaetes	Spirochaetes	Spirochaetales	Spirochaetaceae	Treponema	Treponema sp. 1:Z:AF068 oral clone AF068; AY005081
			Bacteria	Spirochaetes	Spirochaetes	Spirochaetales	Spirochaetaceae	Treponema	uncultured Treponema sp.; 702B11(oral); AM420273
			Bacteria	Spirochaetes	Spirochaetes	Spirochaetales	Spirochaetaceae	Treponema	Treponema sp. I:F:D13; AF023053
			Bacteria	Spirochaetes	Spirochaetes	Spirochaetales	Spirochaetaceae	Treponema	Treponema sp. I:U:Y9; AF061351
o1602	o1602 16S-VaGroup1	GCCCTTCAGTGCTGCAGTA	Bacteria	Firmicutes	Bacilli	Lactobacillales	Enterococcaceae	Vagococcus	Vagococcus elongatus; PPC9; AF445297
			Bacteria	Firmicutes	Bacilli	Lactobacillales	Enterococcaceae	Vagococcus	uncultured bacterium; A0-pApH-106; EU878340
			Bacteria	Firmicutes	Bacilli	Lactobacillales	Enterococcaceae	Vagococcus	Vagococcus sp. SSCS12; AB211029
			Bacteria	Firmicutes	Bacilli	Bacillales	Bacillaceae	Bacillus f	uncultured Bacillus sp.; 84-6; AF526932
			Bacteria	Firmicutes	Bacilli	Lactobacillales	Enterococcaceae	Vagococcus	Vagococcus lutrae; F01595; DQ395281
			Bacteria	Firmicutes	Bacilli	Lactobacillales	Enterococcaceae	Vagococcus	Vagococcus lutrae (T); m1134/97/1; CCUG 39187; Y17152
			Bacteria	Firmicutes	Bacilli	Lactobacillales	Enterococcaceae	Vagococcus	swine fecal bacterium RF1A-Cel11; FJ753801
			Bacteria	Firmicutes	Bacilli	Lactobacillales	Enterococcaceae	Vagococcus	Vagococcus sp. AC-1; FJ211190
			Bacteria	Firmicutes	Bacilli	Lactobacillales	Carnobacteriaceae	Granulicatella	uncultured bacterium; nbu189d05c1; GQ019383
			Bacteria	Firmicutes	Bacilli	Bacillales	Bacillaceae	Bacillus f	uncultured bacterium; SOY105; FJ415722
o1603	o1603 16S-VeGroup1	CGGAATAGAAAGCCACGGC	Bacteria	Firmicutes	Clostridia	Clostridiales	Veillonellaceae	Veillonella	Veillonella montpellierensis; 2001-112662; AY244769
			Bacteria	Firmicutes	Clostridia	Clostridiales	Veillonellaceae	Veillonella	Veillonella sp. oral clone X042; AF287781
			Bacteria	Firmicutes	Clostridia	Clostridiales	Veillonellaceae	Veillonella	uncultured Veillonella sp.; BU083; AF366266
			Bacteria	Firmicutes	Clostridia	Clostridiales	Veillonellaceae	Veillonella	Veillonella dispar (T); DSM 20735; X84006
			Bacteria	Firmicutes	Clostridia	Clostridiales	Veillonellaceae	Veillonella	Veillonella sp. oral clone AA050; AF287782
			Bacteria	Firmicutes	Clostridia	Clostridiales	Veillonellaceae	Veillonella	Veillonella atypica (T); DSM 20739; X84007
			Bacteria	Firmicutes	Clostridia	Clostridiales	Veillonellaceae	Veillonella	Veillonella ratti; ATCC17746; AF186071
			Bacteria	Firmicutes	Clostridia	Clostridiales	Veillonellaceae	Veillonella	Veillonella criceti (T); ATCC17747; AF186072
			Bacteria	Firmicutes	Clostridia	Clostridiales	Veillonellaceae	Veillonella	uncultured bacterium; p-5279-2Wa3; AF371939
			Bacteria	Firmicutes	Clostridia	Clostridiales	Veillonellaceae	Veillonella	Veillonella parvula (T); DSM 2008; X84005
			Bacteria	Firmicutes	Clostridia	Clostridiales	Veillonellaceae	Veillonella	Veillonella atypica; ATCC 17744; AF439641
			Bacteria	Firmicutes	Clostridia	Clostridiales	Veillonellaceae	Veillonella	uncultured Veillonella sp.; 59-7-6; AY423747
			Bacteria	Firmicutes	Clostridia	Clostridiales	Veillonellaceae	Veillonella	Veillonella montpellierensis (T); ADV 281.99; AF473836
			Bacteria	Firmicutes	Clostridia	Clostridiales	Veillonellaceae	Veillonella	Veillonella caviae (T); DSM 20738; AY355140
			Bacteria	Firmicutes	Clostridia	Clostridiales	Veillonellaceae	Veillonella	Veillonella parvula; CIP 60.1; AF439640
			Bacteria	Firmicutes	Clostridia	Clostridiales	Veillonellaceae	Veillonella	Veillonella ratti (T); DSM 20736; AY355138
			Bacteria	Firmicutes	Clostridia	Clostridiales	Veillonellaceae	Veillonella	Veillonella montpellierensis; ADV 2216.03; AY355141
			Bacteria	Firmicutes	Clostridia	Clostridiales	Veillonellaceae	Veillonella	Veillonella dispar; ATCC 17748; AF439639
			Bacteria	Firmicutes	Clostridia	Clostridiales	Veillonellaceae	Veillonella	Veillonella montpellierensis; ADV 3198.03; AY355142
			Bacteria	Firmicutes	Clostridia	Clostridiales	Veillonellaceae	Veillonella	Veillonella rodentium; ATCC 17743; AY514996
o1604	o1604 16S-VeiGroup2	AAAACAGGTGGTGCACGGTT	Bacteria	Firmicutes	Clostridia	Clostridiales	Veillonellaceae	Veillonella	Veillonella montpellierensis; 2001-112662; AY244769
			Bacteria	Firmicutes	Clostridia	Clostridiales	Veillonellaceae	Veillonella	Veillonella sp. oral clone X042; AF287781
			Bacteria	Firmicutes	Clostridia	Clostridiales	Veillonellaceae	Veillonella	uncultured Veillonella sp.; BU083; AF366266
			Bacteria	Firmicutes	Clostridia	Clostridiales	Veillonellaceae	Veillonella	Veillonella dispar (T); DSM 20735; X84006
			Bacteria	Firmicutes	Clostridia	Clostridiales	Veillonellaceae	Veillonella	Veillonella sp. oral clone AA050; AF287782
			Bacteria	Firmicutes	Clostridia	Clostridiales	Veillonellaceae	Veillonella	Veillonella atypica (T); DSM 20739; X84007

			Bacteria	Firmicutes	Clostridia	Clostridiales	Veillonellaceae	Veillonella	uncultured bacterium; p-5279-2Wa3; AF371939
			Bacteria	Firmicutes	Clostridia	Clostridiales	Veillonellaceae	Veillonella	Veillonella parvula (T); DSM 2008; X84005
			Bacteria	Firmicutes	Clostridia	Clostridiales	Veillonellaceae	Veillonella	uncultured Veillonella sp.; 59-7-18; AY394004
			Bacteria	Firmicutes	Clostridia	Clostridiales	Veillonellaceae	Veillonella	uncultured Veillonella sp.; 59-8-23; AY423748
			Bacteria	Firmicutes	Clostridia	Clostridiales	Veillonellaceae	Veillonella	Veillonella atypica; ATCC 17744; AF439641
			Bacteria	Firmicutes	Clostridia	Clostridiales	Veillonellaceae	Veillonella	uncultured Veillonella sp.; 59-7-6; AY423747
			Bacteria	Firmicutes	Clostridia	Clostridiales	Veillonellaceae	Veillonella	Veillonella montpellierensis (T); ADV 281.99; AF473836
			Bacteria	Firmicutes	Clostridia	Clostridiales	Veillonellaceae	Veillonella	Veillonella caviae (T); DSM 20738; AY355140
			Bacteria	Firmicutes	Clostridia	Clostridiales	Veillonellaceae	Veillonella	Veillonella parvula; CIP 60.1; AF439640
			Bacteria	Firmicutes	Clostridia	Clostridiales	Veillonellaceae	Veillonella	uncultured bacterium; B608; AY984660
			Bacteria	Firmicutes	Clostridia	Clostridiales	Veillonellaceae	Veillonella	Veillonella montpellierensis; ADV 2216.03; AY355141
			Bacteria	Firmicutes	Clostridia	Clostridiales	Veillonellaceae	Veillonella	Veillonella dispar; ATCC 17748; AF439639
			Bacteria	Firmicutes	Clostridia	Clostridiales	Veillonellaceae	Veillonella	Veillonella montpellierensis; ADV 3198.03; AY355142
			Bacteria	Firmicutes	Clostridia	Clostridiales	Veillonellaceae	Veillonella	Veillonella rodentium; ATCC 17743; AY514996
o1605	o1605 16S-VeiOral	AGATGAAAGGTGGCCTCTAC	Bacteria	Firmicutes	Clostridia	Clostridiales	Veillonellaceae	Veillonella	uncultured low G+C Gram-positive bacterium; FAC36; DQ451475
			Bacteria	Firmicutes	Clostridia	Clostridiales	Veillonellaceae	Veillonella	uncultured bacterium; BF0002A015; AM696830
			Bacteria	Firmicutes	Clostridia	Clostridiales	Veillonellaceae	Veillonella	uncultured bacterium; P4D7-651; EF509966
			Bacteria	Firmicutes	Clostridia	Clostridiales	Veillonellaceae	Veillonella	uncultured bacterium; P2D1-725; EF510342
			Bacteria	Firmicutes	Clostridia	Clostridiales	Veillonellaceae	Veillonella	uncultured bacterium; P2D1-676; EF510351
			Bacteria	Firmicutes	Clostridia	Clostridiales	Veillonellaceae	Veillonella	uncultured bacterium; P2D1-705; EF510365
			Bacteria	Firmicutes	Clostridia	Clostridiales	Veillonellaceae	Veillonella	uncultured bacterium; P2D1-482; EF510359
			Bacteria	Firmicutes	Clostridia	Clostridiales	Veillonellaceae	Veillonella	uncultured bacterium; P4D7-670; EF509900
			Bacteria	Firmicutes	Clostridia	Clostridiales	Veillonellaceae	Veillonella	uncultured bacterium; P2D1-558; EF510361
			Bacteria	Firmicutes	Clostridia	Clostridiales	Veillonellaceae	Veillonella	uncultured bacterium; P4D7-445; EF509822
			Bacteria	Firmicutes	Clostridia	Clostridiales	Veillonellaceae	Veillonella	Veillonella parvula; ATCC 17745; AY995769
			Bacteria	Firmicutes	Clostridia	Clostridiales	Veillonellaceae	Veillonella	Veillonella sp. oral clone X042; AF287781
			Bacteria	Firmicutes	Clostridia	Clostridiales	Veillonellaceae	Veillonella	Veillonella sp. oral clone AA050; AF287782
			Bacteria	Firmicutes	Clostridia	Clostridiales	Veillonellaceae	Veillonella	uncultured Firmicutes bacterium; M0018_110; EF071193
			Bacteria	Firmicutes	Clostridia	Clostridiales	Veillonellaceae	Veillonella	Veillonella sp. oral clone VeillH4; AY995756
			Bacteria	Firmicutes	Clostridia	Clostridiales	Veillonellaceae	Veillonella	Veillonella sp. oral clone VeillC3; AY995752
			Bacteria	Firmicutes	Clostridia	Clostridiales	Veillonellaceae	Veillonella	Veillonella sp. oral clone VeillB2; AY995751
			Bacteria	Firmicutes	Clostridia	Clostridiales	Veillonellaceae	Veillonella	uncultured bacterium; P2D1-684; EF510345
			Bacteria	Firmicutes	Clostridia	Clostridiales	Veillonellaceae	Veillonella	Veillonella sp. ADV 269.01; AY571668
			Bacteria	Firmicutes	Clostridia	Clostridiales	Veillonellaceae	Veillonella	uncultured bacterium; CD109; DQ441360
o1606	o1606 16S-VeiPar	AAAGGCCTTCTTGCGAACAG	Bacteria	Firmicutes	Clostridia	Clostridiales	Veillonellaceae	Veillonella	uncultured bacterium; SJTU_F_05_84; EF398625
			Bacteria	Firmicutes	Clostridia	Clostridiales	Veillonellaceae	Veillonella	uncultured bacterium; SJTU_F_02_90; EF398361
			Bacteria	Firmicutes	Clostridia	Clostridiales	Veillonellaceae	Veillonella	uncultured bacterium; SJTU_F_03_20; EF398512
			Bacteria	Firmicutes	Clostridia	Clostridiales	Veillonellaceae	Veillonella	uncultured bacterium; SJTU_F_04_87; EF398540
			Bacteria	Firmicutes	Clostridia	Clostridiales	Veillonellaceae	Veillonella	uncultured bacterium; SJTU_F_04_85; EF398542
			Bacteria	Firmicutes	Clostridia	Clostridiales	Veillonellaceae	Veillonella	uncultured bacterium; SJTU_F_05_53; EF398647
			Bacteria	Firmicutes	Clostridia	Clostridiales	Veillonellaceae	Veillonella	uncultured bacterium; SJTU_F_08_17; EF398855
			Bacteria	Firmicutes	Clostridia	Clostridiales	Veillonellaceae	Veillonella	uncultured bacterium; SJTU_F_10_64; EF399045
			Bacteria	Firmicutes	Clostridia	Clostridiales	Veillonellaceae	Veillonella	uncultured bacterium; SJTU_F_12_51; EF399165
			Bacteria	Firmicutes	Clostridia	Clostridiales	Veillonellaceae	Veillonella	uncultured bacterium; SJTU_F_12_53; EF399167
			Bacteria	Firmicutes	Clostridia	Clostridiales	Veillonellaceae	Veillonella	uncultured bacterium; SJTU_F_12_60; EF399174
			Bacteria	Firmicutes	Clostridia	Clostridiales	Veillonellaceae	Veillonella	uncultured bacterium; SJTU_F_12_86; EF399198
			Bacteria	Firmicutes	Clostridia	Clostridiales	Veillonellaceae	Veillonella	uncultured bacterium; SJTU_F_05_25; EF398673
			Bacteria	Firmicutes	Clostridia	Clostridiales	Veillonellaceae	Veillonella	uncultured bacterium; SJTU_F_04_72; EF398554
			Bacteria	Firmicutes	Clostridia	Clostridiales	Veillonellaceae	Veillonella	uncultured bacterium; JSC7-7; DQ532206
			Bacteria	Firmicutes	Clostridia	Clostridiales	Veillonellaceae	Veillonella	Veillonella parvula; ATCC 10790; AY995767
			Bacteria	Firmicutes	Clostridia	Clostridiales	Veillonellaceae	Veillonella	Veillonella sp. oral clone VeillE3; AY995760
			Bacteria	Firmicutes	Clostridia	Clostridiales	Veillonellaceae	Veillonella	Veillonella sp. oral clone VeillD5; AY995759
			Bacteria	Firmicutes	Clostridia	Clostridiales	Veillonellaceae	Veillonella	Veillonella parvula (T); DSM 2008; X84005
			Bacteria	Firmicutes	Clostridia	Clostridiales	Veillonellaceae	Veillonella	Veillonella parvula; CIP 60.1; AF439640
o1607	o1607 16S-VeSpe	CTCTTCTTCGGAAGCCAGAA	Bacteria	Firmicutes	Clostridia	Clostridiales	Veillonellaceae	Veillonella	Veillonella sp. oral clone X042; AF287781
			Bacteria	Firmicutes	Clostridia	Clostridiales	Veillonellaceae	Veillonella	uncultured Veillonella sp.; BU083; AF366266
			Bacteria	Firmicutes	Clostridia	Clostridiales	Veillonellaceae	Veillonella	Veillonella dispar (T); DSM 20735; X84006
			Bacteria	Firmicutes	Clostridia	Clostridiales	Veillonellaceae	Veillonella	Veillonella sp. oral clone AA050; AF287782
			Bacteria	Firmicutes	Clostridia	Clostridiales	Veillonellaceae	Veillonella	Veillonella atypica; ATCC 17744; AY995768
			Bacteria	Firmicutes	Clostridia	Clostridiales	Veillonellaceae	Veillonella	Veillonella atypica (T); DSM 20739; X84007
			Bacteria	Firmicutes	Clostridia	Clostridiales	Veillonellaceae	Veillonella	Veillonella parvula; ATCC 10790; AY995767
			Bacteria	Firmicutes	Clostridia	Clostridiales	Veillonellaceae	Veillonella	uncultured bacterium; p-5279-2Wa3; AF371939
			Bacteria	Firmicutes	Clostridia	Clostridiales	Veillonellaceae	Veillonella	Veillonella parvula (T); DSM 2008; X84005
			Bacteria	Firmicutes	Clostridia	Clostridiales	Veillonellaceae	Veillonella	uncultured Veillonella sp.; 59-7-18; AY394004
			Bacteria	Firmicutes	Clostridia	Clostridiales	Veillonellaceae	Veillonella	uncultured Veillonella sp.; 59-8-23; AY423748
			Bacteria	Firmicutes	Clostridia	Clostridiales	Veillonellaceae	Veillonella	Veillonella atypica; ATCC 17744; AF439641
			Bacteria	Firmicutes	Clostridia	Clostridiales	Veillonellaceae	Veillonella	uncultured Veillonella sp.; 59-7-6; AY423747
			Bacteria	Firmicutes	Clostridia	Clostridiales	Veillonellaceae	Veillonella	Veillonella caviae (T); DSM 20738; AY355140
			Bacteria	Firmicutes	Clostridia	Clostridiales	Veillonellaceae	Veillonella	Veillonella parvula; CIP 60.1; AF439640
			Bacteria	Firmicutes	Clostridia	Clostridiales	Veillonellaceae	Veillonella	uncultured bacterium; BA05; AY985003
			Bacteria	Firmicutes	Clostridia	Clostridiales	Veillonellaceae	Veillonella	uncultured bacterium; B991; AY984994
			Bacteria	Firmicutes	Clostridia	Clostridiales	Veillonellaceae	Veillonella	uncultured bacterium; B608; AY984660
			Bacteria	Firmicutes	Clostridia	Clostridiales	Veillonellaceae	Veillonella	Veillonella dispar; ATCC 17748; AF439639
			Bacteria	Firmicutes	Clostridia	Clostridiales	Veillonellaceae	Veillonella	Veillonella rodentium; ATCC 17743; AY514996
o1608	o1608 16S-XanSpe	AACTGGATGTTGGGCACACT	Bacteria	Proteobacteria	Gammaproteobacteria	Xanthomonadales	Xanthomonadaceae	Rhodanobacter	uncultured bacterium; KD2-87; AY218596
			Bacteria	Proteobacteria	Gammaproteobacteria	Xanthomonadales	Xanthomonadaceae	unclassified_Xanthomonadaceae	uncultured gamma proteobacterium; za04; AJ604540
			Bacteria	Proteobacteria	Gammaproteobacteria	Xanthomonadales	Xanthomonadaceae	Dokdonella	uncultured gamma proteobacterium; Blsiii14; AJ318183
			Bacteria	Proteobacteria	Gammaproteobacteria	Xanthomonadales	Xanthomonadaceae	Dokdonella	uncultured bacterium; KD5-36; AY218737
			Bacteria	Proteobacteria	Gammaproteobacteria	Xanthomonadales	Xanthomonadaceae	unclassified_Xanthomonadaceae	Xanthomonas sp. ML-168b; AF140004
			Bacteria	Proteobacteria	Gammaproteobacteria	Xanthomonadales	Xanthomonadaceae	Dokdonella	uncultured bacterium; KD6-106; AY218747
			Bacteria	Proteobacteria	Gammaproteobacteria	Xanthomonadales	Xanthomonadaceae	Dokdonella	uncultured bacterium; KD5-12; AY218729
			Bacteria	Proteobacteria	Gammaproteobacteria	Xanthomonadales	Xanthomonadaceae	Lysobacter	Xanthomonas sp. ML-122; AF139997
			Bacteria	Proteobacteria	unclassified_Proteobacteria				uncultured bacterium; DR938CH110701SACH52; DQ230968
			Bacteria	Proteobacteria	Gammaproteobacteria	Xanthomonadales	Xanthomonadaceae	Dokdonella	Dokdonella koreensis (T); DS-123; AY987368
			Bacteria	Proteobacteria	Gammaproteobacteria	Xanthomonadales	Xanthomonadaceae	Dokdonella	Dokdonella koreensis; DS-140; AY987369
			Bacteria	Proteobacteria	Gammaproteobacteria	Xanthomonadales	Xanthomonadaceae	Dokdonella	Dokdonella ginsengisoli; Gsoil 191; AB245362
			Bacteria	Proteobacteria	Gammaproteobacteria	Xanthomonadales	Xanthomonadaceae	Dokdonella	Dokdonella fugitiva (T); type strain: A3; AJ969432
			Bacteria	Proteobacteria	Gammaproteobacteria	Xanthomonadales	Xanthomonadaceae	Dokdonella	Dokdonella koreensis; NML 00-0180; EF589678

			Bacteria	Proteobacteria	Gammaproteobacteria	Xanthomonadales	Xanthomonadaceae	Dokdonella	Dokdonella koreensis; NML 01-0233; EF589679
			Bacteria	Proteobacteria	Gammaproteobacteria	Xanthomonadales	Xanthomonadaceae	Dokdonella	uncultured bacterium; FCPS392; EF516860
			Bacteria	Proteobacteria	Gammaproteobacteria	Xanthomonadales	Xanthomonadaceae	unclassified_Xanthomonadaceae	Lysobacter taiwanensis; SEK-1197; DQ323664
			Bacteria	Proteobacteria	Gammaproteobacteria	Xanthomonadales	Xanthomonadaceae	unclassified_Xanthomonadaceae	Lysobacter taiwanensis; DD-1133; DQ323663
			Bacteria	Proteobacteria	Gammaproteobacteria	Xanthomonadales	Xanthomonadaceae	Lysobacter	Lysobacter taiwanensis; CL-1358; DQ314555
			Bacteria	Proteobacteria	Gammaproteobacteria	Xanthomonadales	Xanthomonadaceae	Dokdonella	uncultured gamma proteobacterium; AKYG495; AY921770
o1730	o1730 16S-KinOra	AAGCTAGAGTGTGTCAGAGG	Bacteria	Proteobacteria	Betaproteobacteria	Neisseriales	Neisseriaceae	Kingella	Neisseria sp. oral clone JC012; AY349388
			Bacteria	Proteobacteria	Gammaproteobacteria	Xanthomonadales	Xanthomonadaceae	Rhodanobacter	uncultured bacterium; KRA30-20; AY081991
			Bacteria	Proteobacteria	Gammaproteobacteria	Xanthomonadales	Xanthomonadaceae	unclassified_Xanthomonadaceae	uncultured bacterium; KD5-24; AY218735
			Bacteria	Proteobacteria	Gammaproteobacteria	Xanthomonadales	Xanthomonadaceae	Rhodanobacter	uncultured bacterium; KD9-56; AY218670
			Bacteria	Proteobacteria	Gammaproteobacteria	Xanthomonadales	Xanthomonadaceae	Rhodanobacter	uncultured bacterium; KD3-139; AY218606
			Bacteria	Proteobacteria	Betaproteobacteria	unclassified_Betaproteobacteria			uncultured bacterium; zEL64; DQ415856
			Bacteria	Proteobacteria	Gammaproteobacteria	Xanthomonadales	Xanthomonadaceae	unclassified_Xanthomonadaceae	uncultured soil bacterium; F47_Pitesti; DQ378209
			Bacteria	Proteobacteria	Gammaproteobacteria	Xanthomonadales	Xanthomonadaceae	unclassified_Xanthomonadaceae	uncultured bacterium; SC-10; AB255087
			Bacteria	Proteobacteria	Gammaproteobacteria	Xanthomonadales	Xanthomonadaceae	Rhodanobacter	uncultured bacterium; KD8-16; AY218686
			Bacteria	Proteobacteria	Betaproteobacteria	Burkholderiales	Burkholderiaceae	Ralstonia	uncultured bacterium; 655890; DQ404678
			Bacteria	Proteobacteria	Gammaproteobacteria	Xanthomonadales	Xanthomonadaceae	Rhodanobacter	uncultured bacterium; KD9-36; AY218667
			Bacteria	Proteobacteria	Gammaproteobacteria	Xanthomonadales	Xanthomonadaceae	Dyella	Dyella ginsengisoli; Gsoil 3046; AB245367
			Bacteria	Proteobacteria	Betaproteobacteria	Burkholderiales	Oxalobacteraceae	unclassified_Oxalobacteraceae	syncytium endosymbiont of Diaphorina citri; AB038368
			Bacteria	Proteobacteria	Betaproteobacteria	Burkholderiales	Burkholderiaceae	Polynucleobacter	uncultured beta proteobacterium; C3; DQ340187
			Bacteria	Proteobacteria	Gammaproteobacteria	Xanthomonadales	Xanthomonadaceae	Dyella	Dyella ginsengisoli; LA-3; EF191353
			Bacteria	Proteobacteria	Betaproteobacteria	Neisseriales	Neisseriaceae	Kingella	Kingella oralis (T); L06164
			Bacteria	Proteobacteria	Gammaproteobacteria	Xanthomonadales	Xanthomonadaceae	unclassified_Xanthomonadaceae	Frateuria sp. WJ64; AY495957
			Bacteria	Proteobacteria	Gammaproteobacteria	Xanthomonadales	Xanthomonadaceae	Dokdonella	uncultured gamma proteobacterium; AKYG1642; AY921834
			Bacteria	Proteobacteria	Gammaproteobacteria	Xanthomonadales	Xanthomonadaceae	Dyella	arsenic resistant soil bacterium 7-03; AF308875
			Bacteria	Proteobacteria	Gammaproteobacteria	Xanthomonadales	Xanthomonadaceae	unclassified_Xanthomonadaceae	uncultured gamma proteobacterium; JG35+U2B-AG12; AM116729
o1731	o1731 16S-StrUbe	GTACCCTATTTAAAAGGGGC	Bacteria	Firmicutes	Bacilli	Lactobacillales	Streptococcaceae	Streptococcus	Streptococcus uberis 0140J; AM946015
			Bacteria	Firmicutes	Bacilli	Lactobacillales	Streptococcaceae	Streptococcus	Streptococcus uberis 0140J; AM946015
			Bacteria	Firmicutes	Bacilli	Lactobacillales	Streptococcaceae	Streptococcus	Streptococcus uberis 0140J; AM946015
			Bacteria	Firmicutes	Bacilli	Lactobacillales	Streptococcaceae	Streptococcus	Streptococcus uberis 0140J; AM946015
			Bacteria	Firmicutes	Bacilli	Lactobacillales	Streptococcaceae	Streptococcus	Streptococcus uberis 0140J; AM946015
			Bacteria	Firmicutes	Bacilli	Lactobacillales	Streptococcaceae	Streptococcus	Streptococcus uberis; JCM 5709; AB023573
			Bacteria	Firmicutes	Bacilli	Lactobacillales	Streptococcaceae	Streptococcus	Streptococcus uberis; HN1; AB023576
			Bacteria	Firmicutes	Bacilli	Lactobacillales	Streptococcaceae	Streptococcus	Streptococcus uberis; ATCC 27958; AB002527
			Bacteria	Firmicutes	Bacilli	Lactobacillales	Streptococcaceae	Streptococcus	Streptococcus uberis; 1-362B-1; AB370974
			Bacteria	Firmicutes	Bacilli	Lactobacillales	Streptococcaceae	Streptococcus	Streptococcus uberis; ATCC 27958; U41048
			Bacteria	Firmicutes	Bacilli	Lactobacillales	Streptococcaceae	Streptococcus	Streptococcus uberis (T); ATCC 19436, NCTC 3858; AB002526
			Bacteria	Firmicutes	Bacilli	Lactobacillales	Streptococcaceae	Streptococcus	Streptococcus uberis; SC 13; GU108497
o1732	o1732 16S-CarHom	ACATCCTAGGAACTTGGCAG	Bacteria	Proteobacteria	Gammaproteobacteria	Cardiobacteriales	Cardiobacteriaceae	Cardiobacterium	uncultured bacterium; A_D_01_38; FJ470420
			Bacteria	Proteobacteria	Gammaproteobacteria	Cardiobacteriales	Cardiobacteriaceae	Cardiobacterium	Cardiobacterium sp. A; AB 2167; AF144697
			Bacteria	Proteobacteria	Gammaproteobacteria	Cardiobacteriales	Cardiobacteriaceae	Cardiobacterium	Cardiobacterium hominis (T); M35014
			Bacteria	Proteobacteria	Gammaproteobacteria	unclassified_Gammaproteobacteria			Cellvibrio sp. J115; EU143369
			Bacteria	Proteobacteria	Gammaproteobacteria	Cardiobacteriales	Cardiobacteriaceae	Cardiobacterium	uncultured Cardiobacterium sp.; 24B754; FJ976411
			Bacteria	Proteobacteria	Gammaproteobacteria	Cardiobacteriales	Cardiobacteriaceae	Cardiobacterium	uncultured bacterium; nbw285d10c1; GQ086085
			Bacteria	Proteobacteria	Gammaproteobacteria	Cardiobacteriales	Cardiobacteriaceae	Cardiobacterium	uncultured Cardiobacterium sp.; 17BA54; FJ976363
			Bacteria	Proteobacteria	Gammaproteobacteria	Cardiobacteriales	Cardiobacteriaceae	Cardiobacterium	uncultured bacterium; nbu415b03c1; GQ039216
o1733	o1733 16S-CamHom1	AGAGGCAGATGGAACTCTTG	Bacteria	Proteobacteria	Epsilonproteobacteria	Campylobacterales	Campylobacteraceae	Campylobacter	Campylobacter hominis ATCC BAA-381; CP000776
			Bacteria	Proteobacteria	Epsilonproteobacteria	Campylobacterales	Campylobacteraceae	Campylobacter	Campylobacter hominis ATCC BAA-381; CP000776
			Bacteria	Proteobacteria	Epsilonproteobacteria	Campylobacterales	Campylobacteraceae	Campylobacter	Campylobacter hominis ATCC BAA-381; CP000776
			Bacteria	Proteobacteria	Epsilonproteobacteria	Campylobacterales	Campylobacteraceae	Campylobacter	Campylobacter hominis; HS-B; AF062491
			Bacteria	Proteobacteria	Epsilonproteobacteria	Campylobacterales	Campylobacteraceae	Campylobacter	Campylobacter hominis (T); NCTC 13146; CH001A; AJ251584
			Bacteria	Proteobacteria	Epsilonproteobacteria	Campylobacterales	Campylobacteraceae	Campylobacter	Campylobacter hominis; UNSWCD; GQ167659
			Bacteria	Proteobacteria	Epsilonproteobacteria	Campylobacterales	Campylobacteraceae	Campylobacter	uncultured bacterium; nbu272e10c1; GQ021119
			Bacteria	Proteobacteria	Epsilonproteobacteria	Campylobacterales	Campylobacteraceae	Campylobacter	uncultured bacterium; nbw1009a09c1; GQ047130
			Bacteria	Proteobacteria	Epsilonproteobacteria	Campylobacterales	Campylobacteraceae	Campylobacter	uncultured bacterium; nbw760b07c1; GQ014202
			Bacteria	Proteobacteria	Epsilonproteobacteria	Campylobacterales	Campylobacteraceae	Campylobacter	uncultured bacterium; nbw761f06c1; GQ014589
			Bacteria	Proteobacteria	Epsilonproteobacteria	Campylobacterales	Campylobacteraceae	Campylobacter	uncultured bacterium; nbu524c09c1; GQ038878
			Bacteria	Proteobacteria	Epsilonproteobacteria	Campylobacterales	Campylobacteraceae	Campylobacter	Campylobacter hominis; NCTC 13146; DQ174182
			Bacteria	Proteobacteria	Epsilonproteobacteria	Campylobacterales	Campylobacteraceae	Campylobacter	uncultured bacterium; nbw761e12c1; GQ014585
			Bacteria	Proteobacteria	Epsilonproteobacteria	Campylobacterales	Campylobacteraceae	Campylobacter	uncultured bacterium; hoa5_05e09; EU344611
			Bacteria	Proteobacteria	Epsilonproteobacteria	Campylobacterales	Campylobacteraceae	Campylobacter	Campylobacter hominis; 2; EU781615
			Bacteria	Proteobacteria	Epsilonproteobacteria	Campylobacterales	Campylobacteraceae	Campylobacter	Campylobacter helveticus; NCTC 12472; DQ174163
o1734	o1734 16S-CamRec1	TGAGAGGCCGCATGCCATA	Bacteria	Proteobacteria	Gammaproteobacteria	Pasteurellales	Pasteurellaceae	Haemophilus	Haemophilus influenzae; M8551; AY613452
			Bacteria	Proteobacteria	Gammaproteobacteria	Pasteurellales	Pasteurellaceae	Haemophilus	Haemophilus influenzae; M11102; AY613445
			Bacteria	Proteobacteria	Gammaproteobacteria	Pasteurellales	Pasteurellaceae	Haemophilus	Haemophilus influenzae; M10079; AY613458
			Bacteria	Proteobacteria	Gammaproteobacteria	Pasteurellales	Pasteurellaceae	Haemophilus	Haemophilus influenzae; M10938; AY613459
			Bacteria	Proteobacteria	Gammaproteobacteria	Pasteurellales	Pasteurellaceae	Haemophilus	Haemophilus influenzae; M10259; AY613460
			Bacteria	Proteobacteria	Gammaproteobacteria	Pasteurellales	Pasteurellaceae	Haemophilus	Haemophilus influenzae; M9674; AY613461
			Bacteria	Proteobacteria	Gammaproteobacteria	Pasteurellales	Pasteurellaceae	Haemophilus	Haemophilus influenzae; M8263; AY613462
			Bacteria	Proteobacteria	Gammaproteobacteria	Pasteurellales	Pasteurellaceae	Haemophilus	Haemophilus influenzae; M7986; AY613463
			Bacteria	Proteobacteria	Gammaproteobacteria	Pasteurellales	Pasteurellaceae	Haemophilus	Haemophilus influenzae; M7983; AY613464
			Bacteria	Proteobacteria	Gammaproteobacteria	Pasteurellales	Pasteurellaceae	Haemophilus	Haemophilus influenzae; M10473; AY613447
			Bacteria	Proteobacteria	Gammaproteobacteria	Pasteurellales	Pasteurellaceae	Haemophilus	Haemophilus influenzae; M10984; AY613448
			Bacteria	Proteobacteria	Gammaproteobacteria	Pasteurellales	Pasteurellaceae	Haemophilus	Haemophilus influenzae; M10313; AY613449
			Bacteria	Proteobacteria	Gammaproteobacteria	Pasteurellales	Pasteurellaceae	Haemophilus	Haemophilus influenzae; M8902; AY613450
			Bacteria	Proteobacteria	Gammaproteobacteria	Pasteurellales	Pasteurellaceae	Haemophilus	Haemophilus influenzae; M7697; AY613453
			Bacteria	Proteobacteria	Gammaproteobacteria	Pasteurellales	Pasteurellaceae	Haemophilus	Haemophilus influenzae; M11131; AY613465
			Bacteria	Proteobacteria	Gammaproteobacteria	Pasteurellales	Pasteurellaceae	Haemophilus	Haemophilus influenzae; M6859; AY613456
			Bacteria	Proteobacteria	Gammaproteobacteria	Pasteurellales	Pasteurellaceae	Haemophilus	Haemophilus influenzae (T); M35019
			Bacteria	Proteobacteria	Gammaproteobacteria	Pasteurellales	Pasteurellaceae	Haemophilus	Haemophilus influenzae; CIP 5483; X87977
			Bacteria	Proteobacteria	Gammaproteobacteria	Pasteurellales	Pasteurellaceae	Haemophilus	Haemophilus aegyptius (T); M75044
			Bacteria	Proteobacteria	Gammaproteobacteria	Pasteurellales	Pasteurellaceae	Haemophilus	Haemophilus aegyptius; CCUG 25716; AY362905
o1735	o1735 16S-CamSpu	AAAGGACGCGTAGGTGGATA	Bacteria	Proteobacteria	Epsilonproteobacteria	Campylobacterales	Campylobacteraceae	Campylobacter	Campylobacter sputorum subsp. sputorum (T); biovar sputorum;LMG 7795; X67775
			Bacteria	Proteobacteria	Epsilonproteobacteria	Campylobacterales	Campylobacteraceae	Campylobacter	Campylobacter sputorum subsp. sputorum; LMG 10388; AF550635
			Bacteria	Proteobacteria	Epsilonproteobacteria	Campylobacterales	Campylobacteraceae	Campylobacter	Campylobacter sputorum subsp. sputorum; CSP-2; AF550639
			Bacteria	Proteobacteria	Epsilonproteobacteria	Campylobacterales	Campylobacteraceae	Campylobacter	Campylobacter sputorum subsp. sputorum; LMG 11761; AF550636
			Bacteria	Proteobacteria	Epsilonproteobacteria	Campylobacterales	Campylobacteraceae	Campylobacter	Campylobacter sputorum subsp. sputorum; LMG 6617; AF550637
			Bacteria	Proteobacteria	Epsilonproteobacteria	Campylobacterales	Campylobacteraceae	Campylobacter	Campylobacter sputorum subsp. sputorum; CSP-1; AF550638

			Bacteria	Proteobacteria	Epsilonproteobacteria	Campylobacterales	Campylobacteraceae	Campylobacter	Campylobacter sputorum subsp. sputorum; CCUG 886; DQ174152
			Bacteria	Proteobacteria	Epsilonproteobacteria	Campylobacterales	Campylobacteraceae	Campylobacter	Campylobacter sputorum subsp. sputorum; NCTC 11367; DQ174151
			Bacteria	Proteobacteria	Epsilonproteobacteria	Campylobacterales	Campylobacteraceae	Campylobacter	Campylobacter sputorum subsp. sputorum; CCUG 24261; DQ174153
			Bacteria	Proteobacteria	Epsilonproteobacteria	Campylobacterales	Campylobacteraceae	Campylobacter	Campylobacter sputorum subsp. sputorum; LMG 7795; DQ174149
			Bacteria	Proteobacteria	Epsilonproteobacteria	Campylobacterales	Campylobacteraceae	Campylobacter	Campylobacter sputorum subsp. sputorum; NZ504-95; DQ174154
			Bacteria	Proteobacteria	Epsilonproteobacteria	Campylobacterales	Campylobacteraceae	Campylobacter	Campylobacter sputorum subsp. sputorum; NCTC 11415; DQ174150
			Bacteria	Proteobacteria	Epsilonproteobacteria	Campylobacterales	Campylobacteraceae	Campylobacter	Campylobacter sputorum subsp. sputorum; BU-112B; AF022768
			Bacteria	Proteobacteria	Epsilonproteobacteria	Campylobacterales	Campylobacteraceae	Campylobacter	uncultured bacterium; nbu374h02c1; GQ035643
o1736	o1736 16S-MarHyd	AGGCTTGACGTTACCTACAG	Bacteria	Proteobacteria	Gammaproteobacteria	Alteromonadales	Incertae sedis 7	Marinobacter	Marinobacter sp. CAB; U61848
			Bacteria	Proteobacteria	Gammaproteobacteria	Alteromonadales	Incertae sedis 7	Marinobacter	Marinobacter hydrocarbonoclasticus (T); VT8; AJ000726
			Bacteria	Proteobacteria	Gammaproteobacteria	Alteromonadales	Incertae sedis 7	Marinobacter	Marinobacter sp. HB7; AB089804
			Bacteria	Proteobacteria	Gammaproteobacteria	Alteromonadales	Incertae sedis 7	Marinobacter	Marinobacter hydrocarbonoclasticus; ATCC 27132T; AB021372
			Bacteria	Proteobacteria	Gammaproteobacteria	Alteromonadales	Incertae sedis 7	Marinobacter	Marinobacter hydrocarbonoclasticus; Y16735
			Bacteria	Proteobacteria	Gammaproteobacteria	Alteromonadales	Incertae sedis 7	Marinobacter	Marinobacter hydrocarbonoclasticus (T); MBIC1303 (=ATCC27132); AB019148
			Bacteria	Proteobacteria	Gammaproteobacteria	Alteromonadales	Incertae sedis 7	Marinobacter	Marinobacter hydrocarbonoclasticus; OC-11; AY669171
			Bacteria	Proteobacteria	Gammaproteobacteria	Alteromonadales	Incertae sedis 7	Marinobacter	Marinobacter hydrocarbonoclasticus; OC-9; AY669169
			Bacteria	Proteobacteria	Gammaproteobacteria	Alteromonadales	Incertae sedis 7	Marinobacter	Marinobacter sp. NT N68; AB166999
			Bacteria	Proteobacteria	Gammaproteobacteria	Alteromonadales	Incertae sedis 7	Marinobacter	Marinobacter sp. CBF L52; AB166952
			Bacteria	Proteobacteria	Gammaproteobacteria	Alteromonadales	Incertae sedis 7	Marinobacter	Marinobacter sp. NT N107; AB167025
			Bacteria	Proteobacteria	Gammaproteobacteria	Alteromonadales	Incertae sedis 7	Marinobacter	Marinobacter sp. NT N127; AB167041
			Bacteria	Proteobacteria	Gammaproteobacteria	Alteromonadales	Incertae sedis 7	Marinobacter	Marinobacter sp. Libra; AY734434
			Bacteria	Proteobacteria	Gammaproteobacteria	Alteromonadales	Incertae sedis 7	Marinobacter	Marinobacter sp. NT N80; AB167006
			Bacteria	Proteobacteria	Gammaproteobacteria	Alteromonadales	Incertae sedis 7	Marinobacter	Marinobacter hydrocarbonoclasticus; OC-1; AY669163
			Bacteria	Proteobacteria	Gammaproteobacteria	Oceanospirillales	Alcanivoraceae	Alcanivorax	Alcanivorax sp. EPR 7; AY394866
			Bacteria	Proteobacteria	Gammaproteobacteria	Alteromonadales	Incertae sedis 7	Marinobacter	Marinobacter sp. NT N78; AB167004
			Bacteria	Proteobacteria	Gammaproteobacteria	Alteromonadales	Incertae sedis 7	Marinobacter	bacterium K54; AY345466
			Bacteria	Proteobacteria	Gammaproteobacteria	Alteromonadales	Incertae sedis 7	Marinobacter	Marinobacter hydrocarbonoclasticus; ATCC 49840; X67022
			Bacteria	Proteobacteria	Gammaproteobacteria	Alteromonadales	Incertae sedis 7	Marinobacter	Marinobacter hydrocarbonoclasticus; OC-8; AY669168
			Bacteria	Proteobacteria	Epsilonproteobacteria	Campylobacterales	Campylobacteraceae	Arcobacter	uncultured bacterium; A28; EU234123
o1738	o1738 16S-VibNer	ACTGGCACACTAGAGTACTG	Bacteria	Proteobacteria	Gammaproteobacteria	Vibrionales	Vibrionaceae	Vibrio	Vibrio sp. S-9; DQ978990
			Bacteria	Proteobacteria	Gammaproteobacteria	Vibrionales	Vibrionaceae	Vibrio	Vibrio sp. S-4; DQ978985
			Bacteria	Proteobacteria	Gammaproteobacteria	Vibrionales	Vibrionaceae	Vibrio	Vibrio sp. S-11; DQ978992
			Bacteria	Proteobacteria	Gammaproteobacteria	Vibrionales	Vibrionaceae	Vibrio	uncultured bacterium; O2-37; GQ866111
			Bacteria	Proteobacteria	Gammaproteobacteria	Vibrionales	Vibrionaceae	Vibrio	Vibrio sp. S-12; DQ978993
			Bacteria	Proteobacteria	Gammaproteobacteria	Vibrionales	Vibrionaceae	Vibrio	Vibrio fortis; LMG 21559; AJ514914
			Bacteria	Proteobacteria	Gammaproteobacteria	Vibrionales	Vibrionaceae	Vibrio	Vibrio sp. 2-2; FJ025761
			Bacteria	Proteobacteria	Gammaproteobacteria	Vibrionales	Vibrionaceae	Vibrio	Vibrio sp. MB53; AB518969
			Bacteria	Proteobacteria	Gammaproteobacteria	Vibrionales	Vibrionaceae	Vibrio	Vibrio nereis (T); ATCC 25917T; X74716
			Bacteria	Proteobacteria	Gammaproteobacteria	Vibrionales	Vibrionaceae	Vibrio	Vibrio sp. 14-1; FJ025771
o1739	o1739 16S-VibAlg	TTGGGAACGATAACGGCGTT	Bacteria	Proteobacteria	Gammaproteobacteria	Vibrionales	Vibrionaceae	Vibrio	Vibrio alginolyticus; ATCC 17749; X56576
o1741	o1741 16S-VeiRat	AACTGGAGTATCGGAGAGGA	Bacteria	Firmicutes	Clostridia	Clostridiales	Veillonellaceae	Dialister	uncultured bacterium; TuCw9; DQ071438
			Bacteria	Firmicutes	Clostridia	Clostridiales	Veillonellaceae	Dialister	uncultured bacterium; TuCw54; DQ071538
			Bacteria	Firmicutes	Clostridia	Clostridiales	Veillonellaceae	Dialister	uncultured bacterium; TuCw50; DQ071474
			Bacteria	Firmicutes	Clostridia	Clostridiales	Veillonellaceae	Dialister	uncultured bacterium; TuCc32; DQ071524
			Bacteria	Firmicutes	Clostridia	Clostridiales	Veillonellaceae	Dialister	uncultured bacterium; TuCw74; DQ071535
			Bacteria	Firmicutes	Clostridia	Clostridiales	Veillonellaceae	Dialister	uncultured bacterium; Z83; DQ353930
			Bacteria	Firmicutes	Clostridia	Clostridiales	Veillonellaceae	Dialister	uncultured bacterium; Z89; DQ353933
			Bacteria	Firmicutes	Clostridia	Clostridiales	Veillonellaceae	Dialister	uncultured bacterium; WTB_M8; EU009830
			Bacteria	Firmicutes	Clostridia	Clostridiales	Veillonellaceae	Dialister	uncultured bacterium; WTB_I47; EU009817
			Bacteria	Firmicutes	Clostridia	Clostridiales	Veillonellaceae	Dialister	uncultured bacterium; WTB_X48; EU009856
			Bacteria	Firmicutes	Clostridia	Clostridiales	Veillonellaceae	Veillonella	Veillonella ratti; ATCC17746; AF186071
			Bacteria	Firmicutes	Clostridia	Clostridiales	Veillonellaceae	Veillonella	uncultured bacterium; TS55_a01h02; FJ369881
			Bacteria	Firmicutes	Clostridia	Clostridiales	Veillonellaceae	Veillonella	uncultured bacterium; TS55_a02g07; FJ369962
			Bacteria	Firmicutes	Clostridia	Clostridiales	Veillonellaceae	Veillonella	uncultured bacterium; TS55_a01g05; FJ369872
			Bacteria	Firmicutes	Clostridia	Clostridiales	Veillonellaceae	Veillonella	Veillonella ratti (T); DSM 20736; AY355138
			Bacteria	Firmicutes	Clostridia	Clostridiales	Veillonellaceae	Veillonella	uncultured bacterium; TS55_a01c04; FJ369825
			Bacteria	Firmicutes	Clostridia	Clostridiales	Veillonellaceae	Veillonella	uncultured bacterium; TS55_a01a03; FJ369800
			Bacteria	Firmicutes	Clostridia	Clostridiales	Veillonellaceae	Veillonella	uncultured bacterium; TS55_a01e08; FJ369852
			Bacteria	Lentisphaerae	Lentisphaerae	Victivallales	Victivallaceae	Victivallis	uncultured bacterium; AFYEL_aaj68b01; EU465101
			Bacteria	Firmicutes	Clostridia	Clostridiales	Veillonellaceae	Veillonella	Veillonella ratti; ADV4313.2; AY211542
o1742	o1742 16S-VeiCri	TCGGCATCGGAGTTCATTGA	Bacteria	Firmicutes	Clostridia	Clostridiales	Veillonellaceae	Veillonella	Veillonella criceti (T); ATCC17747; AF186072
o1743	o1743 16S-VeiRatCri	AAGCTCTCAGTTCGGATCGT	Bacteria	Firmicutes	Clostridia	Clostridiales	Veillonellaceae	Propionispora	Propionispora hippei; type strain: KS; 1; AJ508928
			Bacteria	Firmicutes	Clostridia	Clostridiales	Veillonellaceae	Veillonella	uncultured Veillonella sp.; mother V4; 11V4; AM157449
			Bacteria	Firmicutes	Clostridia	Clostridiales	Veillonellaceae	Sporomusa	Sporomusa aerivorans; TMAM3; AJ506192
			Bacteria	Firmicutes	Clostridia	Clostridiales	Veillonellaceae	Sporomusa	Sporomusa aerivorans (T); type strain: TMAO3; AJ506191
			Bacteria	Firmicutes	Clostridia	Clostridiales	Veillonellaceae	Propionispora	Propionispora hippei (T); type strain: KS; 5; AJ508927
			Bacteria	Firmicutes	Clostridia	Clostridiales	Veillonellaceae	Veillonella	Veillonella sp. oral clone HB016; DQ087189
			Bacteria	Firmicutes	Clostridia	Clostridiales	Veillonellaceae	Veillonella	Veillonella sp. oral clone ASCB03; AY923122
			Bacteria	Firmicutes	Clostridia	Clostridiales	Veillonellaceae	Propionispora	Propionispora vibrioides (T); FKBS1; AJ279802
			Bacteria	Firmicutes	Clostridia	Clostridiales	Veillonellaceae	Sporomusa	Sporomusa acidovorans (T); DSM 3132 Type; AJ279798
			Bacteria	Firmicutes	Clostridia	Clostridiales	Veillonellaceae	Dendrosporobacter	Dendrosporobacter quercicolus; DSM 1736(T); AJ010962
			Bacteria	Firmicutes	Clostridia	Clostridiales	Veillonellaceae	Veillonella	Veillonella ratti; ATCC17746; AF186071
			Bacteria	Firmicutes	Clostridia	Clostridiales	Veillonellaceae	Veillonella	Veillonella criceti (T); ATCC17747; AF186072
			Bacteria	Firmicutes	Bacilli	Bacillales	Bacillaceae	unclassified_Bacillaceae	uncultured compost bacterium 4-75; AB034721
			Bacteria	Firmicutes	Clostridia	Clostridiales	Veillonellaceae	Dendrosporobacter	Dendrosporobacter quercicolus (T); M59110
			Bacteria	Firmicutes	Clostridia	Clostridiales	Veillonellaceae	Anaerosinus	Selenomonadaceae SB90; AJ229242
			Bacteria	Firmicutes	Clostridia	Clostridiales	Veillonellaceae	Anaerosinus	Pelosinus fermentans; R7; DQ145536
			Bacteria	Firmicutes	Clostridia	Clostridiales	Veillonellaceae	Veillonella	Veillonella ratti (T); DSM 20736; AY355138
			Bacteria	Firmicutes	Clostridia	Clostridiales	Veillonellaceae	Sporomusa	Sporomusa rhizae (T); type strain: RS; AM158322
			Bacteria	Firmicutes	Clostridia	Clostridiales	Veillonellaceae	Veillonella	Veillonella ratti; ADV4313.2; AY211542
			Bacteria	Firmicutes	Bacilli	Lactobacillales	unclassified_"Lactobacillales"		uncultured Enterococcus sp.; Rs-L13; AB100496
o1744	o1744 16S-CapHea	AAATGGGAAGCGACGTAGGT	Bacteria	Bacteroidetes	Flavobacteria	Flavobacteriales	Flavobacteriaceae	Capnocytophaga	Capnocytophaga haemolytica; A0404; DQ009624
			Bacteria	Bacteroidetes	Flavobacteria	Flavobacteriales	Flavobacteriaceae	Capnocytophaga	Capnocytophaga haemolytica; LMG 16021 T; JCM 8565 T; U41349
o1745	o1745 16S-StrSin	ACCTTTTAGCGGGGGATAAC	Bacteria	Firmicutes	Bacilli	Lactobacillales	Streptococcaceae	Streptococcus	Streptococcus castoreus (T); type strain: M605815/03/2; AJ606047
			Bacteria	Firmicutes	Bacilli	Lactobacillales	Streptococcaceae	Streptococcus	uncultured Streptococcus sp.; 202D12(oral); AM420044
			Bacteria	Firmicutes	Bacilli	Lactobacillales	Streptococcaceae	Streptococcus	Streptococcus sinensis (T); HKU4; AF432856
			Bacteria	Firmicutes	Bacilli	Lactobacillales	Streptococcaceae	Streptococcus	Streptococcus sinensis; HKU5; AF432855
			Bacteria	Firmicutes	Bacilli	Lactobacillales	Streptococcaceae	Streptococcus	Streptococcus sinensis; HKU6; AF432857

			Bacteria	Firmicutes	Bacilli	Lactobacillales	Streptococcaceae	Streptococcus	uncultured Streptococcus sp.; EHFS1_S16f; EU071536
			Bacteria	Firmicutes	Bacilli	Lactobacillales	Streptococcaceae	Streptococcus	Streptococcus oligofermentans (T); 2-4T; AY099095
			Bacteria	Firmicutes	Bacilli	Lactobacillales	Streptococcaceae	Streptococcus	Streptococcus devriesei; 26901-02; EU483245
			Bacteria	Firmicutes	Bacilli	Lactobacillales	unclassified_"Lactobacillales"		uncultured bacterium; PeM67; AJ576421
			Bacteria	Firmicutes	Bacilli	Lactobacillales	Enterococcaceae	unclassified_"Enterococcaceae"	uncultured bacterium; PeH28; AJ576345
			Bacteria	Firmicutes	Bacilli	Lactobacillales	unclassified_"Lactobacillales"		uncultured bacterium; PeH30; AJ576347
			Bacteria	Firmicutes	Bacilli	Lactobacillales	Streptococcaceae	Streptococcus	Streptococcus sinensis; HDP 2005-0155; EF371928
			Bacteria	Firmicutes	Bacilli	Lactobacillales	Streptococcaceae	Streptococcus	Streptococcus sp. VA1051_00; AY049738
			Bacteria	Firmicutes	Bacilli	Lactobacillales	unclassified_"Lactobacillales"		uncultured bacterium; PeM54; AJ576400
			Bacteria	Firmicutes	Bacilli	Lactobacillales	unclassified_"Lactobacillales"		uncultured bacterium; PeM58; AJ576415
			Bacteria	Firmicutes	Bacilli	Lactobacillales	Streptococcaceae	Streptococcus	Streptococcus devriesei (T); CCUG 47156; AJ564067
			Bacteria	Firmicutes	Bacilli	Lactobacillales	Streptococcaceae	Streptococcus	Streptococcus devriesei; 52173-03; EU483246
			Bacteria	Proteobacteria	Gammaproteobacteria	Legionellales	Coxiellaceae	Aquicella	uncultured gamma proteobacterium; Sliv-7; FM877660
			Bacteria	Proteobacteria	Gammaproteobacteria	Legionellales	Coxiellaceae	Aquicella	uncultured gamma proteobacterium; Sliv-27; FM877663
			Bacteria	Proteobacteria	Gammaproteobacteria	Legionellales	Coxiellaceae	Aquicella	uncultured gamma proteobacterium; Sliv-129; FM877667
o1746	o1746 16S-StrCan	ACAGGTGCTTGCACTAGTCT	Bacteria	Firmicutes	Bacilli	Lactobacillales	Streptococcaceae	Streptococcus	Streptococcus canis (T); ATCC 43498, DSM 20715; AB002483
			Bacteria	Firmicutes	Bacilli	Lactobacillales	Streptococcaceae	Streptococcus	Streptococcus sp. 'group G'; ATCC 27961; AB002517
			Bacteria	Firmicutes	Bacilli	Lactobacillales	Streptococcaceae	Streptococcus	Streptococcus canis; 1-82MP; EU075056
			Bacteria	Firmicutes	Bacilli	Lactobacillales	Streptococcaceae	Streptococcus	Streptococcus canis; 11-ninetysevenmp; EU075066
			Bacteria	Firmicutes	Bacilli	Lactobacillales	Streptococcaceae	Streptococcus	Streptococcus canis; CG40; AJ413205
			Bacteria	Firmicutes	Bacilli	Lactobacillales	Streptococcaceae	Streptococcus	Streptococcus canis; 3-35MP; EU075058
			Bacteria	Firmicutes	Bacilli	Lactobacillales	Streptococcaceae	Streptococcus	Streptococcus canis; NCTC12191; AJ413203
			Bacteria	Firmicutes	Bacilli	Lactobacillales	Streptococcaceae	Streptococcus	Streptococcus canis; 20-71MP; EU075074
			Bacteria	Firmicutes	Bacilli	Lactobacillales	Streptococcaceae	Streptococcus	Streptococcus canis; 5-69MP; EU075060
			Bacteria	Firmicutes	Bacilli	Lactobacillales	Streptococcaceae	Streptococcus	Streptococcus canis; CG5; AJ413204
			Bacteria	Firmicutes	Bacilli	Lactobacillales	Streptococcaceae	Streptococcus	Streptococcus canis; ATCC 43496; DQ303184
			Bacteria	Firmicutes	Bacilli	Lactobacillales	Streptococcaceae	Streptococcus	Streptococcus canis; 8-87MP; EU075063
			Bacteria	Firmicutes	Bacilli	Lactobacillales	Streptococcaceae	Streptococcus	Streptococcus canis; 12-97MPnew; EU075067
			Bacteria	Firmicutes	Bacilli	Lactobacillales	Streptococcaceae	Streptococcus	Streptococcus canis; 6-30MP; EU075061
			Bacteria	Firmicutes	Bacilli	Lactobacillales	Streptococcaceae	Streptococcus	Streptococcus canis; 16-14MPbeta; EU075071
			Bacteria	Firmicutes	Bacilli	Lactobacillales	Streptococcaceae	Streptococcus	Streptococcus canis; 19-90MP; EU075073
			Bacteria	Firmicutes	Bacilli	Lactobacillales	Streptococcaceae	Streptococcus	Streptococcus canis; 23-87MPnew; EU075076
			Bacteria	Firmicutes	Bacilli	Lactobacillales	Streptococcaceae	Streptococcus	Streptococcus dysgalactiae subsp. equisimilis; 34MPbeta; EU075034
			Bacteria	Firmicutes	Bacilli	Lactobacillales	Streptococcaceae	Streptococcus	Streptococcus canis; 88MP; EU075054
			Bacteria	Firmicutes	Bacilli	Lactobacillales	Streptococcaceae	Streptococcus	Streptococcus canis; 33MP; EU075042
o1747	o1747 16S-PreOul	AAACGATGACGAGAGCTTGC	Bacteria	Bacteroidetes	Bacteroidetes	Bacteroidales	Prevotellaceae	Prevotella	Prevotella oulorum (T); ATCC43324; L16472
			Bacteria	Bacteroidetes	Bacteroidetes	Bacteroidales	Prevotellaceae	Prevotella	Prevotella oulorum; C08KA; GQ422734
o1748	o1748 16S-LauSp	CTCCGCATGTAGCAGTGAAA	Bacteria	Proteobacteria	Betaproteobacteria	Burkholderiales	unclassified_Burkholderiales		uncultured Lautropia sp.; 2.6; DQ016727
			Bacteria	Proteobacteria	Betaproteobacteria	Burkholderiales	unclassified_Burkholderiales		uncultured bacterium; BF0001D034; AM697419
			Bacteria	Proteobacteria	Betaproteobacteria	Burkholderiales	Alcaligenaceae	Derxia	Lautropia sp. oral clone AP009; AY005030
			Bacteria	Proteobacteria	Betaproteobacteria	Burkholderiales	Alcaligenaceae	Derxia	uncultured Lautropia sp.; 24B473; FJ976403
o1749	o1749 16S-HaeInf	GGTATTGAGGAAGGGATGTG	Bacteria	Proteobacteria	Gammaproteobacteria	Pasteurellales	Pasteurellaceae	Haemophilus	Haemophilus quentini; MCCM 02026; AF224307
			Bacteria	Proteobacteria	Gammaproteobacteria	Pasteurellales	Pasteurellaceae	Haemophilus	uncultured bacterium; P1D1-509; EF511991
			Bacteria	Proteobacteria	Gammaproteobacteria	Pasteurellales	Pasteurellaceae	Haemophilus	uncultured bacterium; P1D1-566; EF511892
			Bacteria	Proteobacteria	Gammaproteobacteria	Pasteurellales	Pasteurellaceae	Haemophilus	uncultured bacterium; P1D1-694; EF511970
			Bacteria	Proteobacteria	Gammaproteobacteria	Pasteurellales	Pasteurellaceae	Haemophilus	uncultured bacterium; P1D1-749; EF511876
			Bacteria	Proteobacteria	Gammaproteobacteria	Pasteurellales	Pasteurellaceae	Haemophilus	uncultured bacterium; P1D1-755; EF511864
			Bacteria	Proteobacteria	Gammaproteobacteria	Pasteurellales	Pasteurellaceae	Haemophilus	uncultured bacterium; P1D1-518; EF511867
			Bacteria	Proteobacteria	Gammaproteobacteria	Pasteurellales	Pasteurellaceae	Haemophilus	uncultured bacterium; P1D1-688; EF511947
			Bacteria	Proteobacteria	Gammaproteobacteria	Pasteurellales	Pasteurellaceae	Haemophilus	uncultured bacterium; P1D1-535; EF511926
			Bacteria	Proteobacteria	Gammaproteobacteria	Pasteurellales	Pasteurellaceae	Haemophilus	uncultured bacterium; P1D1-707; EF511934
			Bacteria	Proteobacteria	Gammaproteobacteria	Pasteurellales	Pasteurellaceae	Haemophilus	uncultured bacterium; P1D1-700; EF511887
			Bacteria	Proteobacteria	Gammaproteobacteria	Pasteurellales	Pasteurellaceae	Haemophilus	uncultured bacterium; P1D1-498; EF511952
			Bacteria	Proteobacteria	Gammaproteobacteria	Pasteurellales	Pasteurellaceae	Haemophilus	uncultured bacterium; P1D1-676; EF511955
			Bacteria	Proteobacteria	Gammaproteobacteria	Pasteurellales	Pasteurellaceae	Haemophilus	uncultured bacterium; P1D1-751; EF511877
			Bacteria	Proteobacteria	Gammaproteobacteria	Pasteurellales	Pasteurellaceae	Haemophilus	uncultured bacterium; P1D1-573; EF511884
			Bacteria	Proteobacteria	Gammaproteobacteria	Pasteurellales	Pasteurellaceae	Haemophilus	uncultured bacterium; P1D1-521; EF511891
			Bacteria	Proteobacteria	Gammaproteobacteria	Pasteurellales	Pasteurellaceae	Haemophilus	uncultured bacterium; P1D1-523; EF511918
			Bacteria	Proteobacteria	Gammaproteobacteria	Pasteurellales	Pasteurellaceae	Haemophilus	Haemophilus influenzae; 2406; X87979
			Bacteria	Proteobacteria	Gammaproteobacteria	Pasteurellales	Pasteurellaceae	Haemophilus	Haemophilus influenzae; 16N; X87978
			Bacteria	Proteobacteria	Gammaproteobacteria	Pasteurellales	Pasteurellaceae	Haemophilus	Haemophilus quentini; S32F2; AY962106
o1750	o1750 16S-CamSp	CCATAGTGGAGGACAACAGT	Bacteria	Proteobacteria	Epsilonproteobacteria	Campylobacterales	Campylobacteraceae	Campylobacter	Campylobacter curvus; LMG 11034; AF550650
			Bacteria	Proteobacteria	Epsilonproteobacteria	Campylobacterales	Campylobacteraceae	Campylobacter	Campylobacter curvus; LMG 11127; AF550651
			Bacteria	Proteobacteria	Epsilonproteobacteria	Campylobacterales	Campylobacteraceae	Campylobacter	Campylobacter curvus; LMG 13936; AF550652
			Bacteria	Proteobacteria	Epsilonproteobacteria	Campylobacterales	Campylobacteraceae	Campylobacter	Campylobacter sp. oral clone BB120; AY005038
			Bacteria	Proteobacteria	Epsilonproteobacteria	Campylobacterales	Campylobacteraceae	Campylobacter	Campylobacter curvus; ATCC 35224; DQ174165
			Bacteria	Proteobacteria	Epsilonproteobacteria	Campylobacterales	Campylobacteraceae	Campylobacter	uncultured bacterium; 284; GQ398428
			Bacteria	Proteobacteria	Epsilonproteobacteria	Campylobacterales	Campylobacteraceae	Campylobacter	uncultured Campylobacter sp.; 202B08(oral); AM420041
			Bacteria	Proteobacteria	Epsilonproteobacteria	Campylobacterales	Campylobacteraceae	Campylobacter	uncultured Campylobacter sp.; 3178; FJ976205
o1751	o1751 16S-CapSp	GTCACCGCAAGGAGCTGTT	Bacteria	Bacteroidetes	Flavobacteria	Flavobacteriales	Flavobacteriaceae	Capnocytophaga	uncultured bacterium; A_S_01_44; FJ470580
			Bacteria	Bacteroidetes	Flavobacteria	Flavobacteriales	Flavobacteriaceae	Capnocytophaga	Capnocytophaga sp. AHN9528; DQ012324
			Bacteria	Bacteroidetes	Flavobacteria	Flavobacteriales	Flavobacteriaceae	Capnocytophaga	Capnocytophaga sp. AHN9576; DQ012327
			Bacteria	Bacteroidetes	Flavobacteria	Flavobacteriales	Flavobacteriaceae	Capnocytophaga	Capnocytophaga sp. AHN9607; DQ012328
			Bacteria	Bacteroidetes	Flavobacteria	Flavobacteriales	Flavobacteriaceae	Capnocytophaga	Capnocytophaga sp. oral clone EL043; AY008312
			Bacteria	Bacteroidetes	Flavobacteria	Flavobacteriales	Flavobacteriaceae	Capnocytophaga	Capnocytophaga sp. AHN8471; DQ009622
			Bacteria	Bacteroidetes	Flavobacteria	Flavobacteriales	Flavobacteriaceae	Capnocytophaga	Capnocytophaga sp. AHN9798; DQ012325
			Bacteria	Bacteroidetes	Flavobacteria	Flavobacteriales	Flavobacteriaceae	Capnocytophaga	Capnocytophaga ochracea; ChDC OS42; AF543292
			Bacteria	Bacteroidetes	Flavobacteria	Flavobacteriales	Flavobacteriaceae	Capnocytophaga	Capnocytophaga ochracea; ChDC OS48; AF543298
			Bacteria	Bacteroidetes	Flavobacteria	Flavobacteriales	Flavobacteriaceae	Capnocytophaga	Capnocytophaga sp. ChDC OS44; AF543294
			Bacteria	Bacteroidetes	Flavobacteria	Flavobacteriales	Flavobacteriaceae	Capnocytophaga	Capnocytophaga sp. P2 oral strain P4P_12; AY429469
			Bacteria	Bacteroidetes	Flavobacteria	Flavobacteriales	Flavobacteriaceae	Capnocytophaga	uncultured Capnocytophaga sp.; 602A12(oral); AM420232
			Bacteria	Bacteroidetes	Flavobacteria	Flavobacteriales	Flavobacteriaceae	Capnocytophaga	Capnocytophaga sp. oral strain A47ROY; AY005077
			Bacteria	Bacteroidetes	Flavobacteria	Flavobacteriales	Flavobacteriaceae	Capnocytophaga	Capnocytophaga sp. ChDC OS43; AF543293
			Bacteria	Bacteroidetes	Flavobacteria	Flavobacteriales	Flavobacteriaceae	Capnocytophaga	Capnocytophaga sp. AHN8751; DQ012323
o1752	o1752 16S-CapHae	CCGATAGCGAAGGCATGTTA	Bacteria	Bacteroidetes	Flavobacteria	Flavobacteriales	Flavobacteriaceae	Capnocytophaga	Capnocytophaga haemolytica; A0404; DQ009624
			Bacteria	Bacteroidetes	Flavobacteria	Flavobacteriales	Flavobacteriaceae	Capnocytophaga	Capnocytophaga haemolytica; LMG 16021 T; JCM 8565 T; U41349
			Bacteria	Bacteroidetes	Flavobacteria	Flavobacteriales	Flavobacteriaceae	Capnocytophaga	Capnocytophaga haemolytica (T); ATCC 51501; X97247
o1753	o1753 16S-PrePal	TGCACGTGTGCGTTATTGCA	Bacteria	Bacteroidetes	Bacteroidetes	Bacteroidales	Prevotellaceae	Prevotella	Prevotella sp. oral clone DO022; AF385519

			Bacteria	Bacteroidetes	Bacteroidetes	Bacteroidales	Prevotellaceae	Prevotella	uncultured Prevotella sp.; 401G10(oral); AM420128
			Bacteria	Bacteroidetes	Bacteroidetes	Bacteroidales	Prevotellaceae	Prevotella	uncultured bacterium; P3D5-486; EF508938
			Bacteria	Bacteroidetes	Bacteroidetes	Bacteroidales	Prevotellaceae	Prevotella	Prevotella pallens (T); 10371; Y13105
			Bacteria	Bacteroidetes	Bacteroidetes	Bacteroidales	Prevotellaceae	Prevotella	Prevotella pallens; 9423; Y13106
			Bacteria	Bacteroidetes	Bacteroidetes	Bacteroidales	Prevotellaceae	Prevotella	Prevotella pallens; 8792; Y13107
			Bacteria	Bacteroidetes	Bacteroidetes	Bacteroidales	Prevotellaceae	Prevotella	uncultured bacterium; nbw893d03c1; GQ025654
			Bacteria	Bacteroidetes	Bacteroidetes	Bacteroidales	Prevotellaceae	Prevotella	uncultured bacterium; nbw831g11c1; GQ009695
			Bacteria	Bacteroidetes	Bacteroidetes	Bacteroidales	Prevotellaceae	Prevotella	uncultured bacterium; nbw829f03c1; GQ009292
			Bacteria	Bacteroidetes	Bacteroidetes	Bacteroidales	Prevotellaceae	Prevotella	uncultured bacterium; nbw831b07c1; GQ009655
			Bacteria	Bacteroidetes	Bacteroidetes	Bacteroidales	Prevotellaceae	Prevotella	uncultured bacterium; nbw893c09c1; GQ025650
			Bacteria	Bacteroidetes	Bacteroidetes	Bacteroidales	Prevotellaceae	Prevotella	uncultured bacterium; nbw893f07c1; GQ025664
			Bacteria	Bacteroidetes	Bacteroidetes	Bacteroidales	Prevotellaceae	Prevotella	uncultured bacterium; nbw893f09c1; GQ025666
			Bacteria	Bacteroidetes	Bacteroidetes	Bacteroidales	Prevotellaceae	Prevotella	uncultured bacterium; nbw893f10c1; GQ025667
			Bacteria	Bacteroidetes	Bacteroidetes	Bacteroidales	Prevotellaceae	Prevotella	uncultured bacterium; nbu86f07c1; GQ006703
			Bacteria	Bacteroidetes	Bacteroidetes	Bacteroidales	Prevotellaceae	Prevotella	uncultured bacterium; nbw828c10c1; GQ009188
			Bacteria	Bacteroidetes	Bacteroidetes	Bacteroidales	Prevotellaceae	Prevotella	uncultured bacterium; nbw829a11c1; GQ009243
			Bacteria	Bacteroidetes	Bacteroidetes	Bacteroidales	Prevotellaceae	Prevotella	uncultured bacterium; nbw830c12c1; GQ009609
			Bacteria	Bacteroidetes	Bacteroidetes	Bacteroidales	Prevotellaceae	Prevotella	uncultured bacterium; nbw831d05c1; GQ009671
			Bacteria	Bacteroidetes	Bacteroidetes	Bacteroidales	Prevotellaceae	Prevotella	uncultured bacterium; nbw829h02c1; GQ009311
o1754	o1754 16S-BerSp	TGCGGAACATGTGTGCAATC	Bacteria	Bacteroidetes	Flavobacteria	Flavobacteriales	Flavobacteriaceae	unclassified_Flavobacteriaceae	uncultured Bergeyella sp.; 602D02(oral); AM420234
			Bacteria	Bacteroidetes	Flavobacteria	Flavobacteriales	Flavobacteriaceae	unclassified_Flavobacteriaceae	uncultured Bergeyella sp.; 450a; EU644465
			Bacteria	Bacteroidetes	Flavobacteria	Flavobacteriales	Flavobacteriaceae	unclassified_Flavobacteriaceae	Bergeyella sp. oral clone AK152; AY008691
			Bacteria	Bacteroidetes	Flavobacteria	Flavobacteriales	Flavobacteriaceae	unclassified_Flavobacteriaceae	uncultured bacterium; nbw491b01c1; GQ101136
			Bacteria	Bacteroidetes	Flavobacteria	Flavobacteriales	Flavobacteriaceae	unclassified_Flavobacteriaceae	uncultured bacterium; nbw492d08c1; GQ101217
			Bacteria	Bacteroidetes	Flavobacteria	Flavobacteriales	Flavobacteriaceae	unclassified_Flavobacteriaceae	uncultured bacterium; nbw205f11c1; GQ073152
			Bacteria	Bacteroidetes	Flavobacteria	Flavobacteriales	Flavobacteriaceae	unclassified_Flavobacteriaceae	uncultured bacterium; nbw164a01c1; GQ074513
			Bacteria	Bacteroidetes	Flavobacteria	Flavobacteriales	Flavobacteriaceae	unclassified_Flavobacteriaceae	uncultured bacterium; nbw491g09c1; GQ101166
			Bacteria	Bacteroidetes	Flavobacteria	Flavobacteriales	Flavobacteriaceae	unclassified_Flavobacteriaceae	uncultured Bergeyella sp.; 12B448; FJ976338
			Bacteria	Bacteroidetes	Flavobacteria	Flavobacteriales	Flavobacteriaceae	unclassified_Flavobacteriaceae	uncultured bacterium; nbw791g04c1; GQ011168
			Bacteria	Bacteroidetes	Flavobacteria	Flavobacteriales	Flavobacteriaceae	unclassified_Flavobacteriaceae	uncultured bacterium; nbw224b09c1; GQ075945
o1755	o1755 16S-AtoFos	ATAGCTACGGGAAACCGTAG	Bacteria	Actinobacteria	Actinobacteria	Coriobacteriales	Coriobacteriaceae	Atopobium	Atopobium fossor; JCM 9981; AB015945
			Bacteria	Actinobacteria	Actinobacteria	Coriobacteriales	Coriobacteriaceae	Atopobium	Atopobium fossor (T); L34620
o1756	o1756 16S-AtoMin	ATGGCACACAAGACAAAGCG	Bacteria	Actinobacteria	Actinobacteria	Coriobacteriales	Coriobacteriaceae	Atopobium	Atopobium minutum; NCFB 2751; X67148
			Bacteria	Actinobacteria	Actinobacteria	Coriobacteriales	Coriobacteriaceae	Atopobium	Atopobium minutum (T); M59059
			Bacteria	Actinobacteria	Actinobacteria	Coriobacteriales	Coriobacteriaceae	Atopobium	Atopobium minutum; S44204
o1757	o1757 16S-TreSp	AGGGCGATCGGACACATTG	Bacteria	Spirochaetes	Spirochaetes	Spirochaetales	Spirochaetaceae	Treponema	Treponema sp. I:8:G57; I:P:G57; AF056337
o1758	o1758 16S-CamHom2	ACTCGAAGCGGGGATGCAA	Bacteria	Proteobacteria	Epsilonproteobacteria	Campylobacterales	Campylobacteraceae	Campylobacter	Campylobacter hominis ATCC BAA-381; CP000776
			Bacteria	Proteobacteria	Epsilonproteobacteria	Campylobacterales	Campylobacteraceae	Campylobacter	Campylobacter hominis ATCC BAA-381; CP000776
			Bacteria	Proteobacteria	Epsilonproteobacteria	Campylobacterales	Campylobacteraceae	Campylobacter	Campylobacter hominis ATCC BAA-381; CP000776
			Bacteria	Proteobacteria	Epsilonproteobacteria	Campylobacterales	Campylobacteraceae	Campylobacter	Campylobacter hominis; HS-B; AF062491
			Bacteria	Proteobacteria	Epsilonproteobacteria	Campylobacterales	Campylobacteraceae	Campylobacter	Campylobacter hominis; HS-A; AF062490
			Bacteria	Proteobacteria	Epsilonproteobacteria	Campylobacterales	Campylobacteraceae	Campylobacter	Campylobacter hominis; HS-C; AF062492
			Bacteria	Proteobacteria	Epsilonproteobacteria	Campylobacterales	Campylobacteraceae	Campylobacter	Campylobacter hominis (T); NCTC 13146; CH001A; AJ251584
			Bacteria	Proteobacteria	Epsilonproteobacteria	Campylobacterales	Campylobacteraceae	Campylobacter	Campylobacter hominis; UNSWCD; GQ167659
o1760	o1760 16S-StrSal2	GGGCAAATGCTCCACTACAA	Bacteria	Firmicutes	Bacilli	Lactobacillales	Streptococcaceae	Streptococcus	Streptococcus salivarius; AF459433
			Bacteria	Firmicutes	Bacilli	Lactobacillales	Streptococcaceae	Streptococcus	uncultured bacterium; 014B-H10; DQ905624
			Bacteria	Firmicutes	Bacilli	Lactobacillales	Streptococcaceae	Streptococcus	uncultured bacterium; SJTU_C_02_44; EF403924
			Bacteria	Firmicutes	Bacilli	Lactobacillales	Streptococcaceae	Streptococcus	uncultured bacterium; SJTU_C_09_54; EF404405
			Bacteria	Firmicutes	Bacilli	Lactobacillales	Streptococcaceae	Streptococcus	uncultured bacterium; SJTU_F_03_24; EF398508
			Bacteria	Firmicutes	Bacilli	Lactobacillales	Streptococcaceae	Streptococcus	uncultured bacterium; SJTU_F_04_26; EF398592
			Bacteria	Firmicutes	Bacilli	Lactobacillales	Streptococcaceae	Streptococcus	uncultured bacterium; SJTU_F_09_45; EF398951
			Bacteria	Firmicutes	Bacilli	Lactobacillales	Streptococcaceae	Streptococcus	uncultured bacterium; SJTU_C_03_56; EF404014
			Bacteria	Firmicutes	Bacilli	Lactobacillales	Streptococcaceae	Streptococcus	uncultured bacterium; BF0002A135; AM696950
			Bacteria	Firmicutes	Bacilli	Lactobacillales	Streptococcaceae	Streptococcus	Streptococcus salivarius; M58839
			Bacteria	Firmicutes	Bacilli	Lactobacillales	Streptococcaceae	Streptococcus	uncultured bacterium; 2-002-b7; DQ905025
			Bacteria	Firmicutes	Bacilli	Lactobacillales	Streptococcaceae	Streptococcus	human oral bacterium C23; AF202012
			Bacteria	Firmicutes	Bacilli	Lactobacillales	Streptococcaceae	Streptococcus	uncultured bacterium; RL186_aah62b10; DQ806173
			Bacteria	Firmicutes	Bacilli	Lactobacillales	Streptococcaceae	Streptococcus	uncultured bacterium; RL180_aao74b10; DQ808231
			Bacteria	Firmicutes	Bacilli	Lactobacillales	Streptococcaceae	Streptococcus	uncultured bacterium; RL203_aai62a07; DQ805392
			Bacteria	Firmicutes	Bacilli	Lactobacillales	Streptococcaceae	Streptococcus	uncultured bacterium; TS25_a04b05; FJ366220
			Bacteria	Firmicutes	Bacilli	Lactobacillales	Streptococcaceae	Streptococcus	uncultured bacterium; TS21_a01d05; FJ365649
			Bacteria	Firmicutes	Bacilli	Lactobacillales	Streptococcaceae	Streptococcus	uncultured bacterium; TS149_a01d10; FJ363284
			Bacteria	Firmicutes	Bacilli	Lactobacillales	Streptococcaceae	Streptococcus	uncultured bacterium; LY03; AY976333
			Bacteria	Firmicutes	Bacilli	Lactobacillales	Streptococcaceae	Streptococcus	uncultured bacterium; C1_734; EU767000
o1761	o1761 16S-AerUri	AGCGACCTTGTGAAAGCAAG	Bacteria	Firmicutes	Bacilli	Lactobacillales	Aerococcaceae	Aerococcus	Aerococcus urinae; 1656-92; U64458
			Bacteria	Firmicutes	Bacilli	Lactobacillales	Aerococcaceae	Aerococcus	Aerococcus urinae; CDC 1515(85); AY422712
			Bacteria	Firmicutes	Bacilli	Lactobacillales	Aerococcaceae	Aerococcus	Aerococcus urinae; NL 95028; AY422713
			Bacteria	Firmicutes	Bacilli	Lactobacillales	Aerococcaceae	Aerococcus	Aerococcus urinae; NL 95012; AY422714
			Bacteria	Firmicutes	Bacilli	Lactobacillales	Aerococcaceae	Aerococcus	Aerococcus urinae; CDC 3352(95); AY422715
			Bacteria	Firmicutes	Bacilli	Lactobacillales	Aerococcaceae	Aerococcus	Aerococcus urinae; CCUG 32514; AY422716
			Bacteria	Firmicutes	Bacilli	Lactobacillales	Aerococcaceae	Aerococcus	Aerococcus urinae; NL 95031; AY422711
			Bacteria	Firmicutes	Bacilli	Lactobacillales	Aerococcaceae	Aerococcus	Aerococcus urinae; DK E2T; AY422717
			Bacteria	Firmicutes	Bacilli	Lactobacillales	Aerococcaceae	Aerococcus	Aerococcus urinae; 944-94; U64459
			Bacteria	Firmicutes	Bacilli	Lactobacillales	Aerococcaceae	Aerococcus	uncultured bacterium; BF0002C045; AM697348
			Bacteria	Firmicutes	Bacilli	Lactobacillales	Aerococcaceae	Aerococcus	Aerococcus urinae; G1-84; U64457
			Bacteria	Firmicutes	Bacilli	Lactobacillales	Aerococcaceae	Aerococcus	Aerococcus urinae (T); M77819
			Bacteria	Firmicutes	Bacilli	Lactobacillales	Aerococcaceae	Aerococcus	Aerococcus urinae; F1-84; U64456
			Bacteria	Firmicutes	Bacilli	Lactobacillales	Aerococcaceae	Aerococcus	Aerococcus urinae; 998-93; U64460
o1762	o1762 16S-GraEle	CCATCAGAGGGGGATAACAT	Bacteria	Firmicutes	Bacilli	Lactobacillales	Carnobacteriaceae	Granulicatella	Granulicatella sp. oral clone ASCA05; DQ341469
			Bacteria	Firmicutes	Bacilli	Lactobacillales	Carnobacteriaceae	Granulicatella	Granulicatella elegans (T); DSM 11693; B1333; AF016390
			Bacteria	Firmicutes	Bacilli	Lactobacillales	Carnobacteriaceae	Granulicatella	Granulicatella sp. oral clone ASCG05; AY923146
			Bacteria	Firmicutes	Bacilli	Lactobacillales	Carnobacteriaceae	Granulicatella	Granulicatella adiacens; 04356101; AY879304
			Bacteria	Firmicutes	Bacilli	Lactobacillales	Carnobacteriaceae	Granulicatella	Granulicatella adiacens; 02357001; AY879302
			Bacteria	Firmicutes	Bacilli	Lactobacillales	Carnobacteriaceae	Granulicatella	Granulicatella adiacens; 04424062; AY879305
			Bacteria	Firmicutes	Bacilli	Lactobacillales	Carnobacteriaceae	Granulicatella	Granulicatella adiacens; 03385274; AY879300
			Bacteria	Firmicutes	Bacilli	Lactobacillales	Carnobacteriaceae	Granulicatella	Granulicatella adiacens; 04305056; AY879303
			Bacteria	Firmicutes	Bacilli	Lactobacillales	Carnobacteriaceae	Granulicatella	Granulicatella sp. oral clone ASCB09; AY953251
			Bacteria	Firmicutes	Bacilli	Lactobacillales	Carnobacteriaceae	Granulicatella	Granulicatella adiacens; 03252090; AY879299

			Bacteria	Firmicutes	Bacilli	Lactobacillales	Carnobacteriaceae	Granulicatella	Granulicatella para-adiacens; 03476079; AY879301
			Bacteria	Firmicutes	Bacilli	Lactobacillales	Carnobacteriaceae	Granulicatella	Granulicatella para-adiacens; 04401102; AY879298
			Bacteria	Firmicutes	Bacilli	Lactobacillales	Carnobacteriaceae	Granulicatella	Granulicatella adiacens; 10/833; AJ312375
			Bacteria	Firmicutes	Bacilli	Lactobacillales	Carnobacteriaceae	Granulicatella	Granulicatella elegans; 4067-96; Y15413
			Bacteria	Firmicutes	Bacilli	Lactobacillales	Carnobacteriaceae	Granulicatella	uncultured organism; MC0604c8; AY897990
			Bacteria	Firmicutes	Bacilli	Lactobacillales	Carnobacteriaceae	Granulicatella	Abiotrophia para-adiacens; TKT1; AB022027
			Bacteria	Firmicutes	Bacilli	Lactobacillales	Carnobacteriaceae	Granulicatella	Granulicatella elegans; HHC5; AB022026
			Bacteria	Firmicutes	Bacilli	Lactobacillales	Carnobacteriaceae	Granulicatella	uncultured bacterium; LC1022b35; DQ228568
			Bacteria	Firmicutes	Bacilli	Lactobacillales	Carnobacteriaceae	Granulicatella	Granulicatella elegans; 2640-91; Y15408
			Bacteria	Firmicutes	Bacilli	Lactobacillales	Carnobacteriaceae	Granulicatella	Abiotrophia sp. oral clone OH2A; AY947496
o1763	o1763 16S-PreSp	AGTCGTCGGAAGTCATCTGA	Bacteria	Bacteroidetes	Bacteroidetes	Bacteroidales	Prevotellaceae	Prevotella	uncultured Prevotellaceae bacterium; 201B11(oral); AM420018
			Bacteria	Bacteroidetes	Bacteroidetes	Bacteroidales	Prevotellaceae	Prevotella	Prevotella maculosa; W1609; EF534314
			Bacteria	Bacteroidetes	Bacteroidetes	Bacteroidales	Prevotellaceae	Prevotella	Prevotella maculosa; W1609; EF534315
			Bacteria	Bacteroidetes	Bacteroidetes	Bacteroidales	Prevotellaceae	Prevotella	Prevotella sp. oral clone AA016; AY005058
			Bacteria	Bacteroidetes	Bacteroidetes	Bacteroidales	Prevotellaceae	Prevotella	Prevotella sp. oral clone AA020; AY005057
			Bacteria	Bacteroidetes	Bacteroidetes	Bacteroidales	Prevotellaceae	Prevotella	uncultured Prevotella sp.; 10B071; FJ976297
			Bacteria	Bacteroidetes	Bacteroidetes	Bacteroidales	Prevotellaceae	Prevotella	uncultured Prevotella sp.; 18B421; FJ976367
o1764	o1764 16S-ActSpRotS	CAATCTCTGCATGGGGTGTT	Bacteria	Actinobacteria	Actinobacteria	Actinomycetales	Micrococcaceae	Rothia	Rothia sp. ChDC B201; AF543279
			Bacteria	Actinobacteria	Actinobacteria	Actinomycetales	Micrococcaceae	Rothia	Rothia sp. ChDC B202; AF543280
			Bacteria	Actinobacteria	Actinobacteria	Actinomycetales	Micrococcaceae	Rothia	Rothia sp. J03; DQ409139
			Bacteria	Actinobacteria	Actinobacteria	Actinomycetales	Micrococcaceae	Rothia	uncultured Rothia sp.; EHFS1_S03f; EU071477
			Bacteria	Actinobacteria	Actinobacteria	Actinomycetales	Micrococcaceae	Rothia	uncultured bacterium; A_D_01_95; FJ470448
			Bacteria	Actinobacteria	Actinobacteria	Actinomycetales	Micrococcaceae	Rothia	Rothia sp. CCUG 25688; AJ131122
			Bacteria	Actinobacteria	Actinobacteria	Actinomycetales	Micrococcaceae	Rothia	uncultured bacterium; FB04G03; FM872928
			Bacteria	Actinobacteria	Actinobacteria	Actinomycetales	Micrococcaceae	Rothia	Actinomyces sp.; CCUG 25688; Y13025
			Bacteria	Actinobacteria	Actinobacteria	Actinomycetales	Micrococcaceae	Rothia	Rothia sp. Smarlab 3302411; AY538697
			Bacteria	Actinobacteria	Actinobacteria	Actinomycetales	Micrococcaceae	Rothia	uncultured Rothia sp.; 603015(oral); AM420244
			Bacteria	Actinobacteria	Actinobacteria	Actinomycetales	Micrococcaceae	Rothia	Arthrobacter sp. B10; AF332382
			Bacteria	Actinobacteria	Actinobacteria	Actinomycetales	Micrococcaceae	Rothia	Rothia aeria; Lan; EU293888
			Bacteria	Actinobacteria	Actinobacteria	Actinomycetales	Micrococcaceae	Rothia	Rothia aeria; SMC-A5889; DQ673319
			Bacteria	Actinobacteria	Actinobacteria	Actinomycetales	Micrococcaceae	Rothia	uncultured bacterium; nbw109f09c1; GQ007787
			Bacteria	Actinobacteria	Actinobacteria	Actinomycetales	Micrococcaceae	Rothia	uncultured bacterium; nbw110a03c1; GQ007813
			Bacteria	Actinobacteria	Actinobacteria	Actinomycetales	Micrococcaceae	Rothia	uncultured bacterium; nbw110c07c1; GQ007837
			Bacteria	Actinobacteria	Actinobacteria	Actinomycetales	Micrococcaceae	Rothia	uncultured bacterium; nbu172h03c1; GQ001424
			Bacteria	Actinobacteria	Actinobacteria	Actinomycetales	Micrococcaceae	Rothia	Rothia aeria (T); GTC867; AB071952
			Bacteria	Actinobacteria	Actinobacteria	Actinomycetales	Micrococcaceae	Rothia	uncultured bacterium; CD64; DQ441315
			Bacteria	Actinobacteria	Actinobacteria	Actinomycetales	Micrococcaceae	Rothia	Rothia aeria; SMC-A2662; DQ673320
o1766	o1766 16S-NeiCan	CTTGGTCTTGACATGTACGG	Bacteria	Proteobacteria	Betaproteobacteria	Neisseriales	Neisseriaceae	Conchiformibius	uncultured bacterium; nbw658d03c1; GQ110342
o1768	o1768 16S-NeiSicFla	GACATGTGCGGAACCCTCCA	Bacteria	Proteobacteria	Betaproteobacteria	Neisseriales	Neisseriaceae	Neisseria	uncultured bacterium; nbu203f01c1; GQ020164
			Bacteria	Proteobacteria	Betaproteobacteria	Neisseriales	Neisseriaceae	Neisseria	uncultured bacterium; nbw868f03c1; GQ030194
			Bacteria	Proteobacteria	Betaproteobacteria	Neisseriales	Neisseriaceae	Neisseria	uncultured bacterium; nbu141c05c1; GQ018639
			Bacteria	Proteobacteria	Betaproteobacteria	Neisseriales	Neisseriaceae	Neisseria	uncultured bacterium; nbw865b06c1; GQ029949
			Bacteria	Proteobacteria	Betaproteobacteria	Neisseriales	Neisseriaceae	Neisseria	uncultured bacterium; nbw110f01c1; GQ007861
			Bacteria	Proteobacteria	Betaproteobacteria	Neisseriales	Neisseriaceae	Neisseria	uncultured bacterium; nbu203e01c1; GQ020152
			Bacteria	Proteobacteria	Betaproteobacteria	Neisseriales	Neisseriaceae	Neisseria	uncultured bacterium; nbu443c04c1; GQ051260
			Bacteria	Actinobacteria	Actinobacteria	Actinomycetales	Actinomycetaceae	Actinomyces	uncultured bacterium; FA04B03; FM872615
o1769	o1769 16S-ActSp	GCTTGACATGTGCCGGTTTG	Bacteria	Actinobacteria	Actinobacteria	Actinomycetales	Actinomycetaceae	Actinomyces	uncultured bacterium; MA03A07; FM873548
			Bacteria	Actinobacteria	Actinobacteria	Actinomycetales	Actinomycetaceae	Actinomyces	Actinomyces sp. oral clone AP064; AF287749
			Bacteria	Actinobacteria	Actinobacteria	Actinomycetales	Actinomycetaceae	Actinomyces	uncultured bacterium; nbw203f12c1; GQ072987
			Bacteria	Actinobacteria	Actinobacteria	Actinomycetales	Actinomycetaceae	Actinomyces	uncultured bacterium; nbw1156b11c1; GQ077217
			Bacteria	Actinobacteria	Actinobacteria	Actinomycetales	Actinomycetaceae	Actinomyces	uncultured bacterium; nbw200c06c1; GQ072716
			Bacteria	Actinobacteria	Actinobacteria	Actinomycetales	Actinomycetaceae	Actinomyces	uncultured bacterium; nbw1155e01c1; GQ076938
			Bacteria	Actinobacteria	Actinobacteria	Actinomycetales	Actinomycetaceae	Actinomyces	uncultured bacterium; nbw227e12c1; GQ069014
			Bacteria	Actinobacteria	Actinobacteria	Actinomycetales	Actinomycetaceae	Actinomyces	uncultured bacterium; nbw145c02c1; GQ067994
			Bacteria	Actinobacteria	Actinobacteria	Actinomycetales	Actinomycetaceae	Actinomyces	uncultured bacterium; nbw208h11c1; GQ073428
o1770	o1770 16S-TM7-seq08	CAATTAGTGCCTTTTGGAACC	Bacteria	TM7				TM7_genera_incertae_sedis	uncultured bacterium; UTFS-OF09-d22-22; AB200303
			Bacteria	TM7				TM7_genera_incertae_sedis	uncultured candidate division TM7 bacterium; 401H12(oral); AM420132
			Bacteria	TM7				TM7_genera_incertae_sedis	uncultured bacterium; 14; FJ534960
			Bacteria	TM7				TM7_genera_incertae_sedis	uncultured bacterium; UTFS-OF09-d22-24; AB200299
			Bacteria	TM7				TM7_genera_incertae_sedis	uncultured bacterium; nbw202b07c1; GQ072867
			Bacteria	TM7				TM7_genera_incertae_sedis	uncultured candidate division TM7 bacterium; 6BB503; FJ976255
			Bacteria	TM7				TM7_genera_incertae_sedis	uncultured bacterium AH040; AF125204
o1771	o1771 16S-TM7-seq17	TTGTGCCTTTTGGAACTCGG	Bacteria	TM7				TM7_genera_incertae_sedis	TM7 phylum sp. oral taxon 348; BN036; GQ422738
			Bacteria	TM7				TM7_genera_incertae_sedis	uncultured candidate division TM7 bacterium; 7BB623; FJ976276
o1773	o1773 16S-seq087(332	GCTCTGTGAAGGTGCTGCAT	Bacteria	Bacteroidetes	Bacteroidetes	Bacteroidales	Prevotellaceae	Prevotella	uncultured bacterium; 47; GQ398420
o1774	o1774 16S-ProPro-seq	GATGGGCGTGCCTTTTTGGT	Bacteria	Actinobacteria	Actinobacteria	Actinomycetales	Propionibacteriaceae	unclassified_Propionibacteriaceae	Propionibacterium propionicum; DSM 43307T; AJ315953
			Bacteria	Actinobacteria	Actinobacteria	Actinomycetales	Propionibacteriaceae	unclassified_Propionibacteriaceae	uncultured bacterium; A_D_03_05; FJ470486
			Bacteria	Actinobacteria	Actinobacteria	Actinomycetales	Propionibacteriaceae	unclassified_Propionibacteriaceae	uncultured Propionibacterium sp.; 402A10(oral); AM420135
			Bacteria	Actinobacteria	Actinobacteria	Actinomycetales	Propionibacteriaceae	unclassified_Propionibacteriaceae	Propionibacterium propionicum; VA1535_00; AF285117
			Bacteria	Actinobacteria	Actinobacteria	Actinomycetales	Propionibacteriaceae	unclassified_Propionibacteriaceae	uncultured Propionibacterium sp.; 6BB945; FJ976260
			Bacteria	Actinobacteria	Actinobacteria	Actinomycetales	Propionibacteriaceae	unclassified_Propionibacteriaceae	uncultured bacterium; nbw357b03c1; GQ087414
			Bacteria	Actinobacteria	Actinobacteria	Actinomycetales	Propionibacteriaceae	unclassified_Propionibacteriaceae	uncultured bacterium; nbw358g10c1; GQ087545
			Bacteria	Actinobacteria	Actinobacteria	Actinomycetales	Propionibacteriaceae	unclassified_Propionibacteriaceae	uncultured bacterium; nbw162c01c1; GQ074375
o1775	o1775 16S-SelSp-seq0	GTTAGAGATAGCTCCCTCTC	Bacteria	Firmicutes	Clostridia	Clostridiales	Veillonellaceae	Selenomonas	Selenomonas sp. oral taxon 140; EL028; GQ422720
			Bacteria	Firmicutes	Clostridia	Clostridiales	Veillonellaceae	Mitsuokella	uncultured bacterium; B1_278; EU764430
			Bacteria	Firmicutes	Clostridia	Clostridiales	Veillonellaceae	Mitsuokella	uncultured bacterium; B1_092; EU764619
o1776	o1776 16S-seq101(244	ACAGAGGTAGAGATACCTTC	Bacteria	Bacteroidetes	Flavobacteria	Flavobacteriales	Flavobacteriaceae	Capnocytophaga	uncultured bacterium; A_S_01_44; FJ470580
o1777	o1777 16S-StrMut4	GGAAGTTACTTCGGTACATCGG	Bacteria	Firmicutes	Bacilli	Lactobacillales	Streptococcaceae	Streptococcus	Streptococcus mutans; UA96; AF139600
			Bacteria	Firmicutes	Bacilli	Lactobacillales	Streptococcaceae	Streptococcus	Streptococcus mutans; AF199; AF139602
			Bacteria	Firmicutes	Bacilli	Lactobacillales	Streptococcaceae	Streptococcus	Streptococcus mutans; LM7; AF139601
			Bacteria	Firmicutes	Bacilli	Lactobacillales	Streptococcaceae	Streptococcus	Streptococcus mutans; 669; AF139603
			Bacteria	Firmicutes	Bacilli	Lactobacillales	Streptococcaceae	Streptococcus	Streptococcus mutans; UA140; AF139599
			Bacteria	Firmicutes	Bacilli	Lactobacillales	Streptococcaceae	Streptococcus	Streptococcus mutans; ATCC 25175; AY188348
			Bacteria	Firmicutes	Bacilli	Lactobacillales	Streptococcaceae	Streptococcus	Streptococcus mutans; ChDC YM9; AY691527
			Bacteria	Firmicutes	Bacilli	Lactobacillales	Streptococcaceae	Streptococcus	Streptococcus mutans; ChDC YM71; AY691528
			Bacteria	Firmicutes	Bacilli	Lactobacillales	Streptococcaceae	Streptococcus	Streptococcus mutans; ChDC YM3; AY691526
			Bacteria	Firmicutes	Bacilli	Lactobacillales	Streptococcaceae	Streptococcus	Streptococcus mutans; ChDC YM97; AY691532
			Bacteria	Firmicutes	Bacilli	Lactobacillales	Streptococcaceae	Streptococcus	Streptococcus mutans; ChDC YM83; AY691529

			Bacteria	Firmicutes	Bacilli	Lactobacillales	Streptococcaceae	Streptococcus	Streptococcus mutans; ChDC YM86; AY691530
			Bacteria	Firmicutes	Bacilli	Lactobacillales	Streptococcaceae	Streptococcus	Streptococcus mutans; NCTC 10449; AJ243965
			Bacteria	Firmicutes	Bacilli	Lactobacillales	Streptococcaceae	Streptococcus	Streptococcus mutans; UA96; AF077835
			Bacteria	Firmicutes	Bacilli	Lactobacillales	Streptococcaceae	Streptococcus	Streptococcus mutans; LM7; AF077832
			Bacteria	Firmicutes	Bacilli	Lactobacillales	Streptococcaceae	Streptococcus	Streptococcus mutans; AF199; AF077833
			Bacteria	Firmicutes	Bacilli	Lactobacillales	Streptococcaceae	Streptococcus	Streptococcus mutans; 669; AF077834
			Bacteria	Firmicutes	Bacilli	Lactobacillales	Streptococcaceae	Streptococcus	human oral bacterium C29; AF202013
			Bacteria	Firmicutes	Bacilli	Lactobacillales	Streptococcaceae	Streptococcus	Streptococcus peroris (T); GTC848; AB008314
			Bacteria	Firmicutes	Bacilli	Lactobacillales	Streptococcaceae	Streptococcus	Streptococcus mutans (T); NCTC 10449(T); X58303
o1779	o1779 16S-seq198(110	AGACTAGTCCTTCGGGACAA	Bacteria	Firmicutes	Clostridia	Clostridiales	Lachnospiraceae	unclassified_"Lachnospiraceae"	Lachnospiraceae bacterium 'Oral Taxon 107'; F0167; FJ577253
			Bacteria	Firmicutes	Clostridia	Clostridiales	Lachnospiraceae	unclassified_"Lachnospiraceae"	uncultured bacterium; nbw493d01c1; GQ101287
o1780	o1780 16S-seq213(100	AATGAGATTAGTCTTTCGGG	Bacteria	Firmicutes	Clostridia	Clostridiales	Lachnospiraceae	unclassified_"Lachnospiraceae"	uncultured bacterium; A_D_01_58; FJ470430
			Bacteria	Firmicutes	Clostridia	Clostridiales	Lachnospiraceae	unclassified_"Lachnospiraceae"	uncultured Lachnospiraceae bacterium; 7BB544; FJ976273
o1782	o1782 16S-seq222(94)	CTTGGAGGTGCTGCATGGTT	Bacteria	Bacteroidetes	Bacteroidetes	Bacteroidales	unclassified_Bacteroidales		Prevotella sp. oral clone ASCG10; AY923148
			Bacteria	Bacteroidetes	Bacteroidetes	Bacteroidales	Prevotellaceae	unclassified_Prevotellaceae	Prevotella sp. oral clone HF050; AY349399
			Bacteria	Bacteroidetes	Bacteroidetes	Bacteroidales	Prevotellaceae	unclassified_Prevotellaceae	Prevotella sp. oral clone IK062; AY349402
			Bacteria	Bacteroidetes	Bacteroidetes	Bacteroidales	Prevotellaceae	Prevotella	uncultured bacterium; 9; EU663608
			Bacteria	Bacteroidetes	Bacteroidetes	Bacteroidales	Prevotellaceae	Prevotella	uncultured Prevotellaceae bacterium; 301H01(oral); AM420077
			Bacteria	Bacteroidetes	Bacteroidetes	Bacteroidales	unclassified_Bacteroidales		uncultured Prevotellaceae bacterium; 701C02(oral); AM420250
			Bacteria	Bacteroidetes	Bacteroidetes	Bacteroidales	Prevotellaceae	unclassified_Prevotellaceae	uncultured Prevotellaceae bacterium; 301C01(oral); AM420060
			Bacteria	Bacteroidetes	Bacteroidetes	Bacteroidales	Prevotellaceae	Prevotella	Prevotella sp. oral clone BU035; AF385564
			Bacteria	Bacteroidetes	Bacteroidetes	Bacteroidales	unclassified_Bacteroidales		uncultured bacterium; FC04E06; FM873252
			Bacteria	Bacteroidetes	Bacteroidetes	Bacteroidales	Prevotellaceae	Prevotella	Prevotellaceae bacterium P4P_62 P1; AY207061
			Bacteria	Bacteroidetes	Bacteroidetes	Bacteroidales	Prevotellaceae	unclassified_Prevotellaceae	uncultured bacterium; P2D15-722; EF510713
			Bacteria	Bacteroidetes	Bacteroidetes	Bacteroidales	Prevotellaceae	unclassified_Prevotellaceae	uncultured Bacteroidales bacterium; EMP_B15; EU794130
			Bacteria	Bacteroidetes	Bacteroidetes	Bacteroidales	Prevotellaceae	unclassified_Prevotellaceae	uncultured bacterium; P2D15-729; EF510744
			Bacteria	Bacteroidetes	Bacteroidetes	Bacteroidales	unclassified_Bacteroidales		uncultured bacterium; P2D15-723; EF510690
			Bacteria	Bacteroidetes	Bacteroidetes	Bacteroidales	unclassified_Bacteroidales		uncultured bacterium; P2D15-732; EF510726
			Bacteria	Bacteroidetes	Bacteroidetes	Bacteroidales	Prevotellaceae	unclassified_Prevotellaceae	uncultured bacterium; CAP_aai00b10; EU459567
			Bacteria	Bacteroidetes	Bacteroidetes	Bacteroidales	Prevotellaceae	Prevotella	uncultured bacterium; I8-14; DQ113733
			Bacteria	Bacteroidetes	Bacteroidetes	Bacteroidales	Prevotellaceae	unclassified_Prevotellaceae	uncultured bacterium oral clone ASCD11; AY923131
			Bacteria	Bacteroidetes	Bacteroidetes	Bacteroidales	Prevotellaceae	unclassified_Prevotellaceae	uncultured Bacteroidetes bacterium; INT-G-7V-7; EU432416
			Bacteria	Bacteroidetes	Bacteroidetes	Bacteroidales	Prevotellaceae	unclassified_Prevotellaceae	uncultured Bacteroidetes bacterium; INT-G-7V-6; EU432417
o1318	o1318 16S-ActHer	CATCGGTTGGTGTGGAAAGT	Bacteria	Actinobacteria	Actinobacteria	Actinomycetales	Thermomonosporaceae	Actinocorallia	Actinocorallia herbida
o1416	o1416 16S-FleOral	CATGCGGCTTAACCGTGTTA	Bacteria	Firmicutes	Clostridia	Clostridiales	Incertae Sedis XV	unclassified_Incertae Sedis XV	uncultured rumen bacterium; A13; DQ085087
			Bacteria	Firmicutes	Clostridia	Clostridiales	Incertae Sedis XV	unclassified_Incertae Sedis XV	Deferribacteres sp. oral clone JV006; AY349371
			Bacteria	Firmicutes	Clostridia	Clostridiales	Incertae Sedis XV	unclassified_Incertae Sedis XV	uncultured Flexistipes sp.; 301F05(oral); AM420072
			Bacteria	Firmicutes	Clostridia	Clostridiales	Incertae Sedis XV	unclassified_Incertae Sedis XV	uncultured Deferribacteraceae bacterium; 501G12(oral); AM420177
			Bacteria	Firmicutes	Clostridia	Clostridiales	Incertae Sedis XV	unclassified_Incertae Sedis XV	Flexistipes-like sp. oral clone BB062; AY005445
			Bacteria	Firmicutes	Clostridia	Clostridiales	Incertae Sedis XV	unclassified_Incertae Sedis XV	uncultured bacterium; MA04D04; FM873769
			Bacteria	Firmicutes	Clostridia	Clostridiales	Incertae Sedis XV	unclassified_Incertae Sedis XV	Deferribacteres sp. oral clone JV023; AY349372
			Bacteria	Firmicutes	Clostridia	Clostridiales	Incertae Sedis XV	unclassified_Incertae Sedis XV	uncultured bacterium; MA04E05; FM873777
			Bacteria	Firmicutes	Clostridia	Clostridiales	Incertae Sedis XV	unclassified_Incertae Sedis XV	Firmicutes oral clone MCE7_5; AF481214
			Bacteria	Firmicutes	Clostridia	Clostridiales	Incertae Sedis XV	unclassified_Incertae Sedis XV	Deferribacteres sp. oral clone JV001; AY349370
			Bacteria	Firmicutes	Clostridia	Clostridiales	Incertae Sedis XV	unclassified_Incertae Sedis XV	uncultured bacterium D084; AF125200
			Bacteria	Firmicutes	Clostridia	Clostridiales	Incertae Sedis XV	unclassified_Incertae Sedis XV	uncultured bacterium; 1103200828008; EU844310
			Bacteria	Firmicutes	Clostridia	Clostridiales	Incertae Sedis XV	unclassified_Incertae Sedis XV	uncultured bacterium BH017; AF125199
			Bacteria	Firmicutes	Clostridia	Clostridiales	Incertae Sedis XV	unclassified_Incertae Sedis XV	Flexistipes-like sp. oral clone BH007; AY005447
			Bacteria	Firmicutes	Clostridia	Clostridiales	Incertae Sedis XV	unclassified_Incertae Sedis XV	uncultured bacterium D006; AF125201
			Bacteria	Firmicutes	Clostridia	Clostridiales	Incertae Sedis XV	unclassified_Incertae Sedis XV	uncultured Synergistetes bacterium; 7.4; GQ149245
			Bacteria	Firmicutes	Clostridia	Clostridiales	Incertae Sedis XV	unclassified_Incertae Sedis XV	uncultured bacterium; BOf7-03; AB288910
			Bacteria	Firmicutes	Clostridia	Clostridiales	Incertae Sedis XV	unclassified_Incertae Sedis XV	uncultured bacterium; Cc3-109; AB299562
			Bacteria	Firmicutes	Clostridia	Clostridiales	Incertae Sedis XV	unclassified_Incertae Sedis XV	uncultured bacterium; Rs-C88; AB089066
			Bacteria	Firmicutes	Clostridia	Clostridiales	Incertae Sedis XV	Aminobacterium	uncultured bacterium; Cc3-068; AB299561
o1493	o1493 16S-Pabas	CATGGCCGTTGGTGGTGC	Bacteria	Actinobacteria	Actinobacteria	Actinomycetales			Rhodococcus opacus B4; AP011115
			Bacteria	Actinobacteria	Actinobacteria	Actinomycetales			Rhodococcus opacus B4; AP011115
			Bacteria	Actinobacteria	Actinobacteria	Actinomycetales			Rhodococcus opacus B4; AP011115
			Bacteria	Actinobacteria	Actinobacteria	Actinomycetales			Rhodococcus opacus B4; AP011115
			Bacteria	Actinobacteria	Actinobacteria	Actinomycetales			Rhodococcus opacus; B-4; AB178563
			Bacteria	Actinobacteria	Actinobacteria	Actinomycetales			Rhodococcus opacus; B-4; AB192962
			Bacteria	Actinobacteria	Actinobacteria	Actinomycetales			Rhodococcus opacus; ML0004; DQ474758
o1590	o1590 16S-SR1	AGTGGCAAACGAGCGAGTAA		SR1				SR1_genera_incertae_sedis	uncultured freshwater bacterium LCK-15; AF107319
				SR1				SR1_genera_incertae_sedis	uncultured freshwater bacterium LCK-23; AF107321
				SR1				SR1_genera_incertae_sedis	uncultured bacterium X112; AF125207
				SR1				SR1_genera_incertae_sedis	uncultured candidate division SR1 bacterium; SRB2; AY193173
				SR1				SR1_genera_incertae_sedis	unidentified bacterium; BD2-14; AB015542
				SR1				SR1_genera_incertae_sedis	uncultured bacterium; Rs-J47; AB089125
				SR1				SR1_genera_incertae_sedis	uncultured candidate division OP11 bacterium; HsW01-043; AB231045
				SR1				SR1_genera_incertae_sedis	uncultured bacterium; E53-57; DQ639513
				SR1				SR1_genera_incertae_sedis	uncultured candidate division OP11 bacterium; MN051; AM157491
				SR1				SR1_genera_incertae_sedis	uncultured rumen bacterium; P5_J23; EU381782
				SR1				SR1_genera_incertae_sedis	uncultured rumen bacterium; P5_E10; EU381859
				SR1				SR1_genera_incertae_sedis	uncultured rumen bacterium; P5_L19; EU381883
				SR1				SR1_genera_incertae_sedis	uncultured rumen bacterium; P5_O10; EU382051
				SR1				SR1_genera_incertae_sedis	uncultured bacterium; MekkoE-149; AM949077
				SR1				SR1_genera_incertae_sedis	uncultured bacterium; IR_aaa02b12; EU474681
				SR1				SR1_genera_incertae_sedis	uncultured bacterium; M0111_69; EU104066
				SR1				SR1_genera_incertae_sedis	uncultured bacterium; A_M1_03_29; FJ470540
				SR1				SR1_genera_incertae_sedis	uncultured candidate division SR1 bacterium; DSR1-6; FJ479809
				SR1				SR1_genera_incertae_sedis	uncultured candidate division SR1 bacterium; DSR1-8; FJ479811
				SR1				SR1_genera_incertae_sedis	uncultured candidate division SR1 bacterium; DSR1-12; FJ479815
o1767	o1767 16S-VeiSp	TGTTCCTCTTCTTCGGAAGC	Bacteria	Firmicutes	Clostridia	Clostridiales	Veillonellaceae	Veillonella	Veillonella parvula; ATCC 17745; AY995769
			Bacteria	Firmicutes	Clostridia	Clostridiales	Veillonellaceae	Veillonella	Veillonella sp. oral clone X042; AF287781
			Bacteria	Firmicutes	Clostridia	Clostridiales	Veillonellaceae	Veillonella	uncultured Veillonella sp.; BU083; AF366266
			Bacteria	Firmicutes	Clostridia	Clostridiales	Veillonellaceae	Veillonella	Veillonella dispar (T); DSM 20735; X84006
			Bacteria	Firmicutes	Clostridia	Clostridiales	Veillonellaceae	Veillonella	Veillonella sp. oral clone AA050; AF287782
			Bacteria	Firmicutes	Clostridia	Clostridiales	Veillonellaceae	Veillonella	Veillonella atypica; ATCC 17744; AY995768
			Bacteria	Firmicutes	Clostridia	Clostridiales	Veillonellaceae	Veillonella	Veillonella dispar; ATCC 17748; AY995770

			Bacteria	Firmicutes	Clostridia	Clostridiales	Veillonellaceae	Veillonella	Veillonella atypica (T); DSM 20739; X84007
			Bacteria	Firmicutes	Clostridia	Clostridiales	Veillonellaceae	Veillonella	Veillonella parvula; ATCC 10790; AY995767
			Bacteria	Firmicutes	Clostridia	Clostridiales	Veillonellaceae	Veillonella	uncultured bacterium; p-5279-2Wa3; AF371939
			Bacteria	Firmicutes	Clostridia	Clostridiales	Veillonellaceae	Veillonella	Veillonella parvula (T); DSM 2008; X84005
			Bacteria	Firmicutes	Clostridia	Clostridiales	Veillonellaceae	Veillonella	Veillonella atypica; ATCC 17744; AF439641
			Bacteria	Firmicutes	Clostridia	Clostridiales	Veillonellaceae	Veillonella	Veillonella caviae (T); DSM 20738; AY355140
			Bacteria	Firmicutes	Clostridia	Clostridiales	Veillonellaceae	Veillonella	Veillonella parvula; CIP 60.1; AF439640
			Bacteria	Firmicutes	Clostridia	Clostridiales	Veillonellaceae	Veillonella	uncultured bacterium; BA05; AY985003
			Bacteria	Firmicutes	Clostridia	Clostridiales	Veillonellaceae	Veillonella	uncultured bacterium; B991; AY984994
			Bacteria	Firmicutes	Clostridia	Clostridiales	Veillonellaceae	Veillonella	uncultured bacterium; B608; AY984660
			Bacteria	Firmicutes	Clostridia	Clostridiales	Veillonellaceae	Veillonella	Veillonella dispar; ATCC 17748; AF439639
			Bacteria	Firmicutes	Clostridia	Clostridiales	Veillonellaceae	Veillonella	Veillonella sp. ADV 3107.03; AY571667
			Bacteria	Firmicutes	Clostridia	Clostridiales	Veillonellaceae	Veillonella	Veillonella rodentium; ATCC 17743; AY514996
o1784	o1784 16S-seq248(79)	AGATACGTCCCTCTCTTTGG	Bacteria	Firmicutes	Clostridia	Clostridiales	Veillonellaceae	Selenomonas	Selenomonas sp. oral clone AA024; AF287797
			Bacteria	Firmicutes	Clostridia	Clostridiales	Veillonellaceae	Selenomonas	uncultured Selenomonas sp.; 17BB68; FJ976364
			Bacteria	Firmicutes	Clostridia	Clostridiales	Veillonellaceae	Selenomonas	uncultured bacterium; nbu87h08c1; GQ006802
			Bacteria	Firmicutes	Clostridia	Clostridiales	Veillonellaceae	Selenomonas	uncultured Selenomonas sp.; 6BB012; FJ976245
